# Fused 3D boron heterocycles *via* EnT catalysis: synthesis, modification and validation as beta-lactamase inhibitors

**DOI:** 10.1039/d5sc05518k

**Published:** 2025-11-03

**Authors:** Hannah M. Kortman, Hao Fang, Kane A. C. Bastick, Charlotte Völkel, Dominik Oberthür, Peter H. Seeberger, Markus Perbandt, John J. Molloy

**Affiliations:** a Department of Biomolecular Systems, Max-Planck-Institute of Colloids and Interfaces Am Mühlenberg 1 14476 Potsdam Germany john.molloy@mpikg.mpg.de; b Department of Chemistry and Biochemistry, Freie Universität Berlin Arnimallee 22 14195 Berlin Germany; c Center for Free-Electron Laser Science CFEL, Deutsches Elektronen-Synchrotron DESY Notkestr. 85 22607 Hamburg Germany; d Department of Chemistry, Institute for Physical Chemistry, University of Hamburg Grindelallee 117 20146 Hamburg Germany markus.perbandt@uni-hamburg.de

## Abstract

The installation of a boron unit into bioactive scaffolds continues to unlock novel modes of molecular recognition in drug discovery. As such, *de novo* strategies to access 3D boron-containing frameworks, that modulate the intrinsic reactivity at boron, are being intensively pursued. Herein, we report a visible light-mediated energy transfer (EnT) catalysis strategy that enables the [2 + 2] cycloaddition of boron-containing heterocycles to construct 3D frameworks with high structural complexity. Leveraging both inter- and intramolecular cycloadditions, a suite of angularly fused boron heterocycles was accessed, offering enhanced steric shielding and modular handles for additional interactions. A boron deletion strategy permits the synthesis of benzofuran scaffolds, otherwise inaccessible *via* direct EnT. Crucially, the resulting 3D architectures mimic structural motifs found in the potent β-lactamase inhibitor Xeruborbactam. The biological relevance of these frameworks was validated by NMR titration, p*K*_a_ analysis, and co-crystallisation with serine β-lactamase CTX-M-14, revealing enantiospecific binding and a well-defined hydrogen bonding network. These results establish a versatile platform for the synthesis of functionalised boron heterocycles with direct translational potential in medicinal chemistry.

## Abstract 2

An energy transfer catalysis strategy enables [2 + 2] cycloadditions of boron heterocycles, yielding 3D frameworks that mimic β-lactamase inhibitors. These structures show promise for medicinal chemistry and can be used in boron deletion strategies.

## Graphical abstract

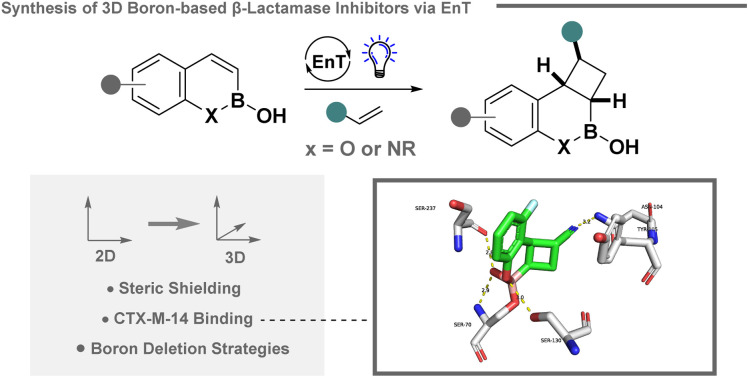

The strategic incorporation of boron as a design feature in drug candidates has vastly accelerated in recent years,^1^ primarily due to their unique ability to elicit site-specific interactions with biomolecules.^2^ Here, boron can serve as a biomolecular antenna, that when efficiently tuned, has the ability to undergo; hydrogen bonding *via* the Brønsted acidic OH moiety,^3^ covalent binding with serine residues of larger protein structures^4^ and diol conjugation of complex glycan scaffolds among other interactions (Fig. 1A).^5^ While the dexterity of boronic acids and hemiboronic esters has culminated in prominent advances in drug design, their propensity to engage P450 enzymes and undergo oxidative deboronation to form inactive metabolites,^6^ often precludes their general use as warheads in drug discovery campaigns. To surmount this challenge, drug candidates are often designed with features that aid the intrinsic stability of the boron moiety (Fig. 1B). For example, the blockbuster cancer therapeutic Bortezomib contains a strategically placed chiral benzylic arm that provides steric shielding.^7^ However, the most prevalent approach is embedding the boron moiety into a parent heterocycle with β-lactamase inhibitor, Xeruborbactam, as exemplar, demonstrating serine-specific binding.^8^ The inception of *de novo* boron heterocycle chemotypes, elegantly designed by the Raines and Hall groups,^6*c*,9^ further underscores boron's potential as a binding motif in drug discovery. Given the ability of boron to selectively engage biological targets, novel approaches to 3D boron heterocycles that aid stability and provide functional handles to potentially elicit additional interactions, would be highly enabling.

**Fig. 1:**
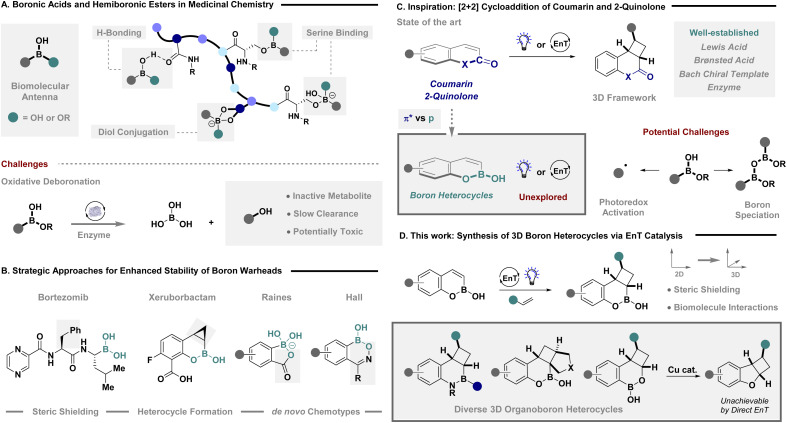
(A) Boronic acids and hemiboronic esters in medicinal chemistry; (B) strategies for enhancing boron stabilisation; (C) inspiration from EnT enabled [2 + 2] cycloaddition; (D) synthesis of 3D boron heterocycles *via* EnT catalysis and confirmation of their interactions with biomolecules.

The photoactivation of heterocycles *via* direct excitation or EnT catalysis has been leveraged with great effect to facilitate [2 + 2] cycloaddition reactions, enabling a transition from 2D to 3D chemical space (Fig. 1C).^10^ Intensive research on coumarin and quinolone systems^11^ have given rise to powerful Lewis^12^ and Brønsted acid activation modes,^13^ the venerable Bach chiral template activation,^14^ and more recently enzyme photocatalysis.^15^ Despite these prominent advances, the excited state properties and EnT photoactivation of boron heterocycles, such as benzoxaborines, remains conspicuously underexplored, which is striking given the recent prominent advances in [2 + 2] cycloaddition of borylated styrenes, dienes and simple alkenes achieved by Brown, Masarwa and our own research group.^16^ In addition, geometric isomerisation of similar substrates *via* sensitisation has also been efficiently demonstrated by Gilmour and coworkers.^17^ The current gap in the literature, regarding boron heterocycles, is potentially due to intractable challenges in speciation control of the B–OH bond,^18^ promoting undesired photoactivation of the product C(sp^3^)–B bond,^19^ or potential [2 + 2] dimerisation that suppresses the target reactivity.^12*a*^ The ability to surmount these challenges, to facilitate an efficient [2 + 2] cycloaddition, would provide direct access to highly coveted 3D boron heterocycles that are structurally analogous to the potent drug candidate Xeruborbactam. The ability to forge these analogues with strategically orientated exit vectors will enable further exploration of chemical space to elicit additional biomolecular interactions,^20^ for the design of functional molecules. Herein we describe the EnT catalysed intermolecular [2 + 2] cycloaddition of boron heterocycles to rapidly construct biologically relevant, fused 3D architectures (Fig. 1D). Translation to 3- and 4- substituted systems containing a tethered alkene enabled expedient access to complex angularly-fused boron heterocycles, notably increasing steric shielding around the boron centre. Leveraging a boron deletion strategy, of benzoxaborinine scaffolds, enabled a formal [2 + 2] cycloaddition of prohibitively high in energy unsubstituted benzofurans. The feasibility of these 3D architectures to emulate state of the art in boron therapeutics was validated by establishing p*K*_a_ profiles, NMR binding studies and confirming stability and solubility in aqueous media. Cognizant of the structural similarities to Xeruborbactam, the ability of our 3D frameworks to replicate biological activity was demonstrated *via* enantiospecific binding with the serine β-lactamase (SBL) CTX-M-14 from *Klebsiella pneumoniae*. Clear serine binding is validated in the enzyme pocket in combination with an unexpected H–bonding interaction of installed substituents on the cyclobutane backbone.

We commenced our reaction optimisation investigating the efficiency of [2 + 2] cycloaddition between benzoxaborine 1 and acrylonitrile (ACN), surveying a series of sensitisers with varying triplet excited state energies (Table 1).^21^ The use of a lower energy sensitiser, Ir(ppy)_3_, was unsuccessful providing low quantities of both cycloadduct 2 and dimer 3 (entry 1). Although the use of higher energy sensitisers enhanced EnT catalysis (entries 2–4), intriguingly, reaction output reflected the physical properties of the photocatalyst. Cationic iridium complexes led to significant dimerisation (entry 2), while homoleptic complex Ir(dFppy)_3_ and thioxanthone suppressed formation of 3. Judicious control of reaction media was critical for reactivity with methanol and THF leading to lower yields due to dimerisation, while the use of toluene enabled a significantly cleaner reaction profile (entries 5–7). Although high yields could be achieved using 5 equivalents of the cheap acrylonitrile coupling partner, the presence of significant amounts of dimer prevented efficient isolation *via* column chromatography. Increasing acrylonitrile (ACN) equivalents and dilution of the reaction afforded the target cycloadduct in 93% yield, with no dimer detectable by ^1^H NMR (entry 8, see SI for comprehensive reaction optimisation). Control reactions demonstrated that both light and catalyst are a prerequisite for the target reactivity (entries 9 and 10). Further studies support efficient EnT from the catalyst to all boron heterocycles (see SI for full details).

**Table 1:** Optimisation of reaction conditions^a^

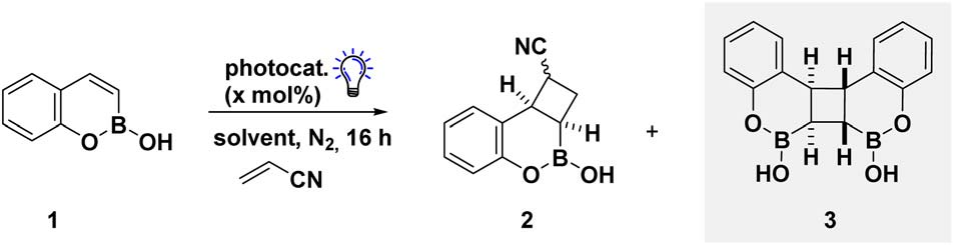						
Entry	Catalyst	*E*_T_ (kJ mol^−1^)	Solvent	ACN (equiv.)	2^b^ (%)	3^b^ (%)
1	Ir(ppy)_3_	231	MeCN	5	13	5
2	(Ir[dF(CF_3_)ppy]_2_(dtbpy))PF_6_	251	MeCN	5	52	40
3^c^	Thioxanthone	265	MeCN	5	77	22
4	Ir(dFppy)_3_	251	MeCN	5	84	15
5	Ir(dFppy)_3_	251	MeOH	5	66	30
6	Ir(dFppy)_3_	251	THF	5	75	23
7	Ir(dFppy)_3_	251	Toluene	5	85	15
8^d^	Ir(dFppy)_3_	251	Toluene	50	93	0
9^d^	No catalyst	—	Toluene	50	0	0
10^e^	Ir(dFppy)_3_	251	Toluene	50	0	0

a Standard conditions: 1 (0.2 mmol, 1 equiv.), ACN (*x* equiv.), photocat (1 mol%), blue LEDs (1 W), solvent (0.05 M), rt, 16 h.

b Determined by ^1^H NMR spectroscopy against a known internal standard (1,3,5-trimethoxybenzene).

c Reaction carried out using 5 mol% catalyst and 390 nm light (5 W).

d Reaction was carried out at 0.025 M concentration.

e Reaction carried out in the absence of light irradiation and at 0.025 M concentration.

Having demonstrated efficient EnT catalysis of benzoxaborine scaffold 1, the scope and limitations were established to develop a series of 3D boron heterocycles (Scheme 1). Initially, the impact of modifying the alkene component was investigated, demonstrating reactivity with electron poor alkenes to obtain a single regioisomer, albeit with poor diastereoselectivity (2, 4 and 5). Structural modifications of the aryl ring were tolerated for electron-rich (6–8) and electron-poor derivatives (9–12) enabling clean isolation of a single major diastereoisomer. Transition to benzazaborines (13–17), also synonymous in medicinal chemistry,^22^ slightly improved diastereoselectivity, presumably due to differences in stereoelectronics of the parent heterocycle. It is pertinent to note heterocycle dimerisation was less problematic and lower quantities of alkene could be used in these cases. The use of comparatively electron rich alkenes was permitted, leading to a decrease in diastereoselectivity (18), while reactivity with heterocycle modifications (19 and 20) and in the absence of the C_(aryl)_–B functionality (21) were also permitted. Simple boron–oxygen exchange with benzoxaborinine scaffolds was well tolerated with a significant increase in diastereoselectivity (22–25). Although comparatively electron rich alkenes eroded diastereoselectivity (26), incorporation of substituents on the core heterocycle was feasible under model reaction conditions (27 and 28), as were the use of acrylamides (29 and 30). Despite concerted efforts, the use of styrenes led to significant polymerisation under model reaction conditions (see SI for full details of unsuccessful substrates).

**Scheme 1:**
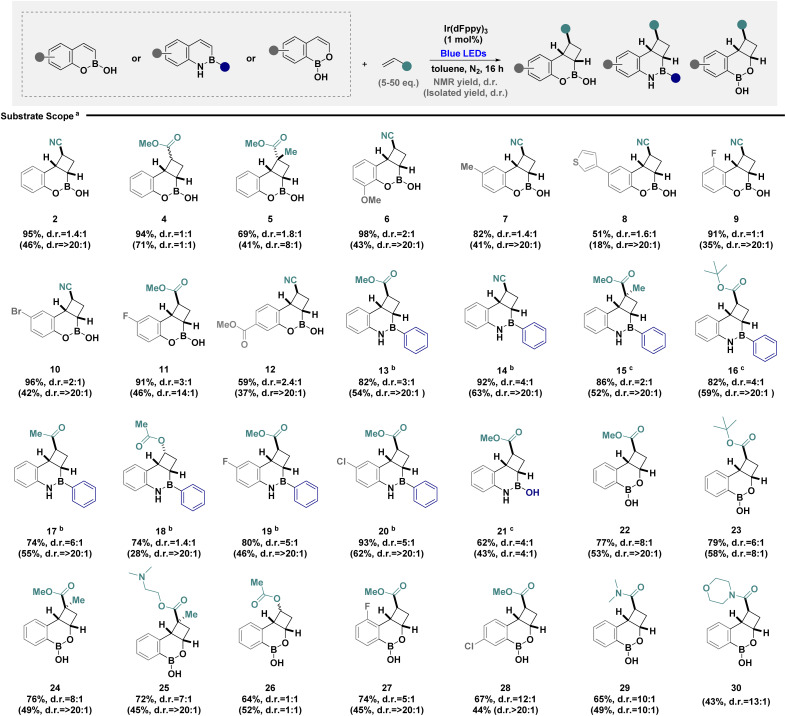
Exploring the substrate scope. ^*a*^Reactions were performed in toluene on a 0.2 mmol scale using boron heterocycle (1 equiv.), alkene (50 equiv.), and Ir(dFppy)_3_ (1 mol%) under blue LED irradiation (1 W) for 16 hours. NMR yield and d.r. was determined by ^1^H NMR spectroscopy against a known internal standard (1,3,5-trimethoxybenzene). Differences between NMR and isolated yields are due to targeted isolation of the major diastereomer as a single isomer; ^*b*^ the reaction was run in THF using 5 equivalents of alkene; ^*c*^the reaction was run in THF using 20 equivalents of alkene.

Efficient sensitisation of non-substituted benzofurans is an intractable challenge due to their prohibitively high triplet excited state energy (301 kJ mol^−1^).^23^ Elegant studies by Sheppard and coworkers have demonstrated the efficient intramolecular Chan–Lam reaction of saturated boron heterocycles.^24^ This reaction could be strategically leveraged to facilitate a formal [2 + 2] cycloaddition of benzofurans *via* a boron deletion strategy (Scheme 2). Using catalytic copper(ii) acetate, cycloadducts containing quaternary centres (31 and 36), aromatic substituents and C–O bonds could be efficiently forged in a single step (32–35).

**Scheme 2:**
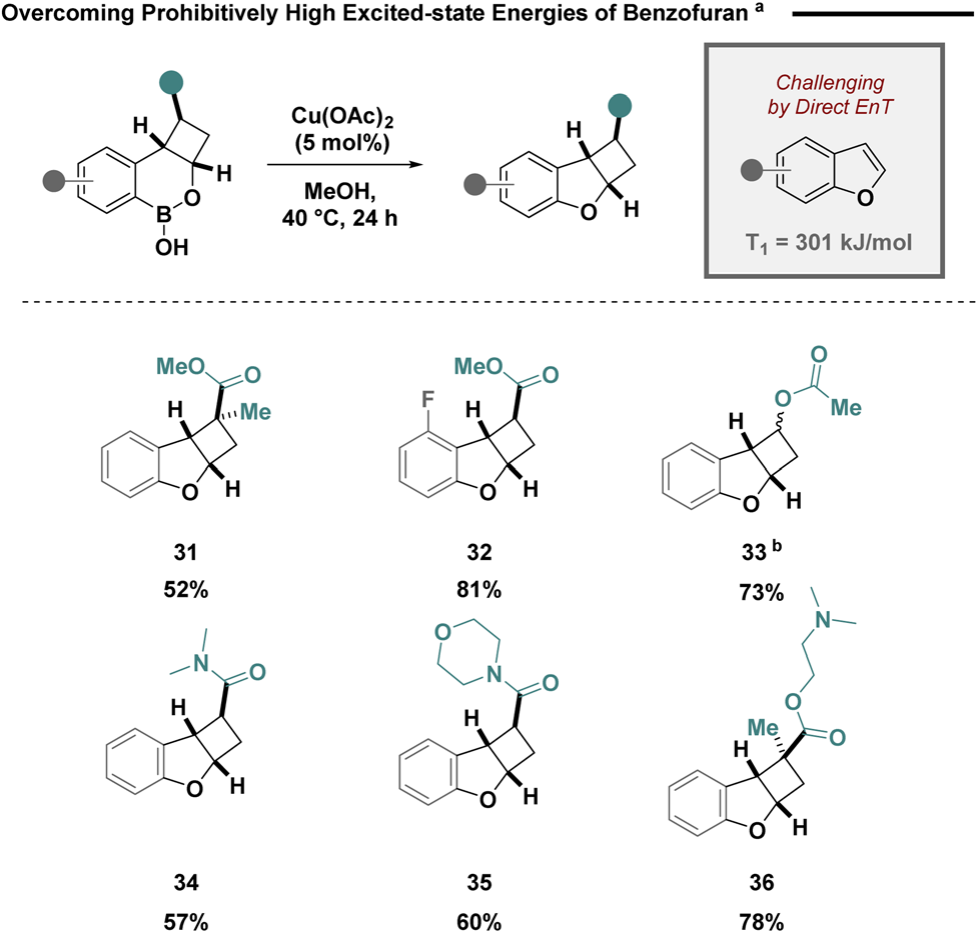
Intramolecular Chan–Lam coupling enabling a formal [2 + 2] cycloaddition of benzofurans. ^*a*^Boron heterocycle (1 equiv.), Cu(OAc)_2_ (5 mol%), MeOH, 40 °C, 24 h; ^*b*^a 1 : 1 mixture of diastereomers was used as starting material. Both diastereomer products could be isolated cleanly in a 1 : 1 ratio.

Given our initial rationale, that steric shielding can aid the intrinsic stability of boron warheads, intramolecular [2 + 2] cycloaddition was next probed to provide angularly-fused tetracyclic scaffolds (Scheme 3). Access to 3-tethered precursors could be readily achieved in a single step employing a cascade regioselective Suzuki–Miyaura/cyclisation protocol,^25^ while 4-tethered analogues could be synthesised using haloboration and subsequent cross-coupling of *o*-alkyl phenols, developed by Ingleson and coworkers.^26^ The use of model reaction conditions enabled efficient cyclisation producing tetracyclic substrates (37–40) with substitution on the tethered backbone. Pleasingly, Xeruborbactam analogues (41 and 42) could be accessed in high yield. Employing 4-tethered systems was also well tolerated providing expedient access to boron heterocycle 43.

**Scheme 3:**
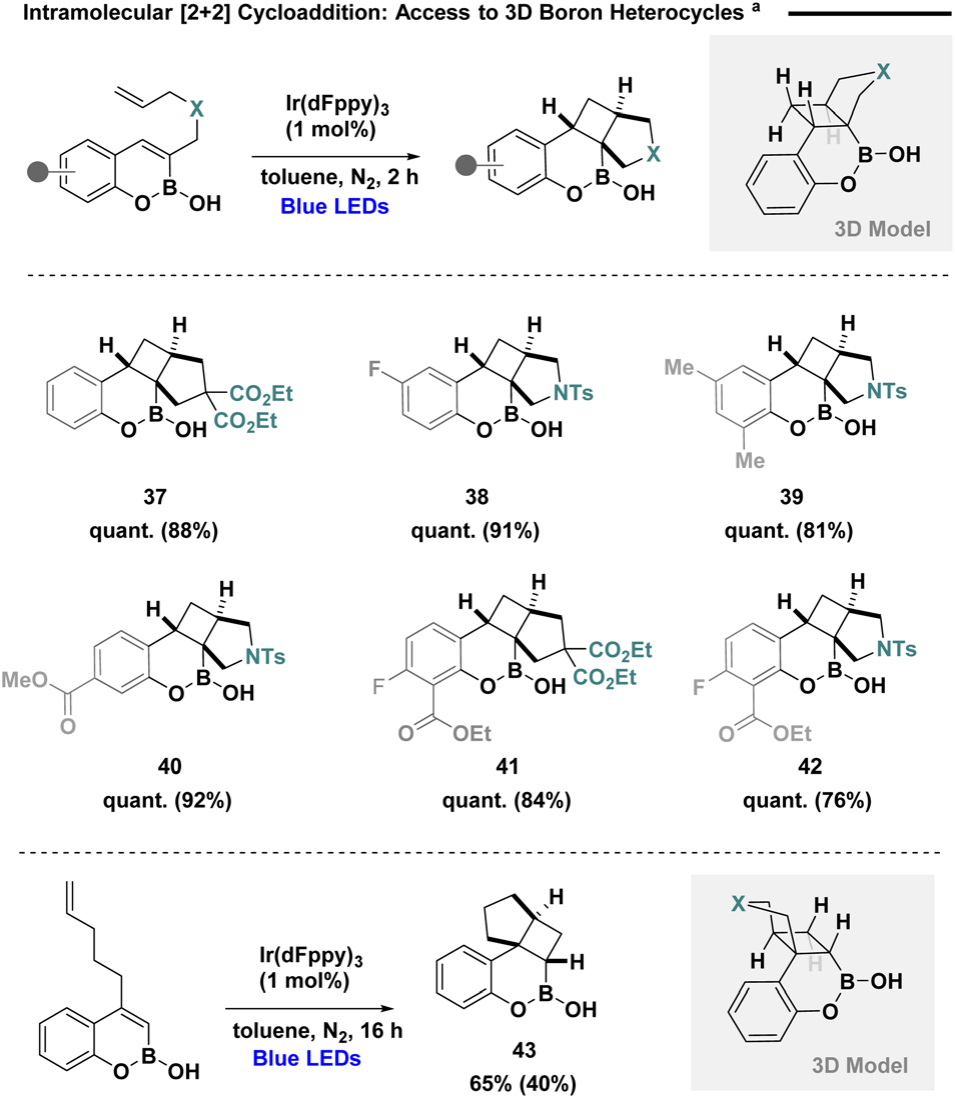
^*a*^Reactions were performed in toluene on a 0.1 mmol scale using boron heterocycle (1 equiv.), and Ir(dFppy)_3_ (1 mol%) under blue LED irradiation (1 W) for 2–16 hours. NMR yield was determined by ^1^H NMR spectroscopy against a known internal standard (1,3,5-trimethoxybenzene).

As the overarching goal of this investigation was to not only provide expedient access to saturated 3D boron heterocycles but to demonstrate their ability to emulate state of the art in boron-based therapeutics,^1,2^ we looked to explore their properties as medicinal chemistry fragments (Fig. 2). Hemiboronic esters are renown for their propensity to engage sugars *via* diol conjugation and serine residues *via* dynamic covalent binding. Inspired by seminal reports by Hall and others,^5*a*,*b*,27^ we first probed complexation with d-(−)-fructose (Fig. 2A). Titrating a solution of the saccharide in the presence of boron scaffold 2, demonstrated that with increasing concentration a signal shift and broadening of signals adjacent to the hemiboronic ester centre was observed. This result clearly indicates efficient complexation of the sugar motif. Translation to an oligopeptide containing a pendent serine reside was also successful in displaying signal broadening synonymous with dynamic covalent binding between B–OH and serine (Fig. 2B, see SI for full details). Intriguingly, model compound 2 could be stored in D_2_O at physiological pH for one week without any substrate degradation (see SI for a comprehensive stability study). Hall and coworkers have elegantly tracked p*K*_a_ using ^11^B NMR to accurately predict boron's ability to fluctuate between hybridisation states at physiological pH.^5*a*^ It was anticipated that transitioning from an unsaturated heterocycle that displays aromatic character, to a saturated analogue would enhance boron's ability to undergo hybridisation and engage biomolecule residues. Precursor 1, a 2D boron heterocycle, was titrated with a p*K*_a_ measured at 8.6 using ^11^B NMR (Fig. 2C). Intriguingly, transitioning to the 3D boron heterocycle led to a decrease in p*K*_a_ (4.4), suggesting these substrates have the ability to covalently bind serine residues at physiological pH.^28^

**Fig. 2:**
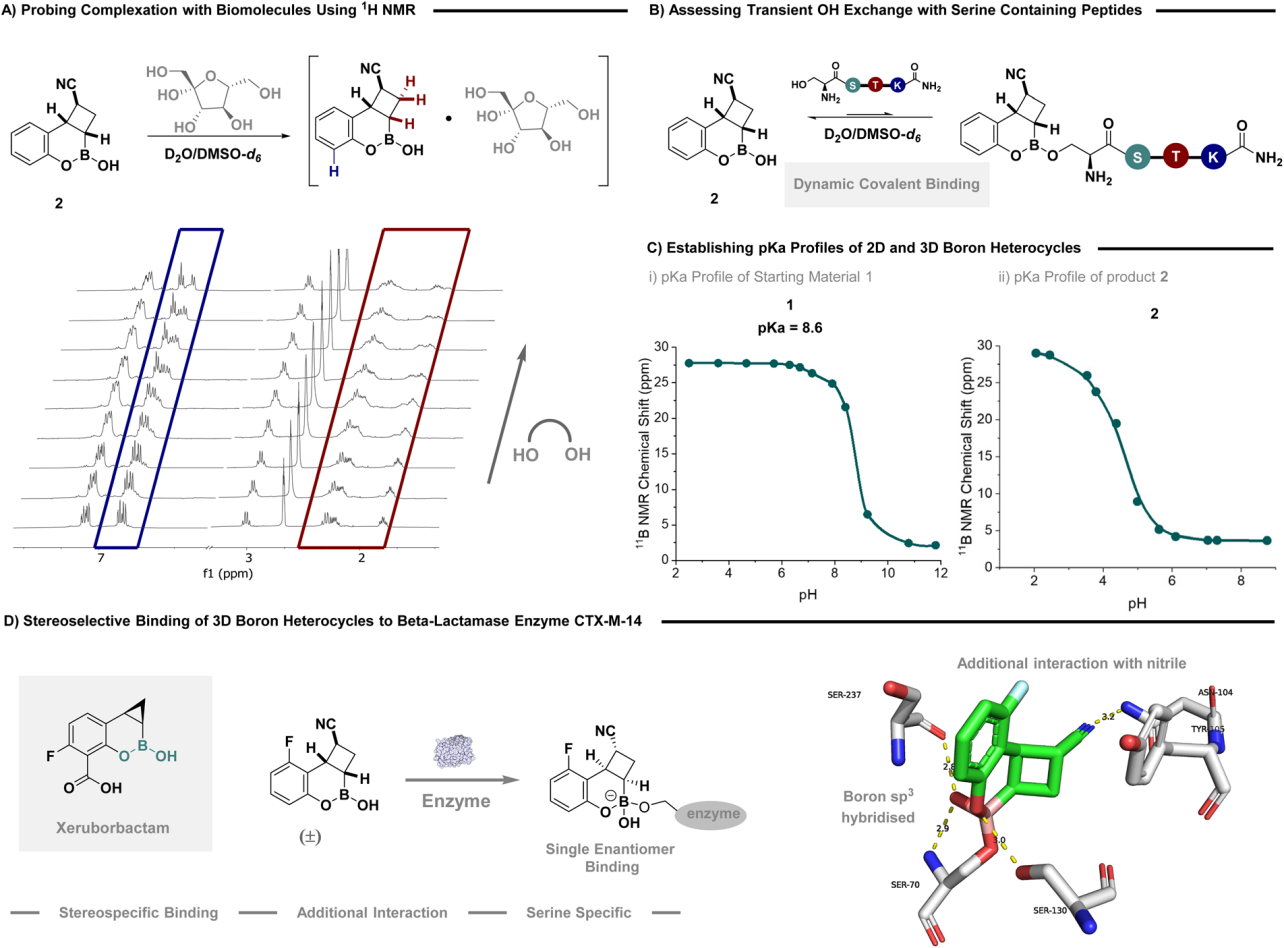
Exploring the properties of 3D boron heterocycles. (A) Probing complexation with biomolecules using ^1^H NMR; (B) monitoring hydroxyl exchange of serine containing oligopeptides; (C) comparing p*K*_a_ profiles of 2D and 3D boron heterocycles; (D) co-crystalisation of compound 9 in the binding pocket of β-lactamase enzyme CTX-M-14.

Finally, to support the possibility for future applications in medicinal chemistry, we sought to co-crystalise substrates in serine β-lactamase CTX-M-14, a major lactamase enzyme (Fig. 2D).^4*c*,29^ A single enantiomer of racemic compound 9 was shown to efficiently bind in the active site. The inhibitor forms a covalent bond with Ser70, yielding a stable tetrahedral adduct that mimics the transition state for β-lactam hydrolysis, similar to Xeruborbactam and other β-lactamase inhibitors. This covalent complex is a hallmark of boronate-based β-lactamase inhibitors and crucial for their mechanism of action.^30^ The bound ligand exhibits defined stereochemistry at all four stereocentres: three carbon atoms, established by the core heterocycle, and upon binding, the boron atom, which becomes a stereocentre due to its transition from trigonal-planar to tetrahedral hybridisation. This demonstrates that a single enantiomer, of the two potential isomers, binds the enzyme efficiently. The crystal structure reveals that all four stereocentres adopt the *R*-configuration. Despite being administered as an enantiomeric mixture, the enzyme binds and stabilises exclusively the (*R*,*R*,*R*,*R*)-configured isomer, highlighting the stereospecific nature of the interaction. This highly specific binding is reinforced by a network of hydrogen bonds. The boronate hydroxyl group forms three critical hydrogen bonds: with the backbone amide of Ser70 and the side chains of Ser130 and Ser237, occupying the enzyme's oxyanion hole. Intriguingly, the nitrile group, installed by the enclosed methodology, engages in a directional hydrogen bond with Asn104, further anchoring the ligand. Tyr105 is also located nearby and may contribute *via* polar or π-interactions. Additionally, the rigid bicyclic and aromatic scaffold is neatly embedded in a hydrophobic pocket, supporting shape complementarity. Together, covalent anchoring, extensive hydrogen bonding, and defined stereochemistry make this compound a potent and selective CTX-M-14 inhibitor. These structural insights emphasise the importance of stereochemistry and geometry in β-lactamase inhibition and offer valuable guidance for designing next-generation inhibitors with enhanced affinity and selectivity.

## Conclusions

In summary, we have established an EnT catalysis platform to access fused 3D organoboron frameworks *via* intermolecular and intramolecular [2 + 2] cycloaddition of boron-containing heterocycles. Benzoxaborinines could be efficiently functionalised *via* a Chan–Lam reaction to permit a formal [2 + 2] of prohibitively high in energy non-substituted benzofurans. Crucially, the resultant frameworks demonstrate enhanced stability and steric shielding at the boron centre and enable functionalisation vectors conducive to biomolecular engagement. The ability of these motifs to emulate the reactivity of state-of-the-art boron therapeutics was validated through rigorous analysis, including p*K*_a_ analysis, stability under physiological conditions, and serine-specific covalent binding. Co-crystallographic analysis with β-lactamase CTX-M-14 confirms precise stereochemical recognition and key directional hydrogen bonding interactions, underscoring the therapeutic promise of these 3D boron chemotypes. Collectively, these findings provide a blueprint for the design of next-generation boron-based inhibitors to access stereochemically defined boron scaffolds with direct translational relevance in medicinal chemistry. Given the enantiospecific nature of enzyme recognition, strategies which render the current methodology enantioselective are currently under investigation.

## Author contributions

H. M. K., H. F., K. A. C. B., C. V., D. O., P. H. S., M. P. & J. J. M. planned the experiments. H. M. K., H. F., K. A. C. B., C. V., D. O. & M. P. performed the experiments. All authors contributed to the analysis and interpretation of the data. M. P. & J. J. M. directed the project and wrote the manuscript with contributions from all authors.

## Conflicts of interest

There are no conflicts to declare.

## Supplementary Material

SC-017-D5SC05518K-s001

SC-017-D5SC05518K-s002

## Data Availability

The data supporting this article have been included as part of the supplementary information (SI). Supplementary information is available. See DOI: https://doi.org/10.1039/d5sc05518k.
