# Supplementary material for: Fused 3D boron heterocycles via EnT catalysis: synthesis, modification and validation as beta-lactamase inhibitors
Source: Chem Sci. 2025 Nov 3;17(1):247–54. doi: 10.1039/d5sc05518k (PMC12599387; doi:10.1039/d5sc05518k)
Supplement: SC-017-D5SC05518K-s002 [file SC-017-D5SC05518K-s002.pdf]

Supplementary Information (SI) for Chemical Science.
This journal is © The Royal Society of Chemistry 2025

Supplementary Information for

Fused 3D Boron Heterocycles via EnT Catalysis: Synthesis, Modification and Validation as Beta-Lactamase Inhibitors

Hannah M. Kortman, Hao Fang, Kane A. C. Bastick, Charlotte Völkel, Dominik Oberthür, Peter H. Seeberger, Markus Perbandt, and John J. Molloy

Table of Contents

General Information S3
Starting Material Synthesis S4
Experimental Setup for Photoreactions S24
Reaction Optimisation S26
Substrate Scope S30
- Assignments of Diastereomers S51
- Study on Effect of Decreased Equivalents of Alkenes S53
- Unsuccessful Substrates S53

Reaction Probes and Studies to Support EnT S56
- Dimerisation S56
- UV/Vis Analysis S57
- Stern–Volmer Quenching Study S61
- Proposed Mechanism S62

Derivatisation and Scale-Up S63
- Derivatisation S63
- Scale-Up Reactions S66

Testing Medicinal Relevance S68
- Binding Studies S68
- Experimental p*K*a Values S71
- FIA Predictions S73
- Stability in D_2O S74

Crystal Data and Structure Refinement S75
References S79
NMR Data S82

General Information

All chemicals were purchased as reagent grade and used without further purification unless stated otherwise. Anhydrous solvents were obtained by passing solvents through activated alumina columns and storing them over activated 4 Å molecular sieves for 24 h prior to use. Degassed solvent refers to bubbling argon through the solvent for a minimum of 15 minutes. Solvents for purification (extraction and chromatography) were purchased as technical grade and distilled on the rotary evaporator prior to use. For column chromatography SiO_2 (40-63 µm for flash chromatography, Macherey Nagel or VWR) and C18-(50 µm Büchi FlashPure EcoFlex 4 g and 12 g) were used as a stationary phase. Analytical thin layer chromatography (TLC) was performed on pre-coated TLC sheets ALUGRAM® XtraSIL G/UV254 (Macherey Nagel). UV light (254 nm), potassium permanganate ($KMnO_4$), vanillin and *p*-anisaldehyde stain solutions were used for visualisation. In case R_f-values were not stated for unreported compounds, the compounds were not stable on silica and R_f-values could not be measured. Concentration under reduced pressure was performed at ~10 mbar and 40 °C, drying at ~10–2 mbar and ambient temperature. NMR spectra were measured on either a Varian 400 MHz, Bruker Ascend 400 MHz, Varian 600 MHz or Bruker Ascend 700 MHz at ambient temperature. The chemical shifts are referenced to the residual solvent peak as internal standard and are reported in ppm. The resonance multiplicity is abbreviated as: s (singlet), d (doublet), t (triplet), q (quartet), quint (quintet), m (multiplet) and br (broad). Assignments of unknown compounds are based on APT, DEPT, COSY(HH), HMBC, HSQC and NOESY spectra. Carbon atoms bearing boron were not observed by ^{13}C NMR and are not reported. High-resolution mass spectra were measured using a Waters Xevo G2-XS QTof or by the MS service of Freie Universität Berlin. Analysis and purification by normal and reverse phase HPLC were performed by using the Agilent 1200 series. Products were lyophilised using a Christ Alpha 2-4 LD plus freeze dryer. UV/Vis spectra were recorded using a Shimadzu UV-1900 I spectrophotometer. The samples were prepared and recorded in quartz cuvettes with a pathlength of 1 cm. R spectra were recorded on a Perkin- Elmer Spectrum 100 FT-IR spectrometer, selected adsorption bands are reported in wavenumbers (cm^{-1}). Photoreactions were performed using Kessil® PR-160L lamps (390 and 440 nm) and WINGER® WEPRB1-S1 Power LED Star royal blue (450nm) LEDs. Emission profiles of the LEDs were obtained using a Broadcom Qmini Spectrometer. Photoreaction setup, including light source emission is comprehensively described (*vide infra*). Stern–Volmer Quenching studies were preformed using a Horiba FluoroMax 4 spectrofluorometer. pH values were obtained using a VWR® pHenomenal® pH 1100L and Mettler Toledo Severn Direct SD20 and the InLab® NMR electrode.

Starting Material Synthesis

General Procedure A: Reduction with Iron

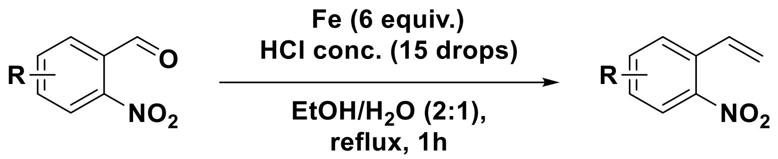

To a round-bottom-flask was added the corresponding benzaldehyde (1 equiv.), iron (6 equiv.), and HCl (15 drops, conc.). To this was added EtOH (40 mL) and water (20 mL) and the flask was equipped with a reflux condenser. The reaction mixture was refluxed at 90 °C for 1 h before it was allowed to cool to ambient temperature. Subsequently, the mixture was diluted with EtOAc (50 mL) and filtered over celite. The filtrate was washed with sat. aq. Na_2CO_3 (60 mL) and the aqueous phase was back extracted with EtOAc (2 × 50 mL). The combined organic phases were dried over Na_2SO_4 and concentrated under reduced pressure. The desired product was used without further purification.

General Procedure B: Wittig olefination

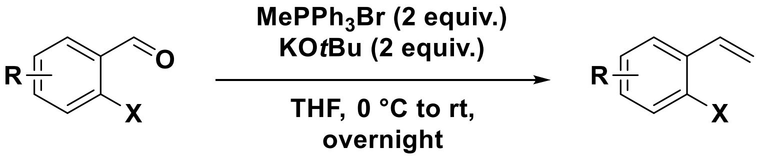

To a flame-dried Schlenck-flask was added methyltriphenylphosphonium bromide (2 equiv.) before purging with nitrogen (3 ×). To the flask was added anhydrous THF (0.1 M) before the portion-wise addition of KO*t*Bu (2 equiv.) at 0 °C. The mixture was stirred for 1 h at ambient temperature before it was cooled to 0 °C again. Then, the corresponding benzaldehyde was added dropwise at 0 °C. In case the benzaldehyde was solid, it was dissolved in anhydrous THF (10 mL) before the dropwise addition. After complete addition, the mixture was stirred at ambient temperature overnight. Upon completion, the reaction was quenched with sat. aq. NH_4Cl (50 mL) and extracted with EtOAc (3 × 40 mL). The combined organic phases were dried over Na_2SO_4 and concentrated under reduced pressure. The crude mixture was purified by flash column chromatography to yield the desired product.

General Procedure C: Borylation

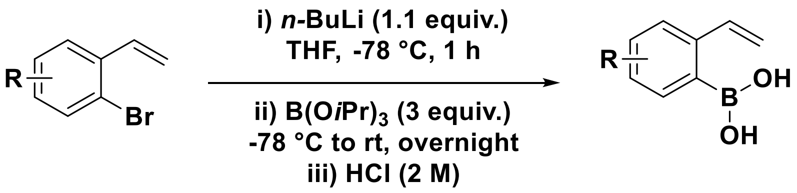

According to literature by Sheppard *et al.*,[7] to an oven-dried round-bottom-flask was added the corresponding bromo-styrene (1 equiv.) and the flask was sealed and purged with nitrogen (3 ×) before the addition of anhydrous THF (0.15 M) *via* syringe. The mixture was cooled to -78 °C before the dropwise addition of *n*-BuLi (2.5 M in hexanes, 1.1 equiv.). The reaction was stirred for 1 h at -78 °C before the addition of triisopropylborate (3 equiv.) and the mixture was subsequently allowed to warm to ambient temperature overnight. Upon completion, the reaction was quenched with HCl (2 M, 50 mL) and extracted with Et_2O (3 × 50 mL). The combined organic phase was dried over Na_2SO_4 and

concentrated under reduced pressure. Purification by flash column chromatography yielded the desired product.

General Procedure D: Formation of Benzoxaborines

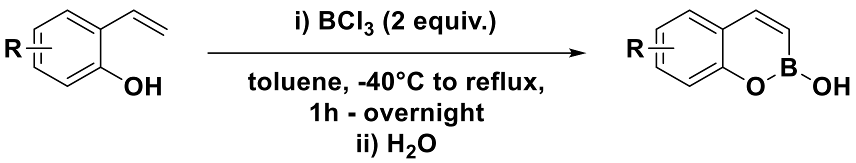

To an oven-dried 2-neck round-bottom-flask, equipped with an oven-dried reflux condenser, was added the corresponding hydroxy styrene (1 equiv.) before purging with nitrogen (3 ×). Subsequently, anhydrous toluene was added via syringe (0.1 M) and the mixture was cool to -30 °C (MeCN/dry ice bath) before the dropwise addition of boron trichloride (1.0 M in hexanes, 2.0 equiv.). The reaction mixture was allowed to warm to ambient temperature over 1 h before it was brought to reflux. The reaction mixture was allowed to cool to ambient temperature upon full conversion of the starting material (determined by TLC). Water (100 mL) was added to quench the reaction and the organics were extracted with EtOAc (3 × 80 mL). The combined organic phases were dried over Na_2SO_4 and concentrated under reduced pressure. Flash column chromatography yielded the desired product.

General Procedure E: Formation of Benzazaborines

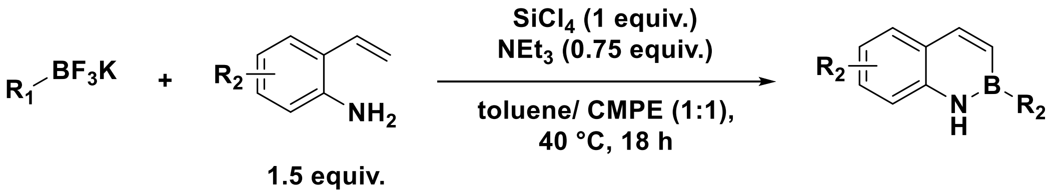

The reaction was performed according to literature procedure of Molander *et al.*[11] To an oven-dried microwave vial was added the corresponding potassium trifluoroborate (1 equiv.). The vial was sealed and purged with nitrogen (3 ×) before the addition of toluene (1 M) and corresponding aniline (1.5 equiv.), dissolved in CMPE (1 M). Subsequently, silicon tetrachloride (1 equiv.) and triethylamine (0.75 equiv.) were added before the mixture was stirred for 18 h at 40 °C. Upon completion the reaction was allowed to cool to ambient temperature before the addition of *n*-hexane (20 mL). The mixture was purified via filtration over a 2 cm silica plug and eluted with 10 % DCM/*n*-hexanes.

General procedure F: Synthesis of Benzoxaborinines

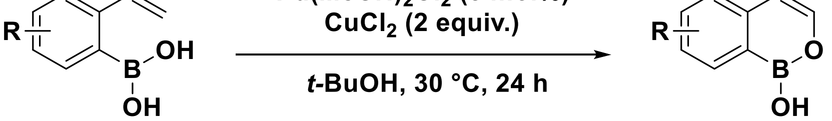

According to literature procedure by Sheppard *et al.*,[7] to a round-bottom-flask was added the corresponding vinyl phenylboronic acid (1 equiv.), $Pd(CH_3CN)_2Cl_2$ (5 mol%) and anhydrous copper (II) chloride (2 equiv.). Subsequently, was added *t*-BuOH (0.04 M) and the resulting mixture was stirred at 30 °C for 24 h. Upon completion the crude mixture was concentrated under reduced pressure and flash column chromatography yielded the desired product.

General Procedure G: Synthesis of intramolecular Starting Materials

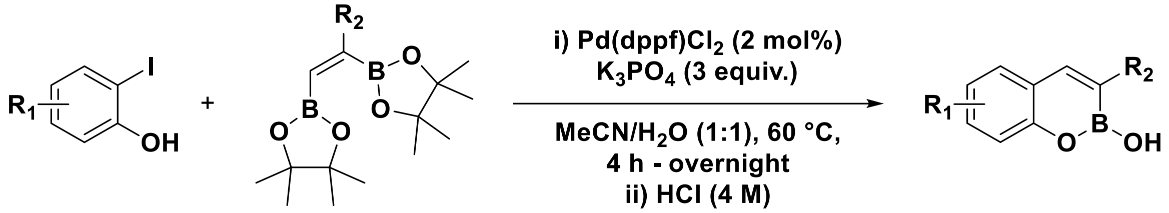

According to literature procedure by Molloy *et al.*,[14] to a 20 mL microwave vial were added the respective 2-iodophenol (1 equiv.), the respective vicinal diboron alkene (1.5 equiv.), Pd(dppf)Cl_2 (2 mol%) and potassium phosphate (3 equiv.). The vail was sealed and purged with nitrogen (3 ×). Subsequently, degassed MeCN and degassed water (1:1, 0.2 M) were added via syringe. The reaction mixture was stirred for 4 h at 60 °C and was subsequently quenched with HCl (4 M, 120 mL) for 10 min. The organics were extracted with DCM (3 × 80 mL) and the combined organic phases were concentrated under reduced pressure. Purification via flash column chromatography (SiO_2, 0%→10% Acetone/*n*-hexane) yielded the desired product.

2-Amino-5-fluorobenzaldehyde (S1)

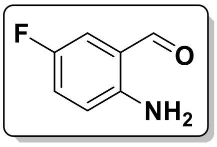

Prepared according to General Procedure **A**, 5-fluoro-2-nitrobenzaldehyde (1.54 g, 9.1 mmol) was converted to **S1**, yielding a yellow oil (1.23 g, 8.9 mmol, 97 %).

^{1}H NMR (400 MHz, $CDCl_3$) δ = 9.81 (s, 1H), 7.17 (dd, *J* = 8.4, 3.0 Hz, 1H), 7.09 (ddd, *J* = 9.0, 7.9, 3.0 Hz, 1H), 6.62 (dd, *J* = 9.0, 4.1 Hz, 1H), 5.99 (s, 2H) ppm.
Analytical data in agreement with literature.[1]

2-Amino-5-chlorobenzaldehyde (S2)

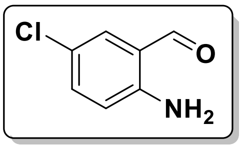

Prepared according to General Procedure **A**, 5-chloro-2-nitrobenzaldehyde (4.14 g, 22.3 mmol) was converted to **S2**, yielding a yellow oil (1.57 g, 10.1 mmol, 45%).

^{1}H NMR (400 MHz, $CDCl_3$) δ = 9.81 (s, 1H), 7.45 (d, *J* = 2.5 Hz, 1H), 7.28 – 7.23 (m, 1H), 6.62 (d, *J* = 8.8 Hz, 1H), 6.12 (s, 2H) ppm.
Analytical data in agreement with literature.[1]

2-Vinylphenol (S3)

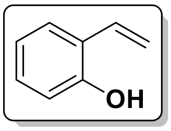

Prepared according to General Procedure **B**, salicylaldehyde (4.26 mL, 40 mmol, 1 equiv.) was converted to **S3**, yielding a colourless oil (4.83 g, 40 mmol, quant.) after flash column chromatography (SiO_2, 0→20% EtOAc/*n*-hexane).

^{1}H NMR (400 MHz, $CDCl_3$) δ = 7.39 (dd, *J* = 7.7, 1.7 Hz, 1H), 7.14 (td, *J* = 7.7, 1.7 Hz, 1H), 6.99 – 6.88 (m, 2H), 6.79 (dd, *J* = 8.1, 1.2 Hz, 1H), 5.74 (dd, *J* = 17.7, 1.4 Hz, 1H), 5.36 (dd, *J* = 11.2, 1.4 Hz, 1H), 5.05 (s, 1H) ppm.
Analytical data in agreement with literature.[2]

2-Methoxy-6-vinylphenol (S4)

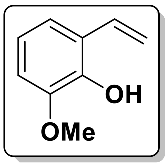

Prepared according to General Procedure **B**, 2-hydroxy-3-methoxybenzaldehyde (4.57 g, 30.0 mmol, 1 equiv.) was converted to **S4**, yielding a colourless solid (4.07 g, 27.0 mmol, 90 %) after purification by flash column chromatography (SiO_2, 0→20% EtOAc/*n*-hexane).

^{1}H NMR (400 MHz, $CDCl_3$) δ = 7.07 (dd, *J* = 7.7, 1.7 Hz, 1H), 7.01 (dd, *J* = 17.8, 11.2 Hz, 1H), 6.86 – 6.70 (m, 2H), 5.88 (s, 1H), 5.80 (dd, *J* = 17.8, 1.5 Hz, 1H), 5.30 (dd, *J* = 11.2, 1.5 Hz, 1H), 3.90 (s, 3H) ppm.
Analytical data in agreement with literature.[3]

4-Methyl-2-vinylphenol (S5)

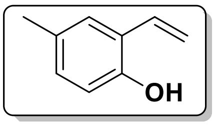

Prepared according to General Procedure **B**, 2-hydroxy-5-methylbenzaldehyde (413 mg, 3 mmol, 1 equiv.) was converted to **S5**, yielding a colourless oil (389 mg, 2.90 mmol, 97 %) after purification by flash column chromatography (SiO_2, 0→10% EtOAc/*n*-hexane).

^{1}H NMR (400 MHz, $CDCl_3$) δ = 7.19 (d, *J* = 2.2 Hz, 1H), 6.96 – 6.87 (m, 2H), 6.69 (d, *J* = 8.1 Hz, 1H), 5.72 (dd, *J* = 17.7, 1.4 Hz, 1H), 5.34 (dd, *J* = 11.2, 1.4 Hz, 1H), 4.80 (s, 1H) ppm.
Analytical data in agreement with literature.[4]

3-Fluoro-2-vinylphenol (S6)

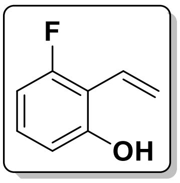

Prepared according to General Procedure **B**, 2-fluoro-6-hydroxybenzaldehyde (981 mg, 7 mmol, 1 equiv.) was converted to **S6**, yielding a colourless oil (406.4 mg, 2.94 mmol, 42 %) after purification by flash column chromatography (SiO_2, 0→10% EtOAc/*n*-hexane).

^{1}H NMR (600 MHz, $CDCl_3$) δ = 7.07 (tdd, *J* = 8.2, 6.4, 1.8 Hz, 1H), 6.78 (dd, *J* = 18.2, 11.8 Hz, 1H), 6.68 – 6.62 (m, 2H), 5.89 (dd, *J* = 18.1, 1.6 Hz, 1H), 5.62 (dd, *J* = 11.8, 1.7 Hz, 1H), 5.42 (s, 1H) ppm.
Analytical data in agreement with literature.[5]

4-Bromo-2-vinylphenol (S7)

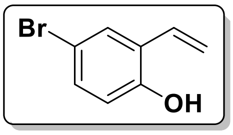

Prepared according to General Procedure **B**, 5-bromo-2-hydroxybenzaldehyde (4.02 g, 20 mmol, 1 equiv.) was converted to **S7**, yielding a white solid (1.99 g, 10.0 mmol, 50 %) after purification by flash column chromatography (SiO_2, 0→20% EtOAc/*n*-hexane).

^{1}H NMR (400 MHz, CDCl3) δ = 7.49 (d, J = 2.4 Hz, 1H), 7.23 (dd, J = 8.5, 2.4 Hz, 1H), 6.85 (dd, J = 17.7, 11.2 Hz, 1H), 6.68 (d, J = 8.6 Hz, 1H), 5.74 (dd, J = 17.7, 1.1 Hz, 1H), 5.40 (dd, J = 11.2, 1.1 Hz, 1H), 4.95 (s, 1H) ppm.
Analytical data in agreement with literature.[2]

4-Fluoro-2-vinylphenol (S8)

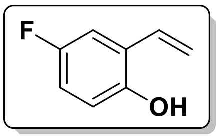

Prepared according to General Procedure **B**, 5-fluoro-2-hydroxybenzaldehyde (4.0 g, 28.5 mmol, 1 equiv.) was converted to **S8**, yielding as a colourless oil (3.72 g, 26.9 mmol, 94 %) after purification by flash column chromatography (SiO_2, 0→20% EtOAc/*n*-hexane).

^{1}H NMR (400 MHz, $CDCl_3$) δ = 7.09 (dd, *J* = 9.4, 3.1 Hz, 1H), 6.94 – 6.81 (m, 2H), 6.73 (dd, *J* = 8.8, 4.6 Hz, 1H), 5.73 (dd, *J* = 17.6, 1.1 Hz, 1H), 5.40 (dd, *J* = 11.1, 1.1 Hz, 1H), 4.79 (s, 1H) ppm.
Analytical data in agreement with literature.[3]

Methyl 3-hydroxy-4-vinylbenzoate (S9)

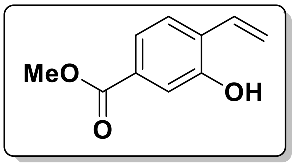

To a round-bottom-flask was added methyl 3-hydroxy-4-iodobenzoate (1.67 g, 6 mmol, 1 equiv.), potassium vinyltrifluoroborate (1.21 g, 9 mmol, 1.5 equiv.), Pd(dppf)Cl_2▪DCM (244 mg, 0.3 mmol, 5 mol%) and Cs_2CO_3 (5.86 g, 18 mmol, 3 equiv.) and the flask was sealed and purged with nitrogen (3 ×) before degassed THF (10 mL) and degassed water (1 mL) were added via syringe. The reaction was stirred at 60 °C overnight. Upon completion, the mixture was cooled to ambient temperature and EtOAc (20 mL) was added. The phases were separated and the aqueous phase was extracted with EtOAc (3 × 20 mL) and the combined organic phases were dried over Na_2SO_4 and concentrated under reduced pressure. Purification by flash column chromatography (SiO_2, 20% EtOAc/*n*-hexane) yielded **S9** as a white solid (637 mg, 3.58 mmol, 60%).

^{1}H NMR (400 MHz, $CDCl_3$) δ = 7.61 – 7.55 (m, 2H), 7.46 (d, *J* = 8.1 Hz, 1H), 7.00 (dd, *J* = 17.7, 11.2 Hz, 1H), 5.92 (s, 1H), 5.87 (dd, *J* = 17.7, 1.3 Hz, 1H), 5.45 (dd, *J* = 11.2, 1.2 Hz, 1H), 3.91 (s, 3H) ppm.
Analytical data in agreement with literature.[6]

(3-Fluoro-2-vinylphenyl)boronic acid (S10)

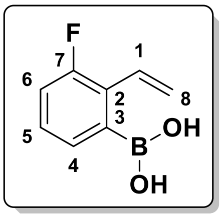

Prepared according to General Procedure **C**, 1-bromo-3-fluoro-2-vinylbenzene (1.75 mg, 8.7 mmol, 1 equiv.) was converted to **S10**, yielding a white solid (988 mg, 6.0 mmol, 68%) after flash column chromatography (SiO_2, 0→20% EtOAc/*n*-hexanes).

R_f (30% EtOAc/*n*-Hexanes) = 0.31.
^{1}H NMR (400 MHz, $CDCl_3$) δ = 7.50 (dd, *J* = 7.4, 1.2 Hz, 1H, H4), 7.32 – 7.21 (m, 1H, H5), 7.11 (ddd, *J* = 10.6, 8.2, 1.3 Hz, 1H, H6), 6.97 (dd, *J* = 17.8, 11.4 Hz, 1H, H1), 5.72 (d, *J* = 17.8 Hz, 1H, H8), 5.62 (dd, *J* = 11.4, 1.4 Hz, 1H, H8) ppm.
^{13}C NMR (100 MHz, $CDCl_3$) δ = 160.4 (d, *J* = 248.5 Hz, C7), 131.5 (d, *J* = 2.3 Hz, C1), 130.1, (C2) 130.0 (d, *J* = 3.7 Hz, C4), 128.9 (d, *J* = 8.1 Hz, C5), 122.3 (d, *J* = 5.5 Hz, C8), 117.7 (d, *J* = 22.6 Hz, C6) ppm.
^{11}B NMR (128 MHz, $CDCl_3$) δ = 30.05 ppm.
^{19}F NMR (376 MHz, $CDCl_3$) δ = -116.01 (dd, *J* = 10.6, 5.0 Hz) ppm.
HRMS (ESI) calc. for $C_8H_7BFO_2^-$ $[M-H]^-$ 165.0529, found 165.0531.
IR (ATR): ῦ = 2972, 2901, 1599, 1571, 1411, 1332, 1296, 1274, 1208, 1161, 1051, 992, 937, 912, 869, 827, 767, 738, 701, 656 cm^{-1}.

(5-Chloro-2-vinylphenyl)boronic acid (S11)

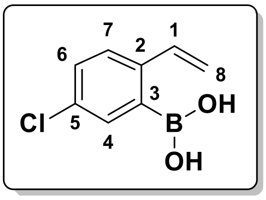

Prepared according to General Procedure **C**, 2-bromo-4-chloro-1-vinylbenzene (1.20 g, 5.5 mmol, 1 equiv.) was converted to **S11**, yielding a white solid (236 mg, 1.4 mmol, 26 %) after flash column chromatography (SiO_2, 0→20% EtOAc/*n*-hexanes).

R_f (20% EtOAc/*n*-Hexanes) = 0.19;

^{1}H NMR (400 MHz, $CDCl_3$) δ = 7.63 (d, *J* = 2.2 Hz, 1H, H4), 7.43 – 7.32 (m, 2H, H6+H7), 7.09 (dd, *J* = 17.4, 11.0 Hz, 1H, H1), 5.64 (dd, *J* = 17.4, 1.1 Hz, 1H, H8), 5.40 (dd, *J* = 11.0, 1.1 Hz, 1H, H8) ppm.
^{13}C NMR (100 MHz, $CDCl_3$) δ = 141.2 (C2), 136.9 (C1), 133.9 (C4), 133.5 (C5), 130.6 (C6), 127.8 (C7), 118.1 (C8) ppm.
^{11}B NMR (128 MHz, $CDCl_3$) δ = 29.73 ppm.
HRMS (ESI) calc. for $C_8H_7BClO_2^-$ $[M-H]^-$ 181.0233, found 181.0233.
IR (ATR): ῦ = 3359, 3256, 2922, 2502, 2436, 1583, 1546, 1476, 1400, 1332, 1255, 1196, 1107, 1052, 991, 913, 883, 860, 815, 764, 709, 672, 643 cm^{-1}.

2-Vinylaniline (S12)

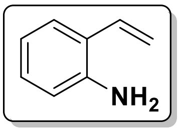

According to literature procedure by Hoveyda *et al.*,[8] to an oven-dried round-bottom-flask were added 2-(2-aminophenyl)ethan-1-ol (6.86 g, 50 mmol, 1 equiv.) and potassium hydroxide (2.81 g, 50 mmol, 1 equiv.) before the flask was connected to a short-path distillation apparatus. The mixture was heated to 180 °C under vacuum and distillation over 4 h yielded **S12** as a clear oil (4.09 g, 34 mmol, 68%).

^{1}H NMR (400 MHz, $CDCl_3$) δ = 7.29 (dd, *J* = 7.7, 1.6 Hz, 1H), 7.09 (td, *J* = 7.7, 1.6 Hz, 1H), 6.83 – 6.73 (m, 2H), 6.69 (dd, *J* = 8.0, 1.2 Hz, 1H), 5.63 (dd, *J* = 17.5, 1.5 Hz, 1H), 5.32 (dd, *J* = 11.1, 1.5 Hz, 1H), 3.83 (s, 2H) ppm.

4-Fluoro-2-vinylaniline (S13)

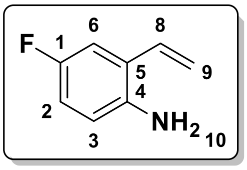

Prepared according to General Procedure **B**, **S1** (1.25 g, 9 mmol, 1 equiv.) was converted to **S13**, yielding a yellow oil (750 mg, 5.47 mmol, 61%) after flash column chromatography (0→20% EtOAc/*n*-hexanes).

R_f (20% EtOAc/*n*-Hexanes) = 0.16
^{1}H NMR (400 MHz, $CDCl_3$) δ = 7.01 (dd, *J* = 9.6, 3.0 Hz, 1H, H6), 6.87 – 6.78 (m, *J* = 8.4, 3.0 Hz, 1H, H2), 6.72 (ddd, *J* = 17.3, 11.0, 1.3 Hz, 1H, H8), 6.62 (dd, *J* = 8.7, 4.9 Hz, 1H, H3), 5.63 (dd, *J* = 17.3, 1.2 Hz, 1H, H9), 5.36 (dd, *J* = 11.0, 1.2 Hz, 1H, H9), 3.62 (s, 2H, H10) ppm.
^{13}C NMR (100 MHz, $CDCl_3$) δ = 156.7 (d, *J* = 236.0 Hz, C1), 139.8 (C4), 131.9 (d, *J* = 2.1 Hz, C8), 125.4 (d, *J* = 7.3 Hz, C5), 117.3 (d, *J* = 7.7 Hz, C3), 116.8 (C9), 115.4 (d, *J* = 22.6 Hz, C2), 113.2 (d, *J* = 22.6 Hz, C6) ppm.
^{19}F NMR (376 MHz, $CDCl_3$) δ = -126.28 (qd, *J* = 8.5, 4.7 Hz) ppm.
HRMS (ESI) calc. for $C_8H_9FN^+$ $[M+H]^+$ 138.0714, found 138.0676.
IR (ATR): ῦ = 3216, 2985, 1724, 1664, 1618, 1494, 1436, 1394, 1375, 1301, 1250, 1153, 1038, 993, 936, 915, 870, 811, 712, 640 cm^{-1}.

4-Chloro-2-vinylaniline (S14)

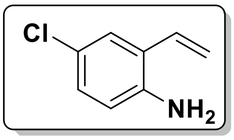

Prepared according to General Procedure **B**, **S2** (1.57 g, 10.1 mmol, 1 equiv.) was converted to **S14**, yielding a yellow oil (1.34 g, 8.7 mmol, 86%) after flash column chromatography (0→20% EtOAc/*n*-hexanes).

^{1}H NMR (400 MHz, $CDCl_3$) δ = 7.24 (d, *J* = 2.4 Hz, 1H), 7.03 (dd, *J* = 8.5, 2.4 Hz, 1H), 6.76 – 6.66 (m, 1H), 6.61 (d, *J* = 8.5 Hz, 1H), 5.63 (dd, *J* = 17.4, 1.3 Hz, 1H), 5.36 (dd, *J* = 11.0, 1.3 Hz, 1H), 3.73 (s, 2H) ppm.
Analytical data in agreement with literature.[9]

2*H*-Benzo[*e*][1,2]oxaborinin-2-ol (1)

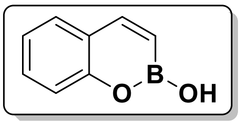

Prepared according to General Procedure **D**, **S3** (2.87 mL, 25 mmol, 1 equiv.) was converted to **1** by refluxing for 1 h, yielding a white solid (3.17 g, 87%) after flash column chromatography (C18, MeCN/water 20%→100%, 0.1% formic acid).

^{1}H NMR (400 MHz, $CDCl_3$) δ = 7.78 (d, *J* = 11.8 Hz, 1H), 7.49 – 7.31 (m, 2H), 7.27 – 7.19 (m, 1H), 7.15 (td, *J* = 7.4, 1.2 Hz, 1H), 6.22 (d, *J* = 11.8 Hz, 1H), 4.71 (s, 1H) ppm.
Analytical data in agreement with literature.[10]

8-Methoxy-2*H*-benzo[*e*][1,2]oxaborinin-2-ol (S15)

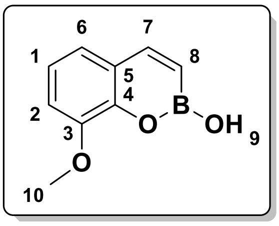

Prepared according to General Procedure **D**, **S4** (1.2 g, 8.0 mmol, 1 equiv.) was converted to **S15** by refluxing for 1 h, yielding a white solid (795 mg, 56%) after flash column chromatography (C18, MeCN/water 20%→100%, 0.1% formic acid).

R_f (10% EtOAc/*n*-hexane) = 0.08.
^{1}H NMR (400 MHz, $CDCl_3$) δ = 7.76 (d, *J* = 11.8 Hz, 1H, H7), 7.09 (dd, *J* = 7.8, 7.8 Hz, 1H, H1), 7.02 (dd, *J* = 7.8, 1.7 Hz, 1H, H6), 6.97 (dd, *J* = 7.8, 1.7 Hz, 1H, H2), 6.27 (d, *J* = 11.8 Hz, 1H, H8), 3.96 (s, 3H, H10) ppm.
^{13}C NMR (100 MHz, $CDCl_3$) δ = 149.4 (C7), 148.8 (C4), 141.9 (C3), 125.3 (C5), 122.1 (C1), 120.9 (C6), 111.5 (C2), 56.3 (C10) ppm.
^{11}B NMR (128 MHz, $CDCl_3$) δ = 27.58 ppm.
HRMS (ESI) calc. for $C_9H_9BO_3Na^+$ $[M+Na]^+$ 199.0537, found 199,0506.
IR (ATR): ṽ = 3504, 1606, 1558, 1477, 1461, 1421, 1370, 1347, 1331, 1271, 1230, 1222, 1199, 1172, 1140, 1074, 1016, 958, 867, 811, 736, 692, 670, 656, 627 cm^{-1}.

6-Methyl-2H-benzo[*e*][1,2]oxaborinin-2-ol (S16)

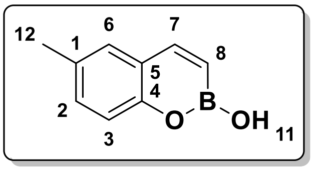

Prepared according to General Procedure **D**, **S5** (376 mg, 2.8 mmol, 1 equiv.) was converted to **S16** by refluxing overnight, yielding a colourless solid (300 mg, 67%) after flash column chromatography (SiO_2, 20% EtOAc/*n*-hexane).

R_f (20 % Acetone/*n*-hexane) = 0.18
^{1}H NMR (400 MHz, $CDCl_3$) δ = 7.75 (d, *J* = 12.1 Hz, 1H, H7), 7.26 – 7.06 (m, 3H, H2+H3+H6), 6.21 (d, *J* = 11.8 Hz, 1H, H8), 4.35 (s, 1H, H11), 2.40 (s, 3H, H12) ppm.
^{13}C NMR (100 MHz, $CDCl_3$) δ 150.5 (C4), 149.6 (C7), 131.7 (C1), 130.4 (C2), 128.9 (C6), 124.3 (C5), 118.2 (C3), 20.8 (C12) ppm.
^{11}B NMR (128 MHz, $CDCl_3$) δ = 27.76 ppm.
HRMS (ESI) calc. for $C_9H_8BO_2^-$ $[M-H]^-$ 159.0623, found 159.0638.
IR (ATR): ṽ = 3023, 2976, 2919, 1599, 1555, 1483, 1437, 1385, 1322, 1273, 1256, 1238, 1213, 1169, 1137, 1103, 1042, 946, 936, 904, 883, 857, 841, 818, 795, 735, 662, 650, 620 cm^{-1}.

6-(Thiophen-3-yl)-2*H*-benzo[*e*][1,2]oxaborinin-2-ol (S17)

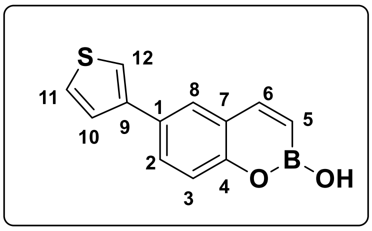

To a 5 mL microwave vial, **S19** (180 mg, 0.8 mmol, 1 equiv.), thiophen-3-ylboronic acid (512 mg, 4 mmol, 5 equiv.), Pd(dppf)Cl_2▪DCM (32 mg, 5 mol%) and K_3PO_4 (510 mg, 3 equiv.). The vial was sealed and purged with nitrogen, before the sequential addition via syringe of degassed THF/H_2O (4 mL, 0.2 M, 8:1). The reaction mixture was stirred at 50 °C for 24 h. After completion, the reaction was allowed to cool down to ambient temperature and 4 M aqueous HCl (16 mL) was added. The reaction mixture was stirred for 5 minutes and then extracted with DCM (3 × 20 mL). The combined organic phases were dried over Na_2SO_4 and concentrated under reduced pressure. The crude residue was purified by flash column chromatography (SiO_2, 0%→25% Acetone/n-hexane), yielding **S17** as an off-white solid (113 mg, 62%).

$\mathbf{R}_f$ (20% Acetone/*n*-hexane) = 0.10
^{1}**H NMR** (400 MHz, $CDCl_3$) δ = 7.81 (d, *J* = 11.8 Hz, 1H, H6), 7.64 – 7.58 (m, 2H, H2+H12), 7.42 (dt, *J* = 3.4, 1.7 Hz, 1H, H8), 7.41 – 7.37 (m, 2H, H10+H11), 7.29 – 7.26 (m, 1H, H3), 6.26 (d, *J* = 11.8 Hz, 1H, H5) ppm.
^{13}**C NMR** (100 MHz, $CDCl_3$) δ = 151.7 (C4), 149.6 (C6), 141.7 (C9), 130.54(C1), 127.8 (C2), 126.5 (C12), 126.5 (C10/11), 126.4 (C10/11), 124.8 (C7), 120.0 (C8), 119.0 (C3) ppm.
^{11}**B NMR** (128 MHz, $CDCl_3$) δ = 27.73 ppm.
HRMS (ESI) calc. for $C_{12}H_8BO_2S^-$ [M-H]$^-$ 227.0344, found 227.0361.
IR (ATR): ṽ = 3097, 1596, 1559, 1528, 1472, 1417, 1398, 1367, 1283, 1259, 1209, 1186, 1141, 1111, 1088, 1006, 936, 896, 881, 859, 840, 821, 801, 778, 762, 745, 670 cm^{-1}.

5-Fluoro-2*H*-benzo[*e*][1,2]oxaborinin-2-ol (S18)

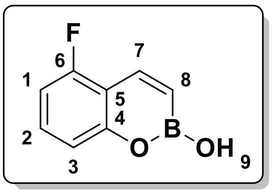

Prepared according to General Procedure **D**, **S6** (276 mg, 2.0 mmol, 1 equiv.) was converted to **S18** by refluxing for 1 h, yielding a yellow solid (292 mg, 89%) after flash column chromatography (C18, MeCN/water 20%→100%, 0.1% formic acid).

$\mathbf{R}_f$ (20% EtOAc/*n*-hexane) = 0.29
^{1}**H NMR** (400 MHz, $CDCl_3$) δ = 8.04 (d, *J* = 12.1 Hz, 1H, H7), 7.38 – 7.27 (m, 1H, H2), 7.04 (d, *J* = 8.4 Hz, 1H, H3), 6.87 (dd, *J* = 9.0, 9.0 Hz, 1H, H1), 6.27 (dd, *J* = 12.1, 2.1 Hz, 1H, H8), 4.78 (s, 1H, H9) ppm.
^{13}**C NMR** (100 MHz, $CDCl_3$) δ = 159.4 (d, *J* = 250.8 Hz, C6), 153.3 (d, *J* = 6.1 Hz, C4), 141.6 (d, *J* = 5.2 Hz, C7), 129.4 (d, *J* = 10.3 Hz, C2), 114.4 (d, *J* = 3.6 Hz, C3), 108.5 (d, *J* = 20.8 Hz, C1) ppm.
^{11}**B NMR** (128 MHz, $CDCl_3$) δ = 27.53 ppm.
^{19}**F NMR** (376 MHz, $CDCl_3$) δ = -121.09 (dd, *J* = 9.6, 6.5 Hz) ppm.
HRMS (ESI) calc. for $C_8H_5BFO_2$ [M-H]$^-$ 163.0372, found 163.0355.
IR (ATR): ṽ = 3233, 1627, 1599, 1552, 1461, 1417, 1338, 1316, 1282, 1265, 1236, 1222, 1159, 1121, 1064, 1008, 869, 833, 813, 767, 680, 665, 635 cm^{-1}.

6-Bromo-2*H*-benzo[*e*][1,2]oxaborinin-2-ol (S19)

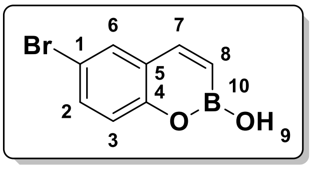

Prepared according to General Procedure **D**, **S7** (2.00 g, 10.0 mmol, 1 equiv.) was converted to **S19** by refluxing overnight, yielding a white solid (1.25 g, 55%) after flash column chromatography (C18, MeCN/water 20%→100%, 0.1% formic acid).

$\mathbf{R}_f$ (20% EtOAc/*n*-hexane) = 0.21;

^{1}H NMR (400 MHz, $CDCl_3$) δ = 7.68 (d, *J* = 11.9 Hz, 1H, H7), 7.54 (d, *J* = 2.4 Hz, 1H, H6), 7.45 (dd, *J* = 8.7, 2.4 Hz, 1H, H2), 7.13 (d, *J* = 8.7 Hz, 1H, H3), 6.27 (d, *J* = 11.9 Hz, 1H, H8), 4.56 (s, 1H, H9) ppm.
^{13}C NMR (100 MHz, $CDCl_3$) δ 151.4 (C4), 148.3 (C7), 132.3 (C2), 131.1 (C6), 126.2 (C5), 120.4 (C3), 114.8 (C1) ppm.
^{11}B NMR (128 MHz, $CDCl_3$) δ = 27.56 ppm.
HRMS (ESI) calc. for $C_8H_5BBrO_2^-$ $[M-H]^-$ 222.9571, found 222.9577.
IR (ATR): ṽ = 3290, 1754, 1739, 1598, 1549, 1473, 1441, 1377, 1355, 1329, 1308, 1266, 1248, 1210, 1186, 1127, 1108, 1067, 996, 894, 876, 818, 790, 732, 708, 640 cm^{-1}.

6-Fluoro-2*H*-benzo[*e*][1,2]oxaborinin-2-ol (S20)

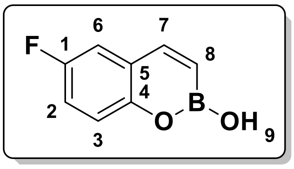

Prepared according to General Procedure **D**, **S8** (1.60 g, 11.6 mmol, 1 equiv.) was converted to **S20** by refluxing for 1 h, yielding a white solid (758 mg, 40%) after flash column chromatography (C18, MeCN/water 20%→100%, 0.1% formic acid).

R*f* (20% EtOAc/*n*-hexane) = 0.24;
^{1}H NMR (400 MHz, $CDCl_3$) δ = 7.70 (d, *J* = 11.9 Hz, 1H, H7), 7.22 – 7.17 (m, 1H, H6), 7.11 – 7.03 (m, 2H, H3, H2), 6.28 (dd, *J* = 11.9, 1.0 Hz, 1H, H8) ppm.
^{13}C NMR (100 MHz, $CDCl_3$) δ = 157.8 (d, *J* = 240.1 Hz, C1), 148.6 (d, *J* = 2.5 Hz, C7), 148.5 (d, *J* = 2.0 Hz, C4), 125.1 (d, *J* = 8.6 Hz, C5), 119.6 (d, *J* = 8.4 Hz, C3), 116.5 (d, *J* = 24.0 Hz, C2), 113.9 (d, *J* = 23.1 Hz, C3) ppm.
^{11}B NMR (128 MHz, $CDCl_3$) δ 27.56 ppm.
^{19}F NMR (376 MHz, $CDCl_3$) δ = -121.38 (td, *J* = 8.3, 4.6 Hz) ppm.
HRMS (ESI) calc. for $C_8H_5BFO_2$ $[M-H]^-$ 163.0372, found 163.0404.
IR (ATR): ṽ = 3298, 1611, 1552, 1484, 1464, 1393, 1337, 1268, 1239, 1149, 1120, 1093, 953, 939, 906, 874, 857, 818, 805, 785, 729, 649, 625 cm^{-1}.

Methyl 2-hydroxy-2*H*-benzo[*e*][1,2]oxaborinine-7-carboxylate (S21)

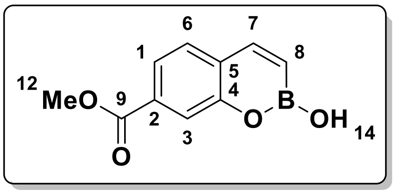

Prepared according to General Procedure **D**, **S9** (445 mg, 2.5 mmol, 1 equiv.) was converted to **S21** by refluxing overnight, yielding a white solid (129 mg, 25%) after flash column chromatography (SiO_2, 20% EtOAc/*n*-hexane).

R*f* (20% Acetone/*n*-hexane) = 0.09
^{1}H NMR (400 MHz, $CDCl_3$) δ = 7.91 (d, *J* = 1.6 Hz, 1H, H3), 7.85 – 7.72 (m, 2H, H7+H1), 7.46 (d, *J* = 8.0 Hz, 1H, H6), 6.37 (d, *J* = 11.8 Hz, 1H, H8), 3.94 (s, 3H, H12) ppm.
^{13}C NMR (100 MHz, $CDCl_3$) δ = 166.8 (C9), 152.0 (C4), 148.6 (C7), 130.9 (C2), 128.8 (C6), 128.3 (C5), 123.4 (C1), 120.0 (C3), 52.5 (C12) ppm.
^{11}B NMR (128 MHz, $CDCl_3$) δ = 27.65 ppm.
HRMS (ESI) calc. for $C_{10}H_8BO_4^-$ $[M-H]^-$ 203.0521, found 203.0538.
IR (ATR): ṽ = 3408, 3351, 2955, 1720, 1703, 1601, 1545, 1502, 1441, 1403, 1341, 1327, 1311, 1265, 1239, 1215, 1150, 1130, 1108, 1097, 1087, 1057, 986, 846, 795, 755, 738, 685, 632, 613 cm^{-1}.

2-Phenyl-1,2-dihydrobenzo[*e*][1,2]azaborinine (S22)

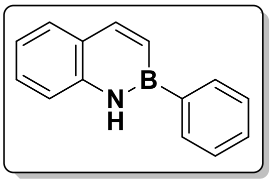

Prepared according to General Procedure **E**, **S12** (2.68 g, 22.5 mmol, 1.5 equiv.) and potassium phenyltrifluoroborate (2.76 g, 15 mmol, 1 equiv.) converted to **S22**, yielding a white solid (2.16 g, 10.5 mmol, 70%).

^{1}H NMR (400 MHz, $CDCl_3$) δ = 8.14 (d, *J* = 11.5 Hz, 1H), 7.97 – 7.87 (m, 2H), 7.67 (dt, *J* = 7.8, 1.0 Hz, 1H), 7.54 – 7.41 (m, 3H), 7.38 – 7.33 (m, 1H), 7.29 (d, *J* = 2.0 Hz, 1H), 7.20 (ddd, *J* = 8.1, 7.1, 1.2 Hz, 1H) ppm.
Analytical data in agreement with literature.[11]

6-Fluoro-2-phenyl-1,2-dihydrobenzo[*e*][1,2]azaborinine (S23)

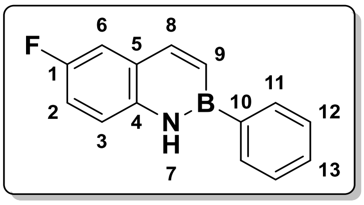

Prepared according to General Procedure **E**, **S13** (1.30 g, 9.5 mmol, 1.5 equiv.) and potassium phenyltrifluoroborate (1.17 g, 6.3 mmol, 1 equiv.) were converted to **S23**, yielding a white solid (736 mg, 3.3 mmol, 52%).

R_f (10% EtOAc/*n*-hexane) = 0.50.
^{1}H NMR (400 MHz, $CDCl_3$) δ = 8.13 (s, 1H, H7), 8.07 (d, *J* = 11.6 Hz, 1H, H8), 7.91 (dd, *J* = 7.2, 2.3 Hz, 2H, H12), 7.53 – 7.45 (m, 3H, H11+H13), 7.37 – 7.27 (m, 3H, H9+H2+H3), 7.20 (td, *J* = 8.5, 2.8 Hz, 1H, H2) ppm.
^{13}C NMR (100 MHz, $CDCl_3$) δ = 157.4 (d, *J* = 239.2 Hz, H1), 144.9 (d, *J* = 3.2 Hz, H8), 136.7 (C4), 132.8 (C12), 129.9 (C13), 128.4 (C11), 126.3 (d, *J* = 8.3 Hz, C5), 119.4 (d, *J* = 8.3 Hz, C3), 116.6 (d, *J* = 24.3 Hz, C2), 114.0 (d, *J* = 21.5 Hz, C6) ppm.
^{11}B NMR (128 MHz, $CDCl_3$) δ = 33.81 ppm.
^{19}F NMR (376 MHz, $CDCl_3$) δ = -122.53 (td, *J* = 8.3, 4.2 Hz) ppm.
HRMS (ESI) calc. for $C_{14}H_{11}BFNNa^{+}$ $[M+Na]^{+}$ 246.0861, 246.0797.
IR (ATR): ṽ = 3381, 1594, 1562, 1493, 1444, 1423, 1279, 1248, 1209, 1156, 1136, 1108, 1068, 1031, 999, 976, 876, 860, 850, 825, 805, 764, 735, 701, 637 cm^{-1}.

6-Chloro-2-phenyl-1,2-dihydrobenzo[*e*][1,2]azaborinine (S24)

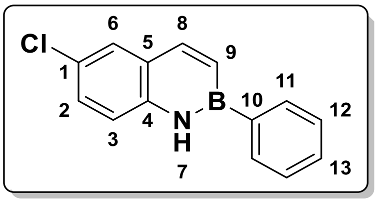

Prepared according to General Procedure **E**, **S14** (1.34 g, 8.7 mmol, 1.5 equiv.) and potassium phenyltrifluoroborate (1.07 g, 5.8 mmol, 1 equiv.) converted to **S24**, yielding a white solid (996 mg, 4.2 mmol, 72%).

R_f (10% EtOAc/*n*-hexane) = 0.49;
^{1}H NMR (400 MHz, $CDCl_3$) δ = 8.12 (s, 1H, H7), 8.04 (d, *J* = 11.7 Hz, 1H, H8), 7.93 – 7.88 (m, 2H), 7.64 (d, *J* = 2.3 Hz, 1H, H6), 7.52 – 7.44 (m, 3H, H11+H13), 7.40 (dd, *J* = 8.6, 2.4 Hz, 1H, H2), 7.33 (dd, *J* = 11.7, 1.9 Hz, 1H, H9), 7.28 (d, *J* = 8.6 Hz, 1H, H3) ppm.
^{13}C NMR (100 MHz, $CDCl_3$) δ = 144.6 (C8), 138.7 (C4), 132.8 (C12), 130.0 (C13), 128.6 (C6+C2), 128.4 (C11), 126.7 (C5), 126.2 (C1), 119.6 (C3) ppm.
^{11}B NMR (128 MHz, $CDCl_3$) δ = 34.11 ppm.
HRMS (ESI) calc. for $C_{14}H_{10}BClN^{-}$ $[M-H]^{-}$ 238.0600, found 238.0603.
IR (ATR): ṽ = 3379, 1611, 1594, 1558, 1479, 1441, 1417, 1364, 1337, 1278, 1210, 1192, 1128, 1085, 999, 975, 949, 910, 889, 827, 810, 765, 742, 699, 633 cm^{-1}.

2-Ethyl-1,2-dihydrobenzo[*e*][1,2]azaborinine (S25)

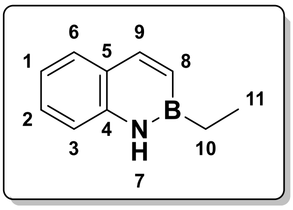

Prepared according to General Procedure **E**, **S12** (791 mg, 6.6 mmol, 1.5 equiv.) and potassium ethyltrifluoroborate (600 mg, 4.4 mmol, 1 equiv.) were converted to **S25**, yielding an off-white solid (577 mg, 3.68 mmol, 83%).

R*f* (10% EtOAc/*n*-hexane) = 0.48;

^{1}H NMR (400 MHz, $CDCl_3$) δ = 7.94 (d, *J* = 11.5 Hz, 1H, H9), 7.65 (s, 1H, H7), 7.59 (d, *J* = 7.8 Hz, 1H, H6), 7.38 (ddd, *J* = 7.8, 7.2, 1.5 Hz, 1H, H1), 7.22 (d, *J* = 8.1 Hz, 1H, H3), 7.13 (ddd, *J* = 8.1, 7.2, 1.1 Hz, 1H, H2), 6.82 (dd, *J* = 11.5, 1.9 Hz, 1H, H8), 1.26 (td, *J* = 6.9, 3.4 Hz, 2H, H10), 1.16 (td, *J* = 7.2, 1.3 Hz, 3H, H11) ppm.

^{13}C NMR (100 MHz, $CDCl_3$) δ = 144.3 (C9), 140.3 (C4), 129.4 (C6), 128.1 (C2), 125.5 (C5), 120.7 (C1), 117.9 (C3), 9.5 (C11) ppm.

^{11}B NMR (128 MHz, $CDCl_3$) δ = 38.46 ppm.

HRMS (ESI) calc. for $C_{10}H_{13}BN^+$ $[M+H]^+$ 158.1136, found 158.1140.

IR (ATR): ῦ = 3365, 2952, 2871, 1611, 1595, 1556, 1437, 1387, 1345, 1308, 1282, 1207, 1154, 1134, 1085, 1016, 942, 894, 813, 755 cm^{-1}.

1*H*-Benzo[*c*][1,2]oxaborinin-1-ol (S26)

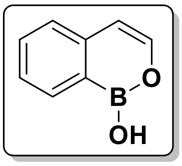

Prepared according to General Procedure **F**, (2-vinylphenyl)boronic acid (148 mg, 1 mmol, 1 equiv.) was converted to **S26**, yielding a light pink solid (63.1 mg, 43%) after flash column chromatography (SiO_2, 0→15% Acetone/*n*-hexane).

^{1}H NMR (600 MHz, $CDCl_3$) δ = 8.02 – 7.93 (m, 1H), 7.59 (td, *J* = 7.5, 1.3 Hz, 1H), 7.43 – 7.35 (m, 2H), 7.03 (d, *J* = 5.5 Hz, 1H), 6.29 (d, *J* = 5.5 Hz, 1H) ppm.

Analytical data in agreement with literature.[7]

5-Fluoro-1*H*-benzo[*c*][1,2]oxaborinin-1-ol (S27)

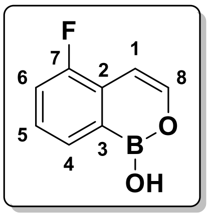

Prepared according to General Procedure **F**, **S10** (830 mg, 5.0 mmol, 1 equiv.) was converted to **S27** yielding, after flash column chromatography (SiO_2, 0→5% Acetone/*n*-hexane) an off-white solid (500 mg, 61%).

R*f* (10% Acetone/*n*-hexane) = 0.12;

^{1}H NMR (400 MHz, $CDCl_3$) δ = 7.77 (d, *J* = 6.6 Hz, 1H, H4), 7.44 – 7.26 (m, 2H, H5+H6), 7.07 (d, *J* = 5.5 Hz, 1H, H8), 6.56 (d, *J* = 5.5 Hz, 1H, H1) ppm.

^{13}C NMR (100 MHz, $CDCl_3$) δ = 157.7 (d, *J* = 251.1 Hz, C7), 143.1 (d, *J* = 1.9 Hz, C8), 130.0 (d, *J* = 13.1 Hz, C2), 128.4 (d, *J* = 4.0 Hz, C4), 127.4 (d, *J* = 7.0 Hz, C5), 118.1 (d, *J* = 20.2 Hz, C6), 102.1 (d, *J* = 4.8 Hz, C1) ppm.

^{11}B NMR (128 MHz, $CDCl_3$) δ = 28.32 ppm.

^{19}F NMR (376 MHz, $CDCl_3$) δ = -122.68 (dd, *J* = 10.2, 5.0 Hz) ppm.

HRMS (ESI) calc. for $C_8H_5BFO_2^-$ $[M-H]^-$ 163.0372, found 163.0372.

IR (ATR): 3429, 2980, 2901, 1635, 1553, 1455, 1435, 1406, 1312, 1274, 1237, 1161, 1138, 1076, 1058, 993, 903, 862, 780, 745, 677, 646, 631 cm^{-1}.

7-Chloro-1*H*-benzo[*c*][1,2]oxaborinin-1-ol (S28)

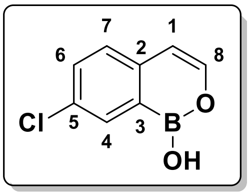

Prepared according to General Procedure **F**, **S11** (100 mg, 0.55 mmol, 1 equiv.) was converted to **S28**, yielding an off-white solid (43 mg, 44%) after flash-column chromatography (SiO_2, 0→15% Acetone/*n*-hexane).

***R**$_f$* (15% Acetone/*n*-hexane) = 0.08;
^{1}H NMR (400 MHz, $CDCl_3$) δ = 7.96 (d, *J* = 2.3 Hz, 1H, H4), 7.53 (dd, *J* = 8.3, 2.3 Hz, 1H, H6), 7.30 (d, *J* = 8.3 Hz, 1H, H7), 7.02 (d, *J* = 5.5 Hz, 1H, H8), 6.26 (d, *J* = 5.5 Hz, 1H, H1) ppm.
^{13}C NMR (100 MHz, $CDCl_3$) δ = 142.8 (C8), 140.2 (C2), 132.7 (C6), 132.4 (C4), 132.2 (C5), 127.1 (C7), 109.2 (C1) ppm.
^{11}B NMR (128 MHz, $CDCl_3$) δ = 28.31 ppm.
HRMS (ESI) calc. for $C_8H_7BClO_2^+$ $[M+H]^+$ 181.0222, found 181.0215.
IR (ATR): ῦ = 3303, 3062, 1632, 1477, 1418, 1402, 1355, 1304, 1262, 1197, 1151, 1134, 1091, 999, 906, 889, 831, 807, 777, 742, 640, 626 cm^{-1}.

Ethyl 2-fluoro-6-hydroxybenzoate (S29)

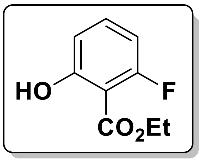

According to a literature procedure by Scott *et al.*,[12] to an open round-bottom-flask was given 2-fluoro-6-hydroxybenzoic acid (4.68 g, 30 mmol, 1 equiv.) and EtOH (300 mL, 0.1 M). To the mixture was added H_2SO_4 (conc., 8 mL, 150 mmol, 5 equiv.) dropwise and then the mixture was heated to reflux and stirred overnight. Subsequently, the mixture was cooled to ambient temperature, quenched with water (50 mL) and diluted with EtOAc (200 mL). The organics were extracted with Et_2O (3 × 300 mL) and the combined organics were washed with 5% aq. $NaHCO_3$ (3 × 300 mL) and brine (300 mL). Then the organics were dried over Na_2SO_4 and concentrated under reduced pressure. Purification by flash column chromatography (SiO_2, *n*-pentane) yielded **S29** as a colourless oil (3.88 g, 70%).

^{1}H NMR (400 MHz, $CDCl_3$) δ = 11.36 (s, 1H), 7.36 (td, *J* = 8.3, 6.0 Hz, 1H), 6.78 (dt, *J* = 8.5, 1.1 Hz, 1H), 6.60 (ddd, *J* = 10.9, 8.3, 1.1 Hz, 1H), 4.45 (q, *J* = 7.1 Hz, 2H), 1.43 (t, *J* = 7.1 Hz, 3H).
Analytical data in agreement with literature.[13]

Ethyl 6-fluoro-2-hydroxy-3-iodobenzoate (S30)

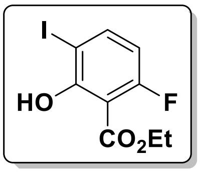

According to a literature procedure by Molloy *et al.*, to an over-dried round-bottom-flask was added **S29** (1.84 g, 10 mmol, 1 equiv.) and thallium (I) acetate (2.77 g, 10.5 mmol, 1.05 equiv.). After dissolving the solids in anhydrous DCM (100 mL, 0.1 M), a solution of iodine (2.67 g, 10.5 mol, 1.05 equiv.) in anhydrous DCM (40 mL) was added dropwise over 2 h via syringe pump. Subsequently, the reaction was stirred at ambient temperature overnight. Upon completion the mixture was filtered over a Celite plug and the crude was concentrated under reduced pressure. Purification by flash column chromatography (SiO_2, *n*-pentane) yielded **S30** as a white solid (1.41 g, 46%).

^{1}H NMR (600 MHz, CDCl3) δ = 12.27 (s, 1H), 7.85 (dd, J = 8.7, 5.7 Hz, 1H),6.49 (dd, J = 10.5, 8.7 Hz, 1H), 4.47 (q, J = 7.1 Hz, 2H), 1.43 (t, J = 7.1 Hz, 3H) ppm;
Analytical data in agreement with literature.[14]

Diethyl 2-allylmalonate (S31)

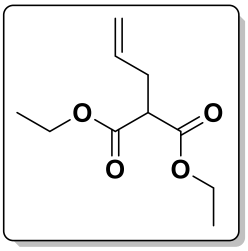

To an oven-dried flask was added NaH (4.20 g, 105 mmol, 1.05 equiv., 60% dispersion in mineral oil) before it was closed and purged with nitrogen (3 ×). Subsequently, anhydrous THF (300 mL) was added and the suspension cooled to 0 °C and stirred for 15 minutes before the dropwise addition of diethyl malonate (15.3 mL, 100 mmol, 1 equiv.). The reaction mixture stirred at 0 °C for 1 h before the allyl bromide (9.51 mL, 110 mmol, 1.1 equiv.) was added dropwise. The cooling bath was removed and the reaction mixture was stirred at ambient temperature for 16 h. Upon completion the reaction was quenched by slow addition of sat. aq. NH_4Cl (100 mL). The organics were extracted with EtOAc (3 × 100 mL). The collected organics were washed with brine (100 mL), dried over Na_2SO_4 and concentrated under reduced pressure. Purification by flash column chromatography (SiO_2, *n*-pentane) yielded **S31** as a clear oil (9.58 g, 76%).

^{1}H NMR (400 MHz, $CDCl_3$) δ = 5.78 (ddt, *J* = 17.0, 10.2, 6.8 Hz, 1H), 5.23 – 4.98 (m, 2H), 4.20 (qd, *J* = 7.1, 1.6 Hz, 4H), 3.42 (t, *J* = 7.6 Hz, 1H), 2.77 – 2.56 (m, 2H), 1.26 (t, *J* = 7.1 Hz, 6H) ppm.
Analytical data in agreement with literature.[15]

Diethyl 2-allyl-2-(prop-2-yn-1-yl)malonate (S32)

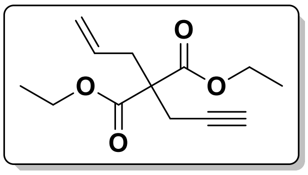

To an oven-dried flask was added NaH (1.89 g, 47.3 mmol, 1.05 equiv., 60% dispersion in mineral oil) before it was closed and purged with nitrogen (3 ×). Subsequently, anhydrous THF (300 mL) was added and the suspension cooled to 0 °C and stirred for 15 minutes before the dropwise addition of **S31** (9.0 g, 45 mmol, 1 equiv.). The reaction mixture stirred at 0 °C for 30 minutes before the propargyl bromide (4.2 mL, 49.5 mmol, 1.1 equiv., 80 wt% in toluene) was added dropwise. The cooling bath was removed and the reaction mixture was stirred at ambient temperature for 16 h. Upon completion the reaction was quenched by slow addition of sat. aq. NH_4Cl (100 mL). The organics were extracted with EtOAc (3 × 100 mL). The collected organics were washed with brine (1 × 100 mL), dried over Na_2SO_4 and concentrated under reduced pressure. Purification by flash column chromatography (SiO_2, *n*-pentane) yielded **S32** as a pale-yellow oil (10.5 g, quant.).

^{1}H NMR (400 MHz, $CDCl_3$) δ = 5.63 (ddt, *J* = 17.4, 10.1, 7.5 Hz, 1H), 5.21 – 5.02 (m, 2H), 4.21 (q, *J* = 7.1 Hz, 4H), 2.85 – 2.75 (m, 4H), 2.01 (t, *J* = 2.7 Hz, 1H), 1.26 (t, *J* = 7.1 Hz, 6H) ppm.
Analytical data in agreement with literature.[16]

***N*-Allyl-4-methylbenzenesulfonamide (S33)**

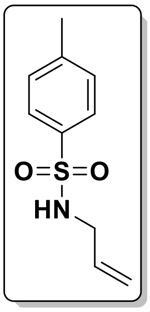

An oven-dried three-necked-flask was purged with nitrogen (3 ×) before the addition of a solution of allylamine (11.2 mL, 150 mmol, 1.5 equiv.) in anhydrous DCM (750 mL). Subsequently, the flask was cooled to 0 °C, then Et_3N was added slowly. Upon the completion of the addition, *para*-tosyl chloride (1.0 equiv.) was added portion wise under a flow of nitrogen. The flask was warmed to ambient temperature and stirred overnight. The reaction mixture was quenched by the addition of sat. aq. NH_4Cl (200 mL), the layers were separated and the organics extracted with DCM (3 × 200 mL). The combined organics were washed with brine (200 mL), dried over Na_2SO_4 and concentrated under reduced pressure to yield **S33** as large white crystals (21.34 g, quant). No further purification was required.

^{1}H NMR (400 MHz, $CDCl_3$) δ = 7.75 (d, *J* = 8.3 Hz, 2H), 7.31 (d, *J* = 7.9 Hz, 1H), 5.72 (ddt, *J* = 17.1, 10.2, 5.8 Hz, 1H), 5.24 – 5.04 (m, 2H), 4.45 (s, 1H), 3.59 (td, *J* = 5.2, 3.0 Hz, 2H), 2.43 (s, 3H).
Analytical data in agreement with literature.[17]

N-Allyl-4-methyl-N-(prop-2-yn-1-yl)benzenesulfonamide (S34)

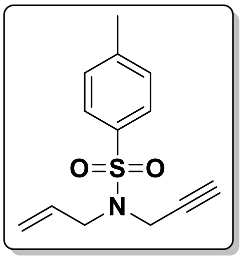

To an oven-dried flask was added sodium hydride (4.2 g, 105 mmol, 1.05 equiv., 60% dispersion in mineral oil) before it was closed and purged with nitrogen (3 ×). Subsequently, anhydrous THF (300 mL) was added and the suspension cooled to 0 °C and stirred for 15 minutes before the dropwise addition of **S33** (21.1 g, 100 mmol, 1 equiv.). The reaction mixture stirred at 0 °C for 30 minutes before the propargyl bromide (13.0 mL, 110 mmol, 1.1 equiv., 80 wt% in toluene) was added dropwise. The cooling bath was removed and the reaction mixture was stirred at ambient temperature for 16 h. Upon completion the reaction was quenched by slow addition of sat. aq. NH_4Cl (100 mL). The organics were extracted with EtOAc (3 × 100 mL). The collected organics were washed with brine (1 × 100 mL), dried over Na_2SO_4 and concentrated under reduced pressure. Purification by flash column chromatography (SiO_2, 20% EtOAc/*n*-hexane) yielded **S34** as a yellow solid (26.80 g, 98%).

^{1}H NMR (400 MHz, $CDCl_3$) δ =7.74 (d, *J* = 8.3 Hz, 2H), 7.30 (d, *J* = 8.0 Hz, 3H), 5.73 (ddt, *J* = 16.7, 10.0, 6.5 Hz, 1H), 5.39 – 5.17 (m, 2H), 4.10 (d, *J* = 2.5 Hz, 2H), 3.83 (dt, *J* = 6.5, 1.3 Hz, 2H), 2.43 (s, 3H), 2.00 (t, *J* = 2.5 Hz, 1H) ppm.
Analytical data in agreement with literature.[18]

(*E*)-(4,4-Bis(ethoxycarbonyl)hepta-1,6-diene-1,2-diyl)diboronic acid, pinacol ester (S35)

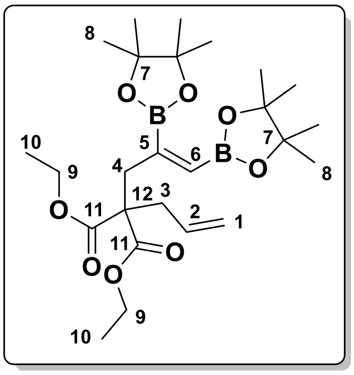

According to a an adapted literature procedure by Miyaura *et al.*[19] to an oven-dried round-bottom flask were added **S32** (3.80 g, 15 mmol, 1 equiv.), bis(pinacolato)diboron (3.81 g, 15 mmol, 1 equiv.) and $Pt(PPh_3)_4$ (560 mg, 0.45 mmol, 3 mol%). The flask was sealed and purged with nitrogen (3 ×) before degassed, anhydrous toluene (20 mL) was added via syringe. The reaction mixture was stirred for 48 h at 100 °C. Upon completion, the mixture was concentrated under reduced pressure and purification via flash column chromatography (SiO_2, 0%→10% EtOAc/*n*-hexane) yielded **S35** as a light-yellow oil (5.18 g, 68%).

R_f (10% Acetone/*n*-hexane) = 0.19.
^{1}H NMR (400 MHz, $CDCl_3$) δ = 5.96 (s, 1H, H6), 5.82 (ddt, *J* = 17.3, 10.0, 7.3 Hz, 1H, H2), 5.14 – 4.94 (m, 2H, H1), 4.26 – 4.04 (m, 4H, H9), 2.86 (s, 2H, H4), 2.63 (d, *J* = 7.3 Hz, 2H, H3), 1.28 (s, 12H, H8), 1.26 (s, 12H, H8), 1.22 (t, *J* = 7.1 Hz, 6H, H10) ppm.
^{13}C NMR (100 MHz, $CDCl_3$) δ = 171.0 (C11), 133.5 (C2), 118.5 (C1), 84.0 (C7), 83.5 (C7), 61.1 (C9), 58.7 (C12), 41.3 (C4), 37.0 (C3), 25.1 (C8), 25.0 (C8), 14.2 (C10) ppm.
^{11}B NMR (128 MHz, $CDCl_3$) δ = 29.92 ppm.
HRMS (ESI) calc. for $C_{25}H_{43}B_2O_8^+$ $[M+H]^+$ 493.3139, found 493.3167.
IR (ATR): ṽ = 2979, 2936, 2347, 1731, 1611, 1405, 1391, 1380, 1371, 1311, 1282, 1249, 1210, 1186, 1166, 1138, 1124, 1113, 1051, 1005, 969, 919, 863, 850, 833, 732, 702, 668 cm^{-1}.

(*E*)-(3-((*N*-allyl-4-methylphenyl)sulfonamido)prop-1-ene-1,2-diyl)diboronic acid, pinacol ester (S36)

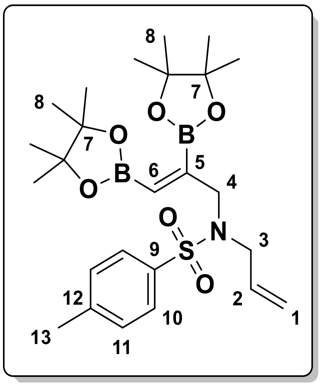

According to a an adapted literature procedure by Miyaura *et al.*,[19] to an oven dried round-bottom flask were added **S34** (4.99 g, 20 mmol, 1 equiv.), bis(pinacolato)diboron (5.08 g, 20 mmol, 1 equiv.) and $Pt(PPh_3)_4$ (747 mg, 0.6 mmol, 3 mol%). The flask was sealed and purged with nitrogen (3 ×) before degasses, anhydrous toluene (20 mL) was added via syringe. The reaction mixture was stirred for 34 h at 100 °C. Upon completion, the mixture was concentrated under reduced pressure and purification via flash column chromatography (SiO_2, 0%→20% EtOAc/*n*-hexane) yielded **S36** as an off-white solid (6.74 g, 67%).

***R**f* (10% Acetone/*n*-hexane) = 0.17.

^{1}H NMR (400 MHz, $CDCl_3$) δ = 7.69 (d, *J* = 8.4 Hz, 2H, H10), 7.26 (d, *J* = 8.4 Hz, 2H, H11), 5.96 (s, 1H, H6), 5.42 (ddt, *J* = 16.7, 10.1, 6.6 Hz, 1H, H2), 5.09 – 5.00 (m, 2H, H1), 3.94 (d, *J* = 1.7 Hz, 2H, H4), 3.82 (d, *J* = 6.6 Hz, 2H, H3), 2.41 (s, 3H, H13), 1.31 (s, 12H, H8), 1.27 (s, 12H, H8) ppm.

^{13}C NMR (100 MHz, $CDCl_3$) δ = 143.1 (C12), 137.9 (C9), 132.0 (C2), 129.7 (C11), 127.5 (C11), 119.5 (C1), 84.2 (C7), 83.7 (C7), 53.5 (C4), 49.7 (C3), 25.2 (C8), 25.0 (C8), 21.6 (C13) ppm.

^{11}B NMR (128 MHz, $CDCl_3$) δ = 30.65 ppm.

HRMS (ESI) calc. for $C_{25}H_{39}B_2NO_6SNa^+$ $[M+Na]^+$ 526.2576, found 526.2593.

IR (ATR): ῦ = 2980, 2930, 2326, 1625, 1405, 1393, 1380, 1372, 1319, 1272, 1216, 1157, 1138, 1124, 1111, 1091, 1067, 1058, 969, 916, 893, 859, 850, 817, 803, 770, 722, 660, 616 cm^{-1}.

Diethyl 2-allyl-2-((2-hydroxy-2*H*-benzo[e][1,2]oxaborinin-3-yl)methyl)malonate (S37)

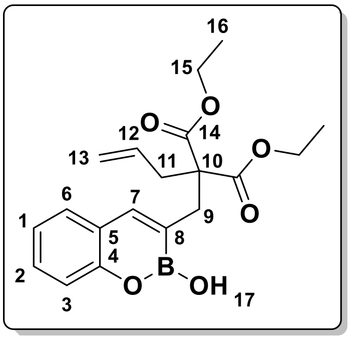

Prepared according to General Procedure **G**, 2-iodophenol (660 mg, 3 mmol, 1 equiv.) and **S35** (2.22 g, 4.5 mmol, 1.5 equiv.), were converted, yielding **S37** as a yellow oil (597 mg, 1.67 mmol, 56%), after flash column chromatography (SiO_2, 0%→10% Acetone/*n*-hexane).

***R**f* (10% Acetone/*n*-hexane) = 0.15.

^{1}H NMR (400 MHz, $CDCl_3$ with a drop of D_2O) δ = 7.43 (s, 1H, H7), 7.38 – 7.31 (m, 2H, H6+H2), 7.26 (d, *J* = 8.5 Hz, 1H, H1), 7.15 – 7.02 (m, 1H, H3), 5.83 – 5.68 (m, 1H, H12), 5.23 – 5.14 (m, 2H, H13), 4.13 (m, 4H, H15), 3.00 (s, 2H, H9), 2.78 (d, *J* = 7.4 Hz, 2H, H11), 1.17 (t, *J* = 7.1 Hz, 6H, H16) ppm.

^{13}C NMR (100 MHz, $CDCl_3$ with a drop of D_2O) δ = 172.0 (C14), 152.3 (C4), 148.2 (C7), 132.2 (C12), 129.3 (C2), 128.3 (C6), 124.0 (C5), 122.2 (C1), 119.7 (C13), 118.4 (C3), 61.9 (C15), 59.5 (C10), 40.3 (C11), 38.2 (C9), 14.2 (C16) ppm.

^{11}B NMR (128 MHz, $CDCl_3$ with a drop of D_2O) δ = 27.91 ppm.

HRMS (ESI) calc. for $C_{19}H_{23}BO_6Na^+$ $[M+Na]^+$ 381.1480, found 381.1473.

IR (ATR): ῦ = 2980, 1724, 1608, 1487, 1413, 1345, 1256, 1206, 1193, 1140, 1095, 1042, 919, 859, 757, 696 cm^{-1}.

***N*-Allyl-*N*-((6-fluoro-2-hydroxy-2*H*-benzo[*e*][1,2]oxaborinin-3-yl)methyl)-4-methylbenzenesulfonamide (S38)**

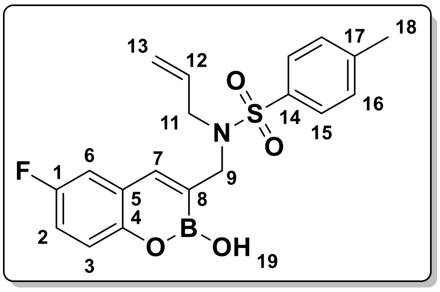

Prepared according to General Procedure **G**, 4-fluoro-2-iodophenol (714 mg, 3 mmol, 1 equiv.) and **S36** (2.26 g, 4.5 mmol, 1.5 equiv.) were converted, yielding **S38** as an off-white solid (464 mg, 1.2 mmol, 40%), after flash column chromatography (SiO_2, 0%→20% Acetone/*n*-hexane).

R R_f (20% EtOAc/*n*-hexane) = 0.12;
^{1}H NMR (400 MHz, $CDCl_3$ with a drop of D_2O) δ = 7.76 (d, *J* = 8.3 Hz, 2H, H15), 7.40 – 7.30 (m, 2H, H16+H7), 7.26 (m, 1H, H3), 7.09 (td, *J* = 8.5, 3.0 Hz, 1H, H2), 7.02 (dd, *J* = 8.5, 3.0 Hz, 1H, H6), 6.98 (s, 1H, H19), 5.52 (ddt, *J* = 16.8, 10.2, 6.6 Hz, 1H, H12), 5.19 – 4.96 (m, 2H, H13), 4.08 (s, 2H, H9), 3.88 (d, *J* = 6.6 Hz, 2H, H11), 2.46 (s, 3H, H18) ppm.
^{13}C NMR (100 MHz, $CDCl_3$ with a drop of D_2O) δ = 157.74 (d, *J* = 240.3 Hz, C1), 148.86 (d, *J* = 1.6 Hz, C4), 145.92 (d, *J* = 2.6 Hz, C7), 144.13 (C17), 136.56 (C14), 132.19 (C12), 130.14 (C16), 127.37 (C15), 123.97 (d, *J* = 8.3 Hz, C5), 119.93 (C13), 119.71 (d, *J* = 8.3 Hz, C3), 116.95 (d, *J* = 24.0 Hz, C2), 113.68 (d, *J* = 23.2 Hz, C6), 50.17 (C11+C9), 21.72 (C18) ppm.
^{11}B NMR (128 MHz, $CDCl_3$ with a drop of D_2O) δ = 27.89 ppm.
^{19}F NMR (376 MHz, $CDCl_3$ with a drop of D_2O) δ = -121.44 (dt, *J* = 8.3, 4.1 Hz) ppm.
HRMS (ESI) calc. for $C_{19}H_{19}BFNO_4SNa^+$ $[M+Na]^+$ 410.1004, found 410.1005.
IR (ATR): ῦ = 3379, 2324, 1619, 1568, 1487, 1451, 1394, 1308, 1268, 1249, 1141, 1098, 1087, 1055, 993, 959, 943, 899, 871, 814, 782, 768, 741, 721, 663 cm^{-1}.

Diethyl 2-allyl-2-((8-(ethoxycarbonyl)-7-fluoro-2-hydroxy-2*H*-benzo[*e*][1,2]oxaborinin-3-yl)methyl)malonate (S39)

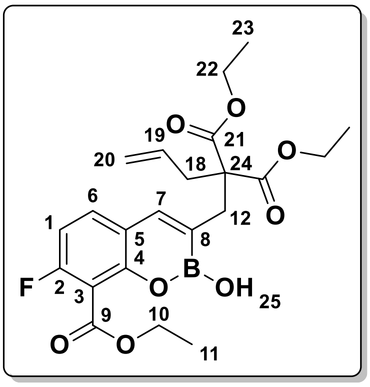

Prepared according to General Procedure **G**, **S30** (310 mg, 1.0 mmol, 1.0 equiv.) and **S35** (632 mg, 1.3 mmol, 1.3 equiv.) were converted, yielding **S39** as a light-brown oil (256 mg, 0.57 mmol, 57%), after flash column chromatography (SiO_2, 0%→15% EtOAc/*n*-pentane).

R_f (10% EtOAc/*n*-hexane) = 0.10
^{1}H NMR (400 MHz, $CDCl_3$ with a drop of D_2O) δ = 7.39 – 7.31 (m, 2H, H7+H6), 6.88 (dd, *J* = 8.8, 8.8 Hz, 1H, H1), 5.85 – 5.64 (m, 1H, H19), 5.20 – 5.11 (m, 2H, H20), 4.45 (q, *J* = 7.1 Hz, 2H, H10), 4.26 – 3.93 (m, 4H, H22), 2.97 (s, 2H, H12), 2.73 (d, *J* = 7.8 Hz, 2H, H18), 1.41 (t, *J* = 7.1 Hz, 3H, H11), 1.17 (t, *J* = 7.1 Hz, 6H, H23) ppm.
^{13}C NMR (101 MHz, $CDCl_3$ with a drop of D_2O) δ = 171.8 (C21), 163.5 (C9), 159.9 (d, *J* = 253.6 Hz, C2), 150.4 (d, *J* = 7.5 Hz, C4), 146.7 (C7), 132.2 (C19), 130.8 (d, *J* = 10.5 Hz, C6), 120.9 (d, *J* = 3.0 Hz, C5), 119.7 (C20), 112.7 (d, *J* = 19.3 Hz, C3), 110.1 (d, *J* = 22.6 Hz, C1), 62.1 (C10), 61.9 (C22), 59.3 (C24), 39.8 (C18), 37.4 (C12), 14.3 (C11), 14.1 (C23) ppm.
^{11}B NMR (128 MHz, $CDCl_3$ with a drop of D_2O) δ = 28.48 ppm.
^{19}F NMR (376 MHz, $CDCl_3$ with a drop of D_2O) δ = -112.15 (dd, *J* = 9.1, 5.9 Hz) ppm.
HRMS (ESI) calc. for $C_{22}H_{26}BFO_8Na^+$ $[M+Na]^+$ 471.1597, found 471.1594.
IR (ATR): ῦ = 3210, 2982, 1729, 1664, 1618, 1602, 1444, 1426, 1400, 1375, 1296, 1248, 1184, 1133, 1095, 1035, 1019, 915, 860, 810, 729, 640 cm^{-1}.

Methyl 3-(((N-allyl-4-methylphenyl)sulfonamido)methyl)-2-hydroxy-2*H*-benzo[*e*][1,2]oxaborinine-7-carboxylate (S40)

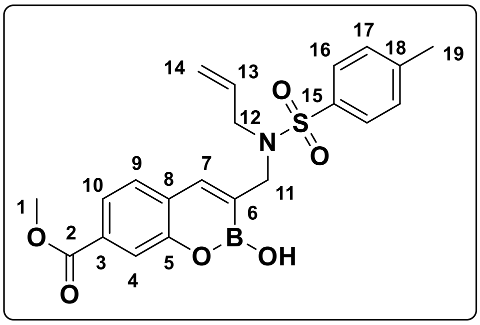

Prepared according to General Procedure **G**, methyl 3-hydroxy-4-iodobenzoate (292 mg, 1.05 mmol, 1 equiv.) and **S36** (630 mg, 1.2 mmol, 1.5 equiv.) were converted, yielding **S40** as an off-white solid (170 mg, 0.40 mmol, 38%), after flash column chromatography (SiO_2, 0%→30% Acetone/*n*-hexane).

R*f* (30% Acetone/*n*-hexane) = 0.13

^{1}H NMR (400 MHz, $CDCl_3$ with a drop of D_2O) δ = 7.94 (d, *J* = 1.6 Hz, 1H, H4), 7.79 (dd, *J* = 8.0, 1.7 Hz, 1H, H10), 7.78 – 7.74 (m, 2H, H16), 7.42 (s, 1H, H7), 7.40 (d, *J* = 8.1 Hz, 1H, H9), 7.36 – 7.32 (m, 2H, H17), 5.52 (ddt, *J* = 16.9, 10.4, 6.5 Hz, 1H, H14), 5.15 – 5.06 (m, 2H, H13), 4.10 (s, 1H, H11), 3.94 (s, 3H, H1), 3.89 (d, *J* = 6.6 Hz, 1H, H12), 2.45 (s, 3H, H19) ppm.

^{13}C NMR (100 MHz, $CDCl_3$ with a drop of D_2O) δ = 166.7 (C2), 152.3 (C5), 145.8 (C7), 144.1 (C18), 136.5 (C15), 132.2 (C13), 131.2 (C8), 130.1 (C17), 128.5 (C9), 127.4 (C16), 127.2 (C3), 123.3 (C10), 120.0 (C4/14), 120.0 (C4/14), 52.5 (C1), 50.3 (C12), 50.1 (C11), 21.7 (C19) ppm.

^{11}B NMR (128 MHz, $CDCl_3$ with a drop of D_2O) δ = 27.33 ppm.

HRMS (ESI) calc. for $C_{21}H_{22}BNO_6SNa^+$ $[M+Na]^+$ 450.1153, found 450.1151.

IR (ATR): ṽ = 3483, 1704, 1615, 1598, 1499, 1439, 1403, 1383, 1339, 1321, 1301, 1210, 1150, 1120, 1087, 1047, 1015, 999, 983, 966, 939, 925, 894, 837, 815, 803, 761, 741, 718, 699, 680, 658 cm^{-1}.

N-allyl-N-((2-hydroxy-6,8-dimethyl-2*H*-benzo[*e*][1,2]oxaborinin-3-yl)methyl)-4-methylbenzenesulfonamide (S41)

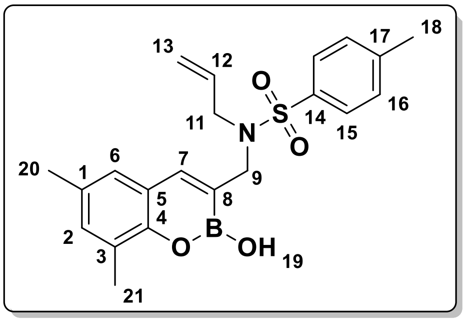

Prepared according to General Procedure **G**, 2-iodo-4,6-dimethylphenol (248 mg, 1 mmol, 1 equiv.) and **S36** (755 mg, 1.5 mmol, 1.5 equiv.) were converted, yielding **S41** as a white solid (299 mg, 0.75 mmol, 75%), after flash column chromatography (SiO_2, 0%→7.5% Acetone/*n*-hexane).

R*f* (30% Acetone/*n*-hexane) = 0.21.

^{1}H NMR (400 MHz, $CDCl_3$) δ = 7.77 (d, *J* = 8.3 Hz, 2H, H15), 7.41 – 7.30 (m, 3H, H16, H7), 7.08 (s, 1H, H2), 6.97 (s, 1H, H6), 6.85 (s, 1H, H19), 5.52 (ddt, *J* = 16.8, 10.3, 6.5 Hz, 1H, H12), 5.15 – 5.03 (m, 2H, H13), 4.07 (s, 2H, H9), 3.87 (d, *J* = 6.5 Hz, 2H, H11), 2.45 (s, 3H, H18), 2.41 (s, 3H, H21), 2.32 (s, 3H, H20) ppm.

^{13}C NMR (100 MHz, $CDCl_3$) δ = 149.1 (C4), 147.5 (C7), 144.0 (C17), 136.8 (C14), 132.3 (C12), 132.2 (C2), 131.0 (C5), 130.1 (C16), 127.4 (C15), 126.3 (C6), 122.7 (C1), 119.8 (C13), 50.2 (C9), 49.9 (C11), 21.7 (C18), 20.7 (C20), 16.0 (C21) ppm.

^{11}B NMR (128 MHz, $CDCl_3$) δ = 27.82 ppm.

HRMS (ESI) calc. for $C_{21}H_{24}BNO_4SNa^+$ $[M+Na]^+$ 420.1411, found 420.1402.

IR (ATR): ṽ = 3447, 1743, 1582, 1424, 1400, 1321, 1262, 1220, 1146, 1090, 1041, 1018, 991, 927, 899, 856, 814, 757, 691, 659, 606 cm^{-1}.

Ethyl 3-(((N-allyl-4-methylphenyl)sulfonamido)methyl)-7-fluoro-2-hydroxy-2*H*-benzo[*e*][1,2]oxaborinine-8-carboxylate (S42)

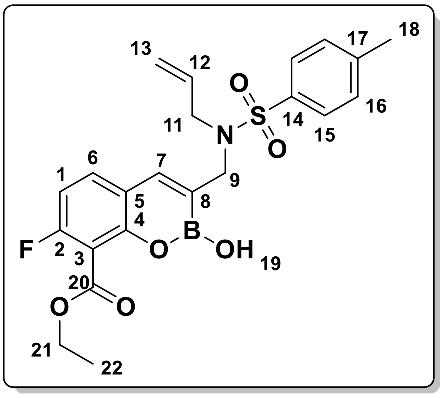

Prepared according to General Procedure **G**, **S30** (310 mg, 1 mmol, 1 equiv.) and **S36** (755 mg, 1.5 mmol, 1.5 equiv.) were converted, yielding **S42** as an off-white solid (347 mg, 0.75 mmol, 75%), after flash column chromatography (SiO_2, 0%→30% Acetone/*n*-hexane).

R*f* (30% Acetone/*n*-hexane) = 0.14
^{1}H NMR (400 MHz, $CDCl_3$) δ = 7.75 (d, *J* = 8.0 Hz, 2H, H15), 7.54 – 7.30 (m, 4H, H16+H6+H7), 6.91 (t, *J* = 8.7 Hz, 1H, H1), 5.51 (ddt, *J* = 16.8, 10.2, 6.5 Hz, 1H, H12), 5.27 – 4.92 (m, 2H, H13), 4.48 (q, *J* = 7.1 Hz, 2H, H21), 4.06 (s, 2H, H9), 3.86 (d, *J* = 6.5 Hz, 2H, H11), 2.44 (s, 3H, H18), 1.42 (t, *J* = 7.1 Hz, 3H, H22) ppm.
^{13}C NMR (100 MHz, $CDCl_3$) δ = 163.2 (C20), 160.1 (d, *J* = 253.8 Hz, C2), 150.7 (d, *J* = 7.7 Hz, C4), 145.3 (C7), 144.0 (C17), 136.6 (C14), 132.2 (C12), 130.9 (d, *J* = 10.6 Hz, H6), 130.1 (C16), 127.4 (C15), 120.5 (d, *J* = 2.8 Hz, C5), 119.9 (C13), 113.2 (d, *J* = 19.6 Hz, C3), 110.2 (d, *J* = 22.8 Hz, C1), 62.2 (C21), 50.3 (C11), 49.7 (C9), 21.7 (C18), 14.4 (C22) ppm.
^{11}B NMR (128 MHz, $CDCl_3$) δ = 27.84 ppm.
HRMS (ESI) calc. for $C_{22}H_{23}BFNO_6SNa^+$ $[M+Na]^+$ 482.1215, found 482.1217.
IR (ATR): ῦ = 3421, 1733, 1624, 1585, 1484, 1414, 1319, 1304, 1252, 1209, 1146, 1090, 989, 939, 927, 902, 815, 765, 738, 715, 701, 659, 626 cm^{-1}.

Trimethyl(phenylethynyl)silane (S43)

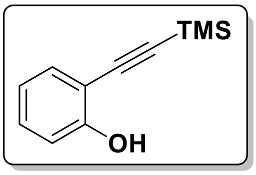

According to a literature procedure by Ingleson *et al.*,[20] to an oven-dried round-bottom flask was added 2-iodophenol (2.2 g, 10 mmol, 1.0 equiv.), copper (I) iodide (171 mg, 0.9 mmol, 0.09 equiv.) and $Pd(PPh_3)_2Cl_2$ (211 mg, 0.3 mmol, 0.03 equiv.) before it was sealed and purge with nitrogen (3 ×). Subsequently, triethylamine (12 mL), THF (25 mL) and trimethylsilylacetylene (1.8 mL, 13 mmol, 1.3 equiv.) were added and the mixture was stirred for 20 h. Upon completion, the reaction was quenched in sat. aq. NH_4Cl (30 mL) and the organics were extracted with EtOAc (3 × 30 mL). The combine organic layers were dried over Na_2SO_4 and concentrated under reduced pressure. Purification flash column chromatography (SiO_2, 0%→10% DCM/*n*-hexane) yielded **S43** as a colourless oil (1.86 g, 9.8 mmol, 98%).

^{1}H NMR (400 MHz, $CDCl_3$) δ = 7.34 (dd, *J* = 7.7, 1.7 Hz, 1H), 7.25 – 7.21 (m, 1H), 6.94 (dd, *J* = 8.3, 1.1 Hz, 1H), 6.85 (ddd, *J* = 7.5, 7.5, 1.1 Hz, 1H), 5.83 (d, *J* = 1.3 Hz, 1H), 0.28 (s, 9H) ppm.
Analytical data in agreement with literature.[21]

2-Ethynylphenol (S44)

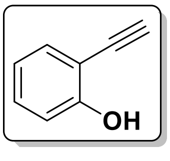

According to a adapted literature procedure by Ingleson *et al.*,[20] to an oven-dried round-bottom flask was added **S43** (1.8 g, 9.8 mmol, 1 equiv.) before it was sealed and purged with nitrogen (3 ×). Subsequently, anhydrous THF (40 mL) was added and the mixture was cooled to 0 °C before tetrabutylammonium fluoride (10 mL, 10 mmol, 1 equiv., 1 M in THF) was added dropwise. The mixture was stirred at ambient temperature for 2 h. Upon completion, the reaction was quenched with sat. aq. NH_4Cl (50 mL) and the organics were extracted with Et_2O (3 × 50 mL). The combined organic phase was washed with sat. aq.

NH_4Cl and water, then dried over Na_2SO_4 and concentrated under reduced pressure. Purification flash column chromatography (SiO_2, 0%→10% EtOAc/*n*-hexane) yielded **S44** as a colourless oil (1.15 g, 9.8 mmol, 99%) that turned purple over time.

^{1}H NMR (400 MHz, $CDCl_3$) δ = 7.38 (dd, *J* = 7.7, 1.7 Hz, 1H), 7.31 – 7.26 (m, 1H), 6.96 (dd, *J* = 8.3, 1.1 Hz, 1H), 6.88 (ddd, *J* = 7.5, 7.5, 1.1 Hz, 1H), 5.30 (s, 1H), 3.47 (s, 1H) ppm.
Analytical data in agreement with literature.[21]

Di(pent-4-en-1-yl)zinc (S45)

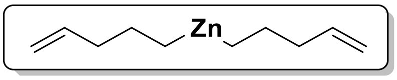

According to a adapted literature procedure by Baran *et al.*,[22] to an oven-dried round-bottom-flask were added magnesium turnings (729 mg, 30 mmol, 2 equiv.) and iodine (38 mg, 0.15 mmol, 0.01 equiv.). To another oven-dried round-bottom-flask was added 5-bromopent-1-ene (1.78 mL, 15 mmol, 1 equiv.) and THF (20 mL) via syringe. To the magnesium and iodine was added 2 mL of the solution. In case the brown colour did not disappear, the flask was heated with a heat gun until this was achieved. The remainder of the solution in THF was added dropwise while heating with a heat gun. Upon completion, the mixture was stirred for 1 h before it was titrated against iodine (0.5 M Grignard solution was established). In a separated Schlenck-flask zinc chloride (1.36 g, 10 mmol) was dried under vacuum over night while stirring at 150 °C. To the cooled flask was added anhydrous THF (10 mL) and the solution was stirred for 5 min. To another oven-dried round-bottom-flask was added 3 mL of the zinc chloride-solution and 12 mL of the Grignard-solution was added dropwise and the mixture was stirred for at least 10 min. The conversion was assumed to be quantitative and used for the next step.

4-(Pent-4-en-1-yl)-2*H*-benzo[*e*][1,2]oxaborinin-2-ol (S46)

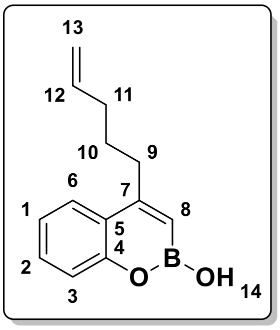

According to adapted literature procedure by Ingelson *et al.*,[20] to an oven-dried round-bottom-flask was added **S44** (118 mg, 1 mmol, equiv.) and anhydrous DCM (3 mL). To this solution was added boron trichloride (1.0 M in hexanes, 2.0 mL, 2 mmol. 2 equiv.) dropwise over 5 minutes and the upon completion the reaction was stirred for 1 h. Subsequently, all volatiles were removed under reduced pressure. To the resulting solid was added solution of $Pd_2(dba)_3$ (114 mg, 0.12 mmol, 0.12 equiv.) and Ruphos (56 mg, 0.12 mmol, 0.12 equiv.) in anhydrous THF (4.5 mL) and the freshly prepared **S45**. The reaction mixture was stirred at ambient temperature for 1 h and subsequently at 60 °C for 16 h. Upon completion the reaction mixture was allowed to cool to ambient temperature and the mixture was slowly added to sat. aq. $NaHCO_3$ (15 mL) at 0 °C. The mixture was stirred over night at ambient temperature before it was acidified with HCl (1 M). The product was extracted with EtOAc (3 × 20 mL), dried over Na_2SO_4 and concentrated under reduced pressure. Purification by flash column chromatography (SiO_2, 0%→5% Acetone/*n*-hexane and then 50→100% DCM/*n*-pentane) yielded **S46** as an off-white oil (48.6 mg, 0.23 mmol, 23%).

R_f (100% DCM) = 0.37.
^{1}H NMR (400 MHz, $CDCl_3$) δ = 7.62 (dd, *J* = 8.0, 1.6 Hz, 1H, H6), 7.38 (ddd, *J* = 8.2, 7.1, 1.6 Hz, 1H, H2), 7.26 (*J* = 8.2, 1.4 Hz, 1H, H3), 7.16 (ddd, *J* = 8.0, 7.1, 1.4 Hz, 1H, H1), 6.02 (d, *J* = 1.1 Hz, 1H, H8), 5.86 (ddt, *J* = 16.9, 10.2, 6.7 Hz, 1H, H12), 5.14 – 4.84 (m, 2H, H13), 2.90 – 2.72 (m, 2H, H9), 2.29 – 2.13 (m, 2H, H11), 1.85 – 1.72 (m, 2H, H10) ppm.
^{13}C NMR (101 MHz, $CDCl_3$) δ = 160.0 (C7), 152.8 (C4), 138.4 (C12), 129.3 (C2), 125.0 (C6), 124.4 (C5), 122.1 (C2), 119.2 (C3), 115.29 (C13), 34.1 (C12), 33.7 (C11), 27.8 (C10) ppm.

^{11}B NMR (128 MHz, $CDCl_3$) δ = 27.74 ppm.
HRMS (ESI) calc. for $C_{13}H_{16}BO_2^+$ $[M+H]^+$ 215.1238, found 215.1226.
IR (ATR): ῦ = 3273, 2930, 2860, 1641, 1601, 1553, 1484, 1457, 1403, 1337, 1305, 1276, 1240, 1177, 1124, 1074, 1039, 998, 909, 879, 837, 804, 751, 699, 643 cm^{-1}.

Ser-Thr-Phe-Lys (S47)

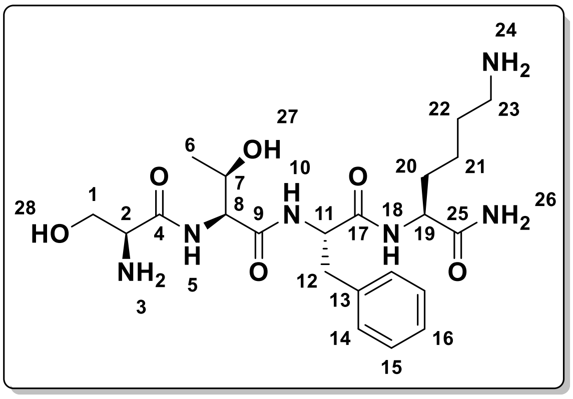

S47 was prepared at 0.1 mmol scale using the novel solid phase synthesizer with 263 mg of pegylated rink amide resin Rink of 0.38 mmol/g loading. Fmoc-deprotection was performed with piperidine (20% in DMF, 2 mL delivered) at 75 °C for 2 min. Coupling reactions were performed with a 5-fold excess of Fmoc-AA-OH (0.25 M in DMF, 2 mL delivered), DIC (0.5 M in DMF) and Oxyma Pure (0.5 M in DMF, 1 mL delivered) at 75 °C for 5 min. Cleavage was performed in a fritted syringe twice for 2 h using a cleavage cocktail of TFA/H_2O/TIPS (90:5:5) and a bench top shaker. Following cleavage, the resin was washed with cleavage cocktail and the solution reduced under nitrogen stream to an oily texture. The oil was precipitated in ice cold Et_2O, centrifuged for 4 minutes at 12 100 rpm. The supernatant was discarded, **S39** was then re-dissolved in Milli-Q water and acetonitrile and lyophilised overnight.

^{1}H NMR (400 MHz, DMSO-*d*6) δ = 8.43 (d, *J* = 8.5 Hz, 1H, H5), 8.10 (d, *J* = 8.1 Hz, 1H, H18), 8.03 (d, *J* = 7.9 Hz, 1H, H10), 7.29 – 7.14 (m, 6H, H_{Ar}, H26), 7.08 (d, *J* = 2.1 Hz, 1H, H26), 5.54 (d, *J* = 5.1 Hz, 1H, H28), 5.09 (d, *J* = 4.7 Hz, 1H, H27), 4.53 (td, *J* = 8.4, 4.7 Hz, 1H, H11), 4.27 (dd, *J* = 8.4, 4.3 Hz, 1H, H8), 4.14 (td, *J* = 8.5, 5.0 Hz, 1H, H19), 4.04 – 3.93 (m 2H, H7+H2), 3.76 – 3.68 (m, 1H, H1), 3.63 (d, *J* = 6.2 Hz, 1H, H1), 3.06 (dd, *J* = 14.0, 4.7 Hz, 1H, H12), 2.86 – 2.80 (m, 1H, H12), 2.79 – 2.69 (m, 2H, H23), 1.76 – 1.59 (m, 1H, H20), 1.57 – 1.44 (m, 3H, H22, H20), 1.28 (td, *J* = 15.5, 7.9 Hz, 2H, H21), 1.08 (s, 1H), 1.01 (d, *J* = 6.2 Hz, 3H, H6) ppm.

^{13}C NMR (101 MHz, DMSO-*d*6) δ = 173.8 (C25), 171.2 (C17) , 169.9 (C9), 167.6 (C4), 138.0 (C13), 129.7 (C14), 128.6 (C15), 126.7 (C16), 67.0 (C7), 61.0 (C1), 58.6 (C8), 54.5 (C2+C11), 52.7 (C19), 37.6 (C12), 31.8 (C20), 27.1 (C22), 22.7 (C21), 19.8 (C6) ppm.

HRMS (ESI) calc. for $C_{22}H_{37}N_6O_6^+$ $[M+H]^+$ 481.2769, found 481.2780.

Method for analytical HPLC:
*Syn*ergi Hydro RP18 column, Phenomenex, 250 × 4.6 mm, 4 µm, flow rate of 1 mL/min with H_2O (0.1% formic acid) and ACN as eluents, solvent regime: isocratic (5 min) 0% ACN, linear gradient to 100% ACN (45 min), isocratic 100% ACN (10 min)]. ELSD Detector: 60 °C, wavelength detection: 214 nm, 254 nm, 270 nm, 280 nm.

Experimental Setup for Photoreactions

Reaction Setup Photocatalyzed [2+2]-Cycloadditions of 2-12, 22-30, 37-42.

The photocatalytic reactions yielding boron heterocycles **2-12, 22-30, 37-42** were carried out using 450 nm LED lamps with a power of 1 W. The reaction vials were placed 1 cm above the WINGER® WEPRB1-S1 Power LED Star royal blue (450nm) LEDs which were placed on a stirring plate (Heidolph MR Hei-End, 800 rpm). To regulate the temperature, a fan was used for cooling. The internal temperature of the photoreactions was measured at 23 °C.

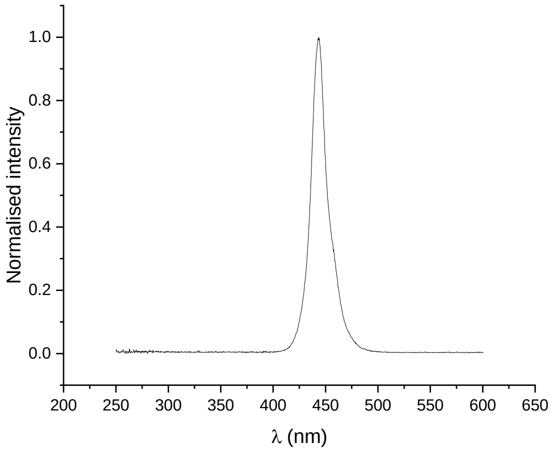

Figure S1: Emission spectrum of WINGER® WEPRB1-S1 Power LED Star royal blue (450nm) 1 W – 201m.

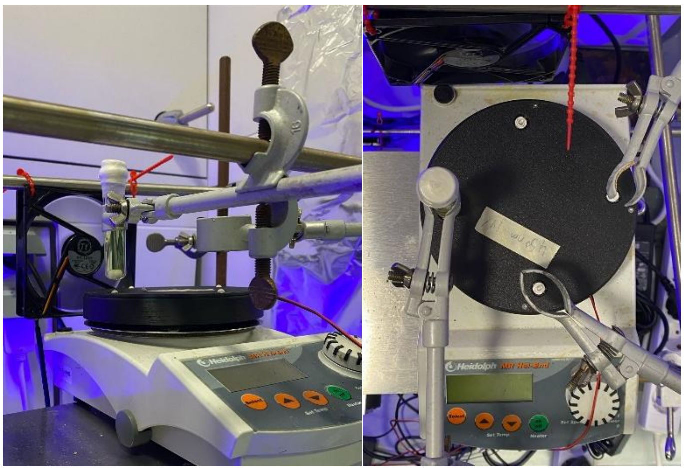

*Figure S2: Reaction set up for photocatalyzed [2+2]-cycloadditions of **2-12, 22-30, 37-42**.*

Reaction Setup Photocatalyzed [2+2]-Cycloadditions of 13-21.

The photocatalytic reactions yielding boron heterocycles **13-21** were carried out using Kessil PR160L-440 LED lamps with the producer's power settings. The lamp was placed 4.5 cm away from the reaction vial, that was placed in the middle of a stirring plate (Roth Rotilabo-Mini-Magnetrührer M3 Stirring speed, 550 rpm). To regulate temperature, a fan was used for cooling.

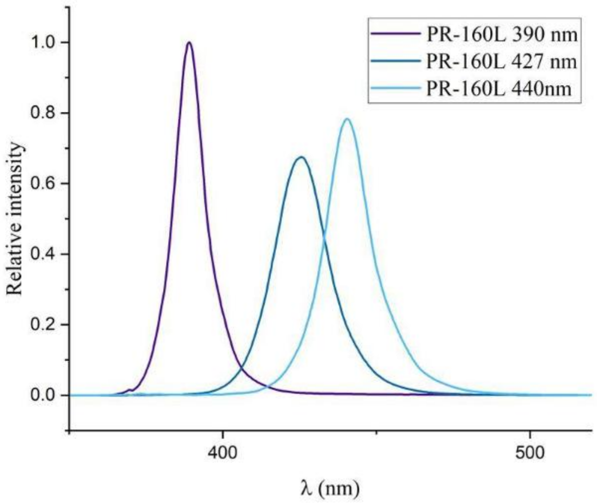

Figure S3: Emission spectra of Kessil PR160L-390, Kessil PR160L-427 and Kessil PR160L-440.

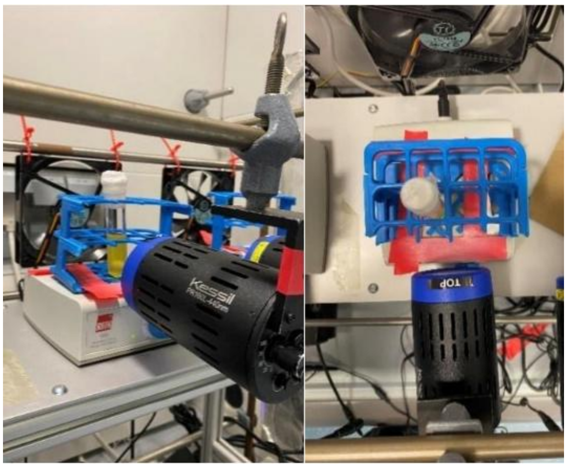

Figure S4: Reaction set-up for photocatalyzed [2+2]-cycloadditions of ***13-21****.*

Reaction Optimisation

Optimisation of [2+2]-Cycloadditon of Benzoxaborines.

Table S1: Catalyst Screening for [2+2]-Cycloadditon of Benzoxaborines.

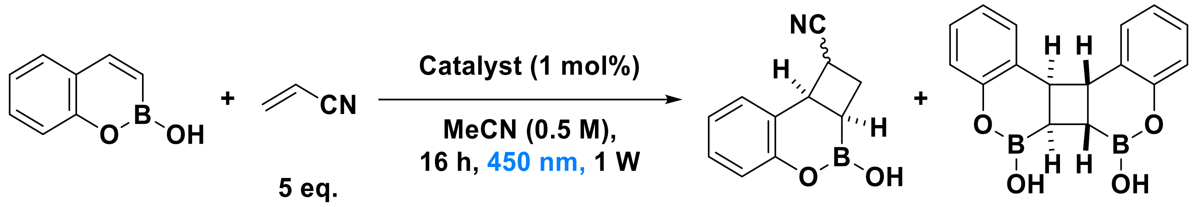

Cat	E_T (kJ/mol)	Yield[a] (%)	Dimer[a] (%)	SM[a] (%)
**Ir(dFppy$_3$)**	**251**	**84 (1.4:1)**	**15**	**0**
Ir(dF(CF$_3$)ppy$_2$)(dtbbpy)PF$_6$	251	52 (1:1.2)	40	0
Ir(pF-ppy$_3$)	245	77 (1:2:1)	21	0
Ir(ppy$_3$)	231	13 (1:1)	5	79
Ir(dtbbpy)(ppy$_3$)	205	<5	<5	91
TX	265	77 (1.2:1)	22	0

[a] Determined by ^{1}H NMR spectroscopy against a known internal standard (1,3,5-trimethoxybenzene).

Table S2: Solvent screening for [2+2]-Cycloadditon of Benzoxaborines.

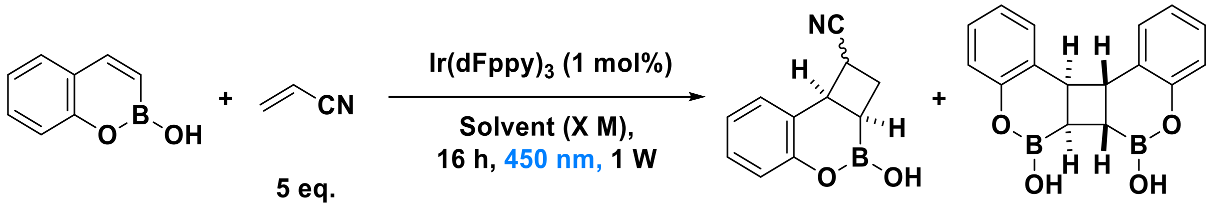

Solvent	Concentration (M)	Yield[a] (%)	Dimer[a] (%)	SM[a] (%)
MeCN	0.05	84	15	0
Toluene	0.05	85	16	0
**Toluene**	**0.025**	**87**	**12**	**0**
DMF	0.05	84	17	0
THF	0.05	75	23	0
MeOH	0.05	66	30	0

[a] Determined by ^{1}H NMR spectroscopy against a known internal standard (1,3,5-trimethoxybenzene).

Table S3: Effect of Acrylonitrile Equivalents and Concentration on [2+2]-Cycloadditon of Benzoxaborines.

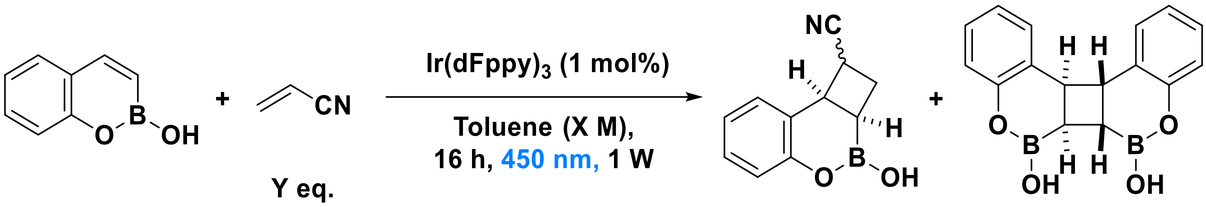

Concentration (M)	Y (equiv.)	Yield[a] (%)	Dimer[a] (%)	SM[a] (%)
0.05	10	87	13	0
0.05	25	93	7	0
0.025	10	87	7	0
0.025	25	94	3	0
**0.025**	**50**	**96**	**0**	**0**

[a] Determined by ^{1}H NMR spectroscopy against a known internal standard (1,3,5-trimethoxybenzene).

Table S4: Light source screening.

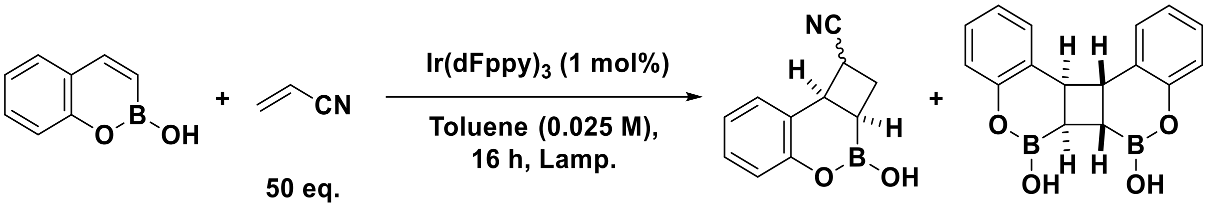

Light source	Yield[a] (%)	Dimer[a] (%)	SM[a] (%)
390 nm (40 W)	80	0	0
427 nm (40 W)	83	0	0
440 nm (40 W)	93	0	0
450 nm (1 W)	96	0	0

[a] Determined by ^{1}H NMR spectroscopy against a known internal standard (1,3,5-trimethoxybenzene).

Table S5: Control Reactions for [2+2]-Cycloadditions of Benzoxaborines.

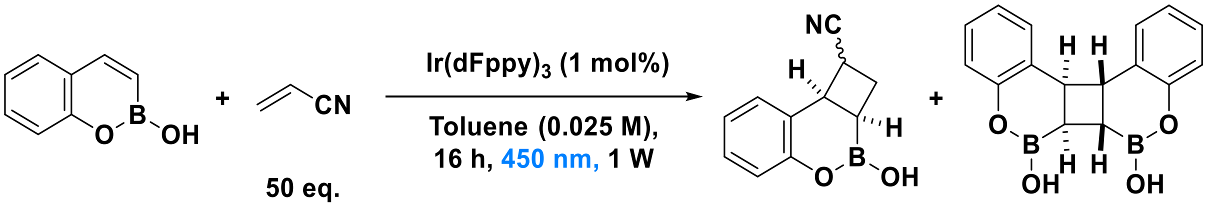

Control	Yield[a] (%)	Dimer[a] (%)	SM[a] (%)
No Light	0	0	98
Air	0	0	60
No catalyst	0	0	99

[a] Determined by ^{1}H NMR spectroscopy against a known internal standard (1,3,5-trimethoxybenzene).

Optimisation of [2+2]-Cycloadditon of Benzoxaborines.

Table S6: Translation of Model Conditions for [2+2]-Cycloadditions of Benzoxaborines to Benzazaborines.

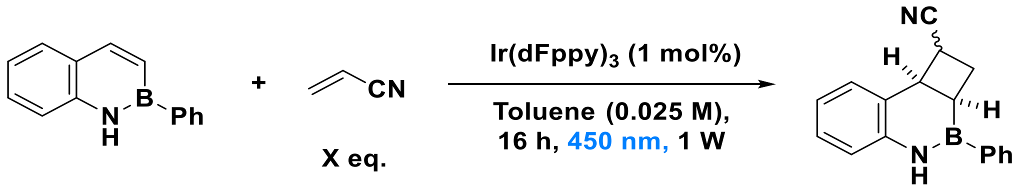

X	Yield[a] (%)	Dimer[a] (%)	SM[a] (%)
5	19	0	80
50	22	0	82

[a] Determined by ^{1}H NMR spectroscopy against a known internal standard (1,3,5-trimethoxybenzene).

Table S7: Catalyst Screening for [2+2]-Cycloadditon of Benzazaborines.

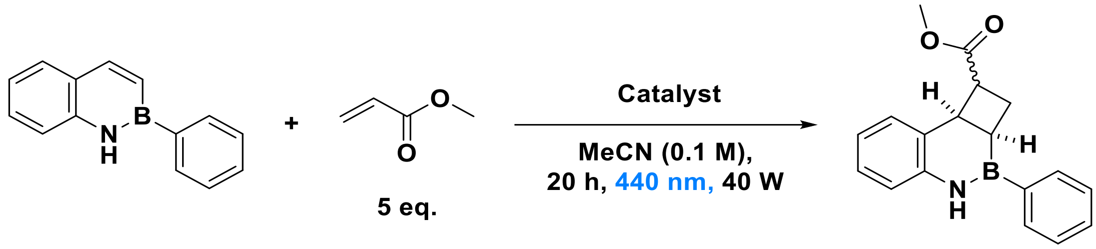

Cat	E_T (Kcal/mol)	Yield[a] (%)	SM[a] (%)
**Ir(dFppy$_3$)**	**60.1**	**66**	**12**
Ir(dF(CF$_3$)ppy$_2$)(dtbbpy)PF$_6$	60.1	20	72
Ir(pCF$_3$-ppy$_3$)	56.4	0	93
Ir(ppy$_3$)	55.2	0	90
TX	65.5	50	42

[a] Determined by ^{1}H NMR spectroscopy against a known internal standard (1,3,5-trimethoxybenzene).

Table S8: Solvent Screening for [2+2]-Cycloadditon of Benzazaborines.

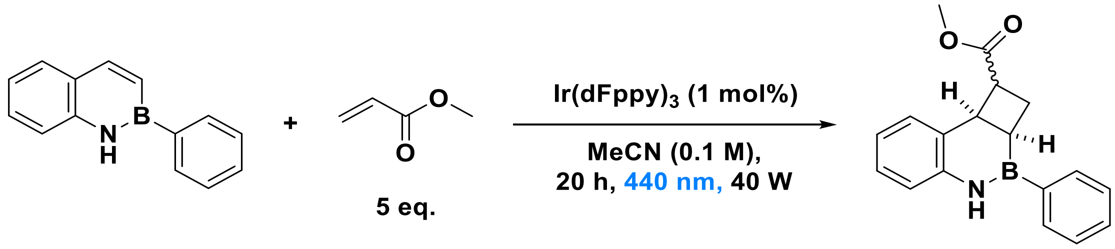

Solvent	Yield[a] (%)	SM[a] (%)
**THF**	**81**	**0**
**DCM**	**81**	**0**
MeCN	66	12
MeCN/H_2O (9:1)	23	15
DCE	66	0
Toluene	76	0
DMF	36	12

[a] Determined by ^{1}H NMR spectroscopy against a known internal standard (1,3,5-trimethoxybenzene).

Table S9: Effect of Concentration on [2+2]-Cycloadditon of Benzoxaborines.

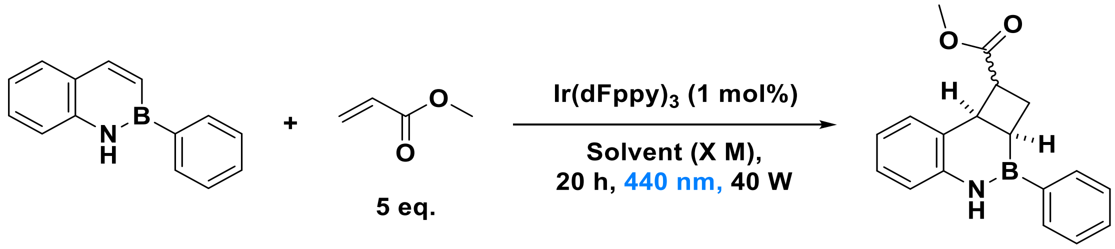

Solvent	Concentration (M)	Yield[a] (%)	SM[a] (%)
**THF**	**0.05**	**82**	**0**
THF	0.1	74	0
THF	0.2	75	0
THF	0.4	58	2
DCM	0.05	65	2
DCM	0.1	74	0
DCM	0.2	63	4

[a] Determined by ^{1}H NMR spectroscopy against a known internal standard (1,3,5-trimethoxybenzene).

Substrate Scope

General Procedure H: Photocatalyzed [2+2]-cycloadditions of benzoxaborines.

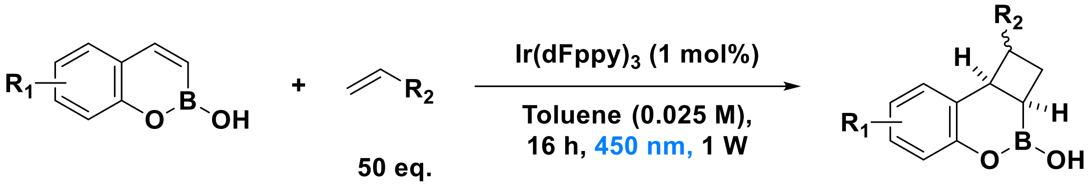

To a 20 mL microwave vial, the specified Benzoxaborine (0.2 mmol, 1 equiv.) and Ir(dFppy)$_3$ (1 mol%) was added. The vial was sealed with septum and purged with nitrogen (3 ×) before the sequential addition of degassed toluene (8 mL, 0.025 M) and alkene (50 equiv.) via syringe. The reaction mixture was stirred under light irradiation (450 nm, 1 LED, 1W) for 16 h. After completion internal standard (1,3,5-trimethoxybenzene), as a solution in MeCN, was added and the reaction mixture was transferred to a round-bottom-flask and the volatiles were removed under reduced pressure. The crude mixture was purified by column chromatography (specified conditions).

General Procedure I: Photocatalyzed [2+2]-cycloadditions of benzazaborines.

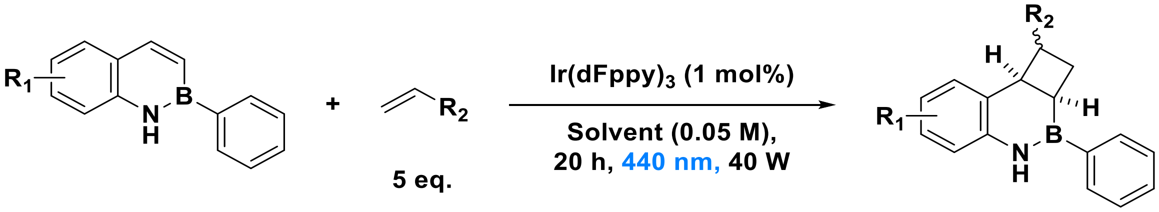

To a 10 mL microwave vial, the specified Benzoxaborine (0.2 mmol, 1 equiv.) and Ir(dFppy)3 (1 mol%) was added. The vial was sealed with septum and purged with nitrogen (3 ×) before the sequential addition of degassed THF (4 mL, 0.05 M) and alkene (5 equiv.) via syringe. The reaction mixture was stirred under light irradiation (440 nm, Kessil, 40W) for 20 h. After completion internal standard (1,3,5-trimethoxybenzene), as a solution in MeCN, was added and the reaction mixture was transferred to a round-bottom-flask and the volatiles were removed under reduced pressure. The crude mixture was purified by column chromatography (C-18, water/MeCN).

3-Hydroxy-1,2a,3,8b-tetrahydro-2*H*-benzo[*e*]cyclobuta[*c*][1,2]oxaborinine-1-carbonitrile (2)

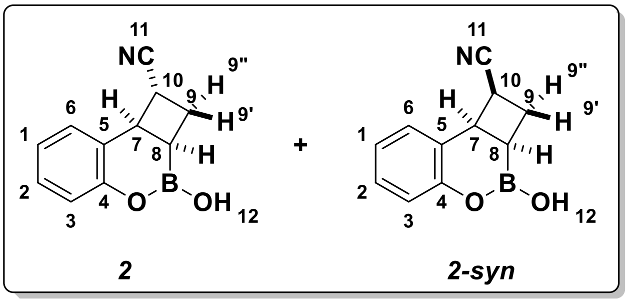

Prepared according to General Procedure **H**, **1** (29.2 mg, 0.2 mmol, 1 equiv.), acrylonitrile (658 µL, 10 mmol, 50 equiv.) and Ir(dFppy)$_3$ (1.5 mg, 1 mol%) were irradiated for 16 h with blue light (440 nm, 1 W). Upon completion, internal standard (1,3,5-trimethoxybenzene), as a solution in MeCN, was added and the mixture was concentrated under reduced pressure, the crude (95% NMR yield, *d.r.* = 1.4:1) was resolved in DCM and filtered. The filtrate was concentrated under reduced pressure and purification by flash column chromatography (C18, 10→100% MeCN/water) yielded **2** (18.3 mg, 0.092 mmol, 46%, *d.r.* = >20:1) and **2-*syn*** (10.7 mg, 0.053 mmol, 27%, *d.r.* = >20:1) as white solids.

Anti

^{1}H NMR (400 MHz, $CDCl_3$) δ = 7.23 (ddd, *J* = 8.0, 7.6, 1.8 Hz, 1H, H2), 7.14 (dd, *J* = 7.6, 1.8 Hz, 1H, H6), 7.02 (ddd, *J* = 7.6, 7.6, 1.2 Hz, 1H, H1), 6.96 (dd, *J* = 8.0, 1.2 Hz, 1H, H3), 4.68 (s, 1H, H12), 3.92 (dd, *J* = 9.4, 9.4 Hz, 1H, H7), 3.04 (dtd, *J* = 9.4, 8.6, 1.2 Hz, 1H, H10), 2.81 – 2.66 (m, 1H, H9"), 2.51 (ddd, *J* = 10.9, 8.6, 3.4 Hz, 1H, H9'), 2.29 (ddd, *J* = 10.9, 9.4, 2.9 Hz, 1H, H8) ppm.
^{13}C NMR (100 MHz, Acetone-*d*$_6$) δ = 153.7 (C4), 129.8 (C2), 129.0 (C6), 126.4 (C11), 123.6 (C1), 121.7 (C5), 119.7 (C3), 42.2 (C7), 31.1 (C10), 27.6 (C9) ppm.
^{11}B NMR (128 MHz, $CDCl_3$) δ = 32.56 ppm.
HRMS (ESI) calc. for $C_{11}H_{11}BNO_2$ $[M+H]^+$ 200.0877, found 200.0878.
IR (ATR): ῦ = 3349, 2949, 2235, 1739, 1584, 1489, 1365, 1322, 1262, 1232, 1217, 1156, 1095, 989, 851, 798, 755, 656 cm^{-1}.

Syn

^{1}H NMR (400 MHz, Acetone-*d*$_6$) δ = 8.01 (s, 1H, H12), 7.20 (dd, *J* = 7.6, 1.8 Hz, 1H, H2), 7.10 (dd, *J* = 7.7, 1.8 Hz, 1H, H6), 6.99 (td, *J* = 7.4, 1.3 Hz, 1H, H1), 6.92 (dd, *J* = 8.1, 1.3 Hz, 1H, H3), 4.01 (d, *J* = 9.1, 9.1 Hz, 1H, H7), 3.82 (ddd, *J* = 9.1, 5.1, 1.9 Hz, 1H, H10), 2.89 – 2.69 (m, 1H, H9"), 2.43 – 2.22 (m, 2H, H8+H9') ppm.
^{13}C NMR (100 MHz, Acetone-*d*$_6$) δ = 154.4 (C4), 130.5 (C6), 129.6 (C2), 124.3 (C11), 123.3 (C1), 121.0 (C5), 119.6 (C3), 38.0 (C10), 31.8 (C11), 27.2 (C9) ppm.
^{11}B NMR (128 MHz, Acetone-*d*$_6$) δ = 31.38 ppm.
HRMS (ESI) calc. for $C_{11}H_{11}BNO_2$ $[M+H]^+$ 200.0877, found 200.0922.
IR (ATR): ῦ = 3369, 3206, 2241, 1737, 1581, 1487, 1433, 1391, 1357, 1316, 1289, 1269, 1227, 1126, 1071, 1019, 1002, 949, 759, 738 cm^{-1}.

Methyl-3-hydroxy-1,2a,3,8b-tetrahydro-2*H*-benzo[*e*]cyclobuta[*c*][1,2]oxaborinine-1-carboxylate (4)

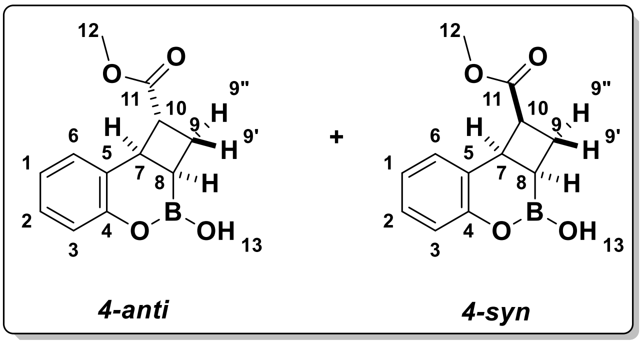

Prepared according to General Procedure **H**, **1** (29.2 mg, 0.2 mmol, equiv.) and methyl acrylate (0.91 mL, 10 mmol, 50 equiv.) and Ir(dFppy)$_3$ (1.5 mg, 1 mol%) were irradiated for 16 h with blue light (440 nm, 1 W). Upon completion, internal standard (1,3,5-trimethoxybenzene), as a solution in MeCN, was added and the mixture was concentrated under reduced pressure, the crude (94% NMR yield, *d.r.* = 1:1) was dissolved in EtOAc (10 mL) and *n*-hexane (10 mL) was added and the mixture was filtered. The filtrated was concentrated under reduced pressure and purification by flash column chromatography (C18, 10→100% MeCN/water) yielded **4** as a light-yellow oil (22.7 mg, 0.098 mg, 71%, *d.r.* = 1:1).

^{1}H NMR (600 MHz, $CDCl_3$) δ = 7.16 (td, *J* = 7.7, 1.7 Hz, 1H, H2-*anti*), 7.14 – 7.10 (m, 1H, H2-*syn*), 7.08 (dd, *J* = 7.6, 1.7 Hz, 1H, H6-*anti*), 7.00 – 6.86 (m, 5H, H6-*syn*+H1-*syn*+H1*anti*+H3-*anti*+H3-*syn*), 4.86 (s, 1H, H13-*anti*), 4.72 (s, 1H, H13-*syn*), 4.05 (t, *J* = 10.1 Hz, 1H, H7-*syn*), 3.82 (dd, *J* = 10.3, 10.1 Hz, 1H, H7-*anti*), 3.71 (s, 3H, H12-*anti*), 3.48 (ddd, *J* = 8.5, 3.5, 1.8 Hz, 1H, H10-*syn*), 3.28 (s, 3H, H12-*syn*), 3.17 – 3.08 (m, 1H, H12-*anti*), 2.65 (ddd, *J* = 10.9, 10.7, 3.5 Hz, 1H, H9''-*anti*), 2.57 (ddd, *J* = 11.4, 10.9, 3.0 Hz, 1H, H9''-*syn*), 2.44 (ddd, *J* = 11.4, 7.4, 3.0 Hz, 1H, H9'-*syn*), 2.34 (ddd, *J* =

10.9, 8.8, 3.5 Hz, 1H, H9’-*anti*), 2.28 – 2.20 (m, 1H, H8-*syn*), 2.14 (td, *J* = 10.3, 3.7 Hz, 1H, H8-*anti*) ppm.
^{13}C NMR (151 MHz, $CDCl_3$) δ = 175.1 (C11-*syn*), 174.5 (C11-*anti*), 153.3 (C4-*syn*), 152.3 (C4-*anti*), 129.2 (C6-*syn*), 128.7 (C6-*anti*), 128.7 (C2-*syn*), 128.5 (C2-*anti*), 127.1 (C5-*anti*), 124.1 (C5-*syn*), 123.2 (C1-*anti*), 122.5 (C1-*syn*), 118.8 (C3-*anti*), 118.8 (C3-*syn*), 52.0 (C12-*anti*), 51.4 (C12-*syn*), 46.8 (C10-*syn*), 46.7 (C10-*anti*), 39.3 (C7-*anti*), 38.2 (C7-*syn*), 25.3 (C9-*anti*), 23.8 (C9-*syn*) ppm.
^{11}B NMR (192 MHz, $CDCl_3$) δ = 32.29 ppm.
HRMS (ESI) calc. for $C_{12}H_{13}BO_4Na^+$ $[M+Na]^+$ 255.0799, found 255.0780.
IR (ATR): ῡ = 3385, 3206, 2986, 2957, 1729, 1604, 1572, 1487, 1451, 1437, 1401, 1374, 1354, 1262, 1238, 1212, 1190, 1170, 1144, 1120, 1093, 1035, 998, 960, 946, 899, 847, 759, 688, 670, 646, 632 cm^{-1}.

Methyl-3-hydroxy-1-methyl-1,2a,3,8b-tetrahydro-2*H*-benzo[*e*]cyclobuta[*c*][1,2]oxaborinine-1-carboxylate (5)

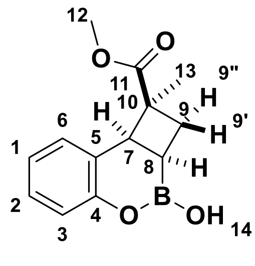

5

Prepared according to General Procedure **H**, **1** (29.2 mg, 0.2 mmol, equiv.) and methyl methacrylate (1.1 mL, 10 mmol, 50 equiv.) and $Ir(dFppy)_3$ (1.5 mg, 1 mol%) were irradiated for 16 h with blue light (440 nm, 1 W). Upon completion, internal standard (1,3,5-trimethoxybenzene), as a solution in MeCN, was added and the mixture was concentrated under reduced pressure, the crude (69% NMR yield, *d.r.* = 1.8:1) was dissolved in EtOAc (10 mL) and *n*-hexane (10 mL) was added and the mixture was filtered. The filtrated was concentrated under reduced pressure and purification by flash column chromatography (C18, 10→100% MeCN/water) yielded **5** as a yellow oil (21.2 mg, 0.081 mg, 41%, *d.r.* = 8:1).

^{1}H NMR (400 MHz, Acetone-*d*$_6$) δ = 7.61 (s, 1H, H14), 7.09 (td, *J* = 7.6, 1.9 Hz, 1H, H1), 6.93 (dd, *J* = 7.6, 1.9 Hz, 1H, H6), 6.89 – 6.82 (m, 1H, H1), 6.79 (dd, *J* = 8.1, 1.3 Hz, 1H, H3), 3.58 (d, *J* = 10.9 Hz, 1H, H7), 3.19 (s, 3H, H12), 2.73 – 2.59 (m, 1H, H9’), 2.23 (t, *J* = 11.2 Hz, 1H, H9”), 2.10 (td, *J* = 11.2, 4.0 Hz, 1H, H8), 1.45 (s, 3H, H13) ppm.
^{13}C NMR (100 MHz, Acetone-*d*$_6$) δ = 175.9 (C11), 154.3 (C4), 129.9 (C6), 129.0 (C2), 125.4 (C5), 122.5 (C1), 119.1 (C3), 53.2 (C10), 51.1 (C12), 47.4 (C7), 32.8 (C9), 24.9 (C13) ppm.
^{11}B NMR (128 MHz, $CDCl_3$) δ = 32.74 ppm.
HRMS (ESI) calc. for $C_{13}H_{15}BO_4Na^+$ $[M+Na]^+$ 269.0955, found 269.0937.
IR (ATR): ῡ = 3204, 2953, 1703, 1606, 1591, 1451, 1434, 1189, 1130, 884, 749, 725, 639 cm^{-1}.

3-Hydroxy-5-methoxy-1,2a,3,8b-tetrahydro-2*H*-benzo[*e*]cyclobuta[*c*][1,2]oxaborinine-1-carbonitrile (6)

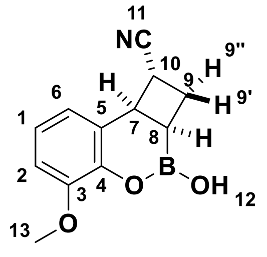

6

Prepared according to General Procedure **H**, **S15** (35.2 mg, 0.2 mmol, 1 equiv.), acrylonitrile (658 µL, 10 mmol, 50 equiv.) and $Ir(dFppy)_3$ (1.5 mg, 1 mol%) were irradiated for 16 h with blue light (440 nm, 1 W). Upon completion, internal standard (1,3,5-trimethoxybenzene), as a solution in MeCN, was added and the mixture was concentrated under reduced pressure, the crude (98% NMR yield, *d.r.* = 2:1) was dissolved in DCM (20 mL) and filtered and the filter was thoroughly washed with DCM. The filtrate was concentrated under reduced pressure and purification by flash column chromatography (C18, 10→100% MeCN/water) yielded **6** as a white solid (19.9 mg, 0.087 mmol, 43%, *d.r.* = >20:1).

^{1}H NMR (600 MHz, Acetone-d_6) δ = 8.17 (s, 1H, H12), 6.96 – 6.88 (m, 2H, H1+H2), 6.70 (dd, *J* = 6.3, 2.8 Hz, 1H, H6), 3.85 (dd, *J* = 10.0, 9.5 Hz, 1H, H7), 3.81 (s, 3H, H13), 3.23 (ddd, *J* = 10.0, 8.7, 1.2 Hz, 1H, H10), 2.65 (ddd, *J* = 11.2, 10.6, 10.0 Hz, 1H, H9"), 2.48 (ddd, *J* = 10.9, 8.6, 2.9 Hz, 1H, H9'), 2.26 – 2.19 (m, 1H, H8) ppm.
^{13}C NMR (100 MHz, Acetone-d_6) δ = 150.4 (C3), 143.1 (C4), 126.9 (C5), 123.3 (C2), 121.7 (C11), 120.5 (C1), 113.0 (C6), 56.2 (C13), 42.3 (C7), 31.0 (C10), 27.7 (C9) ppm.
^{11}B NMR (128 MHz, Acetone-d_6) δ = 32.30 ppm.
HRMS (ESI) calc. for $C_{12}H_{12}BNO_3Na^+$ $[M+Na]^+$ 252.0802, found 252.0781.
IR (ATR): ṽ = 3357, 2956, 2237, 1585, 1484, 1437, 1395, 1361, 1328, 1289, 1263, 1223, 1090, 970, 856, 841, 782, 735 cm^{-1}.

3-Hydroxy-7-methyl-1,2a,3,8b-tetrahydro-2*H*-benzo[*e*]cyclobuta[*c*][1,2]oxaborinine-1-carbonitrile (7)

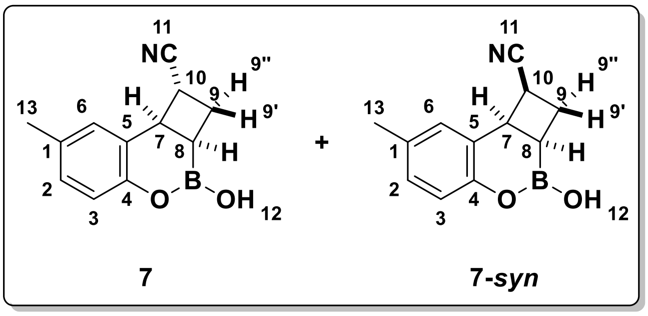

Prepared according to General Procedure **H**, **S16** (32.0 mg, 0.2 mmol, 1 equiv.), acrylonitrile (658 µL, 10 mmol, 50 equiv.) and Ir(dFppy)$_3$ (1.5 mg, 1 mol%) were irradiated for 16 h with blue light (440 nm, 1 W). Upon completion, internal standard (1,3,5-trimethoxybenzene), as a solution in MeCN, was added and the mixture was concentrated under reduced pressure, the crude (82% NMR yield, *d.r.* = 1.4:1) was resolved in DCM and filtered. The filtrate was concentrated under reduced pressure and purification by flash column chromatography (C18, 10→100% MeCN) yielded **7** (17.3 mg, 0.081 mmol, 41%, *d.r.* = >20:1) and **7-*syn*** (12.5 mg, 0.059 mmol, 29%, *d.r.* = >20:1) as white solids.

Anti
^{1}H NMR (400 MHz, Acetone) δ = 8.15 (bs, 1H, H12), 7.00 (dd, *J* = 8.3, 2.3 Hz, 1H, H2), 6.92 (d, *J* = 2.2 Hz, 1H, H6), 6.79 (d, *J* = 8.2 Hz, 1H, H3), 3.81 (t, *J* = 9.4 Hz, 1H, H7), 3.19 (dtd, *J* = 9.9, 8.7, 1.2 Hz, 1H, H10), 2.64 (q, *J* = 10.6 Hz, 1H, H9"), 2.46 (dddd, *J* = 10.7, 8.6, 3.4, 0.9 Hz, 1H, H9'), 2.25 (s, 3H, H13), 2.24 – 2.17 (m, 1H, H8) ppm.
^{13}C NMR (101 MHz, Acetone) δ = 151.5 (C4), 132.7 (C1), 130.2 (C2), 129.3 (C6), 126.0 (C5), 121.7 (C11), 119.4 (C3), 42.2 (C7), 31.0 (C10), 27.6 (C9), 20.5 (C13) ppm.
^{11}B NMR (128 MHz, Acetone) δ = 32.28 ppm.
HRMS (ESI) calc. for $C_{12}H_{13}BNO_2^+$ $[M+H]^+$ 214.1034, found 214.1020.
IR (ATR): ṽ = 3332, 2250, 1497, 1421, 1385, 1349, 1328, 1295, 1269, 1229, 1190, 1147, 1124, 1093, 1041, 991, 975, 883, 864, 831, 810, 785, 673, 647 cm^{-1}.

Syn
^{1}H NMR (400 MHz, Acetone) δ = 8.02 (s, 1H, H12), 7.00 (dd, *J* = 8.3, 2.2 Hz, 1H, H2), 6.89 (d, *J* = 2.2 Hz, 1H, H6), 6.81 (d, *J* = 8.2 Hz, 1H, H3), 3.96 (t, *J* = 9.1 Hz, 1H, H7), 3.84 – 3.75 (m, 1H, H10), 2.78 (q, *J* = 10.3 Hz, 1H, H9"), 2.37 – 2.26 (m, 2H, H9'+H8), 2.24 (s, 3H, H13) ppm.
^{13}C NMR (100 MHz, Acetone) δ = 152.3 (C4), 132.3 (C1), 130.8 (C6), 130.1 (C2), 123.9 (C11), 121.1 (C5), 119.3 (C3), 38.0 (C7), 31.8 (C10), 27.2 (C9), 20.6 (C13) ppm.
^{11}B NMR (128 MHz, Acetone) δ = 31.69 ppm.
HRMS (ESI) calc. for $C_{12}H_{13}BNO_2^+$ $[M+H]^+$ 214.1034, found 214.1035.

IR (ATR): ṽ = 3433, 2234, 1743, 1497, 1421, 1374, 1348, 1316, 1294, 1272, 1232, 1217, 1127, 1071, 1025, 1002, 904, 821, 800, 708, 655 cm^{-1}.

3-Hydroxy-7-(thiophen-3-yl)-1,2a,3,8b-tetrahydro-2*H*-benzo[*e*]cyclobuta[*c*][1,2]oxaborinine-1-carbonitrile (8)

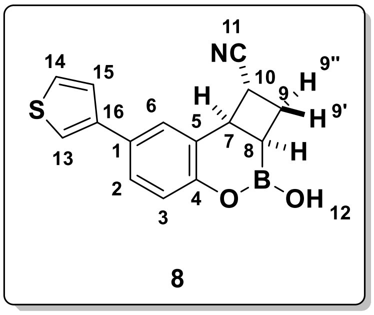

Prepared according to General Procedure **H**, **S17** (45.6 mg, 0.2 mmol, 1 equiv.), acrylonitrile (658 µL, 10 mmol, 50 equiv.) and Ir(dFppy)$_3$ (1.5 mg, 1 mol%) were irradiated for 16 h with blue light (440 nm, 1 W). Upon completion, internal standard (1,3,5-trimethoxybenzene), as a solution in MeCN, was added and the mixture was concentrated under reduced pressure, the crude (68% NMR yield, *d.r.* = 1.6:1) was resolved in DCM and filtered. The filtrate was concentrated under reduced pressure and purification by flash column chromatography (C18, 10→100% MeCN) yielded **8** as a white solid (20.6 mg, 0.084 mmol, 42%, *d.r.* = >20:1).

^{1}H NMR (400 MHz, Acetone) δ = 8.20 (s, 1H, H12), 7.66 (dd, *J* = 3.0, 1.4 Hz, 1H, H14), 7.58 – 7.40 (m, 4H, H2, H13, H15, H6), 6.96 (d, *J* = 8.3 Hz, 1H, H3), 3.94 (t, *J* = 9.4 Hz, 1H, H7), 3.35 – 3.23 (m, 1H, H10), 2.67 (q, *J* = 10.6 Hz, 1H, H9), 2.51 (ddd, *J* = 11.3, 8.6, 3.4 Hz, 1H, H9), 2.34 – 2.22 (m, 1H, H8) ppm.
^{13}C NMR (100 MHz, Acetone) δ = 152.1 (C4), 141.4 (C16), 130.7 (C1), 126.8 (C_{Ar}), 126.4 (C_{Ar}), 126.0 (C_{Ar}), 126.0 ($C_{Thiophene}$), 125.9 (C5), 120.8 (C11), 119.6 (C14), 119.2 (C3), 41.2 (C7), 30.3 (C10), 26.8 (C9) ppm.
^{11}B NMR (128 MHz, Acetone) δ = 32.45 ppm.
HRMS (ESI) calc. for $C_{15}H_{12}BNO_2SNa^+$ $[M+Na]^+$ 304.0574, found 304.0563.
IR (ATR): ṽ = 3368, 2927, 2235, 1703, 1493, 1388, 1365, 1322, 1273, 1236, 1156, 1127, 1090, 993, 887, 863, 831, 782, 649 cm^{-1}.

8-Fluoro-3-hydroxy-1,2a,3,8b-tetrahydro-2*H*-benzo[*e*]cyclobuta[*c*][1,2]oxaborinine-1-carbonitrile (9 and 9-*syn*)

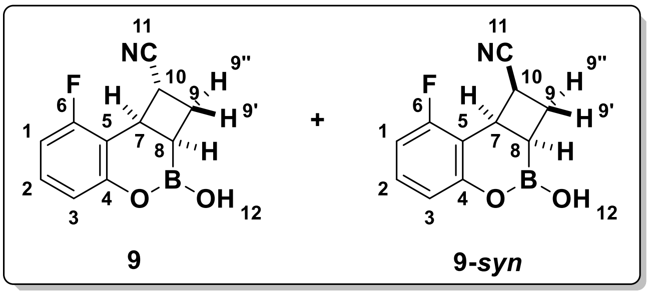

Prepared according to General Procedure **H**, **S18** (32.8 mg, 0.2 mmol, 1 equiv.), acrylonitrile (658 µL, 10 mmol, 50 equiv.) and Ir(dFppy)$_3$ (1.5 mg, 1 mol%) were irradiated for 16 h with blue light (440 nm, 1 W). Upon completion, internal standard (1,3,5-trimethoxybenzene), as a solution in MeCN, was added and the mixture was concentrated under reduced pressure, the crude (91% NMR yield, *d.r.* = 1:1) was dissolved in DCM and filtered. The filtrate was concentrated under reduced pressure and purification by flash column chromatography (C18, 10→100% MeCN/water) yielded **9** (15.1 mg, 0.070 mmol, 35%, *d.r.* = >20:1) and **9-*syn*** (14.8 mg, 0.068 mmol, 34%, *d.r.* = >20:1) as white solids.

Anti
^{1}H NMR (600 MHz, Acetone-d_6) δ = 8.25 (s, 1H, H12), 7.18 (ddd, *J* = 8.0 Hz, 7.8 Hz, 7.7 Hz, 1H, H2), 6.75 (dd, *J* = 8.3 Hz, 8.0 Hz, 1H, H1), 6.72 (d, *J* = 8.3 Hz, 1H, H3),4.10 (dd, *J* = 9.5 Hz, 9.3 Hz 1H, H7), 3.30 (ddd, *J* = 9.3 Hz, 8.9 Hz, 8.9 Hz, 1H, H10), 2,74 – 2,61 (m, 1H, H9”), 2.61 – 2.45 (m, 1H, H9’), 2.34 – 2,23 (m, 1H, H8) ppm.

^{13}**C NMR** (100 MHz, Acetone-*d*$_6$) δ = 161.3 (d, *J* = 243.8 Hz, C6), 155.1 (d, *J* = 6.1 Hz, C4), 130.1 (d, *J* = 10.2 Hz, C2), 121.6 (C11), 115.5 (d, *J* = 3.0 Hz, C3), 114.6 (d, *J* = 21.0 Hz, C5), 109.9 (d, *J* = 21.8 Hz, C1), 35.2 (C7), 30.4 (C10), 27.7 (C9) ppm.
^{11}**B NMR** (192 MHz, Acetone-*d*$_6$) δ = 32.21 ppm.
^{19}**F NMR** (564 MHz, Acetone-*d*$_6$) δ = -118.16 ppm.
HRMS (ESI) calc. for $C_{11}H_{10}BFNO_2^+$ $[M+H]^+$ 218.0783, found 218.0766.
IR (ATR): ῦ = 3359, 2940, 2235, 1700, 1622, 1585, 1467, 1394, 1344, 1315, 1273, 1229, 1189, 1087, 1062, 1008, 788, 757, 731 cm^{-1}.

Syn
^{1}**H NMR** (600 MHz, Acetone-*d*$_6$) δ = 8.16 (s, 1H, H12), 7.32 – 7.10 (m, 1H, H2), 6.86 – 6.47 (m, 2H, H1, H3), 4.14 (dd, *J* = 10.5, 9.7 Hz, 1H, H7), 3.86 (ddd, *J* = 9.7, 9.1, 5.7 Hz, 1H, H10), 2.90 – 2.77 (m, 1H, H9”), 2.39 (ddd, *J* = 10.5 Hz, 10.2 Hz, 6.4 Hz, 1H, H8), 2.31 (ddd, *J* = 12.3 Hz, 6.4, 5.7 Hz, 1H, H9’) ppm.
^{13}**C NMR** (151 MHz, Acetone-*d*$_6$) δ = 161.8 (d, *J* = 243.9 Hz, C6), 155.6 (C4), 130.3 (d, *J* = 10.6 Hz, C2), 120.9 (C11), 115.5 (d, *J* = 3.1 Hz, C3), 112.9 (d, *J* = 20.2 Hz, C5), 109.8 (d, *J* = 21.3 Hz, C1), 32.7 (C7), 30.9 (C8), 27.4 (C9) ppm.
^{11}**B NMR** (192 MHz, Acetone-*d*$_6$) δ = 31.51 ppm.
^{19}**F NMR** (564 MHz, Acetone-*d*$_6$) δ = -115.85 (t, *J* = 8.0 Hz) ppm.
HRMS (ESI) calc. for $C_{11}H_8BFNO_2^-$ $[M-H]^-$ 216.0638, found 216.0657.
IR (ATR): ῦ = 3295, 2247, 1624, 1585, 1470, 1329, 1272, 1226, 1212, 1173, 1150, 1123, 1039, 1002, 970, 894, 814, 787, 736, 604 cm^{-1}.

7-Bromo-3-hydroxy-1,2a,3,8b-tetrahydro-2*H*-benzo[*e*]cyclobuta[*c*][1,2]oxaborinine-1-carbonitrile (10 and 10-*syn*)

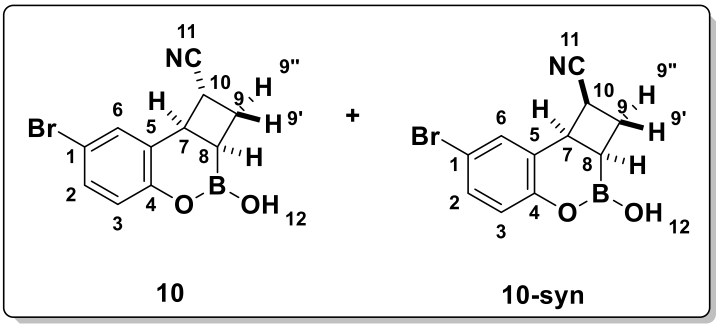

Prepared according to General Procedure **H**, **S19** (45.0 mg, 0.2 mmol, 1 equiv.), acrylonitrile (658 µL, 10 mmol, 50 equiv.) and Ir(dFppy)$_3$ (1.5 mg, 1 mol%) were irradiated for 16 h with blue light (440 nm, 1 W). Upon completion, internal standard (1,3,5-trimethoxybenzene), as a solution in MeCN, was added and the mixture was concentrated under reduced pressure, the crude (96% NMR yield, *d.r.* = 2:1) was resolved in DCM and filtered. The filtrate was concentrated under reduced pressure and purification by flash column chromatography (C18, 10→100% MeCN) yielded **10** (23.1 mg, 0.084 mmol, 42%, *d.r.* = >20:1) and **10-*syn*** (12.5 mg, 0.045 mmol, 23%, *d.r.* = >20:1) as white solids.

Anti
^{1}**H NMR** (400 MHz, Acetone-*d*$_6$) δ = 8.28 (s, 1H, H12), 7.35 (dd, *J* = 8.6, 2.5 Hz, 1H, H2), 7.32 (d, *J* = 2.5 Hz, 1H, H6), 6.88 (d, *J* = 8.6 Hz, 1H, H3), 3.91 (t, *J* = 9.5 Hz, 1H, H7), 3.32 (dtd, *J* = 9.9, 8.7, 1.2 Hz, 1H, H10), 2.67 (q, *J* = 10.6 Hz, 1H, H9’’), 2.57 – 2.44 (m, 1H, H9’), 2.34 – 2.20 (m, 1H, H8) ppm.
^{13}**C NMR** (100 MHz, Acetone-*d*$_6$) δ = 153.2 (C4), 132.5 (C2), 131.5 (C6), 128.9 (C11), 121.4 (C3), 121.8 (C10), 115.0 (C5), 41.6 (C7), 30.9 (C1), 27.6 (C9) ppm.
^{11}**B NMR** (128 MHz, Acetone-*d*$_6$) δ = 32.40 ppm.
HRMS (ESI) calc. for $C_{11}H_9BBrNO_2Na$ $[M+Na]^+$ 299,9802, found 299.9801.

IR (ATR): ῦ = 3428, 2951, 2238, 1477, 1411, 1379, 1338, 1312, 1286, 1267, 1233, 1221, 1193, 1121, 1070, 1026, 920, 882, 854, 836, 719, 640, 605 cm^{-1}.

Syn

^{1}**H NMR** (400 MHz, Acetone-*d₆*) δ = 8.19 (s, 1H, H12), 7.35 (dd, *J* = 8.6, 2.2 Hz, 1H, H2), 7.29 (d, *J* = 2.2 Hz, 1H, H6), 6.90 (d, *J* = 8.6 Hz, 1H, H3), 4.05 (t, *J* = 9.4 Hz, 1H, H7), 3.89 – 3.77 (m, 1H, H10), 2.81 (q, *J* = 10.3 Hz, 1H, H9"), 2.33 (ddt, *J* = 27.9, 11.4, 6.3 Hz, 1H, H8, H9') ppm.
^{13}**C NMR** (100 MHz, Acetone-d_6) δ = 153.0 (C4), 132.2 (C4), 131.5 (C6), 126.1 (C11), 120.8 (C3) 120.0 (C10), 114.0 (C5), 36.8 (C7), 31.0 (C1), 26.3 (C9) ppm.
^{11}**B NMR** (128 MHz, Acetone-d_6) δ = 32.56 ppm.
HRMS (ESI) calc. for $C_{11}H_8BBrNO_2$ $[M-H]^-$ 275.9837, found 275.9846.
IR (ATR): ῦ = 3290, 2950, 2251, 1481, 1415, 1386, 1345, 1326, 1283, 1264, 1230, 1188, 1154, 1121, 1093, 1073, 996, 973, 934, 907, 872, 858, 834, 799, 706, 667, 615 cm^{-1}

Methyl-7-fluoro-3-hydroxy-1,2a,3,8b-tetrahydro-2H-benzo[e]cyclobuta[c][1,2]oxaborinine-1-carboxylate (11)

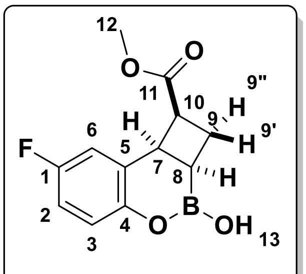

11

Prepared according to General Procedure **H**, **S20** (32.8 mg, 0.2 mmol, equiv.) and methyl acrylate (0.91 mL, 10 mmol, 50 equiv.) and Ir(dFppy)$_3$ (1.5 mg, 1 mol%) were irradiated for 16 h with blue light (440 nm, 1 W). Upon completion, internal standard (1,3,5-trimethoxybenzene), as a solution in MeCN, was added and the mixture was concentrated under reduced pressure, the crude (91% NMR yield, *d.r.* = 2:1) was dissolved in EtOAc (10 mL) and *n*-hexane (10 mL) was added and the mixture was filtered. The filtrated was concentrated under reduced pressure and purification by flash column chromatography (C18, 10→100% MeCN/water) yielded **11** as a yellow oil (23.0 mg, 0.092 mmol, 46%, *d.r.* = 14:1).

^{1}**H NMR** (400 MHz, Acetone-d_6) δ =7.72 (s, 1H, H13), 6.87 (td, *J* = 8.5, 3.1 Hz, 1H, H2), 6.80 (dd, *J* = 8.9, 5.0 Hz, 1H, H3), 6.73 (dd, *J* = 9.0, 3.1 Hz, 1H, H6), 4.07 (t, *J* = 10.1 Hz, 1H, H7), 3.52 (dddd, *J* = 9.9, 8.4, 3.9, 1.6 Hz, 1H, H10), 3.27 (s, 3H, H12), 2.52 (td, *J* = 11.3, 8.3 Hz, 1H, H9'), 2.43 – 2.30 (m, 1H, H9''), 2.27 – 2.11 (m, 1H, H8) ppm.
^{13}**C NMR** (101 MHz, Acetone-d_6) δ = 174.6 (C11), 158.2 (d, *J* = 237.8 Hz, C1), 150.9 (d, *J* = 2.4 Hz, C4), 126.8 (d, *J* = 7.5 Hz, C5), 120.3 (d, *J* = 8.2 Hz, C3), 115.9 (d, *J* = 22.8 Hz, C6), 115.2 (d, *J* = 23.1 Hz, C2), 51.2 (C12), 46.9 (C10), 38.5 (C7), 24.2 (C9) ppm.
^{11}**B NMR** (128 MHz, Acetone-d_6) δ = 31.84 ppm.
^{19}**F NMR** (376 MHz, Acetone-d_6) δ= -124.03 (td, *J* = 8.5, 5.1 Hz) ppm.
HRMS (ESI) calc. for $C_{12}H_{12}BFO_4Na^+$ $[M+Na]^+$ 273.0705, found 273.0685.
IR (ATR): ῦ = 3382, 3359, 2955, 1729, 1708, 1601, 1499, 1479, 1434, 1411, 1339, 1296, 1272, 1245, 1223, 1163, 1137, 1097, 1071, 1041, 999, 963, 948, 923, 869, 821, 788, 764, 728, 703, 652 cm^{-1}.

Methyl 1-cyano-3-hydroxy-1,2a,3,8b-tetrahydro-2*H*-benzo[*e*]cyclobuta[*c*][1,2]oxaborinine-6-carboxylate (12)

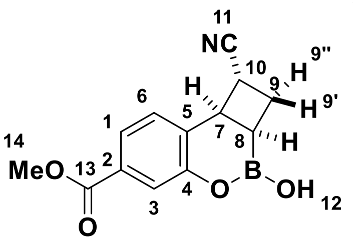

12

Prepared according to General Procedure **H**, **S21** (34.8 mg, 0.17 mmol, equiv.) and acrylonitrile (559 µL, 8.5 mmol, 50 equiv.) and Ir(dFppy)$_3$ (1.3 mg, 1 mol%) were irradiated for 16 h with blue light (440 nm, 1 W). Upon completion, internal standard (1,3,5-trimethoxybenzene), as a solution in MeCN, was added and the mixture was concentrated under reduced pressure, the crude (59% NMR yield, *d.r.* = 2.4:1) was resolved in DCM and filtered. The filtrate was concentrated under reduced pressure and purification by flash column chromatography (C18, 10→100% MeCN) yielded **10** as a white solid (16.2 mg, 0.063 mmol, 37%, *d.r.* = >20:1)

^{1}H NMR (400 MHz, Acetone) δ = 8.35 (s, 1H, H12), 7.63 (d, *J* = 8.0 Hz, 1H, H1), 7.48 (s, 1H, H3), 7.27 (d, *J* = 7.9 Hz, 1H, H6), 3.97 (t, *J* = 9.5 Hz, 1H, H7), 3.87 (s, 3H, H14), 3.33 (q, *J* = 9.2 Hz, 1H, H10), 2.69 (q, *J* = 10.7 Hz, 1H, H9"), 2.59 – 2.39 (m, 1H, H9'), 2.41 – 2.22 (m, 1H, H8) ppm.
^{13}C NMR (100 MHz, Acetone) δ = 166.7 (C13), 153.8 (C4), 131.8 (C5/C2), 131.6 (C5/C2), 129.3 (C6), 124.4 (C1), 121.5 (C11), 120.4 (C3), 52.4 (C14), 41.8 (C7), 30.8 (C10), 27.7 (C9).
^{11}B NMR (128 MHz, Acetone) δ = 32.44 ppm.
HRMS (ESI) calc. for $C_{13}H_{13}BNO_4^+$ [M+H]$^+$ 258.0932, found 258.0910.
IR (ATR): ṽ = 3385, 2955, 2235, 1708, 1573, 1502, 1439, 1391, 1281, 1219, 1157, 1120, 1095, 998, 892, 841, 803, 767, 718 cm^{-1}.

Methyl-3-phenyl-1,2,2a,3,4,8b-hexahydrobenzo[e]cyclobuta[c][1,2]azaborinine-1-carboxylate (13)

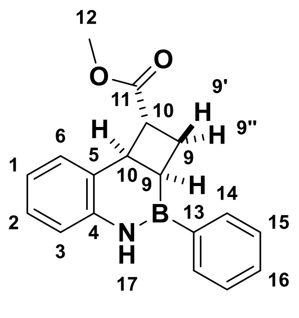

13

Prepared according to General Procedure **I**, **S22** (41 mg, 0.2 mmol, 1 equiv.) and methyl acrylate (91 µL, 1 mmol, 5 equiv.) were converted to **13** yielding an off-white solid (82% NMR yield, *d.r.* = 3:1, 31 mg, 0.11 mmol, 54%, *d.r.* = >20:1) after purification by flash column chromatography (C18, 20→100% MeCN/water).

^{1}H NMR (400 MHz, CDCl$_3$) δ = 7.69 (dd, *J* = 7.6, 1.8 Hz, 2H, H14), 7.49 – 7.41 (m, 3H, H15+H16), 7.14 (td, *J* = 7.6, 1.6 Hz, 1H, H2), 7.09 (dd, *J* = 7.5, 1.5 Hz, 1H, H6), 6.93 (td, *J* = 7.4, 1.2 Hz, 1H, H1), 6.81 (d, *J* = 6.7 Hz, 1H, H3), 6.79 (s, 1H, H17), 3.94 (t, *J* = 9.4 Hz, 1H, H7), 3.70 (s, 3H, H12), 3.24 (dtd, *J* = 10.0, 8.8, 1.2 Hz, 1H, H10), 2.90 – 2.80 (m, 1H, H9'), 2.67 (dddd, *J* = 11.4, 10.0, 3.4, 1.2 Hz, 1H, H8), 2.31 (dddd, *J* = 11.9, 8.8, 3.4, 1.0 Hz, 1H, H9") ppm.
^{13}C NMR (101 MHz, CDCl$_3$) δ = 175.0 (C11), 139.7 (C4), 132.7 (C14), 130.8 (C16), 128.9 (C6), 128.2 (C15), 128.1 (C5), 127.8 (C2), 122.6 (C1), 117.8 (C3), 51.8 (C12), 45.8 (C10), 40.0 (C7), 27.8 (C9) ppm.
^{11}B NMR (128 MHz, CDCl$_3$) δ = 43.34 ppm.
HRMS (ESI) calc. for $C_{18}H_{19}BNO_2^+$ [M+H]$^+$ 292.1504, found 292.1481.
IR (ATR): ṽ = 3379, 2949, 1717, 1596, 1476, 1457, 1427, 1302, 1271, 1242, 1203, 1149, 1067, 1039, 942, 820, 754, 701, 682 cm^{-1}.

3-Phenyl-1,2,2a,3,4,8b-hexahydrobenzo[*e*]cyclobuta[*c*][1,2]azaborinine-1-carbonitrile (14)

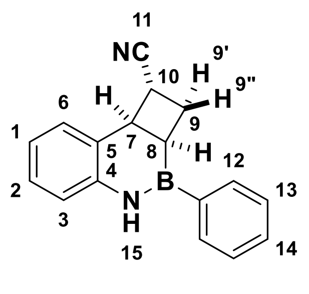

14

Prepared according to General Procedure **I**, **S22** (41 mg, 0.2 mmol, 1 equiv.) and acrylonitrile (66 µL, 1 mmol, 5 equiv.) were converted to **14** yielding an off-white solid (92% NMR-yield, *d.r.* = 4:1, 33 mg, 0.13 mmol, 63%, *d.r.* = >20:1), after purification by flash column chromatography (C18, 20→100% MeCN/water).

^{1}H NMR (600 MHz, $CDCl_3$) δ = 7.70 – 7.65 (m, 2H, H12), 7.53 – 7.48 (m, 1H, H14), 7.47 (tq, *J* = 6.1, 0.9 Hz, 2H, H13), 7.22 (td, *J* = 7.5, 1.2 Hz, 1H, H2), 7.17 (dd, *J* = 7.4, 1.6 Hz, 1H, H6), 7.00 (tt, *J* = 7.5, 0.9 Hz, 1H, H1), 6.86 (dd, *J* = 7.8, 1.2 Hz, 1H, H3), 6.84 (s, 1H, H15), 4.05 (t, *J* = 9.5 Hz, 1H, H7), 3.22 – 3.12 (m, 1H, H10), 2.95 (q, *J* = 10.8 Hz, 1H, H9'), 2.86 – 2.80 (m, 1H, H8), 2.51 – 2.44 (m, 1H, H9") ppm.
^{13}C NMR (151 MHz, $CDCl_3$) δ = 139.4 (C5), 132.6 (C12), 131.1 (C14), 128.7 (C2), 128.6 (C6), 128.3 (C13), 125.7 (C1), 123.1 (C5), 121.4 (C11), 118.1 (C3), 42.1 (C7), 29.7 (C10), 29.2 (C9) ppm.
^{11}B NMR = (192 MHz, $CDCl_3$) δ = 42.62 ppm.
HRMS (ESI) calc. for $C_{17}H_{15}BN_2Na^+$ $[M+Na]^+$ 281.1220, found 281.1208.
IR (ATR): ῦ = 3348, 2973, 2947, 2919, 2888, 2231, 1611, 1513, 1476, 1430, 1364, 1352, 1304, 1273, 1250, 1196, 1154, 1115, 1068, 1001, 988, 919, 817, 772, 761, 734, 702 cm^{-1}.

Methyl-1-methyl-3-phenyl-1,2,2a,3,4,8b-hexahydrobenzo[*e*]cyclobuta[*c*][1,2]azaborinine-1-carboxylate (15)

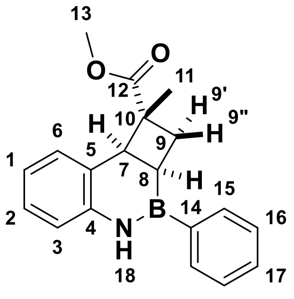

15

Prepared according to General Procedure **I**, **S22** (41 mg, 0.2 mmol, 1 equiv.) and methyl methacrylate (426 µL, 4 mmol, 20 equiv.) were converted to **15** yielding an off-white solid (86% NMR-yield, *d.r.* = 2:1, 32 mg, 0.10 mmol, 52%, *d.r.* = >20:1, containing 10% starting material) after purification by flash column chromatography (C18, 20→100% MeCN/water).

^{1}H NMR (400 MHz, $CDCl_3$) δ = 7.68 (d, *J* = 6.9 Hz, 2H, H15), 7.47 (h, *J* = 6.0 Hz, 3H, H16+H17), 7.14 (t, *J* = 7.6 Hz, 1H, H2), 7.09 (d, *J* = 7.5 Hz, 1H, H6), 6.95 (t, *J* = 7.4 Hz, 1H, H1), 6.79 (d, *J* = 7.8 Hz, 1H, H3), 6.63 (s, 1H, H18), 4.17 (d, *J* = 11.3 Hz, 1H, H7), 3.78 (s, 3H, H13), 3.17 (t, *J* = 11.8 Hz, 1H, H9'), 2.69 (td, *J* = 11.8, 4.7 Hz, 1H, H8), 2.00 (dd, *J* = 11.4, 4.7 Hz, 1H, H9"), 1.13 (s, 3H, H11) ppm.
^{13}C NMR (100 MHz, $CDCl_3$) δ = 178.2 (C12), 141.0 (C4), 132.8 (C15), 130.8 (C17), 130.2 (C6), 128.2 (C16), 127.8 (C2), 125.3 (C5), 122.6 (C3), 117.8 (C3), 52.1 (C13), 48.7 (C10), 41.5 (C7), 34.9 (C9), 20.0 (C11) ppm.
^{11}B NMR (128 MHz, $CDCl_3$) δ = 43.50 ppm.
HRMS (ESI) calc. for $C_{19}H_{21}BNO_2^+$ $[M+H]^+$ 306.1659, found 306.1593.
IR (ATR): ῦ = 3367, 2952, 1708, 1606, 1595, 1476, 1431, 1364, 1298, 1266, 1223, 1147, 1117, 1070, 909, 813, 754, 731, 693, 647 cm^{-1}.

Tert-butyl-3-phenyl-1,2,2a,3,4,8b-hexahydrobenzo[*e*]cyclobuta[*c*][1,2]azaborinine-1-carboxylate (16)

Prepared according to an adapted General Procedure **I**, **S22** (41 mg, 0.2 mmol, 1 equiv.) and *tert*-butyl acrylate (583 µL, 4 mmol, 20 equiv.) were converted to **16** yielding an off-white solid (82% NMR-

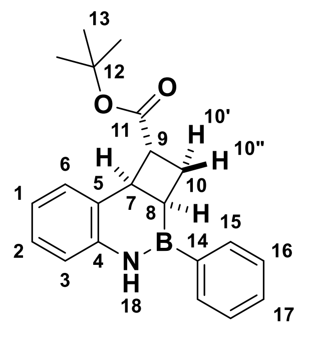

16

yield, *d.r.* = 4:1, 39 mg, 0.12 mmol, 55%, *d.r.* = >20:1, containing 10% hydrolyzed product) after purification by flash column chromatography (C18, 20→100% MeCN/water).

^{1}H NMR (400 MHz, $CDCl_3$) δ = 7.81 – 7.58 (m, 2H, H15), 7.51 – 7.37 (m, 3H, H16+H17), 7.13 (qd, *J* = 7.6, 1.6 Hz, 2H, H2), 6.93 (td, *J* = 7.4, 1.2 Hz, 1H, H1), 6.81 (dd, *J* = 7.8, 1.2 Hz, 1H, H3), 6.76 (s, 1H, H18), 3.87 (t, *J* = 9.4 Hz, 1H, H7), 3.22 – 3.07 (m, 1H, H9), 2.81 (q, *J* = 10.7 Hz, 1H, H10'), 2.64 (dddd, *J* = 11.3, 9.9, 3.5, 1.1 Hz, 1H, H8), 2.35 – 2.17 (m, 1H, H10''), 1.48 (s, 9H, H13) ppm.
^{13}C NMR (101 MHz, $CDCl_3$) δ = 174.0 (C11), 139.8 (C4), 132.7 (C15), 130.7 (C17), 129.1 (C6), 128.4 (C5), 128.2 (C16), 127.7 (C2), 122.5 (C1), 117.8 (C3), 80.2 (C12), 46.9 (C9), 40.2 (C7), 28.3 (C13), 27.7 (C10) ppm.
^{11}B NMR (128 MHz, $CDCl_3$) δ = 43.64 ppm.
HRMS (ESI) calc. for $C_{21}H_{24}BNO_2Na^+$ $[M+Na]^+$ 356.1792, found 356.1777.
IR (ATR): ῦ = 3371, 2975, 2932, 1720, 1608, 1595, 1476, 1456, 1427, 1367, 1302, 1273, 1245, 1147, 1041, 910, 846, 754, 699 cm^{-1}.

1-(3-Phenyl-1,2,2a,3,4,8b-hexahydrobenzo[*e*]cyclobuta[*c*][1,2]azaborinin-1-yl)ethan-1-one (17)

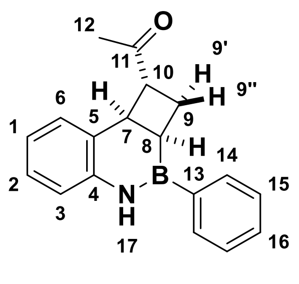

17

Prepared according to General Procedure **I**, **S22** (41 mg, 0.2 mmol, 1 equiv.) and methyl vinyl ketone (83 µL, 1 mmol, 5 equiv.) were converted to **17** yielding an off-white solid (74% NMR-yield, *d.r.* = 6:1, 33.5 mg, 0.11 mmol, 55%, *d.r.* = >20:1.), after purification by flash column chromatography (C18, 20→100% MeCN/water).

^{1}H NMR (400 MHz, $CDCl_3$) δ = 7.77 – 7.62 (m, 2H, H12), 7.54 – 7.36 (m, 3H, H15+H16), 7.14 (td, *J* = 7.6, 1.5 Hz, 1H, H2), 7.08 (dd, *J* = 7.5, 1.5 Hz, 1H, H6), 6.93 (td, *J* = 7.4, 1.2 Hz, 1H, H1), 6.85 – 6.80 (m, 1H, H3), 6.80 (s, 1H, H17), 3.87 (t, *J* = 9.3 Hz, 1H, H7), 3.37 (q, *J* = 9.0 Hz, 1H, H10), 2.78 (q, *J* = 10.6 Hz, 1H, H9'), 2.61 (dddd, *J* = 11.3, 9.9, 3.3, 1.2 Hz, 1H, H8), 2.35 – 2.17 (m, 1H, H9''), 2.04 (s, 3H, H12) ppm.
^{13}C NMR (100 MHz, $CDCl_3$) δ = 208.6 (C11), 139.9 (C4), 132.7 (C14), 130.8 (C16), 128.9 (C6), 128.4 (C5), 128.2 (C15), 127.8 (C2), 122.6 (C1), 117.9 (C3), 54.0 (C10), 39.4 (C7), 28.2 (C12), 27.1 (C9) ppm.
^{11}B NMR (128 MHz, $CDCl_3$) δ = 43.51 ppm.
HRMS (ESI) calc. for $C_{18}H_{19}BNO^+$ $[M+H]^+$ 276.1554, found 276.1548.
IR (ATR): ῦ = 3367, 2927, 1693, 1606, 1594, 1507, 1474, 1426, 1355, 1302, 1265, 1230, 1176, 1113, 1070, 1026, 999, 910, 861, 813, 754, 731, 698, 614 cm^{-1}.

3-Phenyl-1,2,2a,3,4,8b-hexahydrobenzo[*e*]cyclobuta[*c*][1,2]azaborinin-1-yl acetate (18)

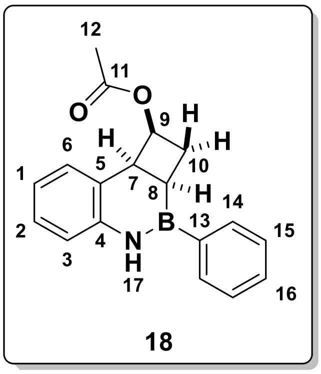

Prepared according to General Procedure **I**, **S22** (41 mg, 0.2 mmol, 1 equiv.) and vinyl acetate (92 µL, 1 mmol, 5 equiv.) were converted to **18** yielding an off-white solid (74% NMR-yield, *d.r.* = 1.4:1, 16.3 mg, 0.056 mmol, 28%, *d.r.* = >20:1.), after purification by flash column chromatography (C18, 20→100% MeCN/water).

^{1}H NMR (600 MHz, $CDCl_3$) δ = 7.71 – 7.63 (m, 2H, H14), 7.51 – 7.38 (m, 3H, H15+H16), 7.14 (td, *J* = 7.6, 1.6 Hz, 1H, H2), 7.07 (ddd, *J* = 7.4, 1.5, 0.7 Hz, 1H, H3), 6.92 (td, *J* = 7.4, 1.2 Hz, 1H, H1), 6.80 (dd, *J* = 7.8, 1.2 Hz, 1H, H6), 6.72 (s, 1H, H17), 5.09 – 4.93 (m, 1H, H9), 3.69 (dd, *J* = 10.2, 7.3 Hz, 1H, H7), 2.69 – 2.52 (m, 3H, H8+H10), 2.04 (s, 3H, H11) ppm.
^{13}C NMR (151 MHz, $CDCl_3$) δ = 170.4 (C11), 140.0 (C4), 132.7 (C14), 130.8 (C16), 128.9 (C3), 128.3 (C15), 128.0 (C2), 126.8 (C5), 122.5 (C1), 118.0 (C6), 74.2 (C9), 44.3 (C7), 34.5 (C10), 21.2 (C12) ppm.
^{11}B NMR (192 MHz, $CDCl_3$) δ = 43.05 ppm.
HRMS (ESI) calc. for $C_{18}H_{19}BNO_2^+$ $[M+H]^+$ 292.1504, found 292.1481.
IR (ATR): ῦ = 3385, 2962, 2926, 2854, 1713, 1609, 1517, 1477, 1431, 1372, 1360, 1338, 1315, 1245, 1216, 1117, 1103, 1070, 1042, 1021, 940, 912, 899, 884, 758, 706, 650, 626, 604 cm^{-1}.

Methyl-7-fluoro-3-phenyl-1,2,2a,3,4,8b-hexahydrobenzo[*e*]cyclobuta[*c*][1,2]azaborinine-1-carboxylate (19)

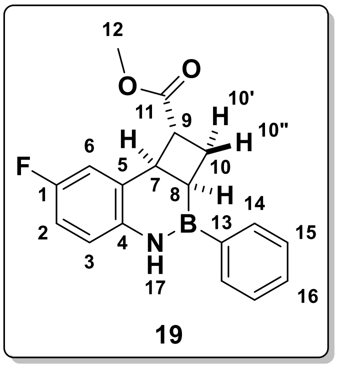

Prepared according to an adapted General Procedure **I**, **S23** (45 mg, 0.2 mmol, 1 equiv.) and methyl acrylate (91 µL, 1 mmol, 5 equiv.) were converted to **19** yielding an off-white solid (80% NMR-yield, *d.r.* = 5:1, 28.4 mg, 0.092 mmol, 46%, *d.r.* = >20:1) after purification by flash column chromatography (C18, 20→100% MeCN/water).

^{1}H NMR (400 MHz, $CDCl_3$) δ = 7.66 (dt, *J* = 6.3, 1.7 Hz, 2H, H14), 7.50 – 7.39 (m, 3H, H15+H16), 6.87 – 6.78 (m, 2H, H2+H6), 6.78 – 6.70 (m, 2H, H3+H17), 3.89 (t, *J* = 9.4 Hz, 1H, H7), 3.71 (s, 3H, H12), 3.26 – 3.19 (m, 1H, H9), 2.83 (q, *J* = 10.7 Hz, 1H, H10'), 2.64 (dddd, *J* = 11.3, 10.0, 3.5, 1.2 Hz, 1H, H8), 2.35 – 2.26 (m, 1H, H10") ppm.
^{13}C NMR (100 MHz, $CDCl_3$) δ = 174.6 (C11), 158.338 (d, *J* = 240.7 Hz, C1), 135.8 (d, *J* = 2.5 Hz, C4), 132.5 (C14), 130.7 (C16), 129.6 (d, *J* = 6.9 Hz, C5), 128.2 (C15), 118.3 (d, *J* = 8.0 Hz, C3), 115.4 (d, *J* = 22.1 Hz, C2), 113.9 (d, *J* = 22.5 Hz, C6), 51.8 (C12), 45.6 (C9), 39.9 (d, *J* = 1.3 Hz, C7), 27.7 (C10) ppm.
^{11}B NMR (128 MHz, $CDCl_3$) δ = 43.22 ppm.
^{19}F NMR (376 MHz, $CDCl_3$) δ = -121.44 (td, *J* = 8.5, 4.8 Hz) ppm.
HRMS (ESI) calc. for $C_{18}H_{18}BFNO_2^+$ $[M+H]^+$ 310.1409, found 310.1393.
IR (ATR): ῦ = 3359, 3223, 2955, 1708, 1601, 1499, 1479, 1434, 1410, 1338, 1296, 1272, 1245, 1223, 1163, 1097, 1041, 963, 948, 923, 869, 821, 764, 728, 703 cm^{-1}.

Methyl-7-chloro-3-phenyl-1,2,2a,3,4,8b-hexahydrobenzo[*e*]cyclobuta[*c*][1,2]azaborinine-1-carboxylate (20)

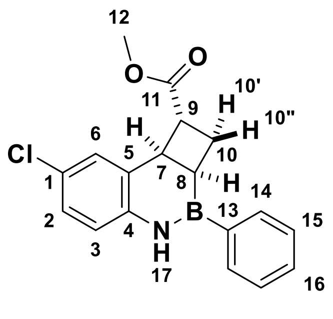

20

Prepared according to an adapted General Procedure **I**, **S24** (48 mg, 0.2 mmol, 1 equiv.) and methyl acrylate (91 µL, 1 mmol, 5 equiv.) were converted to **20** yielding an off-white solid (93% NMR-yield, *d.r.* = 5:1, 40 mg, 0.12 mmol, 61%) after purification by flash column chromatography (C18, 20→100% MeCN/water).

^{1}H NMR (400 MHz, $CDCl_3$) δ = 7.73 – 7.59 (m, 2H, H14), 7.54 – 7.36 (m, 3H, H15+H16), 7.15 – 6.96 (m, 2H, H2+H6), 6.73 (d, *J* = 8.2 Hz, 2H, H3+H17), 3.89 (t, *J* = 9.4 Hz, 1H, H7), 3.72 (s, 3H, H12), 3.21 (dtd, *J* = 10.0, 8.9, 1.2 Hz, 1H, H9), 2.83 (td, *J* = 11.1, 9.8 Hz, 1H, H10'), 2.70 – 2.61 (m, 1H, H8), 2.31 (dddd, *J* = 10.0, 8.9, 3.6, 1.0 Hz, 1H, H10") ppm.
^{13}C NMR (100 MHz, $CDCl_3$) δ = 174.7 (C11), 138.4 (C4), 132.7 (C14), 131.0 (C16), 129.8 (C5), 128.8 (C2), 128.3 (C15), 127.6 (C6), 127.2 (C1), 118.9 (C3), 51.9 (C12), 45.7 (C9), 39.6 (C7), 28.0 (C10) ppm.
^{11}B NMR (128 MHz, $CDCl_3$) δ = 43.38 ppm.
HRMS (ESI) calc. for $C_{18}H_{18}BClNO_2^+$ $[M+H]^+$ 326.1114, found 326.1101.
IR (ATR): ῦ = 3382, 3355, 1710, 1596, 1472, 1436, 1400, 1339, 1301, 1268, 1246, 1203, 1128, 1100, 1045, 1029, 950, 881, 823, 735, 703, 647 cm^{-1}.

Methyl-3-hydroxy-1,2,2a,3,4,8b-hexahydrobenzo[*e*]cyclobuta[*c*][1,2]azaborinine-1-carboxylate (21)

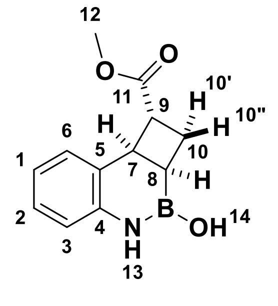

21

According to an adapted General Procedure **I**, **S25** (31.4 mg, 0.2 mmol, 1 equiv.) and methyl acrylate (0.36 mL, 4 mmol, 20 equiv.) were irradiated for 20 h (440 nm, 40 W). Upon completion, internal standard (1,3,5-trimethoxybenzene), as a solution in MeCN, was added and the mixture was concentrated under reduced pressure. The crude (62% NMR yield, *d.r.* = 4:1) was in dissolved in a mixture of THF in water (1:1, 4 mL) and stirred for 1 h. Upon completion the mixture was lyophilised overnight and the crude mixture was dissolved in EtOAc (10 mL) and *n*-hexane (10 mL) was added. Filtration was preformed to remove the formed polymer. The filtrate was concentrated under reduced pressure and flash column chromatography yielded the **21** as a white solid (19.9 mg, 0.086 mmol, 43%, *d.r.* = 4:1).

^{1}H NMR (600 MHz, $CDCl_3$) δ = 7.05 (td, *J* = 7.6, 1.6 Hz, 1H, H), 6.99 (dd, *J* = 7.5, 1.6 Hz, 1H), 6.79 (td, *J* = 7.4, 1.3 Hz, 1H), 6.59 (dd, *J* = 7.8, 1.2 Hz, 1H), 4.75 (s, 1H, H14), 3.82 (t, *J* = 9.5 Hz, 1H, H7), 3.69 (s, 3H, H12), 3.24 (dtd, *J* = 10.1, 8.7, 1.2 Hz, 1H, H9), 2.59 (q, *J* = 10.6 Hz, 1H, H10'), 2.31 – 2.19 (m, 1H, H10"), 2.04 (td, *J* = 9.9, 1.8 Hz, 1H, H8) ppm.
^{13}C NMR (100 MHz, $CDCl_3$) δ 175.0 (C11), 141.8 (C4), 128.6 (C6), 127.9 (C2), 126.4 (C5), 120.8 (C1), 116.8 (C3), 51.8 (C12), 45.6 (C9), 41.9 (C7), 25.9 (C10) ppm.
^{11}B NMR (128 MHz, $CDCl_3$) δ 34.44 ppm.
HRMS (ESI) calc. for $C_{12}H_{15}BNO_3^+$ $[M+H]^+$ 232.1140, found 232.1115.
IR (ATR): ῦ = 3376, 2950, 2512, 1708, 1606, 1478, 1432, 1394, 1360, 1319, 1286, 1234, 1191, 1170, 1108, 1040, 910, 752, 730 cm^{-1}.

Methyl-4-hydroxy-2,2a,4,8b-tetrahydro-1*H*-benzo[*c*]cyclobuta[*e*][1,2]oxaborinine-1-carboxylate (22)

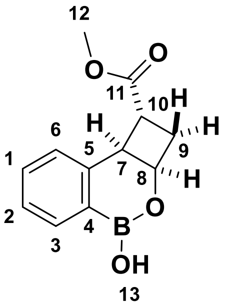

22

Prepared according to General Procedure **H**, **S26** (29.2 mg, 0.2 mmol, 1 equiv.) and methyl acrylate (0.91 mL, 10 mmol, 50 equiv.) were converted to **22** yielding a white solid (77% NMR yield, *d.r.* = 5:1, 24.6 mg, 0.11 mmol, 53%, *d.r.* = >20:1), after purification by flash column chromatography ($B(OH)_3$-SiO_2, 0→3% acetone/*n*-hexane).

R_f (20% EtOAc/*n*-hexane) = 0.17;

^{1}H NMR (400 MHz, $CDCl_3$) δ = 7.82 (dd, *J* = 7.4, 1.5 Hz, 1H, H3), 7.47 (td, *J* = 7.5, 1.5 Hz, 1H, H1), 7.30 (td, *J* = 7.4, 1.2 Hz, 1H, H2), 7.22 – 7.18 (m, 1H, H6), 4.97 (tdd, *J* = 7.0, 4.5, 1.1 Hz, 1H, H8), 4.77 (s, 1H, H13), 3.95 (tt, *J* = 6.7, 1.5 Hz, 1H, H7), 3.76 (s, 3H, H12), 3.19 (dtd, *J* = 9.9, 6.6, 1.2 Hz, 1H, H10), 2.71 (dtd, *J* = 12.5, 6.7, 2.1 Hz, 1H, H9”), 2.40 (dddd, *J* = 12.5, 9.8, 4.5, 1.0 Hz, 1H, H9’) ppm.

^{13}C NMR (100 MHz, $CDCl_3$) δ = 175.0 (C11), 145.4 (C5), 133.3 (C6), 132.5 (C1), 127.1 (C2), 126.9 (C3), 69.7 (C8), 52.2 (C12), 44.3 (C10), 43.6 (C7), 34.3 (C9) ppm.

^{11}B NMR (128 MHz, $CDCl_3$) δ = 27.89 ppm.

HRMS (ESI) calc. for $C_{12}H_{13}BO_4Na^+$ $[M+Na]^+$ 255.0799, found 255.0779.

IR (ATR): ῦ = 3385, 2988, 2959, 2850, 1727, 1604, 1573, 1487, 1451, 1437, 1400, 1375, 1354, 1311, 1262, 1239, 1213, 1170, 1144, 1120, 1093, 1035, 999, 960, 946, 899, 847, 824, 759, 688, 672, 632 cm^{-1}.

Tert-butyl 4-hydroxy-2,2a,4,8b-tetrahydro-1*H*-benzo[*c*]cyclobuta[*e*][1,2]oxaborinine-1-carboxylate (23)

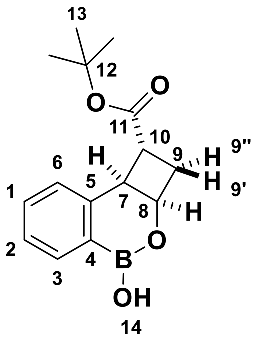

23

Prepared according to General Procedure **H**, **S26** (29.2 mg, 0.2 mmol) and *tert*-butyl acrylate (950 µL, 10 mmol) were converted to **23**, yielding a colourless oil (79% NMR yield, *d.r.* = 6:1, 31.1 mg, 59%, *d.r.* = 8:1) after purification by flash column chromatography ($B(OH)_3$-SiO_2, 0→15% Et_2O/*n*-hexane).

R_f (30% Et_2O/*n*-hexane) = 0.26

^{1}H NMR (400 MHz, $CDCl_3$) δ = 7.82 (dd, *J* = 7.4, 1.5 Hz, 1H, H3), 7.46 (td, *J* = 7.6, 1.5 Hz, 1H, H2), 7.34 – 7.28 (m, 1H, H1), 7.19 (d, *J* = 7.7 Hz, 1H, H6), 4.93 (tdd, *J* = 6.9, 4.2, 1.1 Hz, 1H, H8), 3.92 – 3.80 (m, 1H, H7), 3.11 (dtd, *J* = 9.7, 6.8, 1.1 Hz, 1H, H10), 2.66 (dtd, *J* = 12.5, 6.8, 2.0 Hz, 1H, H9”), 2.34 (dddd, *J* = 12.6, 9.7, 4.2, 1.0 Hz, 1H, H9’), 1.50 (s, 9H, H13) ppm.

^{13}C NMR (100 MHz, $CDCl_3$) δ = 173.8 (C11), 145.7 (C5), 133.3 (C6), 132.4 (C2), 127.2 (C3), 126.8 (C1), 80.9 (C12), 69.8 (C8), 45.7 (C10), 43.6 (C7), 34.0 (C9), 28.3 (C13) ppm.

^{11}B NMR (128 MHz, $CDCl_3$) δ = 27.85 ppm.

HRMS (ESI) calc. for $C_{15}H_{19}BO_4Na^+$ $[M+Na]^+$ 297.1268, found 297.1260.

IR (ATR): ῦ = 3367, 2992, 2932, 1713, 1602, 1572, 1489, 1456, 1380, 1361, 1311, 1291, 1252, 1239, 1226, 1156, 1141, 1093, 1028, 992, 975, 913, 876, 847, 744, 721, 682, 665, 627 cm^{-1}.

Methyl-4-hydroxy-1-methyl-2,2a,4,8b-tetrahydro-1*H*-benzo[*c*]cyclobuta[*e*][1,2]oxaborinine-1-carboxylate (24)

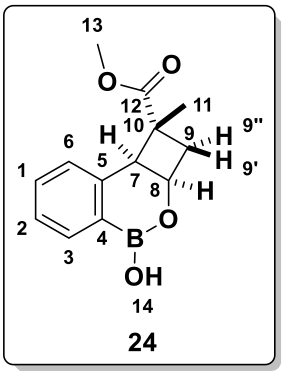

Prepared according to General Procedure **H**, **S26** (29.2 mg, 0.2 mmol) and (2-dimethylaminoethyl) methacrylate (1.1 mL, 10 mmol) were converted to **24** yielding a colourless oil (76% NMR yield, *d.r.* = 8:1, 24.1 mg, 49%, *d.r.* = >20:1) after purification by flash column chromatography ($B(OH)_3$-SiO_2, 0→1% acetone/*n*-hexane).

$\mathbf{R}_f$ (20% acetone/*n*-hexane) = 0.35

^{1}H NMR (400 MHz, $CDCl_3$) δ = 7.83 (dd, *J* = 7.4, 1.5 Hz, 1H, H3), 7.45 (td, *J* = 7.5, 1.5 Hz, 1H, H1), 7.30 (td, *J* = 7.4, 1.2 Hz, 1H, H2), 7.13 (d, *J* = 7.7 Hz, 1H, H6), 4.91 (td, *J* = 7.4, 3.9 Hz, 1H, H8), 4.78 (bs, 1H, H14), 4.15 (d, *J* = 7.7 Hz, 1H, H7), 3.78 (s, 3H, H13), 3.02 (ddd, *J* = 13.2, 7.2, 1.7 Hz, 1H, H9’’), 2.00 (ddd, *J* = 13.2, 3.9, 1.0 Hz, 1H, H9’), 1.07 (s, 3H, H11) ppm.

^{13}C NMR (100 MHz, $CDCl_3$) δ = 177.8 (C12), 142.0 (C5), 133.1 (C3), 132.0 (C1), 128.4 (C6), 126.8 (C2), 68.5 (C8), 52.4 (C13), 46.2 (C10), 45.8 (C7), 41.4 (C9), 21.6 (C11) ppm.

^{11}B NMR (128 MHz, $CDCl_3$) δ = 27.79 ppm.

HRMS (ESI) calc. for $C_{13}H_{16}BO_4^+$ $[M+H]^+$ 247.1136, found 247.1152.

IR (ATR): ῦ = 3440, 2932, 1728, 1601, 1572, 1487, 1453, 1395, 1368, 1334, 1305, 1235, 1135, 1105, 1059, 1034, 993, 890, 850, 764, 642 cm^{-1}.

2-(Dimethylamino)ethyl 4-hydroxy-1-methyl-2,2a,4,8b-tetrahydro-1*H*-benzo[*c*]cyclobuta[*e*][1,2]oxaborinine-1-carboxylate (25)

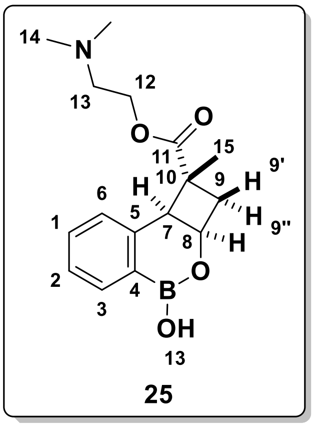

Prepared according to General Procedure **H**, **S26** (29.2 mg, 0.2 mmol) and (dimethylamino)ethyl methacrylate (1.7 mL, 10 mmol) were converted to **25** yielding a light brown oil (72% NMR yield, *d.r.* = 7:1, 27.4 mg, 45%, *d.r.* = >20:1) after purification by flash column chromatography (SiO_2, 0→100% Acetone/*n*-hexane).

$\mathbf{R}_f$ (100% Acetone) = 0.23

^{1}H NMR (400 MHz, $CDCl_3$) δ = 7.82 (dd, *J* = 7.4, 1.5 Hz, 1H, H3), 7.43 (dd, *J* = 7.5, 1.5 Hz, 1H, H1), 7.34 – 7.23 (m, 1H, H2), 7.13 (dd, *J* = 7.6, 1.1 Hz, 1H, H6), 4.88 (td, *J* = 7.4, 3.9 Hz, 1H, H8), 4.30 (td, *J* = 5.9, 1.8 Hz, 2H, H12), 4.13 (d, *J* = 7.6 Hz, 1H, H7), 3.01 (ddd, *J* = 13.1, 7.2, 1.7 Hz, 1H, H9”), 2.67 (t, *J* = 5.9 Hz, 2H, H13), 2.33 (s, 6H, H14), 1.96 (ddd, *J* = 13.0, 3.9, 1.0 Hz, 1H, H9’), 1.06 (s, 3H, H15) ppm.

^{13}C NMR (100 MHz, $CDCl_3$) δ = 177.3 (C11), 142.0 (C5), 133.2 (C3), 131.8 (C1), 128.4 (C6), 126.7 (C2), 68.3 (C8), 63.0 (C12), 57.7 (C13), 46.2 (C10), 45.8 (C7), 45.8 (C14), 41.4 (C9), 21.6 (C15) ppm.

^{11}B NMR (128 MHz, $CDCl_3$) δ = 28.08 ppm.

HRMS (ESI) calc. for $C_{16}H_{23}BNO_4^+$ $[M+H]^+$ 304.1714. found 304.1700.

IR (ATR): ῦ = 2942, 2830, 2784, 1723, 1601, 1571, 1486, 1453, 1393, 1371, 1298, 1232, 1134, 1097, 1011, 958, 912, 859, 765, 732, 663, 643 cm^{-1}.

4-Hydroxy-2,2a,4,8b-tetrahydro-1*H*-benzo[*c*]cyclobuta[*e*][1,2]oxaborinin-1-yl acetate (26)

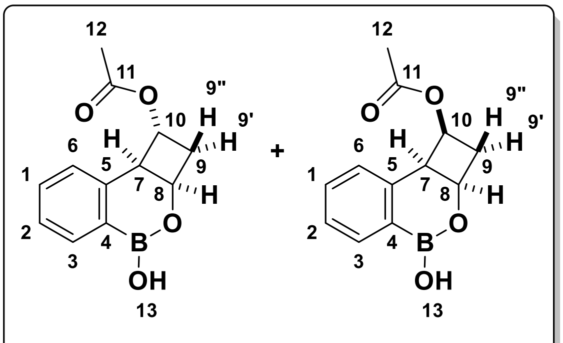

26-anti ***26-syn***

Prepared according to General Procedure **H**, **S26** (29.2 mg, 0.2 mmol) and vinyl acetate (950 µL, 10 mmol) were converted to **26**, yielding a colourless oil (64% NMR yield, *d.r.* = 1:1, 24.3 mg, 52%, *d.r.* = 1:1) after purification by flash column chromatography ($B(OH)_3$-SiO_2, 0→7% acetone/*n*-hexane).

$\mathbf{R}_f$ (20% EtOAc/*n*-hexane) = 0.34

^{1}H NMR (400 MHz, $CDCl_3$) δ = 7.85 (d, *J* = 7.4 Hz, 1H, H6-*syn*), 7.81 (d, *J* = 6.6 Hz, 1H, H6-*anti*), 7.46 (dtd, *J* = 11.9, 7.6, 1.6 Hz, 2H, H2-*syn*, H2-*anti*), 7.31 (t, *J* = 7.4 Hz, 3H, H1-*syn*, H1-*anti*, H3-*anti*), 7.01 (d, *J* = 7.8 Hz, 1H, H3-*syn*), 5.18 (q, *J* = 7.4 Hz, 1H, H10-*syn*), 5.04 (dt, *J* = 7.7, 5.4 Hz, 1H, H10-*anti*), 4.99 (dt, *J* = 7.2, 3.6 Hz, 1H, H8-*anti*), 4.68 (q, *J* = 7.1 Hz, 1H, H8-*syn*), 4.21 (td, *J* = 7.5, 3.4 Hz, 1H, H7-*syn*), 3.71 (ddd, *J* = 7.6, 5.1, 2.5 Hz, 1H, H7-*anti*), 2.95 (dtd, *J* = 12.4, 6.9, 3.5 Hz, 1H, H9'-*syn*), 2.62 (ddd, *J* = 12.3, 7.6, 4.2 Hz, 1H, H9"-*anti*), 2.49 (dddd, *J* = 13.1, 7.6, 5.3, 2.6 Hz, 1H, H9'-*anti*), 2.32 (dt, *J* = 12.4, 7.3 Hz, 1H, H9"-*syn*), 2.10 (s, 3H, H12-*anti*), 1.87 (s, 3H, H12-*syn*) ppm.

^{13}C NMR (100 MHz, $CDCl_3$) δ = 170.8 (C11-*syn)*, 170.6 (C11-*anti*), 143.7 (C5-*anti*), 140.5 (C5-*syn*), 133.3 (C6-*anti*), 133.3 (C6-*syn*), 132.4 (C2-*anti*), 131.7 (C2-*syn*), 129.1 (C3-*syn*), 127.4 (C3-*anti*), 127.0 (C1-*anti*), 126.6 (C1-*syn*), 75.1 (C10-*anti*), 67.9 (C8-*anti*), 65.4 (C10-*syn*), 65.0 (C8-*syn*), 47.8 (C7-*anti*), 46.4 (C7-*syn*), 40.0 (C9-*syn*), 38.7 (C9-*anti*), 21.1 (C12-*anti*), 20.9 (C12-*syn*) ppm.

^{11}B NMR (128 MHz, $CDCl_3$) δ = 27.81 ppm.

HRMS (ESI) calc. for $C_{12}H_{13}BO_4Na^+$ $[M+Na]^+$ 255.0799, found 255.0771.

IR (ATR): 3413, 2971, 2901, 1735, 1719, 1600, 1572, 1487, 1451, 1359, 1309, 1231, 1056, 1012, 976, 897, 854, 765, 640 cm^{-1}.

Methyl-8-fluoro-4-hydroxy-2,2a,4,8b-tetrahydro-1*H*-benzo[*c*]cyclobuta[*e*][1,2]oxaborinine-1-carboxylate (27)

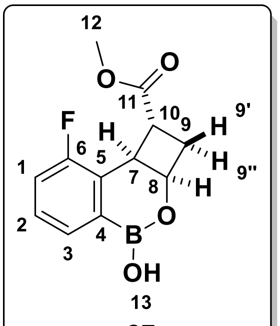

27

Prepared according to General Procedure **H**, **S27** (29.2 mg, 0.2 mmol, 1 equiv.) and methyl acrylate (0.91 mL, 10 mmol, 50 equiv.) were converted to **27**, yielding a white solid (74% NMR yield, *d.r.* = 5:1, 22.4 mg, 0.090 mmol, 45%, *d.r.* = >20:1), after purification by flash column chromatography ($B(OH)_3$-SiO_2, 0→1% acetone/*n*-hexane).

Rf (10% acetone/*n*-hexane) = 0.13

^{1}H NMR (400 MHz, $CDCl_3$) δ = 7.62 (dd, *J* = 7.3, 1.2 Hz, 1H), 7.33 – 7.27 (m, 1H), 7.16 (ddd, *J* = 9.5, 8.2, 1.2 Hz, 1H), 5.05 (tdd, *J* = 7.2, 5.3, 1.1 Hz, 1H, H8), 4.11 (t, *J* = 6.7 Hz, 1H, H7), 3.76 (s, 3H, H12), 3.24 – 3.09 (m, 1H, H10), 2.73 (dddd, *J* = 12.7, 7.0, 5.7, 2.3 Hz, 1H, H9'), 2.41 (dddd, *J* = 12.6, 10.0, 5.3, 1.0 Hz, 1H, H9'') ppm.

^{13}C NMR (101 MHz, $CDCl_3$) δ = 174.8 (C11), 159.9 (d, *J* = 248.2 Hz, C6), 131.6 (d, *J* = 14.2 Hz, C5), 129.0 (d, *J* = 3.6 Hz, C3), 128.5 (d, *J* = 7.2 Hz, C2), 118.9 (d, *J* = 20.4 Hz, C1), 69.3 (C8), 52.3 (C12), 42.7 (C10), 38.4 (d, *J* = 1.5 Hz, C7), 34.6 (C9) ppm.

^{11}B NMR (128 MHz, $CDCl_3$) δ = 27.22 ppm.

^{19}F NMR (376 MHz, $CDCl_3$) δ = -118.64 (dd, *J* = 9.7, 5.0 Hz) ppm.

HRMS (ESI) calc. for $C_{12}H_{13}BFO_4$ $[M+H]^+$ 251.0885, found 251.0880.
IR (ATR): ῦ = 3391, 2955, 2923, 2851, 1721, 1614, 1460, 1436, 1400, 1358, 1304, 1286, 1242, 1220, 1166, 1153, 1034, 1015, 879, 846, 800, 741, 685, 662 cm^{-1}.

Methyl-6-chloro-4-hydroxy-2,2a,4,8b-tetrahydro-1*H*-benzo[*c*]cyclobuta[*e*][1,2]oxaborinine-1-carboxylate (28)

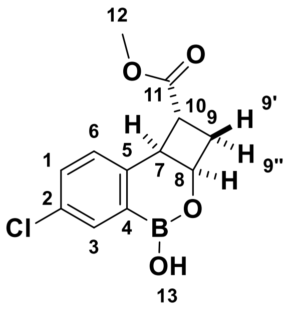

28

Prepared according to General Procedure **H**, **S28** (36.0 mg, 0.2 mmol) and methyl acrylate (0.91 mL, 10 mmol) were converted to **28** yielding a white solid (67% NMR yield, *d.r.* = 12:1, 23.6 mg, 0.089 mmol, 44%, *d.r.* = >20:1) after purification by flash column chromatography ($B(OH)_3$-SiO_2, 0→20% Et_2O/*n*-hexane).

Rf (30% acetone/*n*-hexane) = 0.31
^{1}H NMR (400 MHz, $CDCl_3$) δ = 7.78 (d, *J* = 2.3 Hz, 1H, H3), 7.41 (dd, *J* = 8.2, 2.3 Hz, 1H, H1), 7.13 (d, *J* = 8.2 Hz, 1H, H6), 5.02 – 4.90 (m, 2H, H8+H13), 3.90 (t, *J* = 6.9 Hz, 1H, H7), 3.15 (dt, *J* = 9.4, 6.6 Hz, 1H, H10), 2.70 (dtd, *J* = 13.3, 6.8, 1.9 Hz, 1H, H9"), 2.38 (ddd, *J* = 13.2, 9.7, 4.3 Hz, 1H, H9') ppm.
^{13}C NMR (100 MHz, $CDCl_3$) δ = 174.7 (C11), 143.5 (C5), 133.1 (C3), 133.1 (C2), 132.6 (C1), 128.7 (C6), 69.8 (C8), 52.3 (C12), 44.3 (C10), 42.9 (C7), 34.2 (C9) ppm.
^{11}B NMR (128 MHz, $CDCl_3$) δ = 27.58 ppm.
HRMS (ESI) calc. for $C_{12}H_{13}BClO_4$ $[M+H]^+$ 267.0590, found 267.0590.
IR (ATR): ῦ = 3441, 2955, 2917, 2850, 1704, 1592, 1563, 1479, 1416, 1390, 1347, 1294, 1249, 1222, 1171, 1153, 1098, 1082, 1032, 1004, 894, 840, 827, 794, 705, 649 cm^{-1}.

4-Hydroxy-N,N-dimethyl-2,2a,4,8b-tetrahydro-1*H*-benzo[*c*]cyclobuta[*e*][1,2]oxaborinine-1-carboxamide (29)

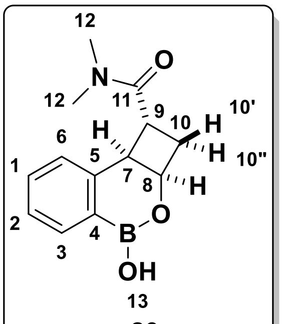

29

Prepared according to General Procedure **H**, **S26** (29.2 mg, 0.2 mmol) and *N,N*-dimethylacrylamide (1.0 mL, 10 mmol) were converted to **29** yielding a colourless oil (65% NMR yield, *d.r.* = 10:1, 24.0 mg, 49%, *d.r.* = 10:1) after purification by flash column chromatography (SiO_2, 0→100% acetone/*n*-hexane).

R_f (100% EtOAc) = 0.25
^{1}H NMR (400 MHz, $CDCl_3$) δ = 7.84 (dd, *J* = 7.3, 1.5 Hz, 1H, H3), 7.41 (td, *J* = 7.5, 1.5 Hz, 1H, H1), 7.27 (ddd, *J* = 7.5, 6.0, 1.2 Hz, 1H, H2), 7.22 (d, *J* = 7.6 Hz, 1H, H6), 5.35 (s, 1H, H13), 4.97 – 4.87 (m, 1H, H8), 4.15 (t, *J* = 7.1 Hz, 1H, H7), 3.42 (dt, *J* = 9.6, 7.8 Hz, 1H, H9), 2.97 (s, 3H, H12), 2.84 (s, 3H, H12), 2.58 (dddd, *J* = 12.2, 7.8, 6.3, 1.4 Hz, 1H, H9"), 2.38 (ddd, *J* = 12.4, 9.4, 3.1 Hz, 1H, H9') ppm.
^{13}C NMR (100 MHz, $CDCl_3$) δ = 173.2 (C11), 146.1 (C5), 133.4 (C3), 132.3 (C1), 127.2 (C6), 126.7 (C2), 70.2 (C8), 44.4 (C9), 42.2 (C7), 36.9 (C12), 35.7 (C12), 34.8 (C10) ppm.
^{11}B NMR (128 MHz, $CDCl_3$) δ = 28.30 ppm.
HRMS (ESI) calc. for $C_{13}H_{17}BNO_3^+$ $[M+H]^+$ 246.1296, found 246.1275
IR (ATR): ῦ = 3315, 2937, 2245, 1621, 1602, 1571, 1486, 1453, 1391, 1362, 1308, 1263, 1192, 1143, 1087, 1029, 989, 909, 871, 765, 725, 658, 642 cm^{-1}.

(4-Hydroxy-2,2a,4,8b-tetrahydro-1*H*-benzo[*c*]cyclobuta[*e*][1,2]oxaborinin-1-yl)(morpholino)methanone (30)

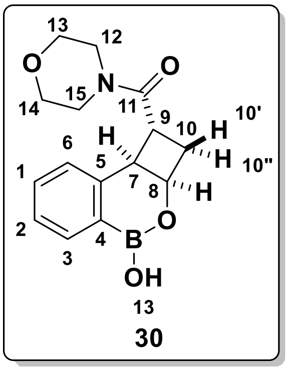

Prepared according to General Procedure **H**, **S26** (29.2 mg, 0.2 mmol) and 1-morpholinoprop-2-en-1-one (1.4 g, 10 mmol) were converted to **30** yielding a brown oil (24.5 mg, 43%, *d.r.* = 13:1) after purification by flash column chromatography (SiO_2, 0→100% acetone/*n*-hexane).

R_f (50% EtOAc/*n*-hexane) = 0.24

^{1}H NMR (400 MHz, $CDCl_3$) δ = 7.86 (dd, *J* = 7.4, 1.5 Hz, 1H, H3), 7.44 (td, *J* = 7.5, 1.5 Hz, 1H, H1), 7.31 (dd, *J* = 7.4, 1.2 Hz, 1H, H2), 7.24 (d, *J* = 8.0 Hz, 1H, H6), 5.51 (s, 1H, H13), 4.93 (tdd, *J* = 6.5, 2.8, 1.0 Hz, 1H, H8), 4.16 (t, *J* = 7.1 Hz, 1H, H7), 3.74 – 3.64 (m, 4H, $H_{Morpholine}$), 3.58 (ddd, *J* = 5.6, 3.6, 1.7 Hz, 2H, $H_{Morpholine}$), 3.42 (dtd, *J* = 9.1, 7.9, 1.0 Hz, 1H, H9), 3.29 – 3.21 (m, 2H, $H_{Morpholine}$), 2.75 – 2.59 (m, 1H, H10”), 2.55 – 2.31 (m, 1H, H10’) ppm.

^{13}C NMR (100 MHz, $CDCl_3$) δ = 171.8 (C11), 145.7 (C5), 133.5 (C3), 132.3 (C1), 127.1 (C2), 126.9 (C6), 70.2 (C8), 67.0 ($C_{Morpholine}$), 66.7 ($C_{Morpholine}$), 45.7 ($C_{Morpholine}$), 44.1 (C9), 42.4 ($C_{Morpholine}$), 42.2 (C7), 34.7 (C10).

^{11}B NMR (128 MHz, $CDCl_3$) δ = 28.30.

HRMS (ESI) calc. for $C_{15}H_{19}BNO_4^+$ $[M+H]^+$ 288.1402, found 288.1407.

IR (ATR): ῦ = 3342, 2858, 2247, 1622, 1602, 1571, 1484, 1453, 1437, 1390, 1361, 1305, 1269, 1236, 1138, 1113, 1068, 1029, 988, 910, 867, 765, 725, 642 cm^{-1}.

Diethyl 5-hydroxy-1,1a,2,10b-tetrahydro-5*H*-benzo[*e*]cyclopenta[1,4]cyclobuta[1,2-*c*][1,2]oxaborinine-3,3(4*H*)-dicarboxylate (37)

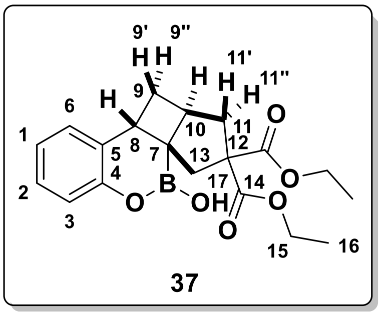

To an oven-dried microwave vial was added **S37** (35.8 mg, 0.1 mmol, 1 equiv.) and $Ir(dFppy)_3$ (0.7 mg, 1 mol%) and the vial was sealed and purged with nitrogen. Subsequently, degassed, anhydrous toluene (4 mL) was added via syringe and the reaction mixture was irradiated for 2 h. Upon completion, volatiles were removed under reduced pressure and the crude mixture was dissolved in *n*-hexanes and filtered through a PTFE syringe filter. Concentration of the filtrated under reduced pressure yielded **37** as a light brown oil (quantitative NMR yield, 31.5 mg, 88%).

^{1}H NMR (400 MHz, Acetone-d_6) δ = 7.76 (s, 1H, H17), 7.09 (ddd, *J* = 8.1, 5.8, 3.3 Hz, 1H, H2), 6.95 – 6.82 (m, 3H, H6+H1+H3), 4.24 (dqd, *J* = 22.6, 7.1, 2.9 Hz, 4H, H15), 3.26 (t, *J* = 8.6 Hz, 1H, H8), 2.93 (tdd, *J* = 8.4, 5.5, 3.6 Hz, 1H, H10), 2.71 – 2.56 (m, 2H, H11’+$H13_{back}$), 2.50 – 2.40 (m, 2H, H11”+$H13_{front}$), 2.24 (ddd, *J* = 12.5, 9.5, 3.1 Hz, 1H, H9’), 1.98 (ddd, *J* = 12.2, 8.6, 7.8 Hz, 1H, H9”), 1.26 (dt, *J* = 15.2, 7.1 Hz, 6H, H16) ppm.

^{13}C NMR (100 MHz, Acetone-d_6) δ = 173.9 (C14), 172.3 (C14), 152.7 (C4), 130.3 (C5), 129.1 (C6), 128.2 (C2), 123.4 (C1), 119.4 (C3), 66.2 (C12), 62.5 (C15), 62.0 (C15), 44.4 (C13), 42.9 (C10), 42.2 (C11), 40.4 (C8), 34.5 (C9), 14.4 (C16), 14.3 (C16) ppm.

^{11}B NMR (128 MHz, Acetone-d_6) δ = 32.92 ppm.

HMRS (ESI) calc. for $C_{19}H_{23}BO_6Na^+$ $[M+Na]^+$ 381.1480, found 381.1472.

IR (ATR): ῦ = 3443, 2979, 2936, 1727, 1591, 1489, 1430, 1368, 1345, 1245, 1196, 1141, 1095, 1071, 1016, 859, 755, 695, 659 cm^{-1}.

9-Fluoro-3-tosyl-1,1a,2,3,4,10b-hexahydro-5*H*-benzo[5',6'][1,2]oxaborinino[3',4':1,4]cyclobuta[1,2-*c*]pyrrol-5-ol (38)

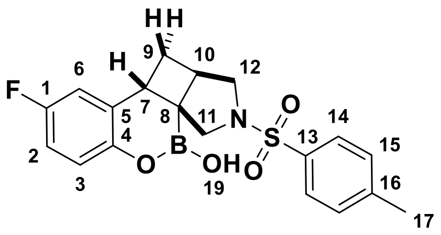

38

To an oven-dried microwave vial was added **S38** (38.7 mg, 0.1 mmol, 1 equiv.) and Ir(dFppy)$_3$ (0.7 mg, 1 mol%) and the vial was sealed and purged with nitrogen. Subsequently, degassed, anhydrous toluene (4 mL) was added via syringe and the reaction mixture was irradiated for 2 h. Upon completion, volatiles were removed under reduced pressure and the crude mixture was dissolved in *n*-hexanes and filtered through a PTFE syringe filter. Concentration of the filtrated under reduced pressure yielded **38** as a light brown oil (quantitative NMR yield, 35.2 mg, 91%).

^{1}H NMR (400 MHz, $CDCl_3$) δ = 7.72 (d, *J* = 8.3 Hz, 2H, H15), 7.34 (d, *J* = 7.9 Hz, 2H, H14), 6.80 (qd, *J* = 8.9, 5.6 Hz, 2H, H2+H3), 6.62 (dd, *J* = 8.7, 2.9 Hz, 1H, H6), 4.74 (s, 1H, H19), 3.59 (d, *J* = 9.9 Hz, 1H, H11), 3.51 (d, *J* = 9.7 Hz, 1H, H12), 3.41 (dd, *J* = 10.1, 7.2 Hz, 1H, H7), 2.96 (ddd, *J* = 9.6, 6.5, 3.7 Hz, 1H, H10), 2.82 (d, *J* = 9.9 Hz, 1H, H11), 2.75 (dd, *J* = 9.8, 6.7 Hz, 1H, H12), 2.49 – 2.34 (m, 4H, H9+H17), 2.13 (ddd, *J* = 12.3, 8.9, 7.1 Hz, 1H, H9) ppm.
^{13}C NMR (100 MHz, CDCl3) δ = 158.5 (d, *J* = 241.6 Hz, C1), 146.8 (d, *J* = 2.4 Hz, C4), 143.9 (C16), 132.2 (C13), 130.3 (d, *J* = 7.2 Hz, C5), 129.8 (C14), 128.2 (C15), 119.7 (d, *J* = 8.3 Hz, C3), 114.7 (d, *J* = 22.7 Hz, C6), 114.3 (d, *J* = 23.1 Hz, C2), 56.4 (C11), 54.7 (C12), 40.9 (C10), 37.0 (C7), 34.6 (C9), 21.7 (C17) ppm.
^{11}B NMR (128 MHz, $CDCl_3$) δ = 31.69 ppm.
^{19}F NMR (376 MHz, $CDCl_3$) δ = -120.15 (td, *J* = 8.2, 5.0 Hz) ppm.
HMRS (ESI) calc. for $C_{19}H_{19}BFNO_4SNa^+$ $[M+Na]^+$ 410.1004, found 410.1004.
IR (ATR): ῦ = 3435, 2970, 2927, 2854, 1598, 1493, 1413, 1377, 1339, 1308, 1285, 1259, 1238, 1213, 1180, 1161, 1147, 1118, 1091, 1057, 1012, 981, 867, 811, 735, 708, 662 cm^{-1}.

7,9-Dimethyl-3-tosyl-1,1a,2,3,4,10b-hexahydro-5*H*-benzo[5',6'][1,2]oxaborinino[3',4':1,4]cyclobuta[1,2-*c*]pyrrol-5-ol (39)

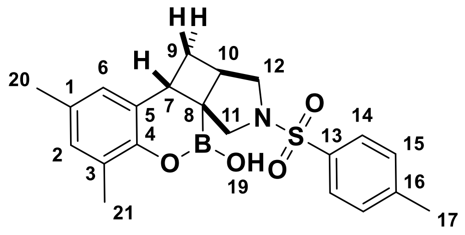

39

To an oven-dried microwave vial was added **S41** (39.6 mg, 0.1 mmol, 1 equiv.) and Ir(dFppy)$_3$ (0.7 mg, 1 mol%) and the vial was sealed and purged with nitrogen. Subsequently, degassed, anhydrous toluene (4 mL) was added via syringe and the reaction mixture was irradiated for 4 h. Upon completion, volatiles were removed under reduced pressure and the crude mixture was dissolved in *n*-hexanes and filtered through a PTFE syringe filter. Concentration of the filtrated under reduced pressure yielded **39** as an off-white solid (quantitative NMR yield, 36.5 mg, 92%).

^{1}H NMR (400 MHz, Acetone) δ = 7.87 (s, 1H, H19), 7.76 (d, *J* = 8.3 Hz, 2H, H14), 7.49 (d, *J* = 8.0 Hz, 2H, H15), 6.80 (s, 1H, H), 6.63 (s, 1H), 3.62 (d, *J* = 9.9 Hz, 1H, H11), 3.47 (d, *J* = 9.8 Hz, 1H, H12), 3.29 (dd, *J* = 10.0, 7.4 Hz, 1H, H7), 2.99 – 2.93 (m, 1H, H10), 2.88 (d, *J* = 9.9 Hz, 1H, H11), 2.77 (dd, *J* = 9.8, 6.8 Hz, 1H, H12), 2.47 (s, 3H, H17), 2.39 – 2.30 (m, 1H, H9), 2.19 (s, 3H, H20), 2.16 (s, 3H, H21) ppm.

^{13}C NMR (100 MHz, Acetone) δ = 148.2 (C4), 144.4 (C16), 133.7 (C13), 132.0 (C1), 130.5 (C15), 130.2 (C2), 129.1 (C3), 129.0 (C14), 127.3 (C5), 127.2 (C6), 57.5 (C11), 55.6 (C12), 41.6 (C10), 38.1 (C7), 35.5 (C9), 21.4 (C17), 20.6 (C20), 16.2 (C21) ppm.
^{11}B NMR (128 MHz, Acetone) δ = 31.32 ppm.
HRMS (ESI) calc. for $C_{21}H_{24}BNO_4SNa^+$ $[M+Na]^+$ 420.1411, found 420.1401.
IR (ATR): ῦ = 3503, 2960, 2855, 1718, 1592, 1474, 1459, 1433, 1362, 1327, 1301, 1271, 1235, 1216, 1167, 1113, 1016, 985, 932, 910, 853, 814, 751, 663 cm^{-1}.

Methyl 5-hydroxy-3-tosyl-1,1a,2,3,4,10b-hexahydro-5*H*-benzo[5',6'][1,2]oxaborinino[3',4':1,4]cyclobuta[1,2-*c*]pyrrole-8-carboxylate (40)

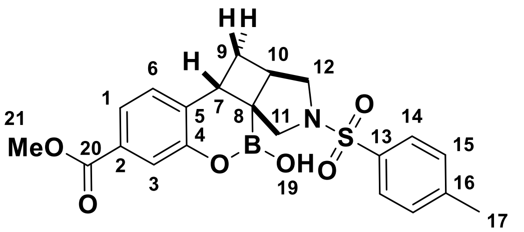

40

To an oven-dried microwave vial was added **S40** (42.7 mg, 0.1 mmol, 1 equiv.) and Ir(dFppy)$_3$ (0.7 mg, 1 mol%) and the vial was sealed and purged with nitrogen. Subsequently, degassed, anhydrous toluene (8 mL) was added via syringe and the reaction mixture was irradiated for 2 h. Upon completion, volatiles were removed under reduced pressure and the crude mixture was dissolved in Et$_2$O and filtered through a PTFE syringe filter. Concentration of the filtrated under reduced pressure yielded **40** as an off-white solid (quantitative NMR yield, 34.6 mg, 81%).

1H NMR (400 MHz, Acetone) δ = 8.06 (s, 1H, H19), 7.81 – 7.72 (m, 2H, H14), 7.59 (dd, J = 7.9, 1.7 Hz, 1H, H1), 7.47 (d, J = 8.0 Hz, 2H, H15), 7.43 (d, J = 1.7 Hz, 1H, H3), 7.15 (d, J = 8.0 Hz, 1H, H6), 3.85 (s, 3H, H21), 3.67 (d, J = 10.0 Hz, 1H, H11), 3.50 – 3.39 (m, 2H, H7+H12), 3.01 (td, J = 8.0, 3.7 Hz, 1H, H10), 2.88 (d, J = 10.0 Hz, 2H, H11), 2.75 (dd, J = 9.9, 6.7 Hz, 1H, H12), 2.46 (s, 3H, H17), 2.45 – 2.36 (m, 2H, H9), 2.12 (ddd, J = 12.9, 9.2, 7.5 Hz, 1H, H9) ppm.
^{13}C NMR (100 MHz, CDCl$_3$) δ = 166.8 (C20), 152.4 (C4), 144.5 (C16), 135.8 (C2), 133.5 (C13), 130.5 (C15), 129.6 (C6), 129.0 (C14), 124.5 (C1), 120.2 (C3), 57.5 (C11), 55.6 (C12), 52.3 (C21), 41.6 (C10), 37.9 (C7), 35.2 (C9), 21.4 (C17) ppm.
HRMS (ESI) calc. for $C_{21}H_{22}BNO_6SNa^+$ $[M+Na]^+$ 450.1153, found 450.1146.
IR (ATR): ῦ = 3737, 3678, 3568, 1714, 1509, 1437, 1339, 1286, 1217, 1161, 1093, 1012, 892, 813, 765, 709, 663 cm^{-1}.

Triethyl 8-fluoro-5-hydroxy-1,1a,2,10b-tetrahydro-5*H*-benzo[*e*]cyclopenta[1,4]cyclobuta[1,2-*c*][1,2]oxaborinine-3,3,7(4*H*)-tricarboxylate (41)

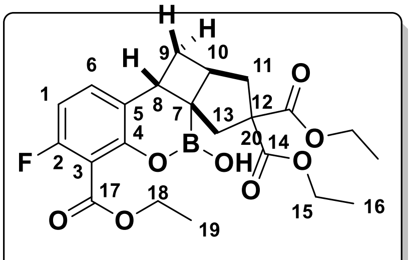

41

To an oven-dried microwave vial was added **S39** (44.8 mg, 0.1 mmol, 1 equiv.) and Ir(dFppy)$_3$ (0.7 mg, 1 mol%) and the vial was sealed and purged with nitrogen. Subsequently, degassed, anhydrous toluene (4 mL) was added via syringe and the reaction mixture was irradiated for 2 h. Upon completion, volatiles were removed under reduced pressure and the crude mixture was dissolved in *n*-hexanes and filtered through a PTFE syringe filter. Concentration of the filtrated under reduced pressure yielded **41** as a light brown oil (quantitative NMR yield, 37.7 mg, 84%).

^{1}H NMR (400 MHz, CDCl$_3$) δ = 7.61 (s, 1H, H20), 6.90 (dd, *J* = 8.6, 6.4 Hz, 1H, H6), 6.68 (t, *J* = 8.6 Hz, 1H, H1), 4.45 (qd, *J* = 7.2, 1.9 Hz, 2H, H18), 4.31 – 4.12 (m, 3H, H19), 3.29 (t, *J* = 8.7 Hz, 1H,

H8), 2.79 – 2.49 (m, 4H, C13, C11, C10), 2.19 – 2.03 (m, 2H, H9), 2.00 (d, J = 14.3 Hz, 1H, H13), 1.40 (t, J = 7.2 Hz, 3H, H18), 1.27 (t, J = 7.1 Hz, 6H, H19) ppm.
^{13}C NMR (101 MHz, $CDCl_3$) δ = 175.22 (C14), 170.83 (C14), 163.89 (C17), 158.70 (d, J = 248.6 Hz, C2), 150.00 (d, J = 7.0 Hz, C4), 129.81 (d, J = 9.8 Hz, C6), 124.84 (d, J = 3.7 Hz, C5), 114.04 (d, J = 19.9 Hz, C3), 109.56 (d, J = 21.3 Hz, C1), 66.34 (C12), 62.73 (C14), 62.01 (C18), 61.73 (C14), 44.88 (C13), 42.61 (C8), 42.09 (C11), 41.59 (C10), 32.38 (C9), 14.36 (C19), 14.21 (C15), 14.05 (C16) ppm.
^{11}B NMR (128 MHz, $CDCl_3$) δ = 33.83 ppm.
^{19}F NMR (376 MHz, $CDCl_3$) δ = -120.04 (q, J = 7.7 Hz) ppm.
HMRS (ESI) calc. for $C_{22}H_{27}BFO_8$ $[M+H]^+$ 449.1777, found 449.1769.
IR (ATR): ῡ = 3224, 2980, 1728, 1662, 1618, 1424, 1400, 1375, 1301, 1248, 1186, 1151, 1095, 1067, 1035, 1019, 860, 811, 734, 682, 646 cm^{-1}.

Ethyl 8-fluoro-5-hydroxy-3-tosyl-1,1a,2,3,4,10b-hexahydro-5*H*-benzo[5',6'][1,2]oxaborinino[3',4':1,4]cyclobuta[1,2-*c*]pyrrole-7-carboxylate (42)

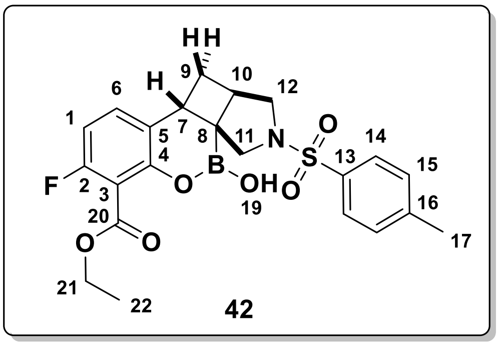

To an oven-dried microwave vial was added **S42** (45.9 mg, 0.1 mmol, 1 equiv.) and Ir(dFppy)$_3$ (0.7 mg, 1 mol%) and the vial was sealed and purged with nitrogen. Subsequently, degassed, anhydrous DMF (4 mL) was added via syringe and the reaction mixture was irradiated for 2 h. Upon completion, volatiles were removed under reduced pressure and Et_2O (2 mL) was added to the crude mixture and the solid was filter and washed with Et_2O (2 × 2 mL). Concentration of the filtrated under reduced pressure yielded **42** as an off-white solid (quantitative NMR yield, 34.9 mg, 76%).

^{1}H NMR (400 MHz, $CDCl_3$) δ = 7.71 (d, J = 8.1 Hz, 2H, H14), 7.33 (d, J = 8.0 Hz, 2H, H15), 6.98 (dd, J = 8.6, 6.2 Hz, 1H, H6), 6.74 (t, J = 8.7 Hz, 1H, H1), 4.38 (q, J = 7.1 Hz, 2H), 3.58 (d, J = 9.9 Hz, 1H), 3.51 (d, J = 9.8 Hz, 1H), 3.44 (dd, J = 10.0, 7.3 Hz, 1H), 2.99 – 2.88 (m, 1H), 2.78 (d, J = 9.8 Hz, 1H), 2.70 (dd, J = 9.9, 6.7 Hz, 1H), 2.43 (s, 4H), 2.09 (ddd, J = 12.5, 9.0, 7.3 Hz, 1H) ppm.
^{13}C NMR (100 MHz, $CDCl_3$) δ 164.5 (C20), 159.0 (d, J = 250.7 Hz, C2), 149.4 (d, J = 6.7 Hz, C4), 143.9 (C16), 132.08 (C13), 131.4 (d, J = 9.8 Hz, C6), 129.8 (C15), 128.2 (C14), 125.4 (C5), 112.7 (d, J = 18.4 Hz, C3), 110.6 (d, J = 21.7 Hz, C1), 62.1 (C21), 56.4 (C11), 54.7 (C12), 41.0 (C10), 36.2 (C7), 34.6 (C9), 21.7 (C17), 14.3 (C22) ppm.
^{11}B NMR (128 MHz, $CDCl_3$) δ 32.10 ppm.
^{19}F NMR (376 MHz, $CDCl_3$) δ = -112.06 – -121.12 (m) ppm.
HRMS (ESI) calc. for $C_{22}H_{23}BFNO_6SNa^+$ $[M+Na]^+$ 482.1215, found 482.1215.
IR (ATR): ῡ = 2979, 1710, 1664, 1507, 1473, 1424, 1341, 1304, 1255, 1223, 1184, 1161, 1093, 1021, 1005, 860, 811, 709, 663 cm^{-1}.

1,2,3,3a,4,4a-Hexahydro-5*H*-benzo[*e*]cyclopenta[2,3]cyclobuta[1,2-*c*][1,2]oxaborinin-5-ol (43)

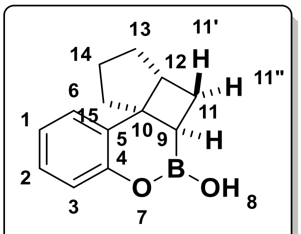

43

To an oven-dried microwave vial was added **S43** (10.7 mg, 0.05 mmol, 1 equiv.) and Ir(dF-ppy)$_3$ (0.3 mg, 1 mol%) and the vial was sealed and purged with nitrogen. Subsequently, degassed, anhydrous toluene (4 mL) was added via syringe and the reaction mixture was irradiated for 16 h. Upon completion, volatiles were removed under reduced pressure and preparative HPLC (C18, MeCN/water) yielded **43** as a colourless oil (65% NMR yield, 4.3 mg, 40%).

^{1}H NMR (400 MHz, $CDCl_3$) δ = 7.11 (ddt, *J* = 8.9, 3.4, 1.8 Hz, 2H), 7.03 – 6.96 (m, 1H), 6.91 (dd, *J* = 8.4, 1.4 Hz, 1H), 4.29 (s, 1H, H8), 2.65 – 2.53 (m, 1H, H12), 2.38 – 2.27 (m, 1H, H11), 2.22 (dq, *J* = 12.4, 6.2 Hz, 1H, H15), 2.11 – 2.01 (m, 1H, H15), 1.97 – 1.73 (m, 5H, H11+H13+H14+H9), 1.65 (dd, *J* = 12.9, 6.5 Hz, 1H, H13) ppm.
^{13}C NMR (100 MHz, $CDCl_3$) δ = 152.0 (C4), 132.2 (C5), 127.3 (C_{Ar}), 127.1 (C_{Ar}), 123.3 (C1), 118.8 (C3), 50.0 (C12), 47.7 (C10), 42.8 (C14), 34.4 (C13), 26.0 (C15), 24.7 (C11) ppm.
^{11}B NMR (128 MHz, $CDCl_3$) δ = 32.37 ppm.
HMRS (ESI) calc. for $C_{13}H_{16}BO_2^+$ $[M+H]^+$ 215.1238, found 215.1222.
IR (ATR): ῦ = 3227, 2946, 2867, 2412, 1739, 1487, 1371, 1331, 1299, 1229, 1217, 1095, 1071, 1009, 972, 933, 751, 650 cm^{-1}

Assignments of Diastereomers

Diastereomers were assigned by NOESY spectroscopy. Important Noe contacts are highlighted below.

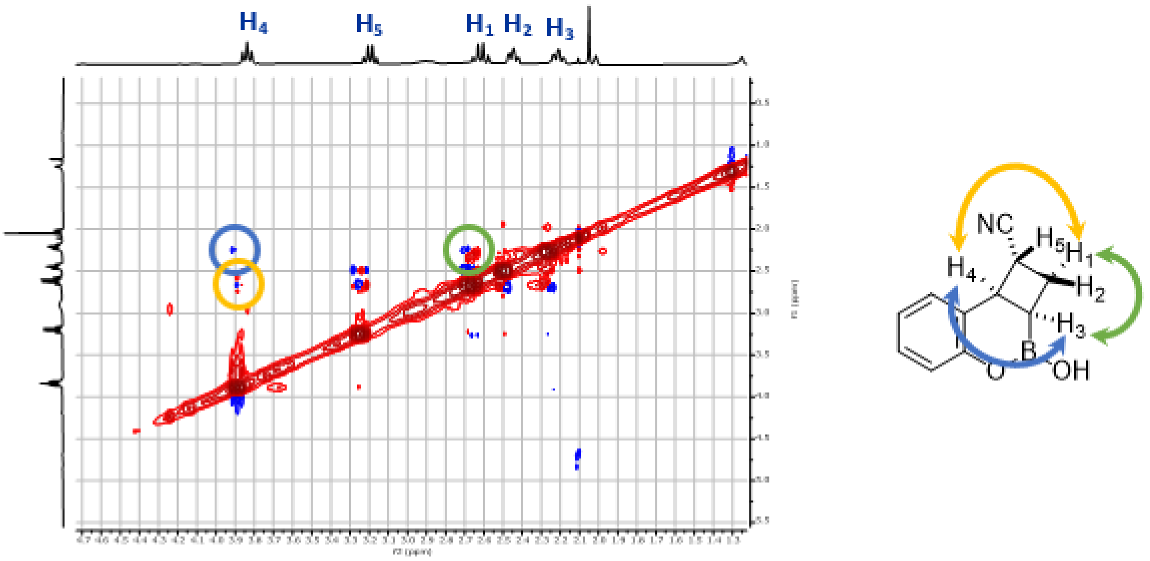

Figure S5: NOESY spectrum with highlighted NOE contacts of 2.

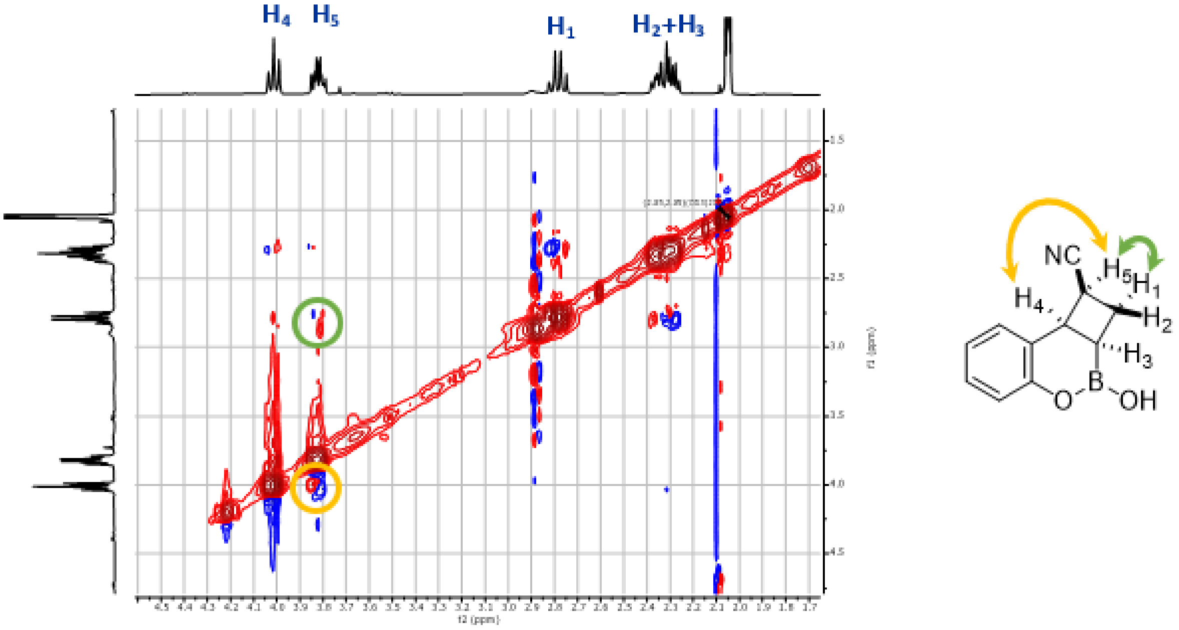

Figure S6: NOESY spectrum with highlighted NOE contacts of 2-syn.

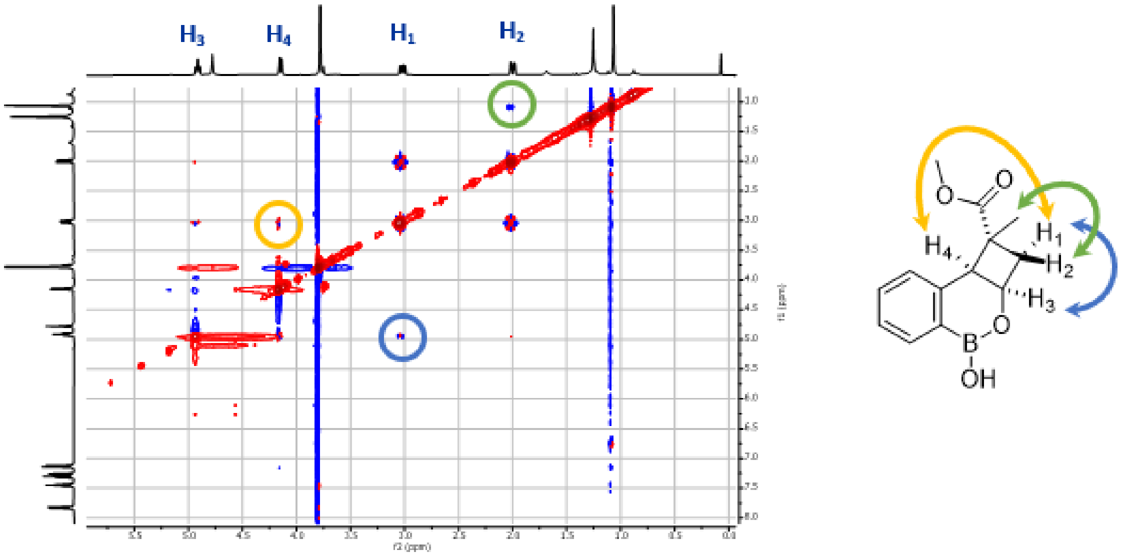

Figure S7: NOESY spectrum with highlighted NOE contacts of 20.

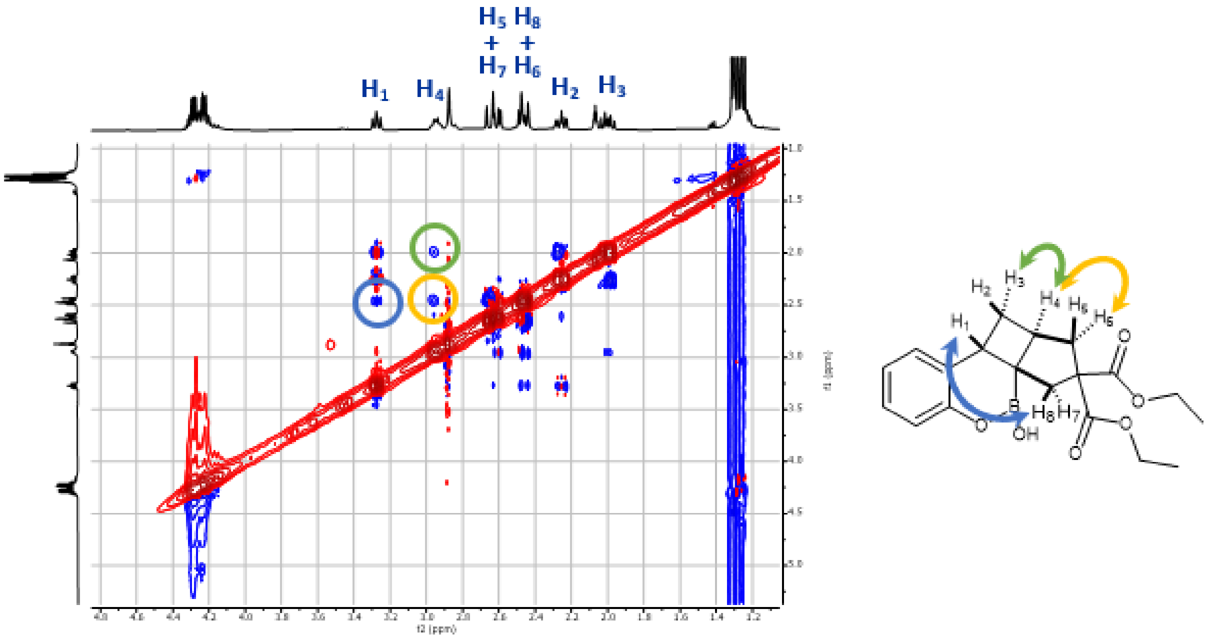

Figure S8: NOESY spectrum with highlighted NOE contacts of .

Study on Effect of Decreased Equivalents of Alkenes

Table S10: [2+2]-cycloadditions of benzoxaborines and 5 equivalents of alkene.

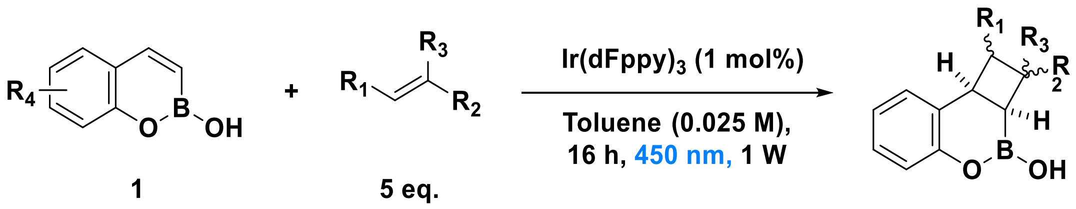

R_1	R_4	Yield[a] (%)	Dimer[a] (%)
CO_2Me	-	76	19
CN	5-F	77	13
CN	6-Me	79	9
CN	8-OMe	71	17

Table S11: [2+2]-cycloadditions of **S21** and 5 equivalents of alkene.

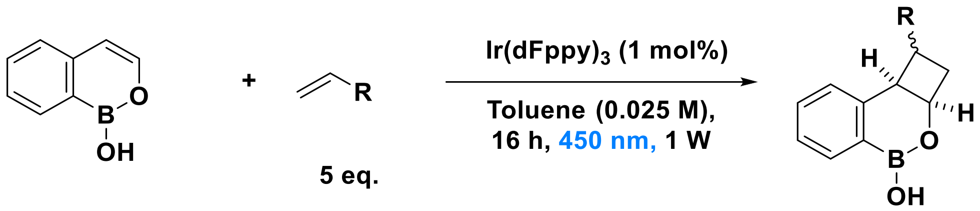

R	Yield[a] (%)	SM[a] (%)
CO_2Me	55	33

Unsuccessful Substrates

Table S12: Electron rich alkenes with benzoxaborine.

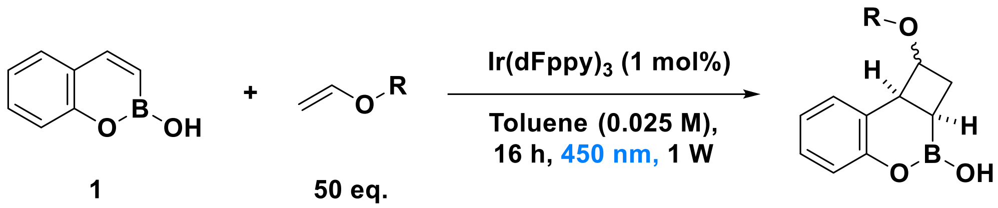

R	Yield[a] (%)	SM[a] (%)
COMe	81	0
Et	85	0

[a]Determined by ^{1}H NMR spectroscopy against a known internal standard (1,3,5-trimethoxybenzene).

Comment: Analysis of the crude reaction mixtures corresponding to the entries in Table S12 reveals clear evidence for the successful formation of the target products. However, due to their inherent instability, isolation of the products was not possible.

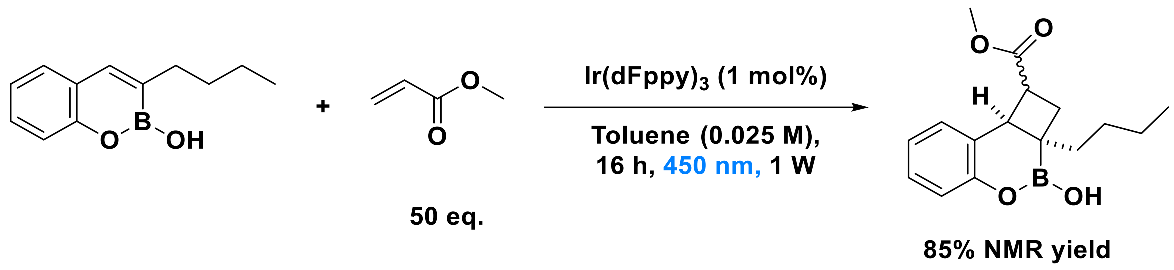

Comment: Attempts of isolation showed signs of degradation.

Table S13: Electron neutral and poor alkenes with benzoxaborine.

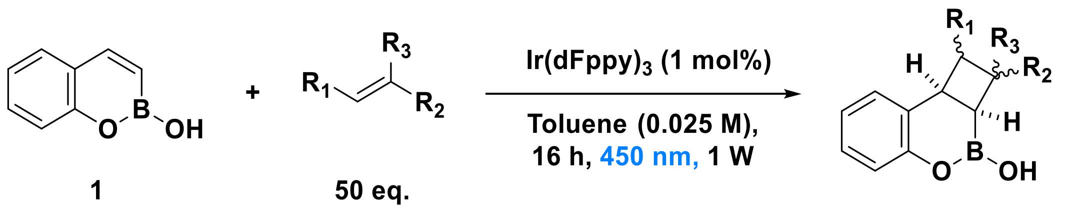

R_1	R_2	R_3	Yield[a] (%)	SM[a] (%)
Ph	H	H	0	99
p-Me-Ph	H	H	0	99
SO_2Ph	H	H	0	98
BPin	H	H	0	40
CO_2Me	CO_2Me	H	0	97
COMe	Me	Me	20[b]	19
$CONMe_2$	H	H	0	98

[a]Determined by ^{1}H NMR spectroscopy against a known internal standard (1,3,5-trimethoxybenzene).
[b]Reaction was not regioselective

Table S14: Electron neutral and poor alkenes with benzoxaborinine.

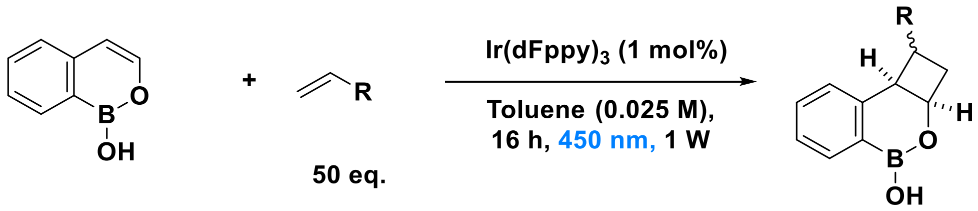

R	Yield[a] (%)	SM[a] (%)
Ph	0	78
SO_2Ph	11	69
BPin	0	51
COMe	22	52

[a]Determined by ^{1}H NMR spectroscopy against a known internal standard (1,3,5-trimethoxybenzene).

Table S15: BCBs with benzoxaborinine.

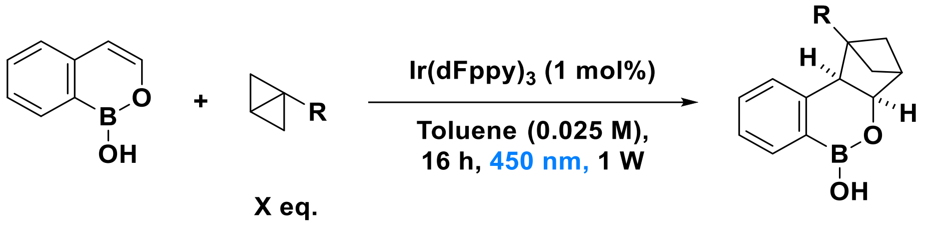

R	X	Yield[a] (%)	SM[a] (%)
Weinreb-amide	1	n.d.	20
Weinreb-amide	5	n.d.	32
COC_4H_9	5	n.d.	33

[a]Determined by ^{1}H NMR spectroscopy against a known internal standard (1,3,5-trimethoxybenzene).

Table S15: Electron neutral and poor alkenes with benzazaborine.

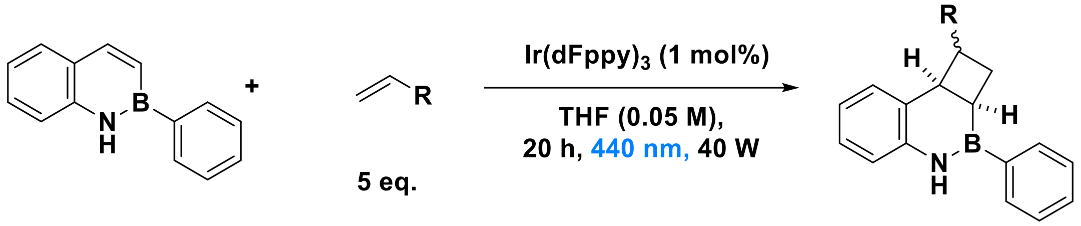

R	Yield[a] (%)	SM[a] (%)
Ph	0	88%
Weinreb-amide	55%	39%

[a]Determined by ^{1}H NMR spectroscopy against a known internal standard (1,3,5-trimethoxybenzene).

Benzazaborine with BCB

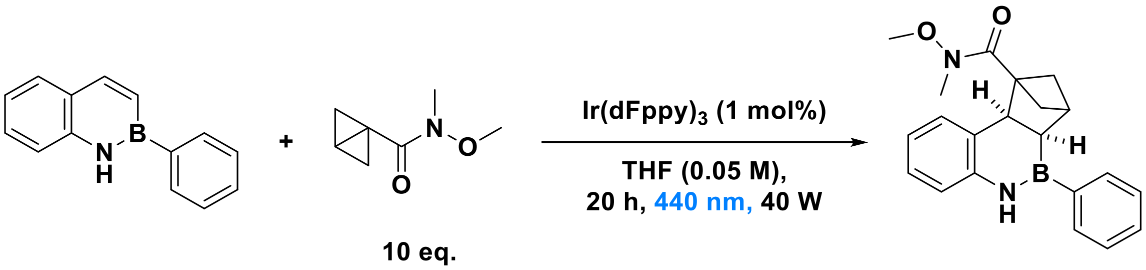

n.d. (92% starting material)

Electron rich benzazaborine

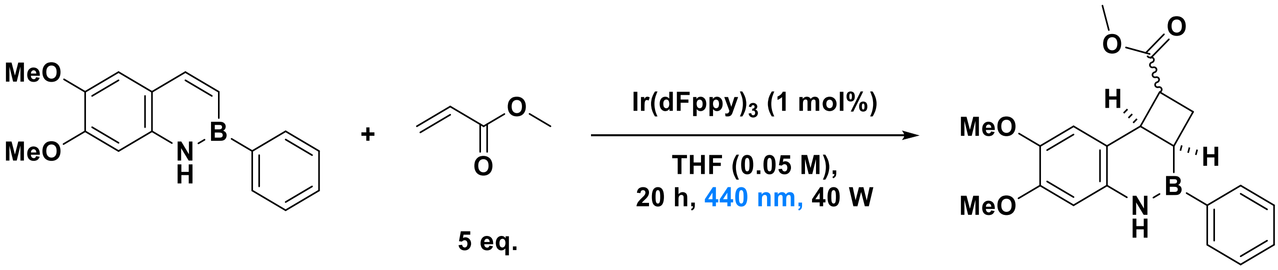

n.d. (50 % starting material)

Reaction Probes and Studies to Support EnT

Dimerisation

Structure **3** was identified as the predominant side product of the [2+2] cycloaddition involving benzoxaborines, and as the principal product formed in the absence of an alkene.

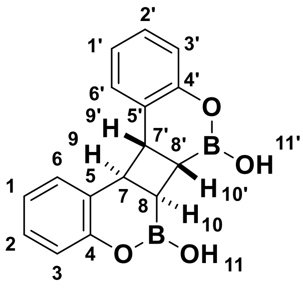

3

According to general procedure **H**, **1** (29.2 mg, 0.2 mmol, 2 equiv.) was converted to **3**, yielding an off-white solid (21.5 mg, 0.037 mg, 37%, of a maximum of 50%) after purification by flash column chromatography (C18, 20→100% MeCN/water).

^{1}H NMR (400 MHz, $CDCl_3$ with a drop of D_2O) δ =7.18 (td, *J* = 7.7, 1.8 Hz, 2H, H2), 7.02 (d, *J* = 8.1 Hz, 2H, H3), 6.96 (td, *J* = 7.4, 1.2 Hz, 2H, H1), 6.85 (dd, *J* = 7.5, 1.7 Hz, 2H, H6), 6.61 (s, 2H, H11), 3.56 (d, *J* = 8.6 Hz, 2H, H9), 2.40 (d, *J* = 8.9 Hz, 2H, H10) ppm.
^{13}C NMR (100 MHz, $CDCl_3$ with a drop of D_2O) δ = 152.2 (C4), 128.3 (C2), 128.0 (C6), 127.8 (C5), 123.3 (C1+C1'), 119.3 (C3+C3'), 44.9 (C7+C7') ppm.
^{11}B NMR (128 MHz, $CDCl_3$ with a drop of D_2O) δ = 34.20 ppm.
HMRS (ESI) calc. for $C_{16}H_{14}B_2O_4Na^+$ $[M+Na]^+$ 315.0970, found 315.0922.
IR (ATR): ṽ = 3210, 2911, 1700, 1601, 1582, 1486, 1456, 1394, 1351, 1301, 1273, 1230, 1197, 1105, 1037, 937, 884, 848, 803, 751, 655, 609 cm^{-1}.

Comment: The sole formation of the dimer in the presence of the photocatalyst supports an EnT mechanism, as no additional reactants are required. This is in line with reports of similar systems using coumarins.[23]

UV/Vis Analysis

All absorption spectra were recorded on a Shimadzu UV-1900 I UV/Vis Spectrophotometer, at medium speed with 0.5 nm steps in the 200-600 nm range using a 1 cm path quartz cuvette. All concentrations are based on the concentration of model reaction conditions.

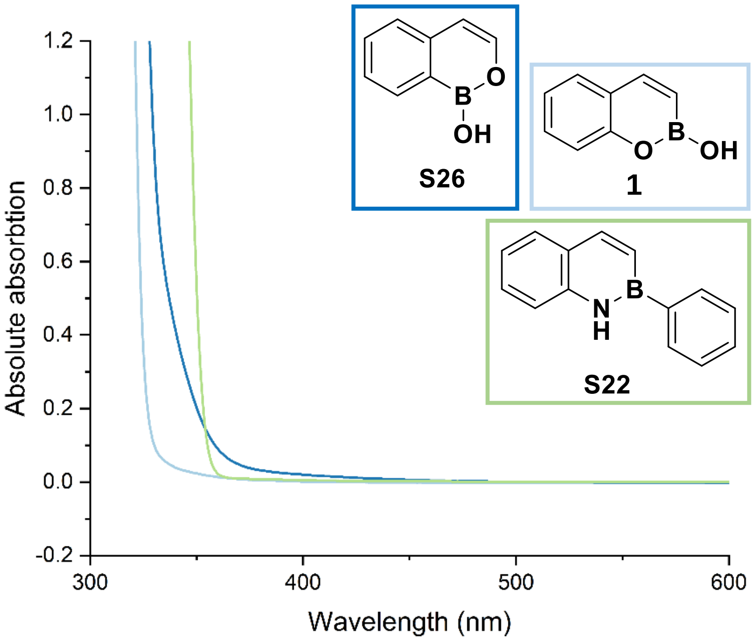

*Figure S9: Absorption spectrum of **1** in Toluene (0.025 M), **S26** in Toluene (0.025 M), and **S22** in THF (0.05 M).*

Comment: As illustrated in Figure S9, none of the model starting materials exhibit absorbance at the wavelengths corresponding to the emission spectrum of the blue LED light employed in this study. This observation effectively rules out the possibility of direct photoexcitation, thereby supporting the involvement of a catalytic activation pathway.

Triplet Energy Calculations

All calculations were performed by using the density functional theory (DFT) with the Gaussian 16, Revision A.03 program package.[24] For the calculation of geometry optimisations, unrestricted B3LYP-D3[25, 26] functional with 6-31G(d,p) basis set were used. Electronic energies of optimised structure were further calculated by single point calculation of uB3LYP with 6-311+G(d,p) basis set. Solvation energy corrections (E_{sol}) were carried out at the same level of single point calculations using polarisable continuum model (PCM)[27] in toluene solvent (ε = 2.3741). Frequency calculations were conducted for all stationary points to verify the presence of a local minima. Thermodynamic parameters, including Gibbs free energies, were calculated at 298 K.
Final solution phase Gibbs free energies (G_{sol}) were computed as described below:

$$G_{sol} = E_{sol} + (G - E)$$

The triplet energy of the compound was calculated using the Gibbs free energies determined for each molecule as described below:

$$E_T = G_{sol}(\text{Triplet}) - G_{sol}(\text{Singlet})$$

Table S16: Energy component of all optimised geometries

	**E_{sol} (Hartree)**	**G – E (Hartree)**	**G_{sol} (Hartree)**	**E_T (kcal/mol)**
^{3}[**1**]	-484.523808	0.097804	-484.426004	60.72
^{1}[**1**]	-484.626960	0.104199	-484.522760	
^{3}[**S21**]	-484.521984	0.097655	-484.424329	59.90
^{1}[**S21**]	-484.624456	0.104671	-484.519785	
^{3}[**S17**]	-620.471204	0.180267	-620.290937	63.19
^{1}[**S17**]	-620.578934	0.187287	-620.391647	

Mulliken spin densities of optimised triplet state structures

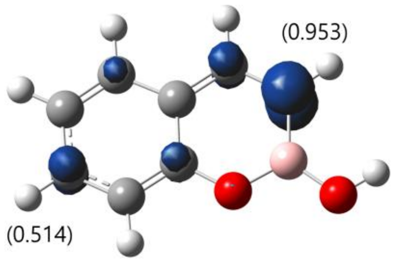

1

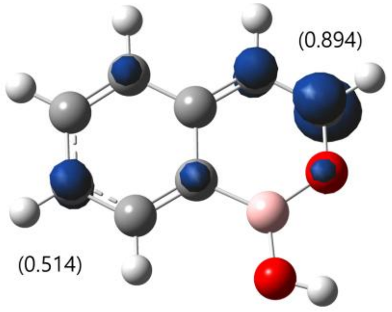

S26

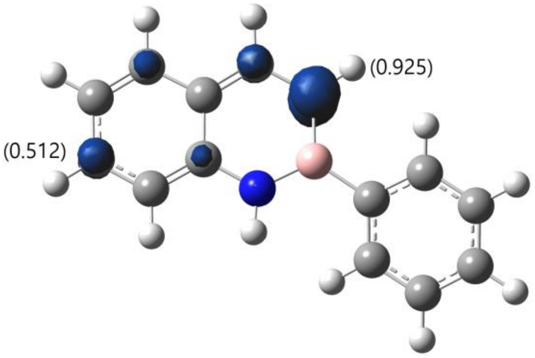

S22

Comment: Triplet energy calculations for the starting materials revealed values within the range accessible by the photocatalyst Ir(dFppy)$_3$, supporting the feasibility of triplet energy transfer. Additionally, the computed spin density distribution showed localisation predominantly at the 3-position, consistent with the observed regioselectivity and suggesting preferential initial radical addition at this site during the reaction.

Cartesian coordinates of all optimised geometries

^{3}[**1**]

C	2.61454600	-1.08784200	0.00006400
C	1.26637000	-1.51082900	0.00007900
C	0.24110800	-0.59417800	-0.00000600
C	0.51568500	0.84753600	-0.00008500
C	1.91859600	1.23130400	-0.00012800
C	2.92148200	0.29510800	-0.00004700
H	3.41022200	-1.82564100	0.00013600
H	1.01248500	-2.56616000	0.00014500
C	-0.50824100	1.76390600	-0.00006400
H	2.14847700	2.29341000	-0.00022500
H	3.96024900	0.61368600	-0.00007200
C	-1.90850700	1.29773600	0.00003200
H	-0.27970700	2.82620200	-0.00010000
H	-2.69689400	2.04617900	0.00009400
O	-1.04237500	-1.07358800	-0.00005300
B	-2.14324100	-0.21851400	0.00000300
O	-3.34729100	-0.86511900	0.00014100
H	-4.08752400	-0.24189300	0.00023200

^{1}[**1**]

C	2.62644500	-1.04528700	0.00007500
C	1.31266300	-1.50286400	0.00003400
C	0.26137300	-0.58184300	-0.00000600
C	0.51369300	0.80942700	-0.00000100
C	1.85391900	1.24217100	0.00004200
C	2.90139200	0.33061800	0.00007900
H	3.44288800	-1.76200600	0.00010700
H	1.07597300	-2.56199500	0.00002900
C	-0.60594000	1.72992500	-0.00002600
H	2.05656500	2.31065500	0.00004600
H	3.92933100	0.68083000	0.00011200
C	-1.88986800	1.29076500	-0.00004700
H	-0.36512100	2.79327400	-0.00001500
H	-2.69587700	2.02301700	-0.00004700
O	-1.00895400	-1.06547200	-0.00005700
B	-2.12072700	-0.23190000	-0.00006900
O	-3.31827400	-0.89480900	-0.00003400
H	-4.06436500	-0.27949900	-0.00005900

^{3}[**S26**]

C	2.41738900	1.14688500	-0.01225800
C	1.04031500	1.46670800	-0.01513300
C	0.06933100	0.48402900	-0.00005800
C	0.47357100	-0.92252300	0.01055800
C	1.89207400	-1.22500000	0.02353200
C	2.82087800	-0.21178800	0.00790300
H	3.16264500	1.93615300	-0.01957400
H	0.73671500	2.51086100	-0.02588300
C	-0.49527800	-1.89430100	-0.01909800
H	2.20575500	-2.26591300	0.03866200
H	3.88103700	-0.45265100	0.01396400
C	-1.94178900	-1.58321600	-0.06745300
H	-0.22824600	-2.94782000	-0.02005600
H	-2.69128100	-2.29993800	0.25084600
O	-2.36748700	-0.28086900	0.00302800
B	-1.45327800	0.77117600	0.00370800
O	-1.94987100	2.03831300	0.01764000
H	-2.92030700	2.03910100	0.01020000

^{1}[**S26**]

C	2.43284600	1.10608300	0.00001100
C	1.08758900	1.45993500	-0.00000400
C	0.08250100	0.47799700	-0.00000300
C	0.45599600	-0.89268000	0.00001400
C	1.82046100	-1.24126000	0.00002800
C	2.79468800	-0.25037100	0.00002700
H	3.20280900	1.87278700	0.00000900
H	0.79979200	2.50810800	-0.00001700
C	-0.59188300	-1.89821300	0.00001400
H	2.10689100	-2.29038400	0.00004100
H	3.84559300	-0.52855400	0.00003800
C	-1.89470900	-1.55646100	-0.00000100
H	-0.32868600	-2.95146300	0.00002700
H	-2.71022100	-2.27176900	-0.00000100
O	-2.33186700	-0.26573700	-0.00001900
B	-1.42541500	0.79276700	-0.00002600
O	-1.92103900	2.06532800	-0.00003300
H	-2.89078400	2.07053500	-0.00006500

^{3}[**S22**]

C	4.07260800	-1.45383300	-0.17228900
C	2.65825500	-1.61157700	-0.19526000
C	1.81200500	-0.53250900	-0.07106200
C	2.36707200	0.81747500	0.08672000
C	3.81673700	0.92947400	0.10741700
C	4.63392700	-0.17368800	-0.01917700

H	4.70882300	-2.32728500	-0.27366400
H	2.23572400	-2.60714700	-0.31385500
C	1.54690700	1.91571300	0.20227900
H	4.24090300	1.92314900	0.22430500
H	5.71374700	-0.05411900	-0.00143300
C	0.07709400	1.77659000	0.18325500
H	1.99729000	2.89973100	0.31017800
H	-0.50584900	2.68227200	0.32948800
N	0.42070100	-0.68648800	-0.09712700
H	0.11495700	-1.63920000	-0.25563900
B	-0.53303600	0.38016700	0.02563100
C	-2.06482700	0.07723200	0.01548700
C	-2.99566600	1.08420700	-0.31430300
C	-2.58171500	-1.19698100	0.33021800
C	-4.36676200	0.83286400	-0.34332600
H	-2.63553500	2.07882700	-0.56590000
C	-3.95126000	-1.45656200	0.31299500
H	-1.90328000	-1.99834800	0.61708600
C	-4.84899300	-0.44069600	-0.02835900
H	-5.06017500	1.62677300	-0.60899100
H	-4.32118100	-2.44656700	0.56756300
H	-5.91745300	-0.63975500	-0.04498300

[1][**S22**]

C	-4.04564900	-1.44629300	0.21005200
C	-2.67090300	-1.62175900	0.23364100
C	-1.81042600	-0.51760500	0.07872100
C	-2.35615500	0.78370600	-0.09756300
C	-3.76021800	0.92660500	-0.11816700
C	-4.59884200	-0.16541300	0.03228900
H	-4.69781900	-2.30710400	0.32957200
H	-2.24382800	-2.61292100	0.37026000
C	-1.47032100	1.91179100	-0.24579000
H	-4.17694400	1.92186200	-0.25481700
H	-5.67687300	-0.03542200	0.01406100
C	-0.11313900	1.78495700	-0.22408000
H	-1.94519600	2.88453600	-0.38097500
H	0.48487500	2.68562500	-0.35434300
N	-0.43601300	-0.66901000	0.09678500
H	-0.11969100	-1.62028500	0.25373600
B	0.51077200	0.39506500	-0.04848400
C	2.04621900	0.08106300	-0.02532600
C	2.97199900	1.06238000	0.37887200
C	2.56366800	-1.17420000	-0.40124500
C	4.34232400	0.80293500	0.42151300
H	2.61048800	2.04419500	0.67604600
C	3.93234400	-1.44228200	-0.37001600
H	1.88778000	-1.95457500	-0.74778300
C	4.82674700	-0.45258700	0.04606800
H	5.03272000	1.57786400	0.74528600
H	4.30286900	-2.41841900	-0.67313000
H	5.89396000	-0.65740100	0.07319800

Stern–Volmer Quenching Study

For the Stern–Volmer quenching experiments six stock solutions were prepared of **2**, **S26**, **ACN** (acrylonitrile) and Ir(dFppy)$_3$ in toluene (160 µM) and **S22** and Ir(dFppy)$_3$ in THF. All solvents were sparged for 30 min prior to making the solutions. To make the desired solutions 1 mL of Ir(dFppy)$_3$ solution was transferred to a quartz vial, equipped with a septum, via syringe and diluted with the desired amount of starting material solution and degassed solvent to 2 mL. In order to determine the initial emission intensity (I_0) a solution of Ir(dFppy)$_3$ (80 µM) was prepared, irradiated at 450 nm and emission intensity measured over the range of 460 nm to 700 nm.
The solutions were irradiated at 450 nm and the photoluminescence intensity (I) was measured over the range of 460 nm to 700 nm. The emission intensity at 469 nm was compared to I_0 at the same wavelength.

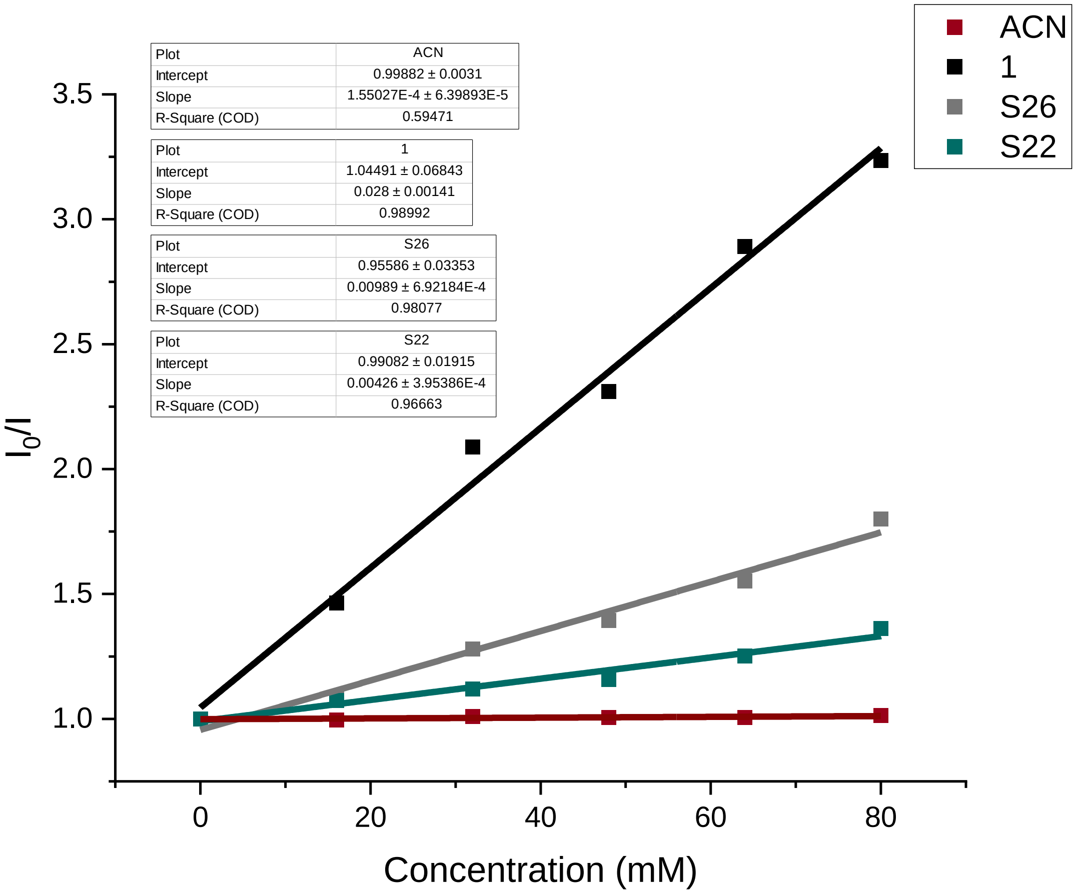

*Figure S10: Stern–Volmer quenching experiment of Ir(dFppy)$_3$ by **ACN**, **1**, **S26** and **S22**.*

Proposed Mechanism

Based on the performed experiments and literature review, we proposed the following, EnT based, mechanism:

The reaction starts by excitation of the photocatalyst upon irradiation with blue light. Subsequently, energy transfer occurs and benzoxaborine **1** is excited to the triplet state **1***. In this state an attack to the terminal position of the olefin of acrylonitrile takes place to generate 1,4-diradical intermediate **I***. Finally, the product **2** formation occurs through intersystem crossing to the open shell singlet and radical-radical recombination.[28]

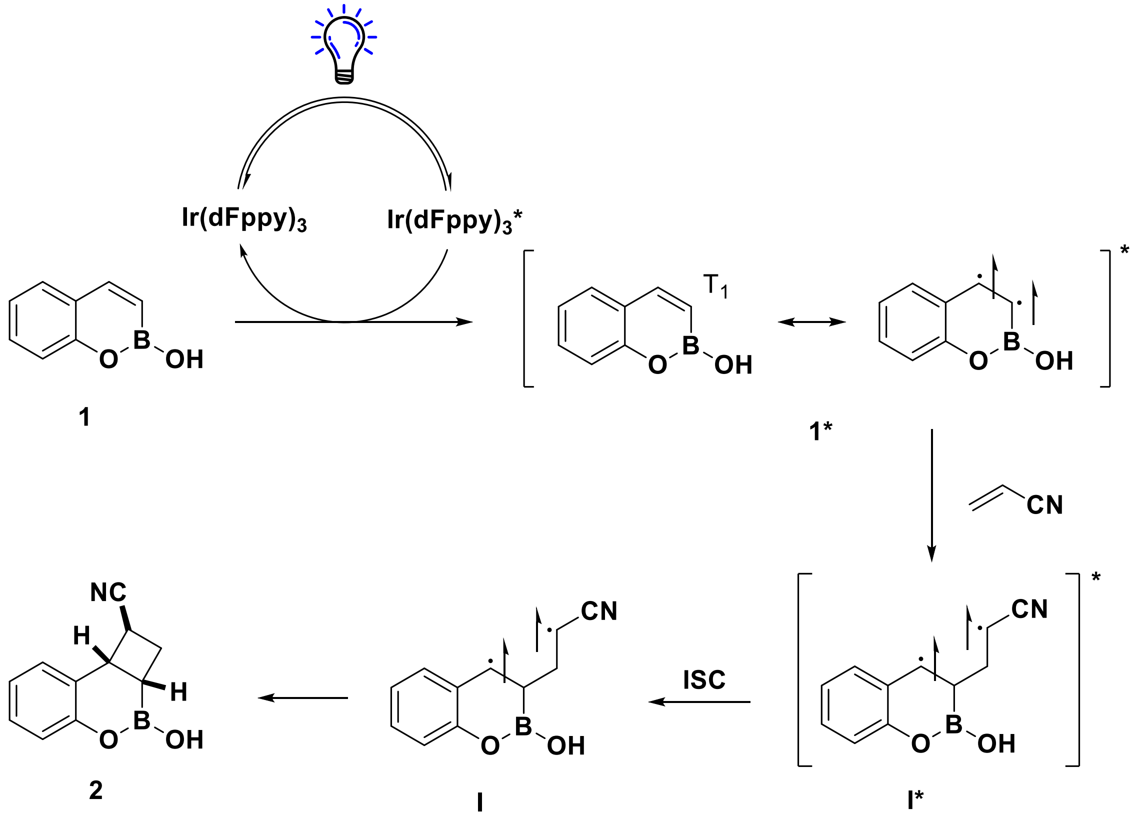

Figure S11: Proposed mechanism of [2+2]-Cycloadditon of Benzoxaborines

Derivatisation and Scale-Up

Derivatisation

General Procedure J: Intramolecular Chan–Lam

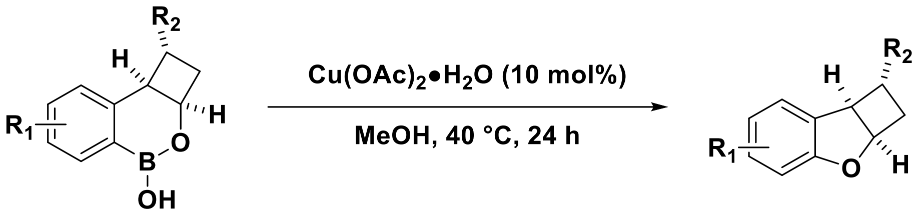

According to adapted literature procedure by Sheppard *et al.*,[29] the corresponding photoproduct was added to a round-bottom-flask. Subsequently, copper (II) acetate monohydrate (10 mol%) and MeOH (1 mL) were added and the reaction mixture was stirred for 24 h at 40 °C under air. Upon completion, the reaction mixture was cooled and the crude was directly purified by flash column chromatography (SiO_2, 0→5% EtOAc/*n*-hexane).

Methyl-1-methyl-1,2,2a,7b-tetrahydrocyclobuta[*b*]benzofuran-1-carboxylate (31)

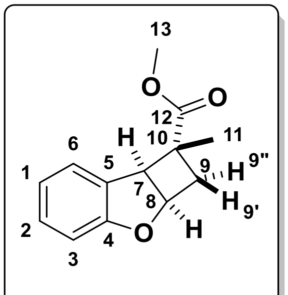

31

Prepared according to General Procedure **J**, **24** (16.1 mg, 0.065 mmol, 1 equiv.) was converted to **31** yielding a colourless oil (7.4 mg, 0.034 mmol, 52%) after flash-column chromatography (SiO_2, 0→5% Acetone/*n*-hexane).

Rf (10% EtOAc/*n*-hexane) = 0.42.
^{1}H NMR (400 MHz, $CDCl_3$) δ = 7.22 – 7.09 (m, 2H), 6.96 – 6.83 (m, 2H), 5.25 (td, *J* = 7.0, 4.8 Hz, 1H, H8), 4.27 (dd, *J* = 7.2, 3.4 Hz, 1H, H7), 3.79 (s, 3H, H13), 2.93 (ddd, *J* = 13.6, 6.8, 3.5 Hz, 1H, H9”), 2.11 (ddd, *J* = 13.6, 4.8, 1.3 Hz, 1H, H9’), 1.17 (s, 3H, H11) ppm.
^{13}C NMR (100 MHz, $CDCl_3$) δ = 177.7 (C12), 161.3 (C4), 129.2 (C2), 127.2 (C6), 126.3 (C5), 120.8 (C1), 110.9 (C3), 77.5 (C8), 52.5 (C13), 51.6 (C7), 45.9 (C10), 40.9 (C9), 20.7 (C11) ppm.
HMRS (ESI) calc. for $C_{13}H_{14}O_3^-$ [M-H]$^-$ 218.0948, found 218.0976.
IR (ATR): ῦ = 2940, 1727, 1608, 1592, 1476, 1460, 1434, 1322, 1301, 1286, 1232, 1193, 1134, 1105, 1075, 1019, 991, 925, 853, 821, 790, 748, 682 cm^{-1}.

Methyl-7-fluoro-1,2,2a,7b-tetrahydrocyclobuta[*b*]benzofuran-1-carboxylate (32)

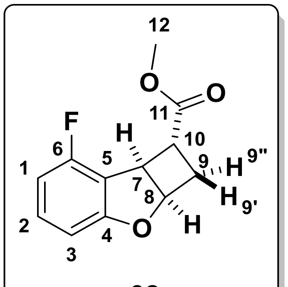

32

Prepared according to General Procedure **J**, **27** (10.7 mg, 0.043 mmol, 1 equiv.) was converted to **32** yielding a colourless oil (7.7 mg, 0.035 mmol, 81%) after flash-column chromatography (SiO_2, 0→5% Acetone/*n*-hexane).

Rf (20% EtOAc/*n*-hexane) = 0.67.
^{1}H NMR (400 MHz, $CDCl_3$) δ = 7.15 (td, *J* = 8.2, 5.8 Hz, 1H, H2), 6.69 – 6.54 (m, 2H, H1, H3), 5.41 (td, *J* = 6.9, 4.6 Hz, 1H, H8), 4.36 – 4.27 (m, 1H, H7), 3.78 (s, 3H, H12), 3.25 (ddd, *J* = 10.0, 4.7, 3.0 Hz, 1H, H10), 2.82 – 2.48 (m, 2H, H9) ppm.

^{13}**C NMR** (100 MHz, $CDCl_3$) δ = 174.8 (C11), 163.2 (d, *J* = 8.0 Hz, C4), 160.1 (d, *J* = 248.4 Hz, C6), 130.8 (d, *J* = 8.6 Hz, C2), 116.0 (d, *J* = 21.5 Hz, C5), 108.2 (d, *J* = 20.3 Hz, C1), 106.7 (d, *J* = 3.5 Hz, C3), 81.2 (C8), 52.3 (C12), 44.8 (C7), 42.5 (C10), 33.4 (C9) ppm.
^{19}**F NMR** (376 MHz, $CDCl_3$) δ = -116.86 (dd, *J* = 8.6, 5.9 Hz) ppm.
HMRS (ESI) calc. for $C_{12}H_{11}FO_3Na^+$ $[M+H]^+$ 245.0584, found 245.0567.
IR (ATR): 1729, 1606, 1592, 1477, 1461, 1433, 1374, 1352, 1334, 1321, 1230, 1215, 1204, 1108, 1093, 1018, 989, 933, 904, 848, 805, 778, 752, 705, 658, 606 cm^{-1}.

1,2,2a,7b-Tetrahydrocyclobuta[*b*]benzofuran-1-yl acetate (33)

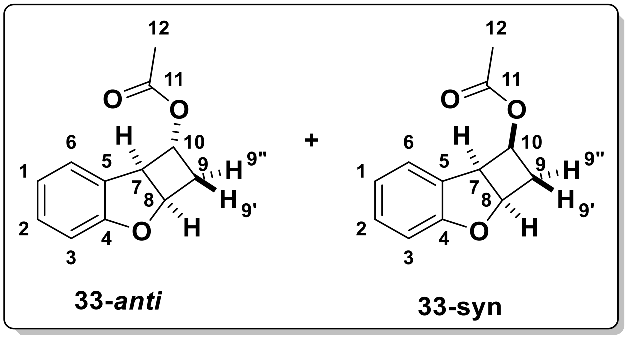

Prepared according to General Procedure **J**, **26** (16 mg, 69 µmol, 1 equiv.) was converted to yielding **33-*anti*** (5.6 mg, 28 µmol, 40%) and **33-*syn*** (4.7 mg, 23 µmol, 33%) as colourless oils after flash-column chromatography (SiO_2, 0→3% EtOAc/*n*-hexane).

Anti
$\mathbf{R}_f$ (20% EtOAc/*n*-hexane = 0.78
^{1}**H NMR** (400 MHz, $CDCl_3$) δ = 7.35 (d, *J* = 7.4 Hz, 1H, H6), 7.19 (t, *J* = 7.6 Hz, 1H, H2), 6.92 (ddd, *J* = 8.5, 7.0, 1.1 Hz, 1H, H1), 6.86 (d, *J* = 8.1 Hz, 1H, H3), 5.35 (ddd, *J* = 8.0, 6.5, 4.1 Hz, 1H, H10), 4.92 (ddd, *J* = 7.4, 3.5, 2.0 Hz, 1H, H8), 4.00 (t, *J* = 5.5 Hz, 1H, H7), 2.76 (dddd, *J* = 14.9, 7.2, 4.1, 1.5 Hz, 1H, H9'), 2.69 – 2.54 (m, 2H, H9"), 2.13 (s, 3H, H12) ppm.
^{13}**C NMR** (100 MHz, $CDCl_3$) δ = 170.6 (C11), 160.8 (C4), 129.3 (C2), 127.1 (C5), 125.9 (C6), 121.3 (C1), 110.5 (C3), 79.0 (C10), 75.1 (C8), 51.9 (C7), 37.6 (C9), 21.2 (C12) ppm.
HMRS (ESI) calc. for $C_{12}H_{12}O_3Na^+$ $[M+Na]^+$ 227.0679, found 227.0684.
IR (ATR): $\tilde{\upsilon}$ = 2946, 1737, 1609, 1592, 1476, 1461, 1372, 1335, 1319, 1236, 1222, 1171, 1157, 1097, 1052, 1015, 979, 903, 857, 830, 749, 639 cm^{-1}.

Syn
$\mathbf{R}_f$ (20% EtOAc/*n*-hexane = 0.88
^{1}**H NMR** (400 MHz, $CDCl_3$) δ = 7.20 (t, *J* = 7.7 Hz, 1H, H2), 7.08 (d, *J* = 7.7 Hz, 1H, H6), 6.89 (ddd, *J* = 8.2, 5.9, 1.2 Hz, 2H, H1, H3), 5.09 (q, *J* = 7.5 Hz, 1H, H10), 4.97 (tdd, *J* = 6.1, 4.8, 0.9 Hz, 1H, H8), 4.52 (q, *J* = 6.0 Hz, 1H, H7), 2.93 (dddd, *J* = 14.2, 8.2, 6.2, 3.9 Hz, 1H, H9"), 2.44 (dddd, *J* = 13.9, 7.5, 4.8, 1.4 Hz, 1H, H9'), 1.98 (s, 3H, H12) ppm.
^{13}**C NM**R (100 MHz, $CDCl_3$) δ = 170.8 (C11), 161.0 (C4), 129.2 (C2), 127.0 (C6), 125.6 (C5), 120.9 (C3), 111.1 (C1), 74.8 (C8), 67.0 (C10), 51.4 (C7), 37.3 (C9), 20.9 (C12) ppm.
HMRS (ESI) calc. for $C_{12}H_{12}O_3Na^+$ $[M+Na]^+$ 227.0679, found 227.0687
IR (ATR): $\tilde{\upsilon}$ = 2957, 2923, 2851, 1730, 1688, 1592, 1477, 1461, 1431, 1374, 1236, 1213, 1183, 1154, 1108, 1093, 1018, 989, 933, 904, 848, 805, 752, 657, 604 cm^{-1}.

N,N-Dimethyl-1,2,2a,7b-tetrahydrocyclobuta[*b*]benzofuran-1-carboxamide (34)

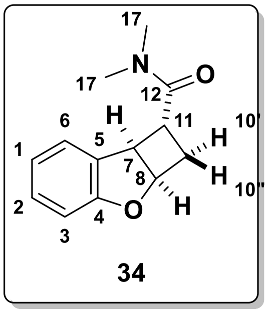

Prepared according to General Procedure **J**, **29** (22.6 mg, 0.092 mmol, 1 equiv.) was converted to **34** yielding a colourless oil (11.5 mg, 0.053 mmol, 57%) after flash-column chromatography (SiO_2, 0→50% EtOAc/*n*-hexane).

$\mathbf{R_f}$ (50% Acetone) = 0.42
^{1}H NMR (400 MHz, $CDCl_3$) δ = 7.17 (ddd, *J* = 9.1, 7.4, 2.7 Hz, 2H, H2+H6), 6.97 – 6.69 (m, 2H, H1+H3), 5.28 (td, *J* = 6.9, 4.1 Hz, 1H, H8), 4.27 (dt, *J* = 6.9, 3.2 Hz, 1H, H7), 3.35 (ddd, *J* = 9.3, 5.3, 3.6 Hz, 1H, H11), 3.01 (s, 3H, H17), 2.96 (s, 3H, H17), 2.78 (dddd, *J* = 13.3, 6.7, 5.3, 2.8 Hz, 1H, H9”), 2.58 (dddd, *J* = 13.8, 9.9, 4.0, 1.3 Hz, 1H, H9’).
^{13}C NMR (100 MHz, $CDCl_3$) δ = 173.3 (C12), 161.3 (C4), 130.2 (C5), 129.0 (C6), 125.0 (C2), 121.0 (C1), 110.7 (C3), 79.8 (C8), 46.5 (C7), 42.8 (C11), 37.0 (C17), 35.8 (C17), 33.0 (C10) ppm.
HRMS (ESI) calc. for $C_{13}H_{15}NO_2Na^+$ $[M+Na]^+$ 240.0995, found 240.0971.
IR (ATR): ῦ = 2936, 1744, 1638, 1592, 1474, 1460, 1397, 1322, 1263, 1235, 1216, 1146, 1094, 1048, 1016, 986, 906, 848, 815, 751, 699, 665 cm^{-1}.

Morpholino(1,2,2a,7b-tetrahydrocyclobuta[*b*]benzofuran-1-yl)methanone (35)

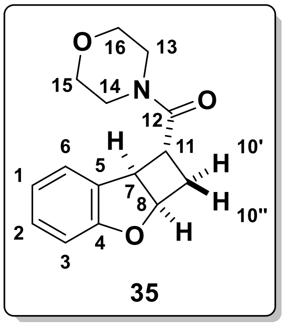

Prepared according to General Procedure **J**, **30** (25.1 mg, 0.087 mmol, 1 equiv.) was converted to **35** yielding a colourless oil (13.7 mg, 0.053 mmol, 60%) after flash-column chromatography (SiO_2, 0→100% EtOAc/*n*-hexane).

$\mathbf{R_f}$ (50% EtOAc/*n*-hexane) = 0.22
^{1}H NMR (400 MHz, $CDCl_3$) δ 7.23 – 7.07 (m, 2H, H6+H2), 7.01 – 6.77 (m, 2H, H3+H1), 5.28 (td, *J* = 6.9, 3.9 Hz, 1H, H8), 4.31 (dt, *J* = 6.8, 3.2 Hz, 1H, H7), 3.68 (dqd, *J* = 9.7, 7.2, 4.4 Hz, 6H, $H_{Morpholine}$), 3.57 – 3.22 (m, 3H, $H_{Morpholine}$+H11), 2.79 (dddd, *J* = 13.7, 6.9, 5.4, 2.7 Hz, 1H, H10”), 2.59 (dddd, *J* = 13.7, 10.0, 3.9, 1.4 Hz, 1H, H10’) ppm.
^{13}C NMR (101 MHz, $CDCl_3$) δ = 171.9 (C12), 161.3 (C4), 129.9 (C5), 129.1 (C2), 124.9 (C6), 121.1 (C1), 110.7 (C3), 79.7 (C8), 67.0 (C15), 66.7 (C16), 46.3 (C7), 45.1 ($C_{Morpholine}$), 42.5 ($C_{Morpholine}$), 42.4 (C11), 33.0 (C10) ppm.
HRMS (ESI) calc. for $C_{15}H_{17}NO_3Na^+$ $[M+Na]^+$ 282.1100, found 282.1084.
IR (ATR): ῦ = 3480, 3206, 2855, 1760, 1704, 1598, 1483, 1420, 1337, 1220, 1184, 1157, 1091, 1038, 1009, 857, 813, 771, 708, 663 cm^{-1}.

2-(Dimethylamino)ethyl 1-methyl-1,2,2a,7b-tetrahydrocyclobuta[*b*]benzofuran-1-carboxylate (36)

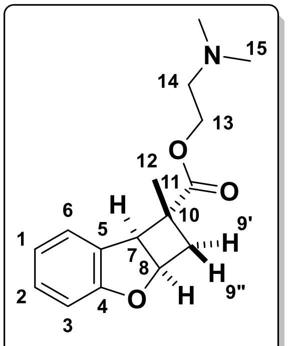

36

Prepared according to General Procedure **J**, **25** (18.4 mg, 0.061 mmol, 1 equiv.) was converted to **36** yielding a colourless oil (13 mg, 0.047 mmol, 78%) after flash-column chromatography (SiO_2, 0→100% Acetone/*n*-hexane).

R_f (100% Acetone) = 0.33

^{1}H NMR (400 MHz, $CDCl_3$) δ = 7.19 (t, *J* = 8.0 Hz, 1H, H2), 7.15 (d, *J* = 7.4 Hz, 1H, H6), 6.90 (td, *J* = 7.4, 0.9 Hz, 1H, H1), 6.86 (d, *J* = 8.0 Hz, 1H, H3), 5.24 (td, *J* = 6.9, 4.8 Hz, 1H, H8), 4.35 (t, *J* = 5.7 Hz, 2H, H13), 4.28 (dd, *J* = 7.1, 3.5 Hz, 1H, H7), 2.93 (ddd, *J* = 13.6, 6.8, 3.5 Hz, 2H, H9”), 2.76 (bs, 2H, H14), 2.41 (s, 6H, H15), 2.11 (ddd, *J* = 13.6, 4.8, 1.3 Hz, 1H, H9’), 1.17 (s, 3H, H12).

^{13}C NMR (100 MHz, $CDCl_3$) δ = 177.1 (C11), 161.4 (C4), 129.3 (C2), 127.3 (C6), 126.3 (C5), 120.9 (C1), 111.0 (C3), 77.4 (C8), 62.7 (C13), 57.6 (C14), 51.7 (C7), 46.0 (C15), 45.6 (C15), 40.9 (C9), 20.7 (C12) ppm.

HRMS (ESI) calc. for $C_{16}H_{22}NO_3^+$ $[M+H]^+$ 276.1594, found 276.1605.

IR (ATR): ῦ = 2970, 2939, 2822, 2772, 1724, 1608, 1592, 1476, 1460, 1375, 1299, 1285, 1232, 1136, 1105, 1075, 1019, 907, 856, 751 cm^{-1}.

Scale-Up Reactions

7-bromo-3-hydroxy-1,2a,3,8b-tetrahydro-2*H*-benzo[*e*]cyclobuta[*c*][1,2]oxaborinine-1-carbonitrile (10)

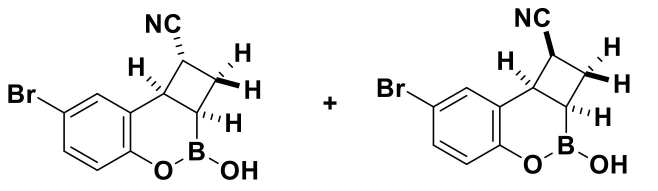

8 *8-syn*

Prepared according to General Procedure **H**, **S19** (224.8 mg, 1.0 mmol, 1 equiv.), acrylonitrile (3.25 mL, 10 mmol, 50 equiv.) and Ir(dFppy)$_3$ (7.0 mg, 1 mol%) were irradiated for 16 h with blue light (440 nm, 1 W). Upon completion, the mixture was concentrated under reduced pressure, the crude was resolved in DCM and filtered. The filtrate was concentrated under reduced pressure and purification by flash column chromatography (C18, 10→100% MeCN) yielded **10** (66.6 mg, 0.24 mmol, 24%, *d.r.* = >20:1) and **10-*syn*** (51.4 mg, 0.18 mmol, 18%, *d.r.* = >20:1) as off-white solids.

Syn

^{1}H NMR (400 MHz, Acetone-*d$_6$*) δ = 8.19 (s, 1H, H12), 7.35 (dd, *J* = 8.6, 2.2 Hz, 1H, H2), 7.29 (d, *J* = 2.2 Hz, 1H, H6), 6.90 (d, *J* = 8.6 Hz, 1H, H3), 4.05 (t, *J* = 9.4 Hz, 1H, H7), 3.89 – 3.77 (m, 1H, H10), 2.81 (q, *J* = 10.3 Hz, 1H, H9”), 2.33 (ddt, *J* = 27.9, 11.4, 6.3 Hz, 1H, H8, H9’) ppm.

Anti

^{1}H NMR (400 MHz, Acetone-*d$_6$*) δ = 8.28 (s, 1H, H12), 7.35 (dd, *J* = 8.6, 2.5 Hz, 1H, H2), 7.32 (d, *J* = 2.5 Hz, 1H, H6), 6.88 (d, *J* = 8.6 Hz, 1H, H3), 3.91 (t, *J* = 9.5 Hz, 1H, H7), 3.32 (dtd, *J* = 9.9, 8.7, 1.2 Hz, 1H, H10), 2.67 (q, *J* = 10.6 Hz, 1H, H9’’), 2.57 – 2.44 (m, 1H, H9’), 2.34 – 2.20 (m, 1H, H8) ppm.

Methyl-3-phenyl-1,2,2a,3,4,8b-hexahydrobenzo[e]cyclobuta[c][1,2]azaborinine-1-carboxylate (13)

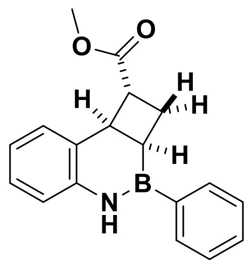

13

Prepared according to General Procedure **I**, **S22** (1.0 g, 4.9 mmol, 1 equiv.) and methyl acrylate (2.2 mL, 24.4 mmol, 5 equiv.) were converted to **13** yielding a pink solid (841 mg, 2.89 mmol, 59%, *d.r.* = >20:1) after purification by flash column chromatography (C18, 20→100% MeCN/water).

^{1}H NMR (400 MHz, $CDCl_3$) δ = 7.74 – 7.66 (m, 2H), 7.52 – 7.36 (m, 3H), 7.13 (td, *J* = 7.6, 1.6 Hz, 1H), 7.08 (dd, *J* = 7.4, 1.6 Hz, 1H), 6.92 (td, *J* = 7.4, 1.2 Hz, 1H), 6.80 (dd, *J* = 7.8, 1.2 Hz, 1H), 6.75 (s, 1H), 3.93 (t, *J* = 9.4 Hz, 1H), 3.70 (s, 3H), 3.23 (dtd, *J* = 9.9, 8.8, 1.2 Hz, 1H), 2.90 – 2.78 (m, 1H), 2.73 – 2.61 (m, 1H), 2.31 (ddt, *J* = 11.2, 8.0, 2.3 Hz, 1H).

Testing Medicinal Relevance

Binding Studies

The binding study was performed according to a procedure disclosed by Hall *et al.*[30] A buffer solution was prepared by dissolving potassium phosphate monobasic (0.1 M) in D_2O. The buffer solution was adjusted to pH = 7.4 by adding 4 M solution of NaOH in D_2O. Solution A was prepared by dissolving **2** (1.99 mg, 10 µmol) in the buffer solution (658 µL). To this was added DMSO-d_6 (120 µL) in order to dissolve **2** fully. Solution B (0.15 M) was prepared by dissolving D-(–)-Fructose (5.44 mg, 30 µmol) or peptide (14.45 mg, 30 µmol) in 200 µL of Solution A. In an NMR-tube was added 350 µL of Solution A and a ^{1}H NMR was measured. The stepwise addition of Solution B was continuously measured by ^{1}H NMR. The results are depicted below.

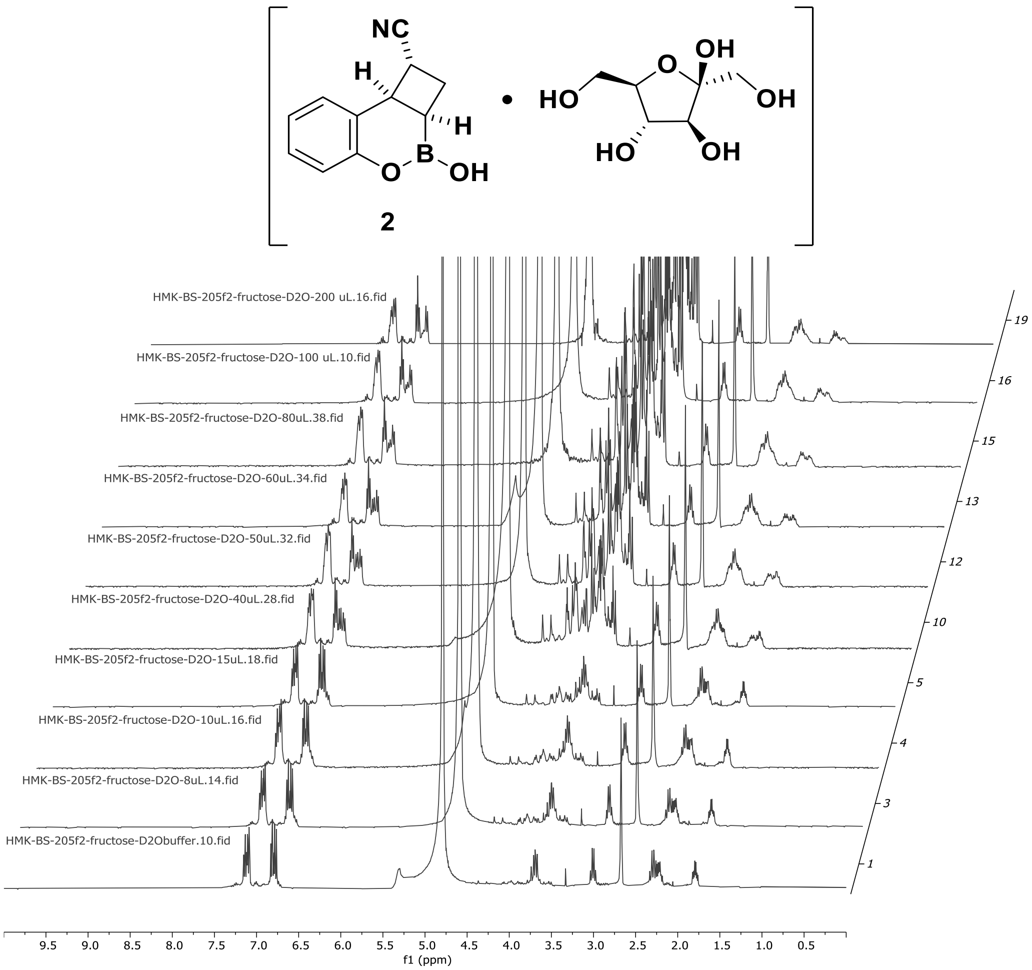

*Figure S12: Binding study of **2** with D-(–)-Fructose in 0.1 M phosphate buffer D_2O: DMSO-d_6 (85:15), pH = 7.4.*

Comment: With increasing amount of D-(–)-Fructose, the peaks at 2.34, 2.27 and 1.83 ppm are decreasing and new peaks are appearing at 2.40, 2.26 and 1.92 ppm, indicating the formation of a diol-boronate complex.

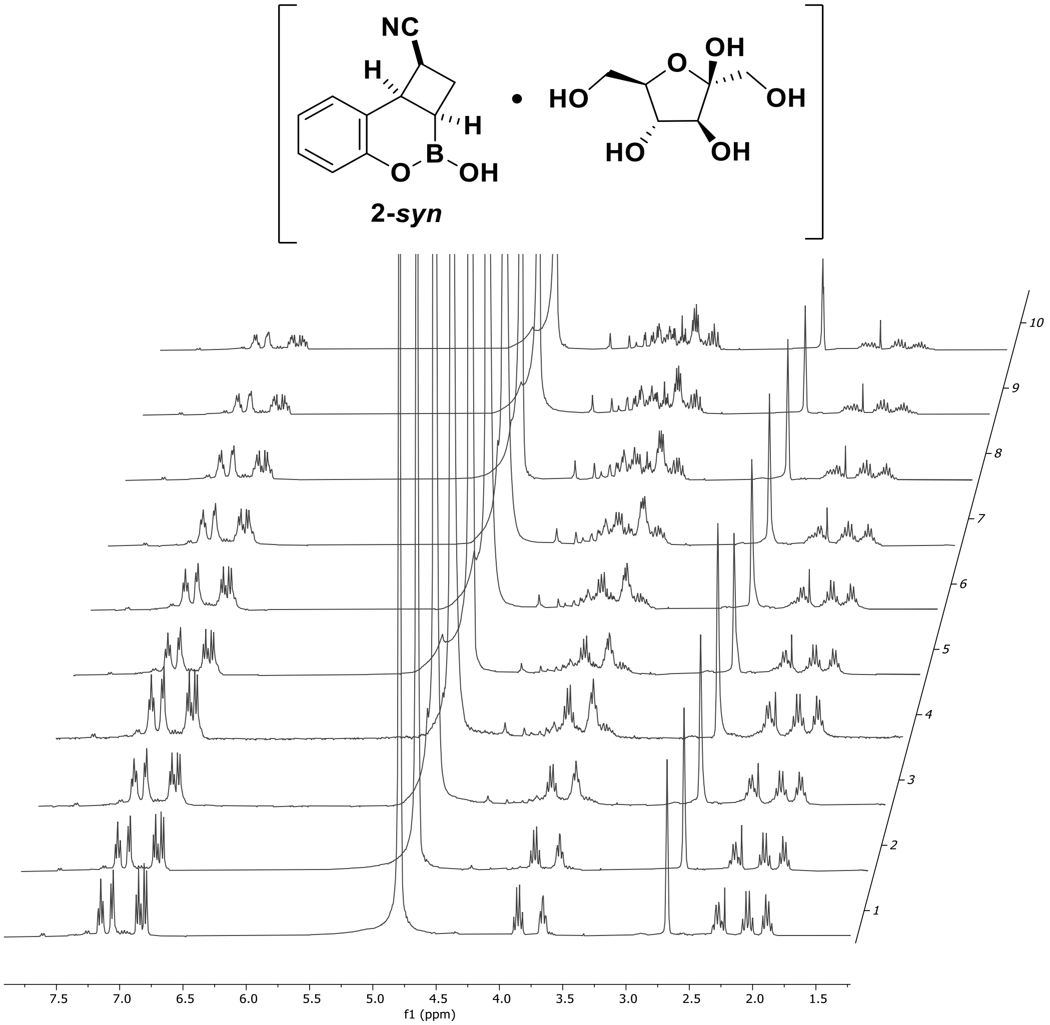

Figure S13: Binding study of **2-syn** *with D-(–)-Fructose in 0.1 M phosphate buffer D_2O: DMSO-d_6 (85:15), pH = 7.4.*

Comment: With increasing amount of D-(–)-Fructose, the peaks at 2.30, 2.06 and 1.90 ppm are decreasing and new peaks are appearing at 2.38, 2.12 and 1.96 ppm, indicating the formation of a diol-boronate complex.

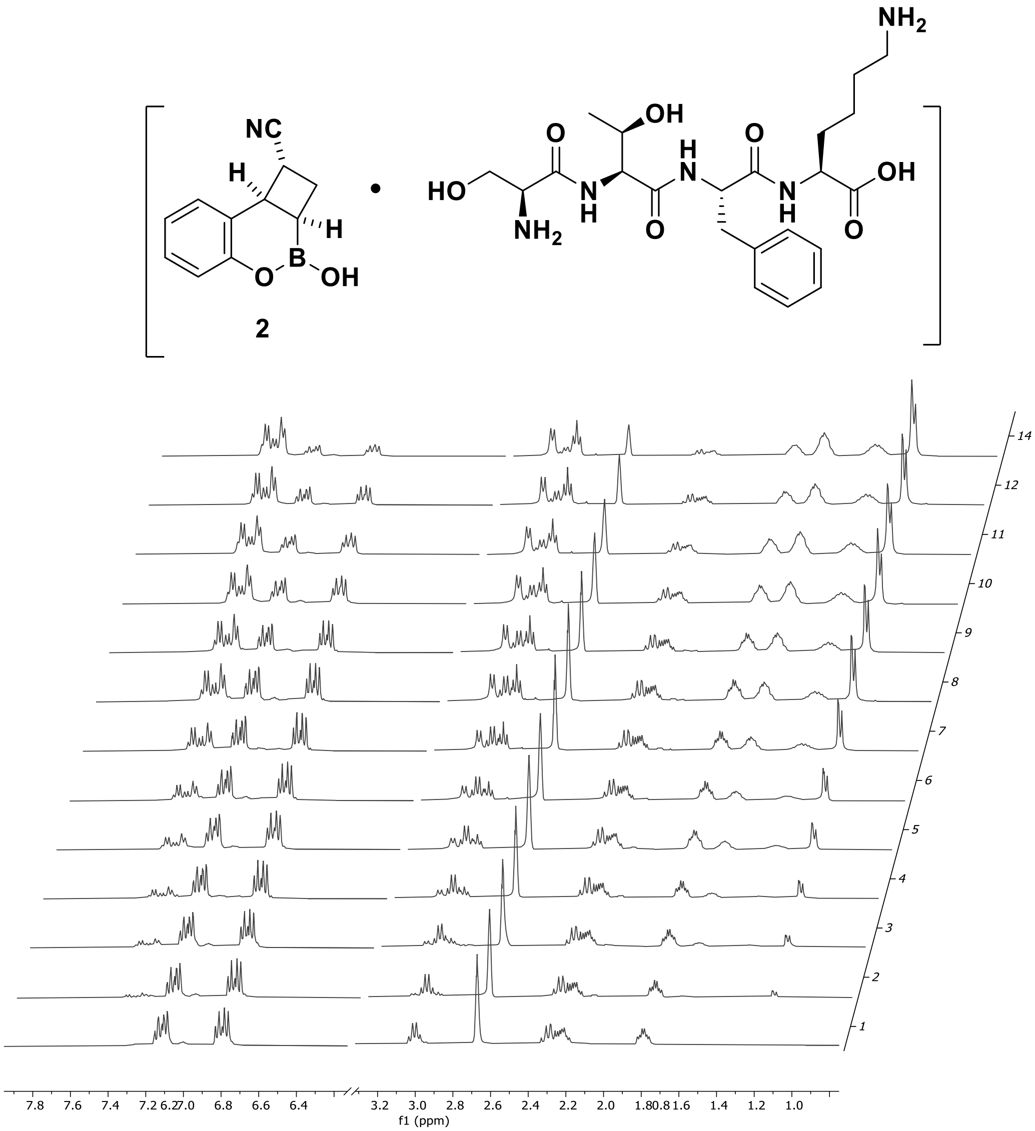

Figure S14:Binding study of 2 with Peptide S47 in 0.1 M phosphate buffer D2O: DMSO-d$_6$ (85:15) at pH = 7.4.

Comment: With increasing concentrations of peptide S39, the signals at 2.34, 2.27, and 1.83 ppm exhibit reduced resolution, suggesting interactions consistent with the formation of a peptide–boronate complex.

Experimental p*K*a Values

Table S17: Determined pKa values.

Entry	Structure	p*K*a
1	**1**	8.64
2	**2**	4.39
3	**2-*syn***	4.39

p*K*a Measurements for Benzoxaborine 1

According to a literature procedure by Hall *et al.*[30], **1** (36.7 mg, 0.25 mmol) was dissolved in 1 mL DMSO before the addition of 5 mL D_2O. The solution was then diluted to 25 mL using a freshly prepared phosphate buffer solution (690 mg Potassium phosphate monobasic in 50 mL water). From this stock solution 12 portions of different pH value were prepared using 1.0 M HCl, 1.0 M NaOH and 0.4 M NaOH and measure using a pH meter. After the pH was adjusted, 0.7 mL of each solution was transferred to an NMR tube and analyzed by ^{11}B NMR spectroscopy.

The measured ^{11}B NMR chemical shifts were plotted against the pH of the solution. To calculate the p*K*a, the function $\log\frac{(\delta_{highest})-(\delta_x)}{(\delta_x)-(\delta_{lowest})}$ was plotted against the pH. In this equation $\delta_{highest}$ is the most downfield chemical shift observed by ^{11}B NMR spectroscopy during the titrations and δ_{lowest} is the most upfield chemical shift observed. The p*K*a is determined by the y-intercept of this plot.

p*K*a Measurements for Benzoxaborine 2 and 2-*syn*.

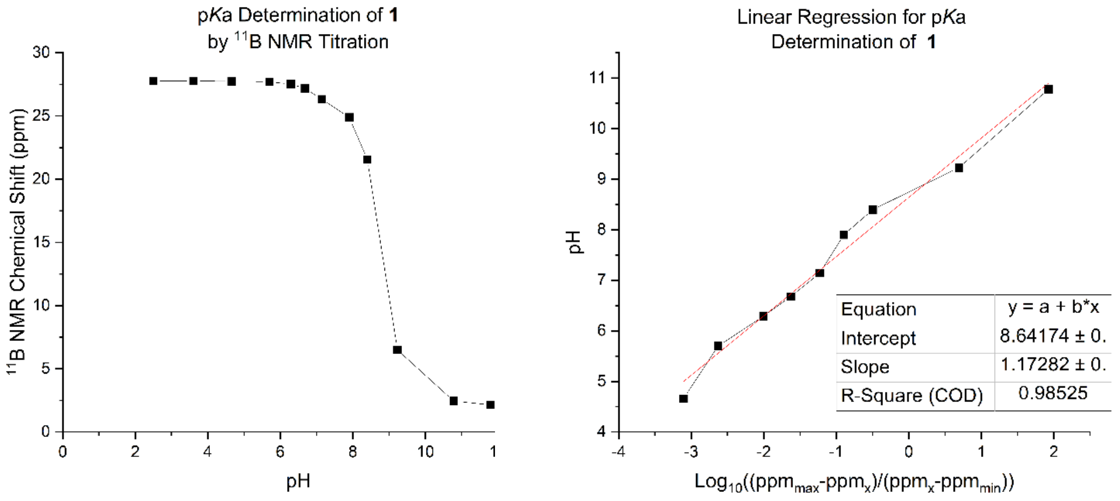

Figure S15: p*K*a determination of **1**.

According to an adapted literature procedure by Hall *et al.*[31] **2** or **2-*syn*** (1.99 mg, 0.01 mmol) was dissolved in 40 µL of DMSO-d_6 before the addition of 200 µL D_2O. The solution was then diluted to 1 mL using a freshly prepared phosphate buffer solution (138 mg Potassium phosphate monobasic in 10 mL water). To an NMR-tube was added 500 µL of the solution. The pH value of the solution was changed by addition of 1.0 M HCl, 1.0 M NaOH and 0.4 M NaOH and ^{11}B NMR spectra were measured immediately afterwards.

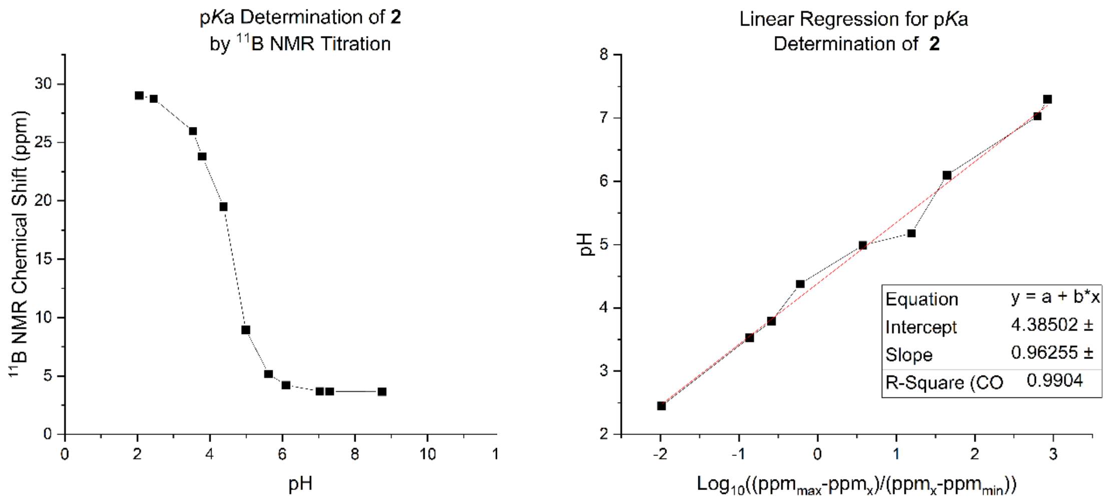

Figure S16: p*K*a determination of **2**.

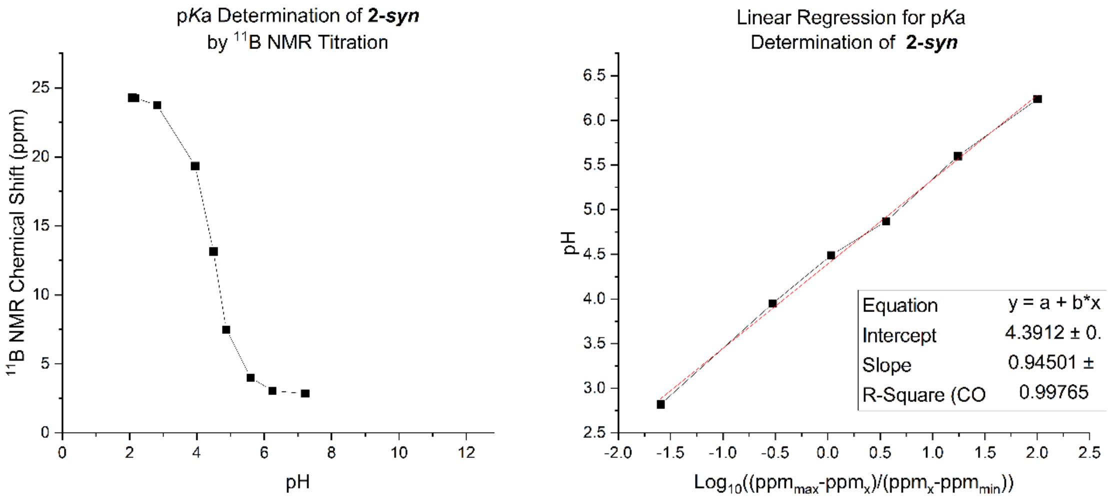

Figure S17: p*K*a determination of ***2-syn***.

FIA Predictions

To estimate the Lewis acidity of **1** and **2** the FIA (Fluoride Ion Affinity) prediction tool developed by Greb *et al.*[32] (available at https://www.grebgroup.de/fia-gnn) was employed. FIA-GNN is a graph neural network that predicts the fluoride ion affinity (FIA) of neutral p-block atom-based molecules in the gas and solution (dichloromethane) phase as defined by the fluorotrimethylsilane anchoring system. 13 different central atom types are included: B, Al, Ga, In, Si, Ge, Sn, Pb, P, As, Sb, Bi, and Te. The atoms of groups 14 and 15 are supported in their high (+IV, +V) and low (+II, +III) oxidation states.

Table S18: FIA Prediction of Boronheterocycles **1** and **2**.

Entry	Structure	FIA_{gas} (kJ/mol)	FIA_{solv} (kJ/mol)	Note
1	**1**	251	109	Lewis acid is in dataset (RI-DSD-BLYP-D3(BJ)/def2-QZVPP level of theory): FIA_{gas}: 240 [kJ/mol] FIA_{solv}: 101 [kJ/mol]
2	**2**	296	140	-

Stability in D_2O

In a 1 mL measuring flask, **1** (1.99 mg, 0.01 mmol) was dissolved in 40 µL DMSO-d_6 and subsequently 0.2 mL D_2O was added and the mixture was diluted to 1 mL with freshly prepared buffer (138 mg potassium phosphate monobasic in 10 mL D_2O). To an NMR was added 0.5 mL of the mixture and the pH was adjuster to 7.4 by adding 0.4 M NaOH and 1.0 M NaOH. Both ^{1}H NMR and ^{11}B NMR were measured at the corresponding time points.

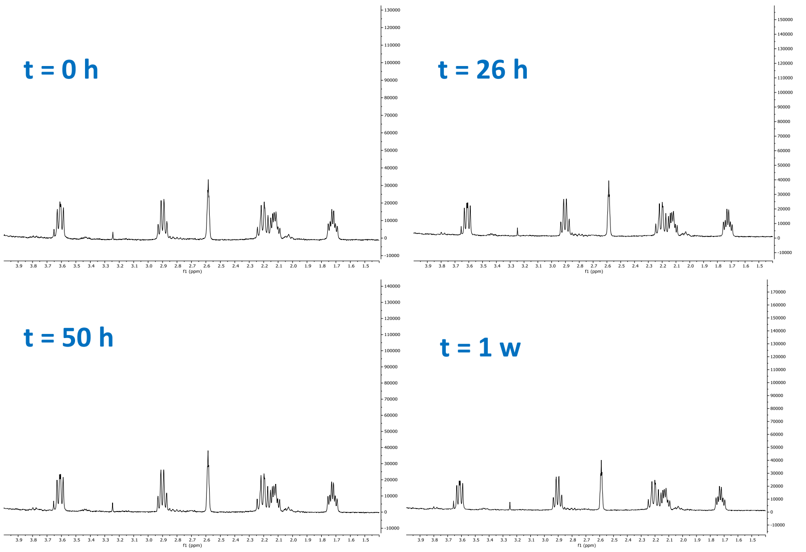

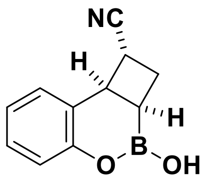

2

*Figure S18: Stability measurements by ^{1}H NMR of **2** in KH_2PO_4 buffer in D_2O at pH = 7.4.*

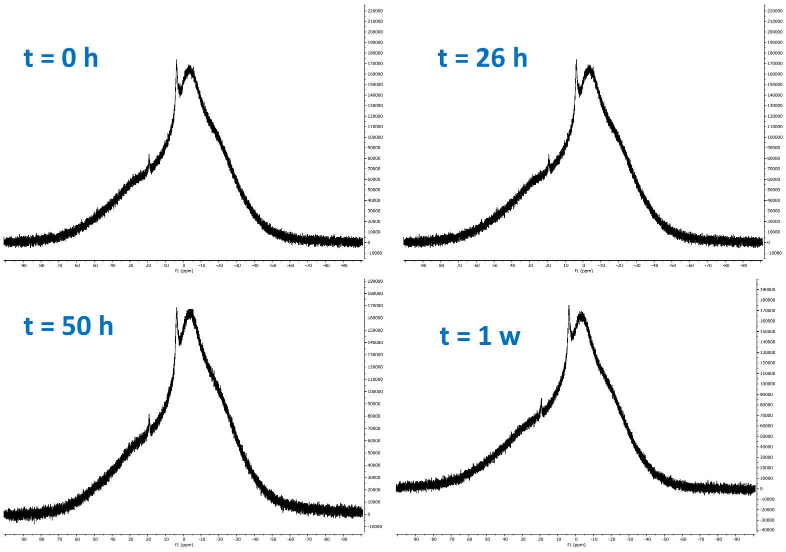

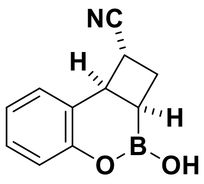

2

*Figure S19: Stability measurements by ^{11}B NMR of **2** in KH_2PO_4 buffer in D_2O at pH = 7.4.*

Comment: The ^{1}H and ^{11}B NMR spectra of **2** exhibit no significant changes over the duration of the study in KH_2PO_4 buffer at pH = 7.4, indicating that **2** remains stable under these conditions.

Crystal Data and Structure Refinement

Parameter	Value
Protein–Ligand Complex	CTX-M-14 – compound 7
Space group	P 32 2 1
Unit cell parameters	a = 41.5, b = 41.5, c = 231.3; α = β = γ = 90°
Wavelength	1.03319 Å
Temperature	100 K
Resolution range	46.3–1.31 Å (1.39–1.31 Å)
Total reflections	963,574 (75,992)
Unique reflections	104,115 (14,484)
Completeness (%)	97.1 (83.4)
Redundancy	9.25 (5.25)
⟨I/σ(I)⟩	18.32 (0.72)
Rmeas (%)	5.4 (194.3)
CC1/2	100 (28.6)
Refinement resolution range	38.55-1.4 Å
No. of reflections (work/test)	87,833 / 1,780
Rwork / Rfree (%)	19.3 / 21.4
No. of atoms (protein/ligand/water/ions)	2,021 / 16 / 236 / 0
Average B-factor ($Å^2$)	Protein: 26.1; Ligand: 25.5; Solvent: 34.66
RMSD bond lengths (Å)	0.003 Å
RMSD bond angles (°)	0.71°
Ramachandran favored (%)	97.69%
Ramachandran outliers (%)	0.38 %
PDB deposition	9R8F

Methods

Molecular cloning, Protein expression and purification.

The CTX-M-14 β-lactamase gene from Klebsiella pneumoniae, cloned into the pCR4 plasmid, was produced, purified, and crystallised as described before[33], with slight optimisations. Briefly, the pCR4:CTX-M-14 construct was transformed into competent E. coli BL21(DE3) pLysS cells (Novagen, Germany). Cells were grown at 37 °C in LB medium containing 100 µg/ml ampicillin for plasmid selection. Protein expression was induced with 1 mM isopropyl β-D-1-thiogalactopyranoside (IPTG) at an optical density (OD600) of 0.7, and cells were harvested 3 h post-induction by centrifugation at 5000 × g, 4 °C. The cell pellet was resuspended in 20 mM MES buffer pH 6 and lysed via sonication. Cell debris was removed by centrifugation at 20,000 × g for 1 h at 4 °C. The clarified supernatant was treated with 1 µl DNase and dialyzed overnight at 4 °C against 20 mM MES (pH 6). After filtration through a 0.2 µm syringe filter, the sample was loaded onto a cation exchange column (5 mL HiTrap SP FF, Cytiva), pre-equilibrated with 20 mM MES (pH 6) and purified using an ÄKTA Pure system. Elution was performed with a gradient of 100 mM NaCl in 20 mM MES (pH 6) over 10 column volumes. The eluted CTX-M-14 was concentrated using a 10 kDa Amicon Ultra-15 centrifugal unit (Merck) to a final concentration of 15 mg/mL.

Protein crystallisation and data collection. A freshly prepared protein solution at a concentration of 15 mg/mL was cleared by centrifugation at 12,000 g. Crystallisation was forced by a microseeding approach. Subsequently, CTX-M-14 crystals appeared within two days employing the sitting-drop vapor-diffusion method at 294 K. 2 µL of protein solution and 2 µL reservoir (30% PEG8000, 200 mM lithium sulphate, 100 mM sodium acetate pH 4.6) were mixed to equilibrate against 100 µL reservoir. To obtain CTX-M-14 complexed with the selected compound 7, a stock solution of 20 mM in reservoir solution supplemented

with 5% DMSO was prepared. Crystals were transferred to the soaking solution and soaked overnight and afterwards cryo-protected in mother liquor supplemented with 25% glycerol. Subsequently, fished crystals were flash-cooled in liquid nitrogen prior to data collection.

Diffraction data collection and structure determination. All X-ray diffraction data were collected using synchrotron radiation at 100 K (Beamline P11, Petra III, DESY, Hamburg, Germany)[34]. The diffraction data were processed using the XDS program package[35]. The structure was determined by molecular replacement with a solvent- and ligand-free structure of CTX-M-14 (PDB entry 6GTH)[33] as search model using the program phaser from the Phenix software suite (Version 1.19.2.4158; http:// www. phenix- online. org/)[36]. The structure was refined anisotropically for all non-solvent atoms using phenix.refine.[37] For manual model re-building the Coot software (Version 0.9.4)[38] was used and the inhibitor was built into the Fo— Fc difference electron density maps and verified by Polder omit maps.[39] POLYGON[40] and MolProbity[41] were used for the validation of the final model. The crystal structure of [Protein] in complex with [Ligand] was determined at 1.40 Å resolution. Crystals belong to space group P3(2)21 with unit cell parameters a = 41.5 Å, b = 41.5 Å, c = 231.3 Å. The structure was refined to Rwork = 19.3% and Rfree = 21.4%, with excellent geometry (Table S1). Figures were generated using PyMOL[42] (The PyMOL Molecular Graphics System, Version 3.1 Schrodinger, LLC., https://pymol.org).

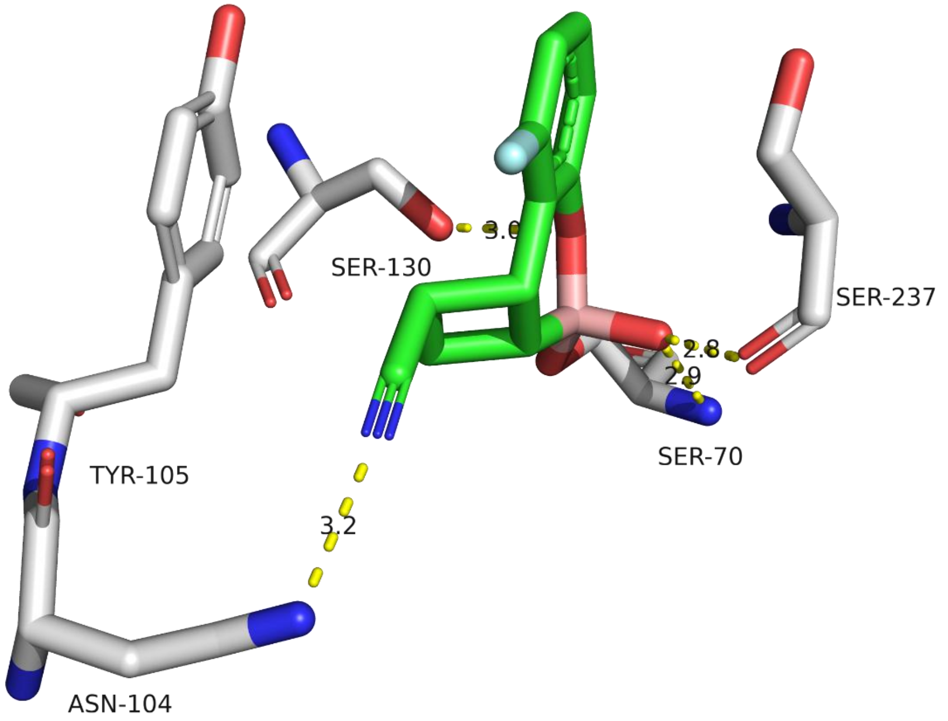

*Figure S20: Active site bonding of **7** with CTX-M-14 β-lactamase.*

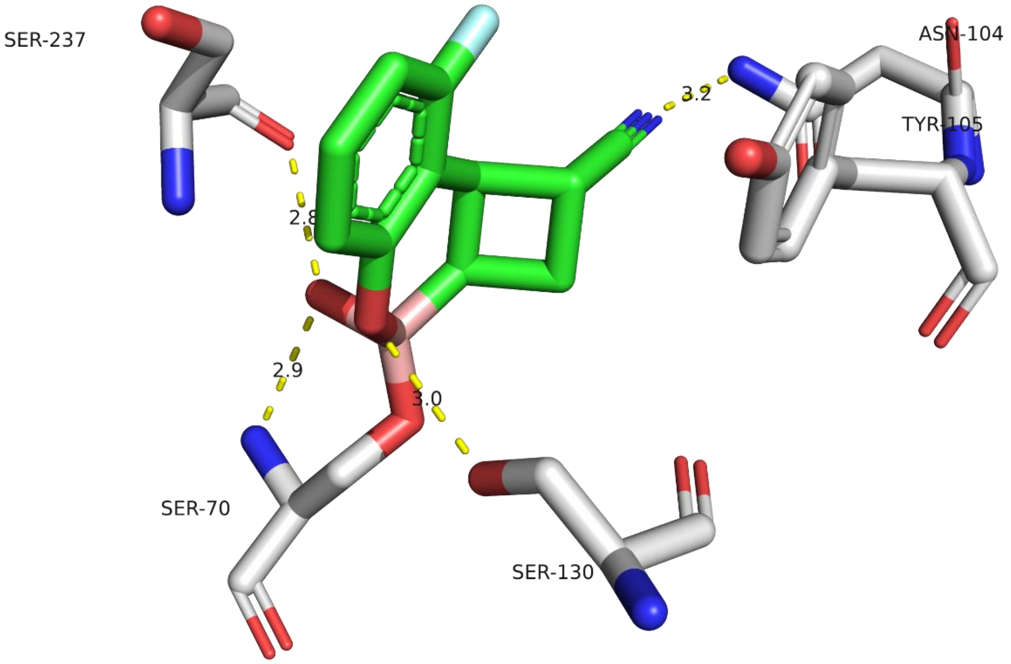

*Figure S21: Active site bonding of **7** with CTX-M-14 β-lactamase.*

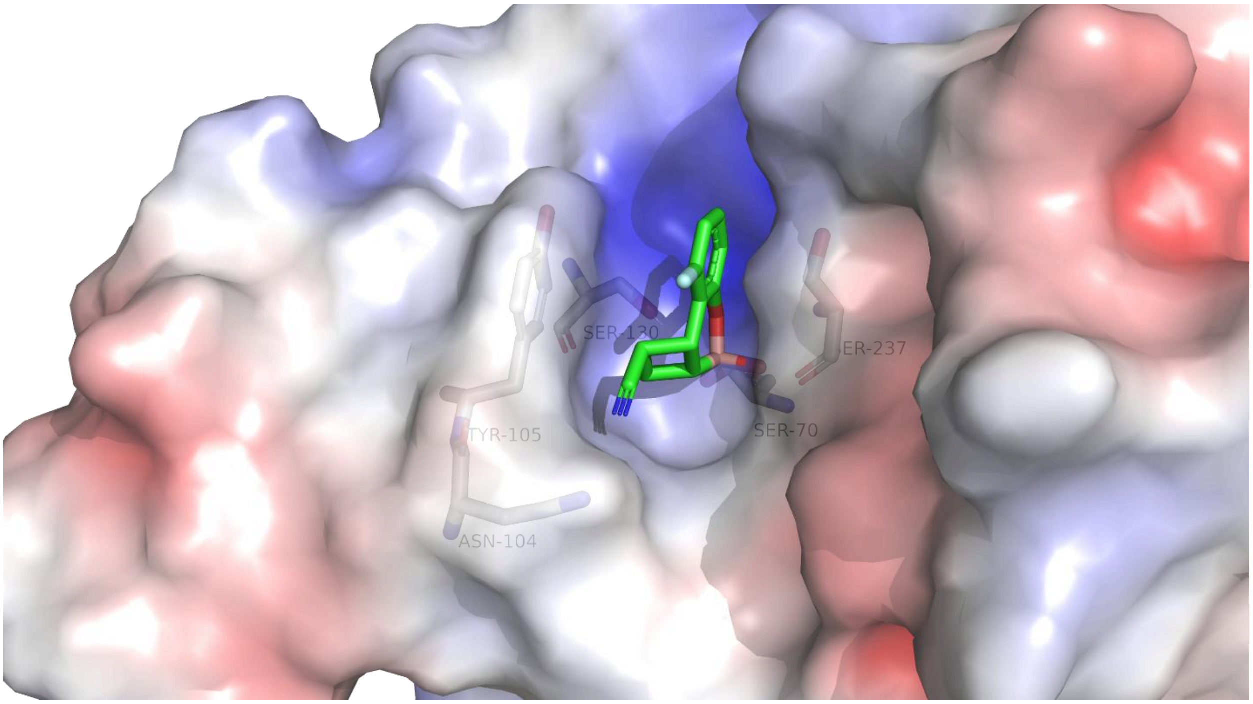

*Figure S22: Density map of the active site bonding of **7** with CTX-M-14 β-lactamase.*

References

1. L. Jarrige, F. Blanchard and G. Masson, Enantioselective Organocatalytic Intramolecular Aza-Diels–Alder Reaction, *Angew. Chem. Int. Ed.*, 2017, **56**, 10573-10576.
2. A. Seoane, N. Casanova, N. Quiñones, J. L. Mascareñas and M. Gulías, Straightforward Assembly of Benzoxepines by Means of a Rhodium(III)-Catalyzed C–H Functionalization of <i>o</i>-Vinylphenols, *J. Am. Chem. Soc.*, 2014, **136**, 834-837.
3. S. K. Murphy, A. Bruch and V. M. Dong, Substrate-Directed Hydroacylation: Rhodium-Catalyzed Coupling of Vinylphenols and Nonchelating Aldehydes, *Angew. Chem. Int. Ed.*, 2014, **53**, 2455-2459.
4. J. Hu, H. Hirao, Y. Li and J. Zhou, Palladium-Catalyzed Asymmetric Intermolecular Cyclization, *Angew. Chem. Int. Ed.*, 2013, **52**, 8676-8680.
5. W.-C. C. Lee, J. Wang, Y. Zhu and X. P. Zhang, Asymmetric Radical Bicyclization for Stereoselective Construction of Tricyclic Chromanones and Chromanes with Fused Cyclopropanes, *J. Am. Chem. Soc.*, 2023, **145**, 11622-11632.
6. S. S. Zimmerman, A. Khatri, E. C. Garnier-Amblard, P. Mullasseril, N. L. Kurtkaya, S. Gyoneva, K. B. Hansen, S. F. Traynelis and D. C. Liotta, Design, Synthesis, and Structure–Activity Relationship of a Novel Series of GluN2C-Selective Potentiators, *J. Med. Chem.*, 2014, **57**, 2334-2356.
7. L. Benhamou, D. W. Walker, D.-K. Bučar, A. E. Aliev and T. D. Sheppard, Synthesis of substituted benzooxaborinin-1-ols via palladium-catalysed cyclisation of alkenyl- and alkynyl-boronic acids, *Org. Biomol. Chem.*, 2016, **14**, 8039-8043.
8. S. J. Dolman, R. R. Schrock and A. H. Hoveyda, Enantioselective Synthesis of Cyclic Secondary Amines through Mo-Catalyzed Asymmetric Ring-Closing Metathesis (ARCM), *Org. Lett.*, 2003, **5**, 4899-4902.
9. H. He, W.-B. Liu, L.-X. Dai and S.-L. You, Ir-Catalyzed Cross-Coupling of Styrene Derivatives with Allylic Carbonates: Free Amine Assisted Vinyl C−H Bond Activation, *J. Am. Chem. Soc.*, 2009, **131**, 8346-8347.
10. H. Saito, S. Otsuka, K. Nogi and H. Yorimitsu, Nickel-Catalyzed Boron Insertion into the C2–O Bond of Benzofurans, *J. Am. Chem. Soc.*, 2016, **138**, 15315-15318.
11. S. R. Wisniewski, C. L. Guenther, O. A. Argintaru and G. A. Molander, A Convergent, Modular Approach to Functionalized 2,1-Borazaronaphthalenes from 2-Aminostyrenes and Potassium Organotrifluoroborates, *J. Org. Chem.*, 2014, **79**, 365-378.
12. S. H. Boyer, A. Gonzalez-De-Castro, J. A. H. Dielemans, L. Lefort, Z. Zhu, M. Gnahn, J. Schörghuber, S. Steinhofer, A. H. M. De Vries and S. J. Hecker, Scalable Synthesis of β-Lactamase Inhibitor QPX7728 by Sequential Nickel-Catalyzed Boron Insertion into a Benzofuran Substrate and Enantioselective Cyclopropanation of the Resulting Vinylboronate, *Org. Process Res. Dev*, 2022, **26**, 925-935.
13. H. B. Minshull and G. C. Lloyd-Jones, TMSCF₃-Mediated Conversion of Salicylates into α,α-Difluoro-3-coumaranones: Chain Kinetics, Anion-Speciation, and Mechanism, *J. Org. Chem.*, 2023, **88**, 17450-17460.
14. A. Marotta, H. M. Kortman, C. Interdonato, P. H. Seeberger and J. J. Molloy, Convergent synthesis of bicyclic boronates <i>via</i> a cascade regioselective Suzuki–Miyaura/cyclisation protocol, *Chem. Commun.*, 2024, **60**, 13223-13226.
15. J. Poldy, R. Peakall and R. A. Barrow, Identification of the First Alkenyl Chiloglottone Congener, *Eur. J. Org. Chem.*, 2012, **2012**, 5818-5827.
16. J. Y. Wu, B. N. Stanzl and T. Ritter, A Strategy for the Synthesis of Well-Defined Iron Catalysts and Application to Regioselective Diene Hydrosilylation, *J. Am. Chem. Soc.*, 2010, **132**, 13214-13216.

17. J. D. Wilden, L. Geldeard, C. C. Lee, D. B. Judd and S. Caddick, Trichlorophenol (TCP) sulfonate esters: A selective alternative to pentafluorophenol (PFP) esters and sulfonyl chlorides for the preparation of sulfonamides, *Chem. Commun.*, 2007, DOI: 10.1039/b614604j, 1074-1076.
18. E. Richmond and J. Moran, Ligand Control of <i>E</i>/<i>Z</i> Selectivity in Nickel-Catalyzed Transfer Hydrogenative Alkyne Semireduction, *J. Org. Chem.*, 2015, **80**, 6922-6929.
19. T. Ishiyama, N. Matsuda, N. Miyaura and A. Suzuki, Platinum(0)-catalyzed diboration of alkynes, *J. Am. Chem. Soc.*, 1993, **115**, 11018-11019.
20. K. Yuan and M. J. Ingleson, Haloboration of o-Alkynyl Phenols Generates Halogenated Bicyclic-Boronates, *Angew. Chem. Int. Ed.*, 2023, **62**, e202301463.
21. J. Bucher, T. Wurm, K. S. Nalivela, M. Rudolph, F. Rominger and A. S. K. Hashmi, Cyclization of Gold Acetylides: Synthesis of Vinyl Sulfonates via Gold Vinylidene Complexes, *Angew. Chem. Int. Ed.*, 2014, **53**, 3854-3858.
22. T. Qin, J. Cornella, C. Li, L. R. Malins, J. T. Edwards, S. Kawamura, B. D. Maxwell, M. D. Eastgate and P. S. Baran, A general alkyl-alkyl cross-coupling enabled by redox-active esters and alkylzinc reagents, *Science*, 2016, **352**, 801-805.
23. C. H. Krauch, S. Farid and G. O. Schenck, Photo-C₄-Cyclodimerisation von Cumarin, *Chem. Ber.*, 1966, **99**, 625-633.
24. G. W. T. M. J. Frisch, H. B. Schlegel, G. E. Scuseria, M. A. Robb, J. R. Cheeseman, G. Scalmani, V. Barone, G. A. Petersson, H. Nakatsuji, X. Li, M. Caricato, A. V. Marenich, J. Bloino, B. G. Janesko, R. Gomperts, B. Mennucci, H. P. Hratchian, J. V. Ortiz, A. F. Izmaylov, J. L. Sonnenberg, D. Williams-Young, F. Ding, F. Lipparini, F. Egidi, J. Goings, B. Peng, A. Petrone, T. Henderson, D. Ranasinghe, V. G. Zakrzewski, J. Gao, N. Rega, G. Zheng, W. Liang, M. Hada, M. Ehara, K. Toyota, R. Fukuda, J. Hasegawa, M. Ishida, T. Nakajima, Y. Honda, O. Kitao, H. Nakai, T. Vreven, K. Throssell, J. A. Montgomery, Jr., J. E. Peralta, F. Ogliaro, M. J. Bearpark, J. J. Heyd, E. N. Brothers, K. N. Kudin, V. N. Staroverov, T. A. Keith, R. Kobayashi, J. Normand, K. Raghavachari, A. P. Rendell, J. C. Burant, S. S. Iyengar, J. Tomasi, M. Cossi, J. M. Millam, M. Klene, C. Adamo, R. Cammi, J. W. Ochterski, R. L. Martin, K. Morokuma, O. Farkas, J. B. Foresman, D. J. Fox, Gaussian 16 Revision A.03).
25. A. D. Becke, Density-functional thermochemistry. III. The role of exact exchange, *J. Chem. Phys.*, 1993, **98**, 5648-5652.
26. S. Grimme, J. Antony, S. Ehrlich and H. Krieg, A consistent and accurate ab initio parametrization of density functional dispersion correction (DFT-D) for the 94 elements H-Pu, *J. Chem. Phys.*, 2010, **132**, 154104.
27. G. Scalmani and M. J. Frisch, Continuous surface charge polarizable continuum models of solvation. I. General formalism, *J. Chem. Phys.*, 2010, **132**, 114110.
28. D. I. Schuster, G. Lem and N. A. Kaprinidis, New insights into an old mechanism: [2 + 2] photocycloaddition of enones to alkenes, *Chem. Rev.*, 1993, **93**, 3-22.
29. C. Körner, P. Starkov and T. D. Sheppard, An Alternative Approach to Aldol Reactions: Gold-Catalyzed Formation of Boron Enolates from Alkynes, *J. Am. Chem. Soc.*, 2010, **132**, 5968-5969.
30. M. Dowlut and D. G. Hall, An Improved Class of Sugar-Binding Boronic Acids, Soluble and Capable of Complexing Glycosides in Neutral Water, *J. Am. Chem. Soc.*, 2006, **128**, 4226-4227.
31. M. Z. H. Kazmi, J. P. G. Rygus, H. T. Ang, M. Paladino, M. A. Johnson, M. J. Ferguson and D. G. Hall, Lewis or Brønsted? A Rectification of the Acidic and Aromatic Nature of Boranol-Containing Naphthoid Heterocycles, *J. Am. Chem. Soc.*, 2021, **143**, 10143-10156.
32. L. M. Sigmund, S. S. S, A. Albers, P. Erdmann, R. S. Paton and L. Greb, Predicting Lewis Acidity: Machine Learning the Fluoride Ion Affinity of <i>p</i>-Block-Atom-Based Molecules, *Angew. Chem. Int. Ed.*, 2024, **63**, e202401084.

33. M. O. Wiedorn, D. Oberthür, R. Bean, R. Schubert, N. Werner, B. Abbey, M. Aepfelbacher, L. Adriano, A. Allahgholi, N. Al-Qudami, J. Andreasson, S. Aplin, S. Awel, K. Ayyer, S. Bajt, I. Barák, S. Bari, J. Bielecki, S. Botha, D. Boukhelef, W. Brehm, S. Brockhauser, I. Cheviakov, M. A. Coleman, F. Cruz-Mazo, C. Danilevski, C. Darmanin, R. B. Doak, M. Domaracky, K. Dörner, Y. Du, H. Fangohr, H. Fleckenstein, M. Frank, P. Fromme, A. M. Gañán-Calvo, Y. Gevorkov, K. Giewekemeyer, H. M. Ginn, H. Graafsma, R. Graceffa, D. Greiffenberg, L. Gumprecht, P. Göttlicher, J. Hajdu, S. Hauf, M. Heymann, S. Holmes, D. A. Horke, M. S. Hunter, S. Imlau, A. Kaukher, Y. Kim, A. Klyuev, J. Knoška, B. Kobe, M. Kuhn, C. Kupitz, J. Küpper, J. M. Lahey-Rudolph, T. Laurus, K. Le Cong, R. Letrun, P. L. Xavier, L. Maia, F. R. N. C. Maia, V. Mariani, M. Messerschmidt, M. Metz, D. Mezza, T. Michelat, G. Mills, D. C. F. Monteiro, A. Morgan, K. Mühlig, A. Munke, A. Münnich, J. Nette, K. A. Nugent, T. Nuguid, A. M. Orville, S. Pandey, G. Pena, P. Villanueva-Perez, J. Poehlsen, G. Previtali, L. Redecke, W. M. Riekehr, H. Rohde, A. Round, T. Safenreiter, I. Sarrou, T. Sato, M. Schmidt, B. Schmitt, R. Schönherr, J. Schulz, J. A. Sellberg, M. M. Seibert, C. Seuring, M. L. Shelby, R. L. Shoeman, M. Sikorski, A. Silenzi, C. A. Stan, X. Shi, S. Stern, J. Sztuk-Dambietz, J. Szuba, A. Tolstikova, M. Trebbin, U. Trunk, P. Vagovic, T. Ve, B. Weinhausen, T. A. White, K. Wrona, C. Xu, O. Yefanov, N. Zatsepin, J. Zhang, M. Perbandt, A. P. Mancuso, C. Betzel, H. Chapman and A. Barty, Megahertz serial crystallography, *Nat. Commun.*, 2018, **9**, 4025.
34. A. Burkhardt, B. Reime, T. Pakendorf, J. Roever, N. Stuebe, J. Meyer, P. Fischer, S. Panneerselvam and A. Meents, The Crystallography Endstation at Beamline P11 at PETRA III., *Acta Cryst.*, 2013, **A69**, s401.
35. W. Kabsch, XDS, *Acta Crystallogr. D*, 2010, **66**, 125-132.
36. D. Liebschner, P. V. Afonine, M. L. Baker, G. Bunkoczi, V. B. Chen, T. I. Croll, B. Hintze, L.-W. Hung, S. Jain, A. J. McCoy, N. W. Moriarty, R. D. Oeffner, B. K. Poon, M. G. Prisant, R. J. Read, J. S. Richardson, D. C. Richardson, M. D. Sammito, O. V. Sobolev, D. H. Stockwell, T. C. Terwilliger, A. G. Urzhumtsev, L. L. Videau, C. J. Williams and P. D. Adams, Macromolecular structure determination using X-rays, neutrons and electrons: recent developments in Phenix, *Acta Crystallogr. D*, 2019, **75**, 861-877.
37. P. V. Afonine, R. W. Grosse-Kunstleve, N. Echols, J. J. Headd, N. W. Moriarty, M. Mustyakimov, T. C. Terwilliger, A. Urzhumtsev, P. H. Zwart and P. D. Adams, Towards automated crystallographic structure refinement with phenix.refine, *Acta Crystallogr. D*, 2012, **68**, 352-367.
38. P. Emsley, B. Lohkamp, W. G. Scott and K. Cowtan, Features and development of Coot, *Acta Crystallogr. D*, 2010, **66**, 486-501.
39. D. Liebschner, P. V. Afonine, N. W. Moriarty, B. K. Poon, O. V. Sobolev, T. C. Terwilliger and P. D. Adams, Polder maps: improving OMIT maps by excluding bulk solvent, *Acta Crystallogr. D*, 2017, **73**, 148-157.
40. A. Urzhumtsev, P. V. Afonine, L. Urzhumtseva and P. D. Adams, POLYGON and other tools: model validation at a glance., *Acta Crystallogr. a-Found. Adv.* , 2010, **66**, S311-S312.
41. C. J. Williams, J. J. Headd, N. W. Moriarty, M. G. Prisant, L. L. Videau, L. N. Deis, V. Verma, D. A. Keedy, B. J. Hintze, V. B. Chen, S. Jain, S. M. Lewis, W. B. Arendall III, J. Snoeyink, P. D. Adams, S. C. Lovell, J. S. Richardson and D. C. Richardson, MolProbity: More and better reference data for improved all-atom structure validation, *Protein Sci.*, 2018, **27**, 293-315.
42. L. Schrödinger and W. L. DeLano, (PyMOL. Retrieved from http://www.pymol.org/)).

NMR Data

^{1}H-NMR of S1 (400 MHz, $CDCl_3$)

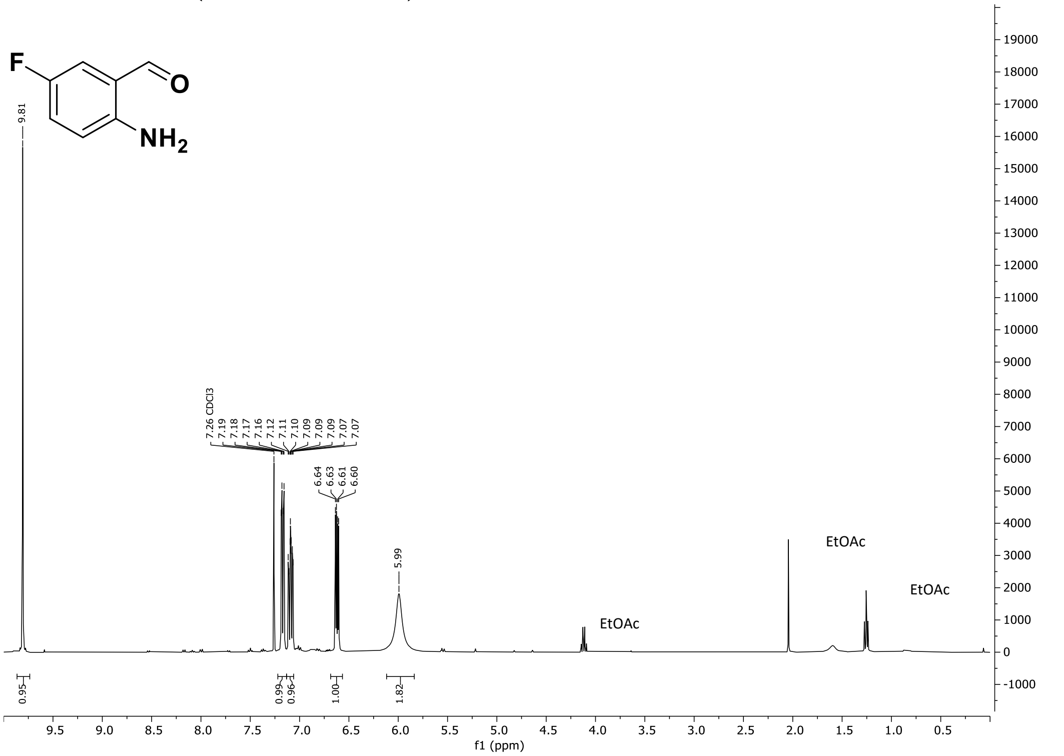

^{1}H-NMR of S2 (400 MHz, $CDCl_3$)

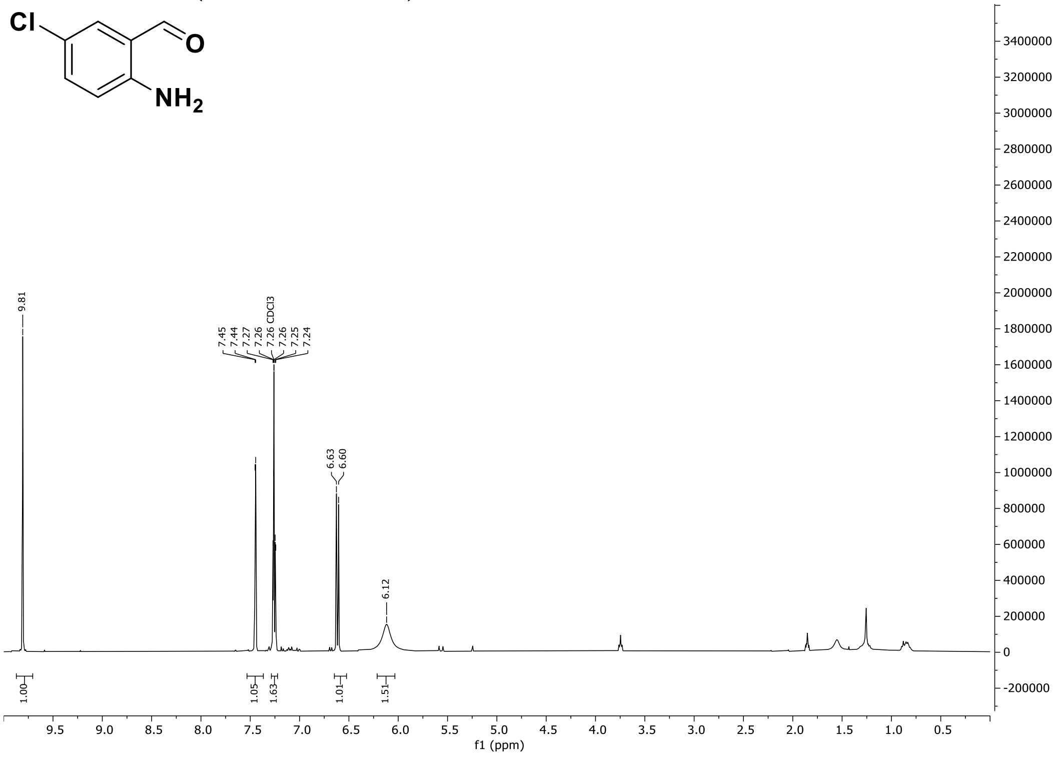

^{1}H-NMR of S3 (400 MHz, $CDCl_3$)

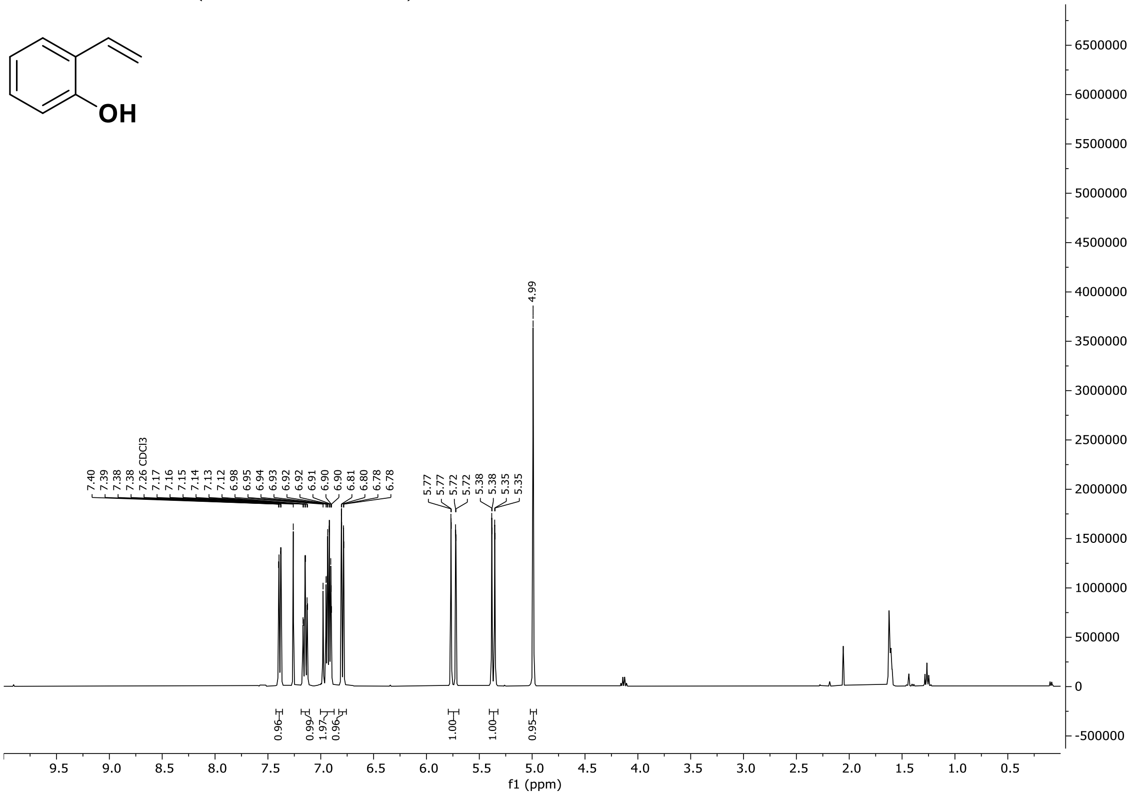

^{1}H-NMR of S4 (400 MHz, $CDCl_3$)

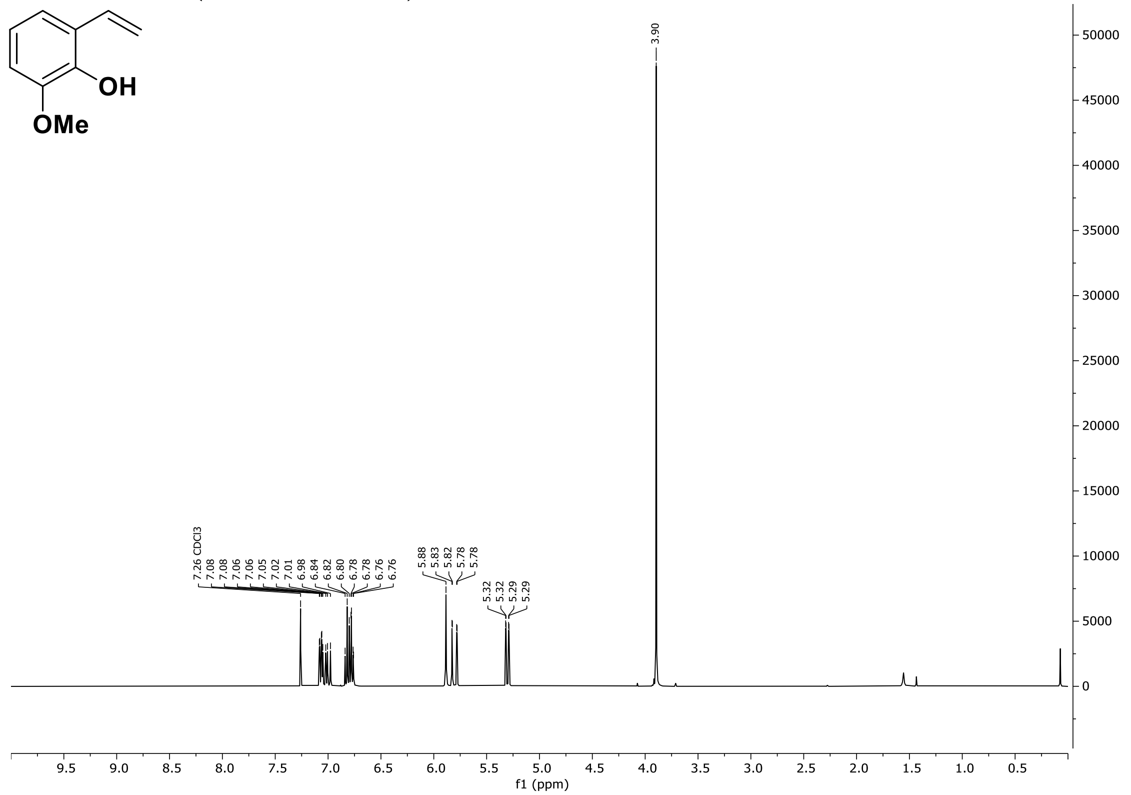

^{1}H-NMR of S5 (400 MHz, $CDCl_3$)

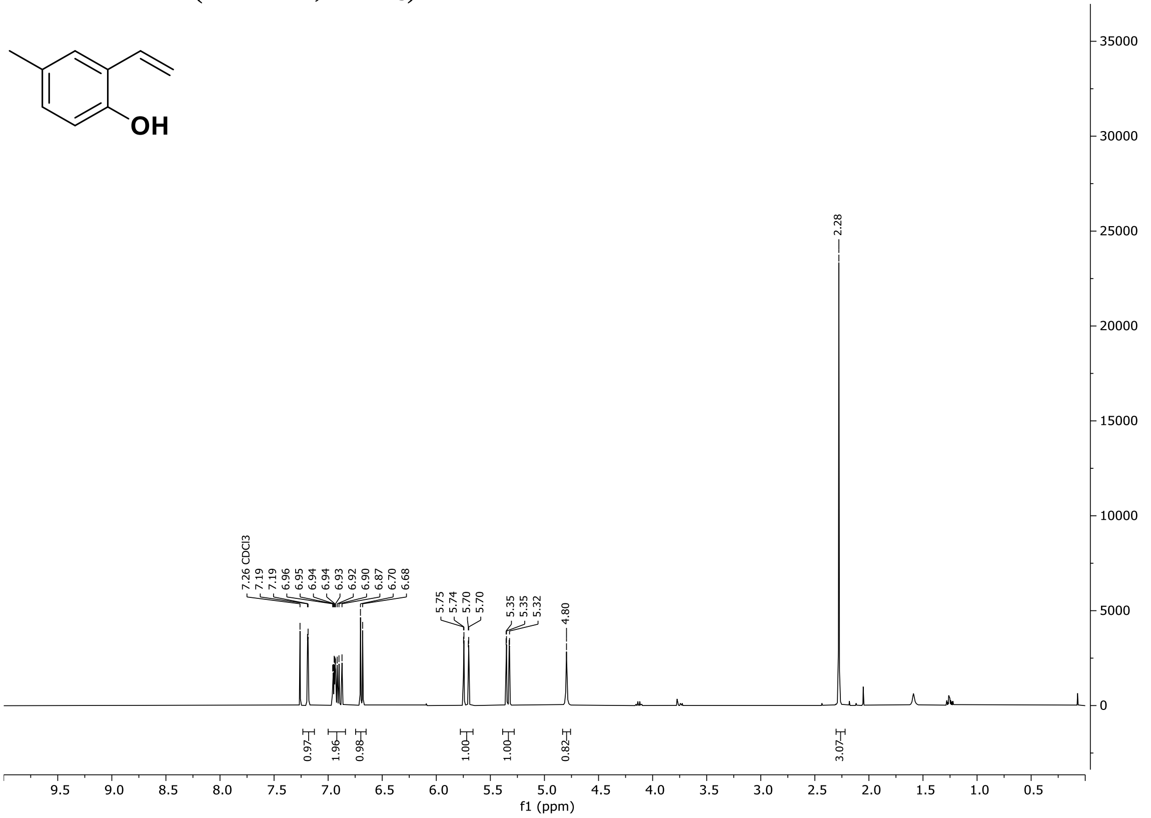

^{1}H-NMR of S6 (600 MHz, $CDCl_3$)

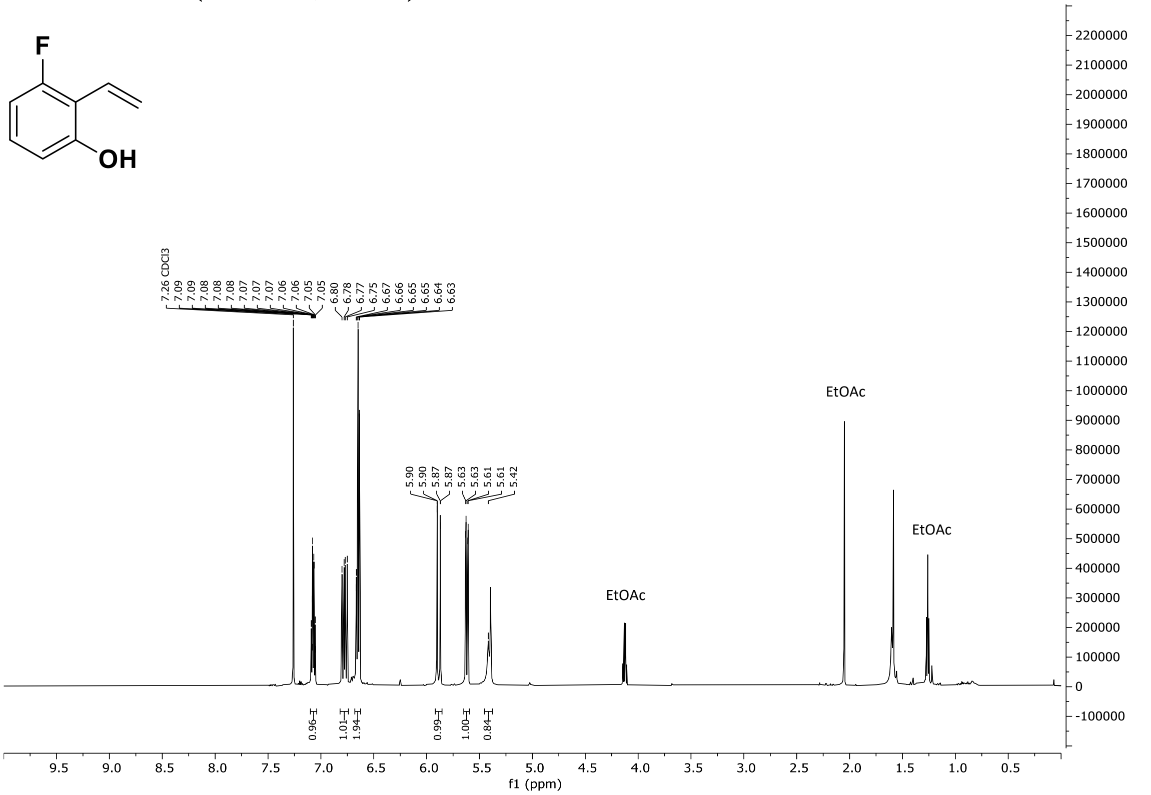

^{1}H-NMR of S7 (400 MHz, $CDCl_3$)

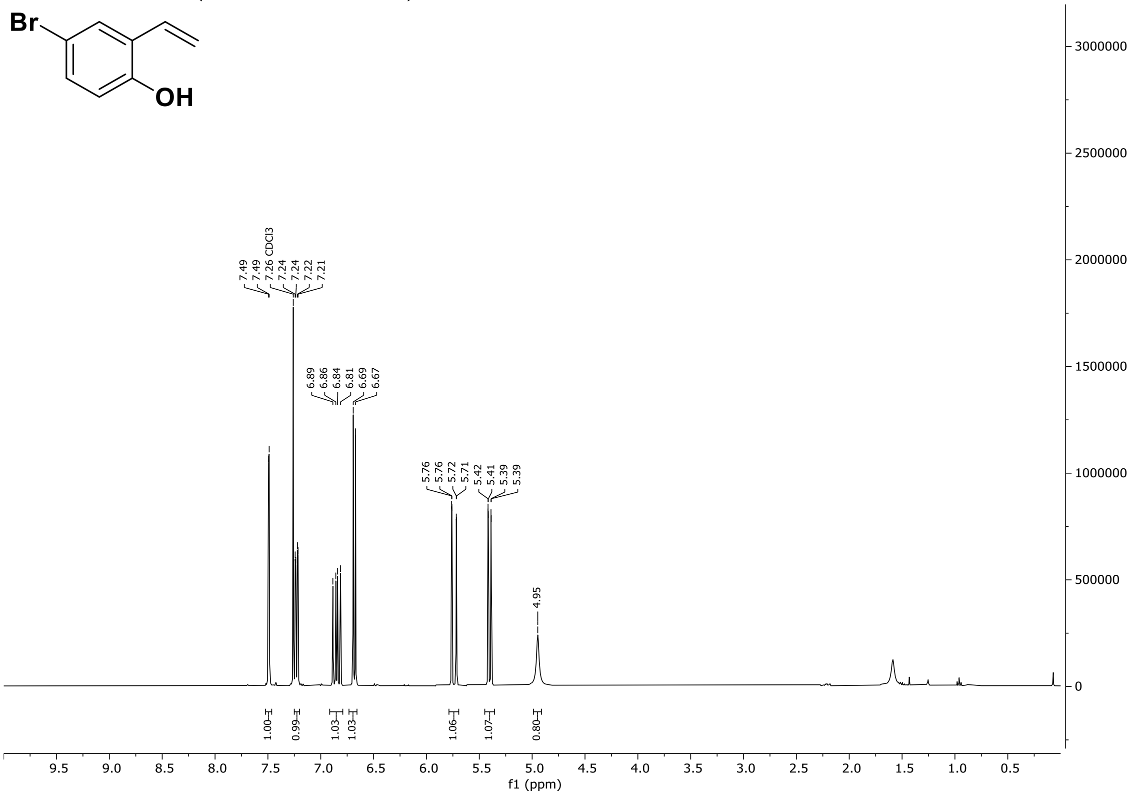

^{1}H-NMR of S8 (400 MHz, $CDCl_3$)

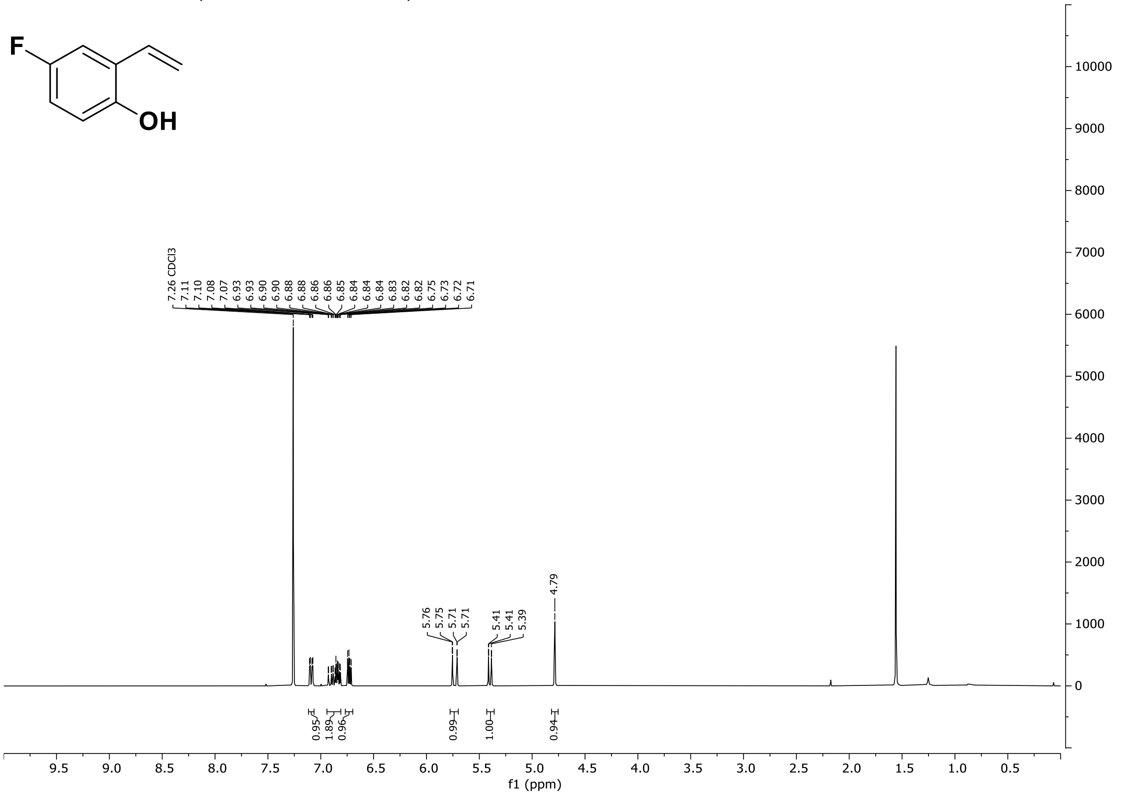

^{1}H-NMR of S9 (400 MHz, $CDCl_3$)

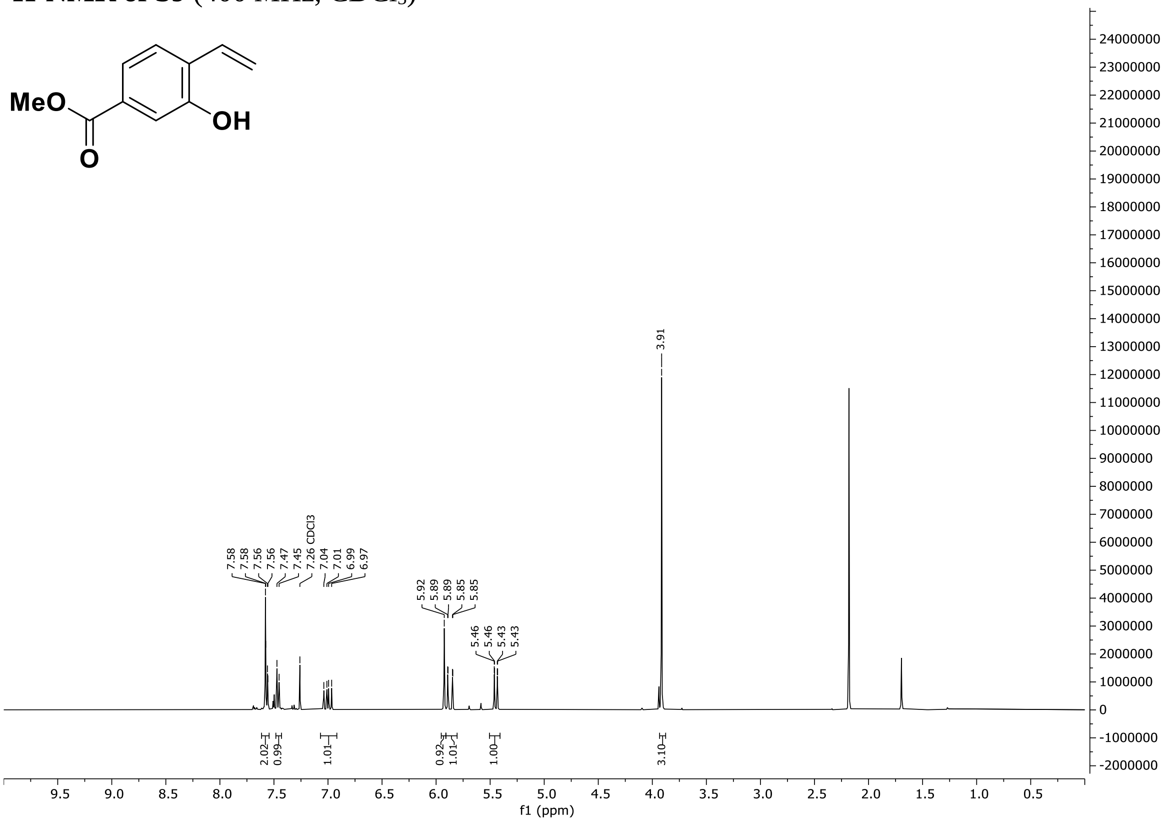

^{1}H-NMR of S10 (400 MHz, $CDCl_3$)

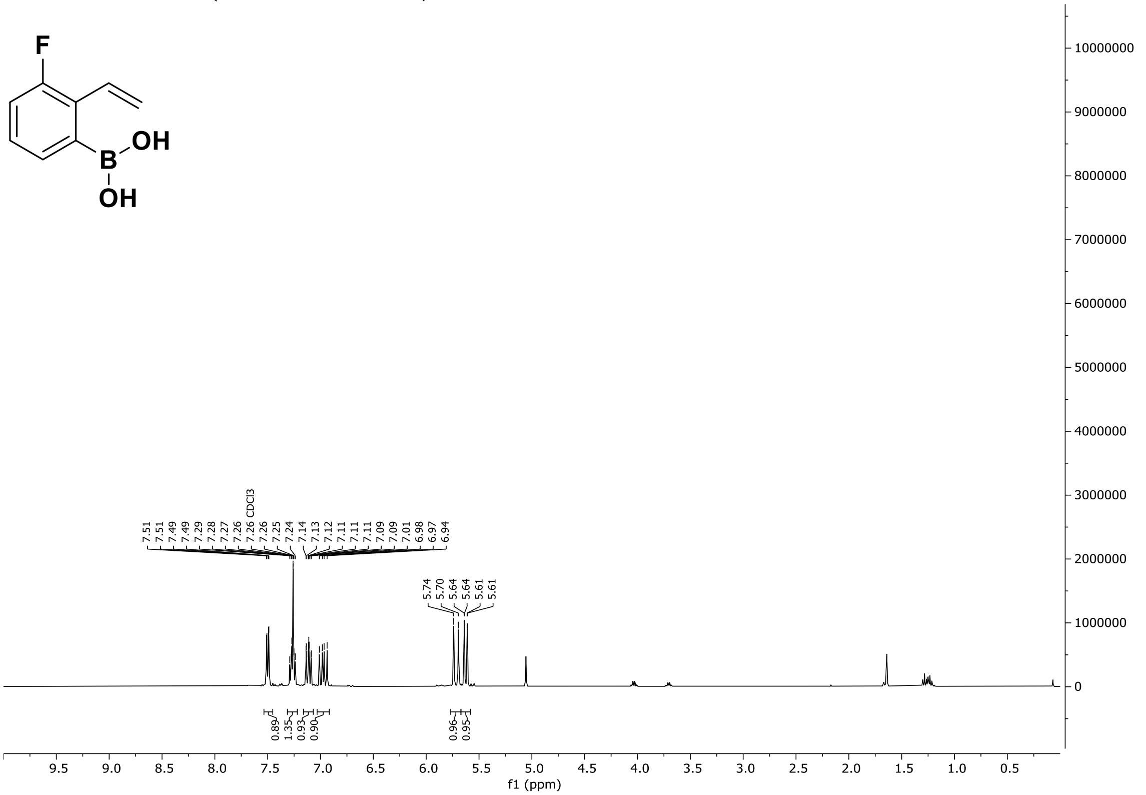

^{13}C-NMR of S10 (100 MHz, $CDCl_3$)

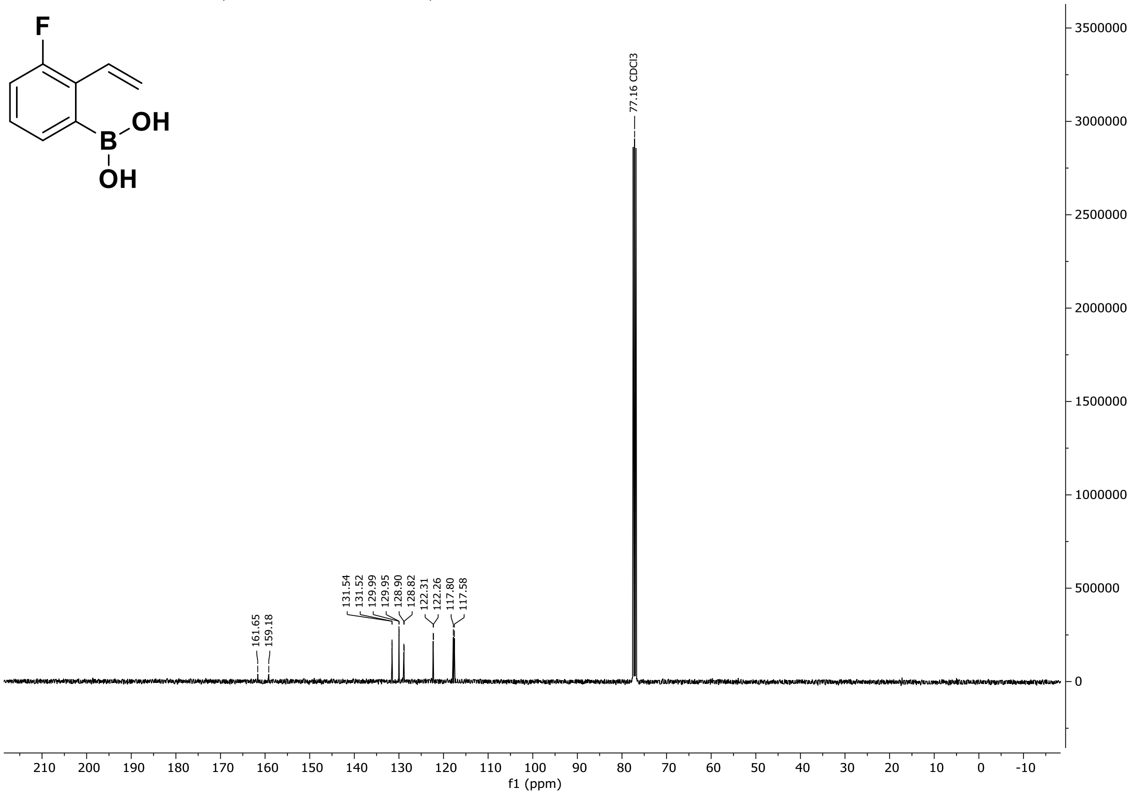

^{11}B-NMR of S10 (128 MHz, $CDCl_3$)

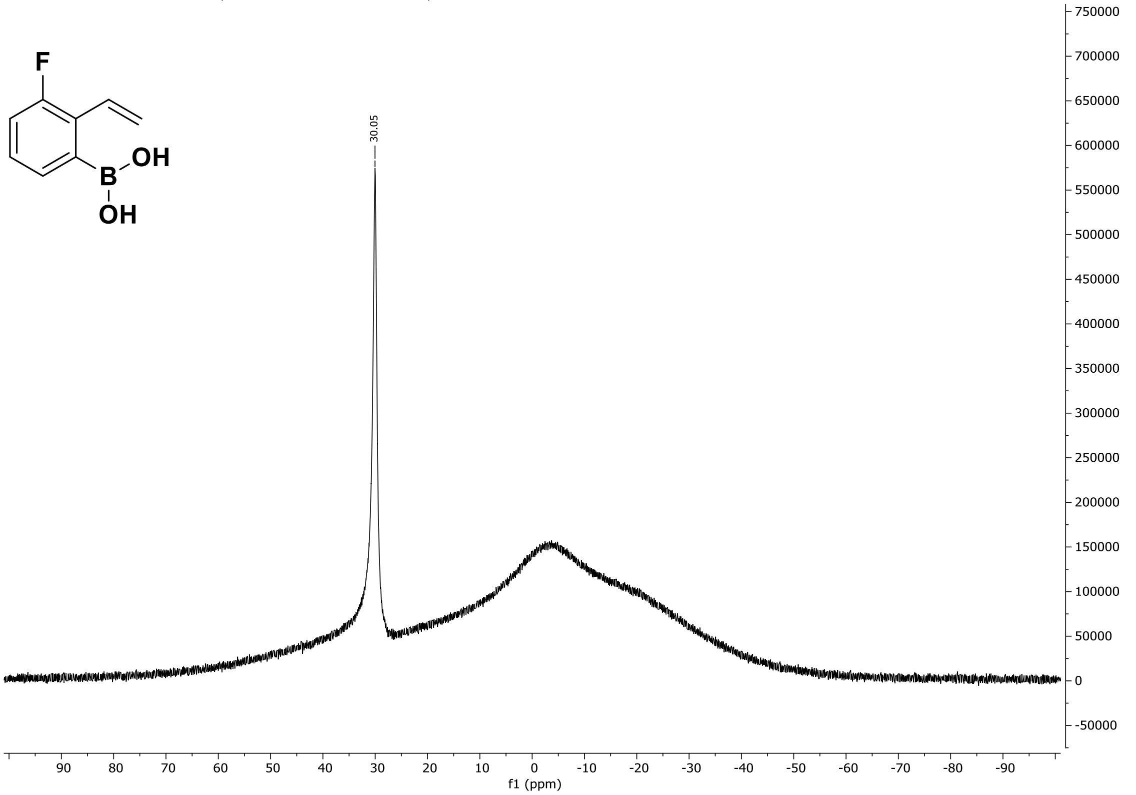

^{19}F-NMR of S10 (376 MHz, $CDCl_3$)

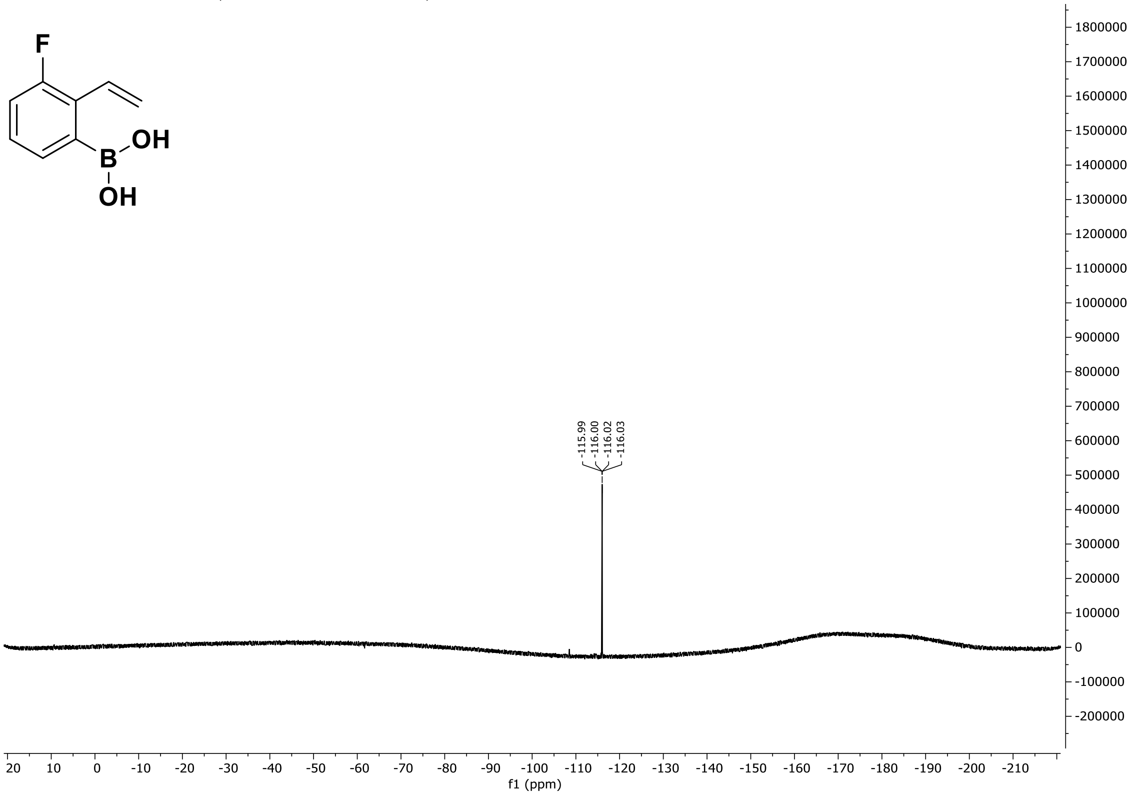

^{1}H-NMR of S11 (400 MHz, $CDCl_3$)

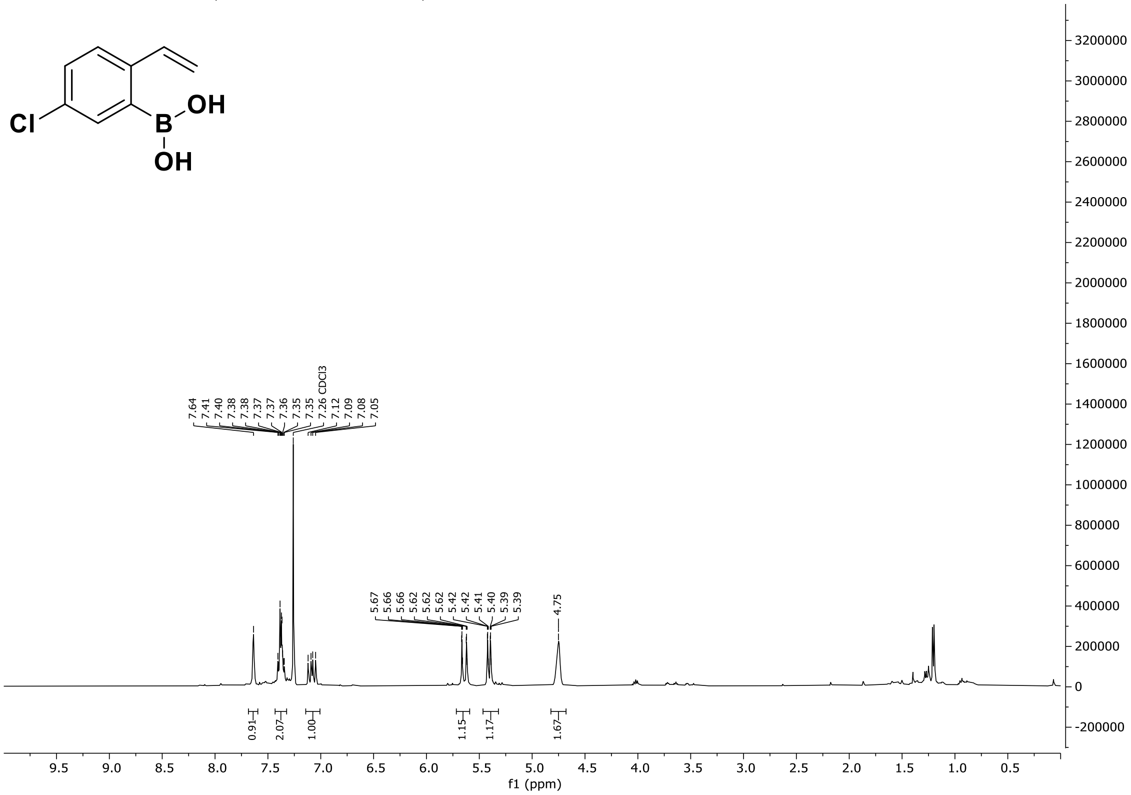

^{13}C-NMR of S11 (100 MHz, $CDCl_3$)

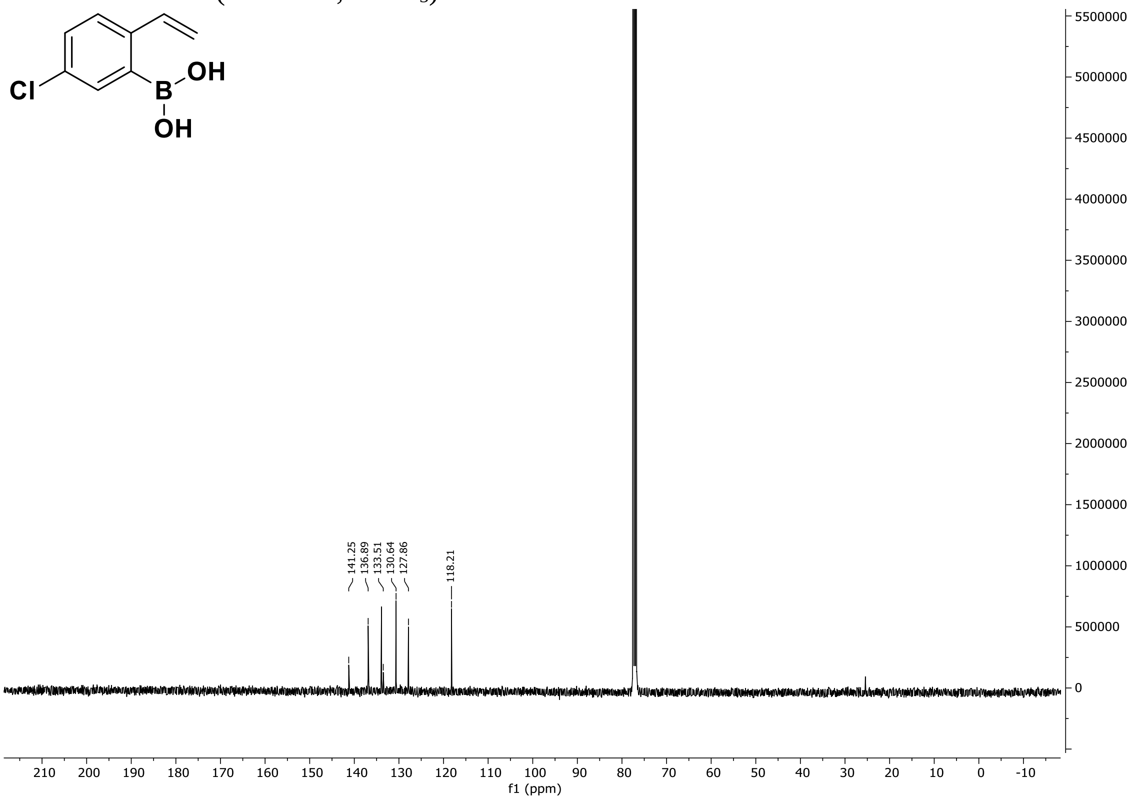

^{11}B-NMR of S11 (128 MHz, $CDCl_3$)

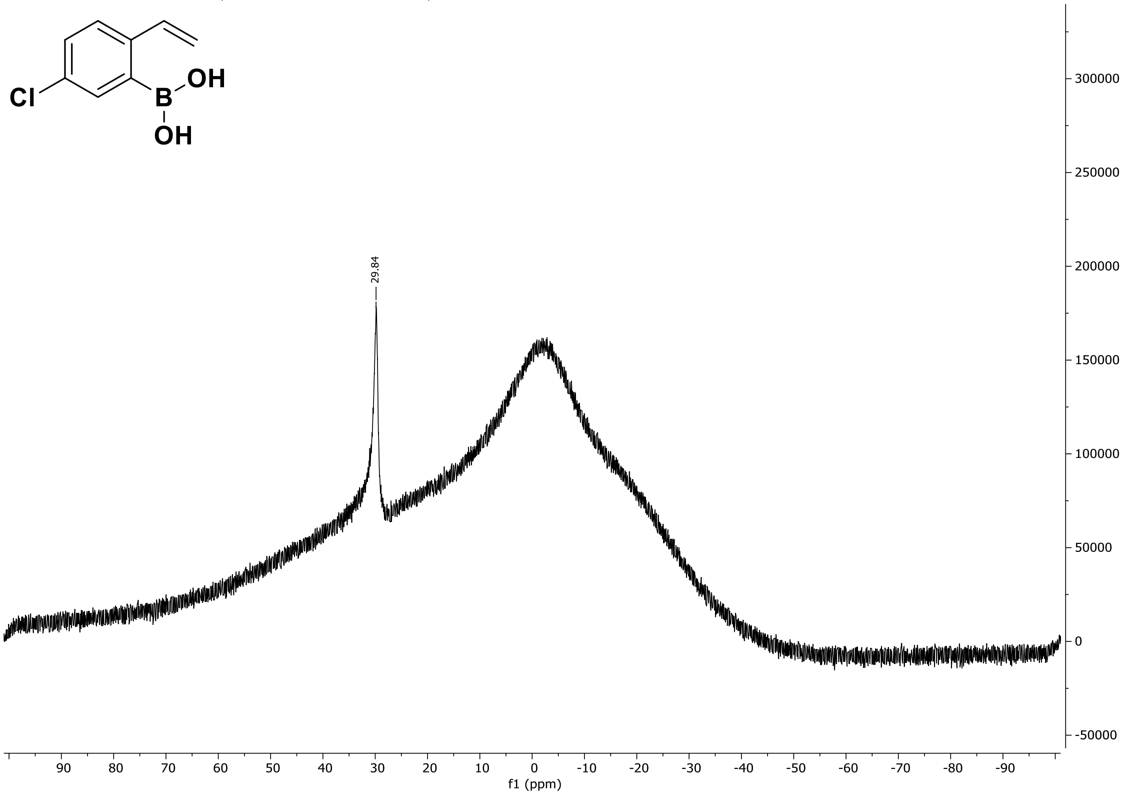

^{1}H-NMR of S12 (400 MHz, $CDCl_3$)

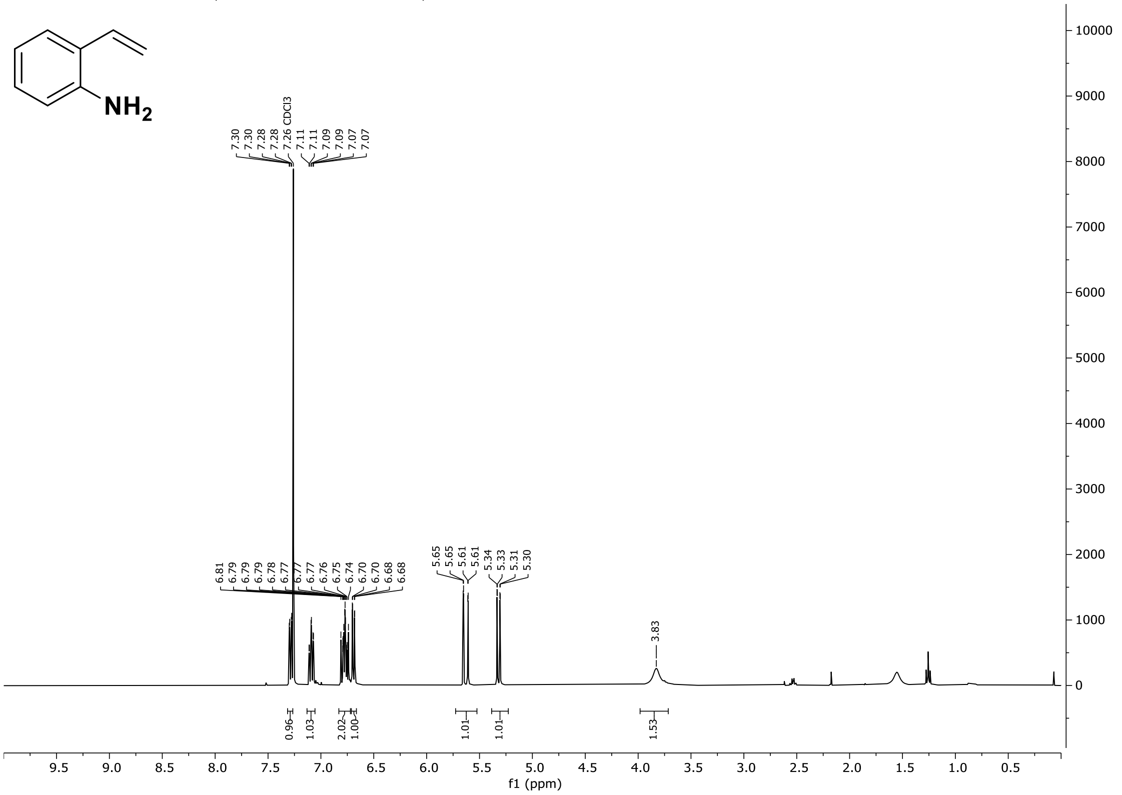

^{1}H-NMR of S13 (400 MHz, $CDCl_3$)

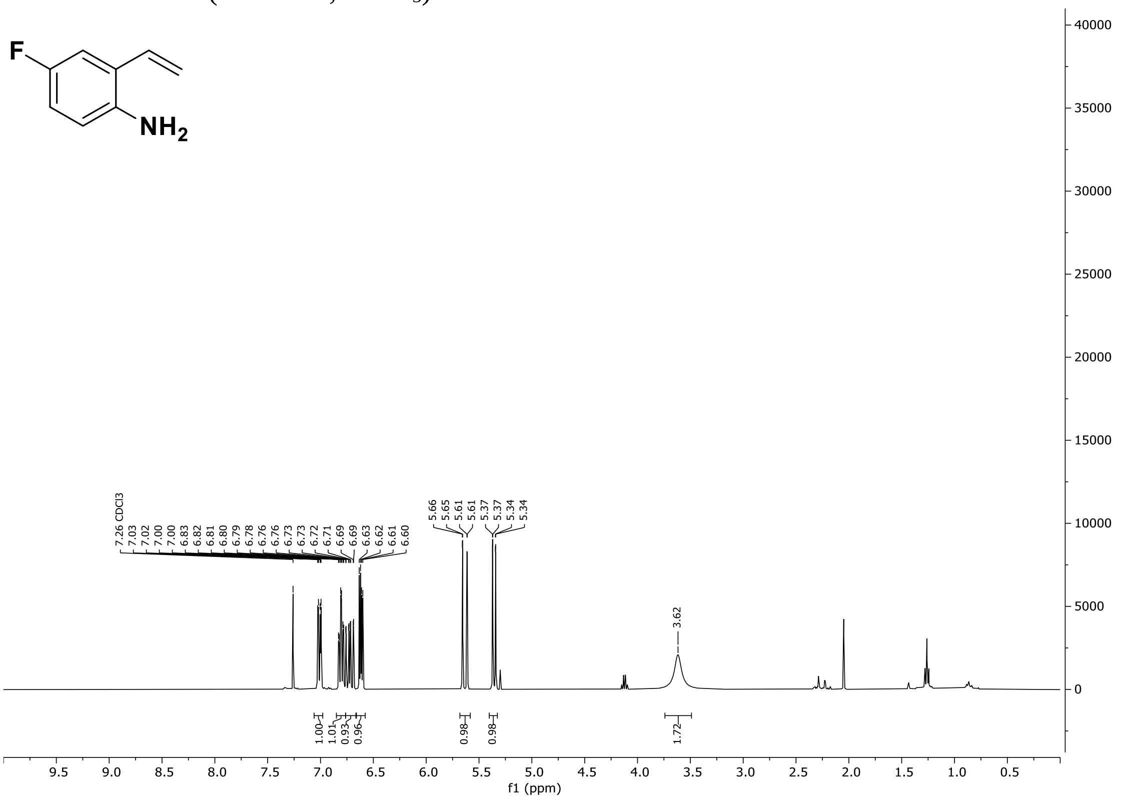

^{13}C-NMR of S13 (100 MHz, $CDCl_3$)

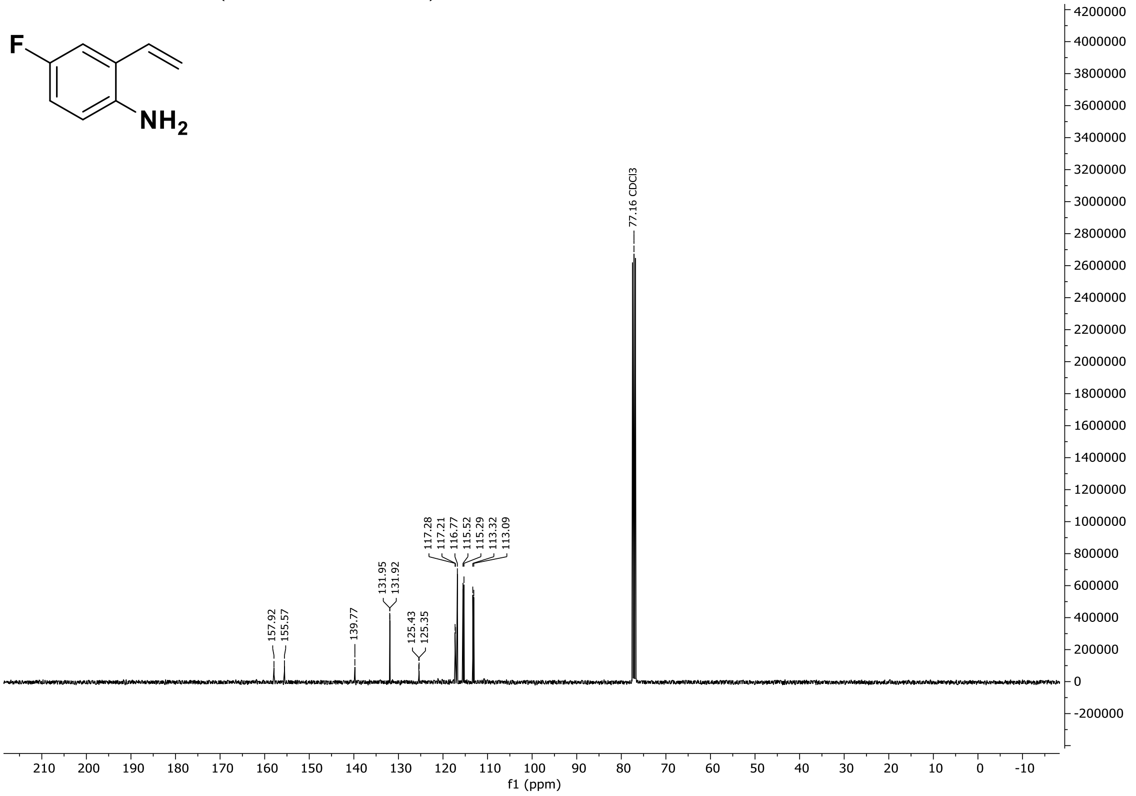

^{19}F-NMR of S13 (376 MHz, $CDCl_3$)

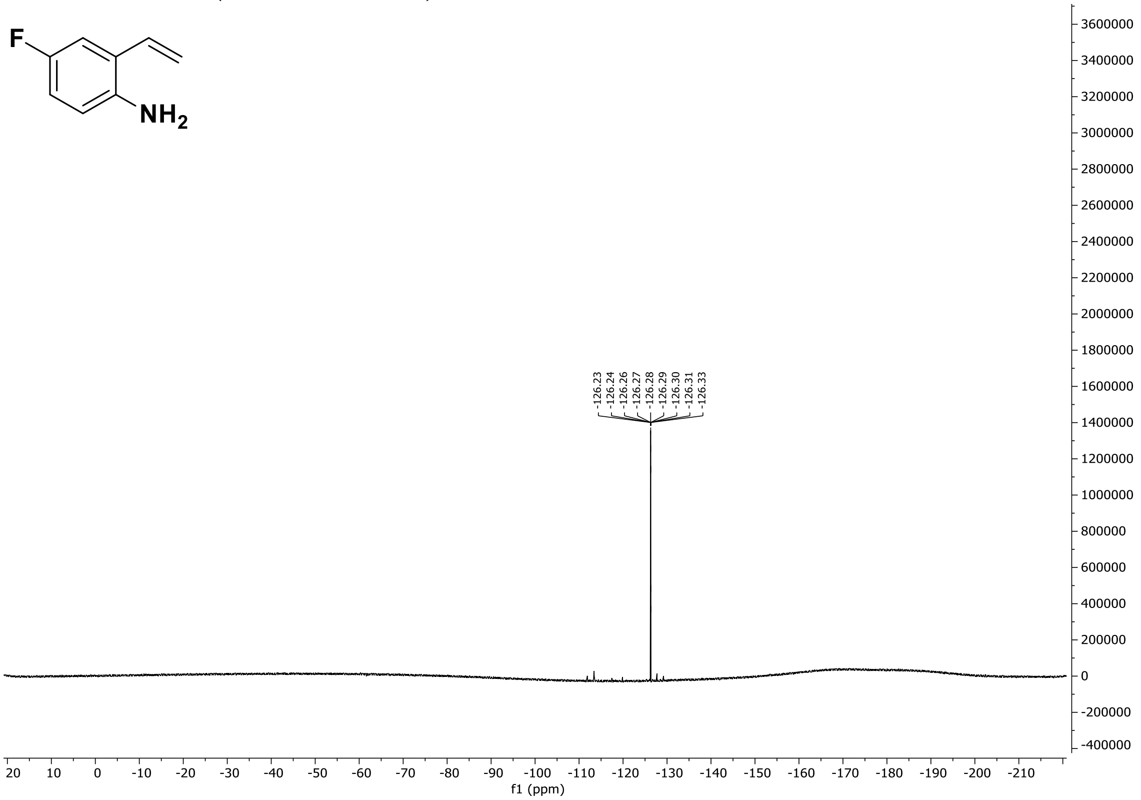

^{1}H-NMR of S14 (400 MHz, $CDCl_3$)

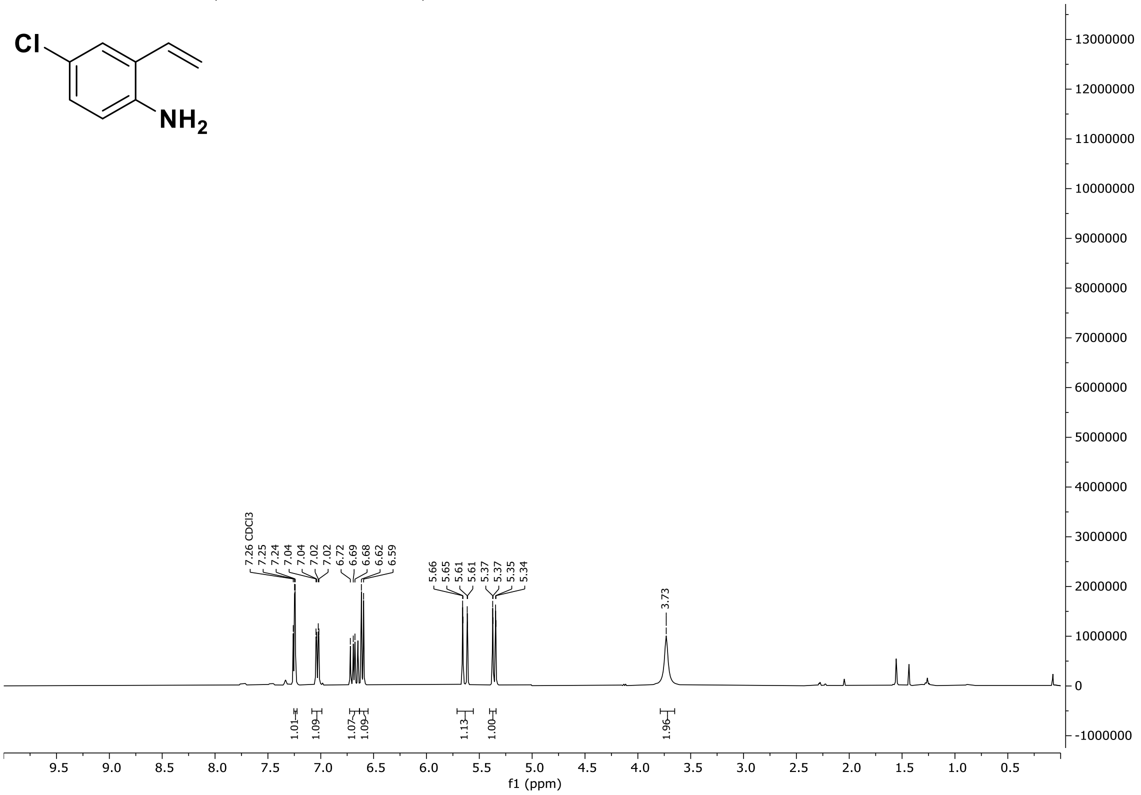

^{1}H-NMR of 1 (400 MHz, $CDCl_3$)

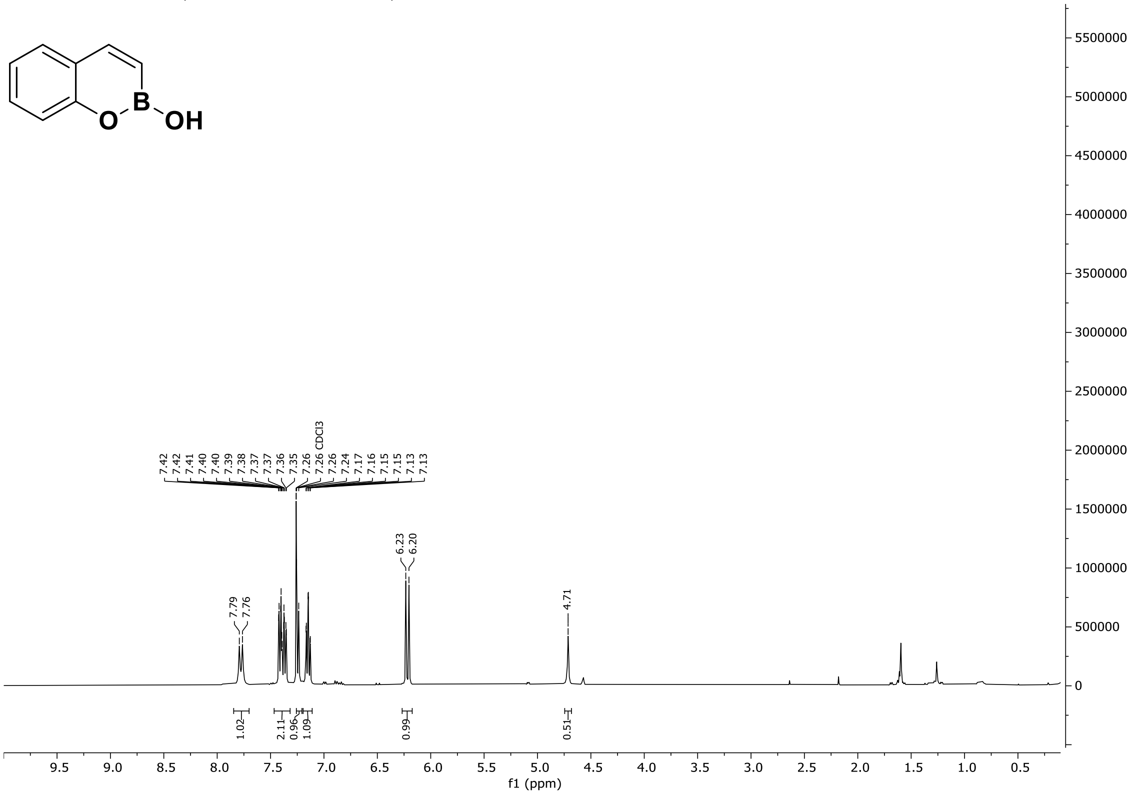

^{1}H-NMR of S15 (400 MHz, $CDCl_3$)

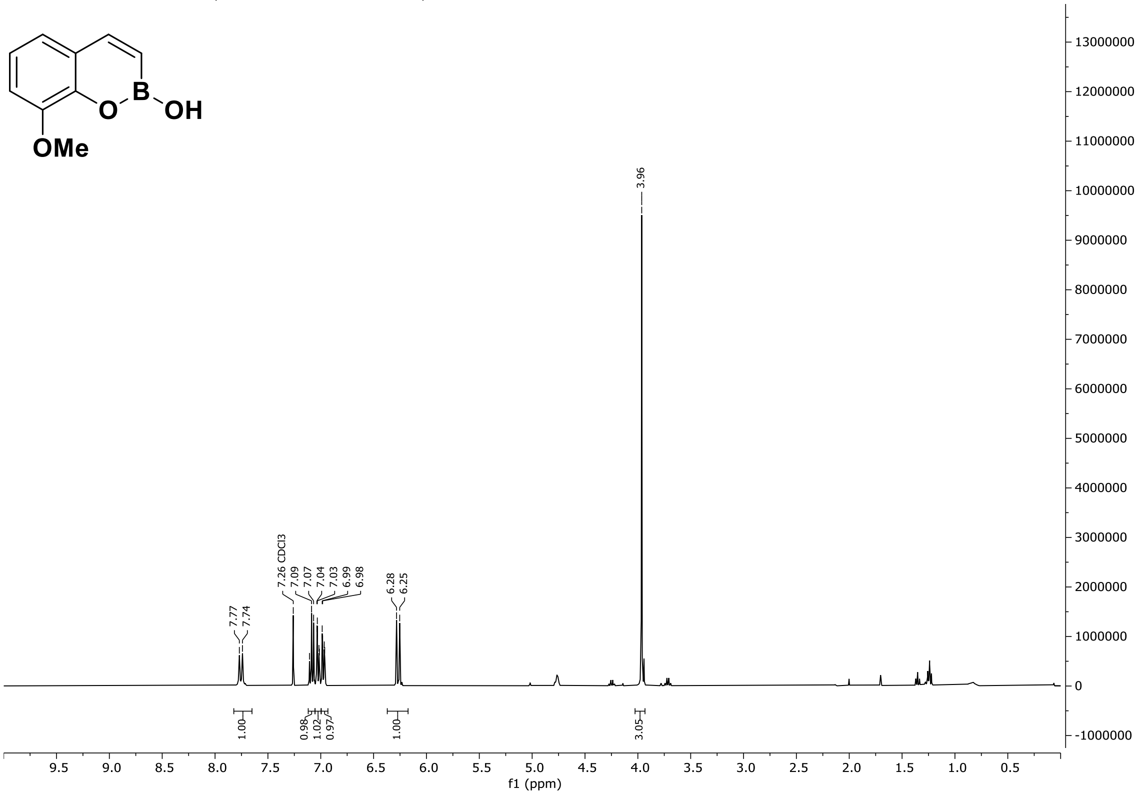

^{13}C-NMR of S15 (100 MHz, $CDCl_3$)

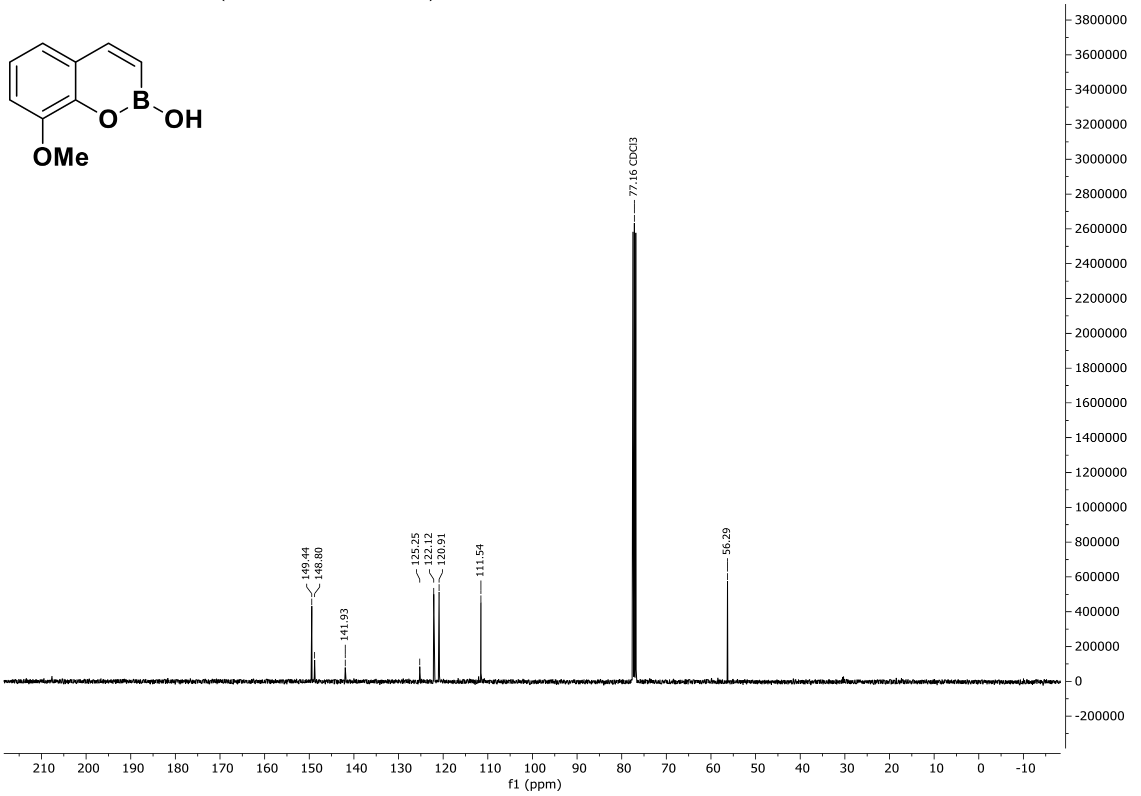

^{11}B-NMR of S15 (128 MHz, $CDCl_3$)

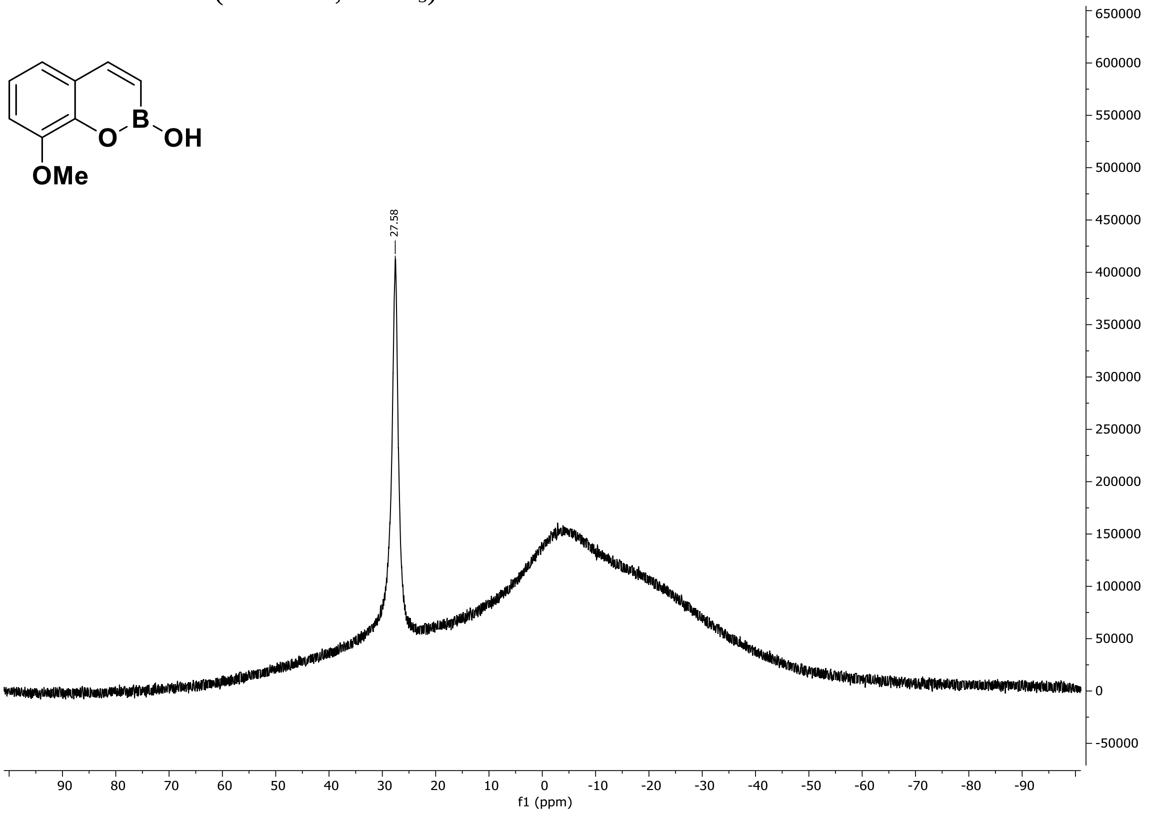

^{1}H-NMR of S16 (400 MHz, $CDCl_3$ with a drop of D_2O)

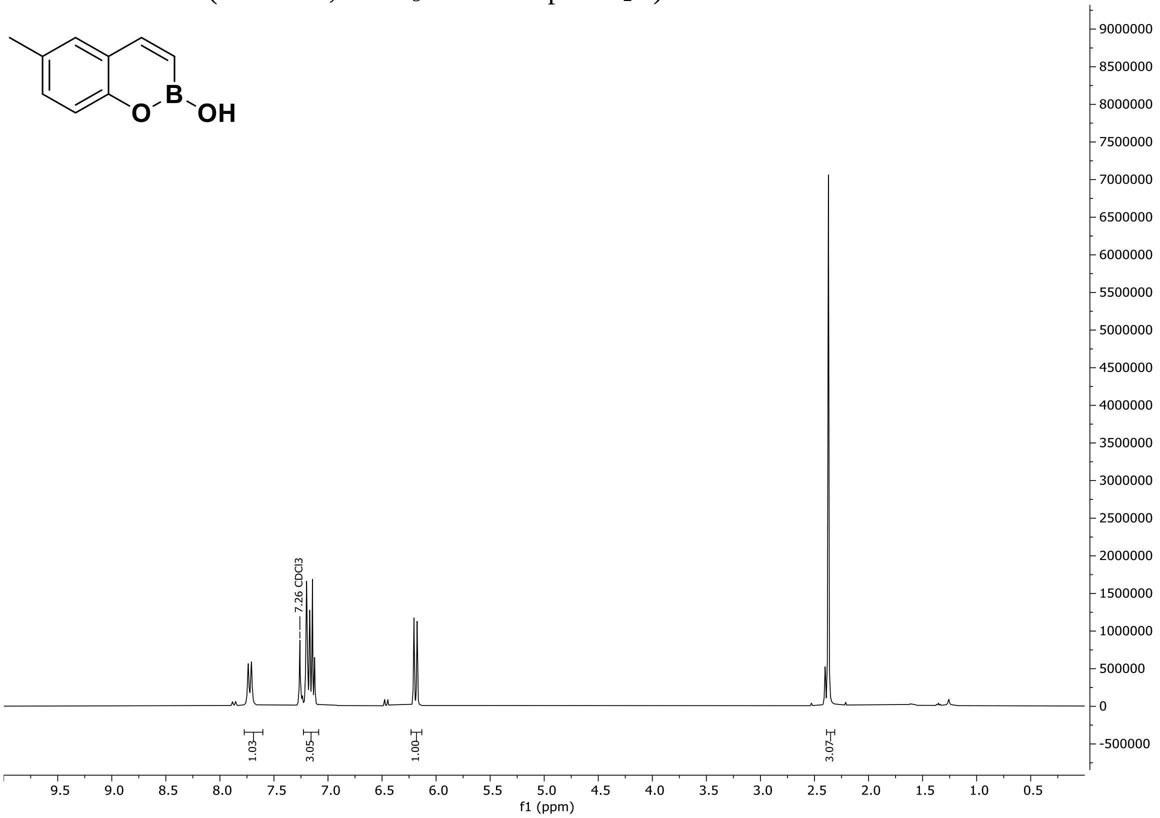

^{13}C-NMR of S16 (100 MHz, $CDCl_3$ with a drop of D_2O)

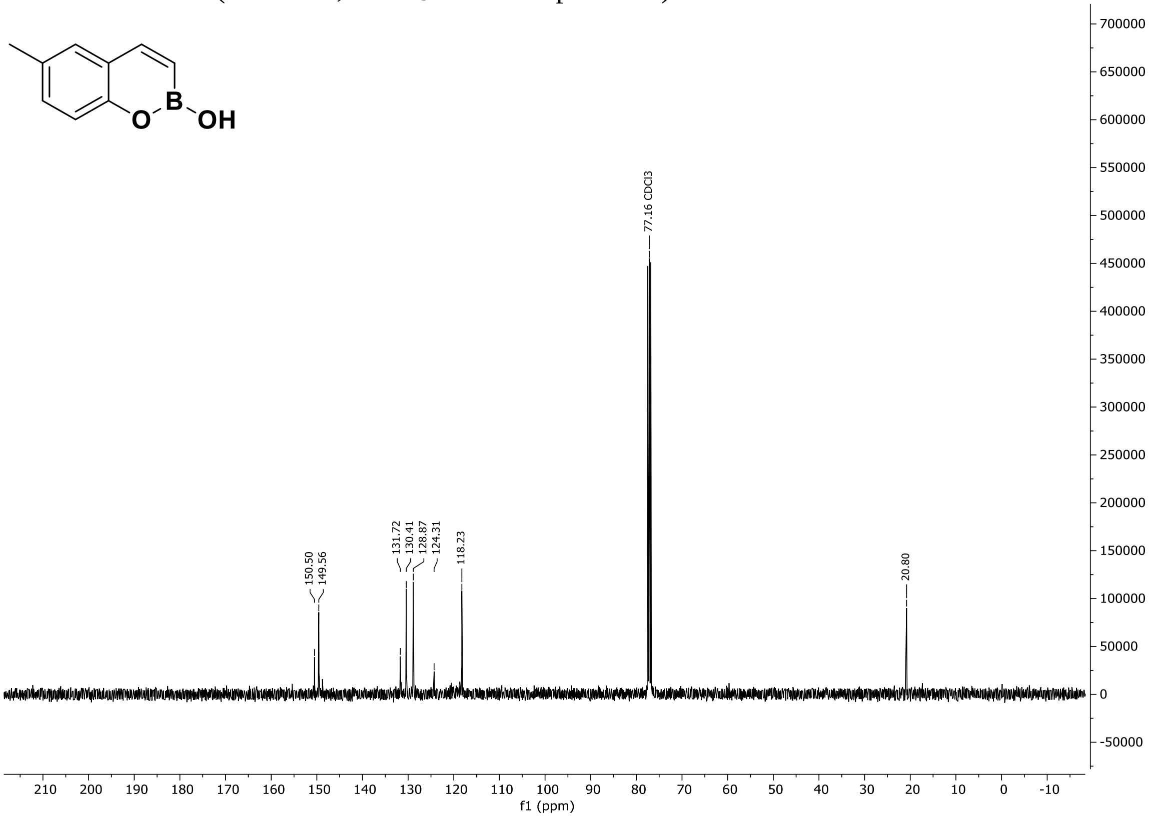

^{11}B-NMR of S16 (128 MHz $CDCl_3$ with a drop of D_2O)

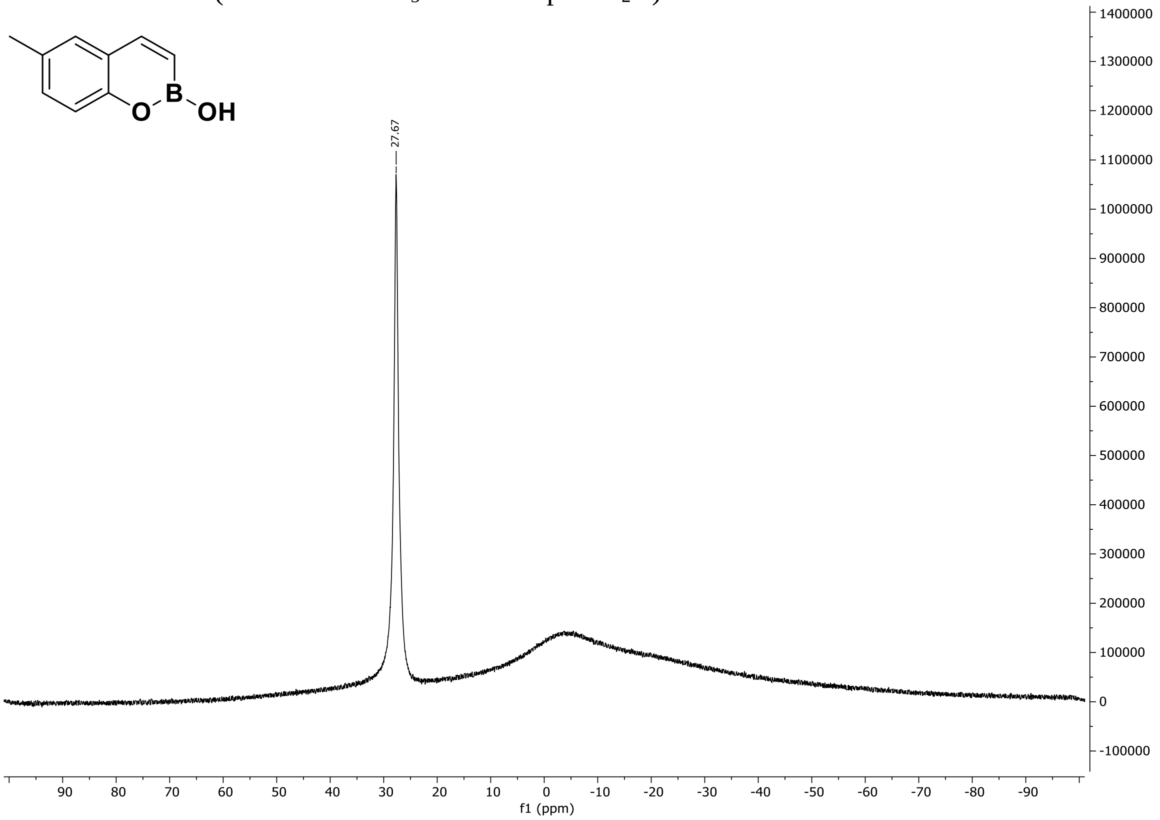

^{1}H-NMR of S17 (400 MHz, $CDCl_3$ with a drop of D_2O)

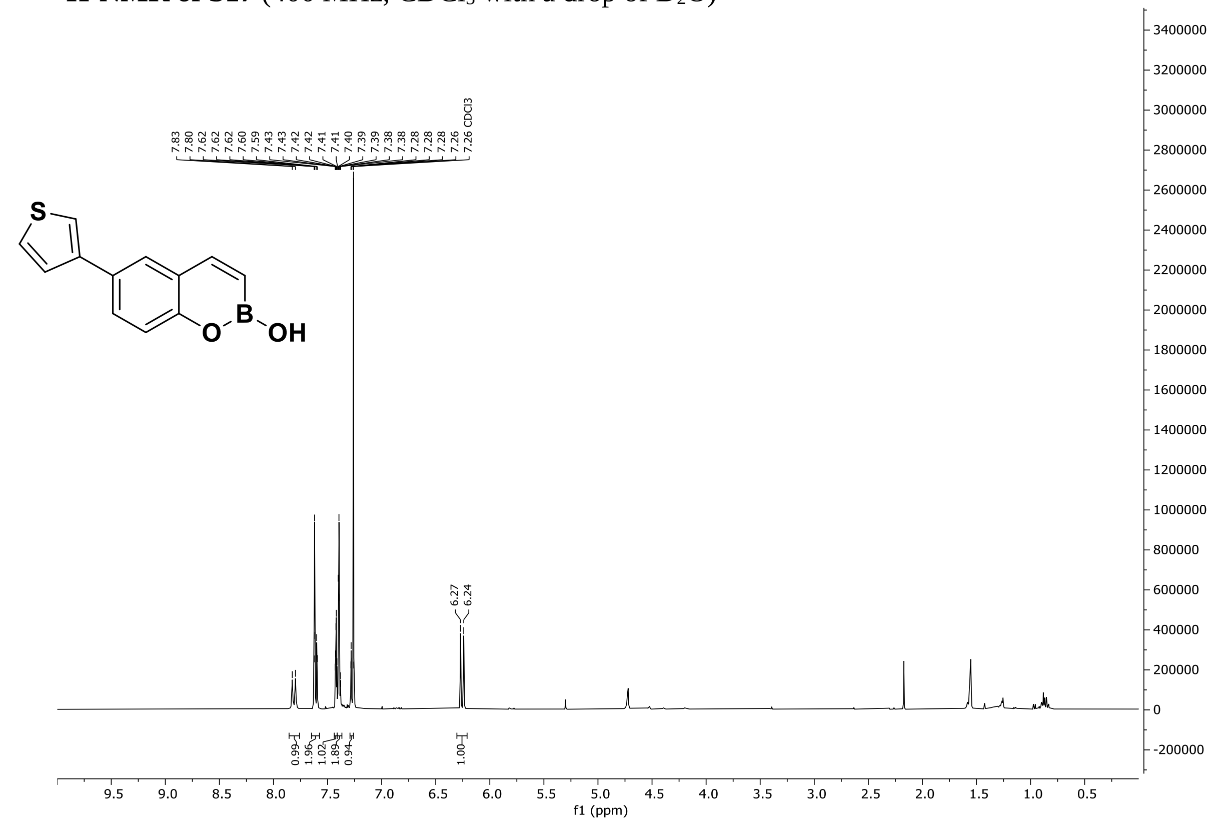

^{13}C-NMR of S17 (100 MHz, $CDCl_3$ with a drop of D_2O)

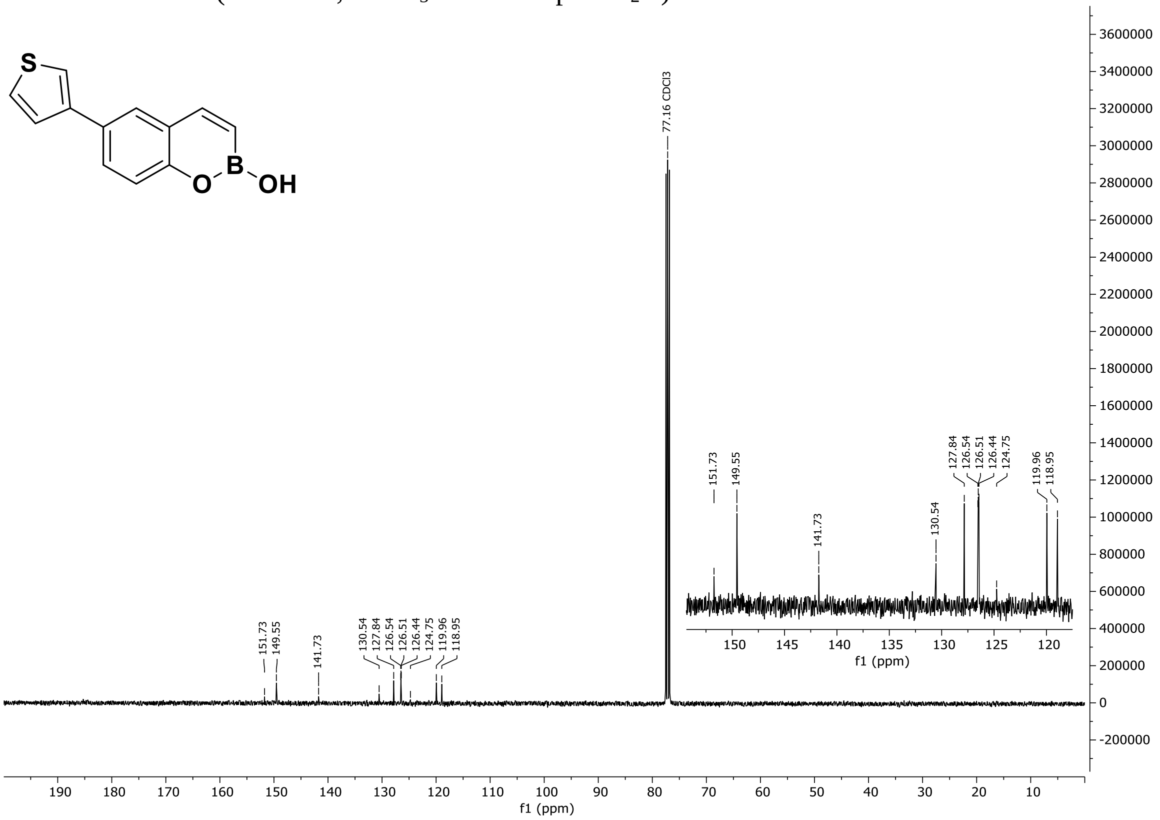

^{11}B-NMR of S17 (128 MHz $CDCl_3$ with a drop of D_2O)

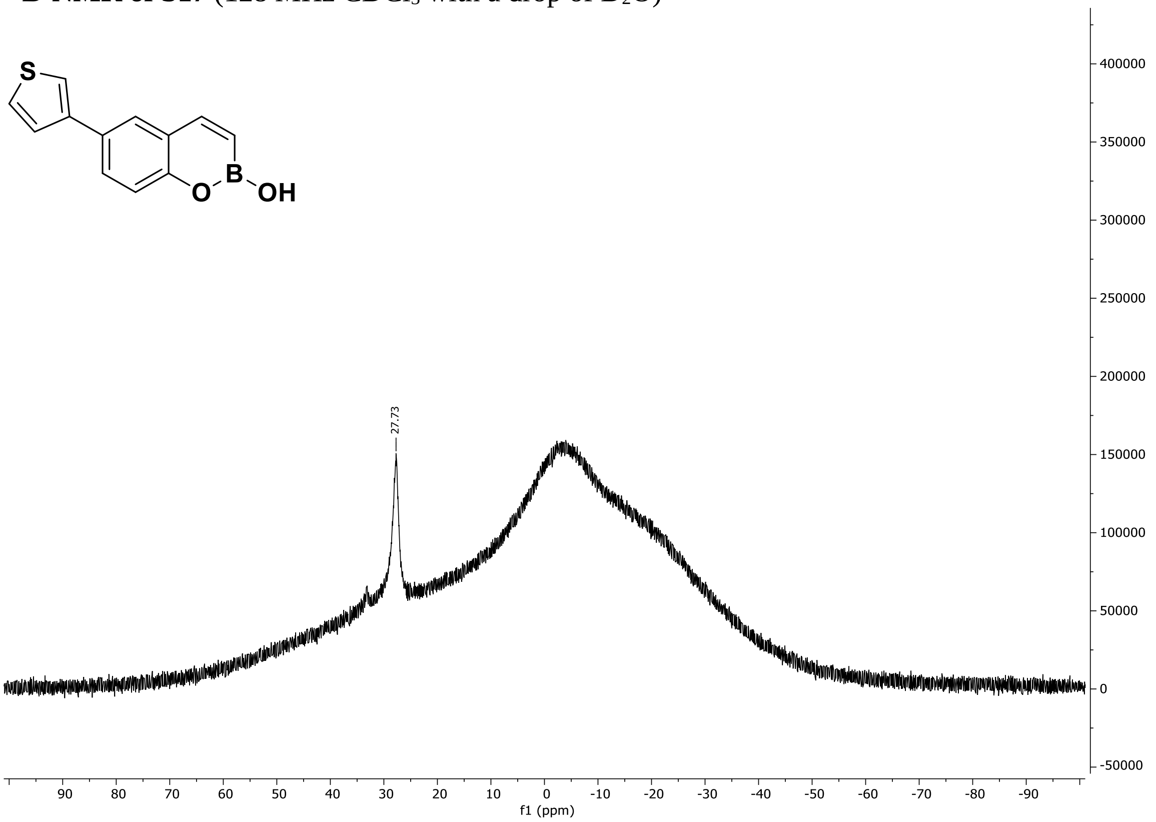

^{1}H-NMR of S18 (400 MHz, $CDCl_3$)

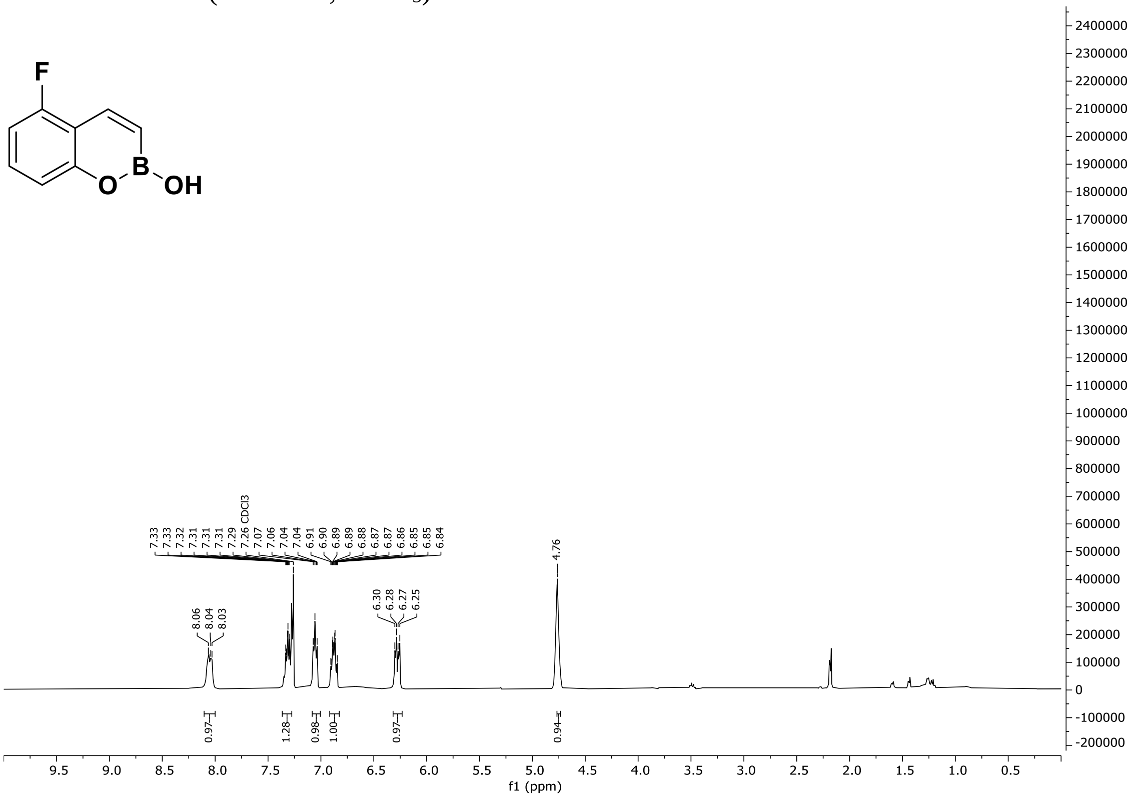

^{13}C-NMR of S18 (100 MHz, $CDCl_3$)

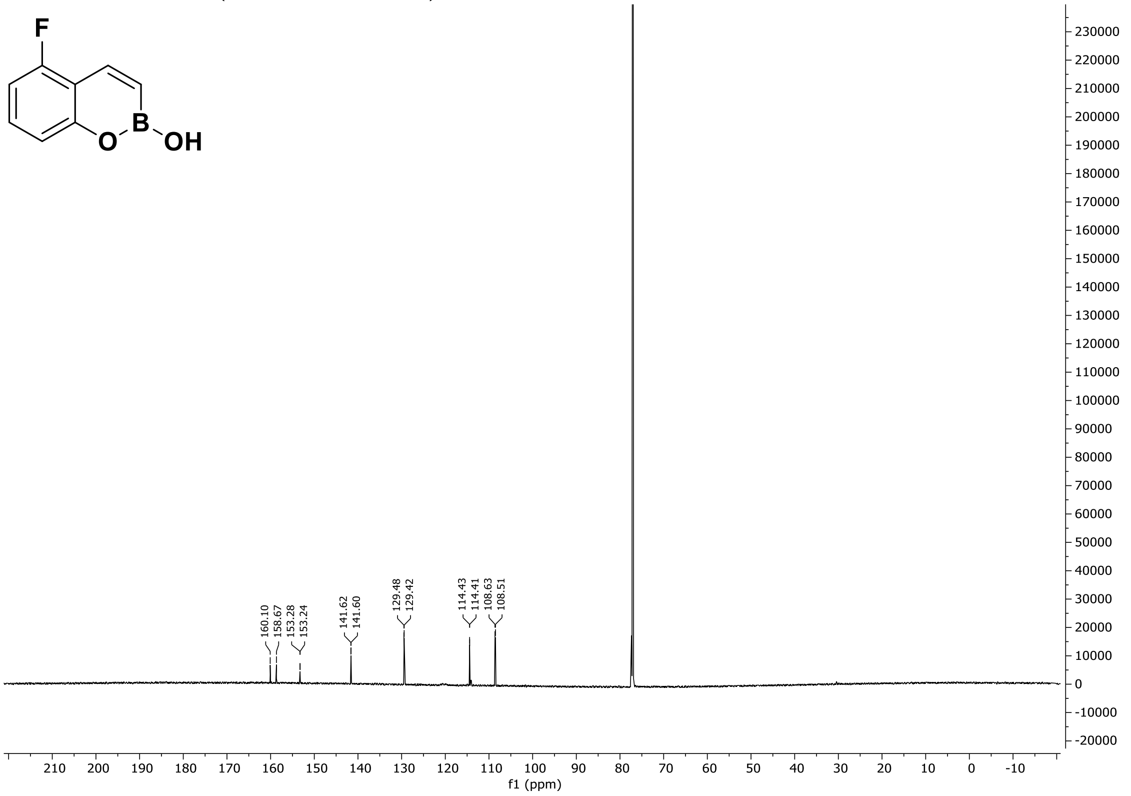

^{11}B-NMR of S18 (128 MHz, $CDCl_3$)

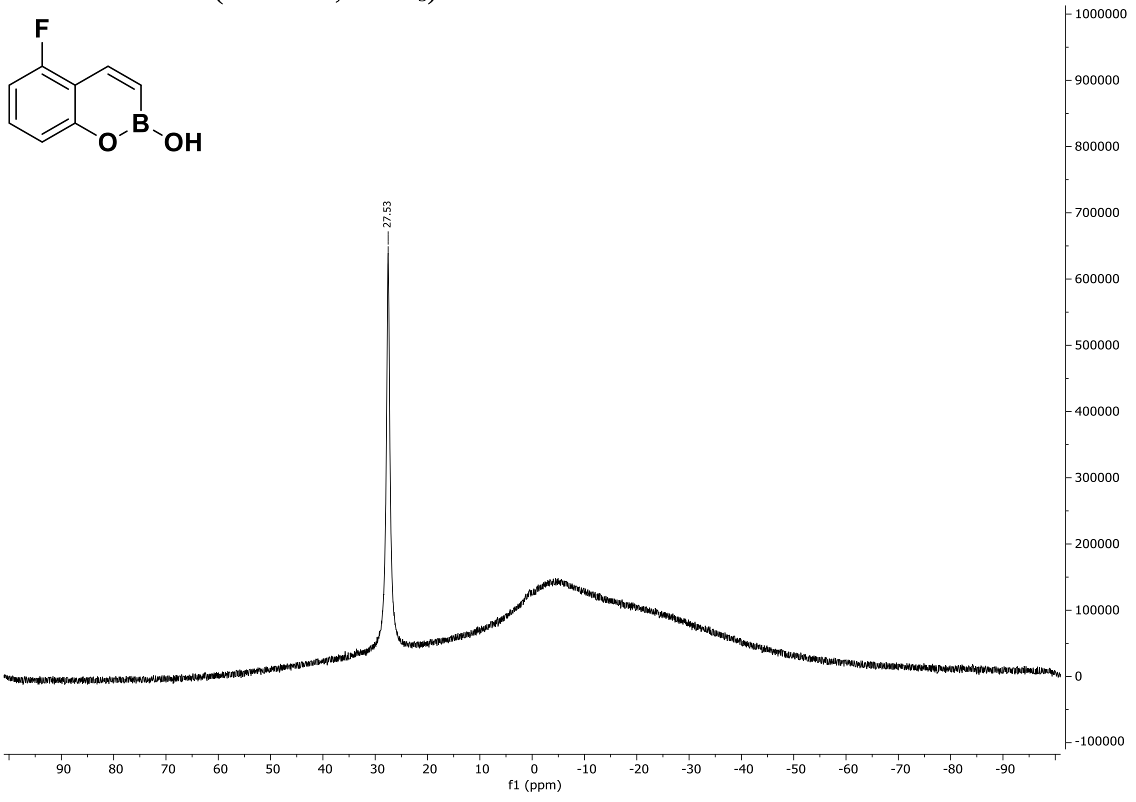

^{19}F-NMR of S18 (376 MHz, $CDCl_3$)

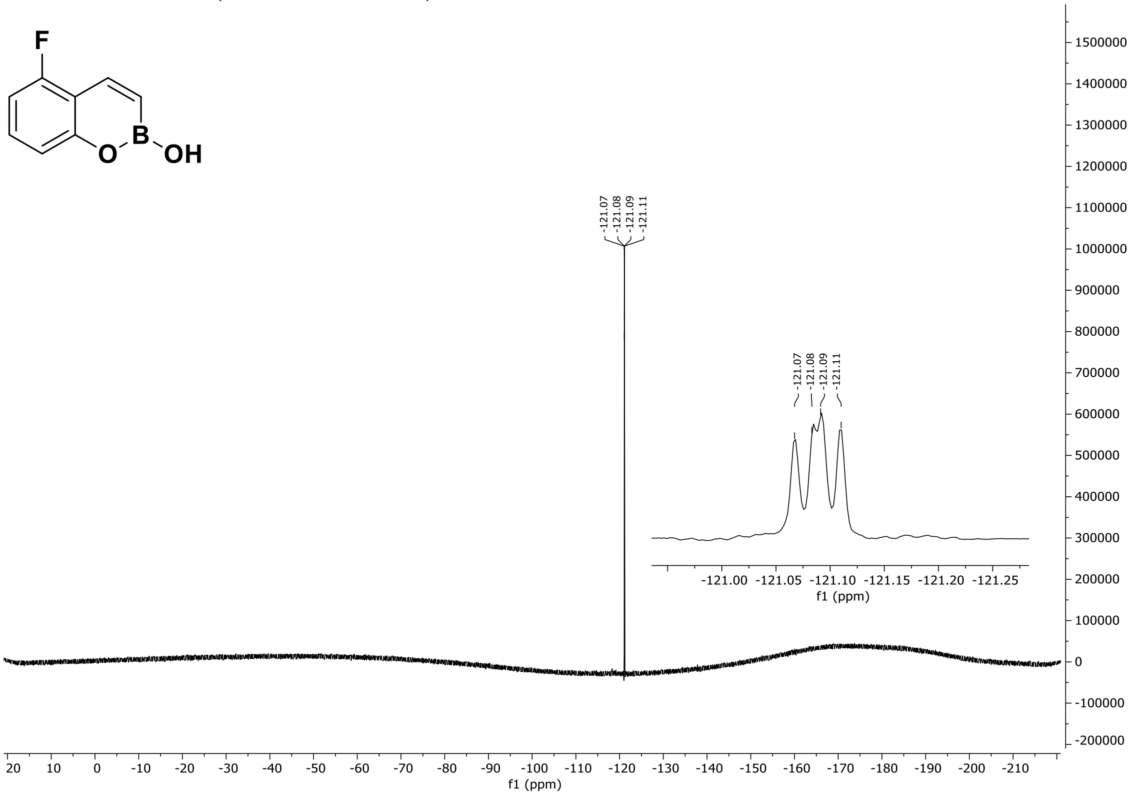

^{1}H-NMR of S19 (400 MHz, $CDCl_3$)

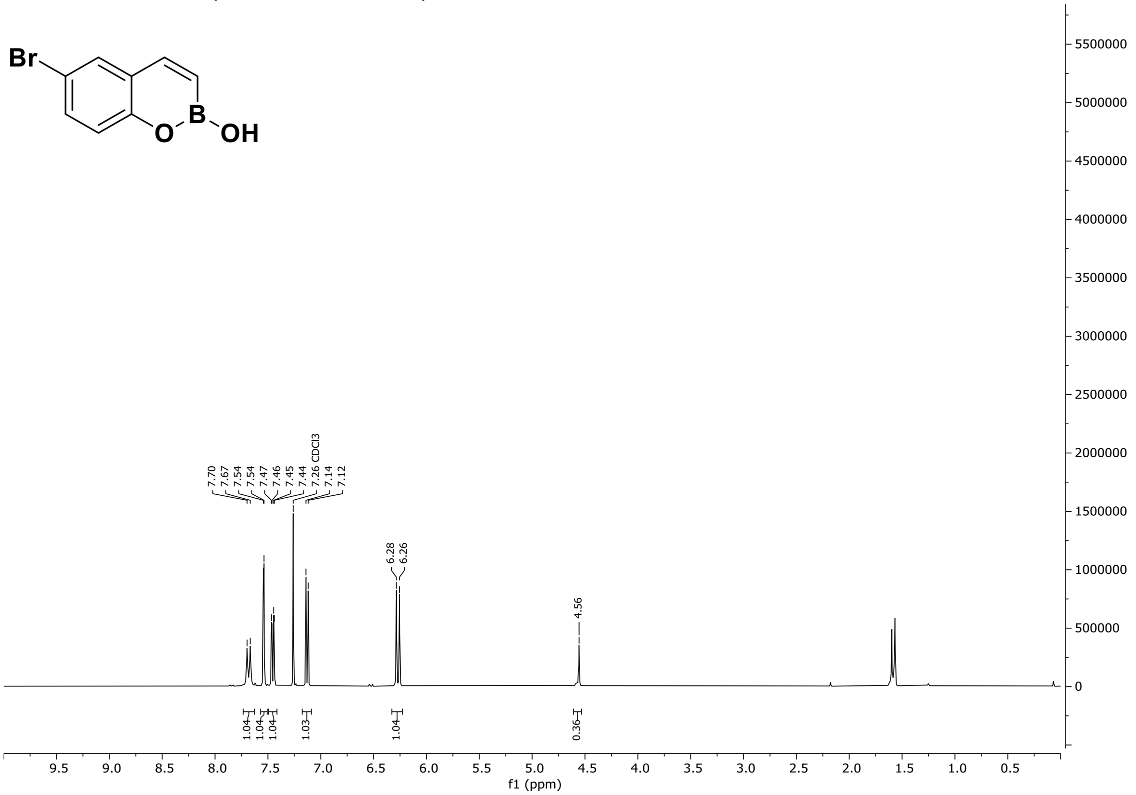

^{13}C-NMR of S19 (100 MHz, $CDCl_3$)

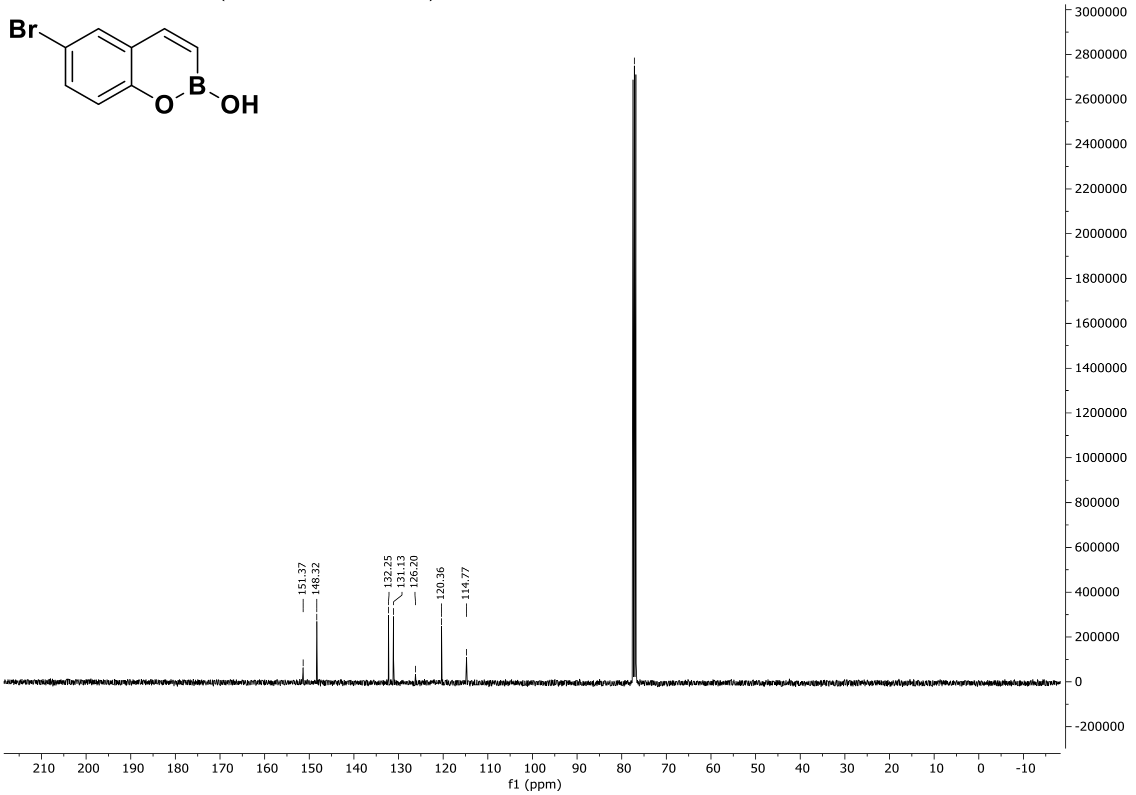

^{11}B-NMR of S19 (128 MHz, $CDCl_3$)

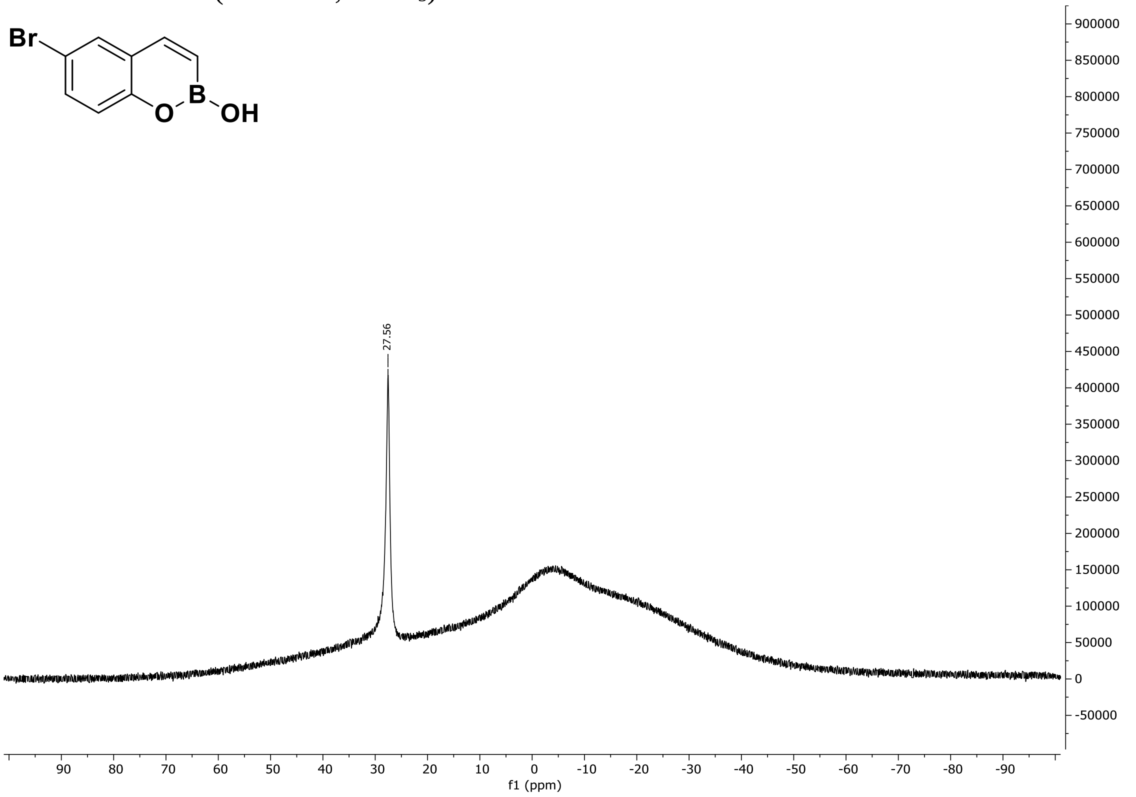

^{1}H-NMR of S20 (400 MHz, $CDCl_3$)

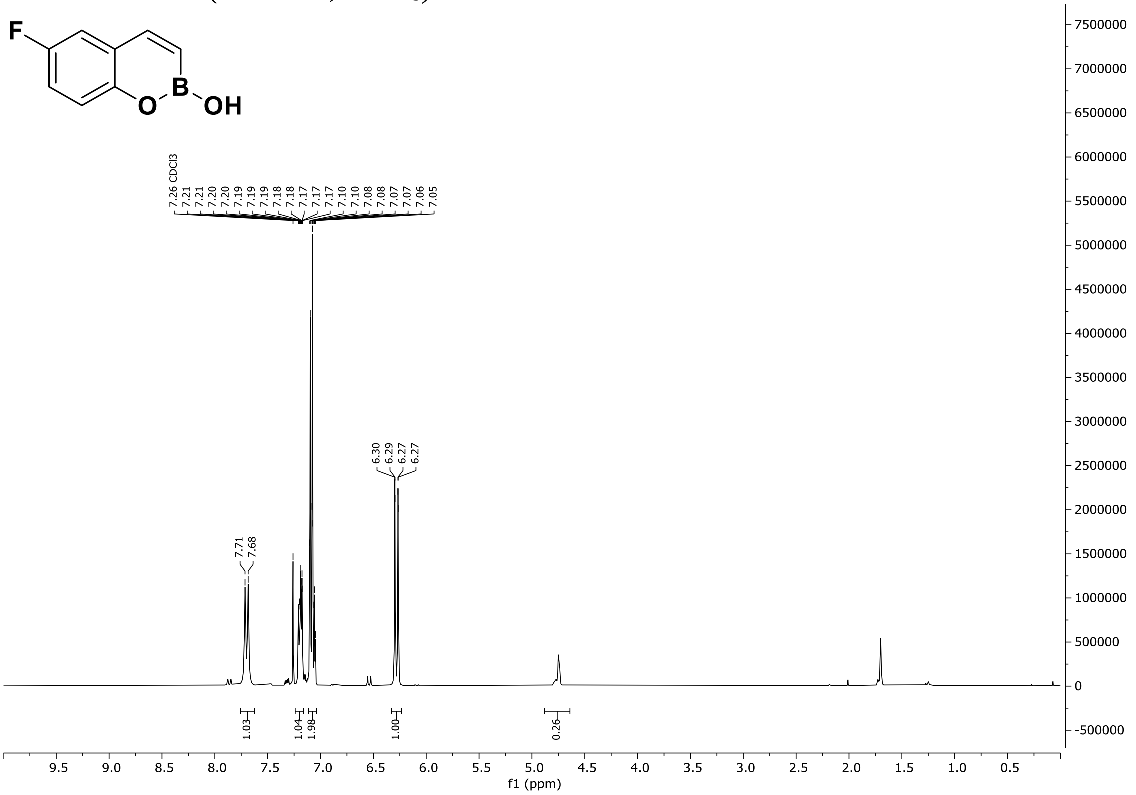

^{13}C-NMR of S20 (100 MHz, $CDCl_3$)

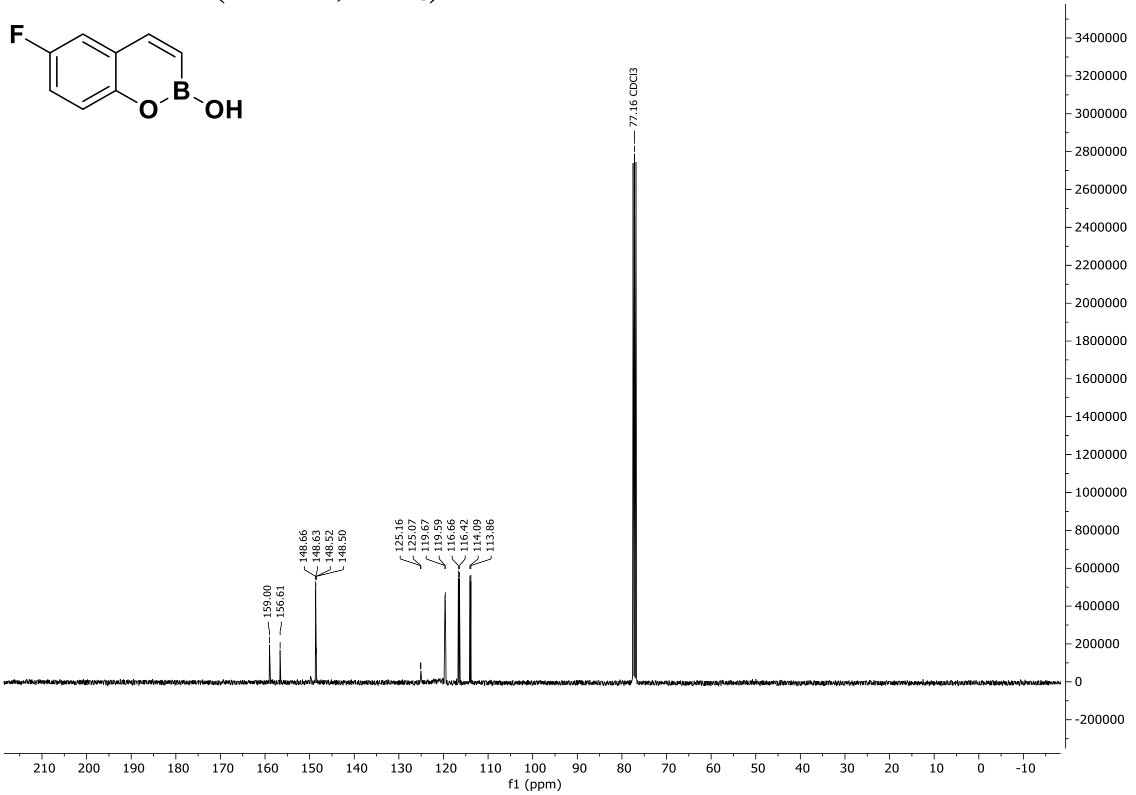

^{11}B-NMR of S20 (128 MHz, $CDCl_3$)

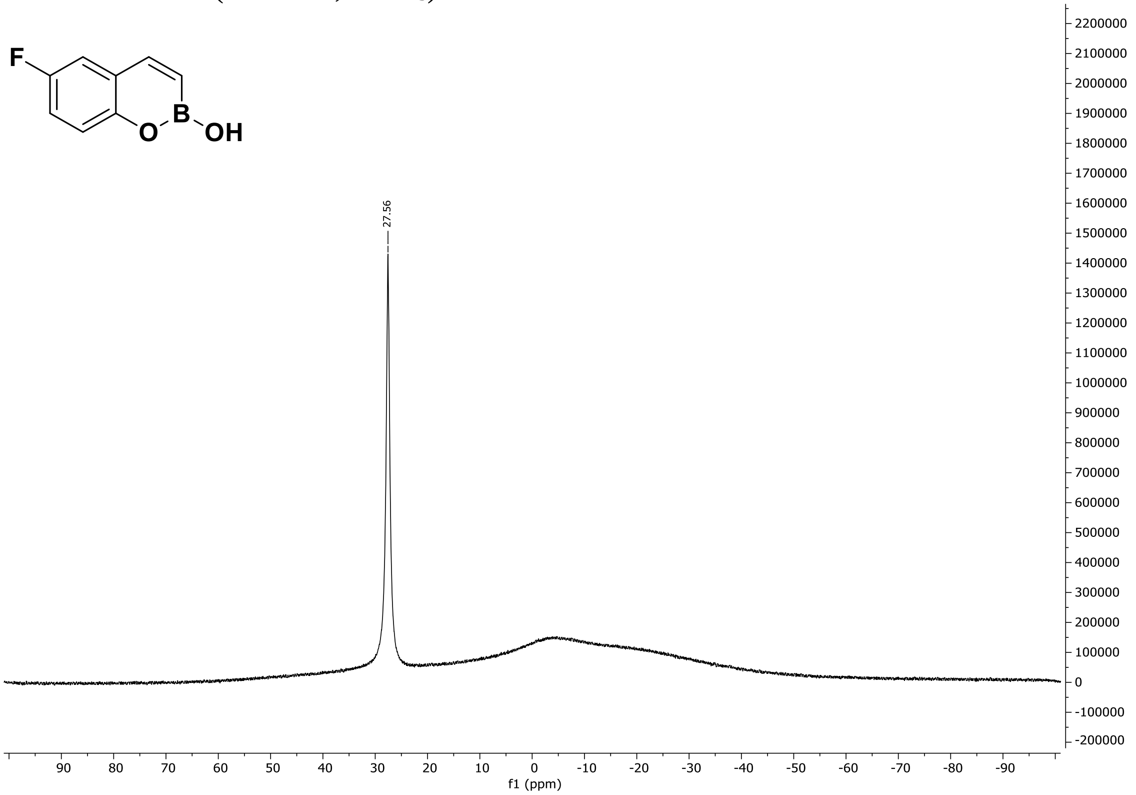

^{19}F-NMR of S20 (376 MHz, $CDCl_3$)

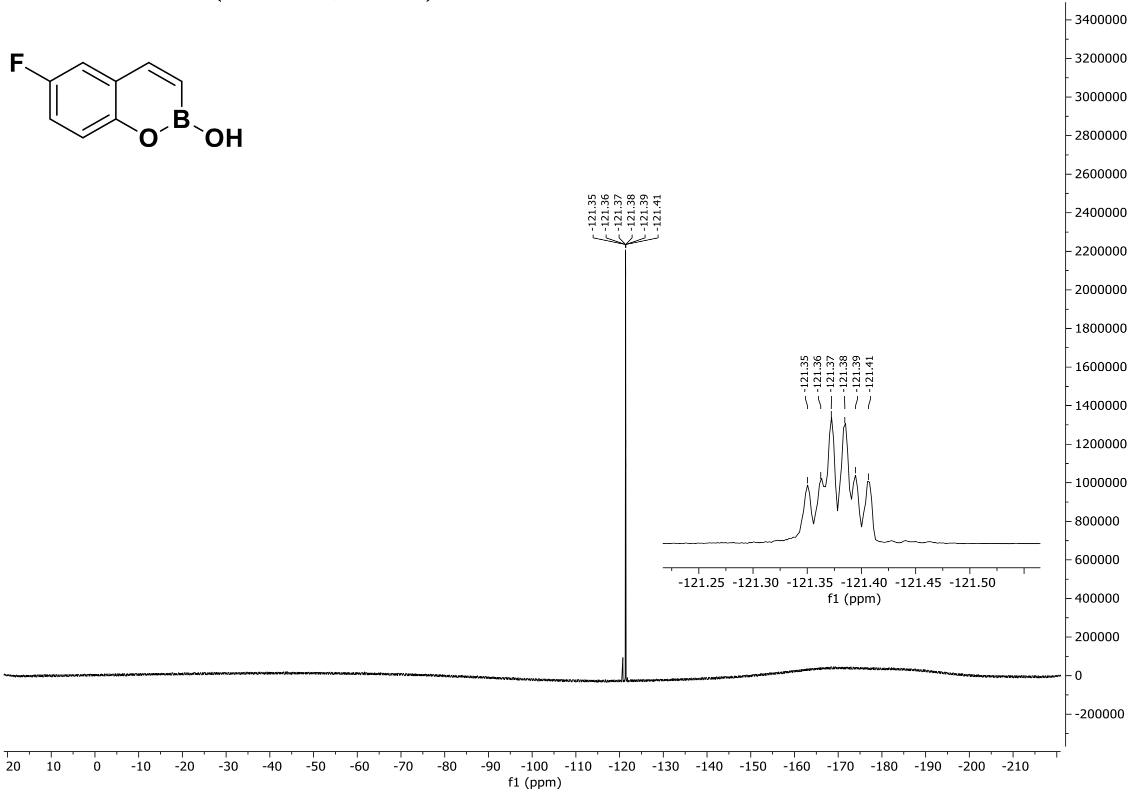

^{1}H-NMR of S21 (400 MHz, $CDCl_3$ with a drop of D_2O)

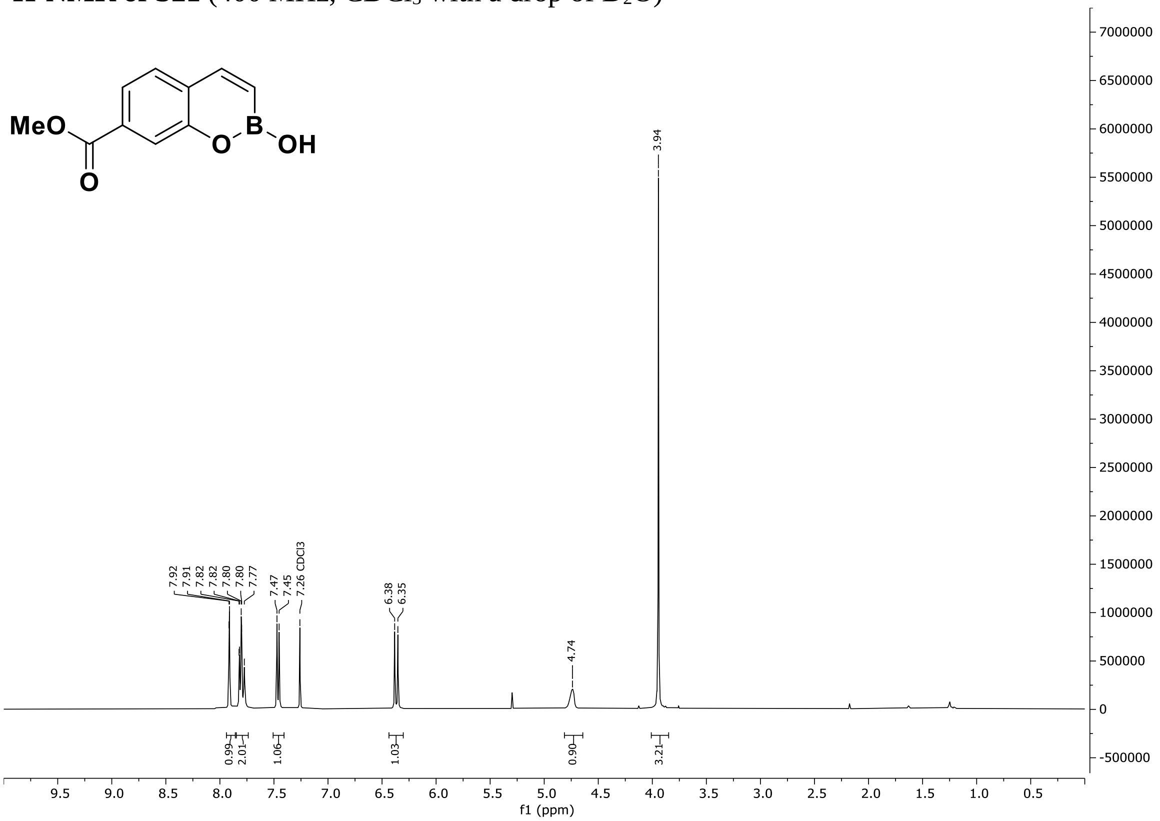

^{13}C-NMR of S21 (100 MHz, $CDCl_3$ with a drop of D_2O)

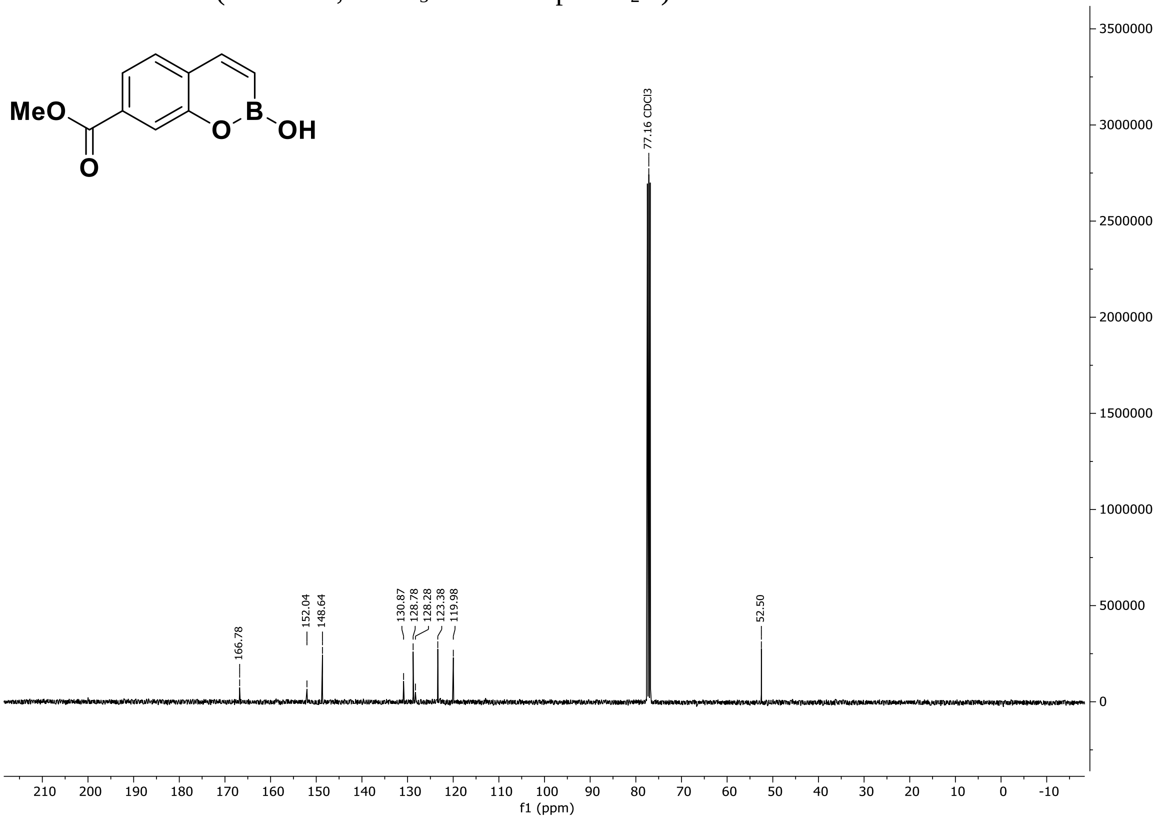

^{11}B-NMR of S21 (128 MHz $CDCl_3$ with a drop of D_2O)

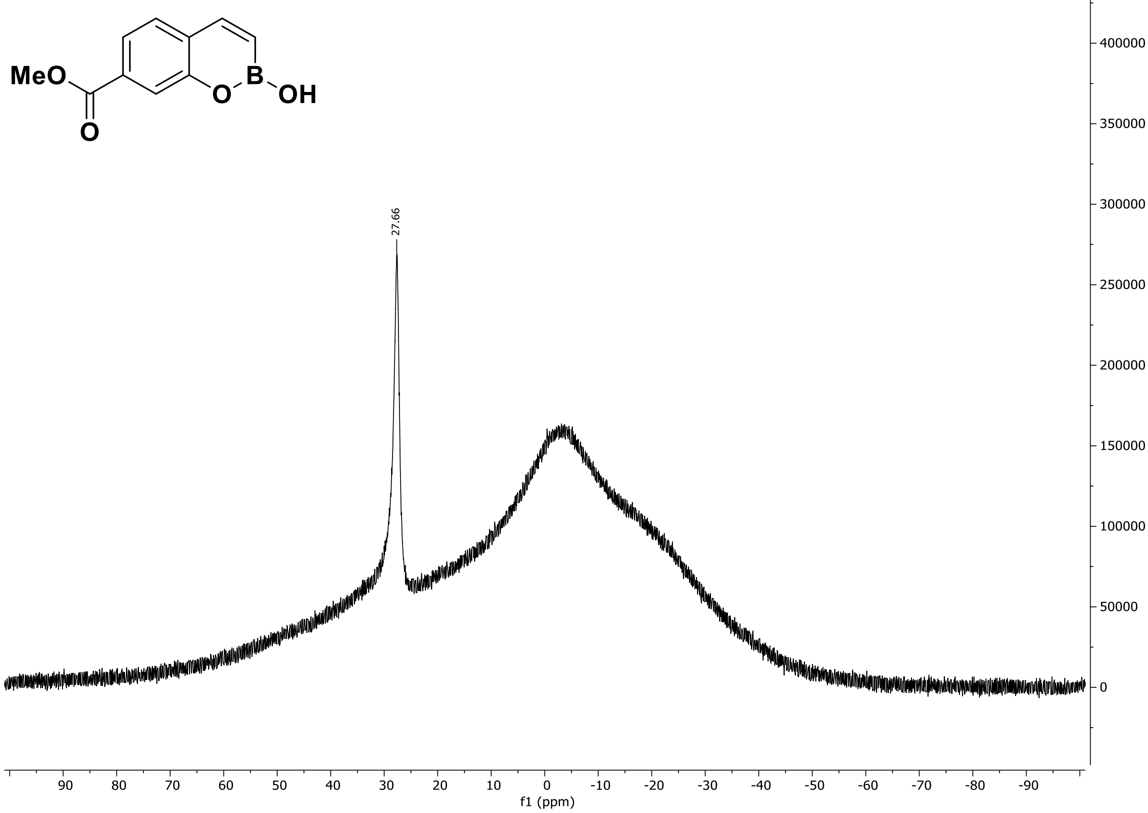

^{1}H-NMR of S22 (400 MHz, $CDCl_3$)

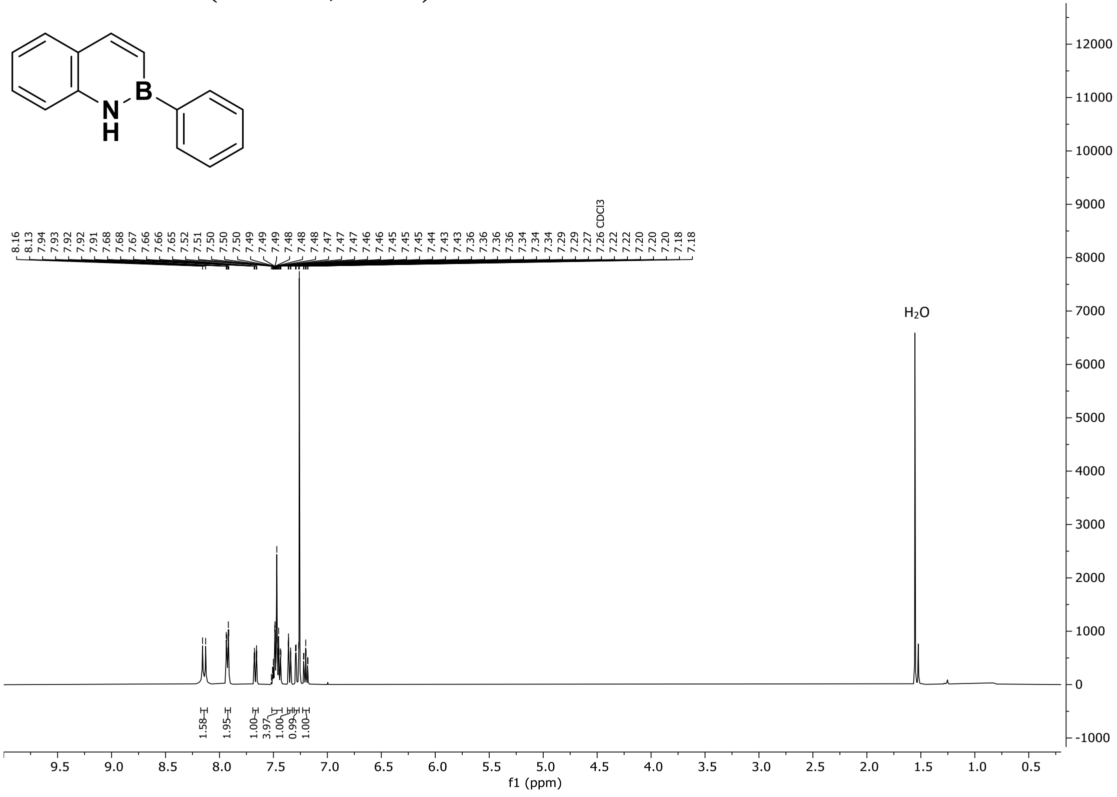

^{1}H-NMR of S23 (400 MHz, $CDCl_3$)

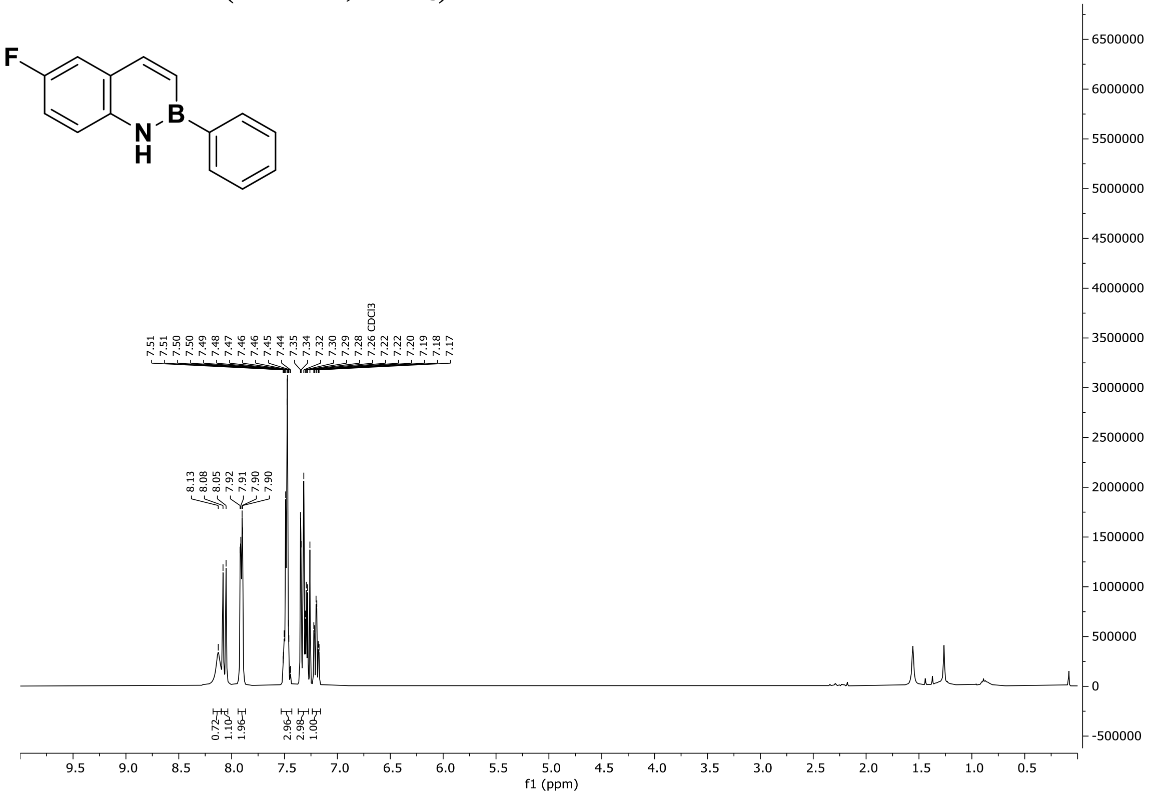

^{13}C-NMR of S23 (100 MHz, $CDCl_3$)

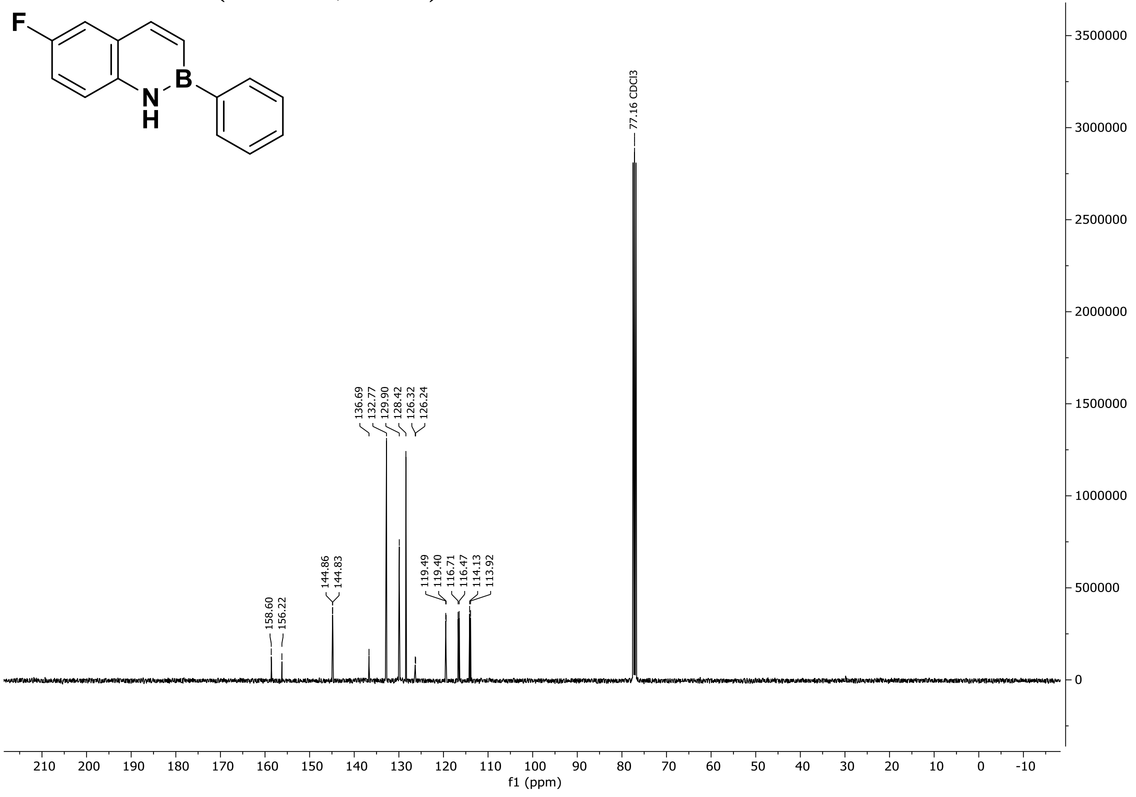

^{11}B-NMR of S23 (128 MHz, $CDCl_3$)

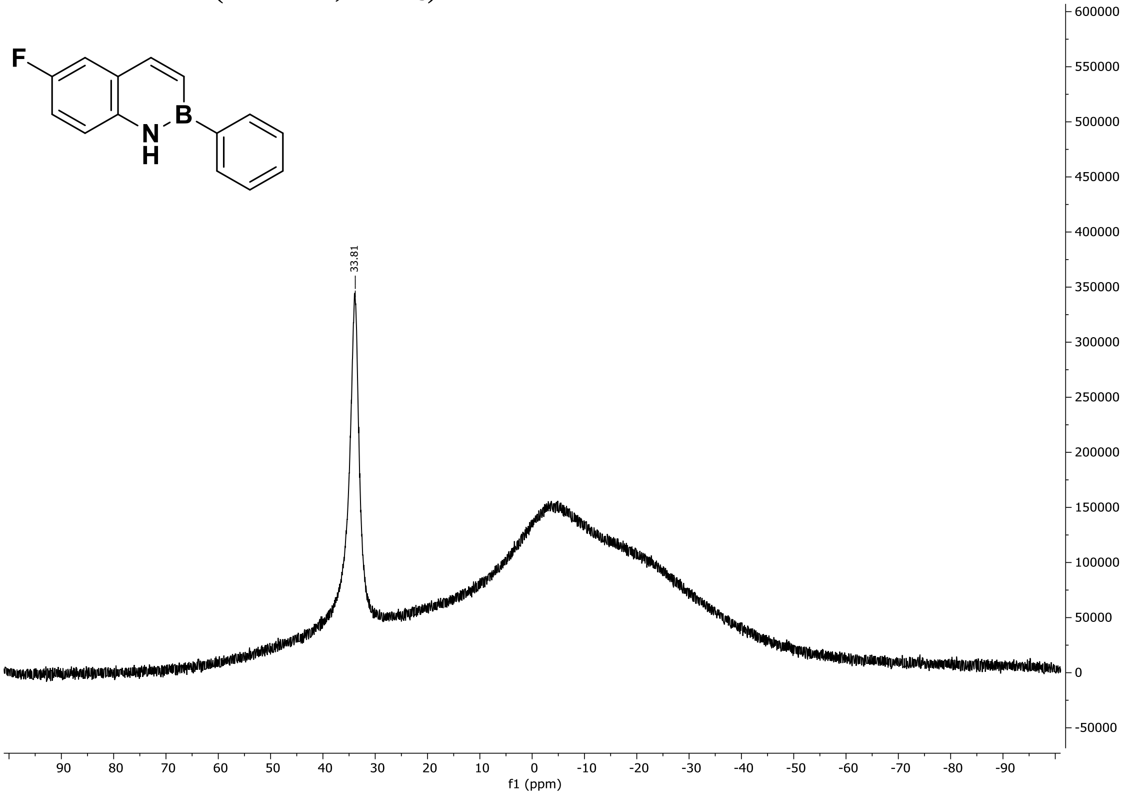

^{19}F-NMR of S23 (376 MHz, $CDCl_3$)

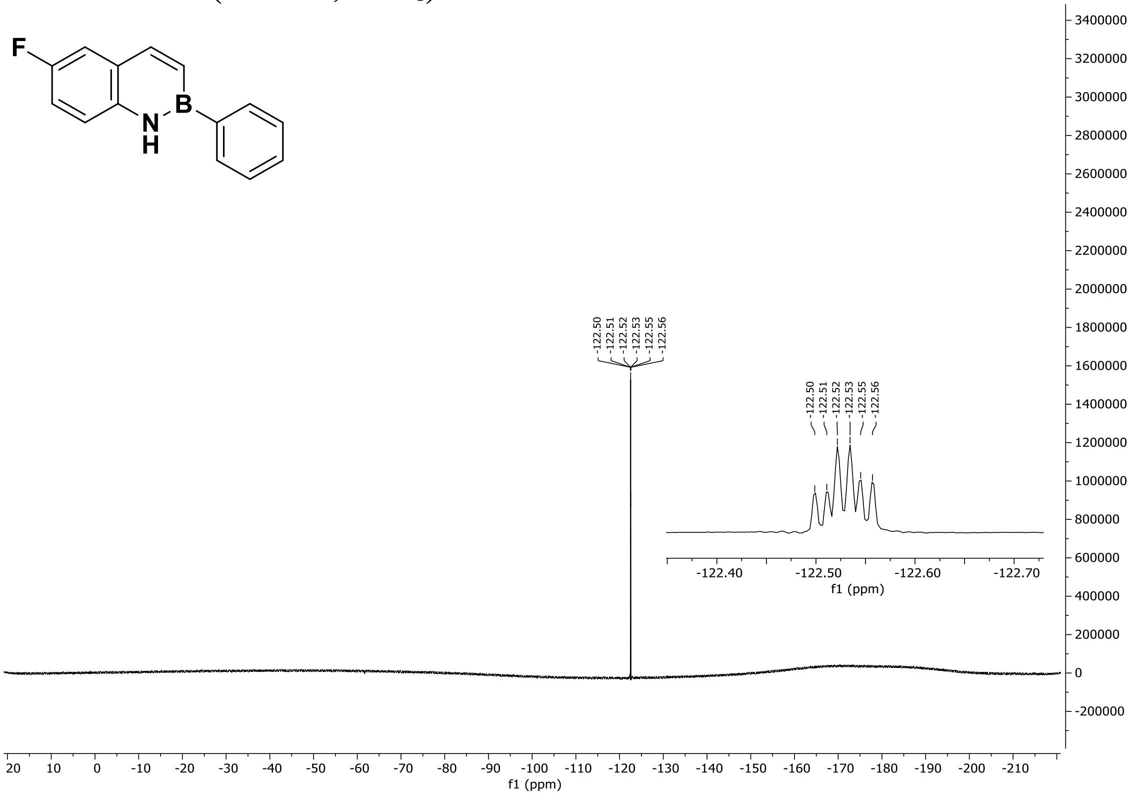

^{1}H-NMR of S24 (400 MHz, $CDCl_3$)

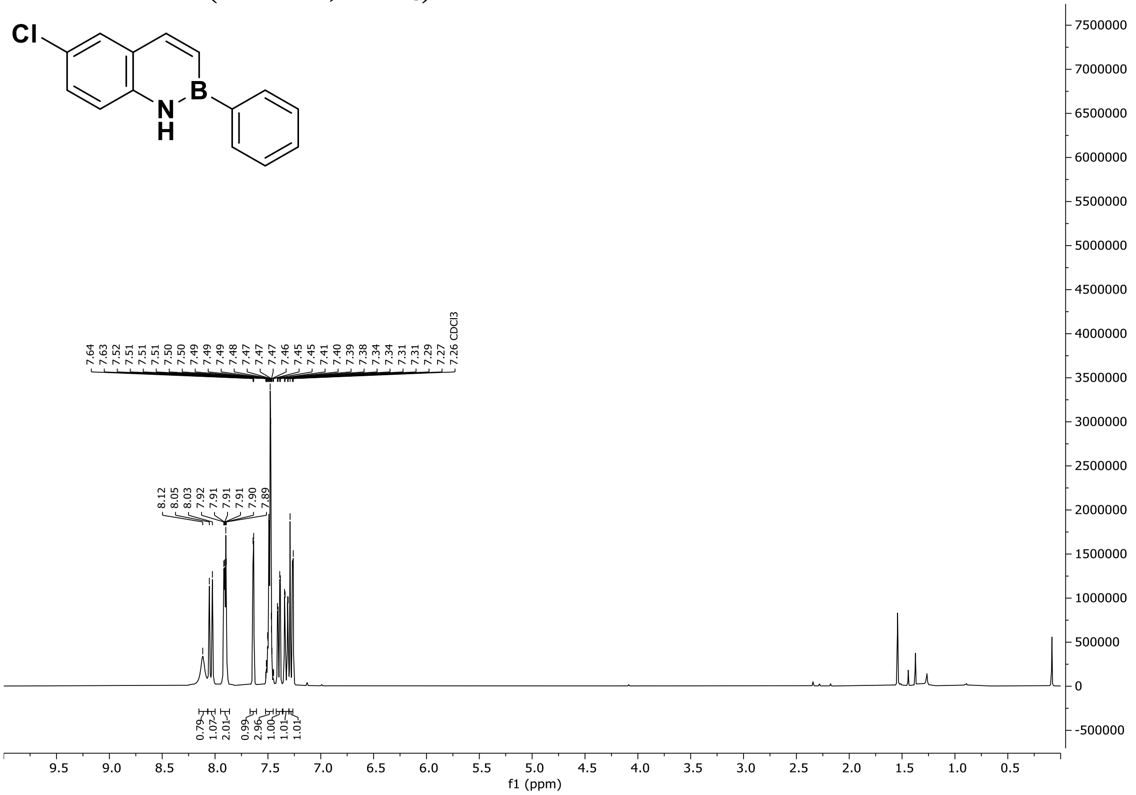

^{13}C-NMR of S24 (100 MHz, $CDCl_3$)

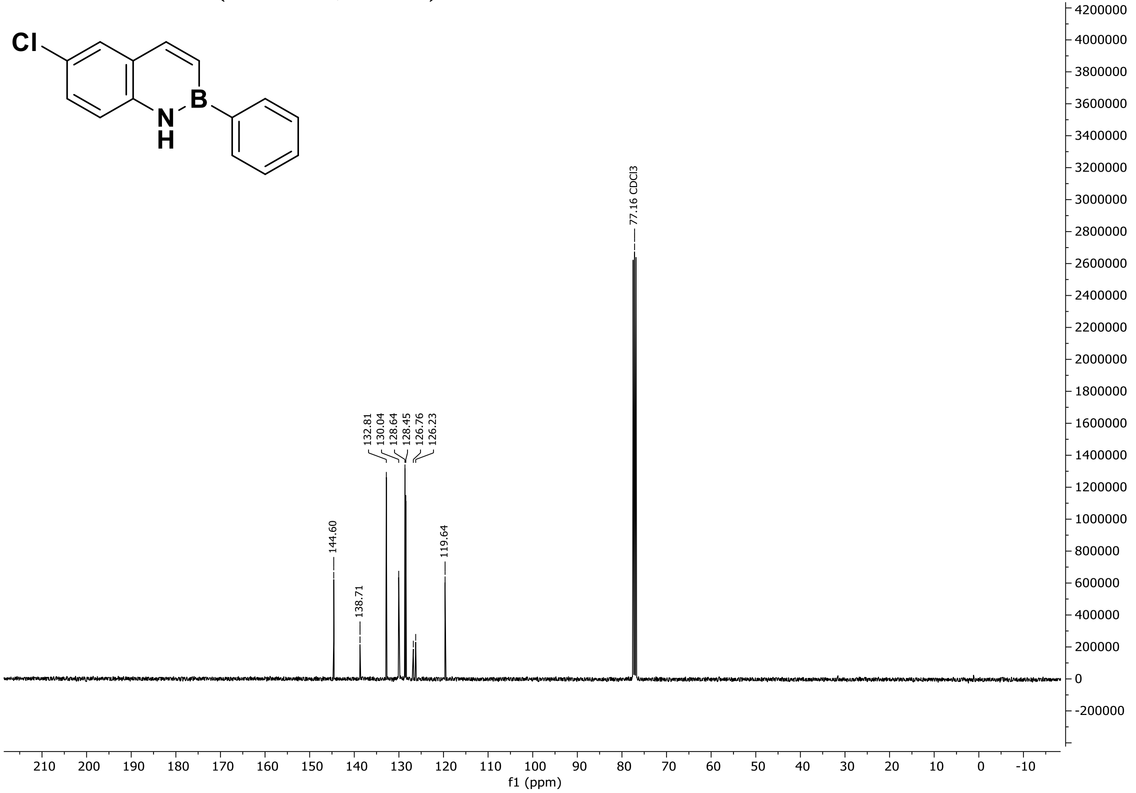

^{11}B-NMR of S24 (128 MHz, $CDCl_3$)

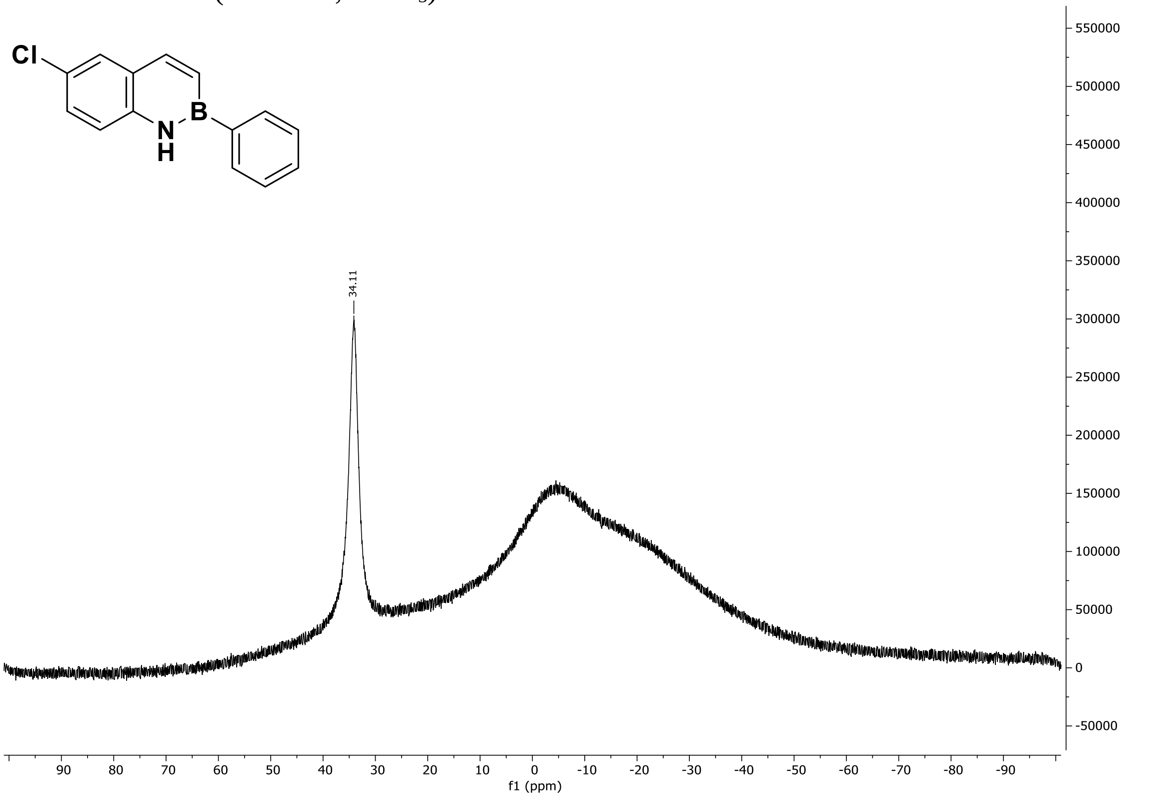

^{1}H-NMR of S25 (400 MHz, $CDCl_3$)

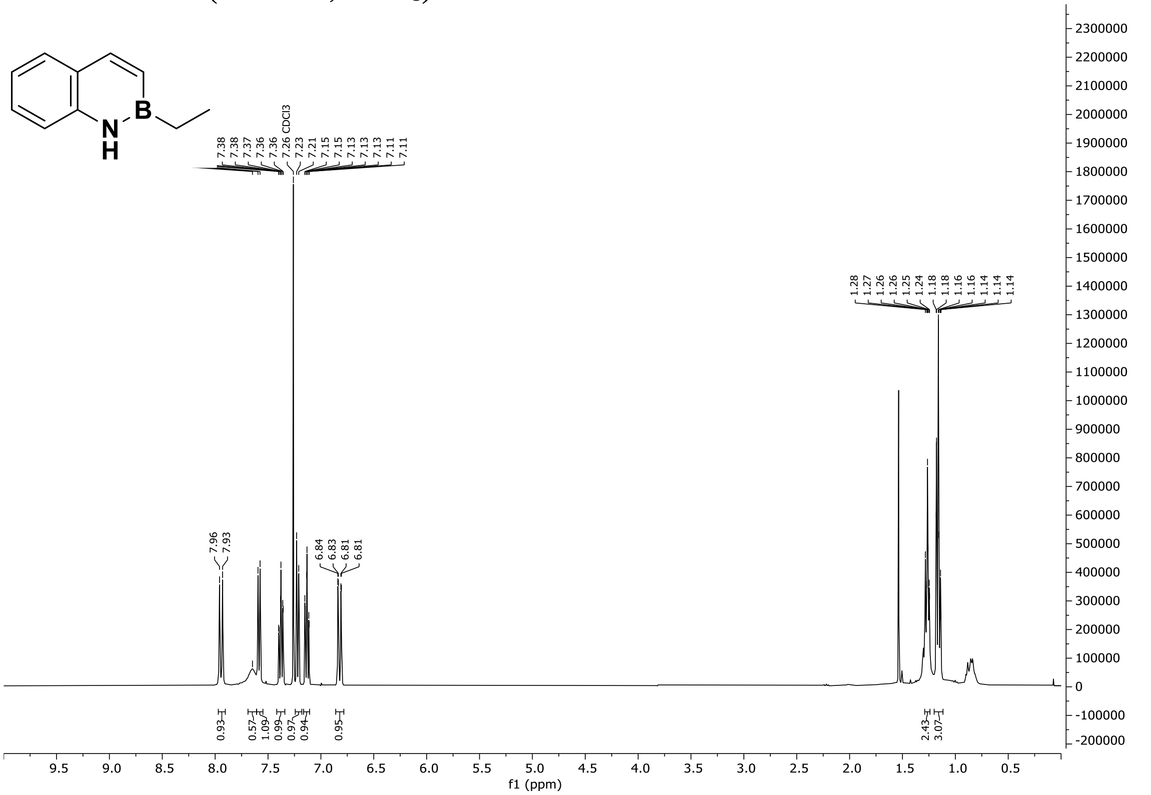

^{13}C-NMR of S25 (100 MHz, $CDCl_3$)

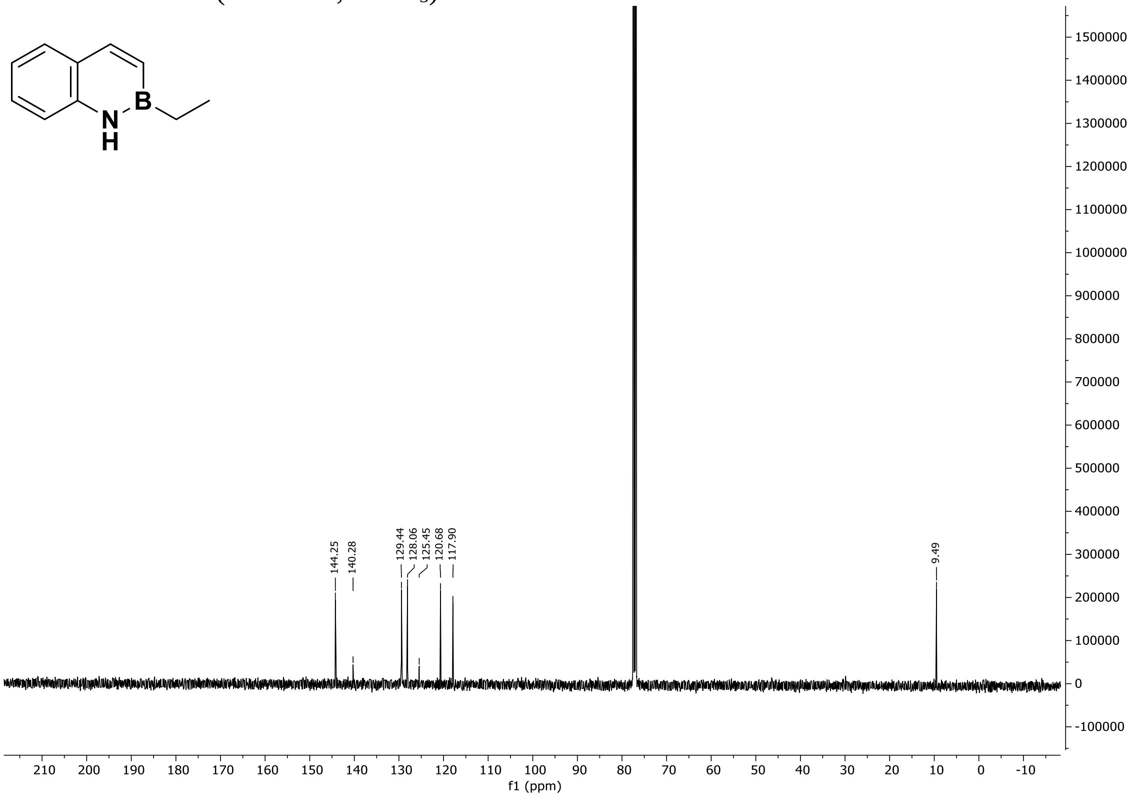

^{11}B-NMR of S25 (128 MHz, $CDCl_3$)

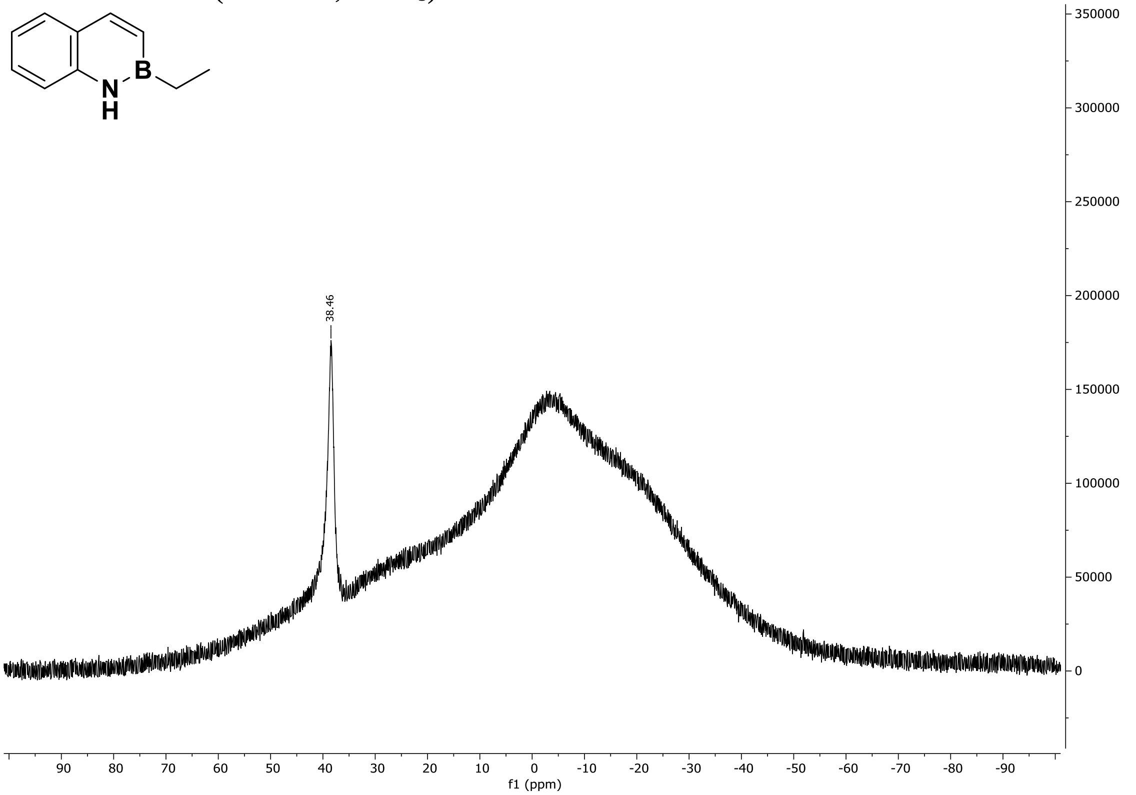

^{1}H-NMR of S26 (600 MHz, $CDCl_3$)

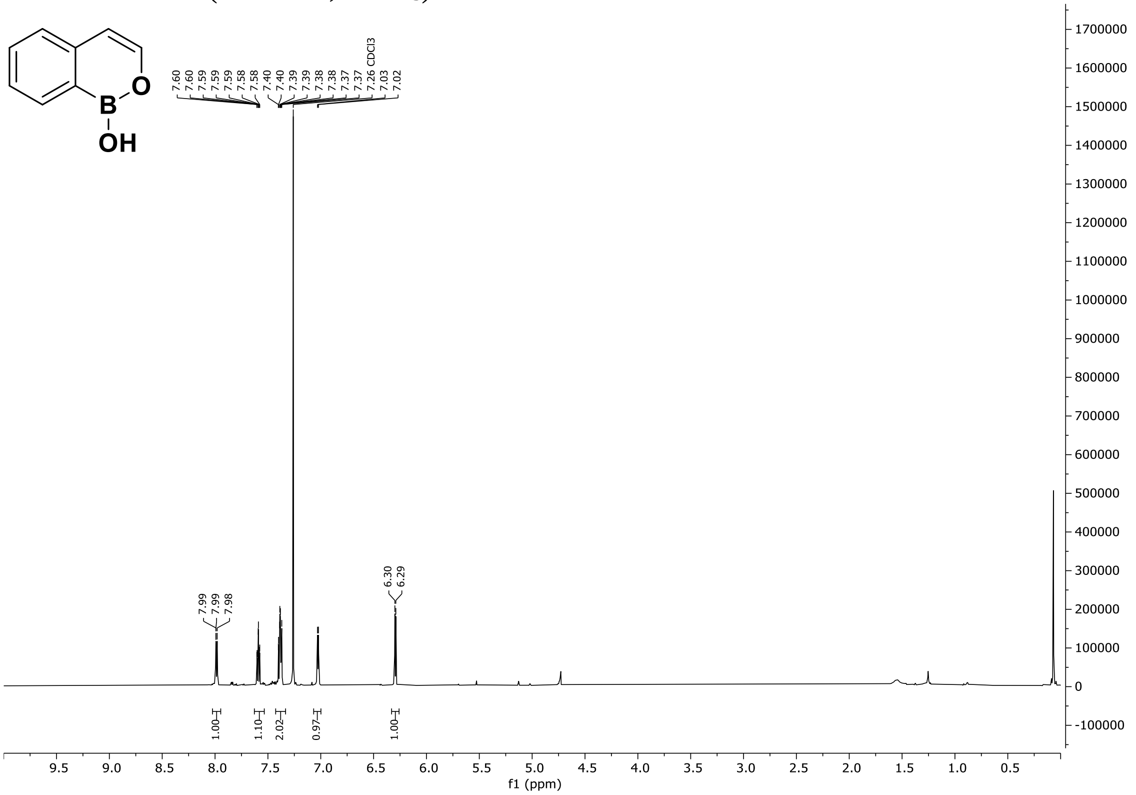

^{1}H-NMR of S27 (400 MHz, $CDCl_3$)

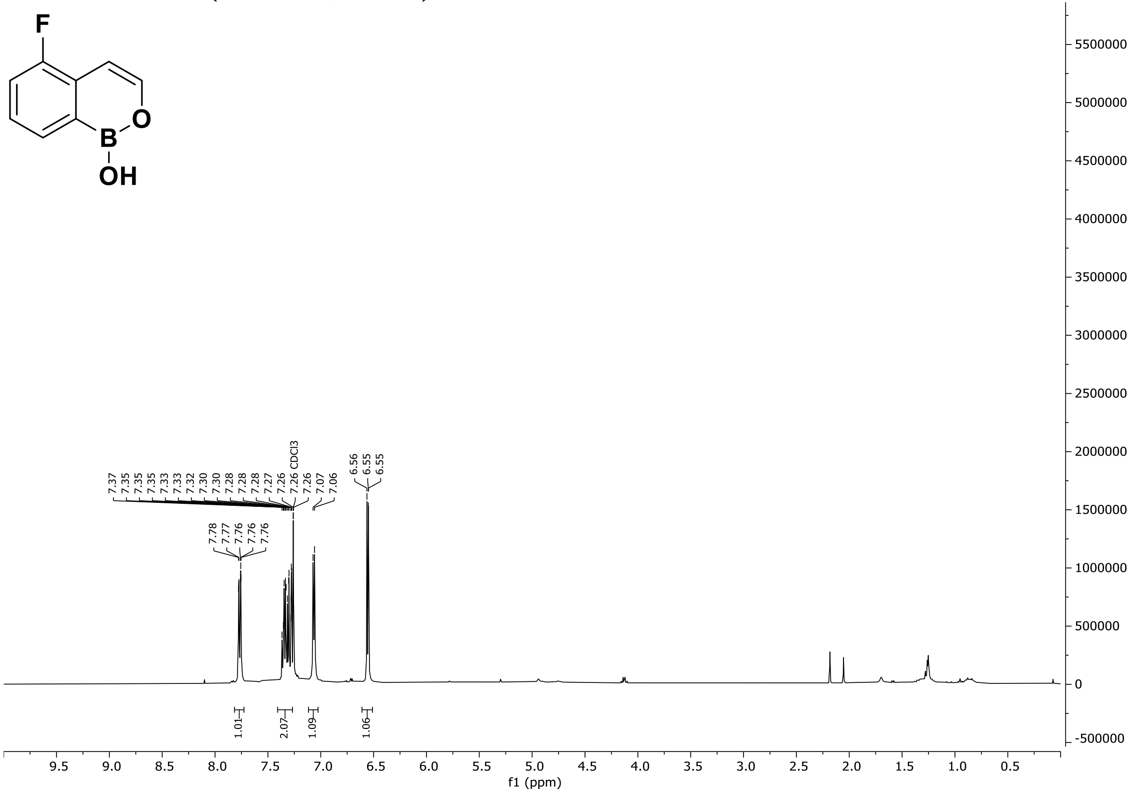

^{13}C-NMR of S27(100 MHz, $CDCl_3$)

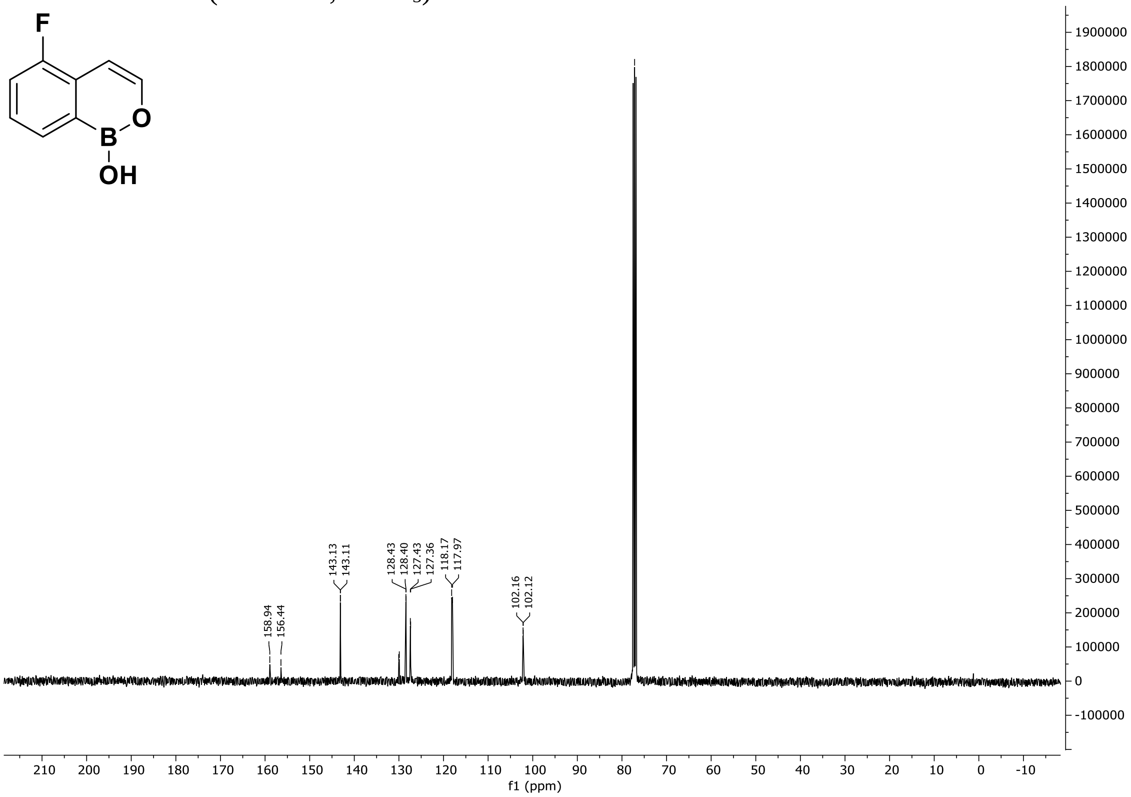

^{11}B-NMR of S27 (128 MHz, $CDCl_3$)

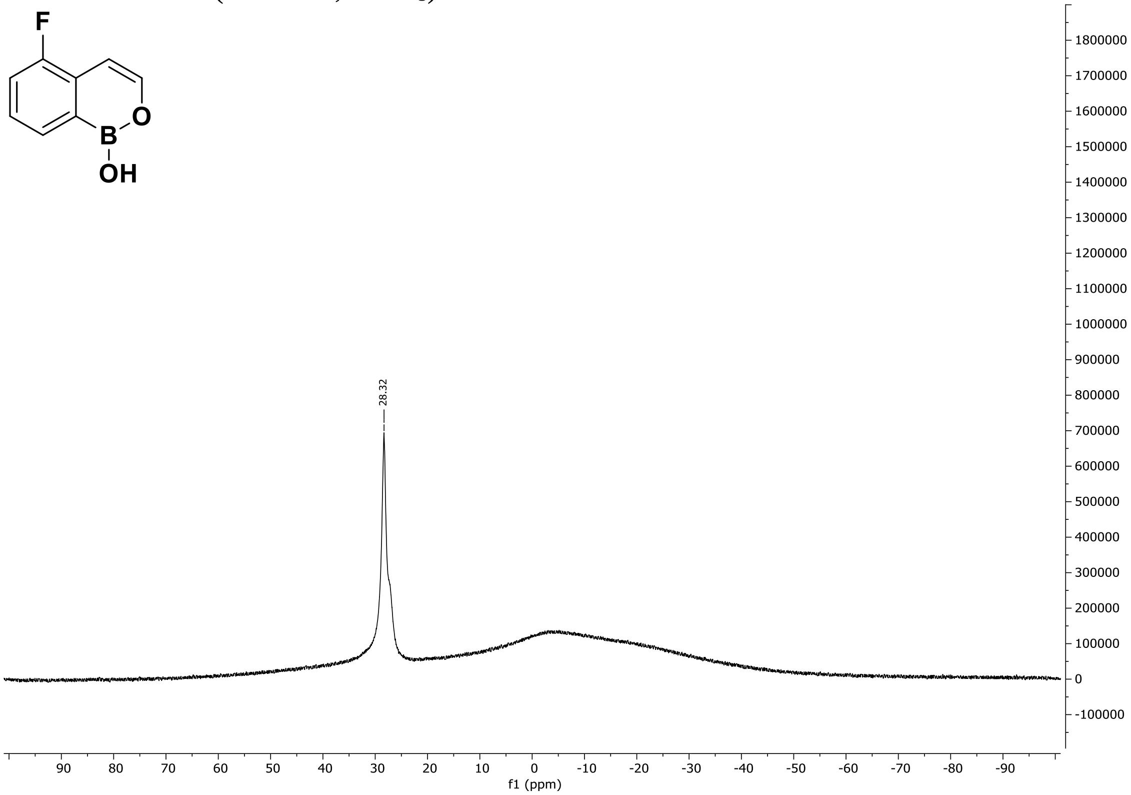

^{19}F-NMR of S27 (376 MHz, $CDCl_3$)

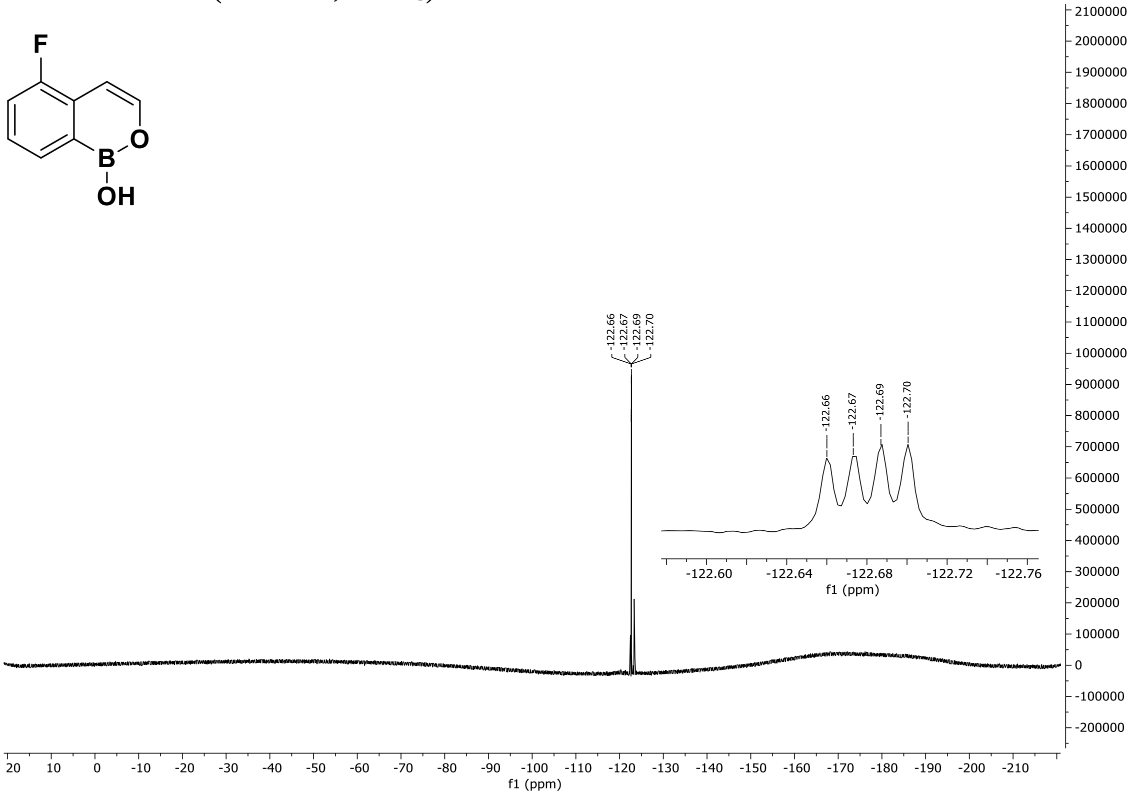

^{1}H-NMR of S28 (400 MHz, $CDCl_3$)

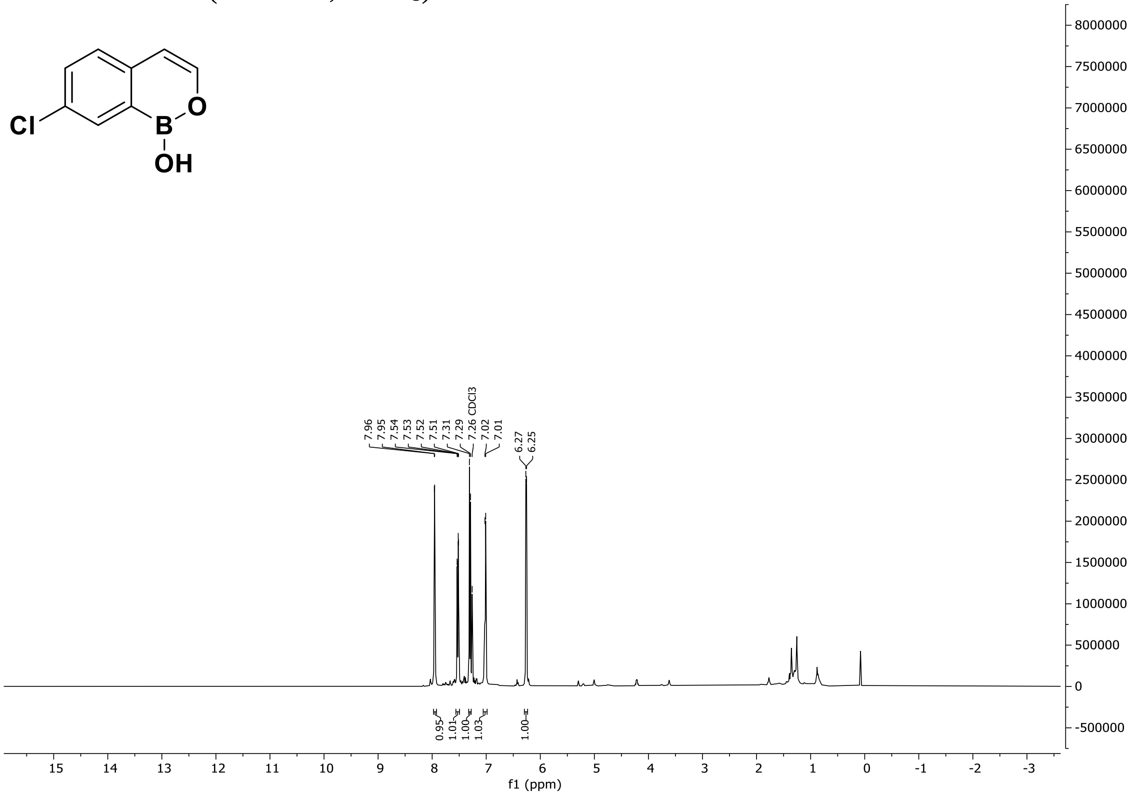

^{13}C-NMR of S28 (100 MHz, $CDCl_3$)

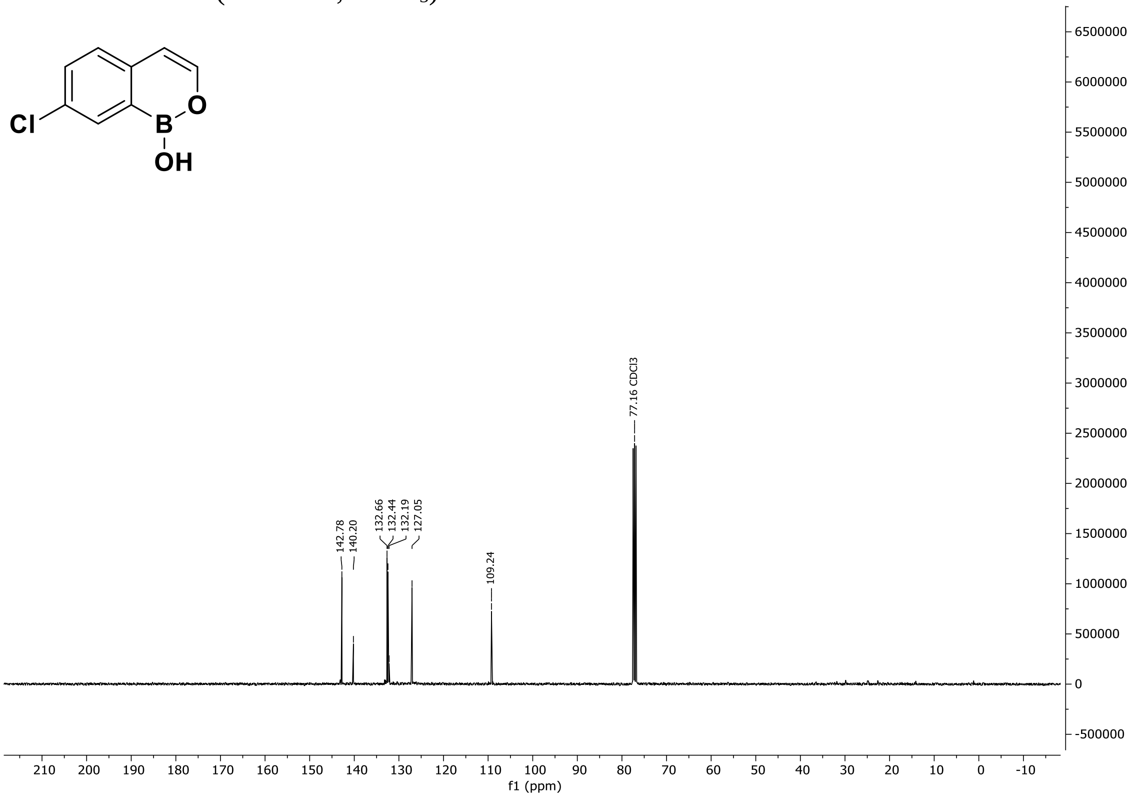

^{11}B-NMR of S28 (128 MHz, $CDCl_3$)

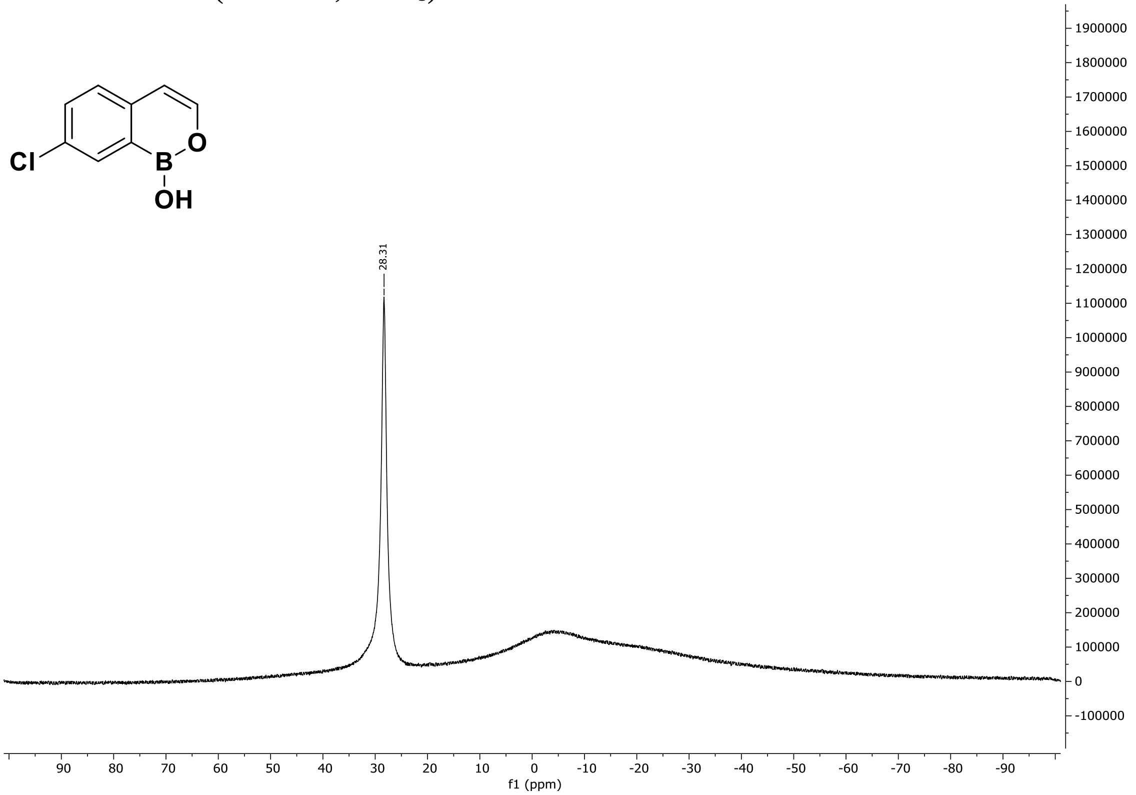

^{1}H-NMR of S29 (400 MHz, $CDCl_3$)

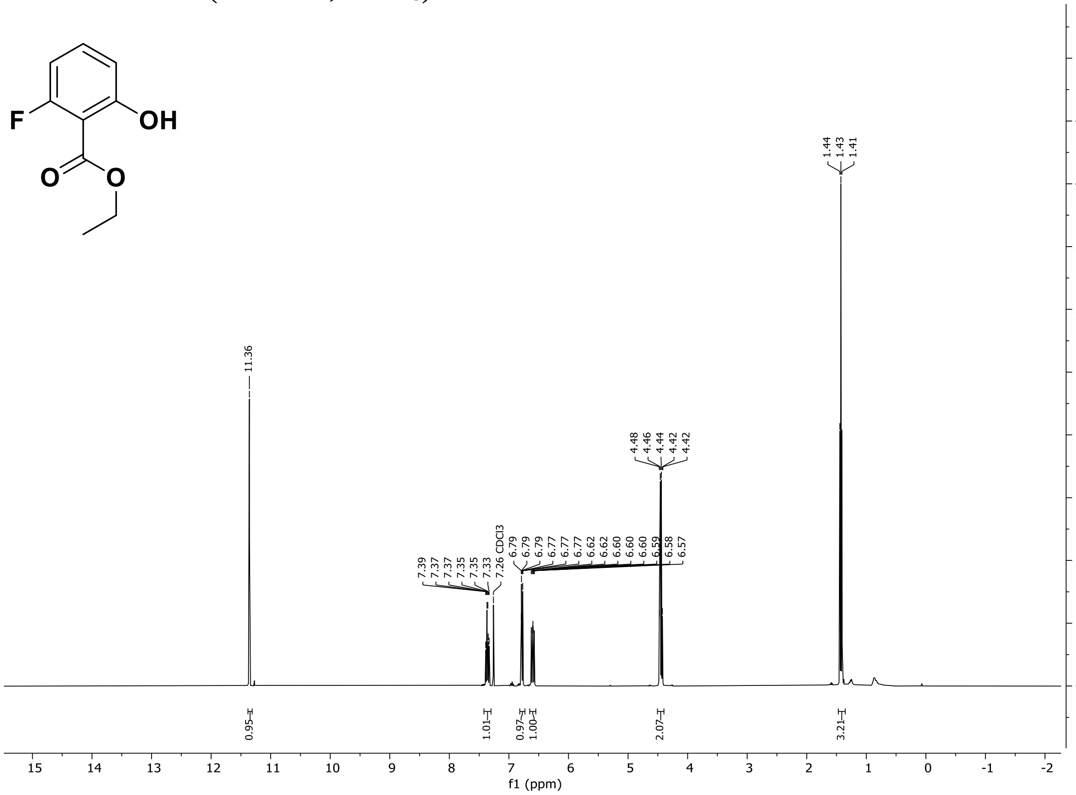

^{1}H-NMR of S30 (600 MHz, $CDCl_3$)

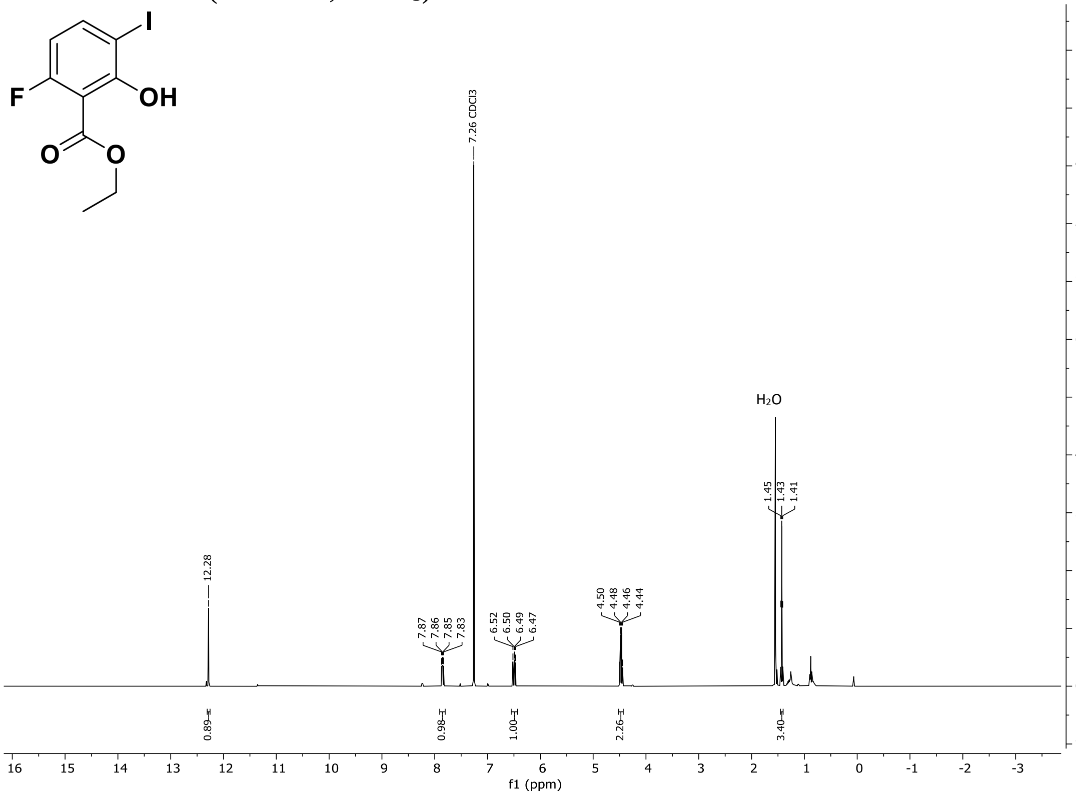

^{1}H-NMR of S31 (400 MHz, $CDCl_3$)

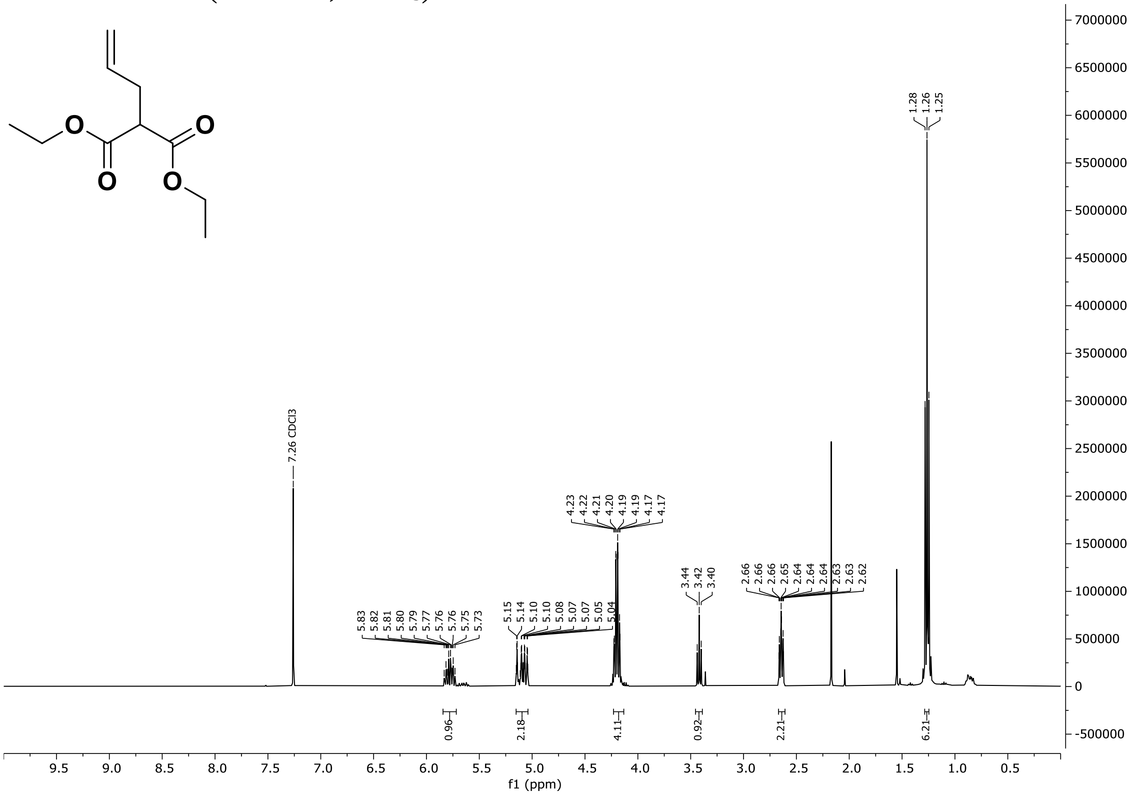

^{1}H-NMR of S32 (400 MHz, $CDCl_3$)

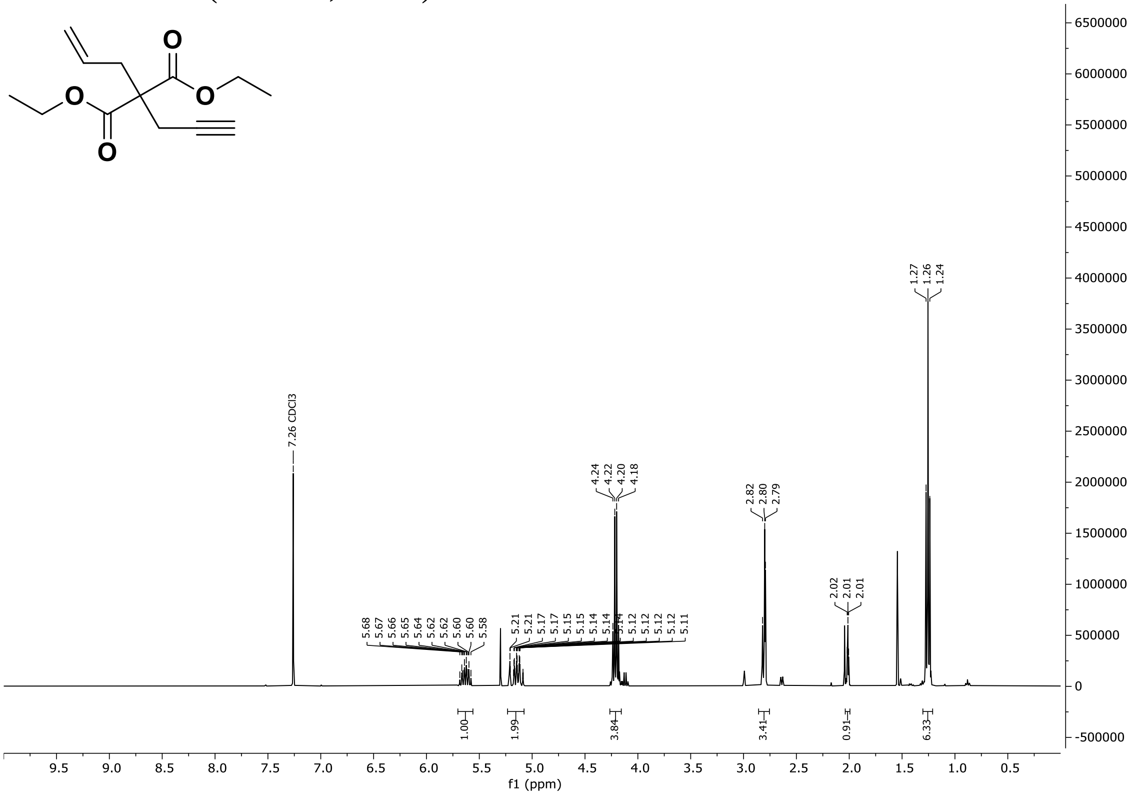

^{1}H-NMR of S33 (400 MHz, $CDCl_3$)

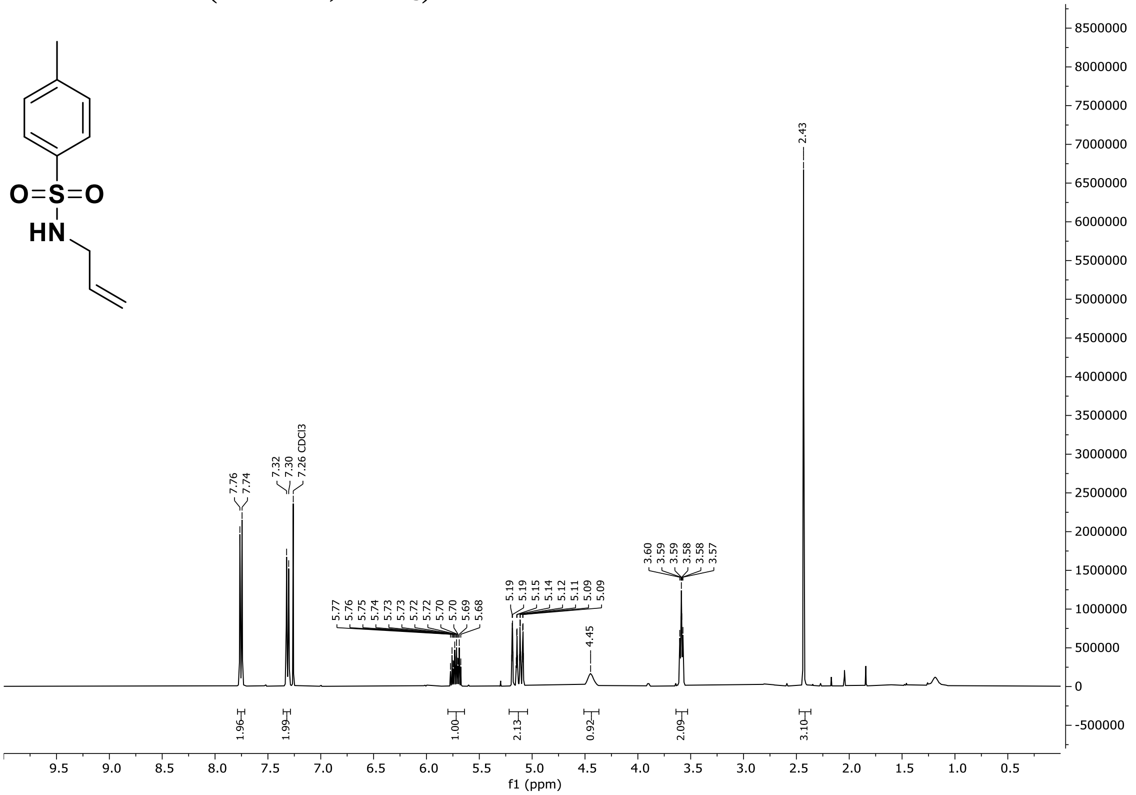

^{1}H-NMR of S34 (400 MHz, $CDCl_3$)

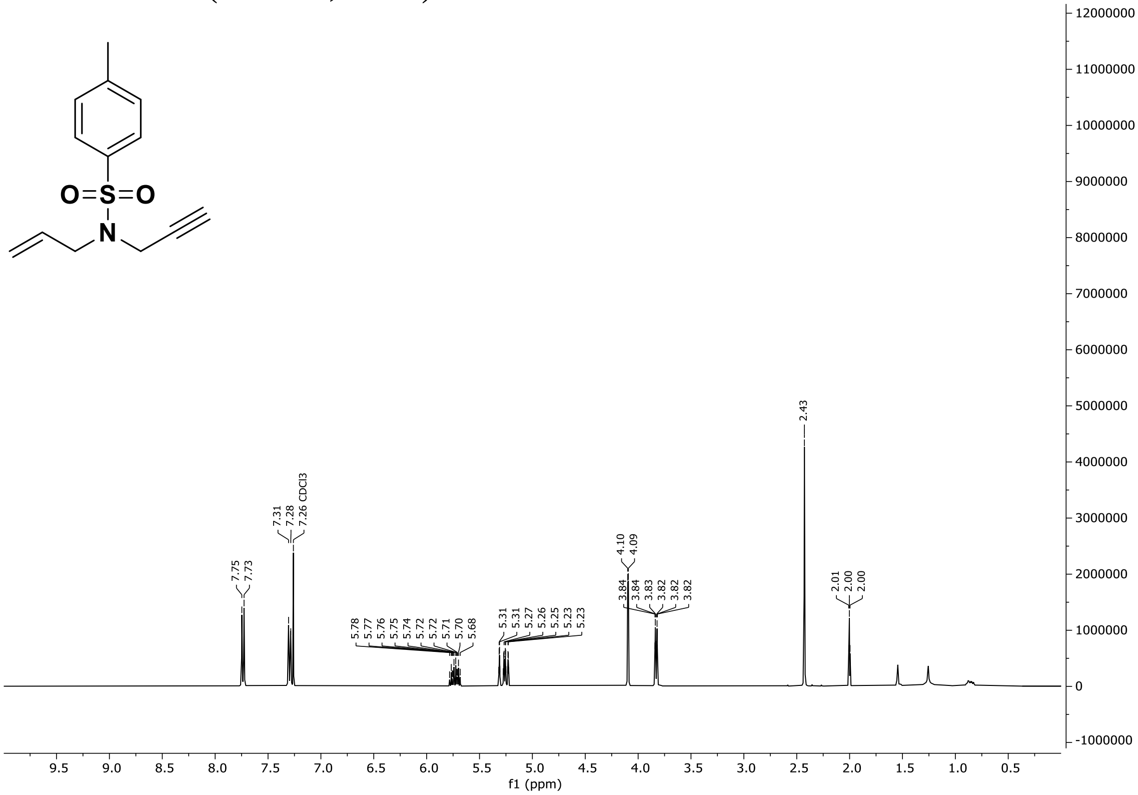

^{1}H-NMR of S35 (400 MHz, $CDCl_3$)

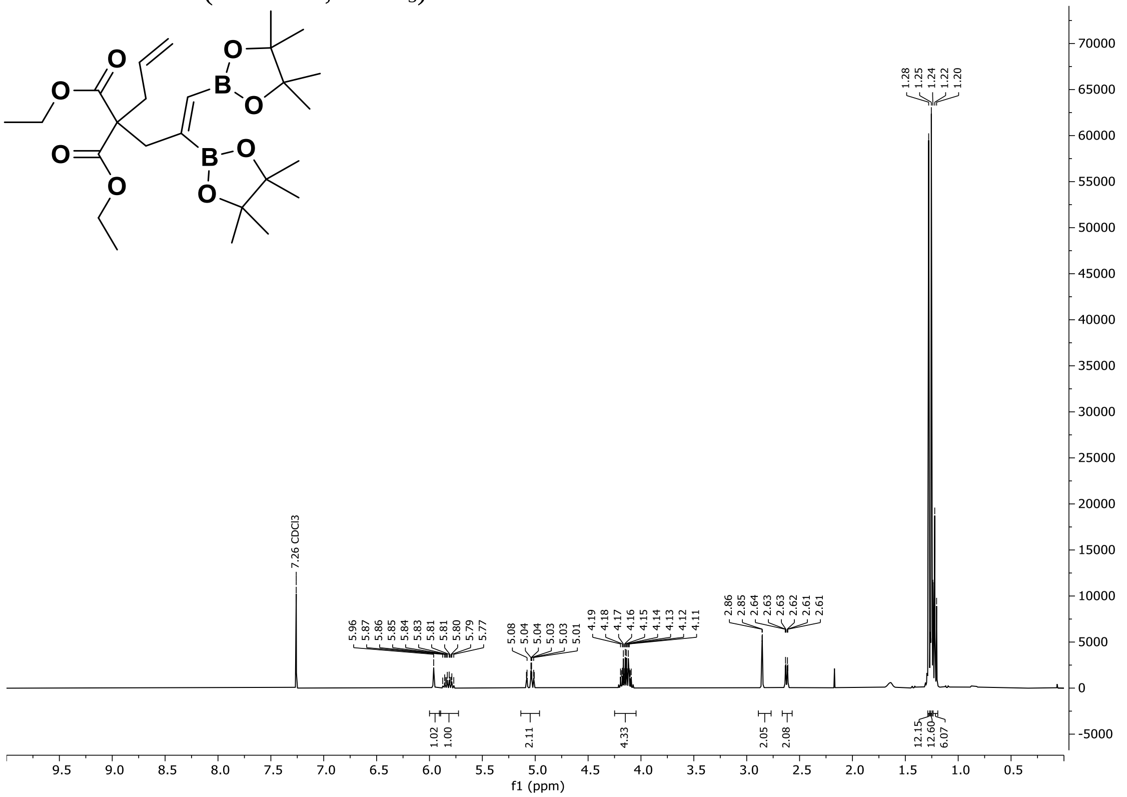

^{13}C-NMR of S35 (100 MHz, $CDCl_3$)

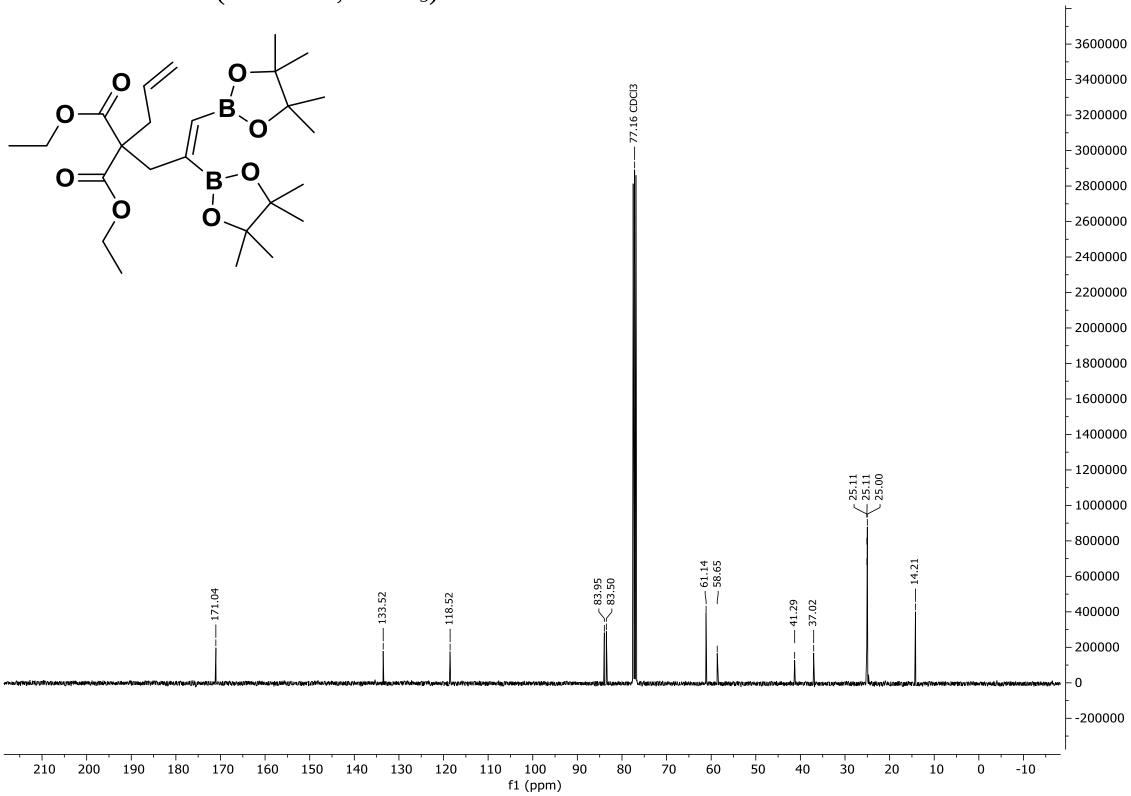

^{11}B-NMR of S35 (128 MHz, $CDCl_3$)

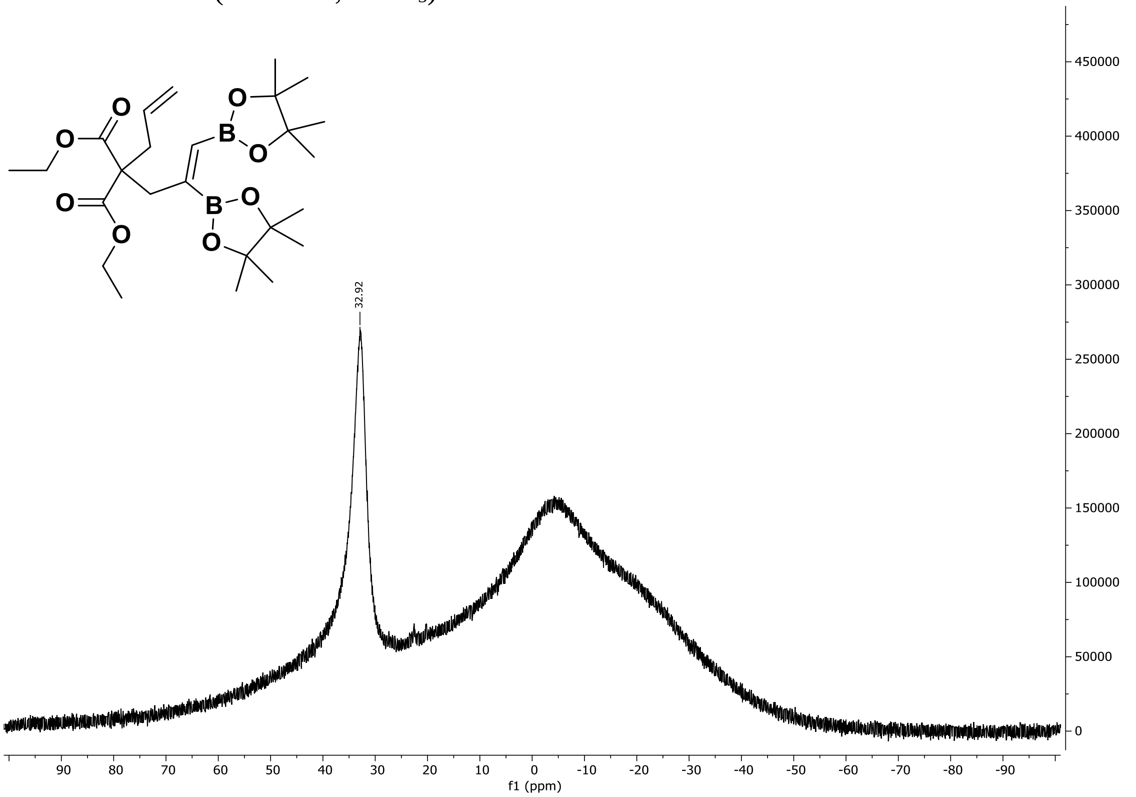

^{1}H-NMR of S36 (400 MHz, $CDCl_3$)

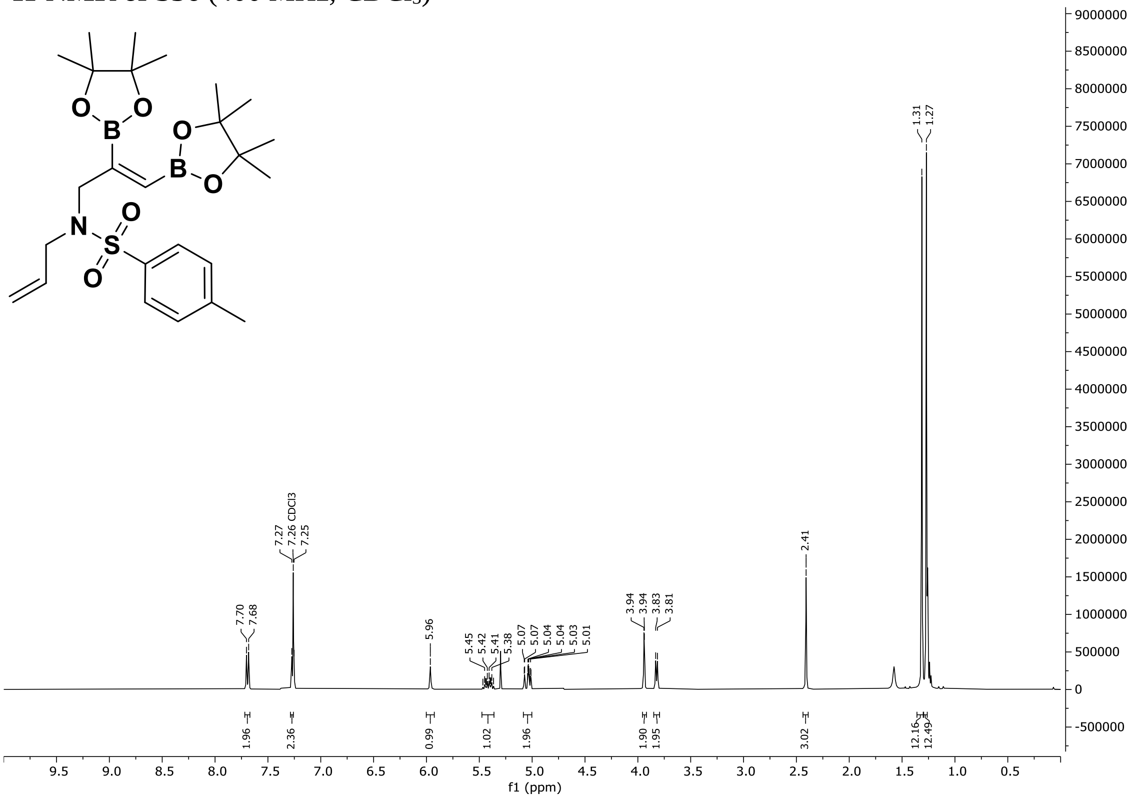

^{13}C-NMR of S36 (100 MHz, $CDCl_3$)

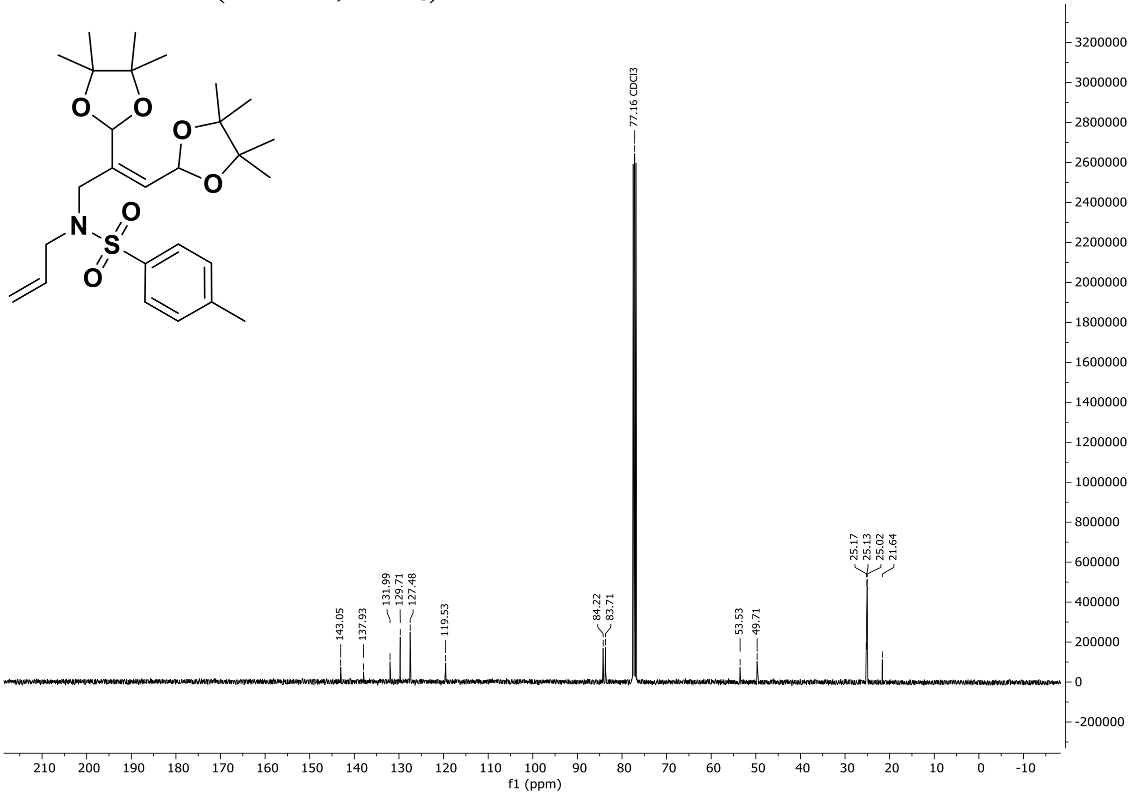

^{11}B-NMR of S36 (128 MHz, $CDCl_3$)

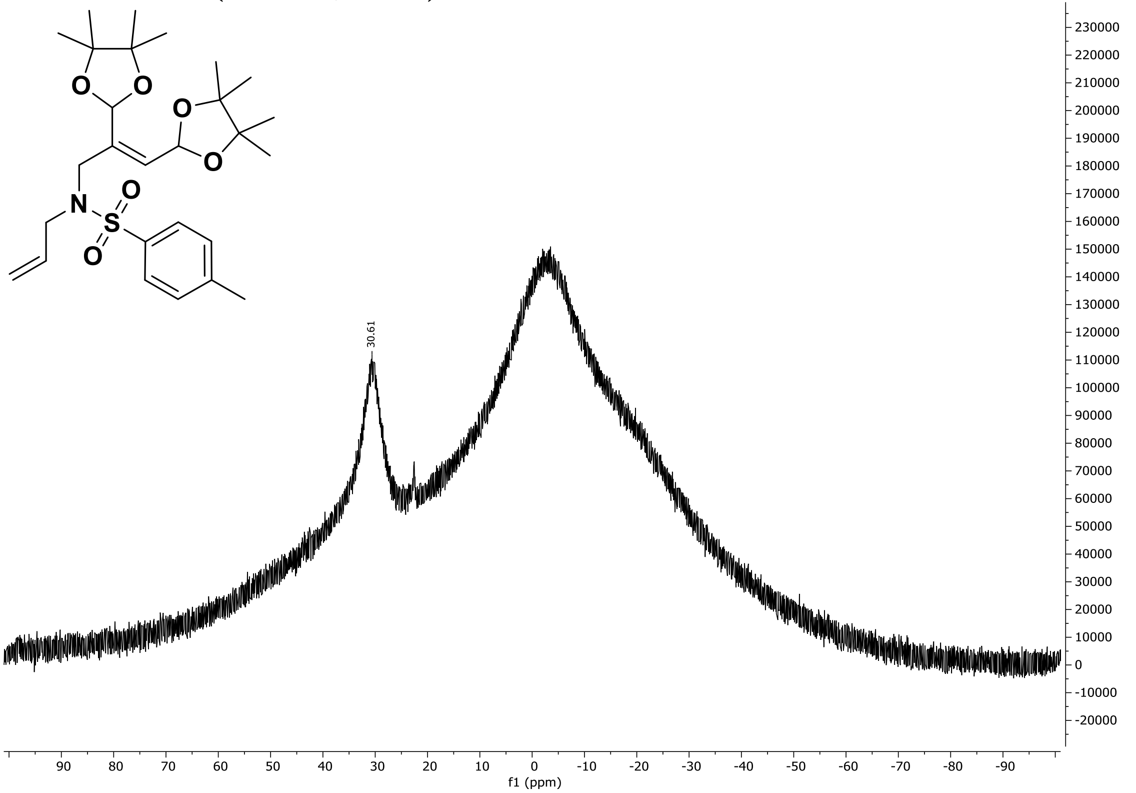

^{1}H-NMR of S37 (400 MHz, $CDCl_3$)

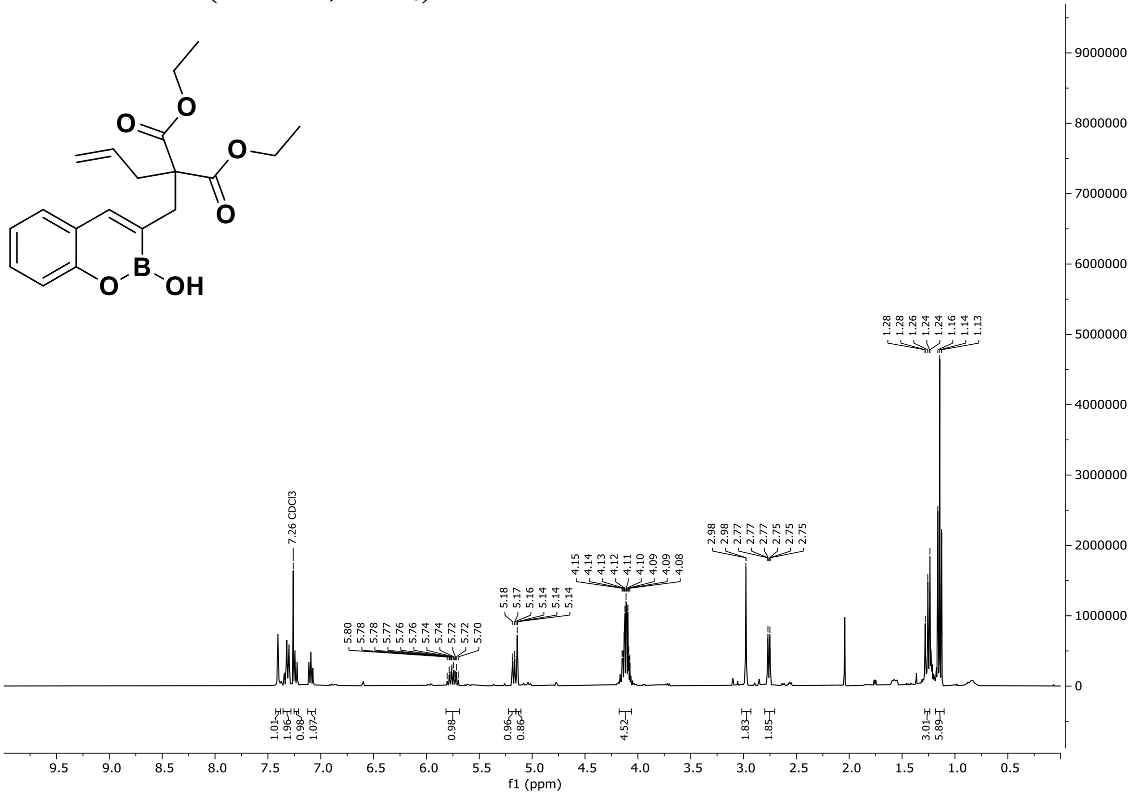

^{13}C-NMR of S37 (100 MHz, $CDCl_3$)

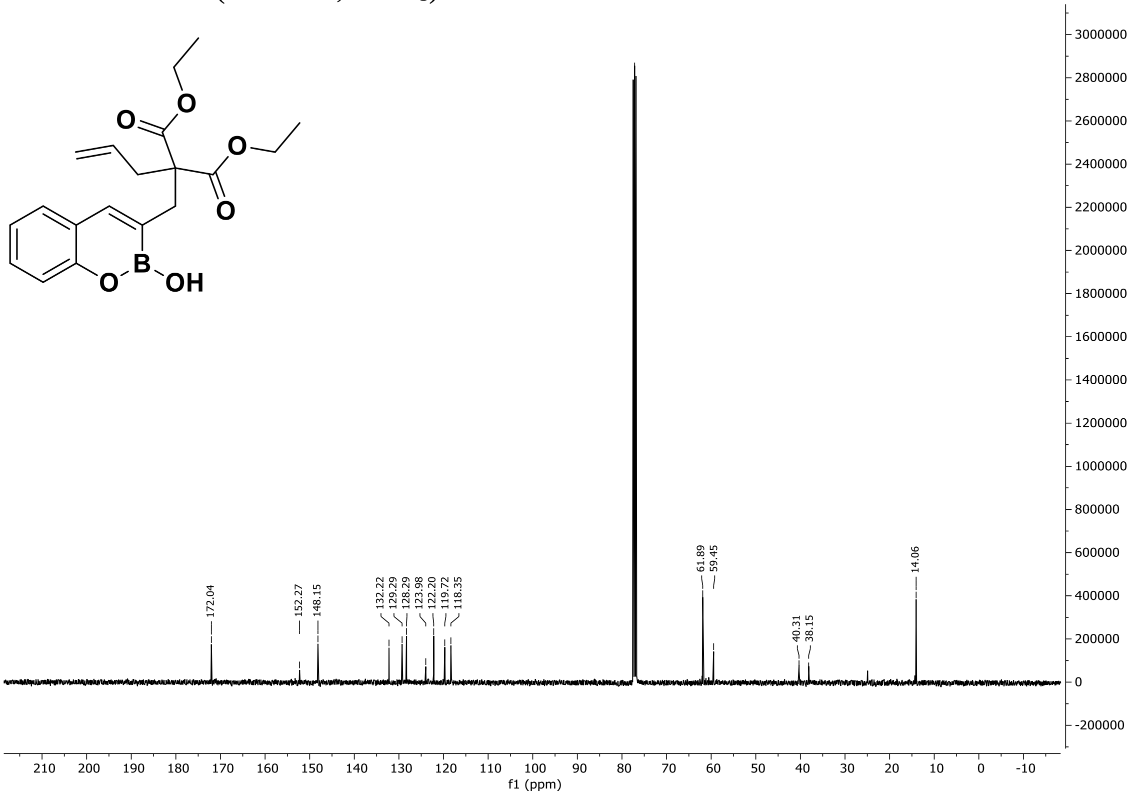

^{11}B-NMR of S37 (128 MHz, $CDCl_3$)

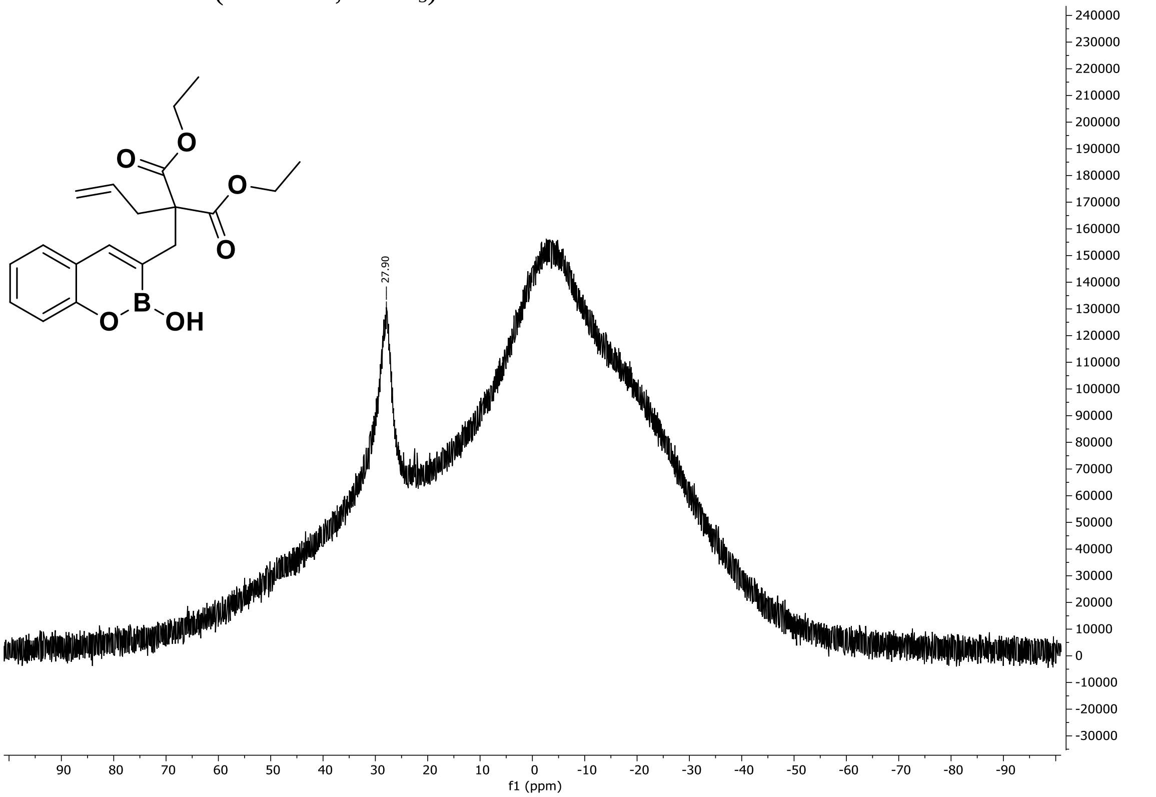

^{1}H-NMR of S38 (400 MHz, $CDCl_3$)

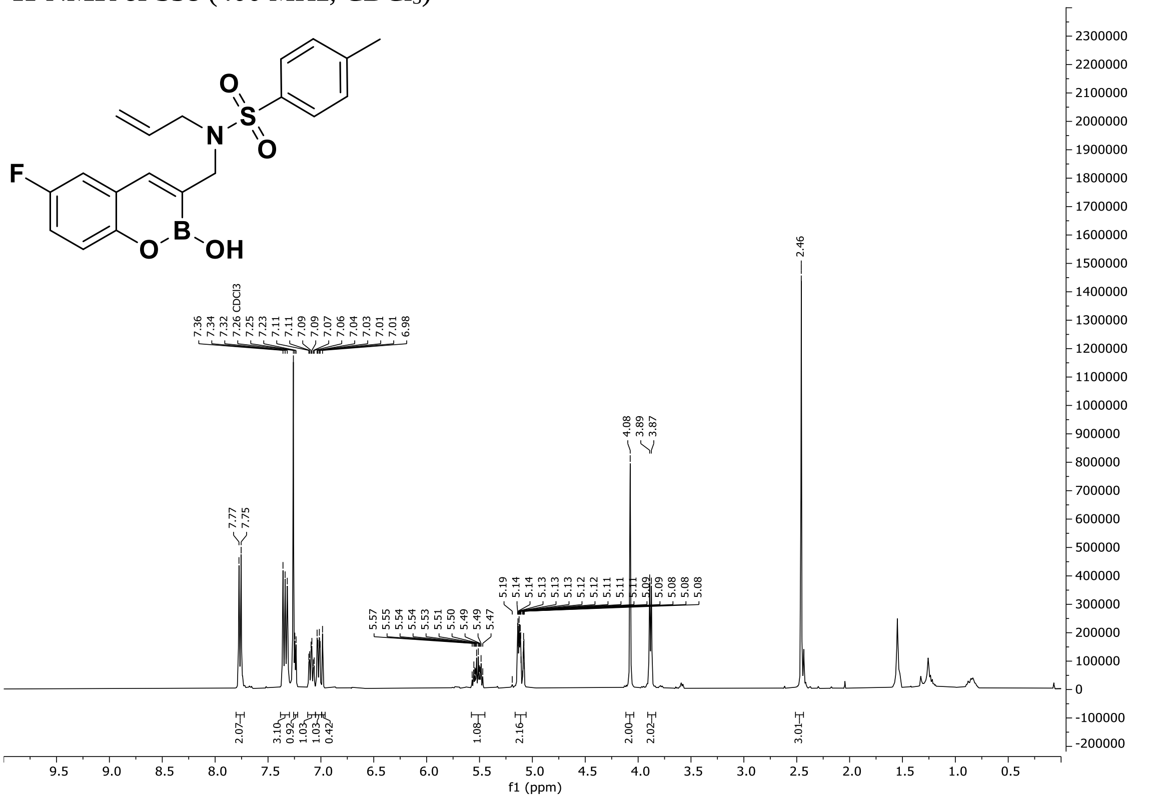

^{13}C-NMR of S38 (100 MHz, $CDCl_3$)

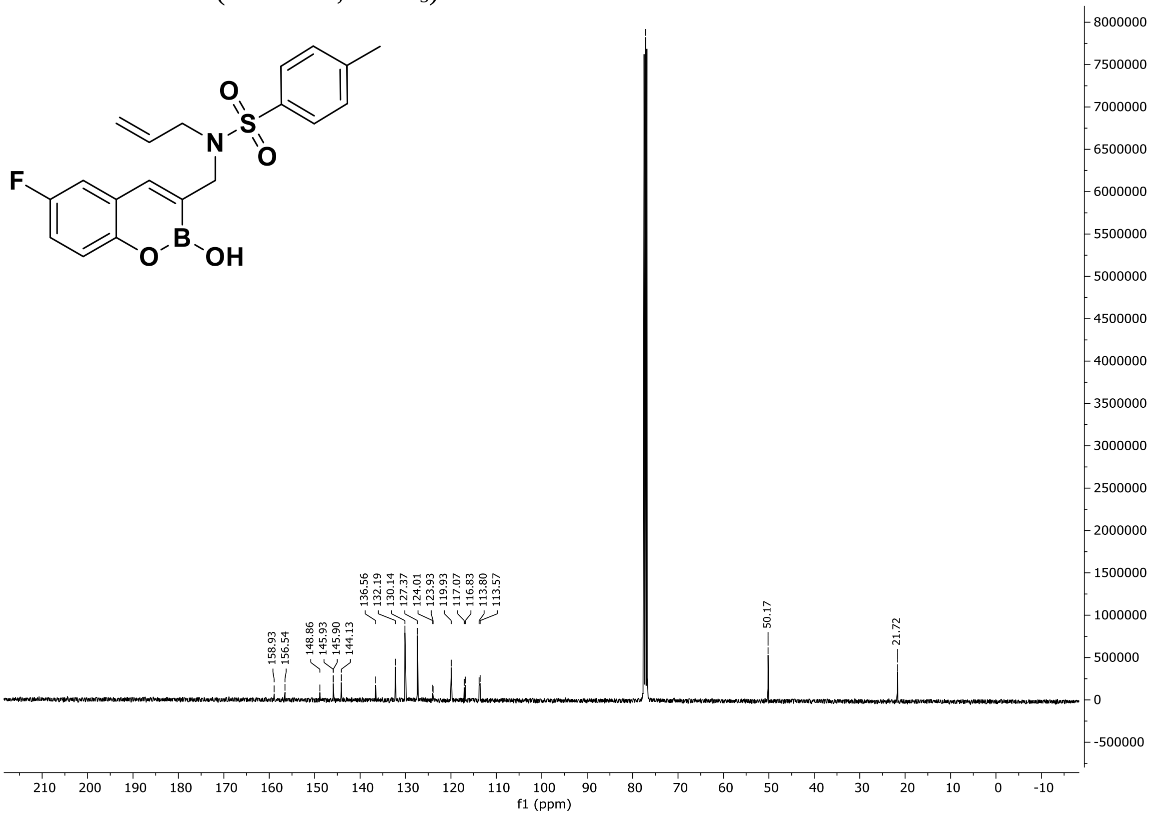

^{11}B-NMR of S38 (128 MHz, $CDCl_3$)

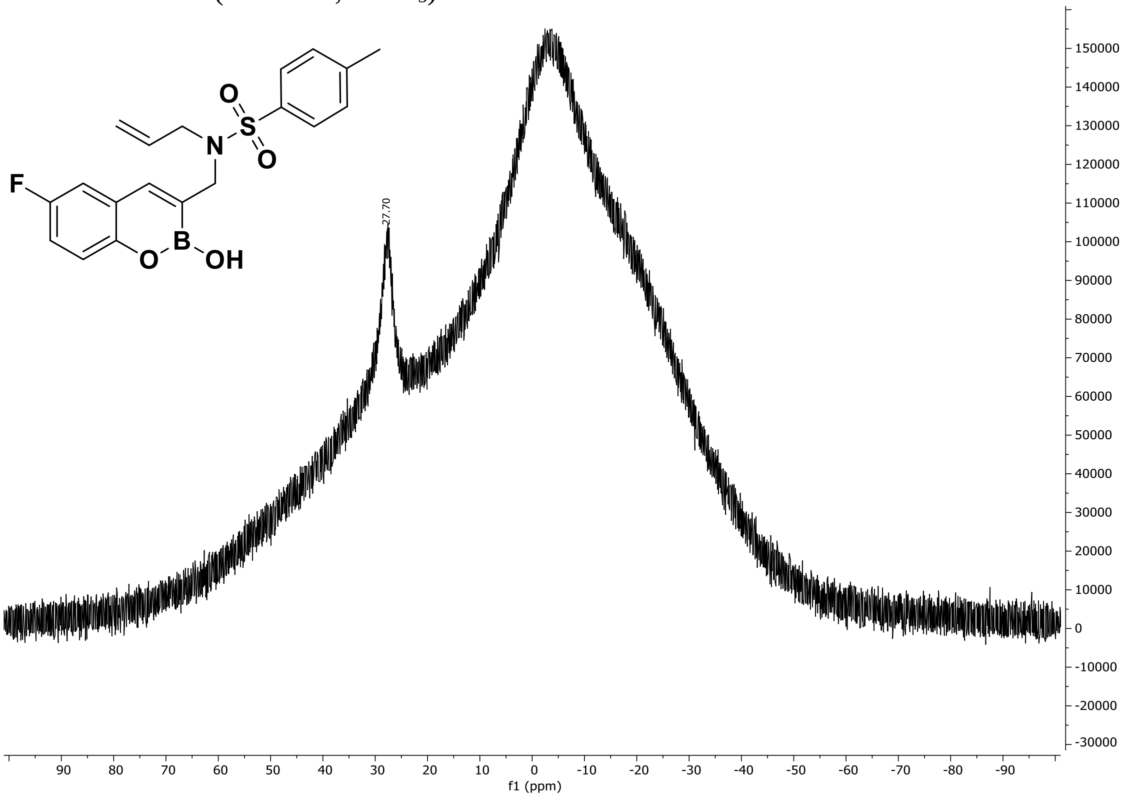

^{19}F-NMR of S38 (376 MHz, $CDCl_3$)

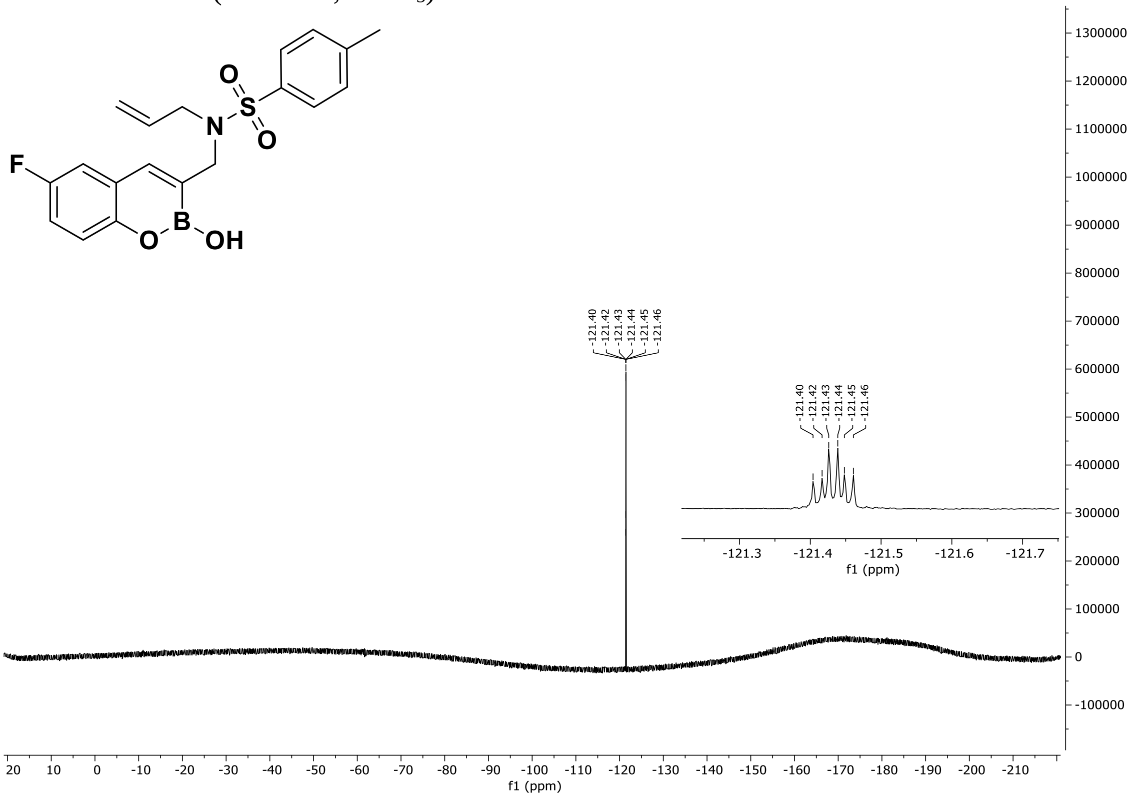

^{1}H-NMR of S39 (400 MHz, $CDCl_3$)

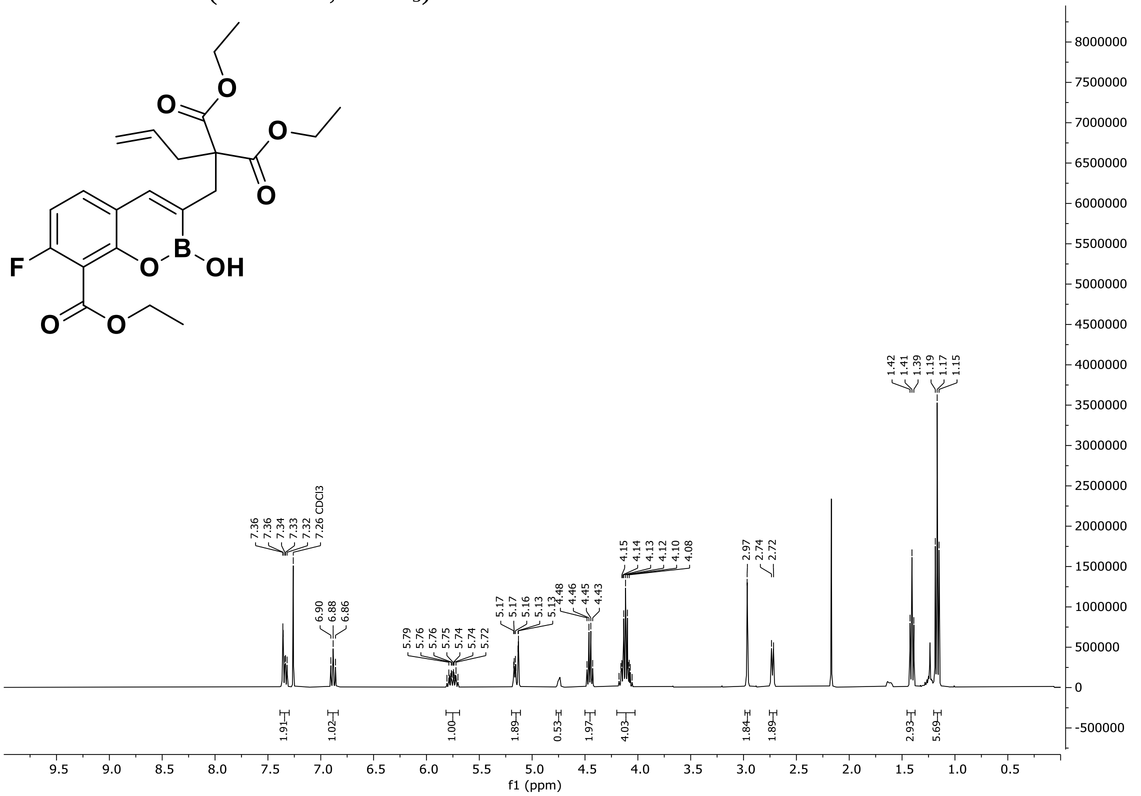

^{13}C-NMR of S39 (101 MHz, $CDCl_3$)

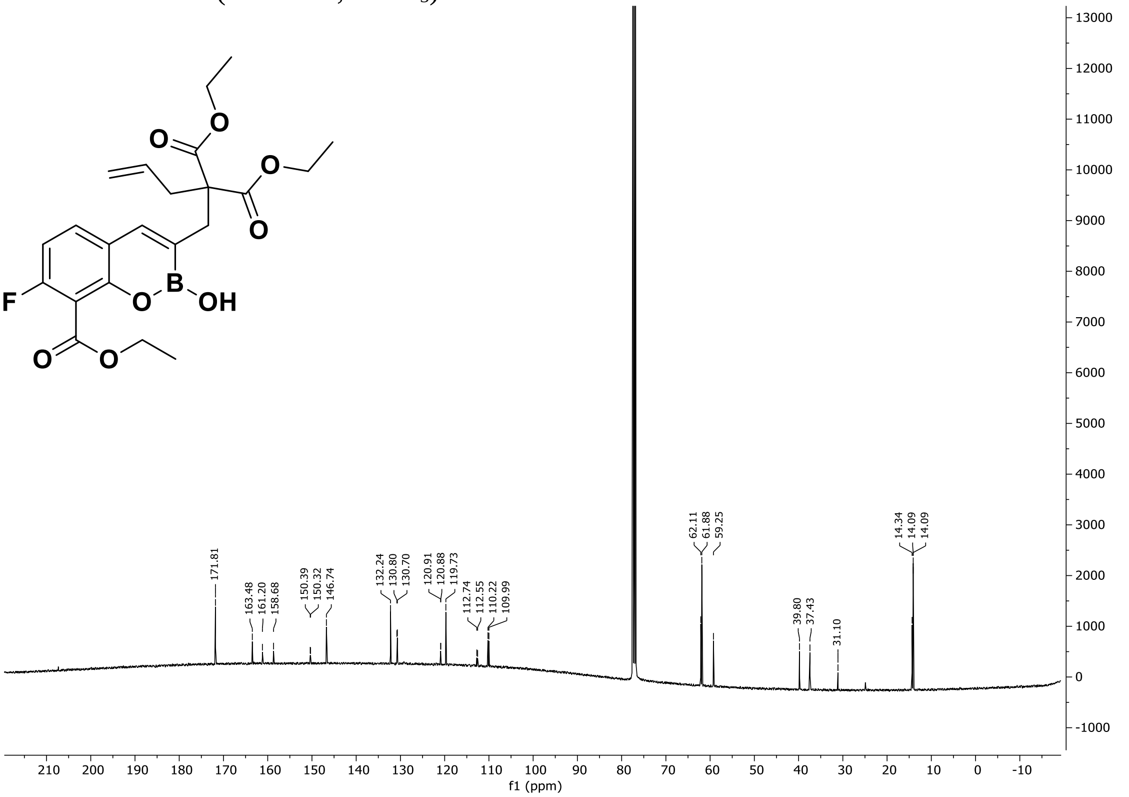

^{11}B-NMR of S39 (128 MHz, $CDCl_3$)

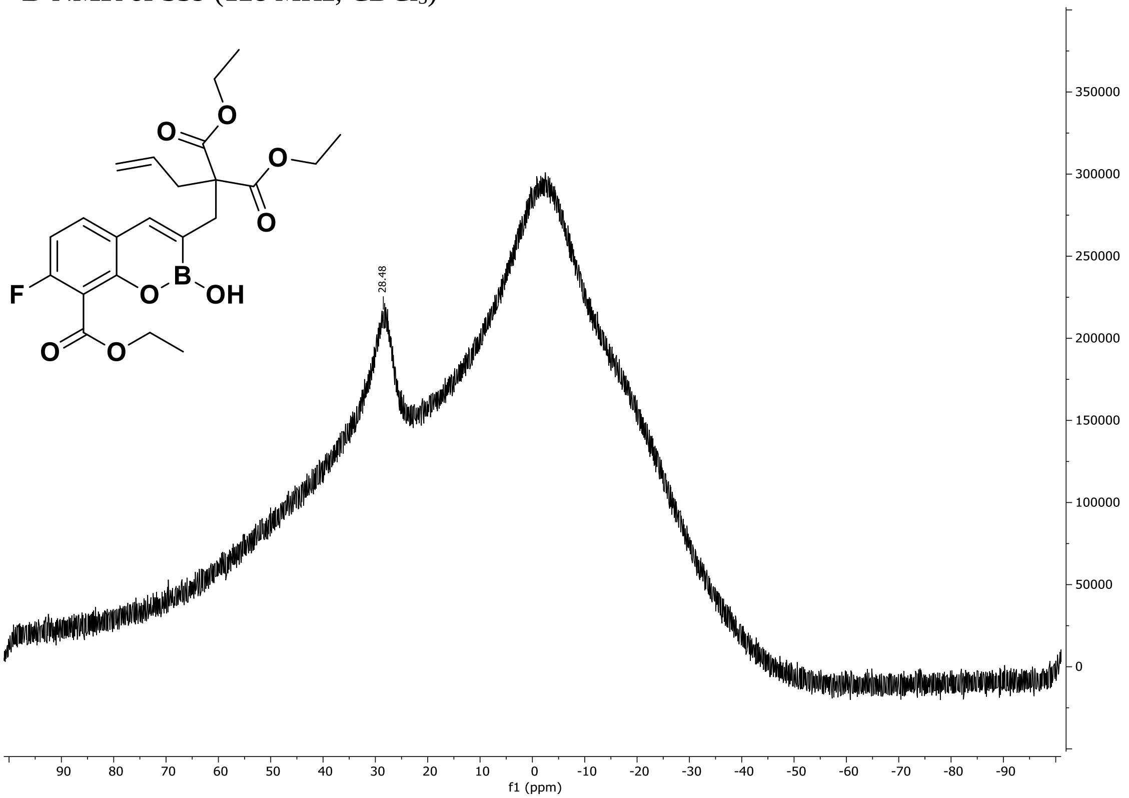

^{19}F-NMR of S39 (376 MHz, $CDCl_3$)

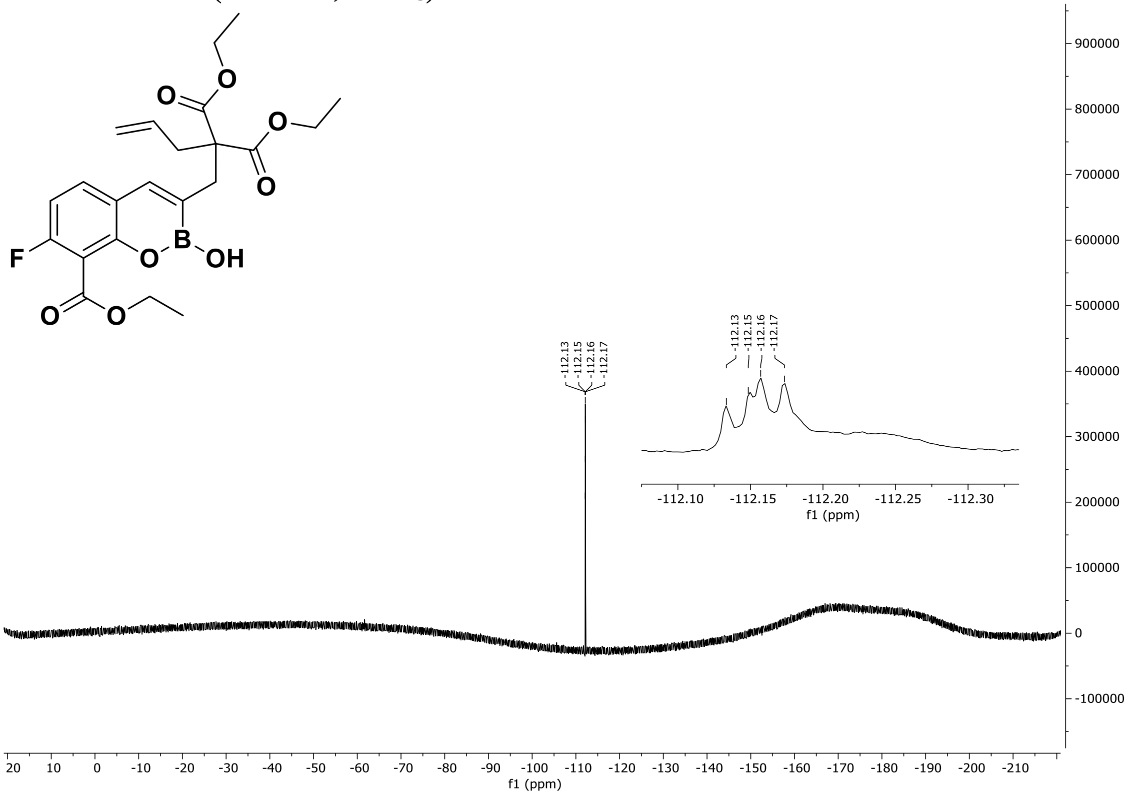

^{1}H-NMR of S40 (400 MHz, $CDCl_3$ with a drop of D_2O)

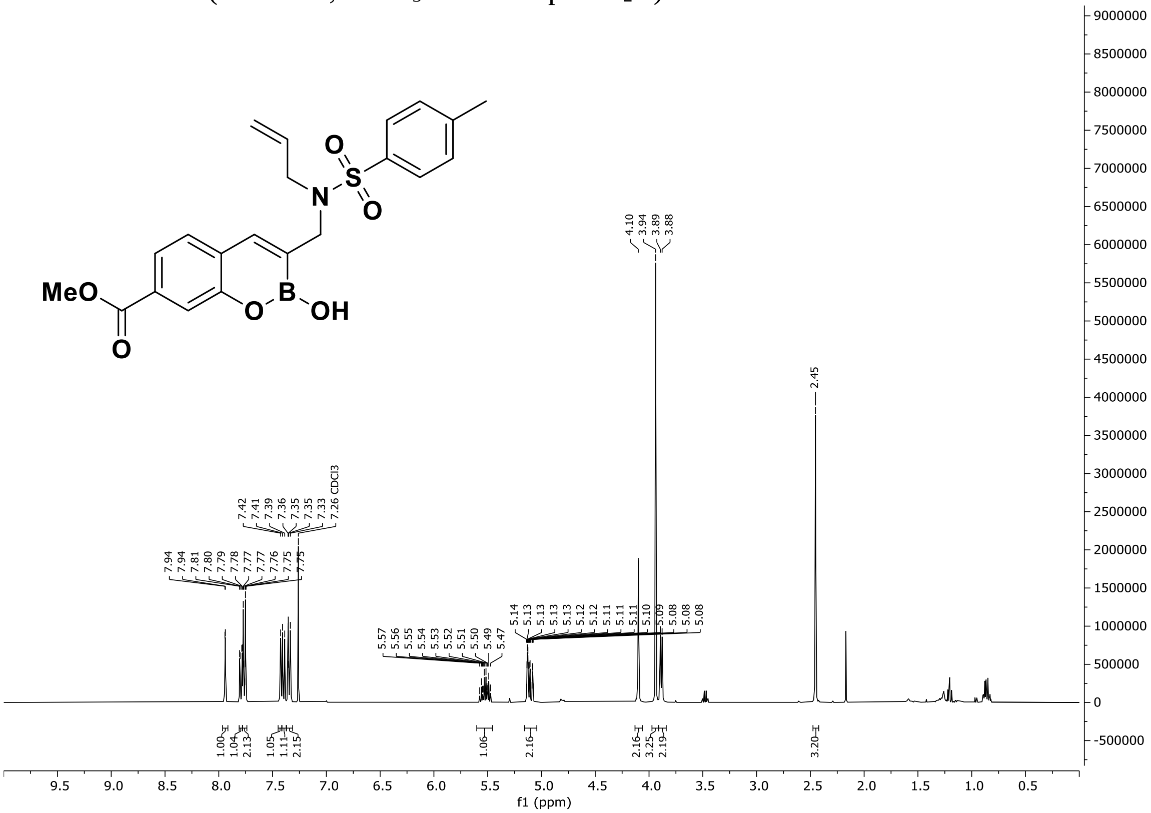

^{13}C-NMR of S40 (100 MHz, $CDCl_3$ with a drop of D_2O)

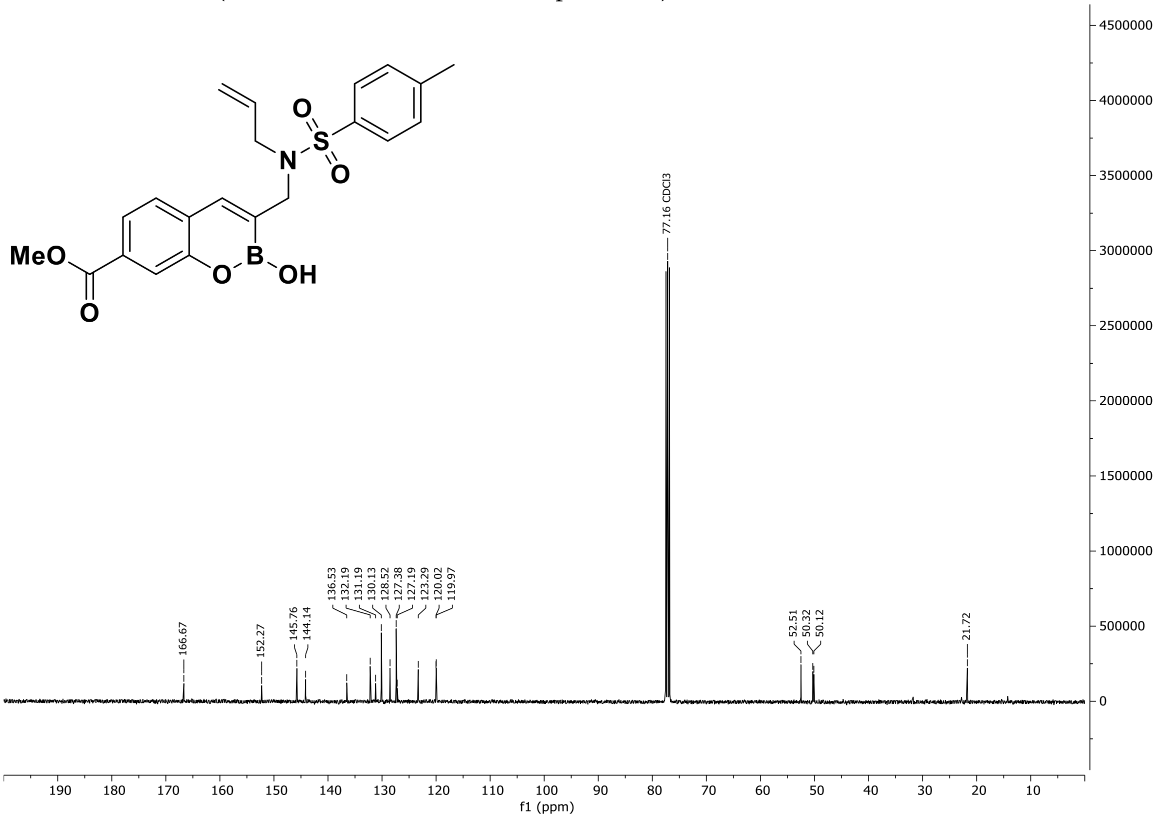

^{11}B-NMR of S40 (128 MHz $CDCl_3$ with a drop of D_2O)

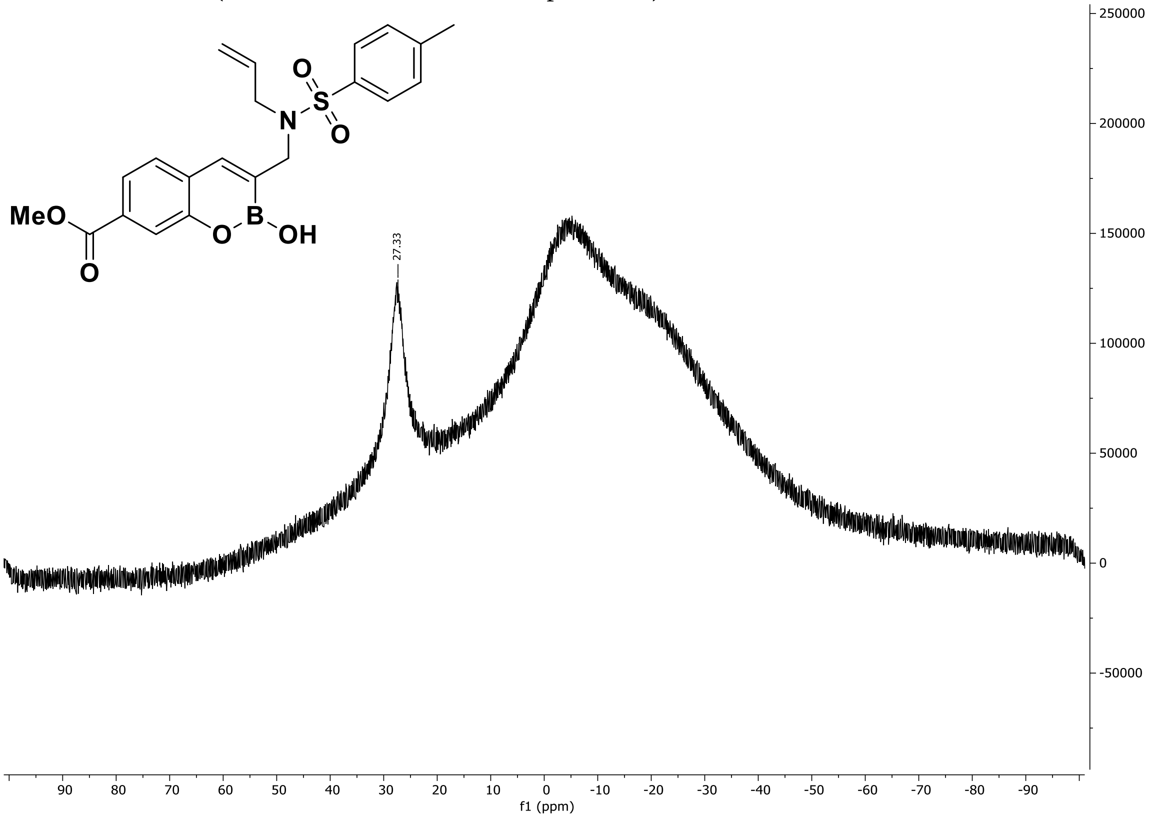

^{1}H-NMR of S41 (400 MHz, $CDCl_3$ with a drop of D_2O)

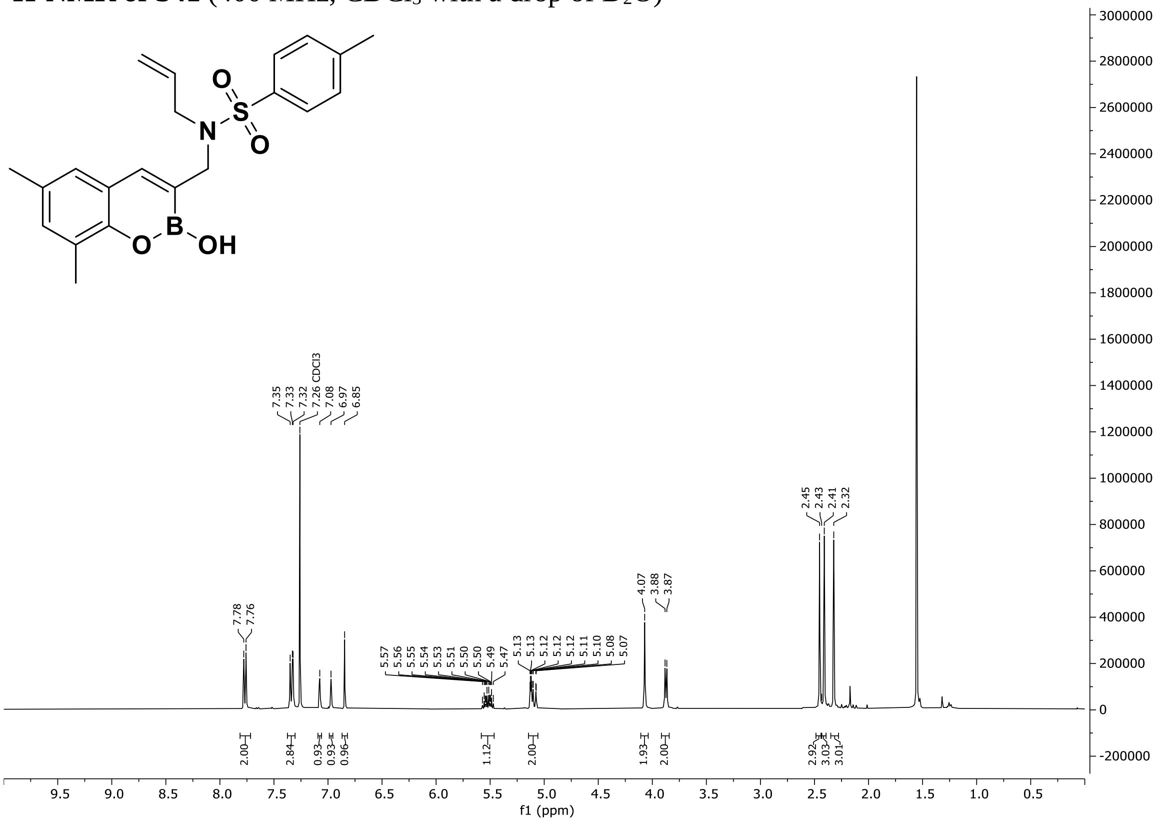

^{13}C-NMR of S41 (100 MHz, $CDCl_3$ with a drop of D_2O)

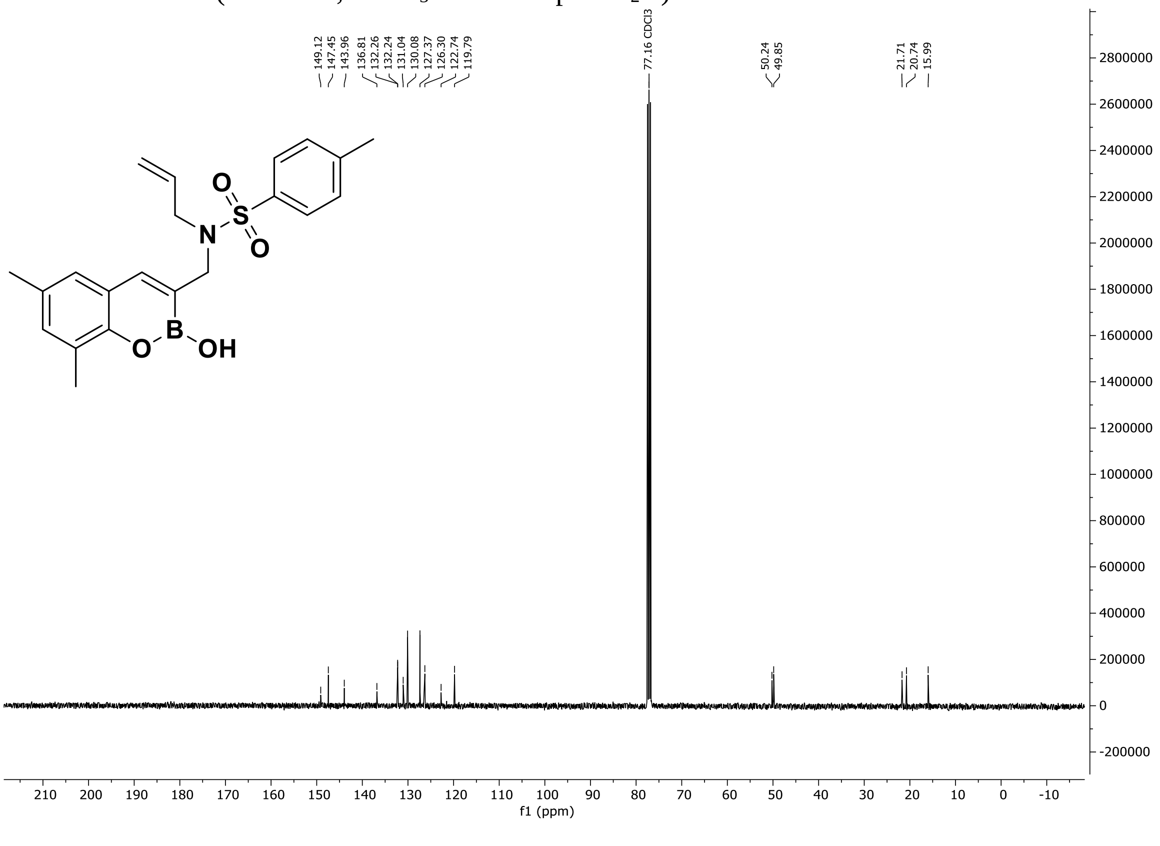

^{11}B-NMR of S41 (128 MHz $CDCl_3$ with a drop of D_2O)

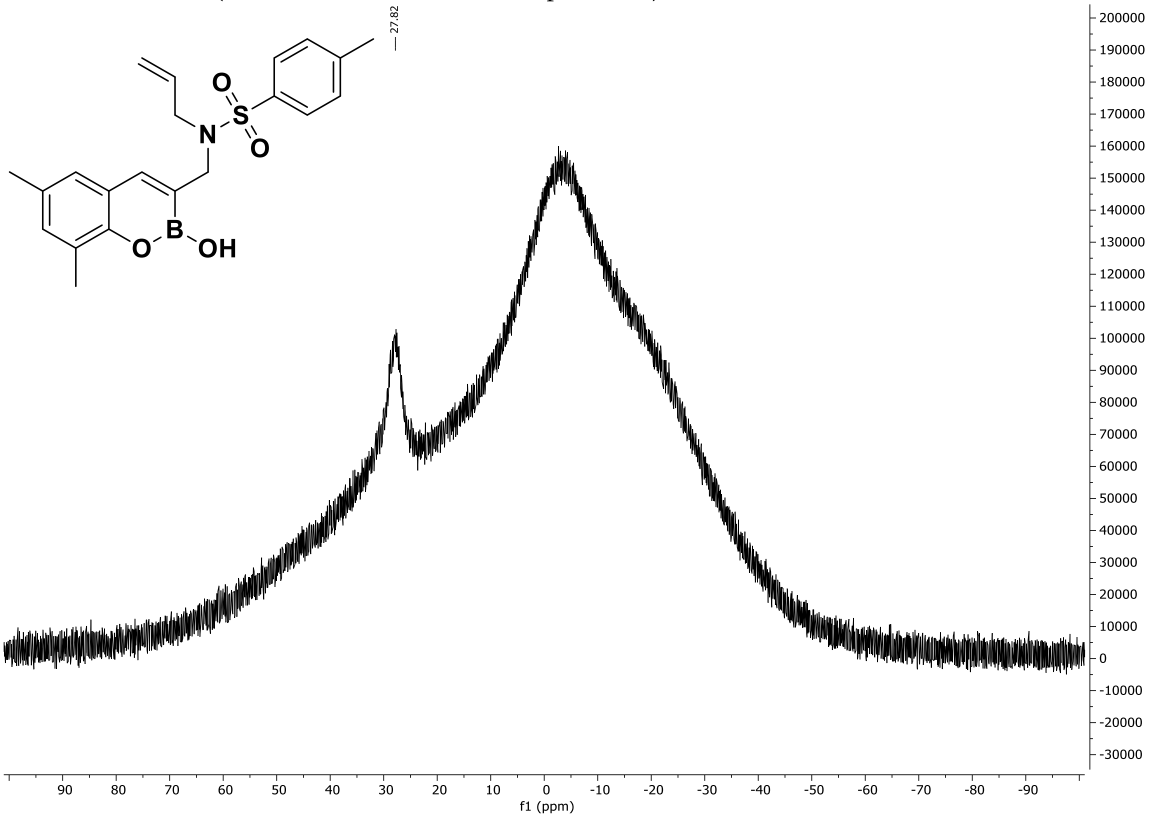

^{1}H-NMR of S42 (400 MHz, $CDCl_3$ with a drop of D_2O)

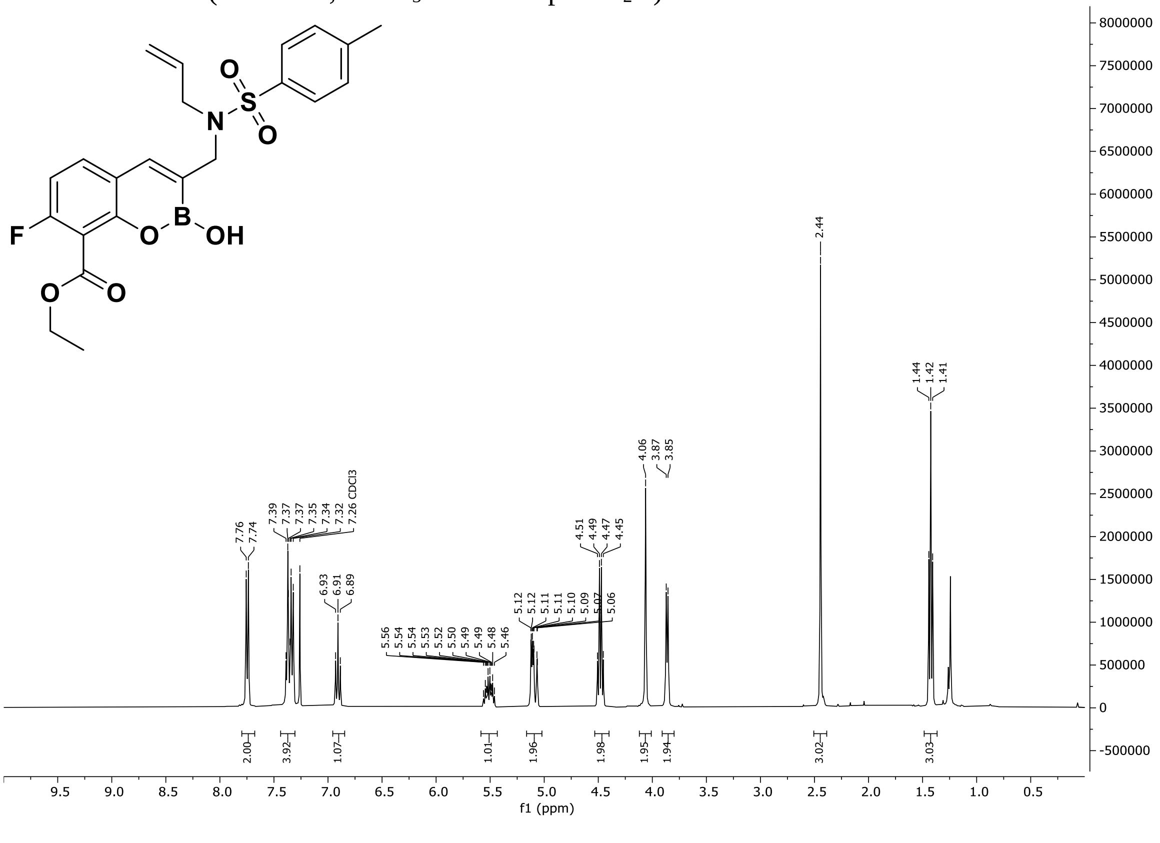

^{13}C-NMR of S42 (100 MHz, $CDCl_3$ with a drop of D_2O)

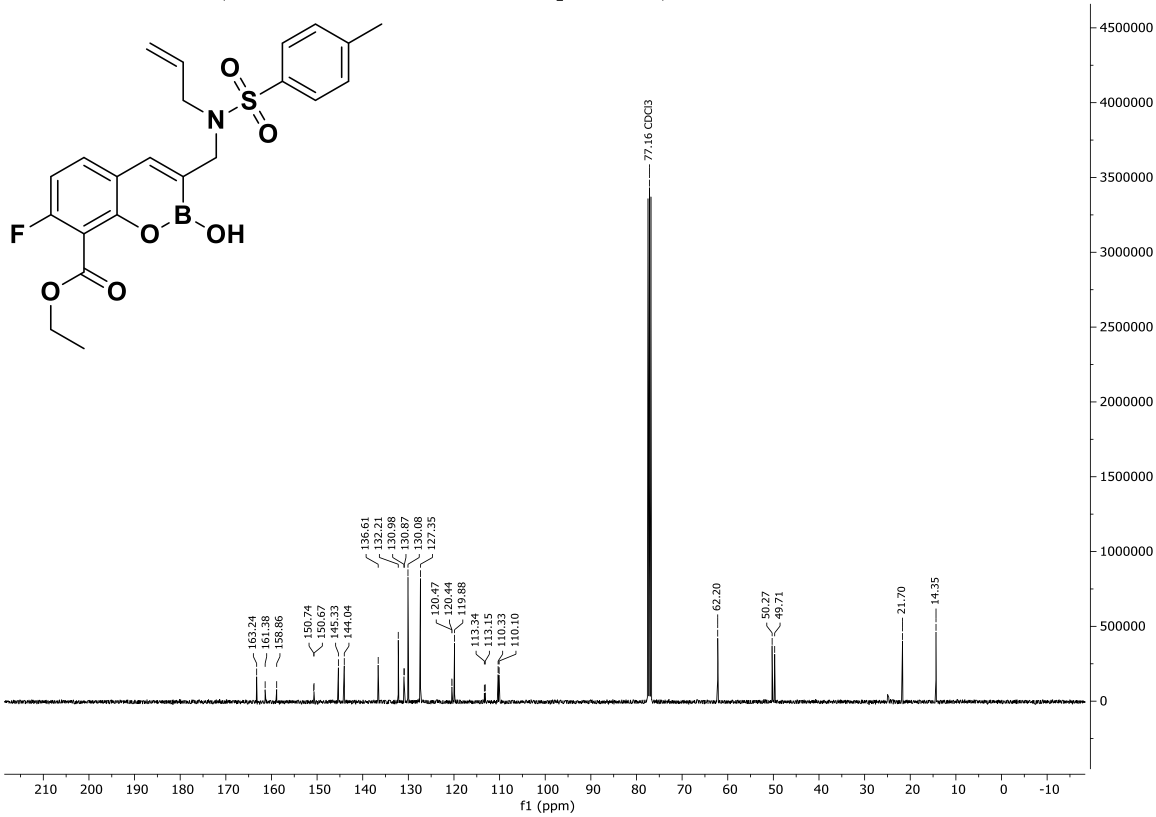

^{11}B-NMR of S42 (128 MHz $CDCl_3$ with a drop of D_2O)

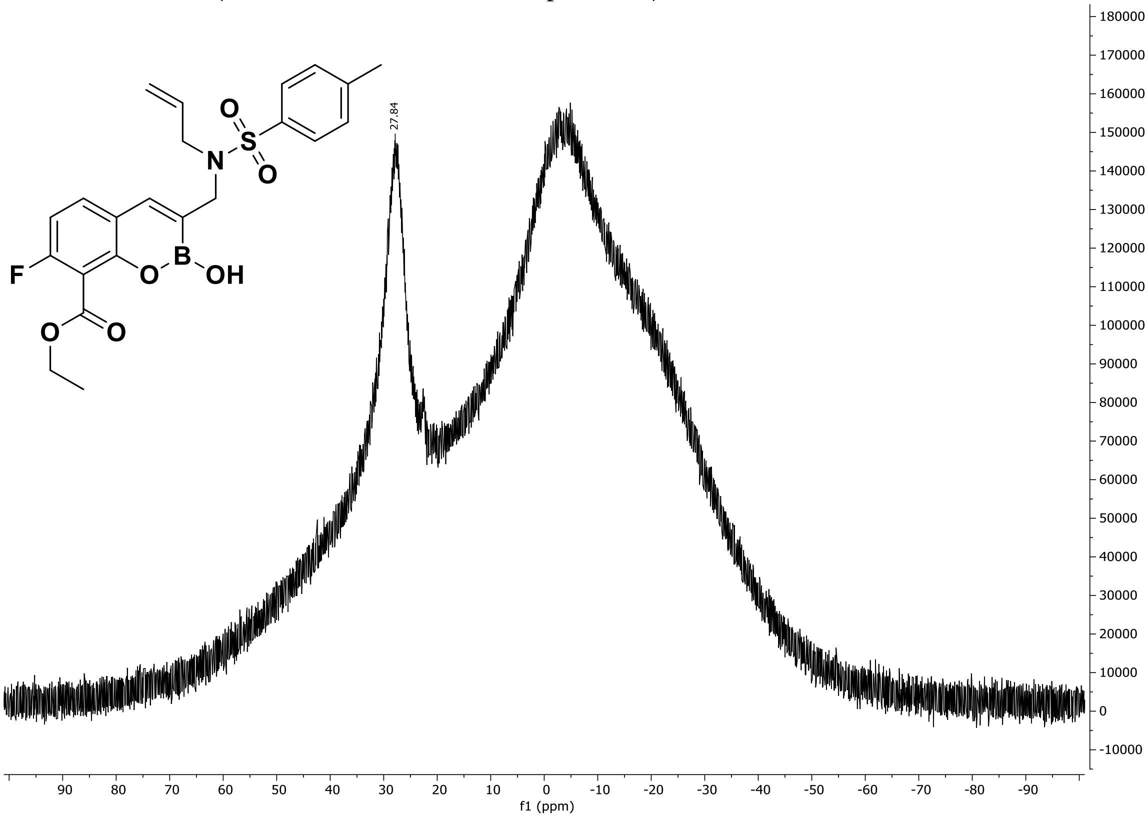

^{19}F-NMR of S42 (376 MHz $CDCl_3$ with a drop of D_2O)

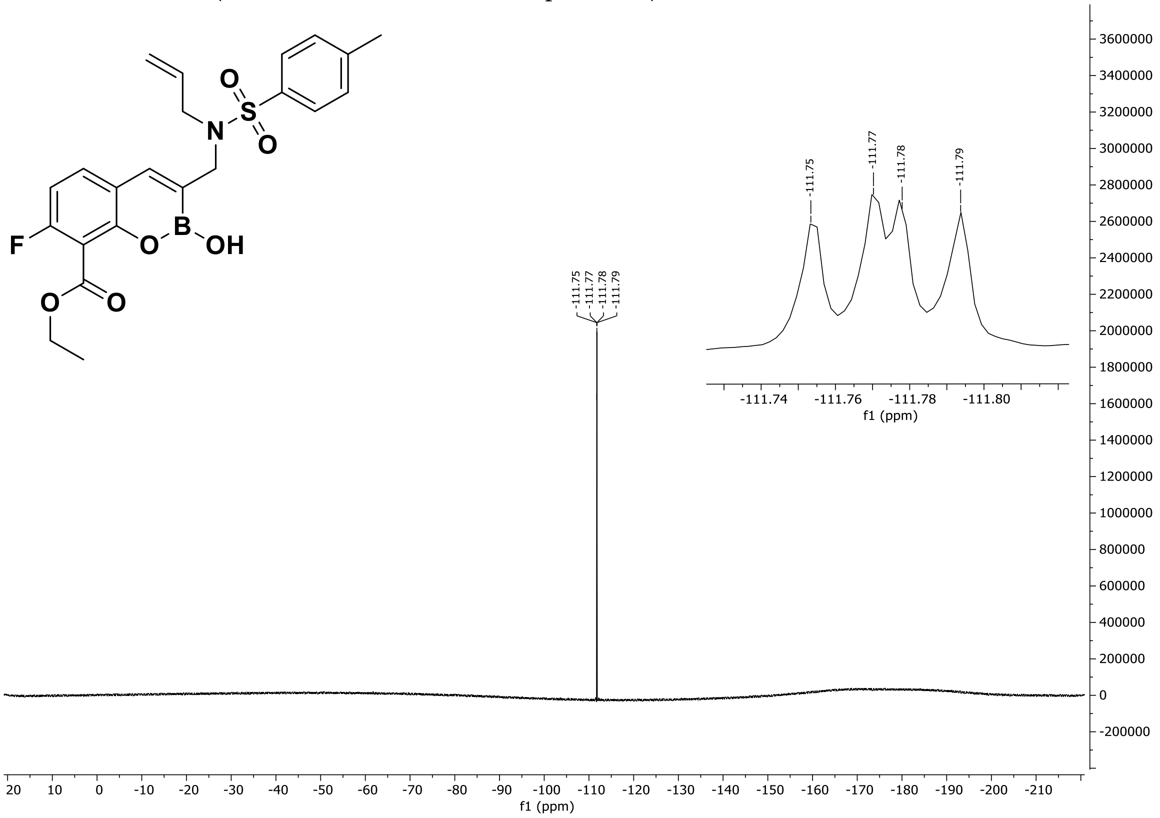

^{1}H-NMR of S43 (400 MHz, $CDCl_3$)

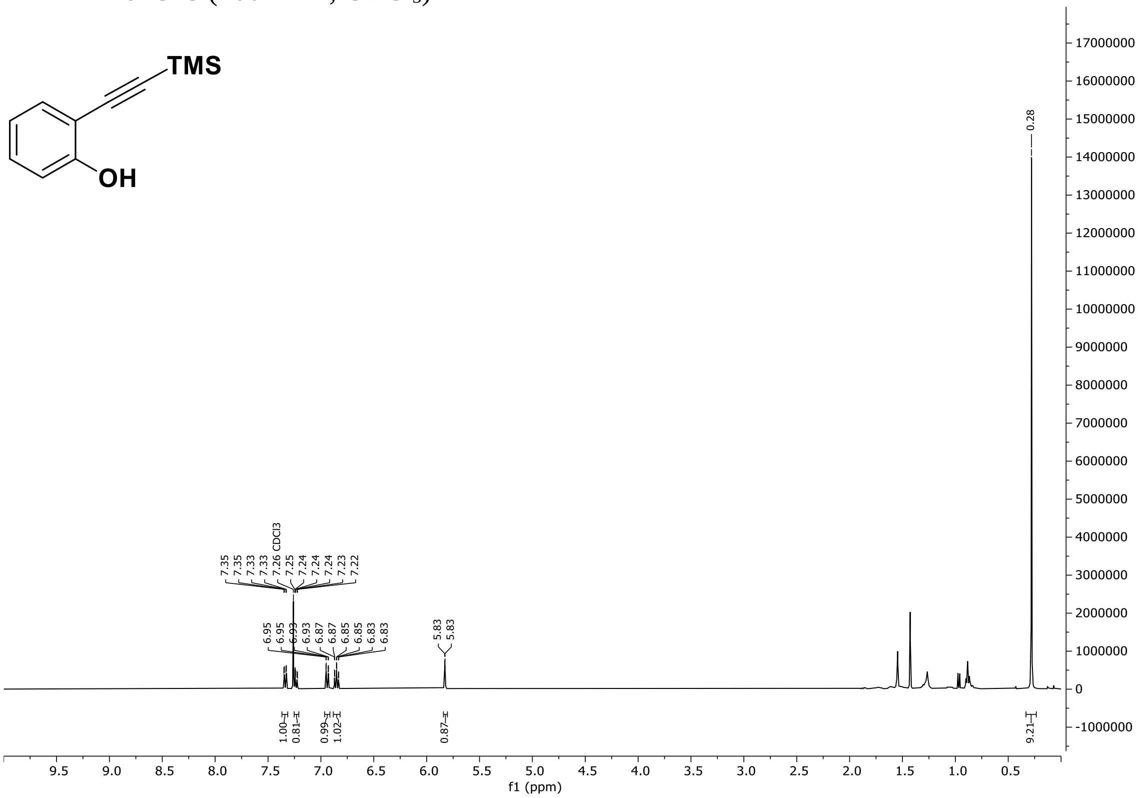

^{1}H-NMR of S44 (400 MHz, $CDCl_3$)

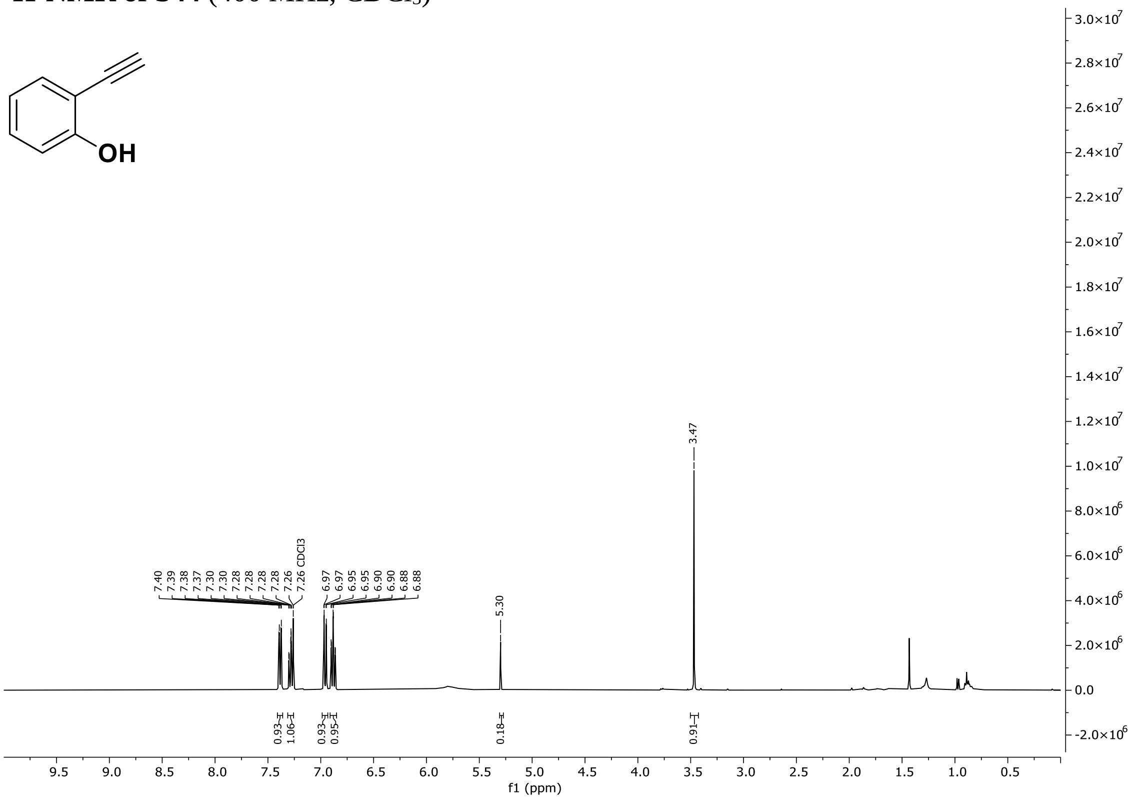

^{1}H-NMR of S46 (400 MHz, $CDCl_3$)

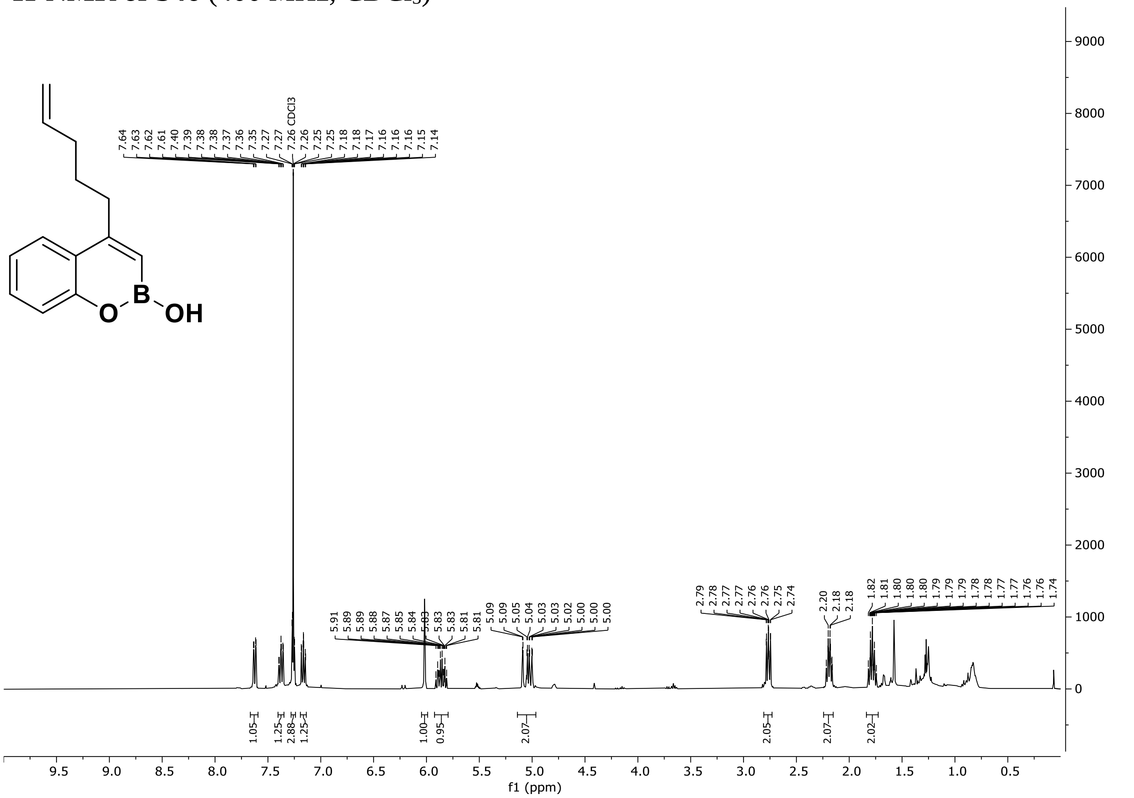

^{13}C-NMR of S46 (101 MHz, $CDCl_3$)

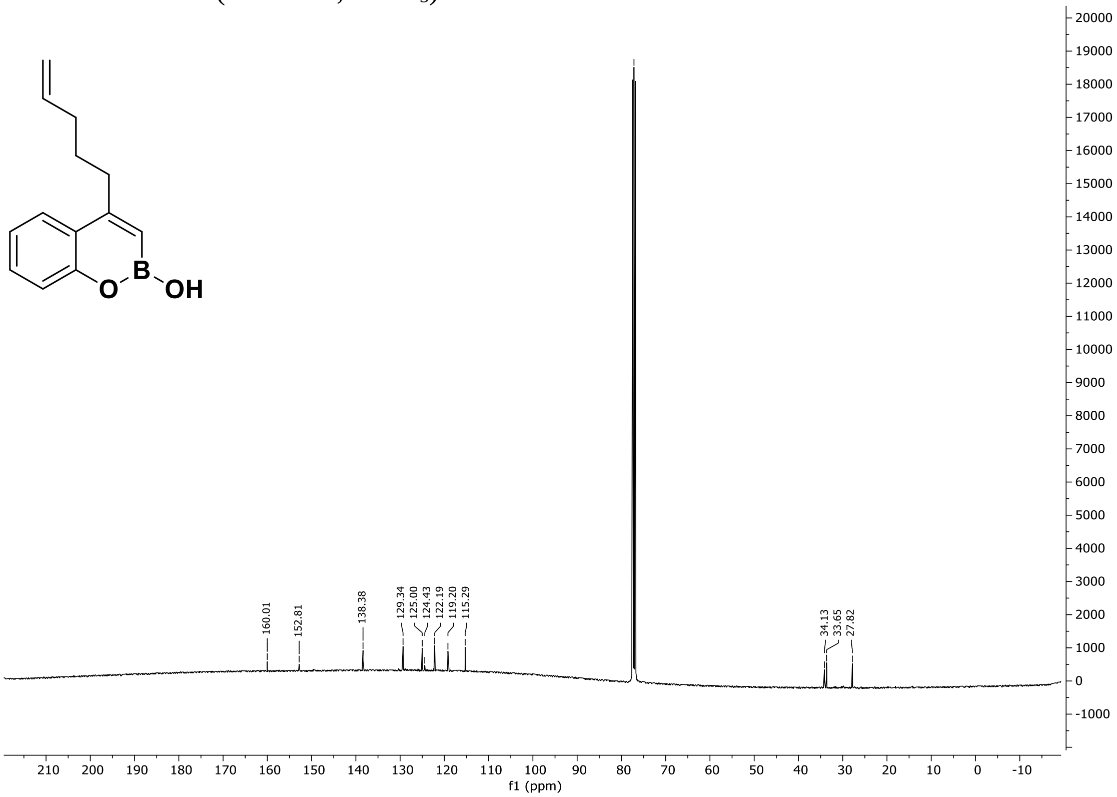

^{11}B-NMR of S46 (128 MHz, $CDCl_3$)

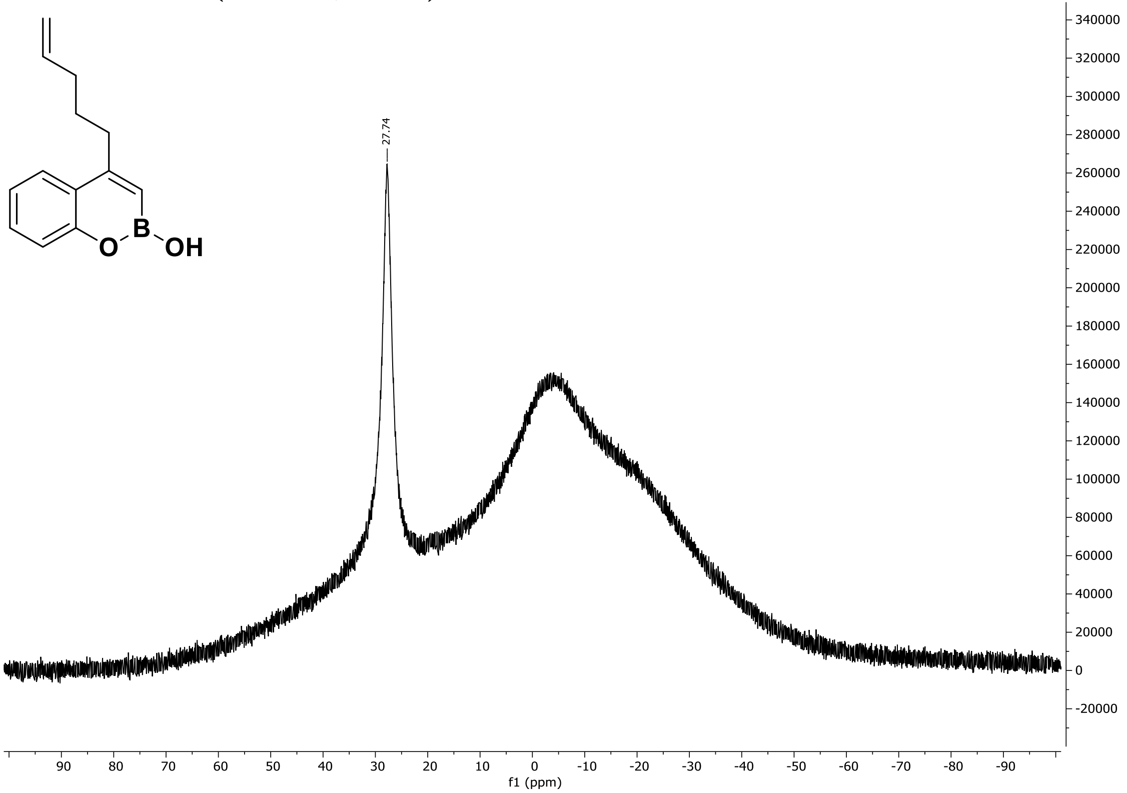

¹H-NMR of S47 (400 MHz, DMSO-*d*6)

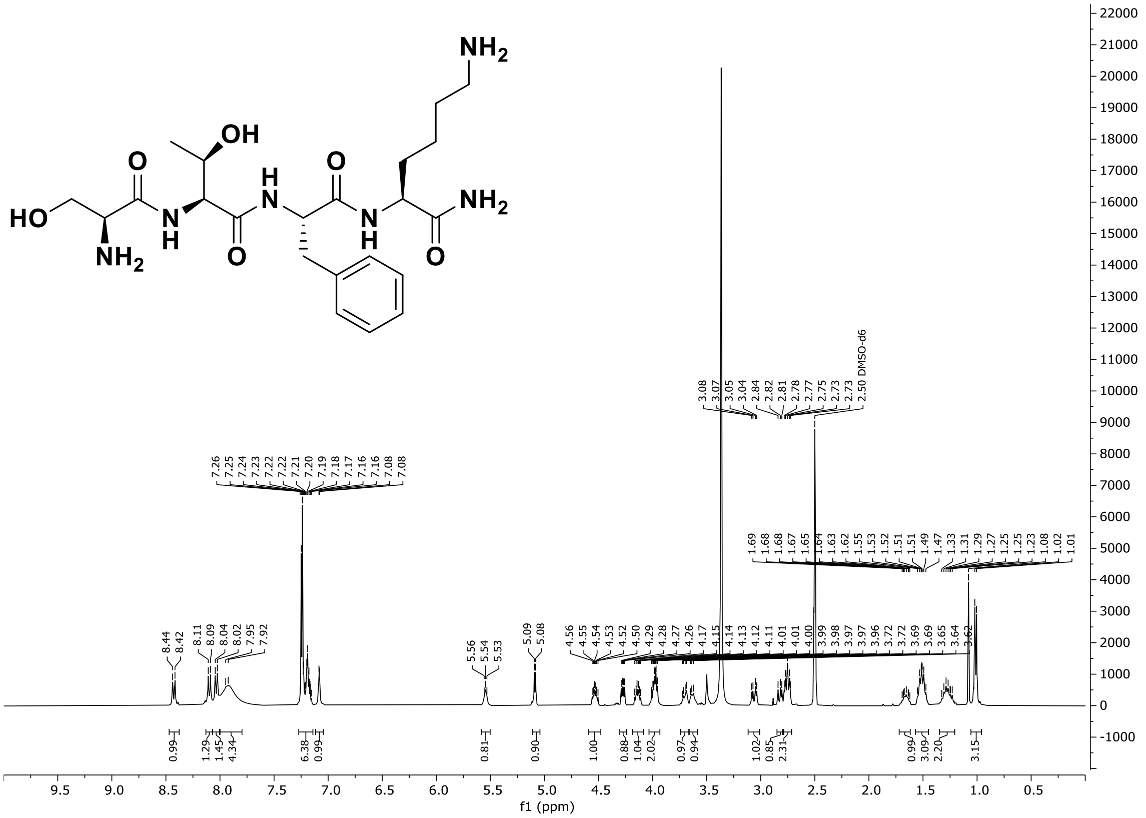

¹³C-NMR of S46 (101 MHz, DMSO-*d*6)

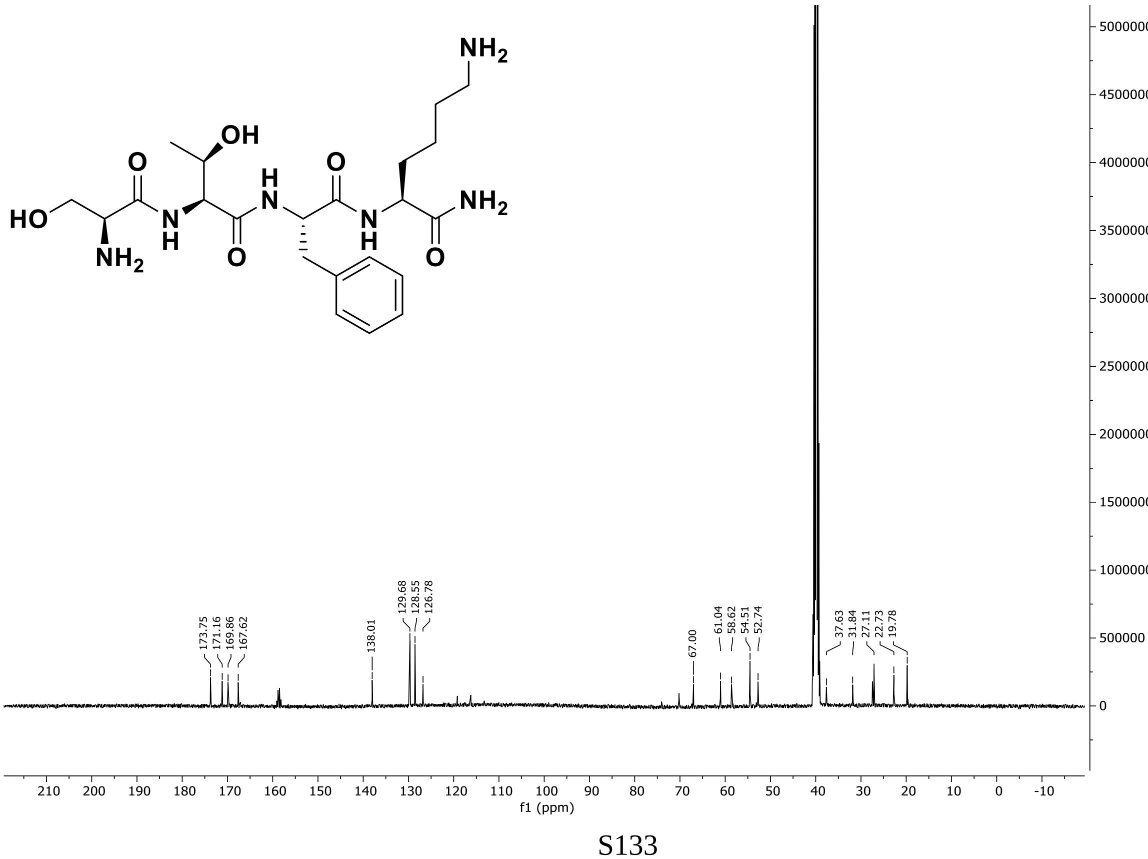

HPLC of S47 (RP18, detection wavelength: 214 nm)

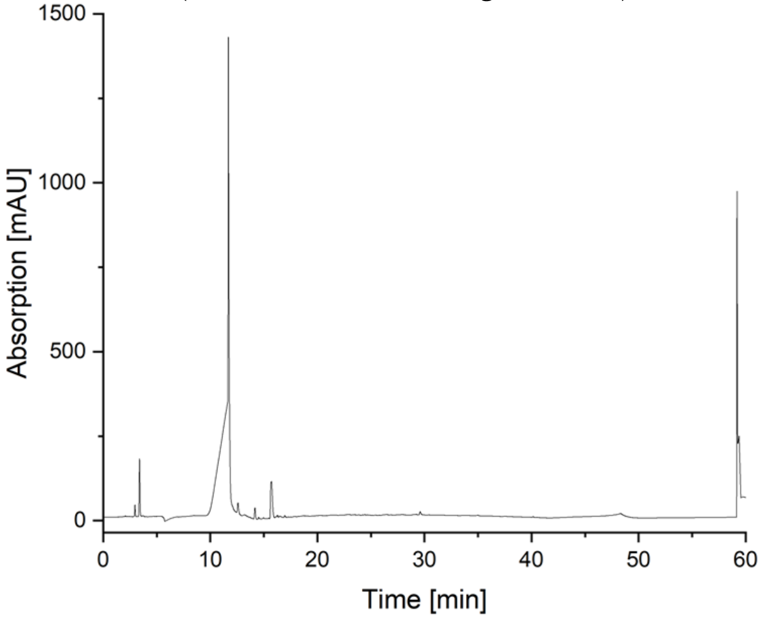

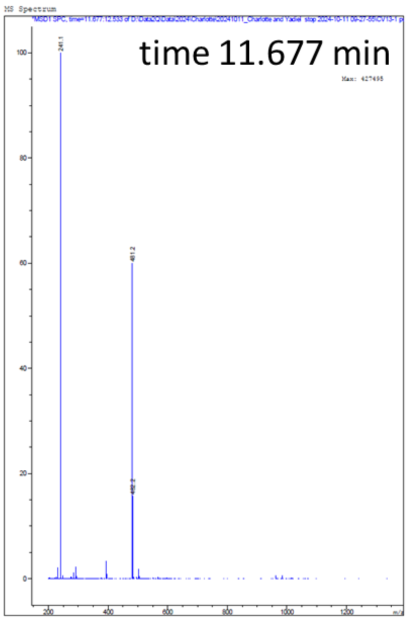

MALDI of S47

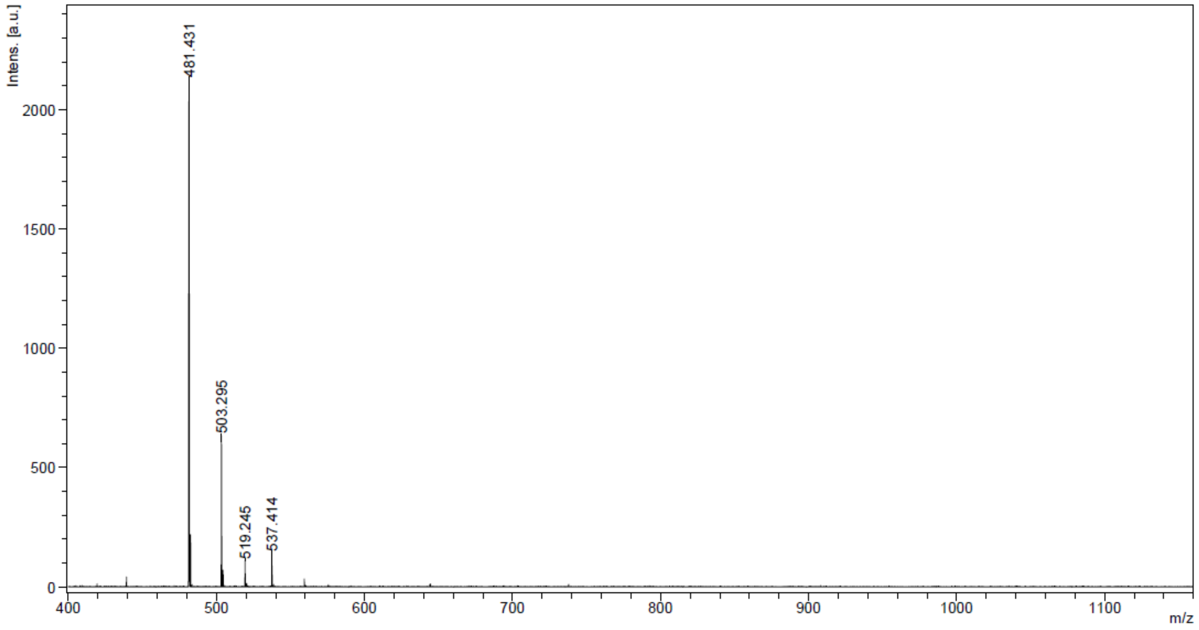

^{1}H-NMR of 2 (400 MHz, $CDCl_3$)

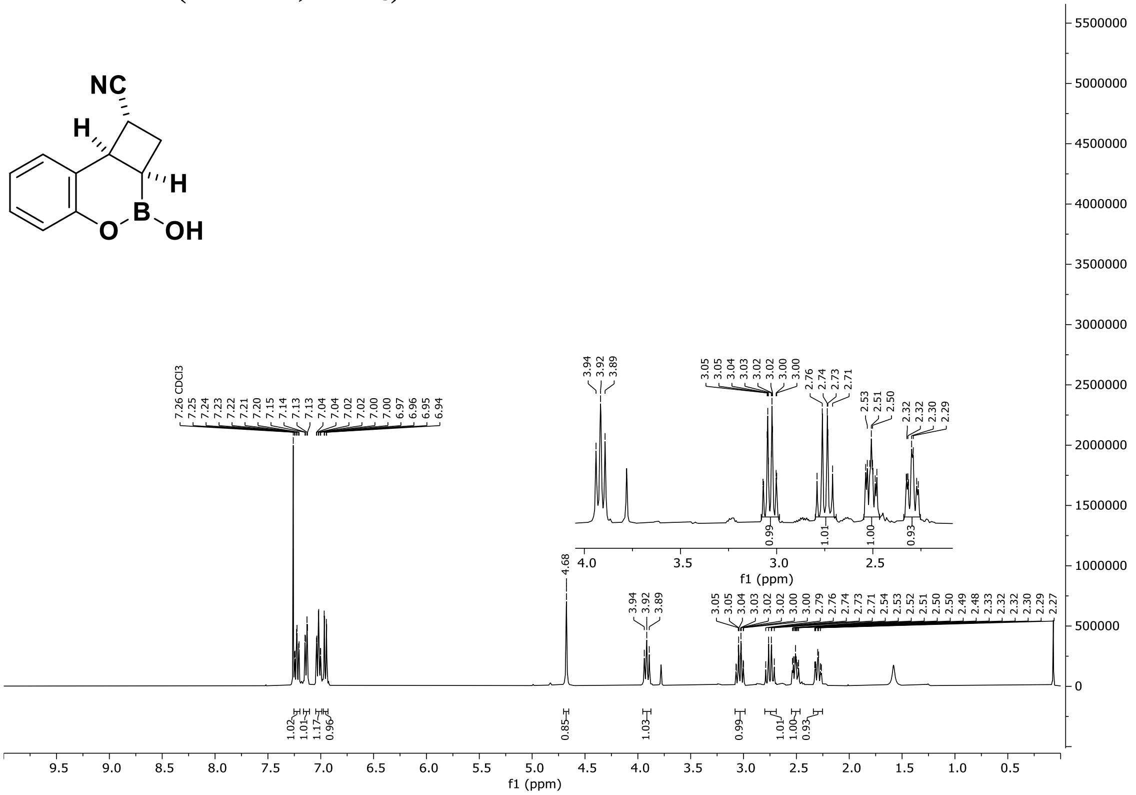

^{13}C-NMR of 2 (100 MHz, Acetone-d_6)

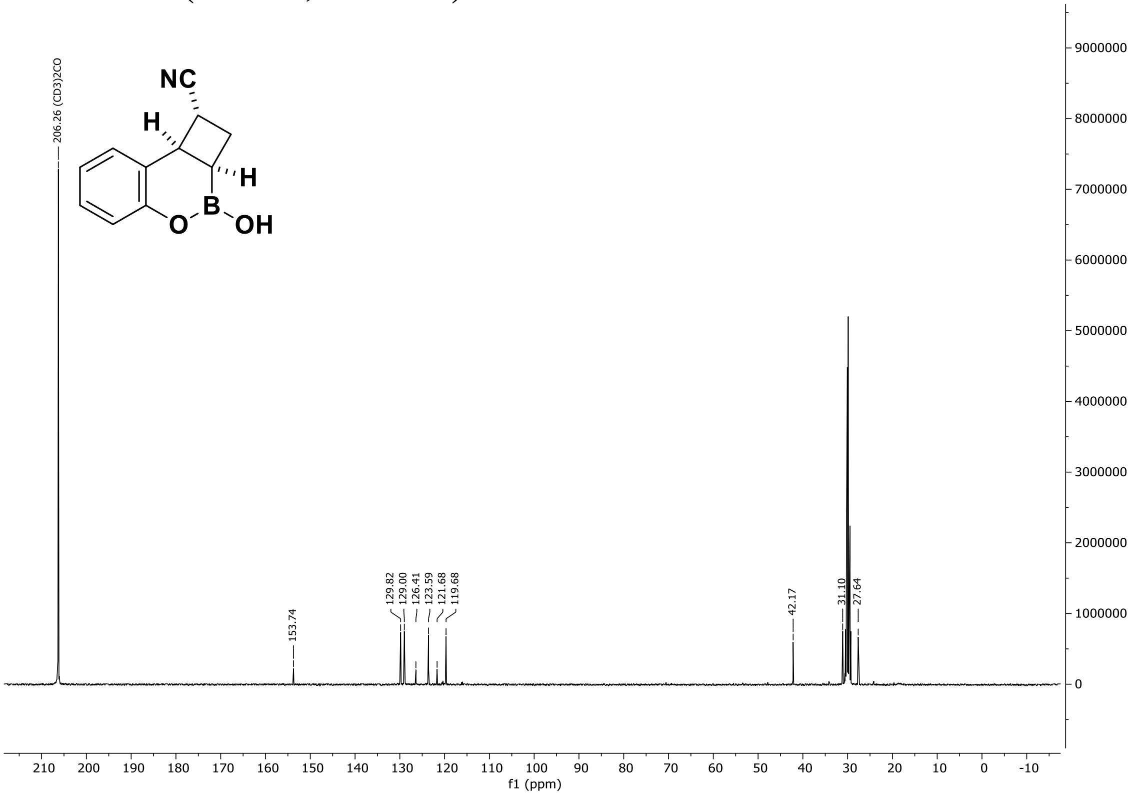

^{11}B-NMR of 2 (128 MHz, $CDCl_3$)

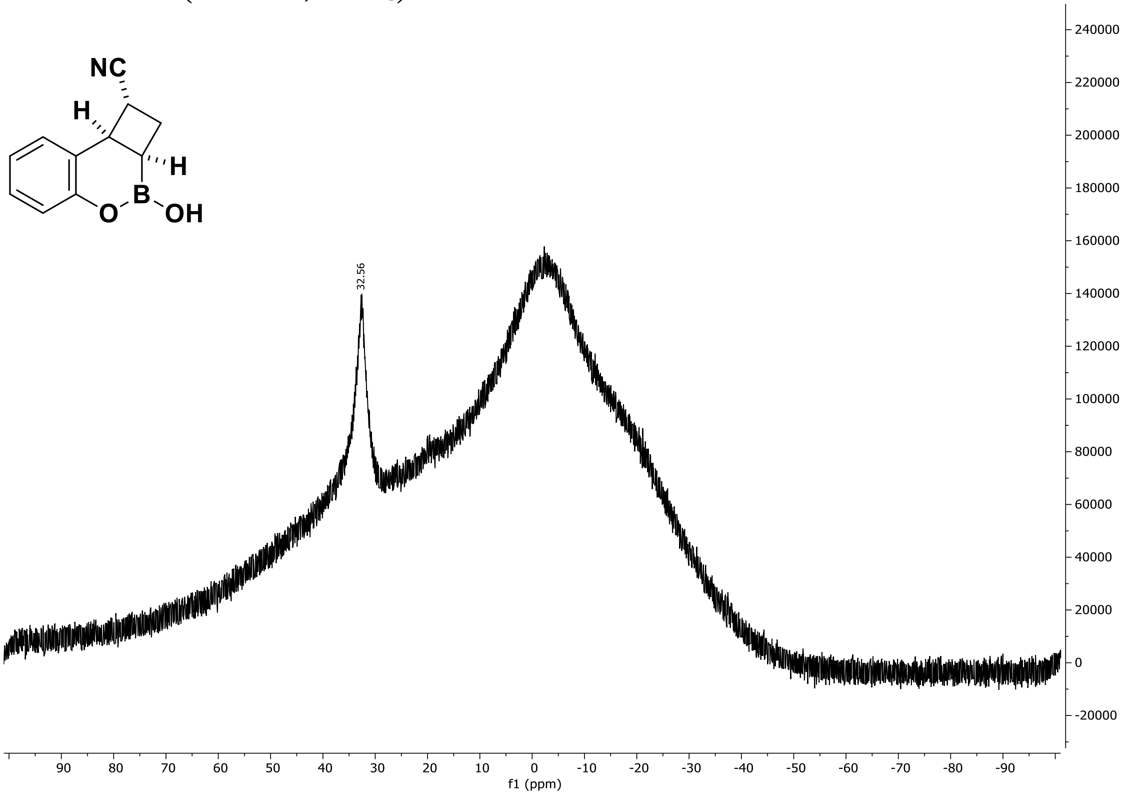

^{1}H-NMR of 2-*syn* (400 MHz, Acetone-d_6)

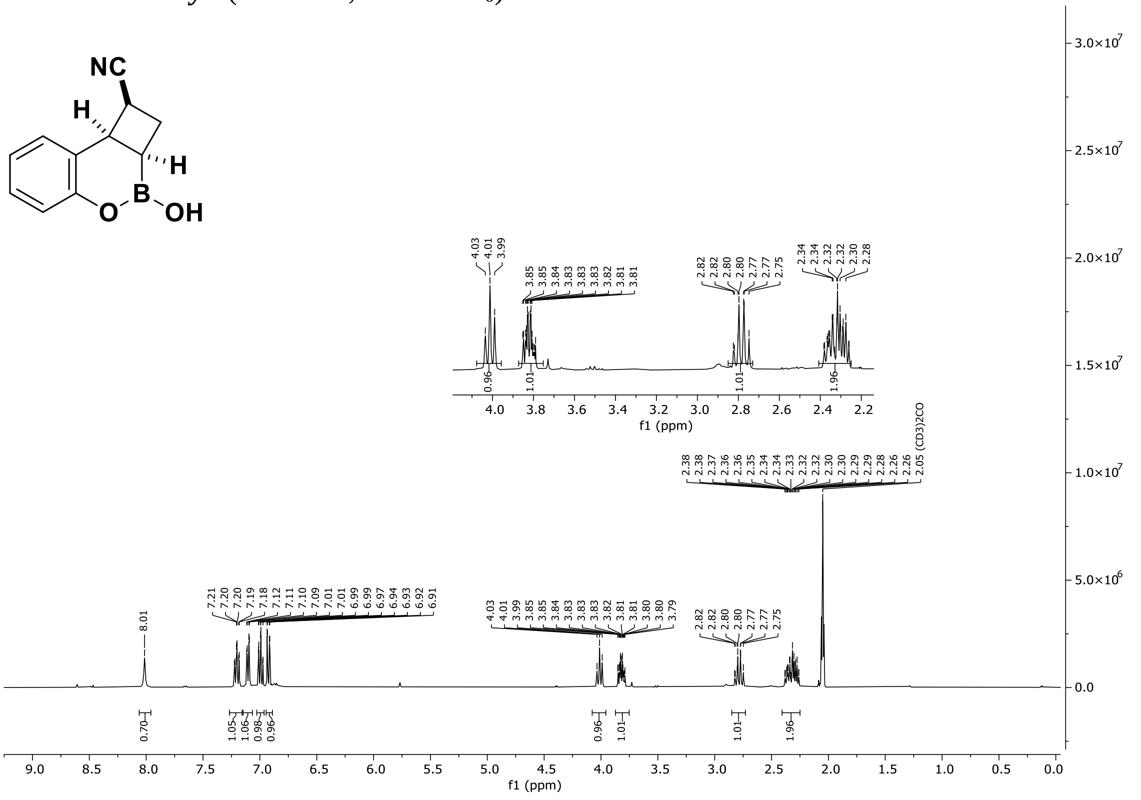

^{13}**C-NMR of 2-*syn*** (100 MHz, Acetone-d_6)

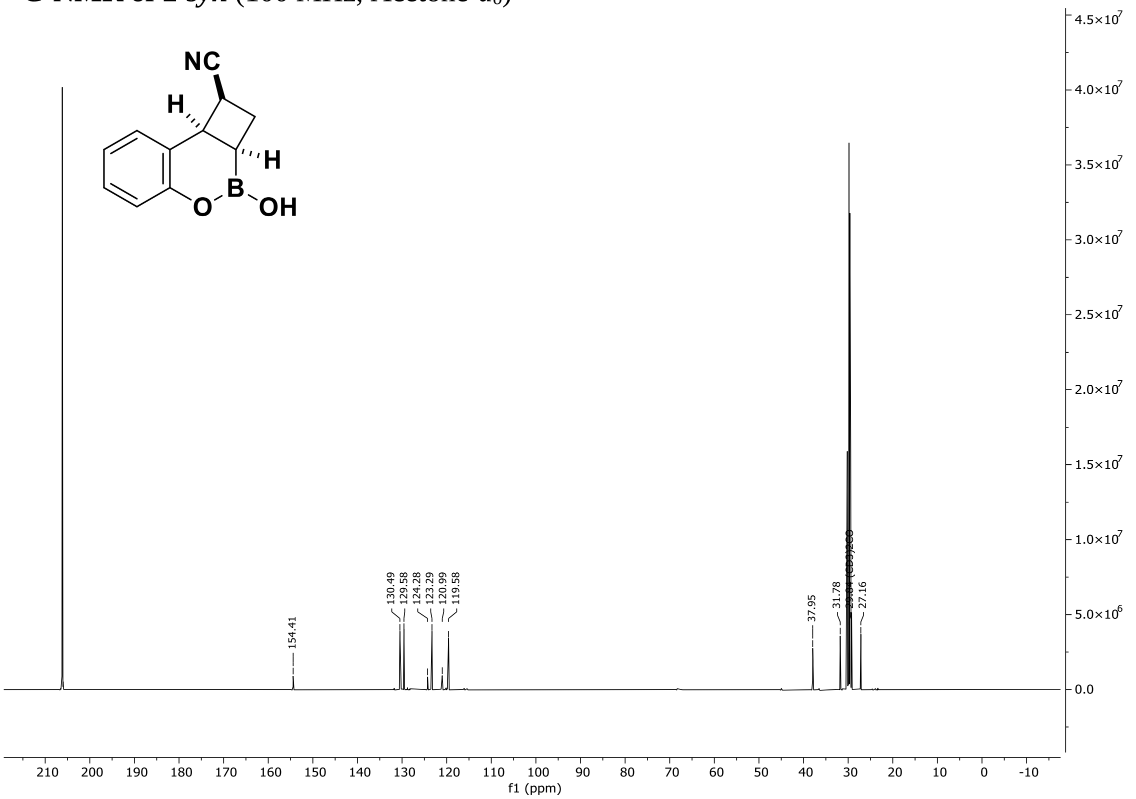

^{11}**B-NMR of 2-*syn*** (128 MHz, Acetone-d_6)

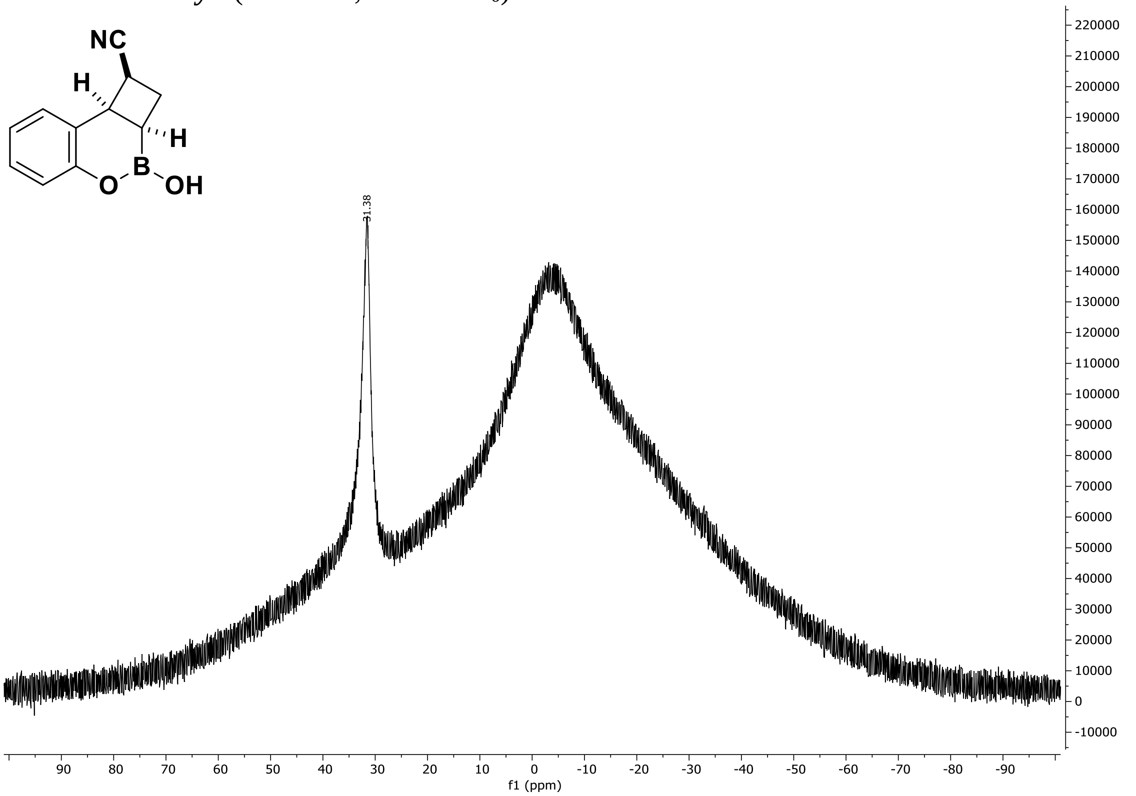

^{1}H-NMR of 4 (600 MHz, $CDCl_3$)

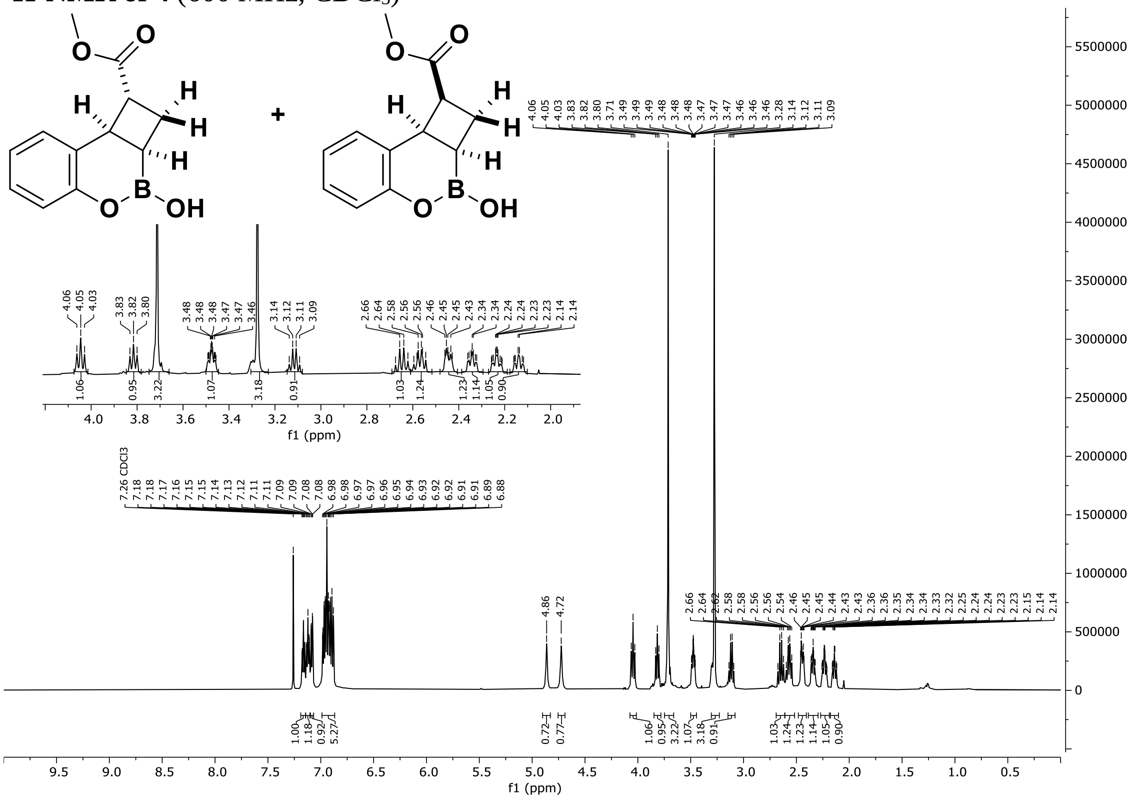

^{13}C-NMR of 4 (151 MHz, $CDCl_3$)

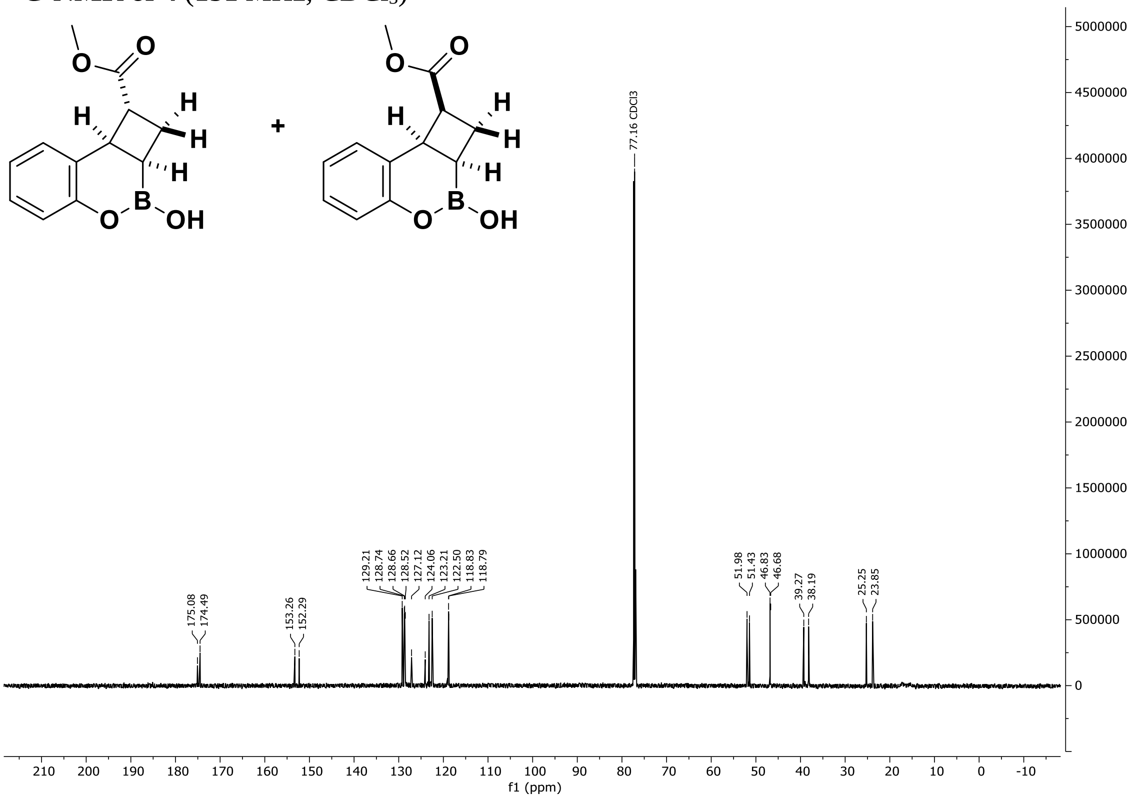

^{11}B-NMR of 4 (192MHz, $CDCl_3$)

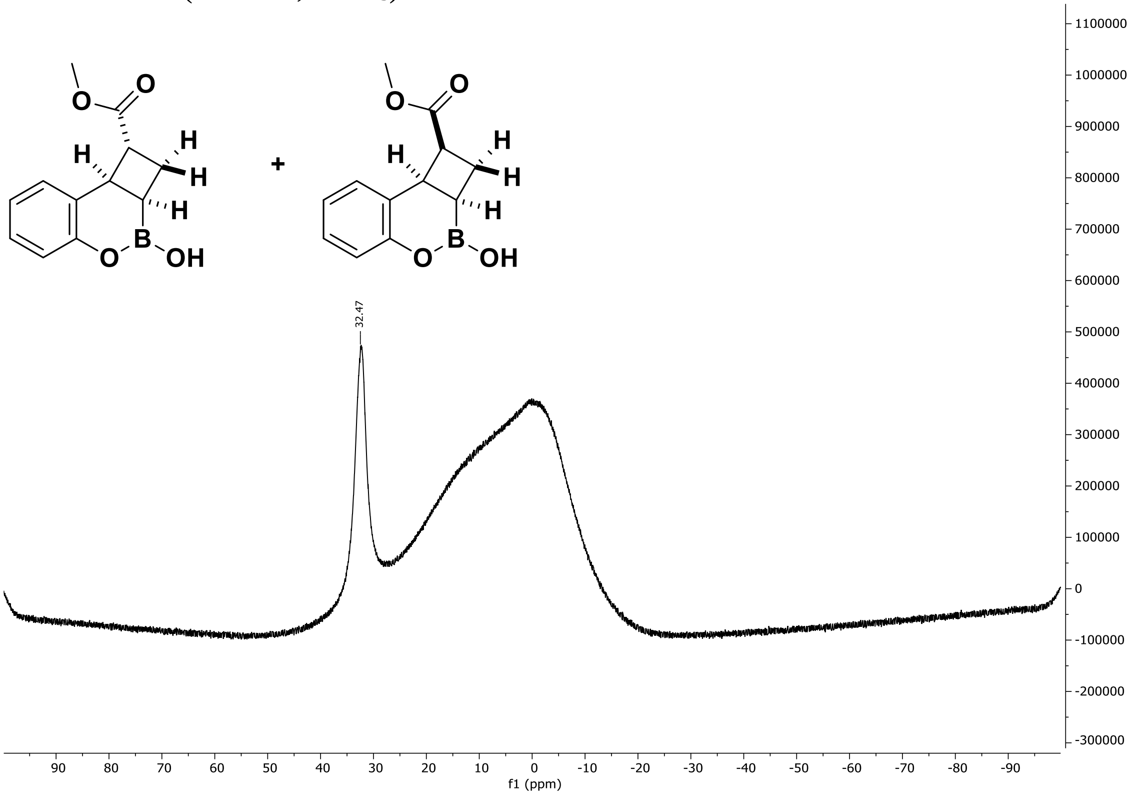

^{1}H-NMR of 5 (400 MHz, Acetone-d_6)

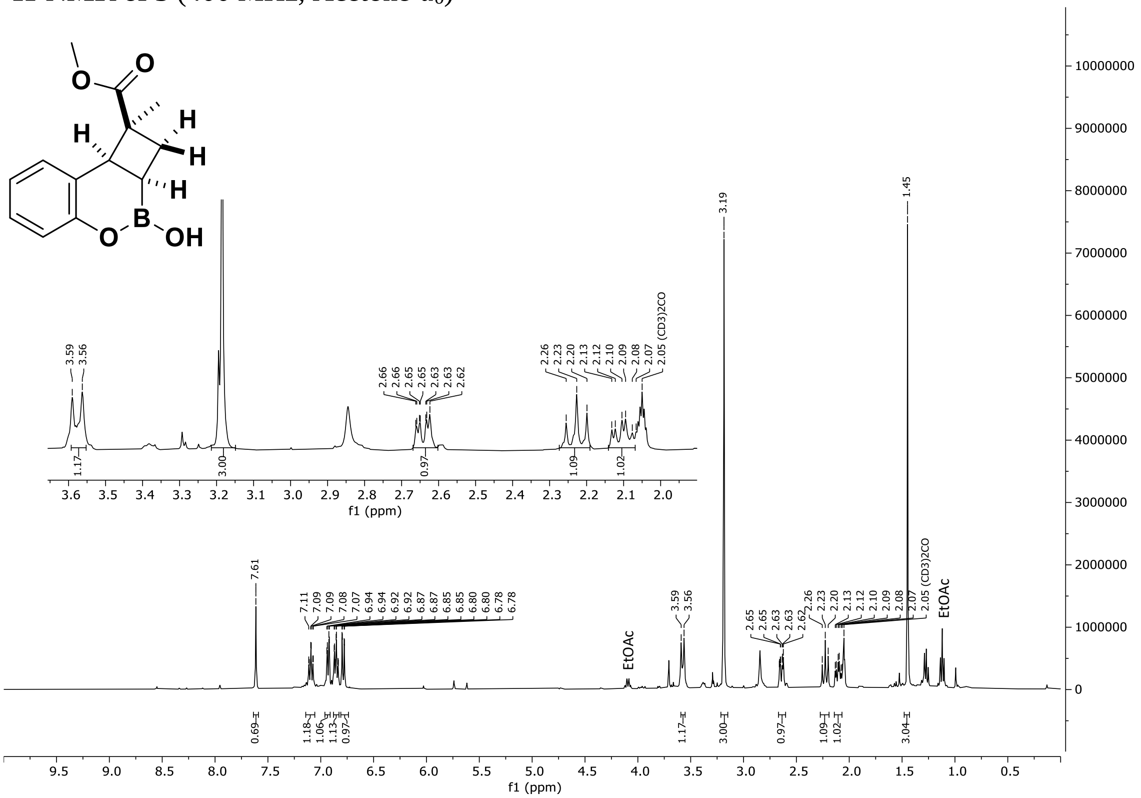

^{13}C-NMR of 5 (100 MHz, Acetone-d_6)

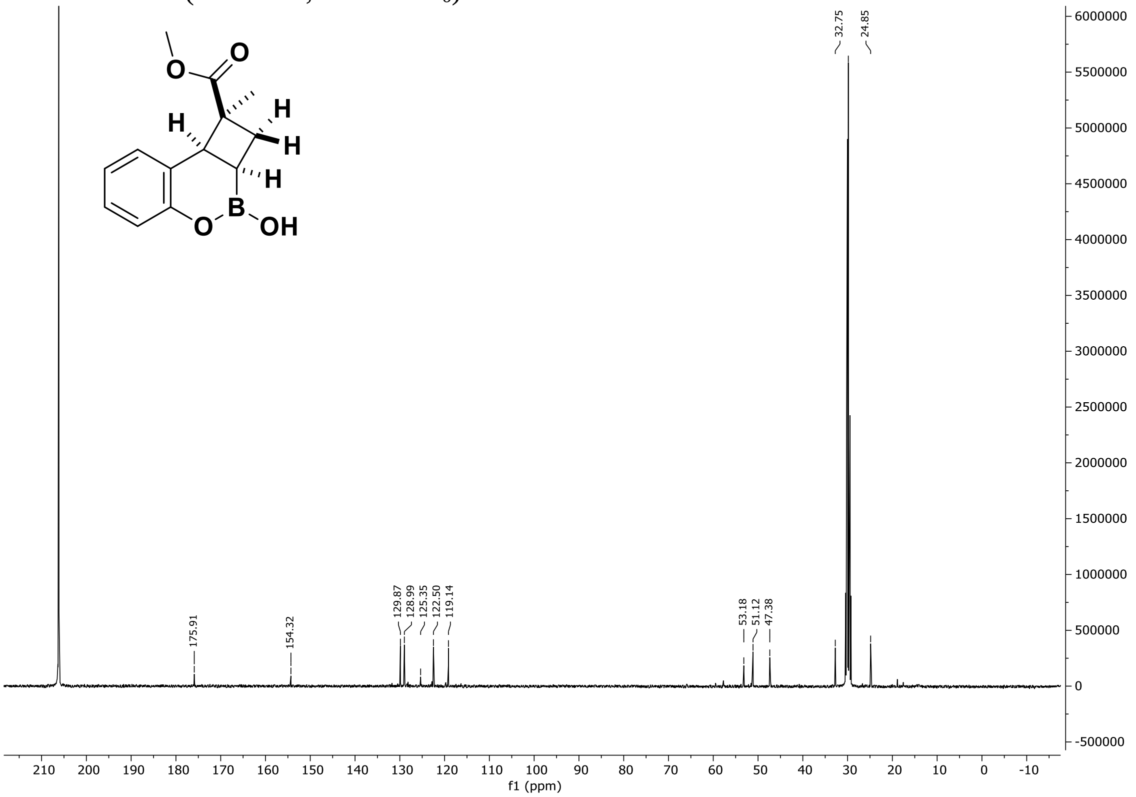

^{11}B-NMR of 5 (128 MHz, Acetone-d_6)

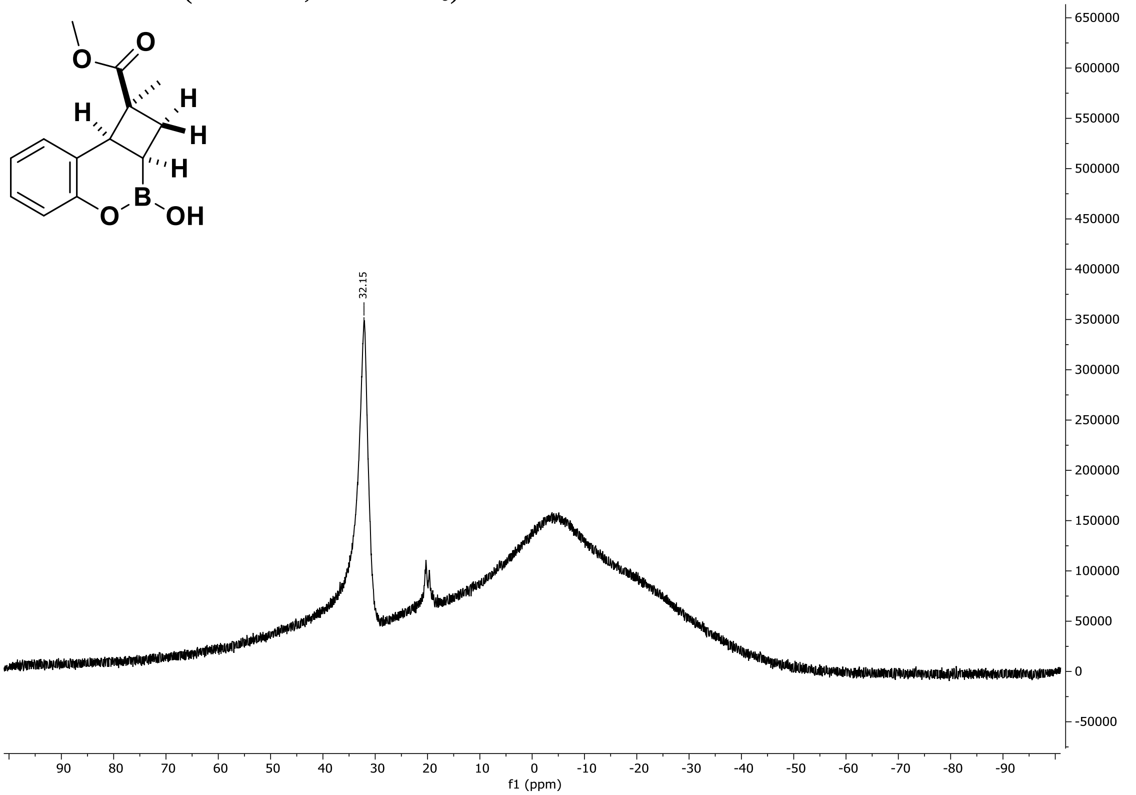

¹H-NMR of 6 (600 MHz, Acetone-d_6)

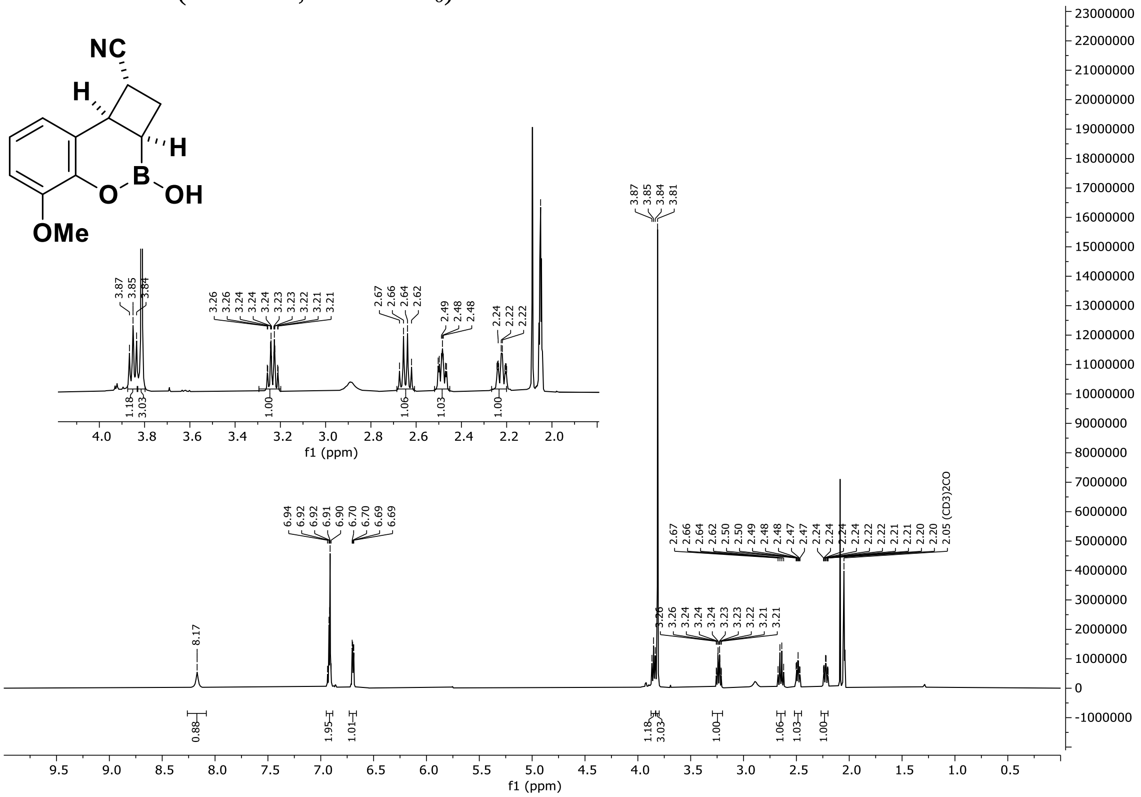

¹³C-NMR of 6 (100 MHz, Acetone-d_6)

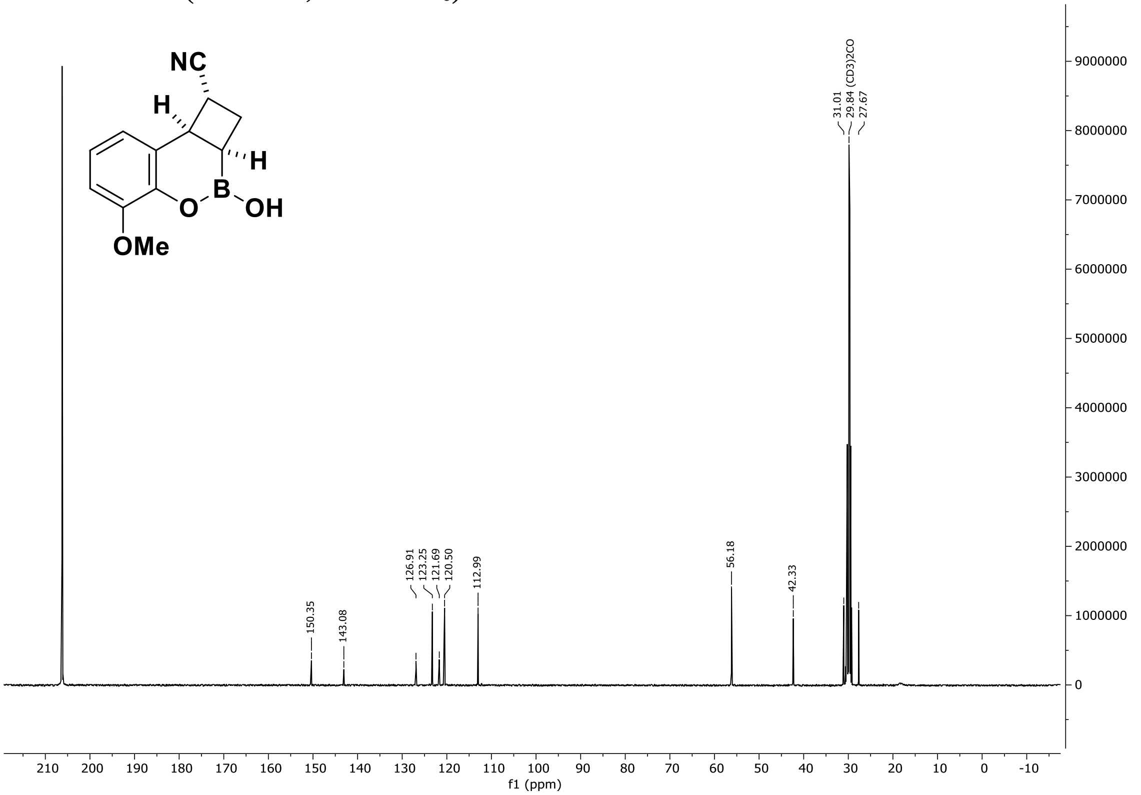

^{11}B-NMR of 6 (128 MHz, Acetone-d_6)

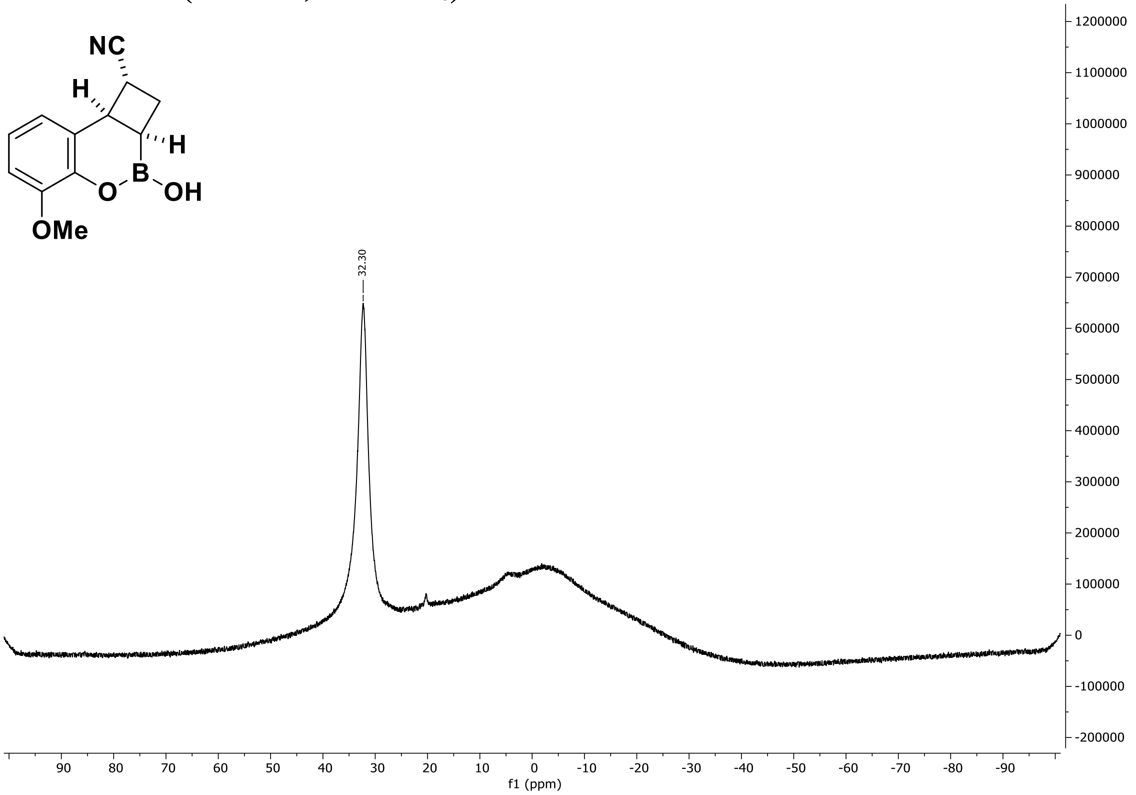

^{1}H-NMR of 7 (400 MHz, Acetone-d_6)

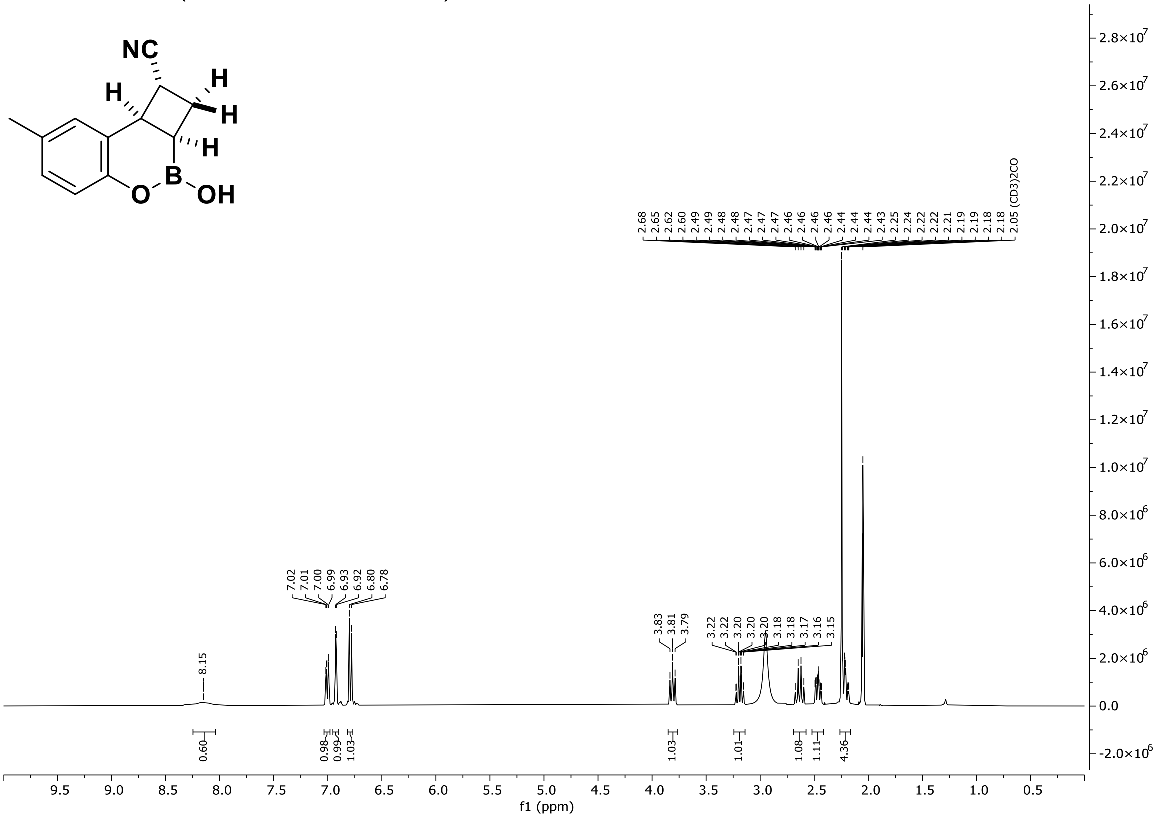

^{13}C-NMR of 7 (100 MHz, Acetone-d_6)

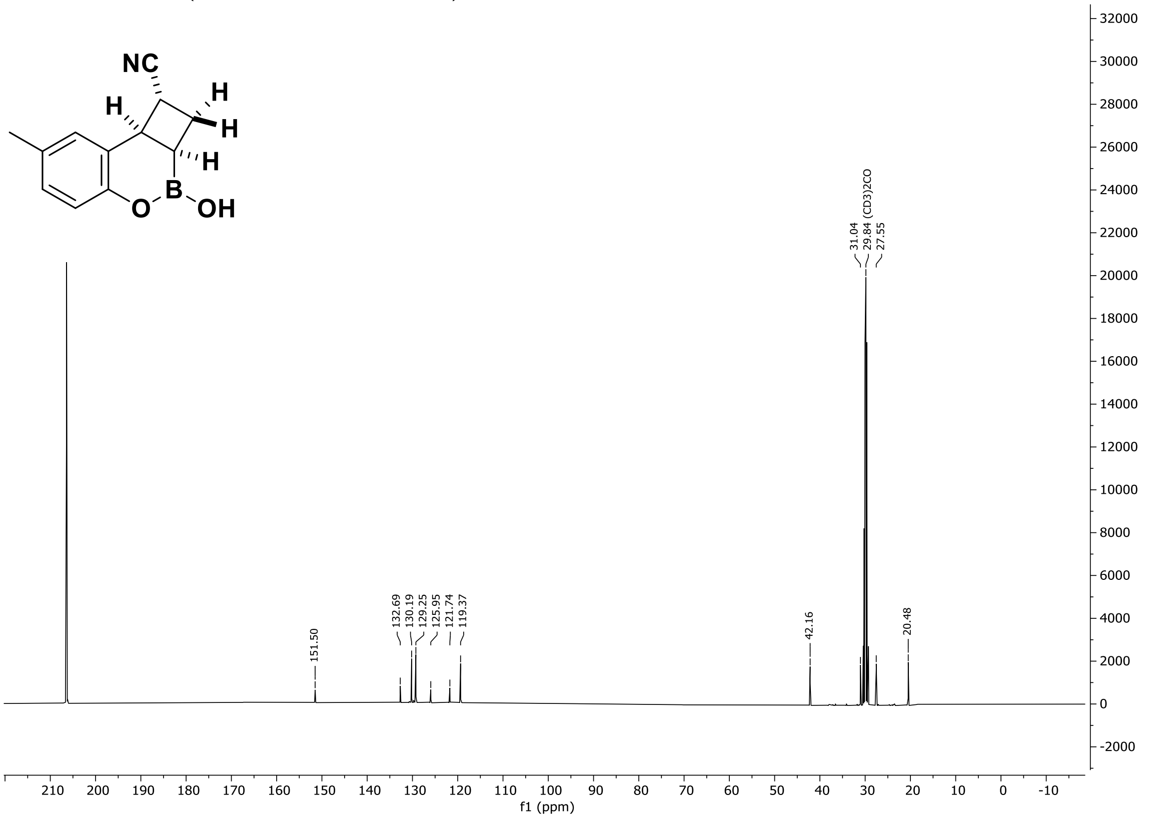

^{11}B-NMR of 7 (128 MHz, Acetone-d_6)

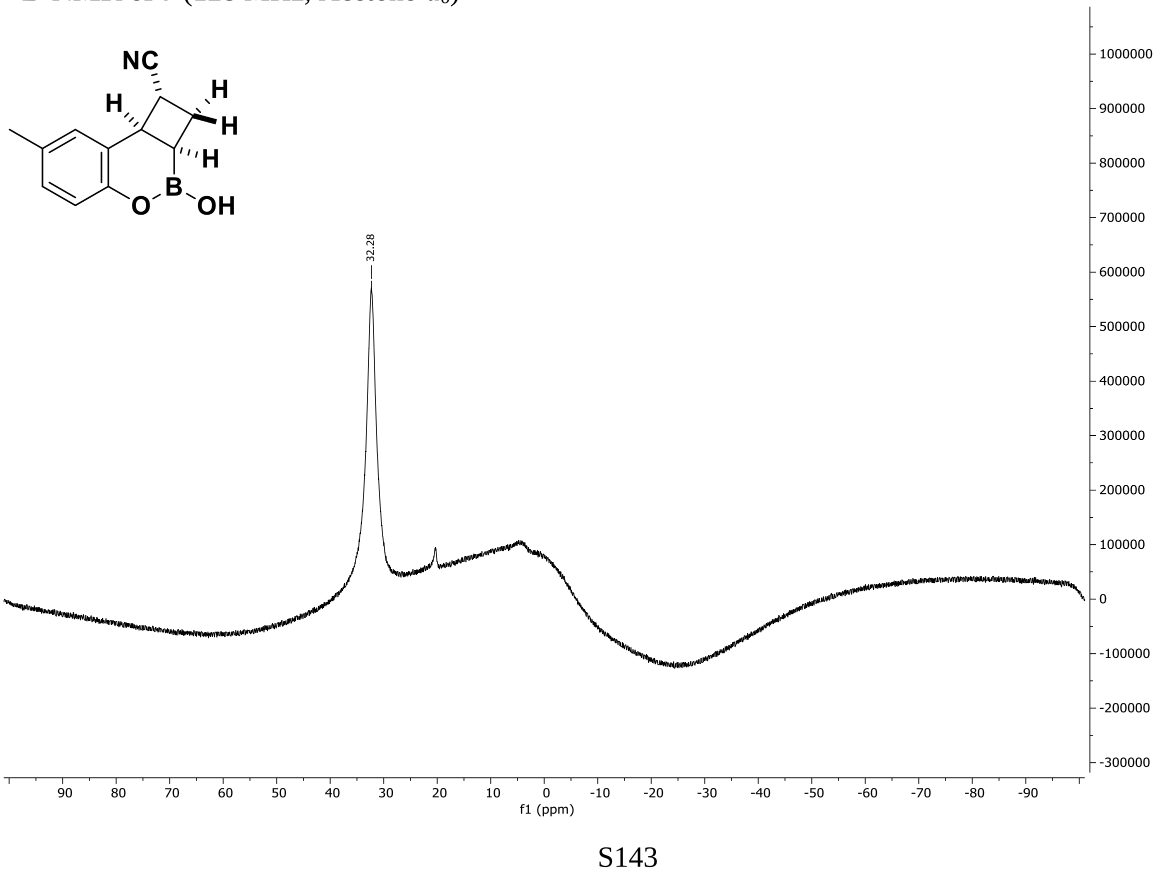

^{1}H-NMR of 7-*syn* (400 MHz, Acetone-d_6)

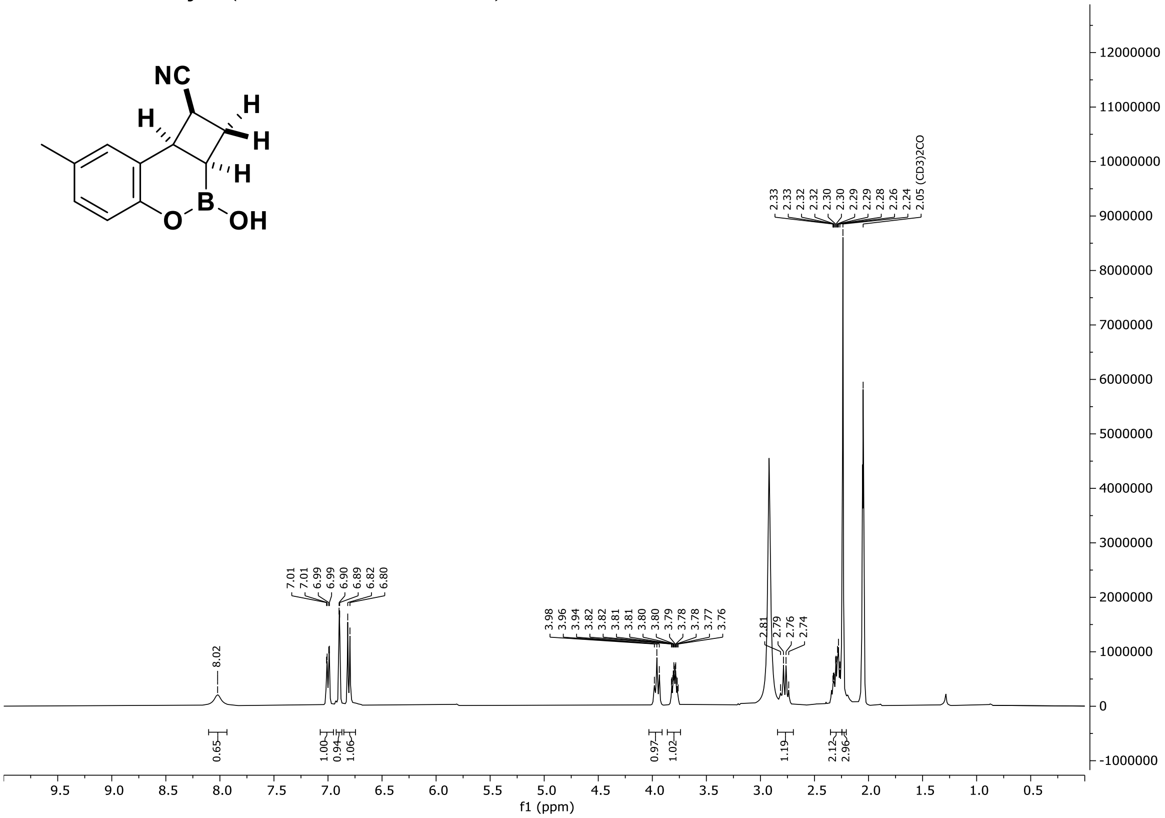

^{13}C-NMR of 7-*syn* (100 MHz, Acetone-d_6)

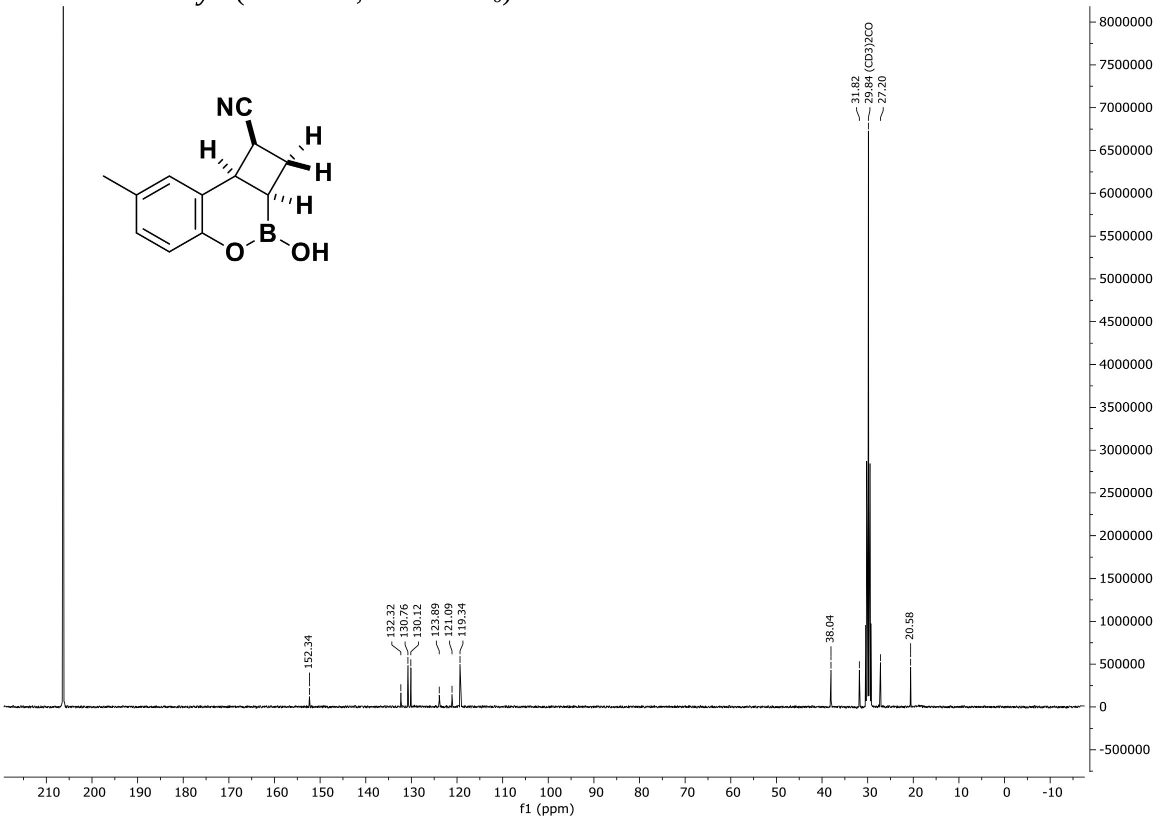

^{11}B-NMR of 7-*syn* (128 MHz, Acetone-d_6)

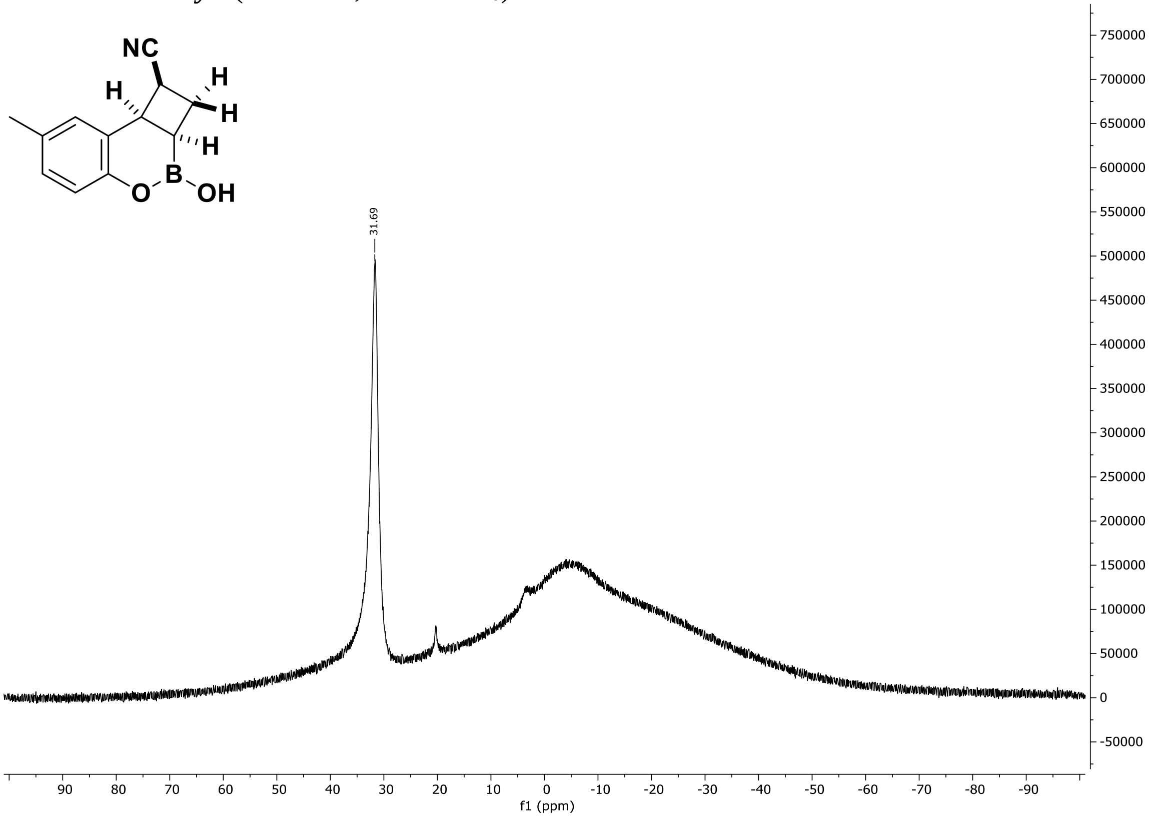

^{1}H-NMR of 8 (400 MHz, Acetone-d_6)

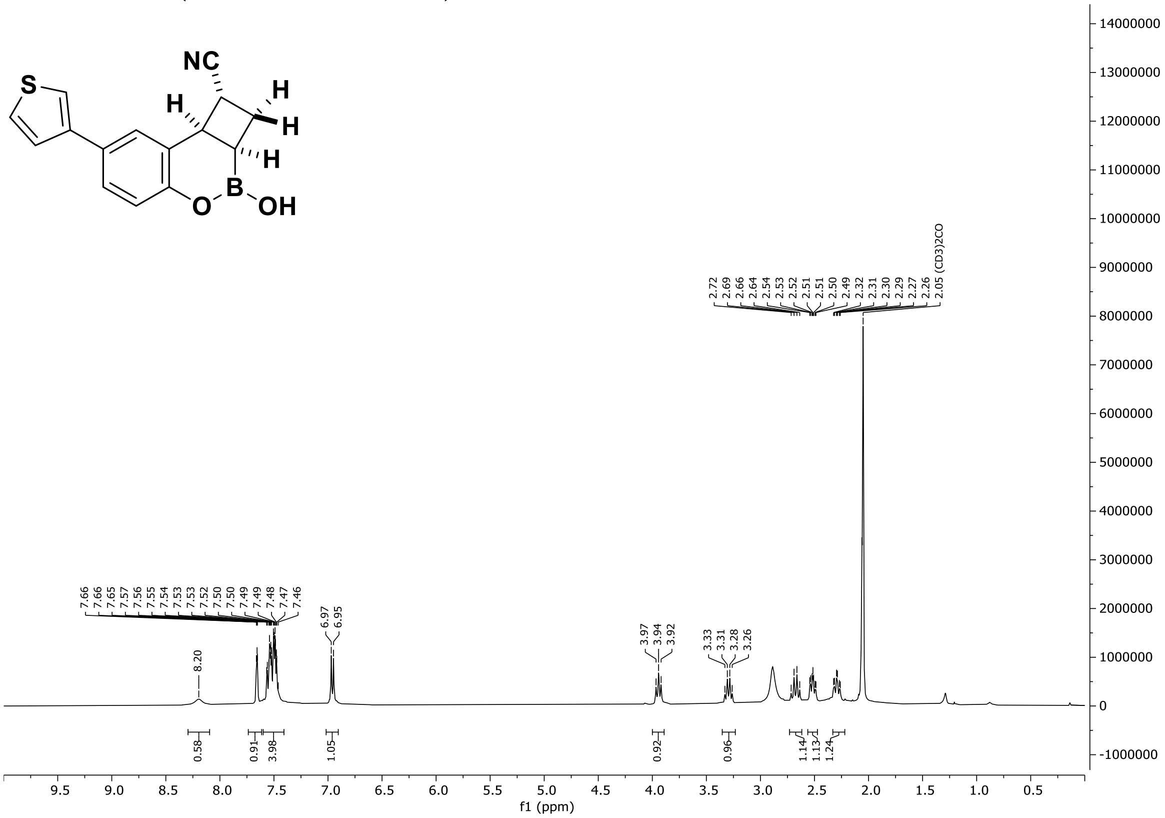

^{13}C-NMR of 8 (100 MHz, Acetone-d_6)

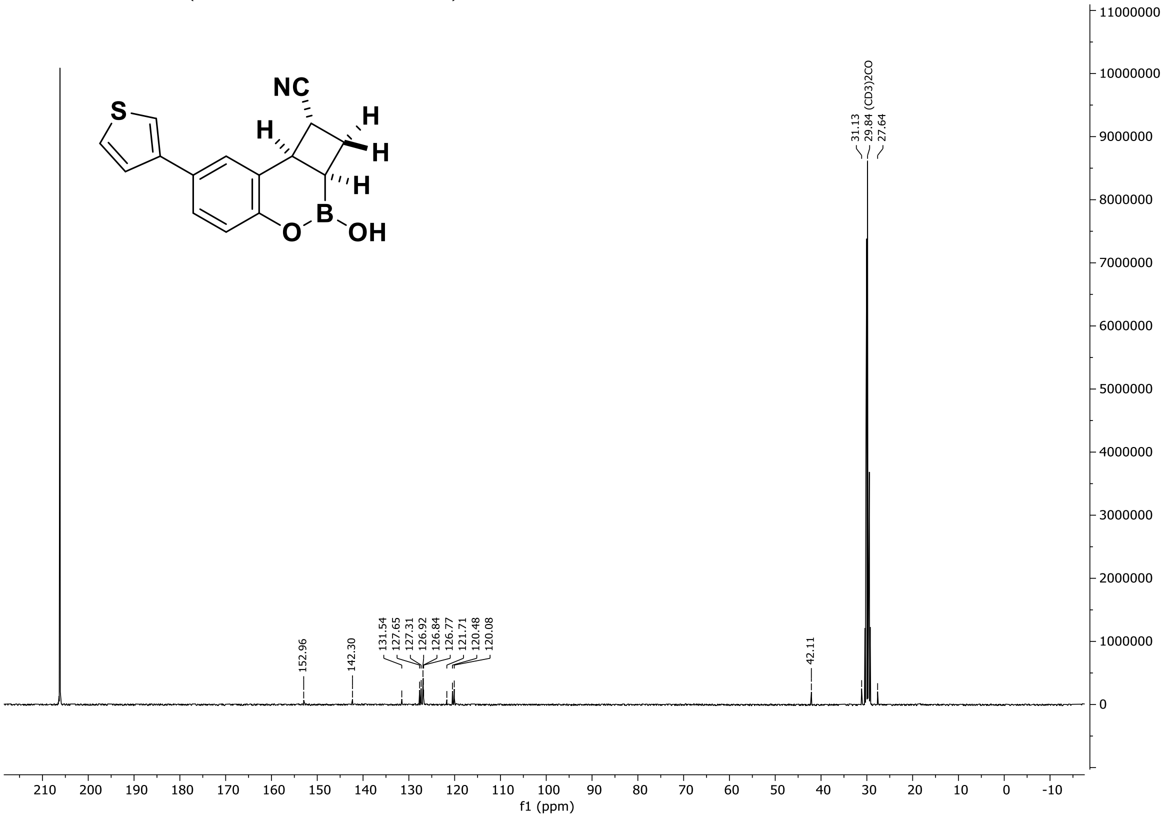

^{11}B-NMR of 8 (128 MHz, Acetone-d_6)

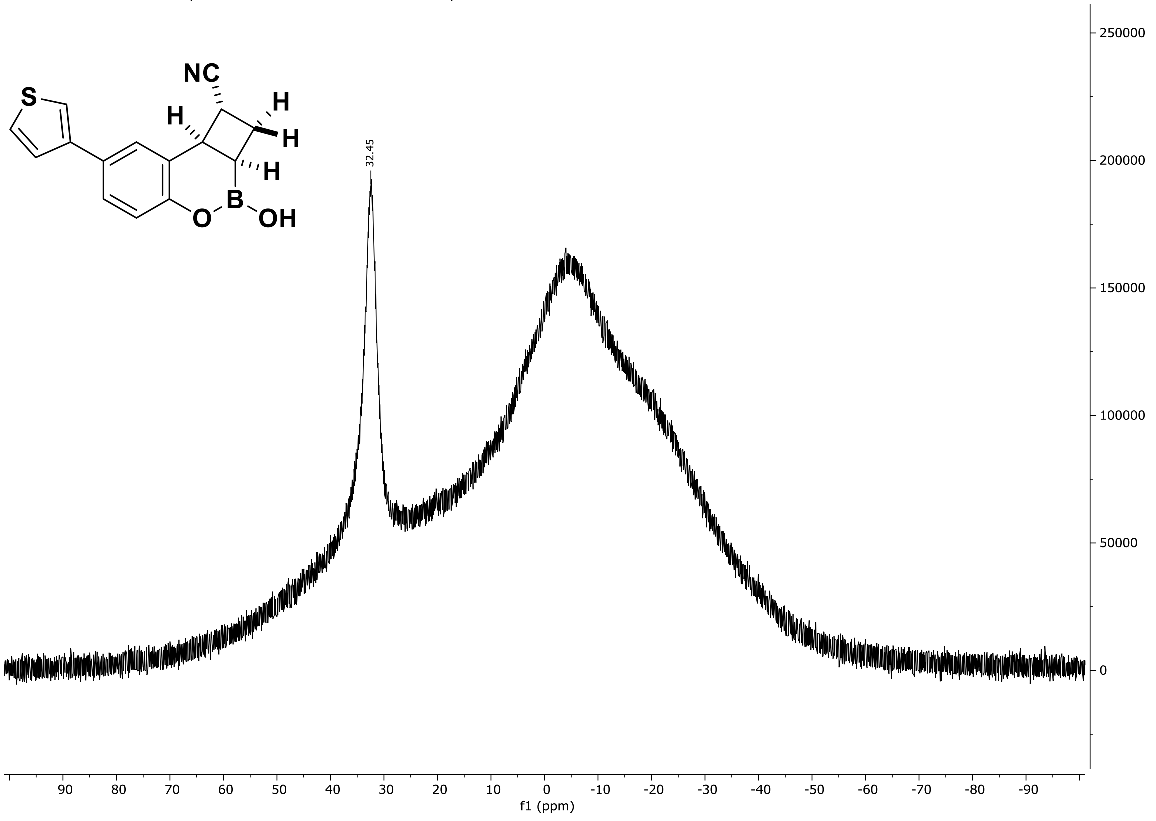

^{1}**H-NMR of 9** (600 MHz, $CDCl_3$)

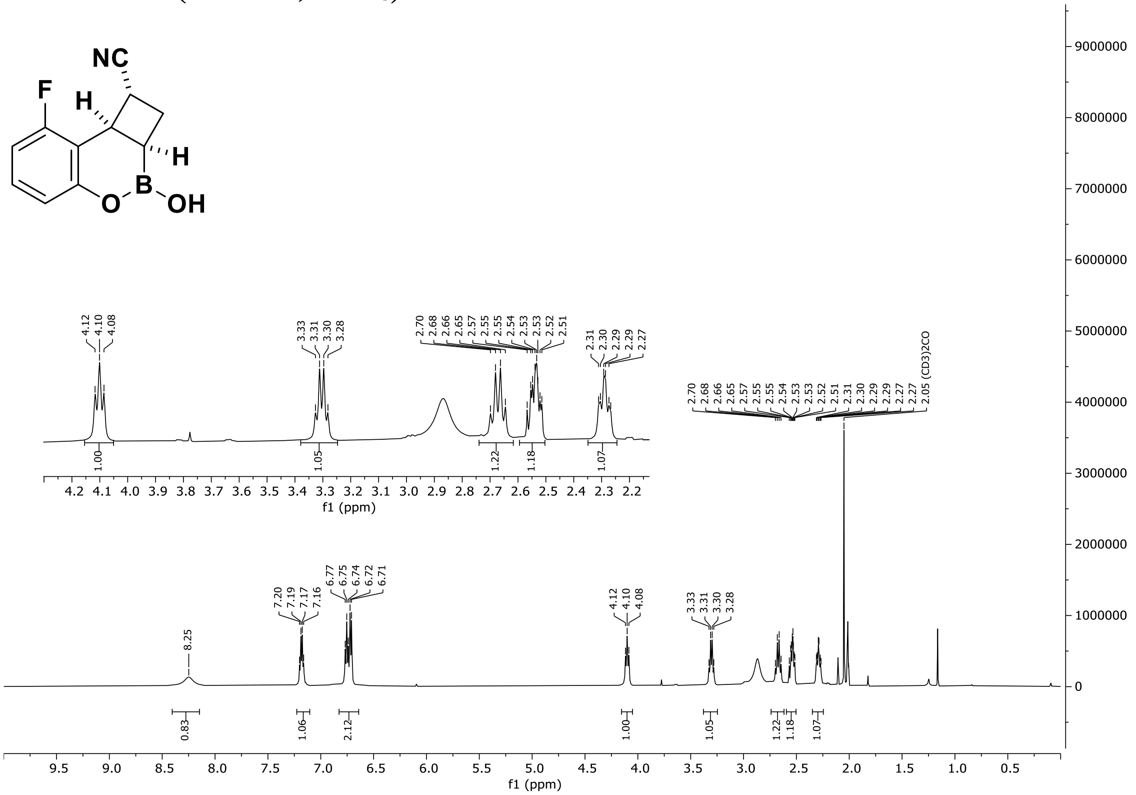

^{13}**C-NMR of 9** (100 MHz, $CDCl_3$)

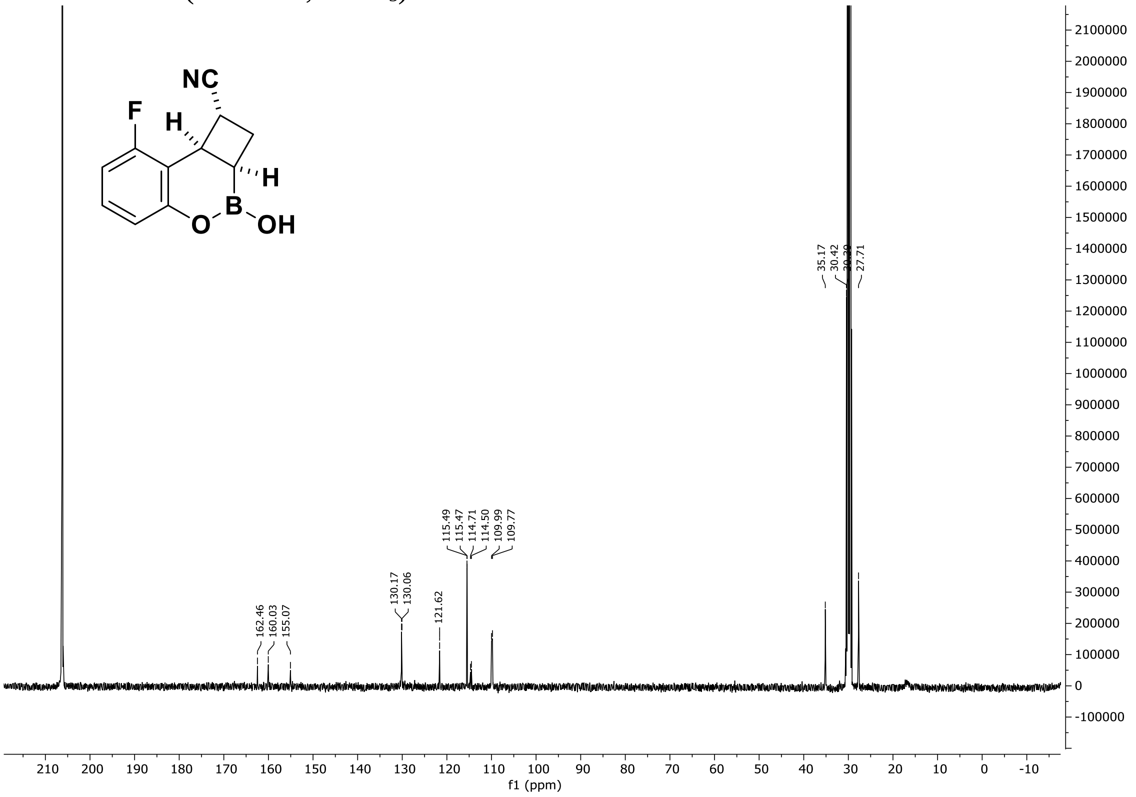

^{11}B-NMR of 9 (192 MHz, $CDCl_3$)

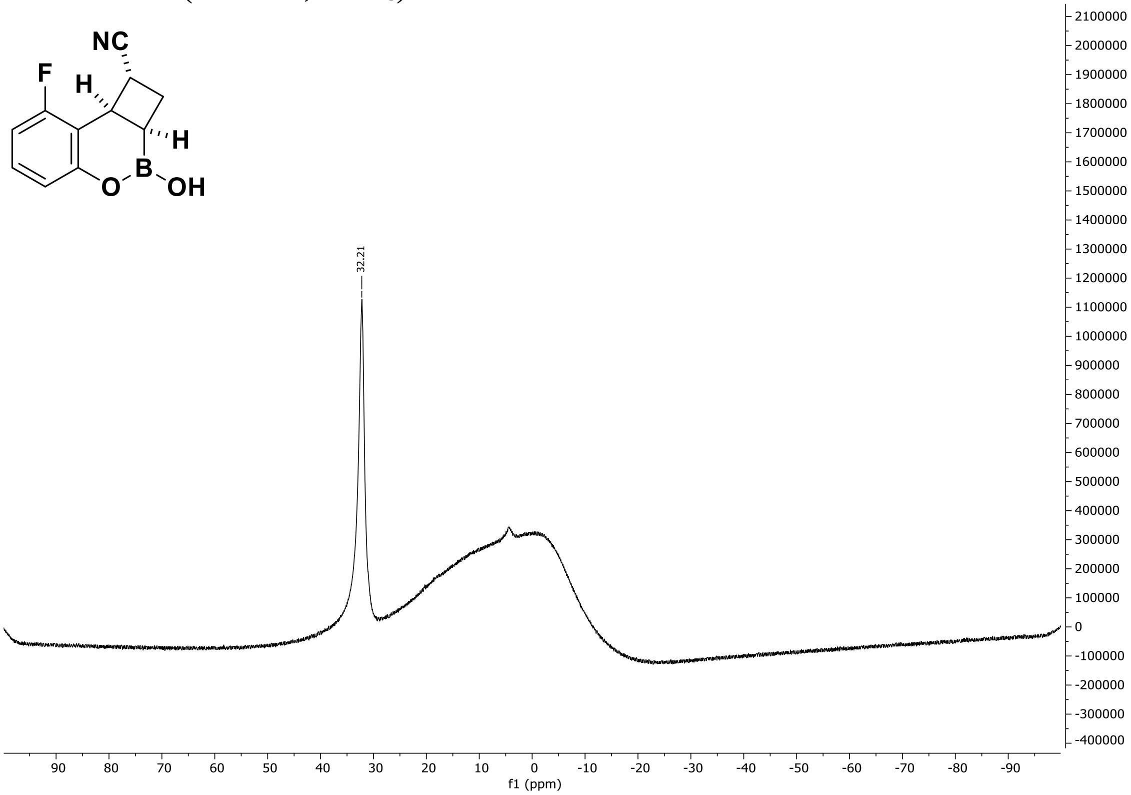

^{19}F NMR of 9 (564 MHz, Acetone-d_6)

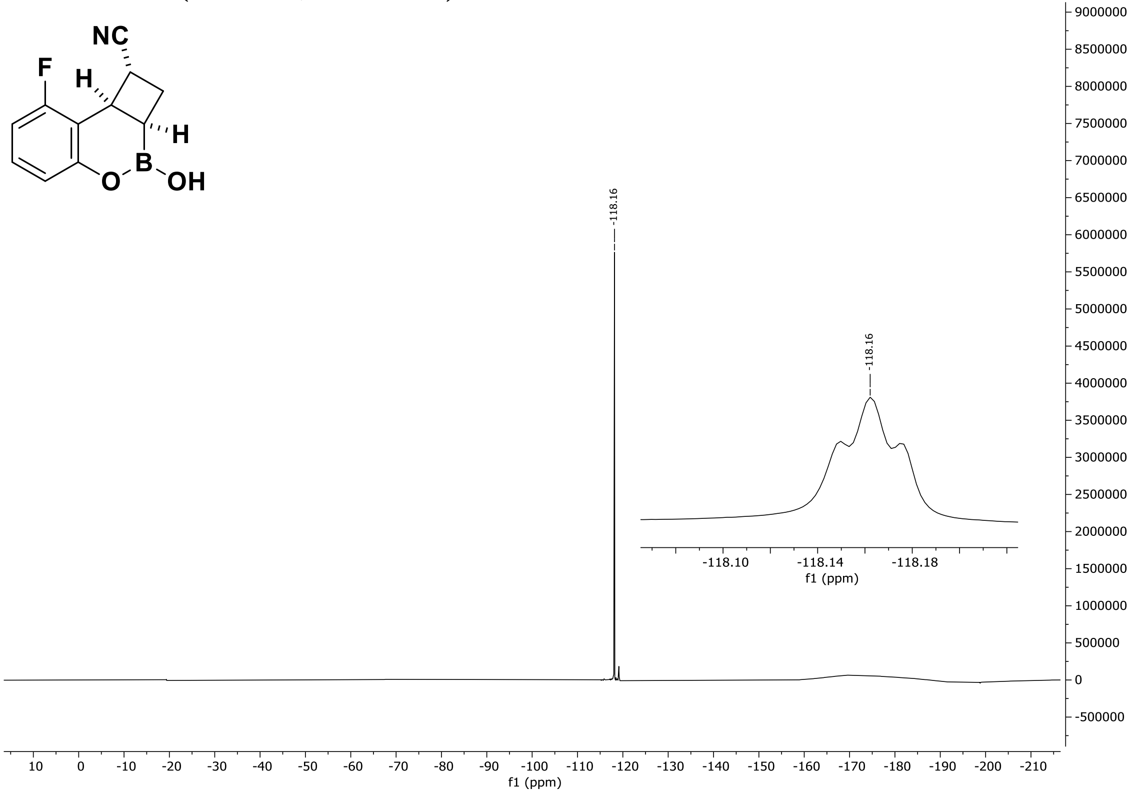

^{1}H-NMR of 9-*syn* (600 MHz, Acetone-d_6)

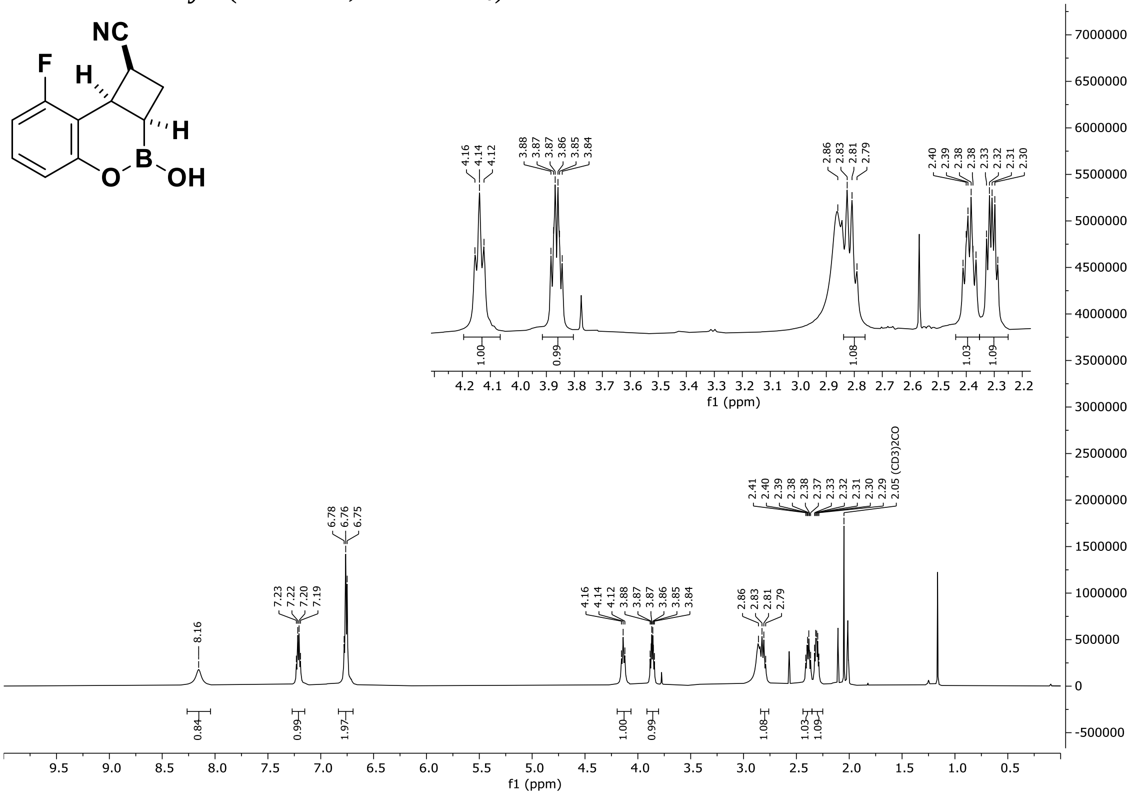

^{13}C-NMR of 9-*syn* (151 MHz, Acetone-d_6)

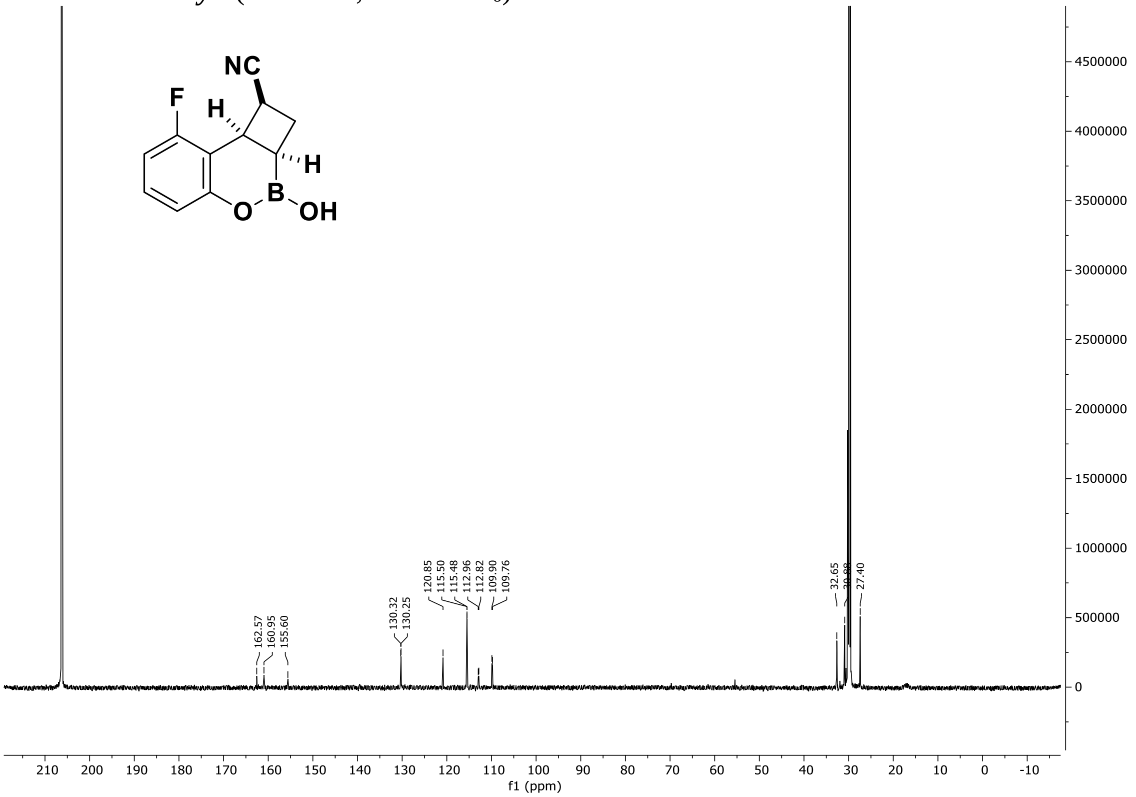

^{11}B-NMR of 9-*syn* (192 MHz, Acetone-d_6)

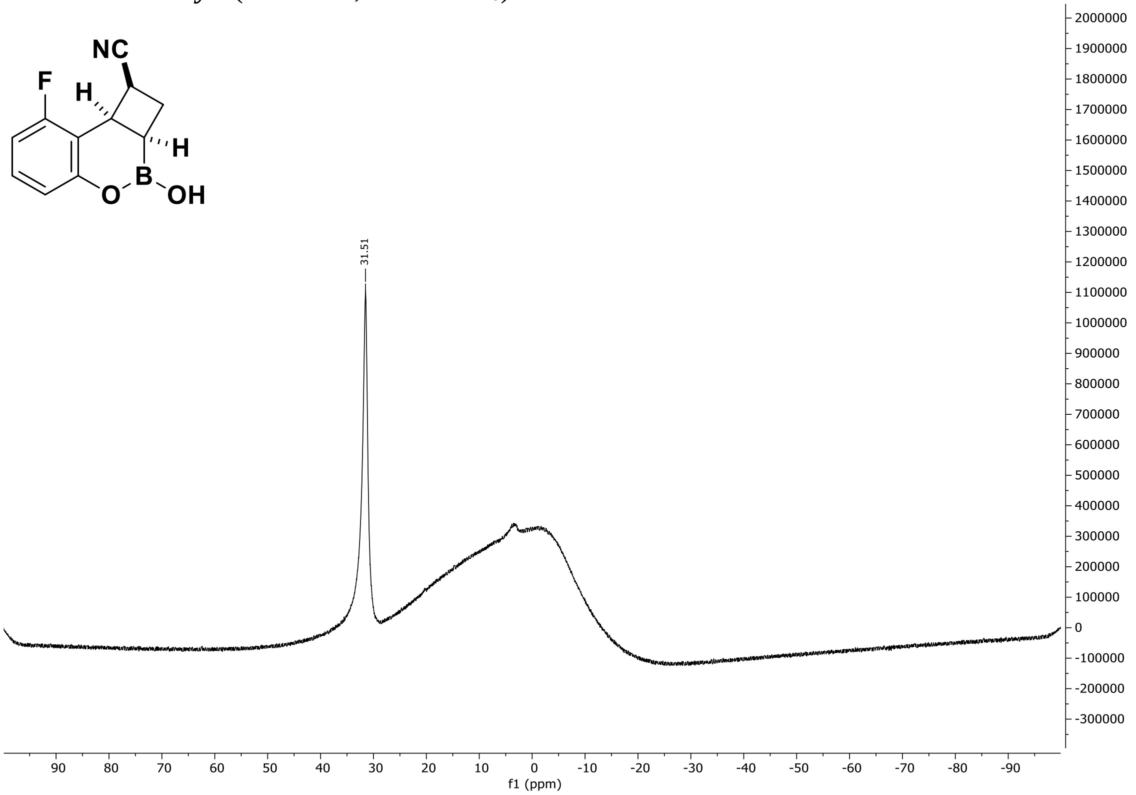

^{19}F NMR of 9-*syn* (564 MHz, Acetone-d_6)

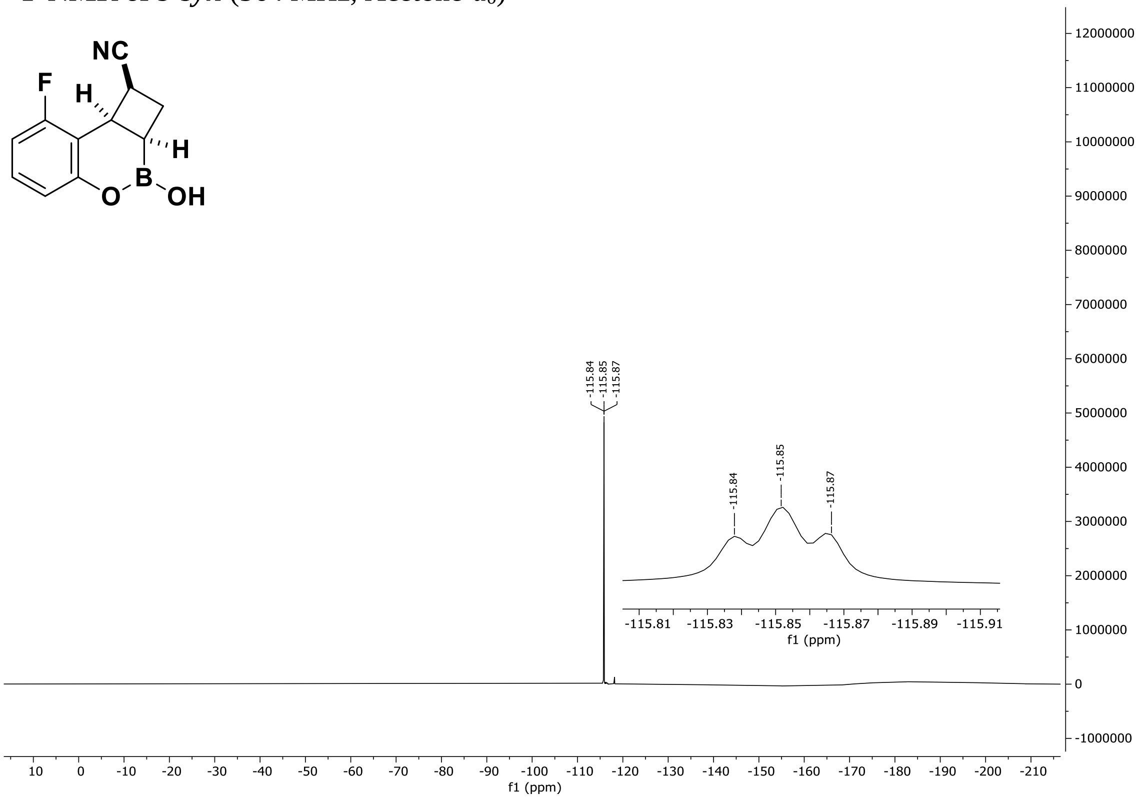

^{1}H-NMR of 10 (400 MHz, Acetone-d_6)

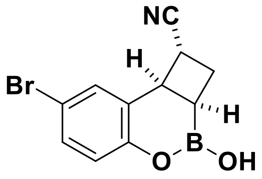

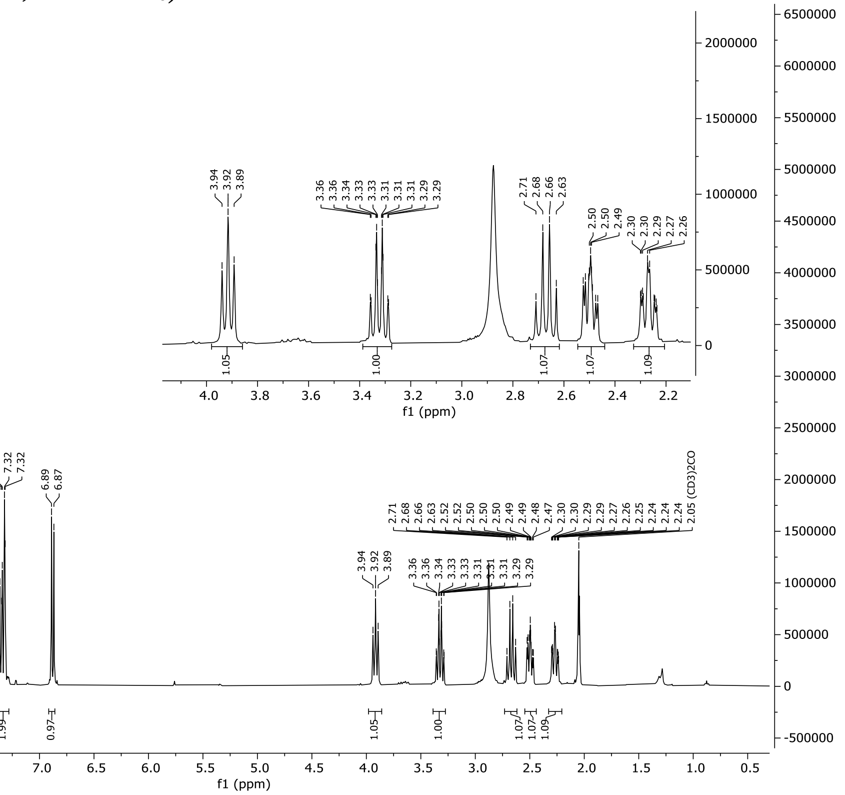

NOESY of 10 (400 MHz, Acetone-d_6)

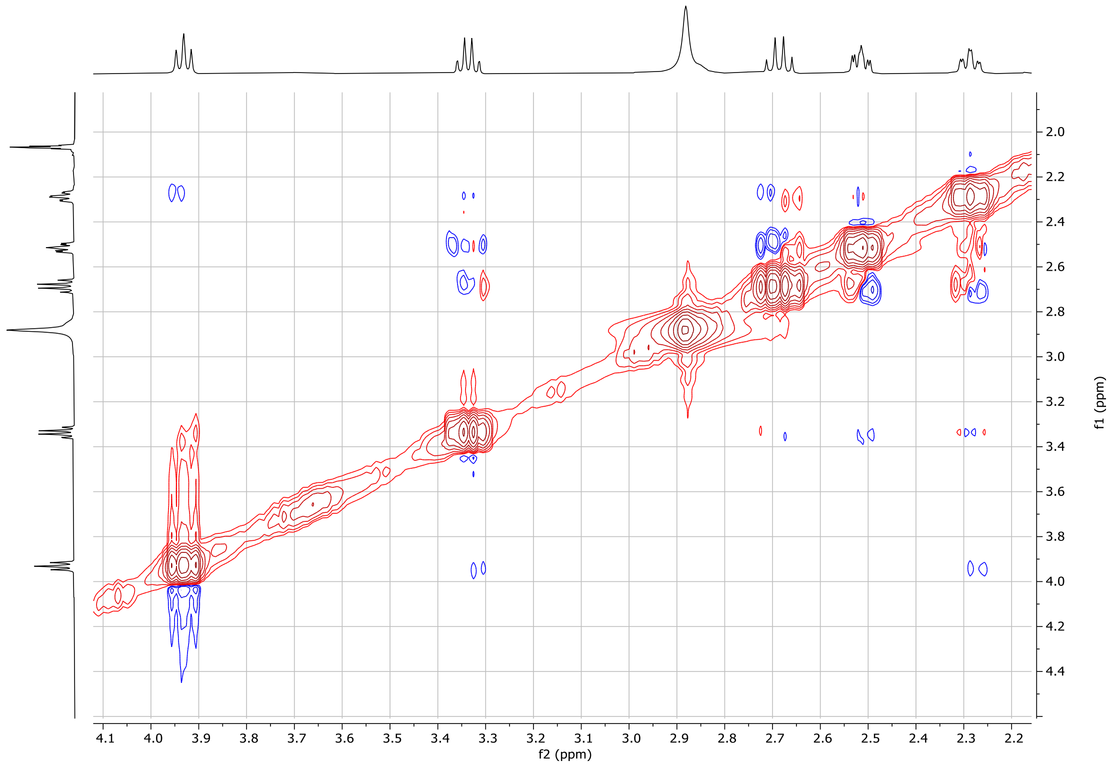

^{13}C-NMR of 10 (100 MHz, Acetone-d_6)

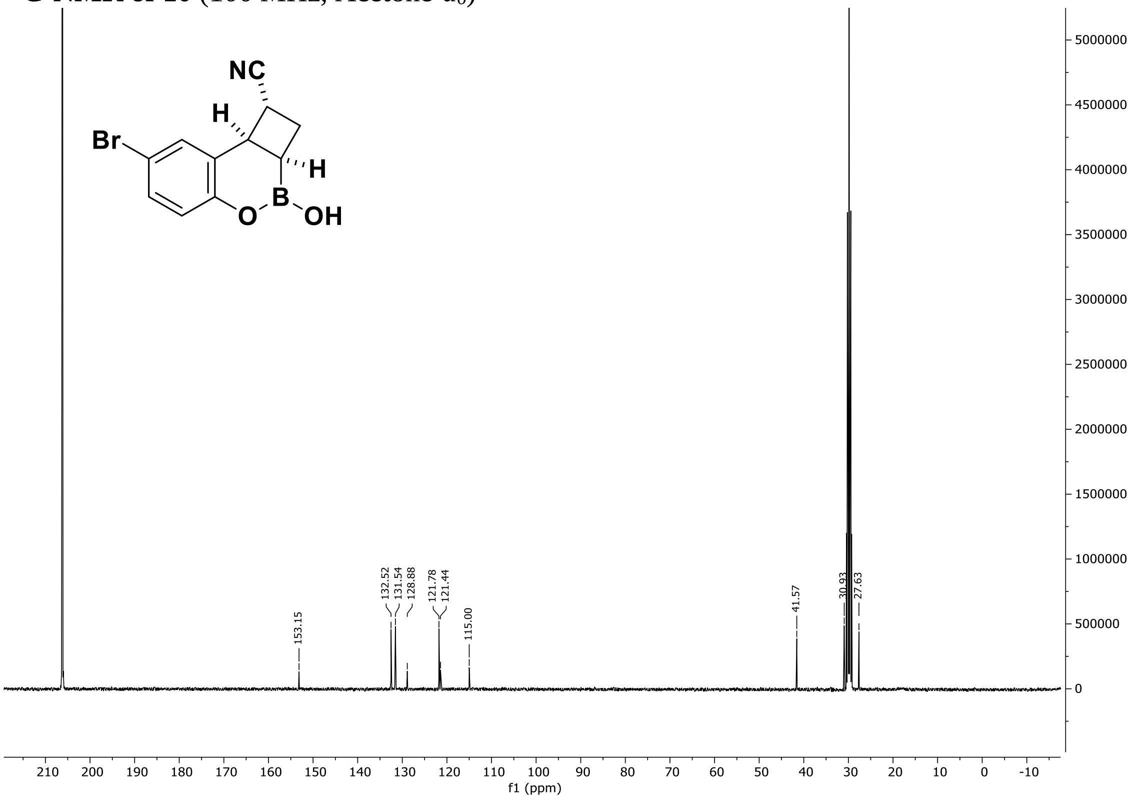

^{11}B-NMR of 10 (128 MHz, Acetone-d_6)

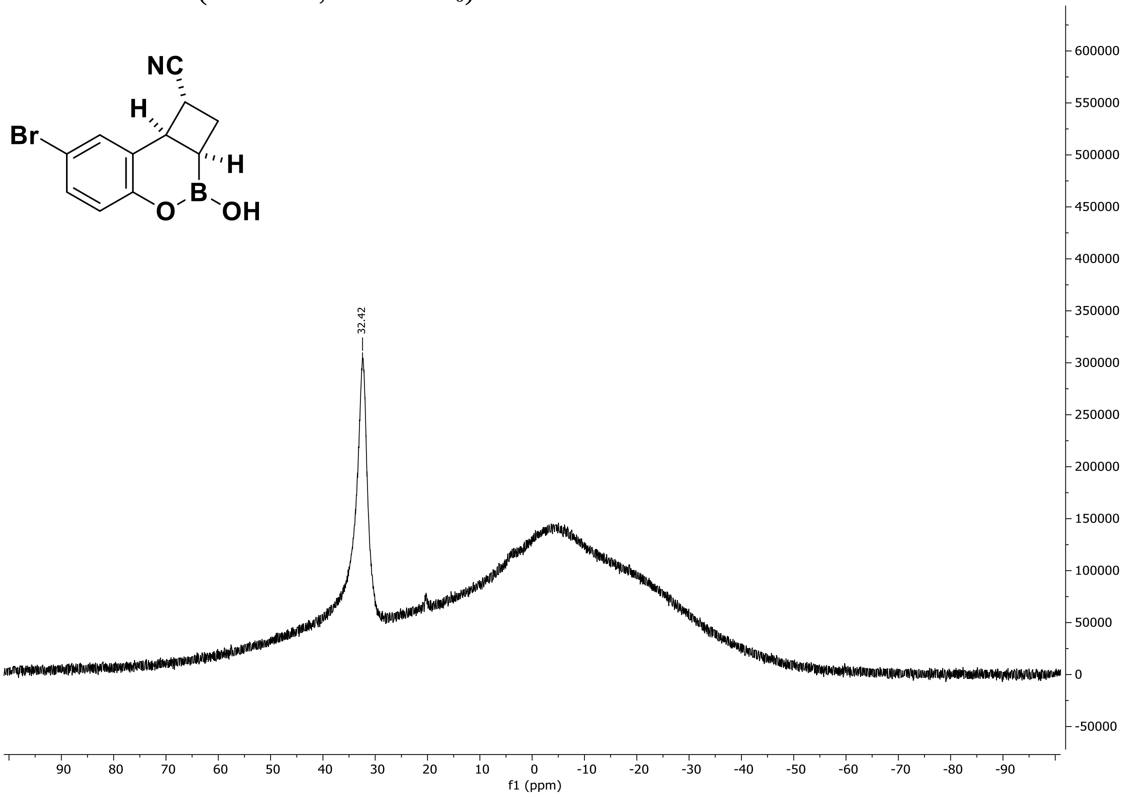

^{1}H-NMR of 10-*syn* (400 MHz, Acetone-d_6)

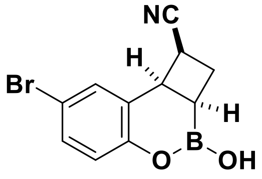

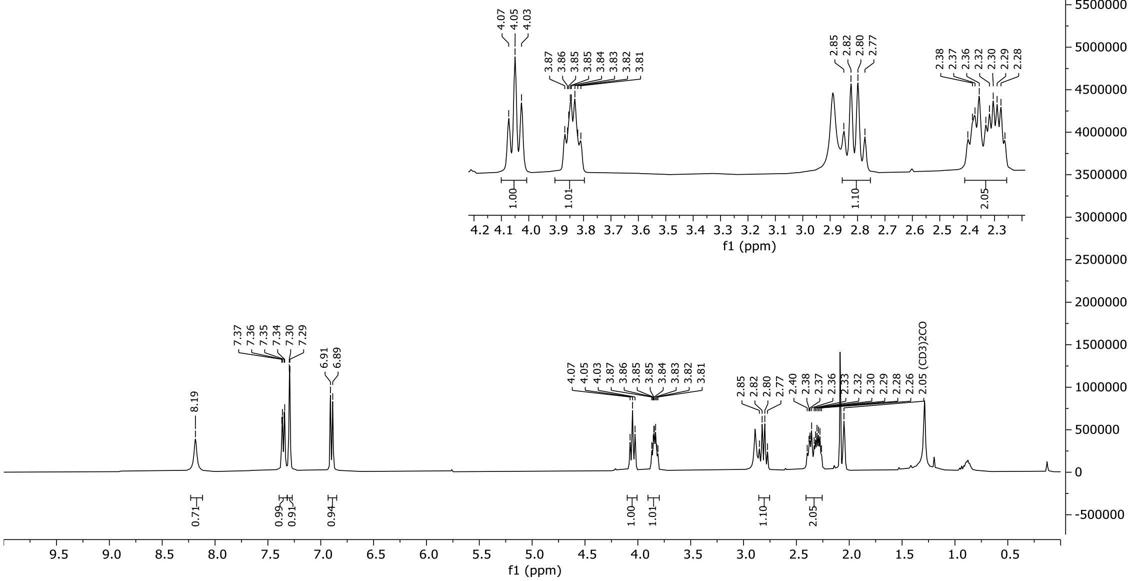

NOESY of 10-*syn* (400 MHz, Acetone-d_6)

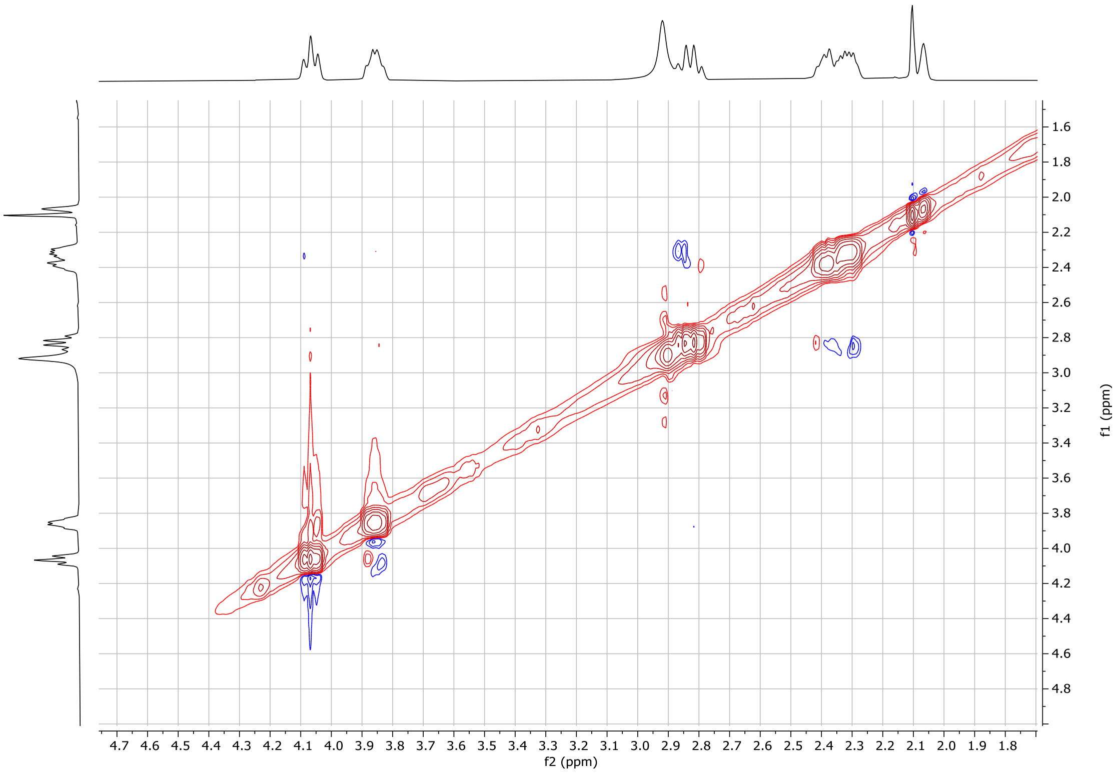

^{13}C-NMR of 10-*syn* (100 MHz, Acetone-d_6)

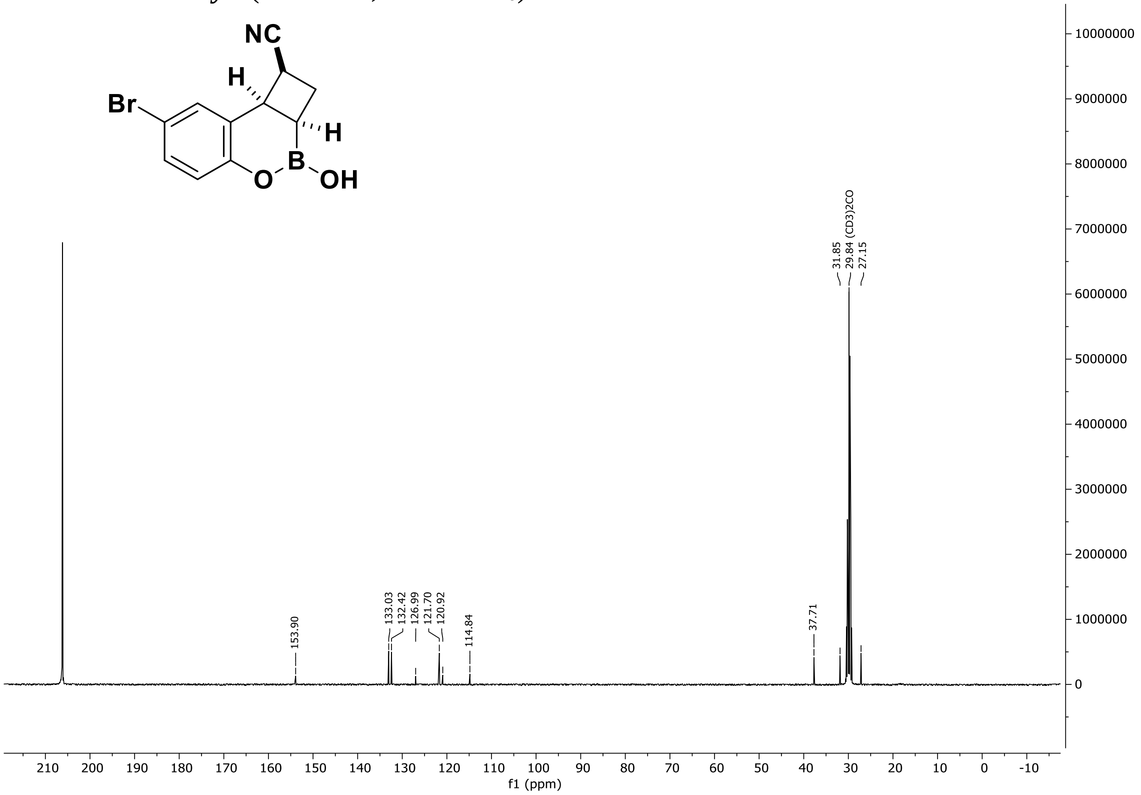

^{11}B-NMR of 10-*syn* (128 MHz, Acetone-d_6)

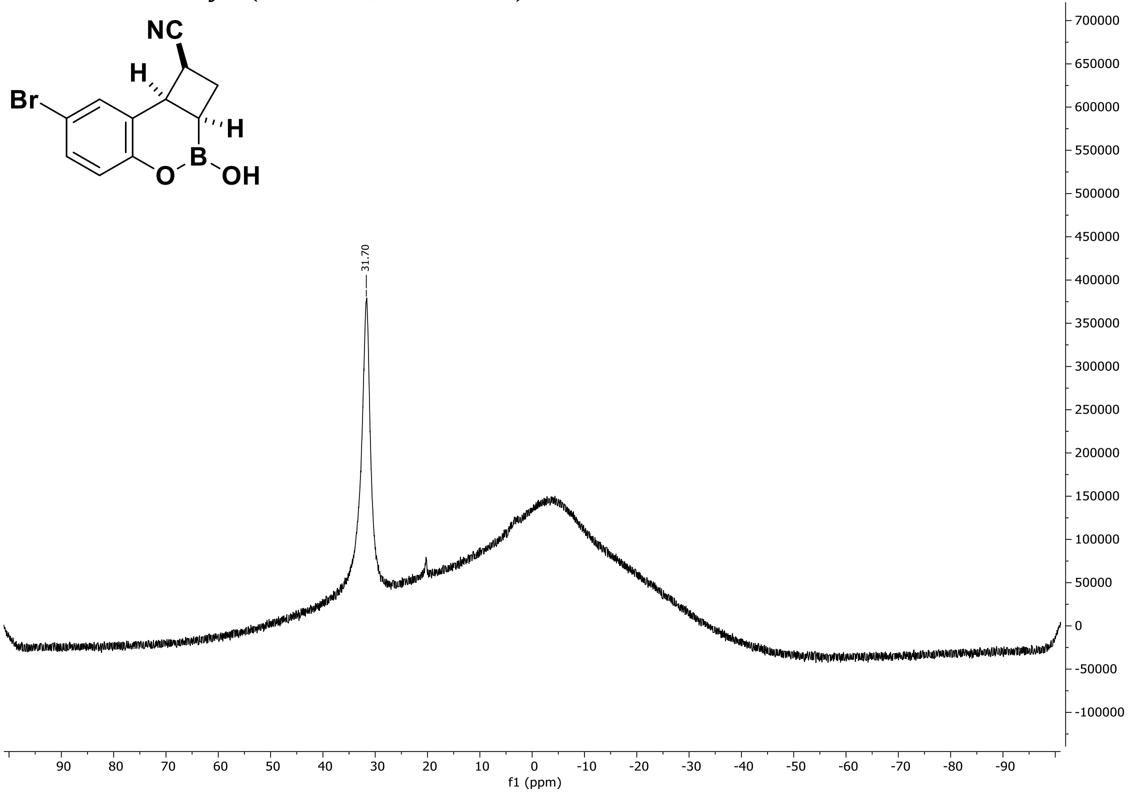

^{1}H-NMR of 11 (400 MHz, Acetone-d_6)

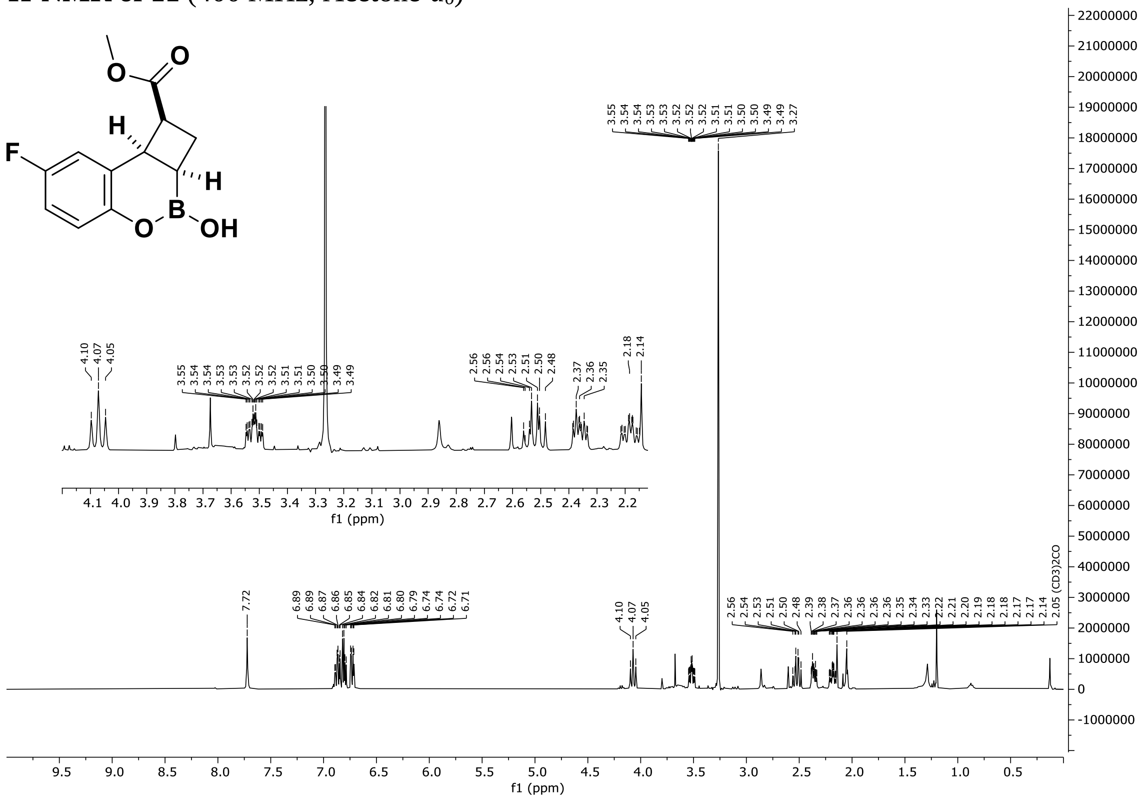

^{13}C-NMR of 11 (101 MHz, Acetone-d_6)

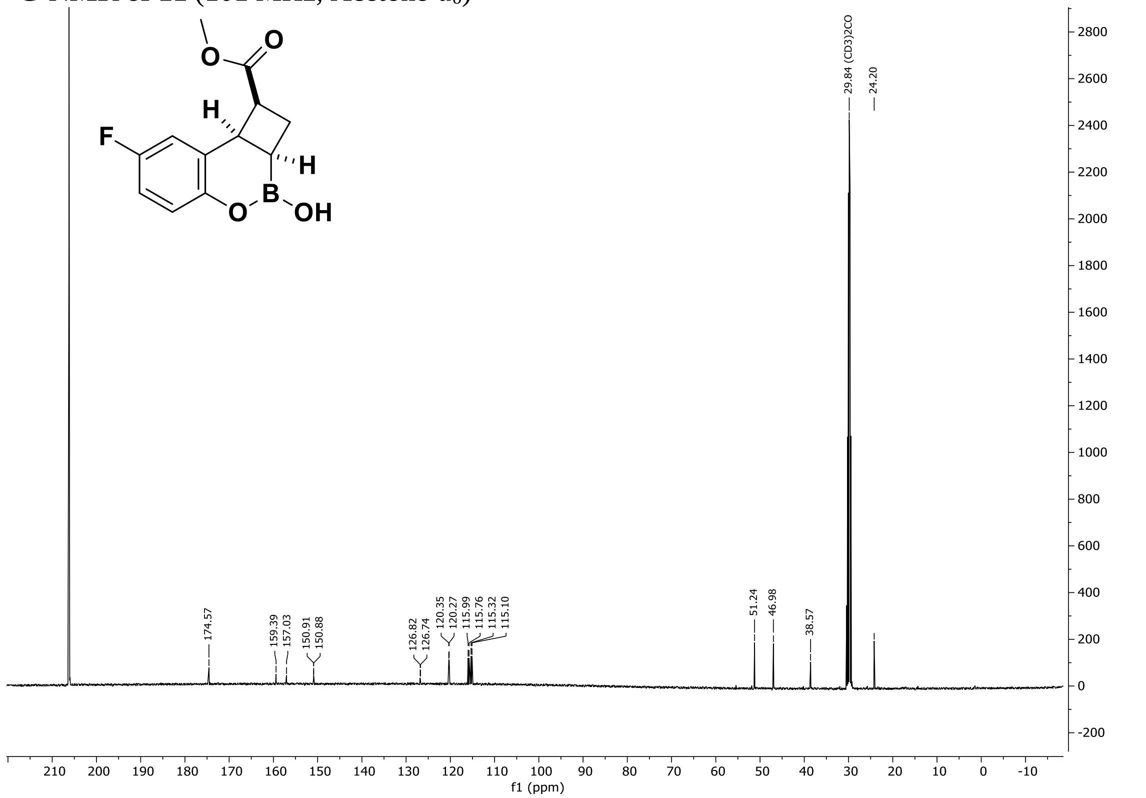

^{11}B-NMR of 11 (128 MHz, Acetone-d_6)

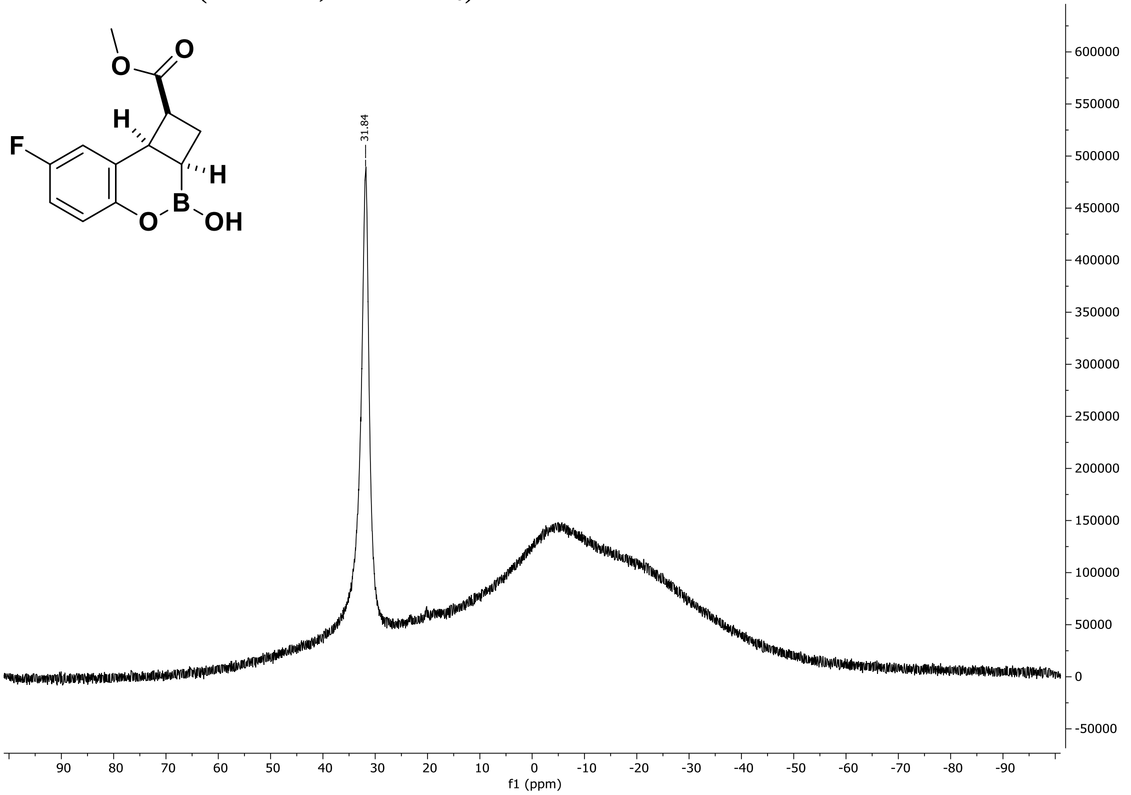

^{19}F NMR of 11 (376 MHz, Acetone-d_6)

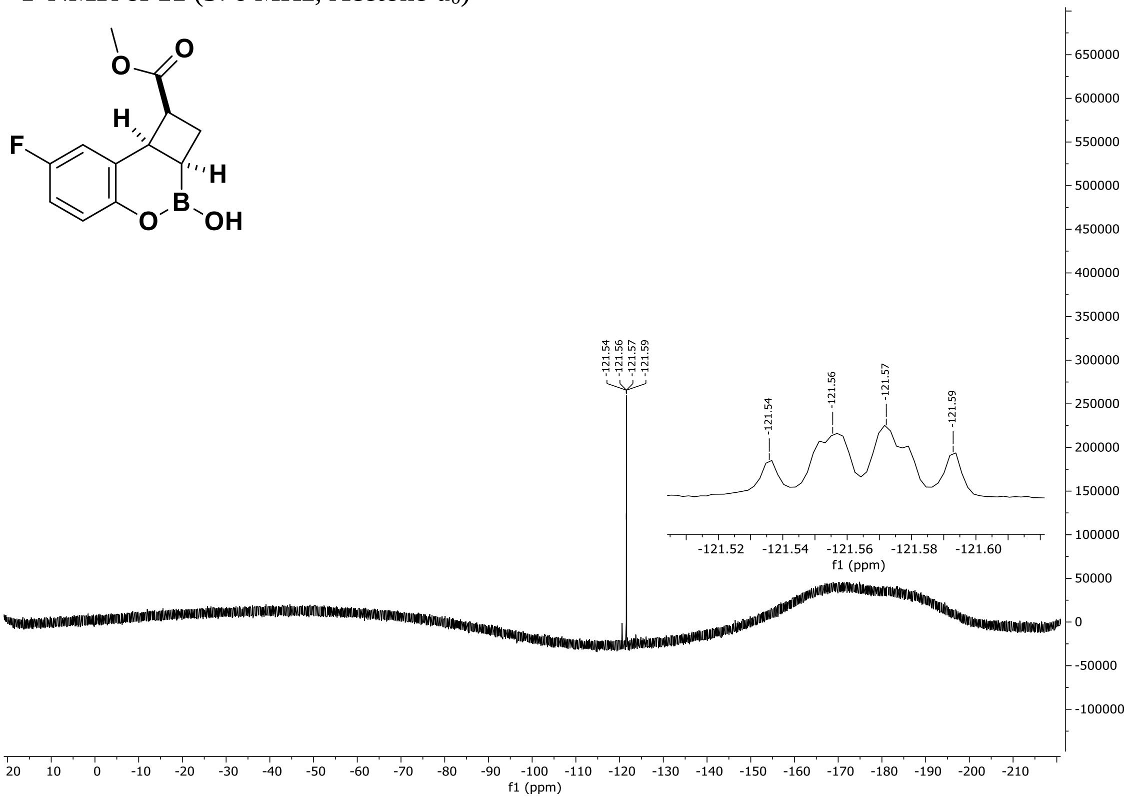

NOESY of 11

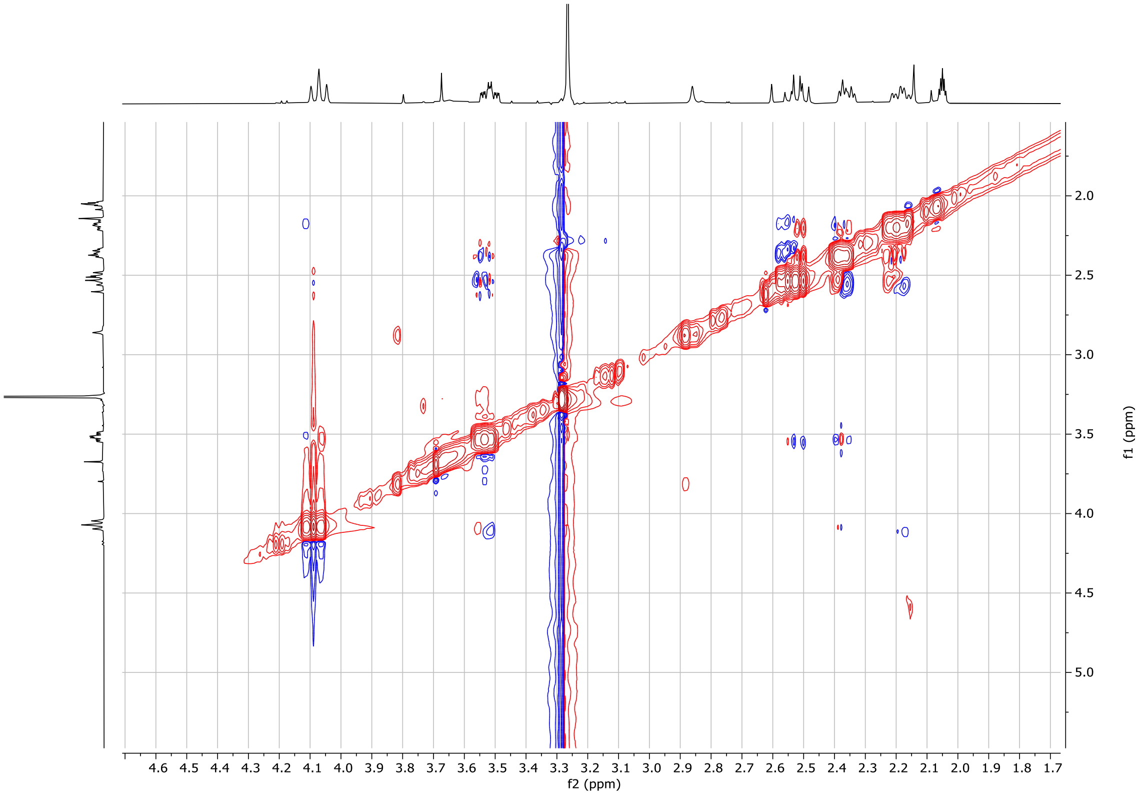

^{1}H-NMR of 12 (400 MHz, Acetone-d_6)

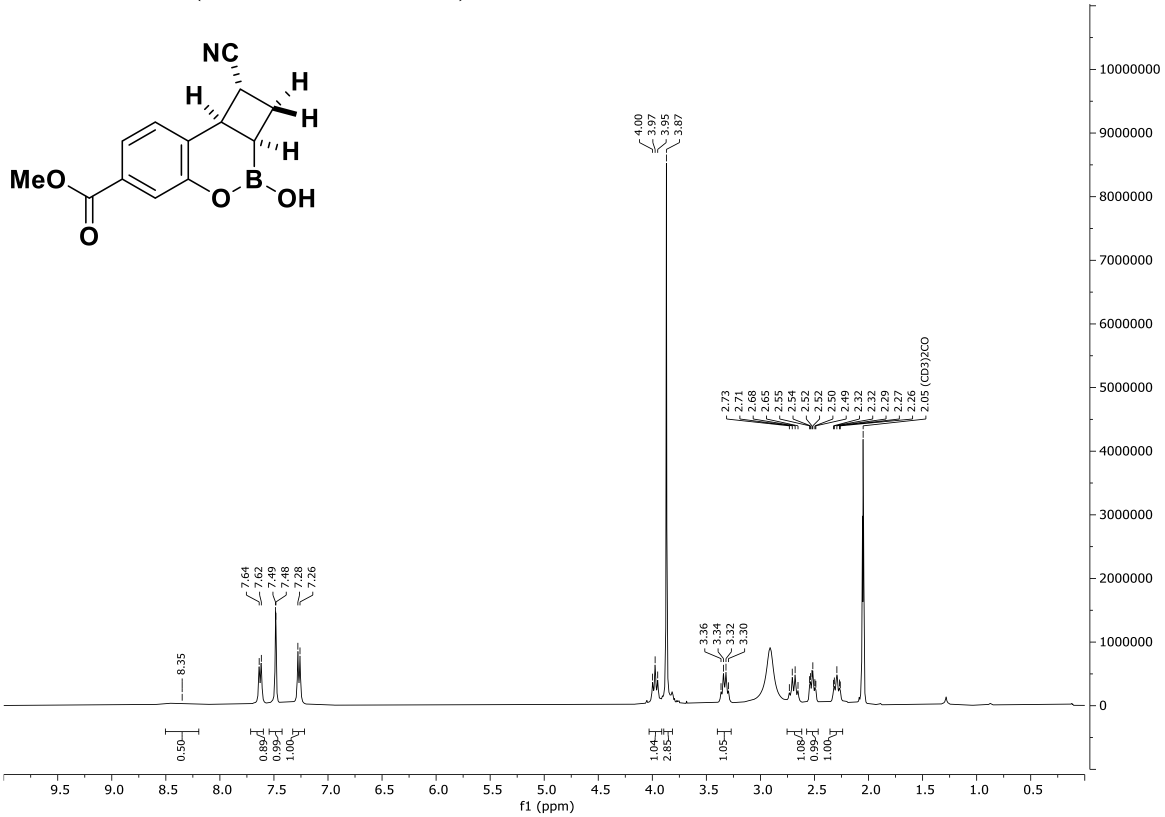

^{13}C-NMR of 12 (100 MHz, Acetone-d_6)

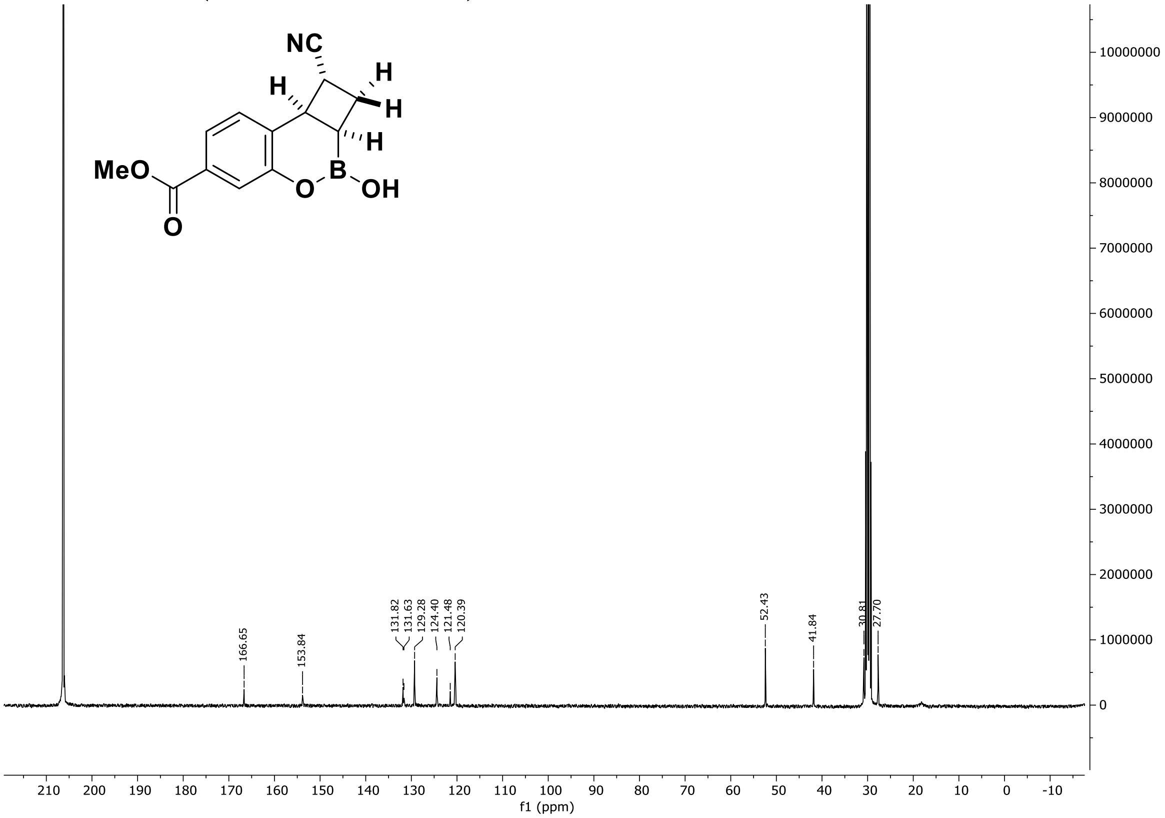

^{11}B-NMR of 12 (128 MHz, Acetone-d_6)

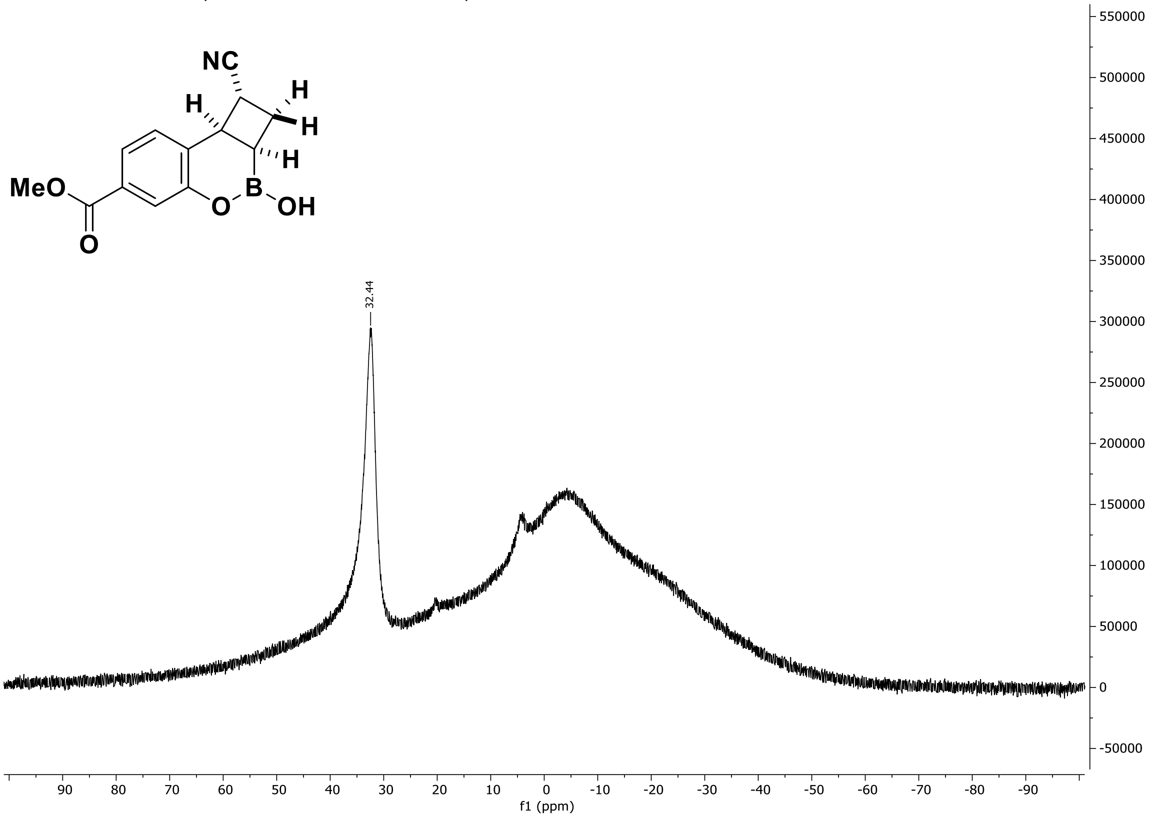

^{1}H-NMR of 13 (400 MHz, $CDCl_3$)

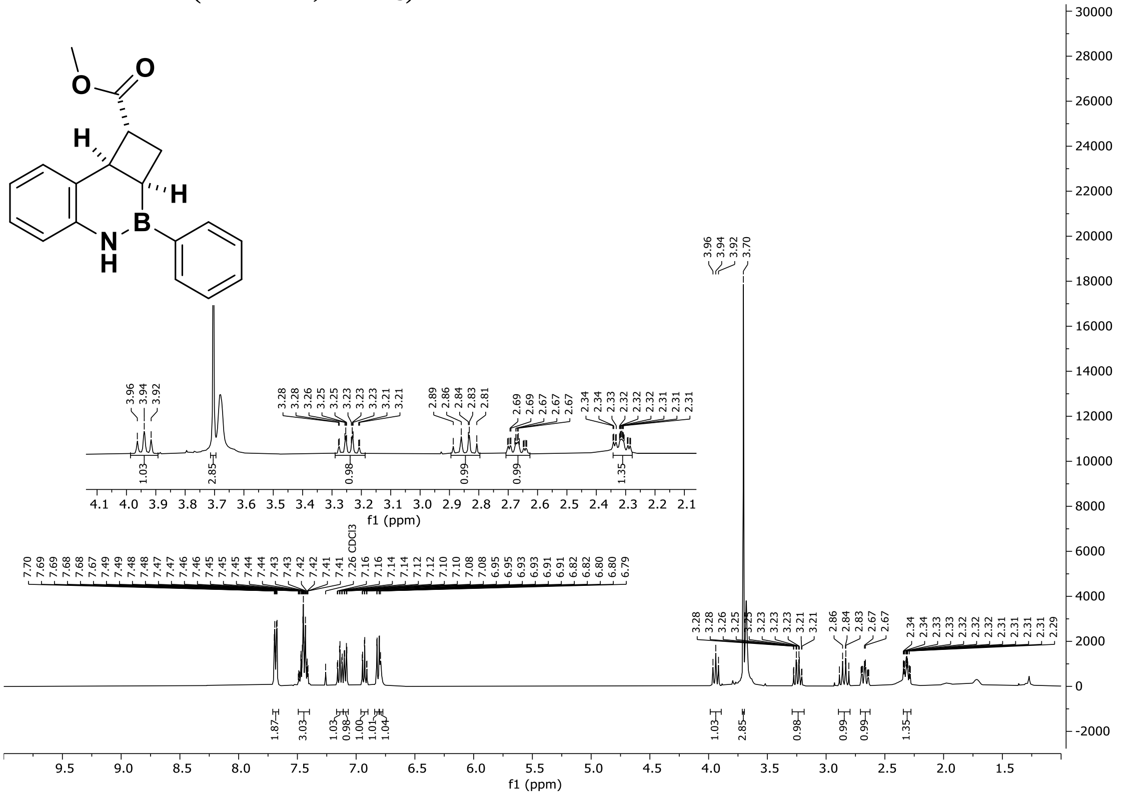

^{13}C-NMR of 13 (101 MHz, $CDCl_3$)

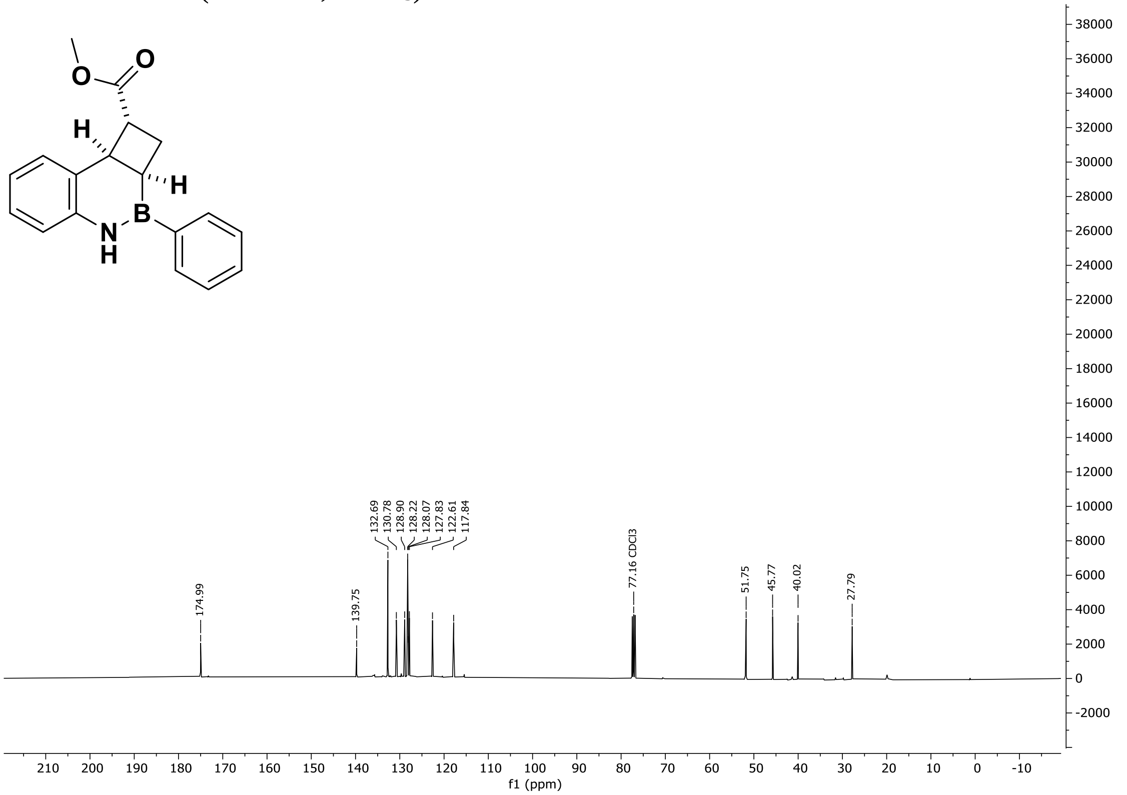

^{11}**B-NMR of 13** (128 MHz, $CDCl_3$)

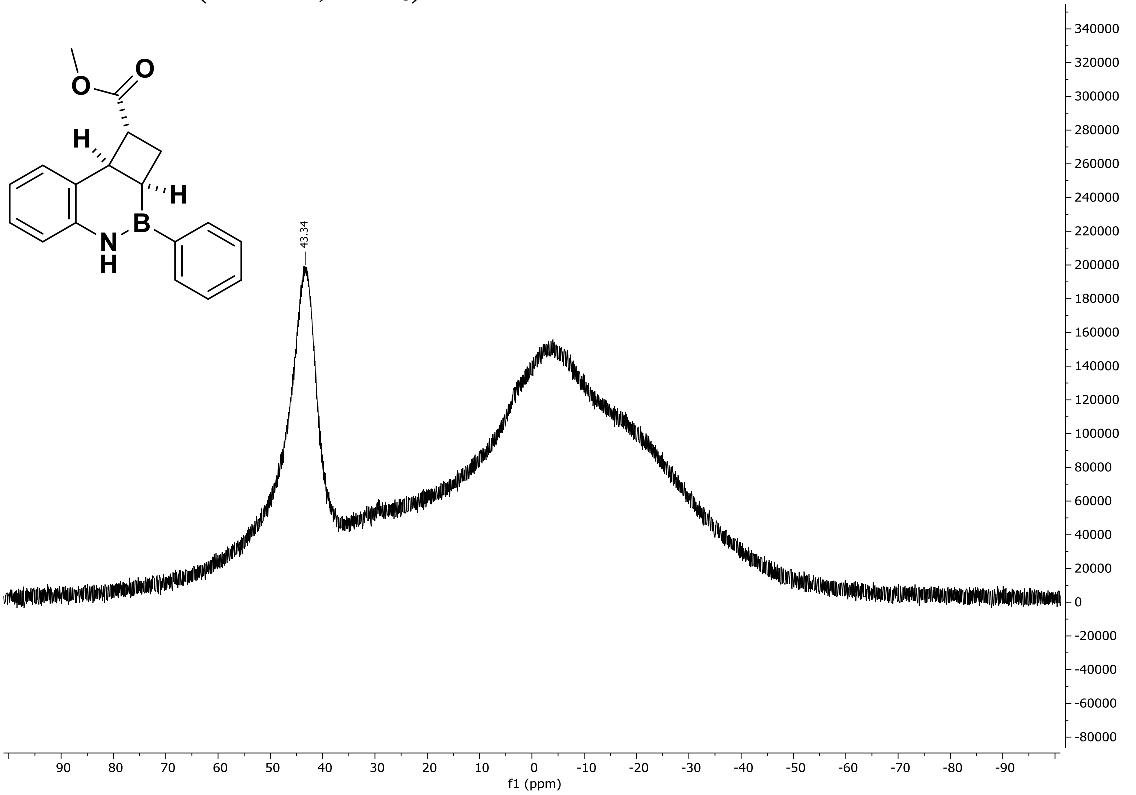

^{1}**H-NMR of 14** (600 MHz, $CDCl_3$)

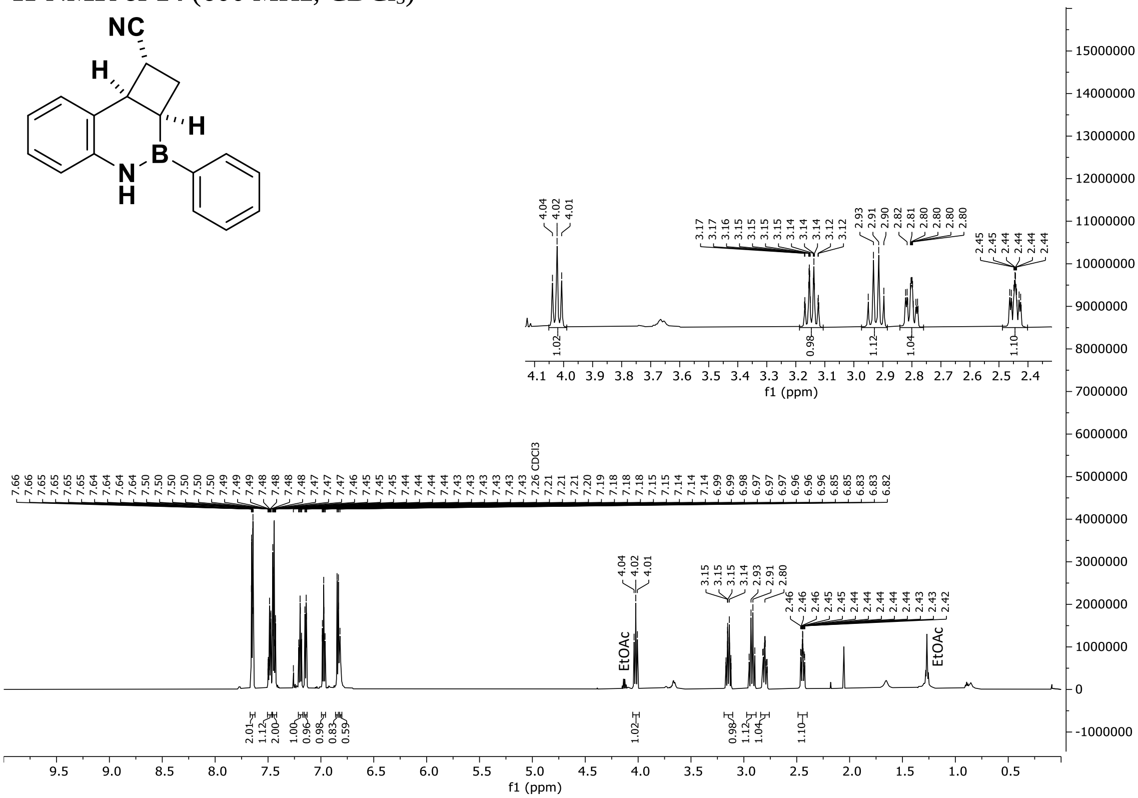

^{13}C-NMR of 14 (151 MHz, $CDCl_3$)

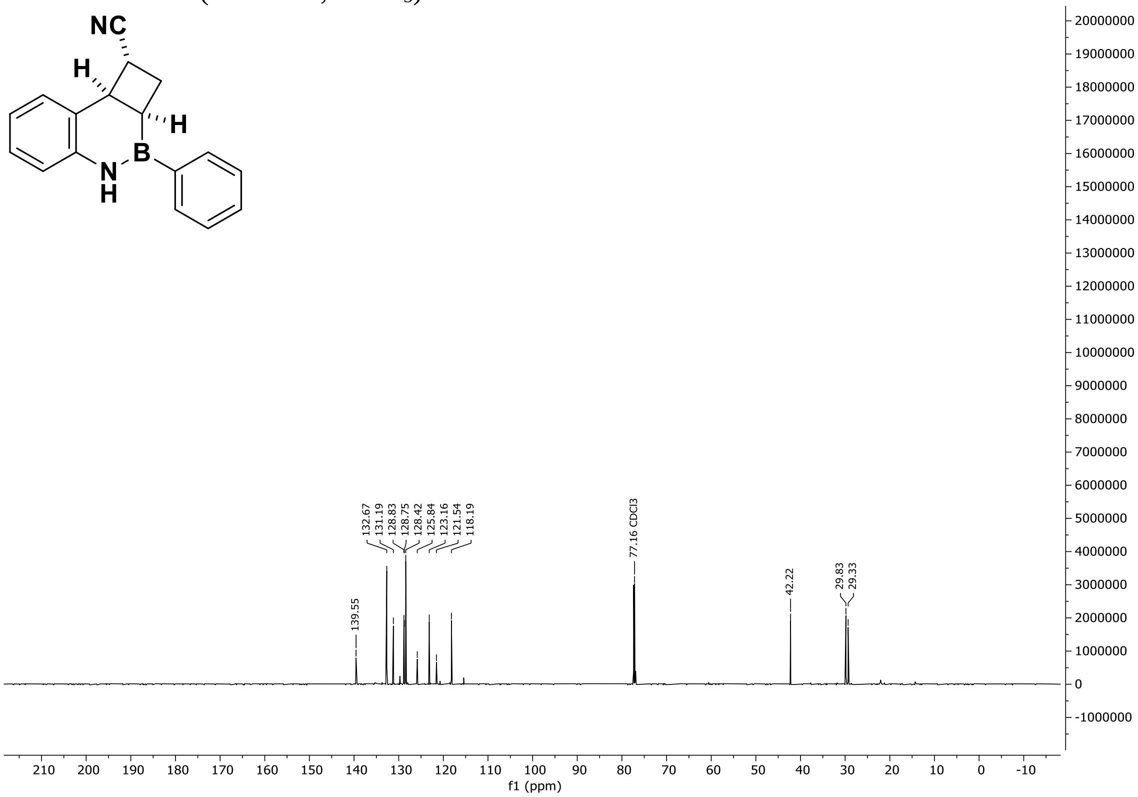

^{11}B-NMR of 14 (192 MHz, $CDCl_3$)

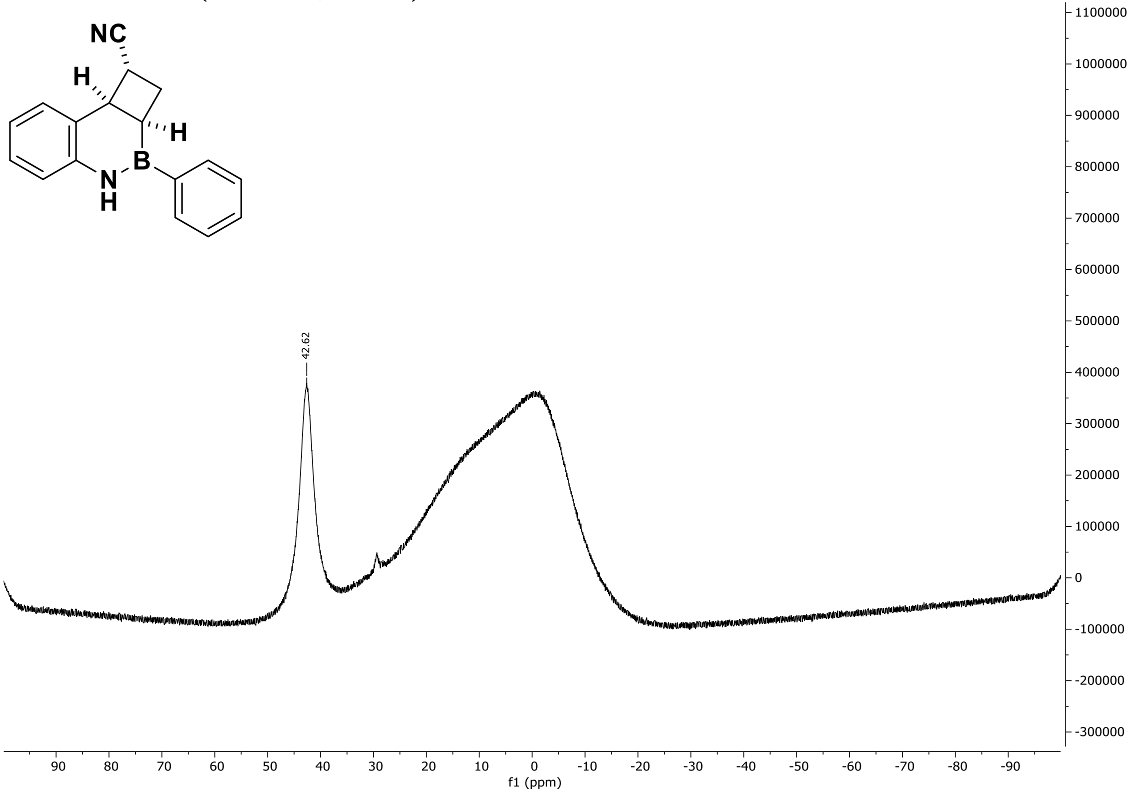

^{1}H-NMR of 15 (400 MHz, $CDCl_3$)

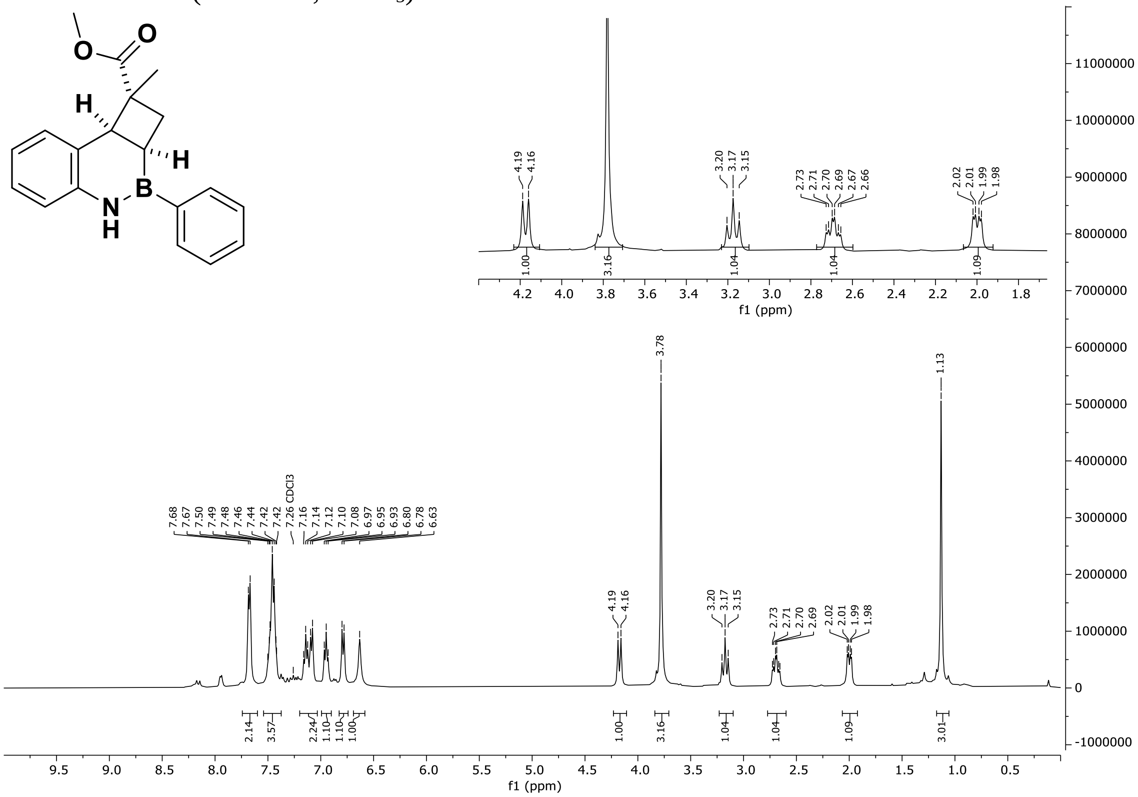

^{13}C-NMR of 15 (101 MHz, $CDCl_3$)

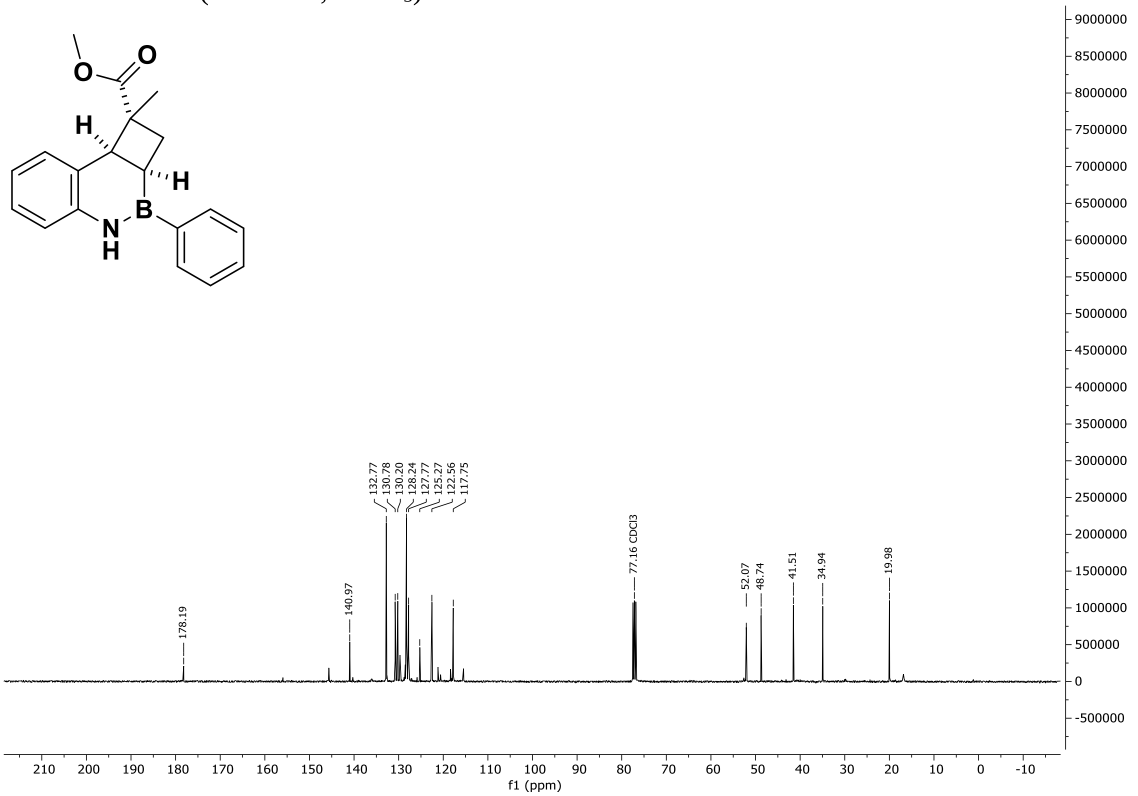

^{11}B-NMR of 15 (128 MHz, $CDCl_3$)

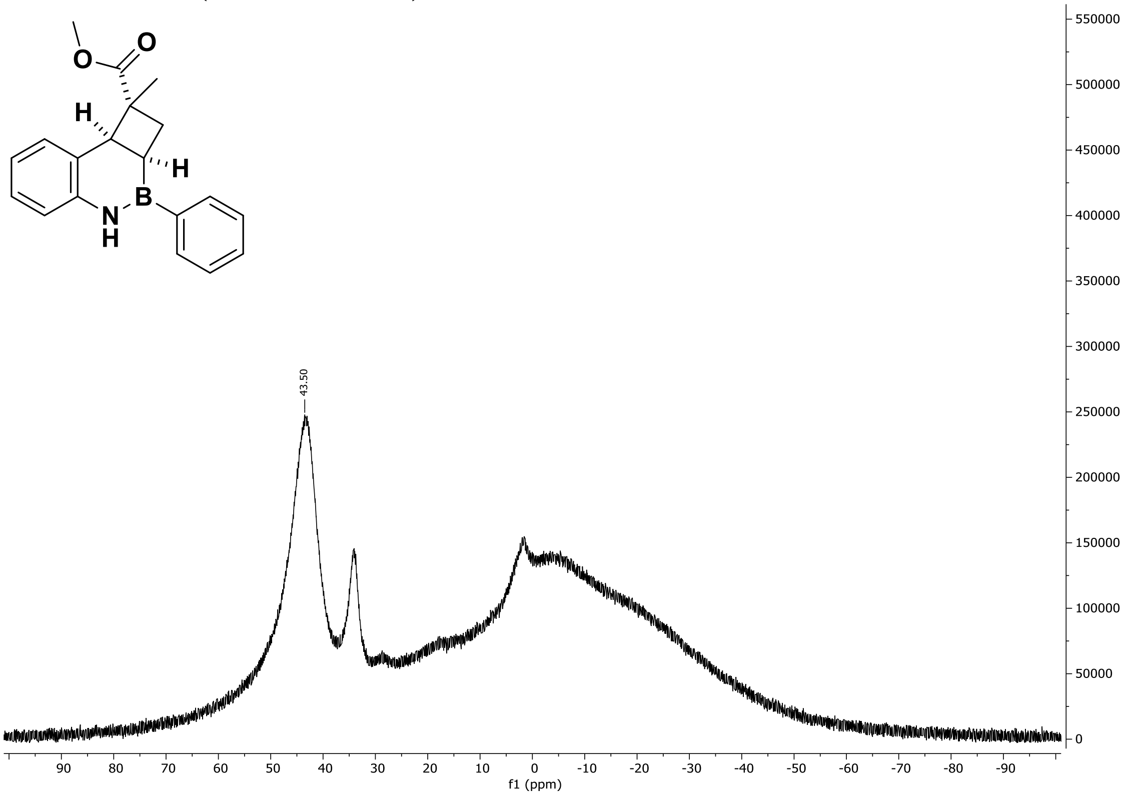

^{1}H-NMR of 16 (400 MHz, $CDCl_3$)

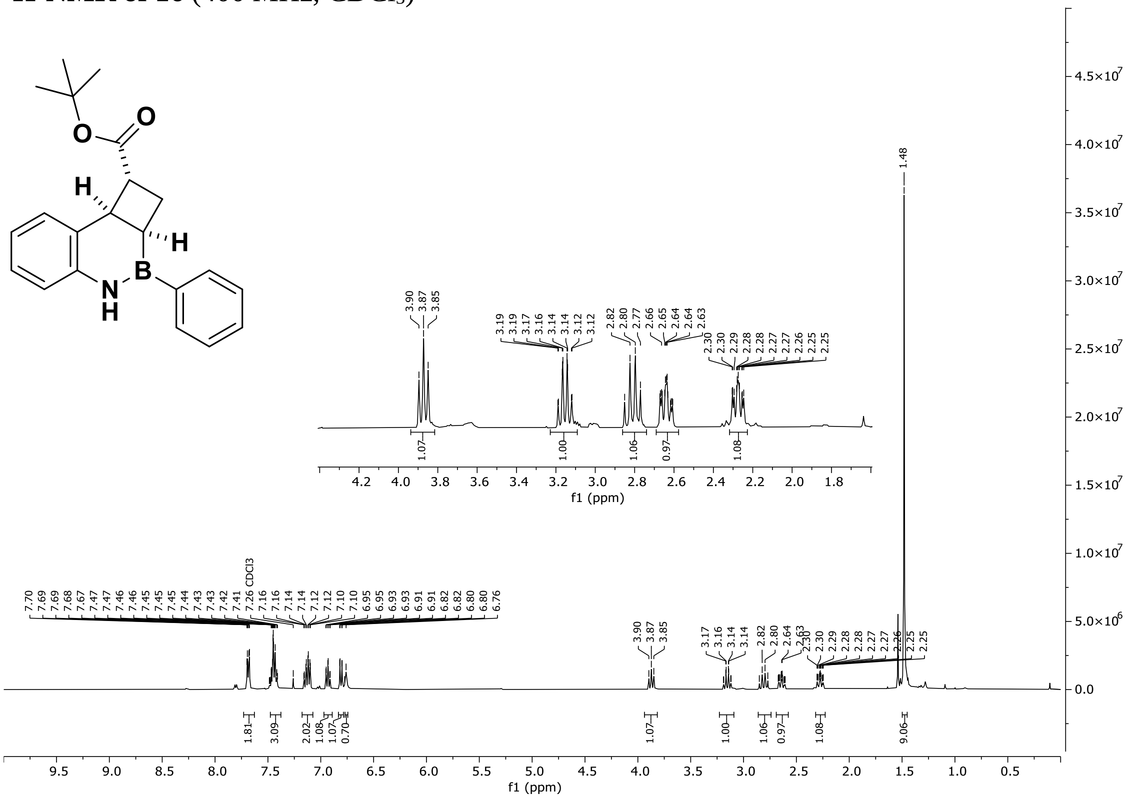

^{13}C-NMR of 16 (101 MHz, $CDCl_3$)

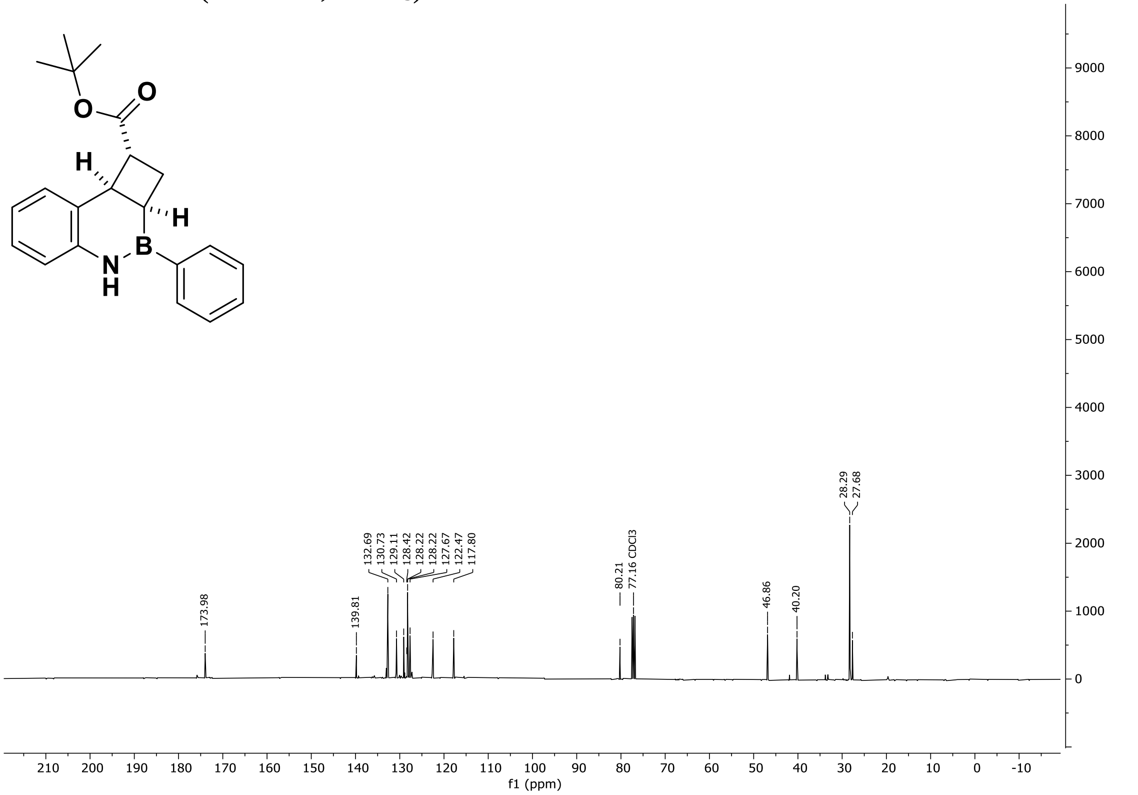

^{11}B-NMR of 16 (128 MHz, $CDCl_3$)

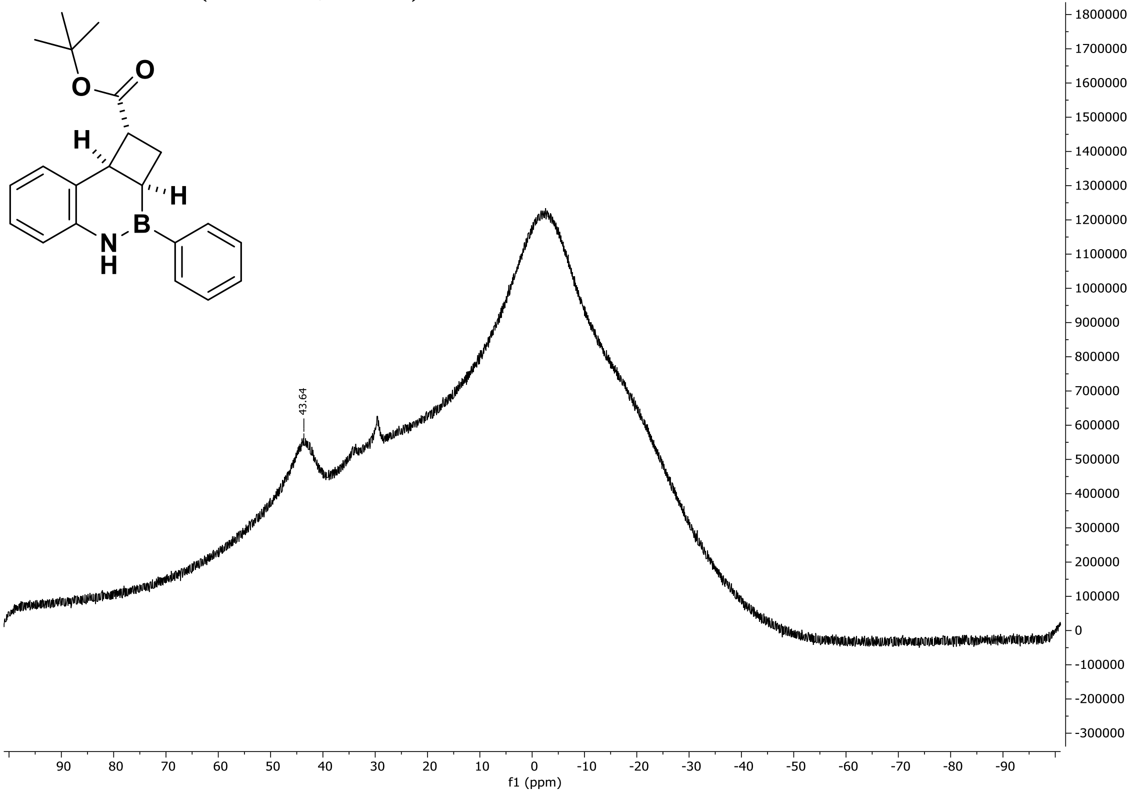

^{1}H-NMR of 17 (400 MHz, $CDCl_3$)

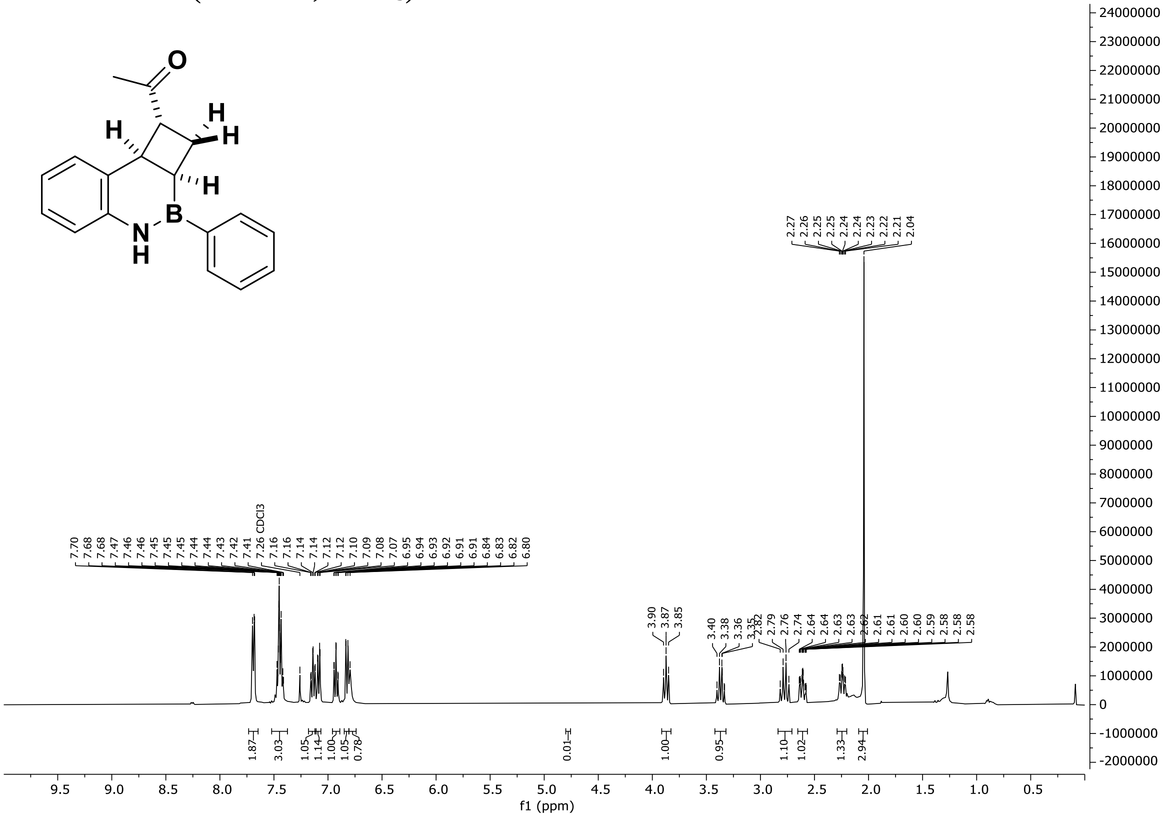

^{13}C-NMR of 17 (100 MHz, $CDCl_3$)

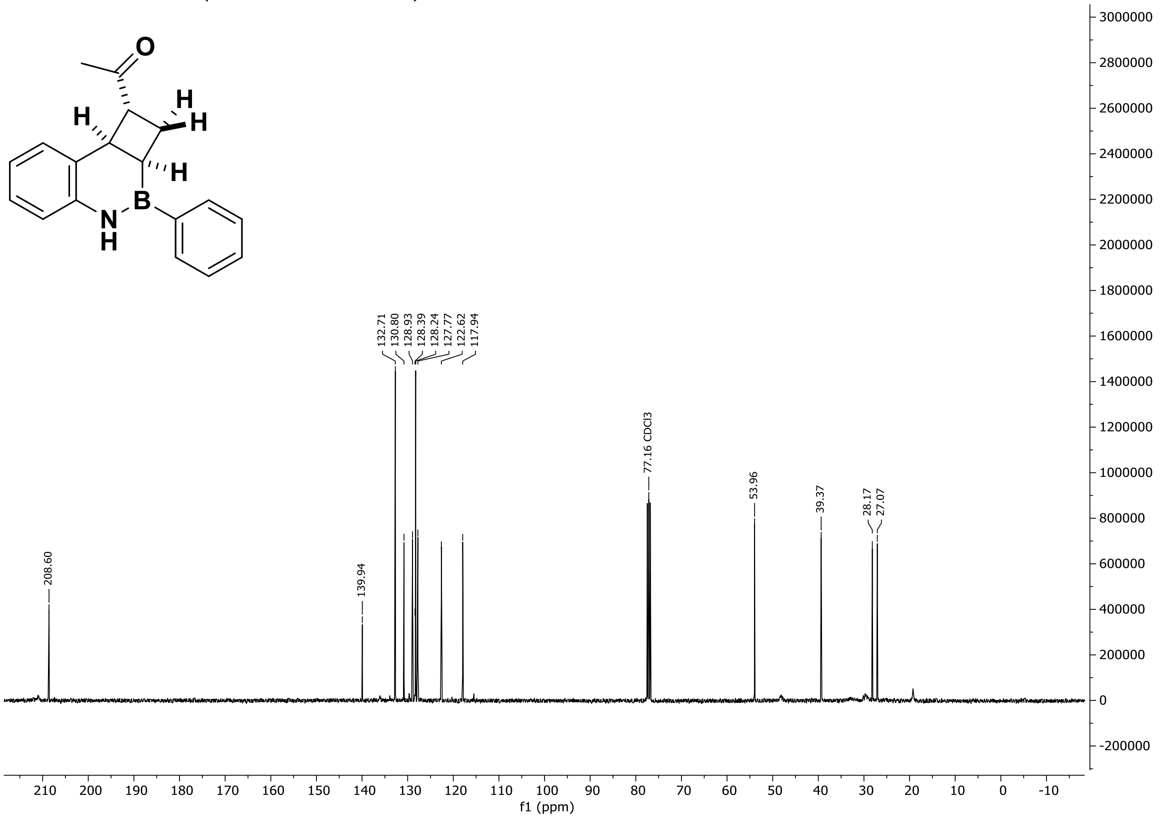

^{11}B-NMR of 17 (128 MHz, Acetone-d_6)

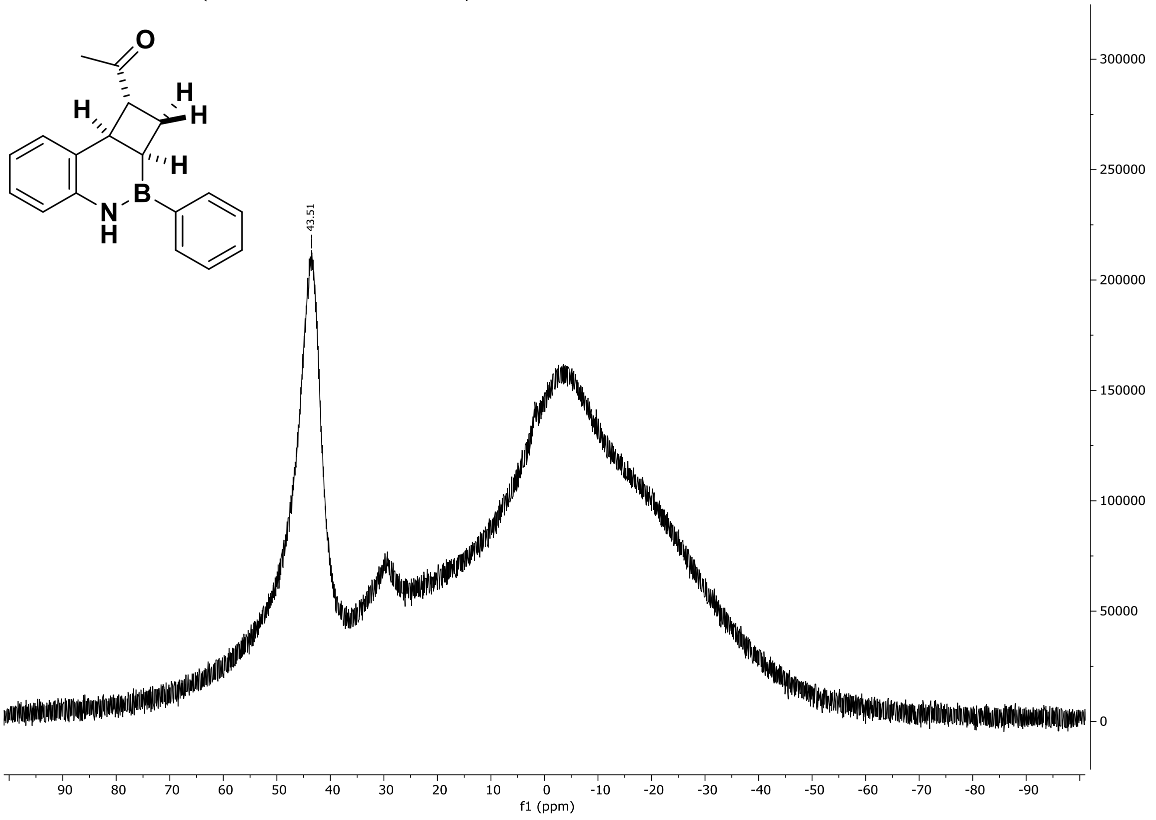

^{1}H-NMR of 18 (600 MHz, $CDCl_3$)

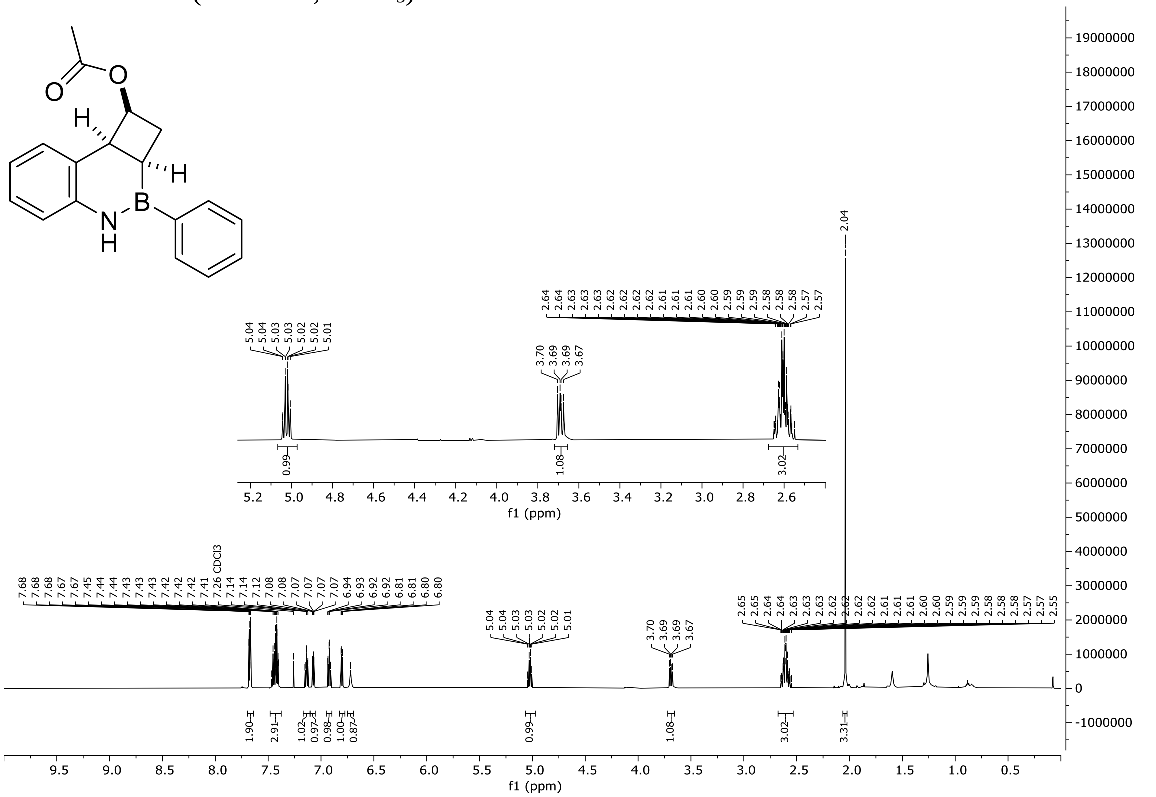

^{13}C-NMR of 18 (151 MHz, $CDCl_3$)

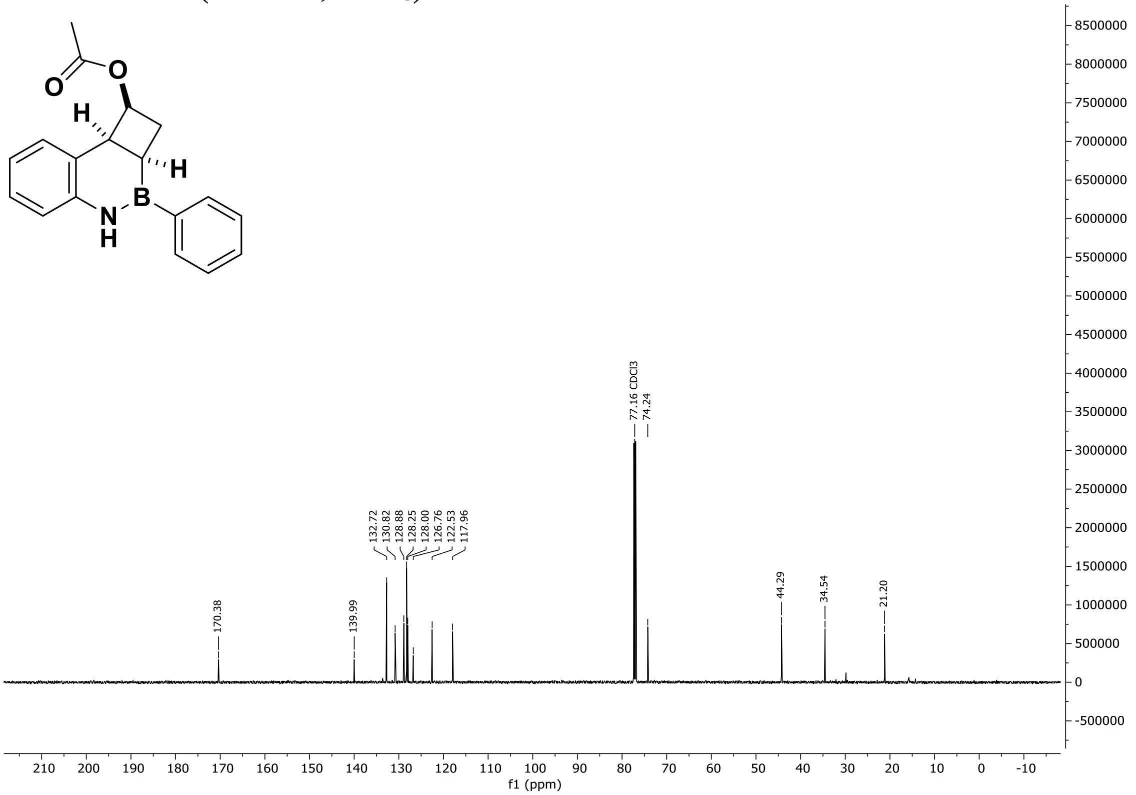

^{11}B-NMR of 18 (192 MHz, $CDCl_3$)

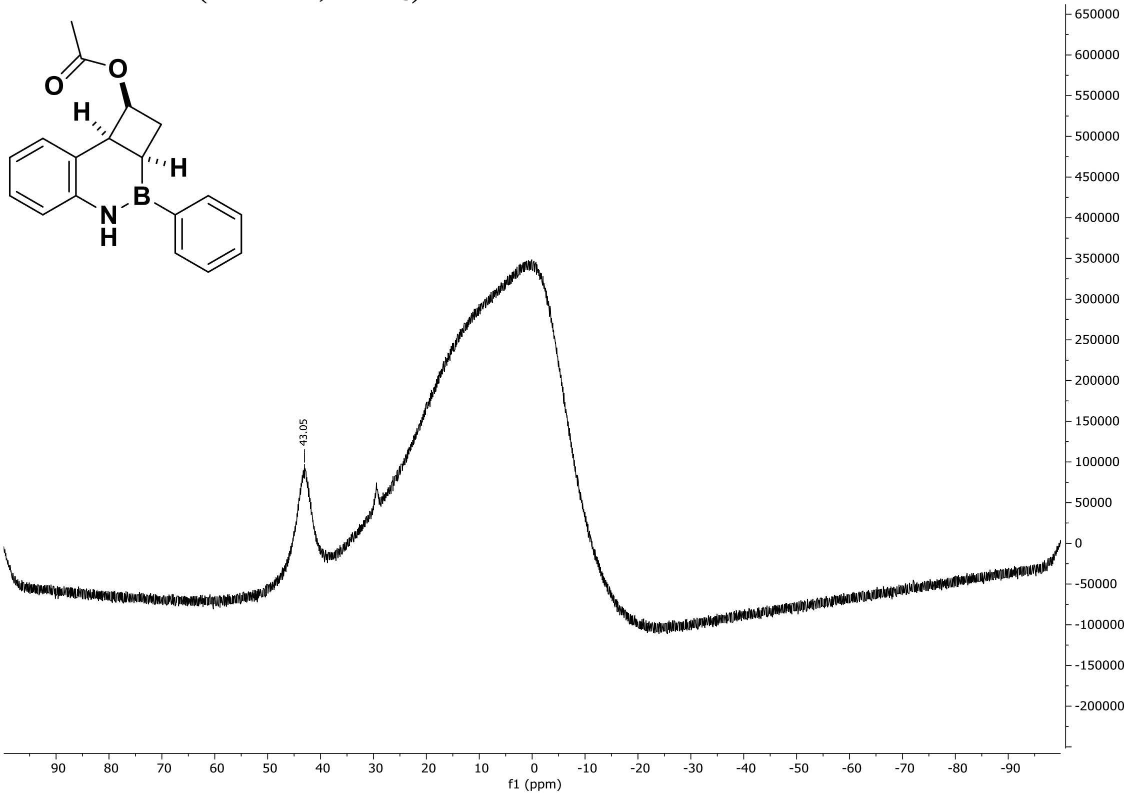

^{1}H-NMR of 19 (400 MHz, $CDCl_3$)

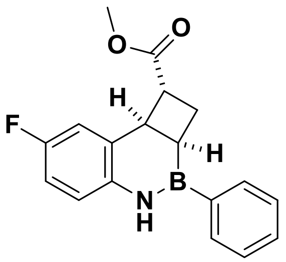

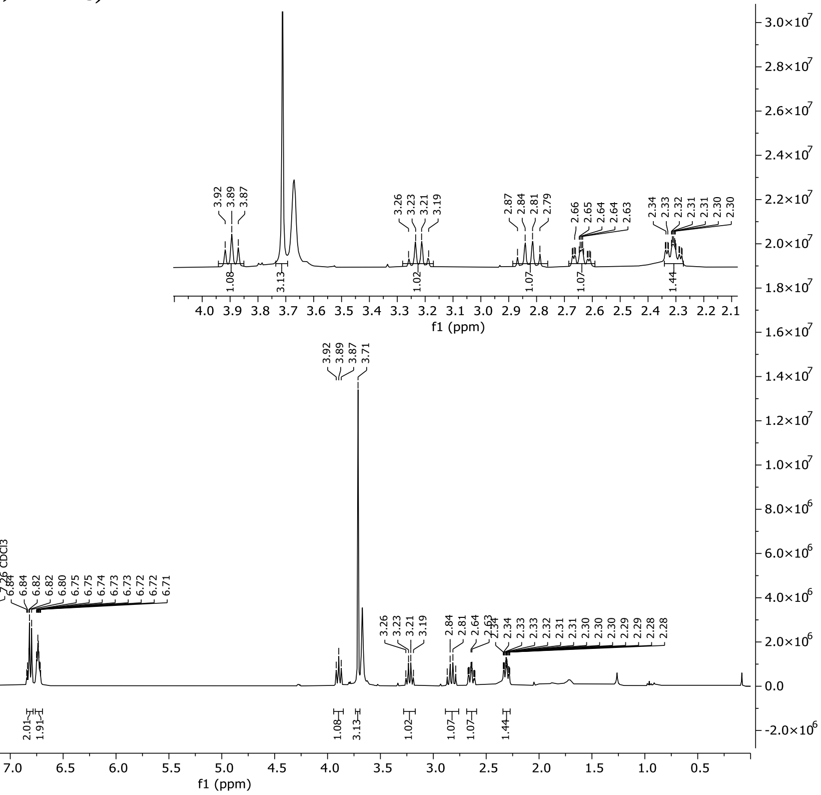

NOESY of 19

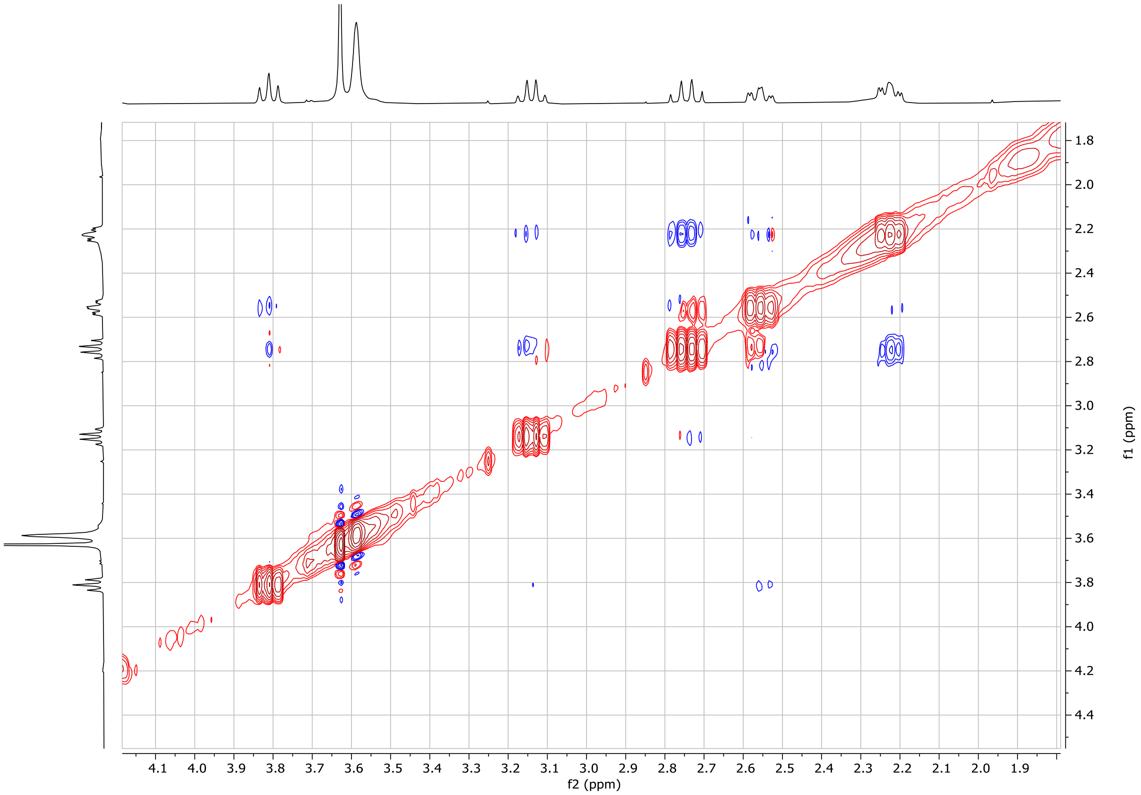

^{13}C-NMR of 19 (100 MHz, $CDCl_3$)

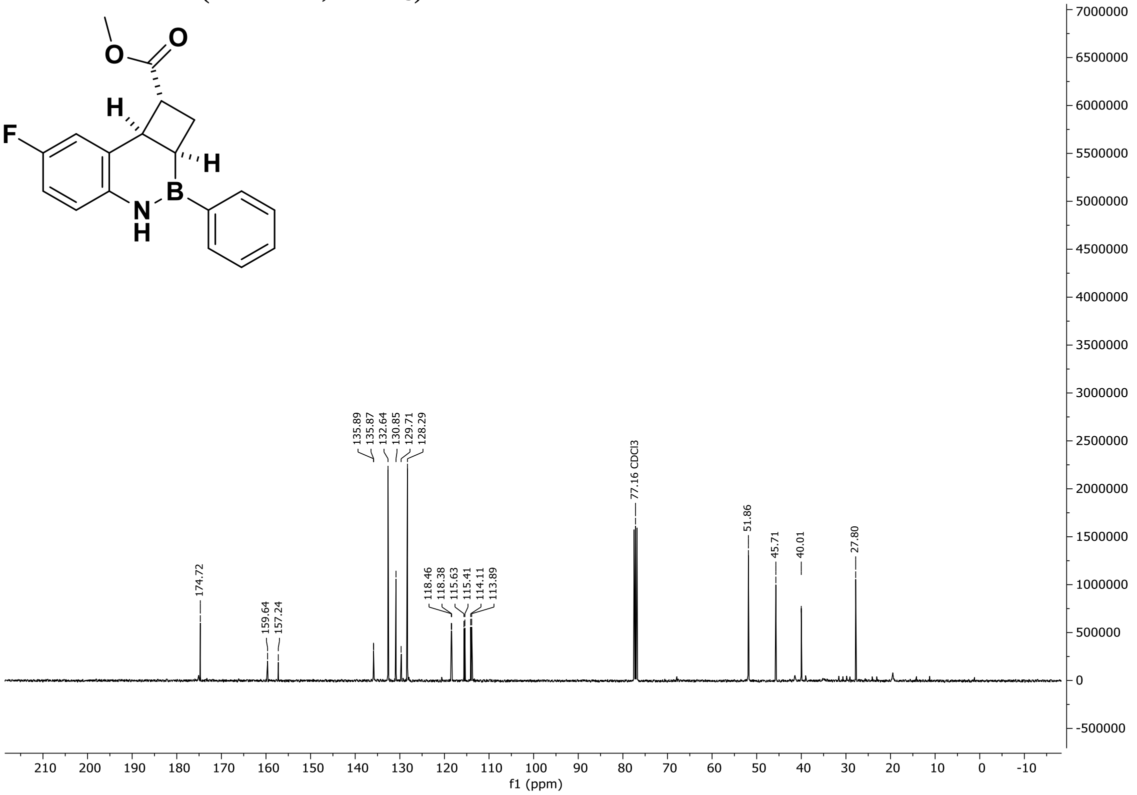

^{11}B-NMR of 19 (128 MHz, $CDCl_3$)

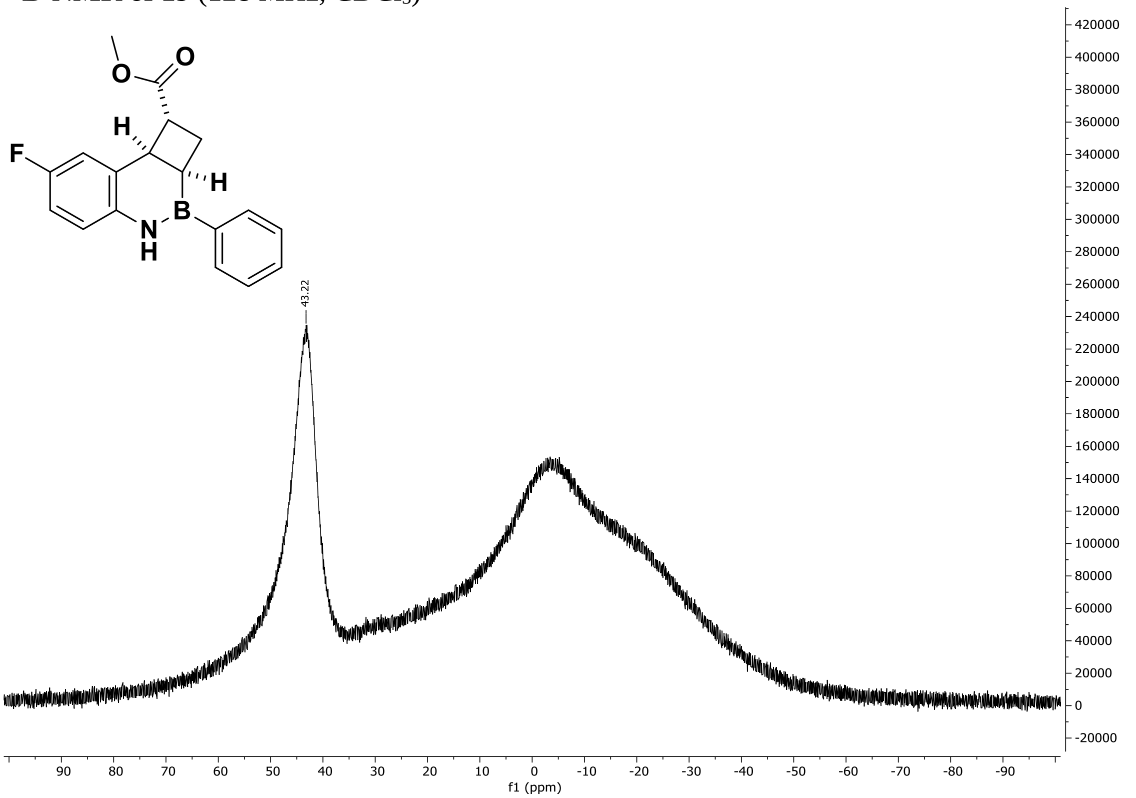

^{19}F-NMR of 19 (376 MHz, $CDCl_3$)

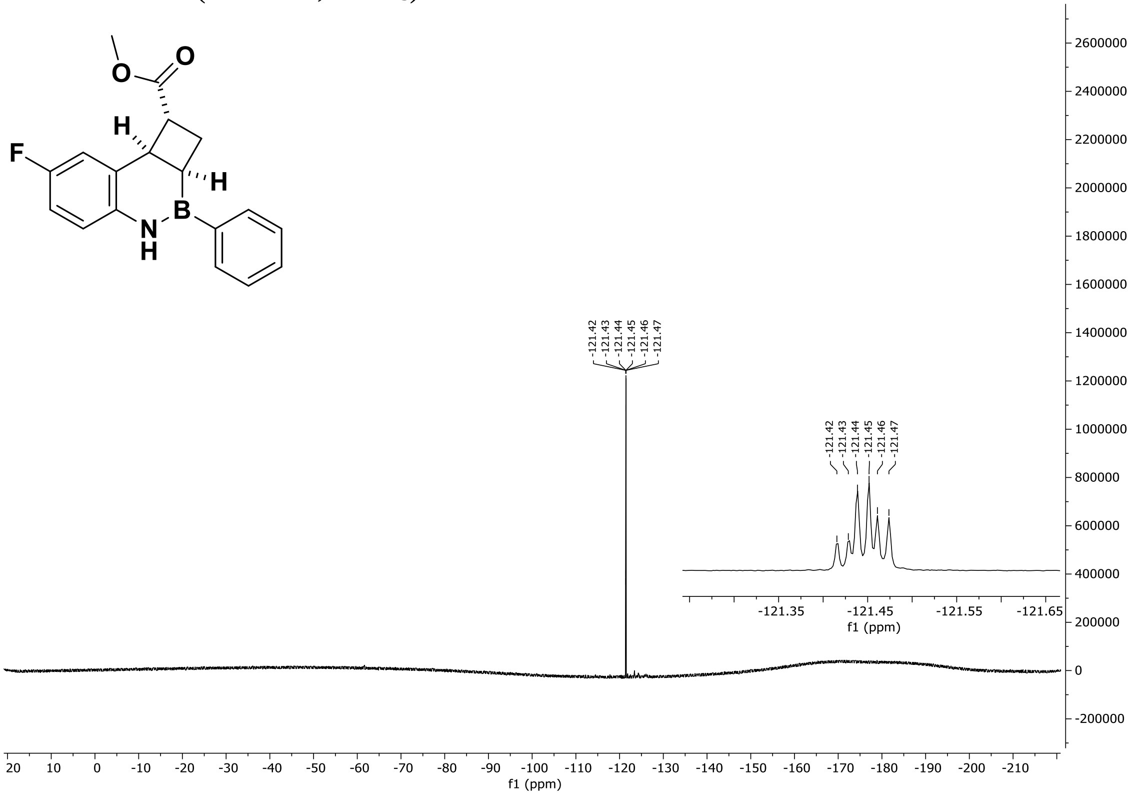

^{1}H-NMR of 20 (400 MHz, $CDCl_3$)

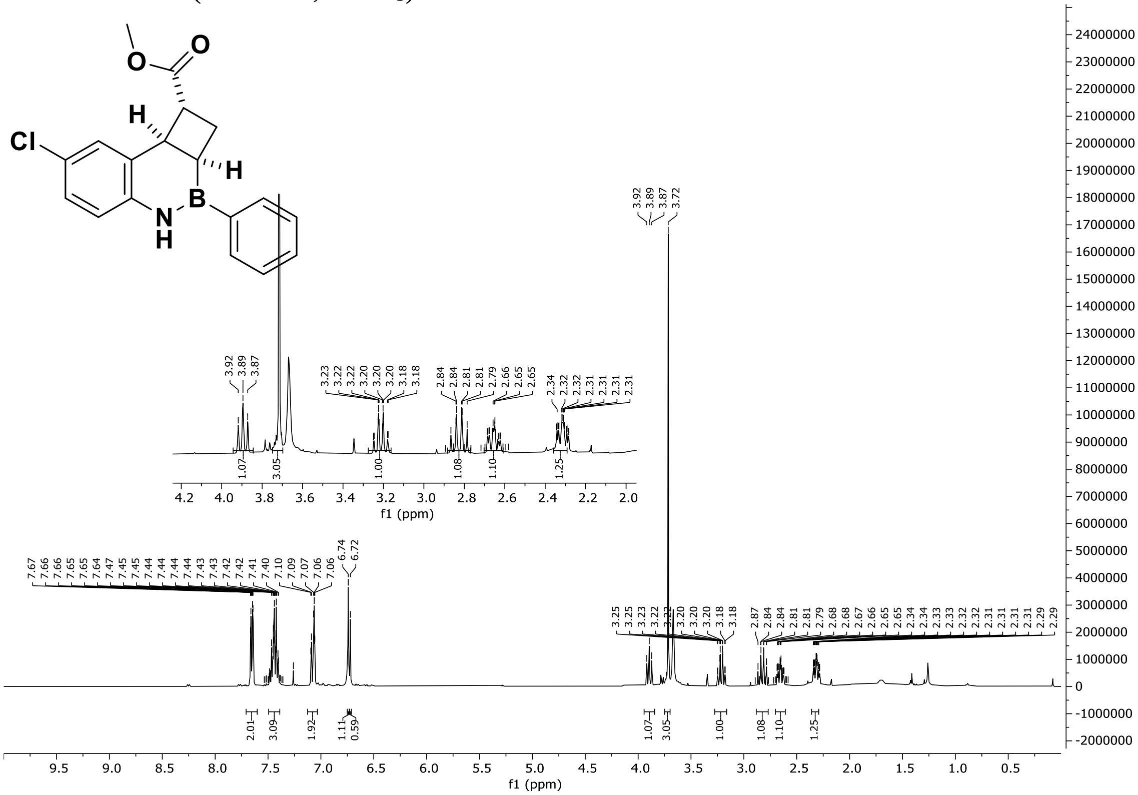

^{13}C-NMR of 20 (100 MHz, $CDCl_3$)

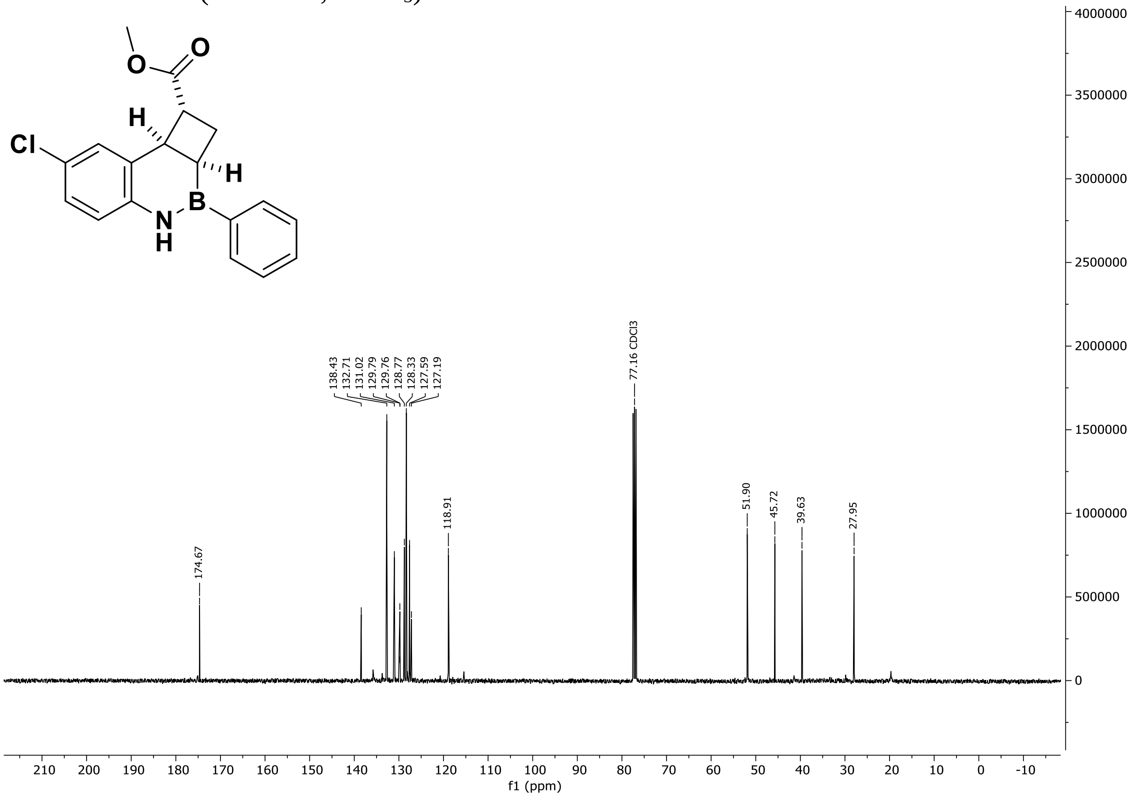

^{11}B-NMR of 20 (128 MHz, $CDCl_3$)

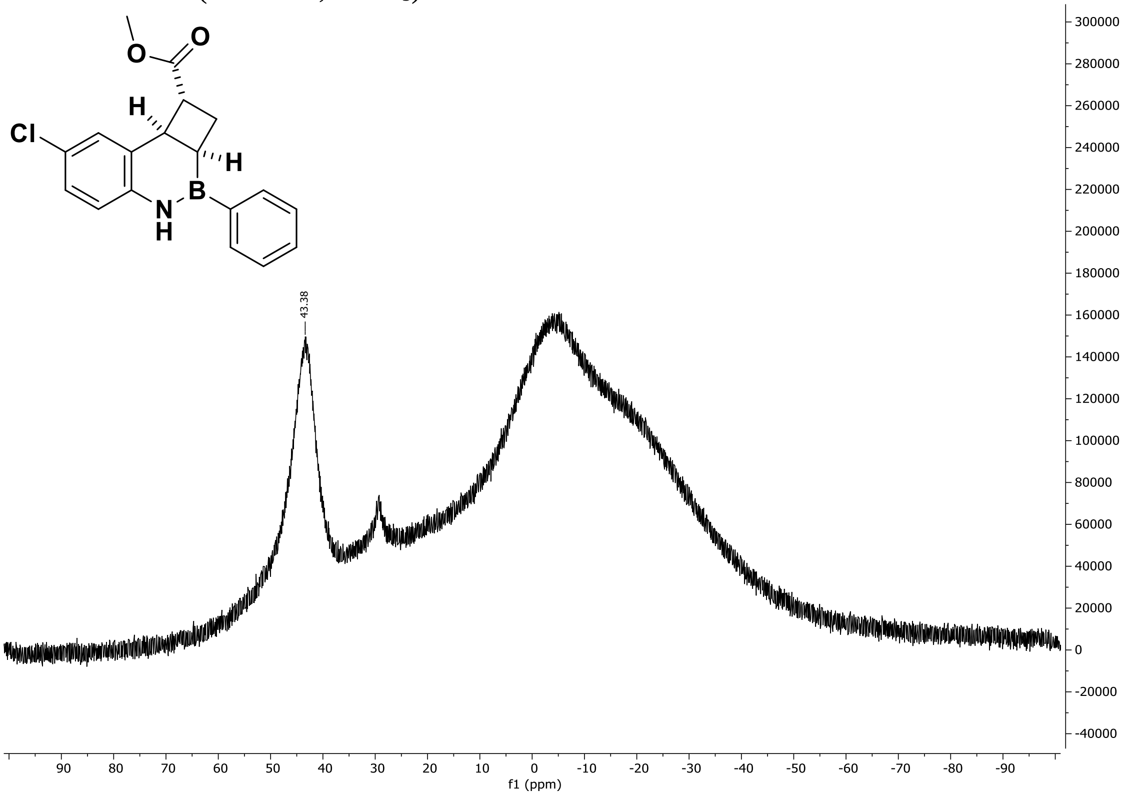

^{1}H-NMR of 21 (600 MHz, $CDCl_3$)

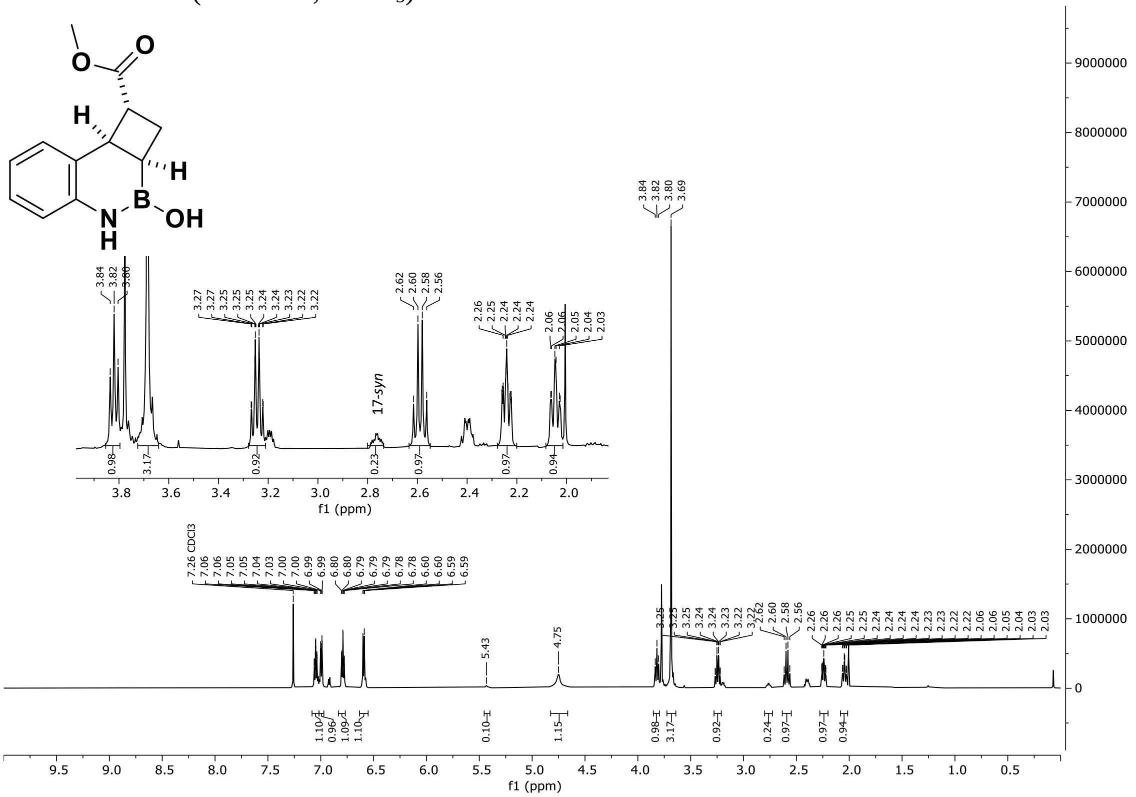

^{13}C-NMR of 21 (100 MHz, $CDCl_3$)

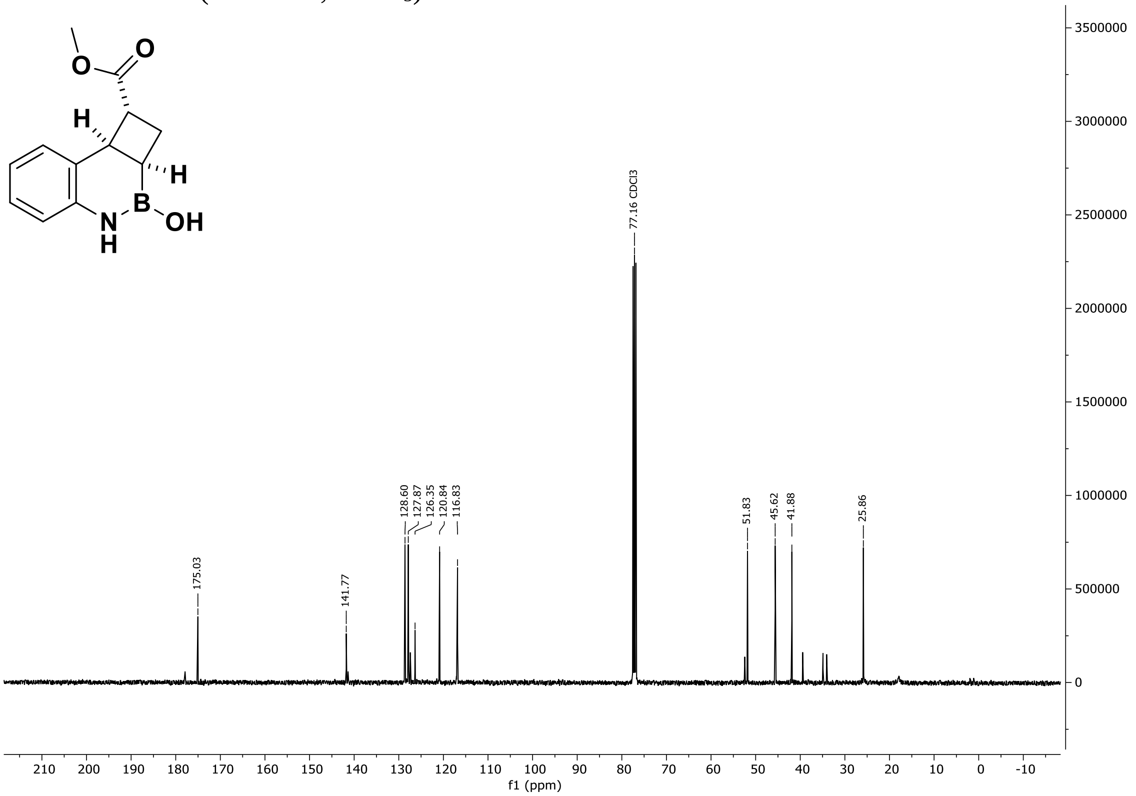

^{11}B-NMR of 21 (128 MHz, $CDCl_3$)

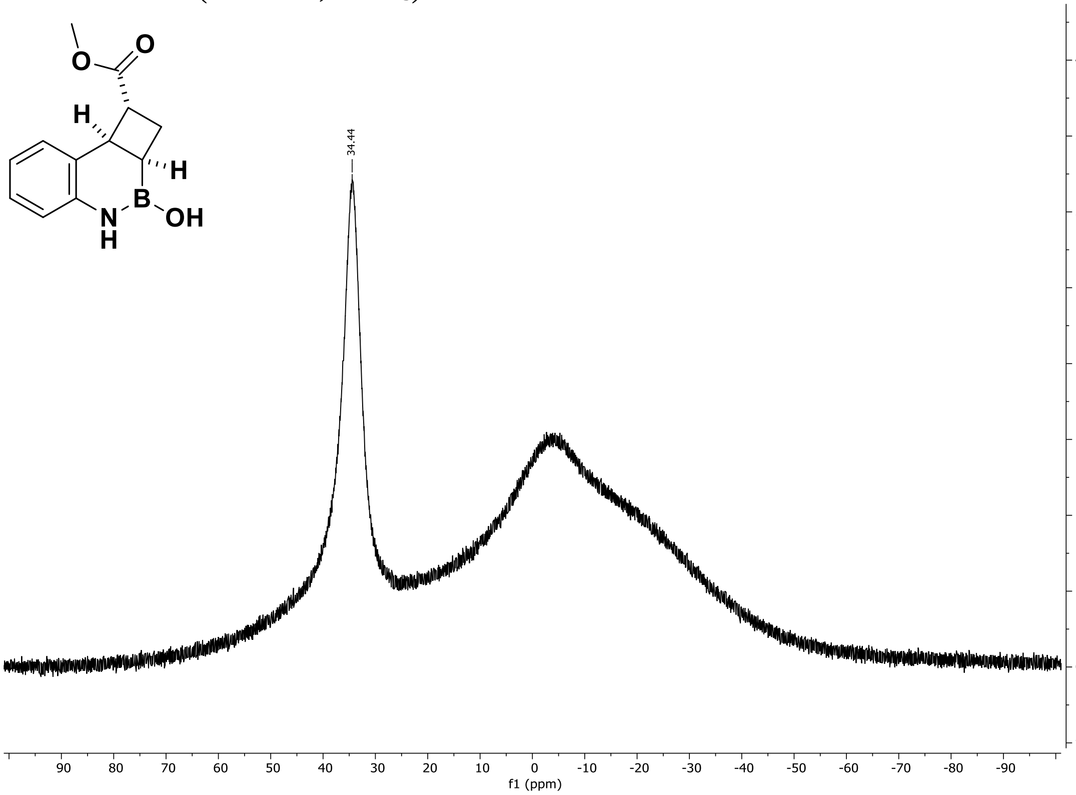

^{1}H-NMR of 22 (400 MHz, $CDCl_3$)

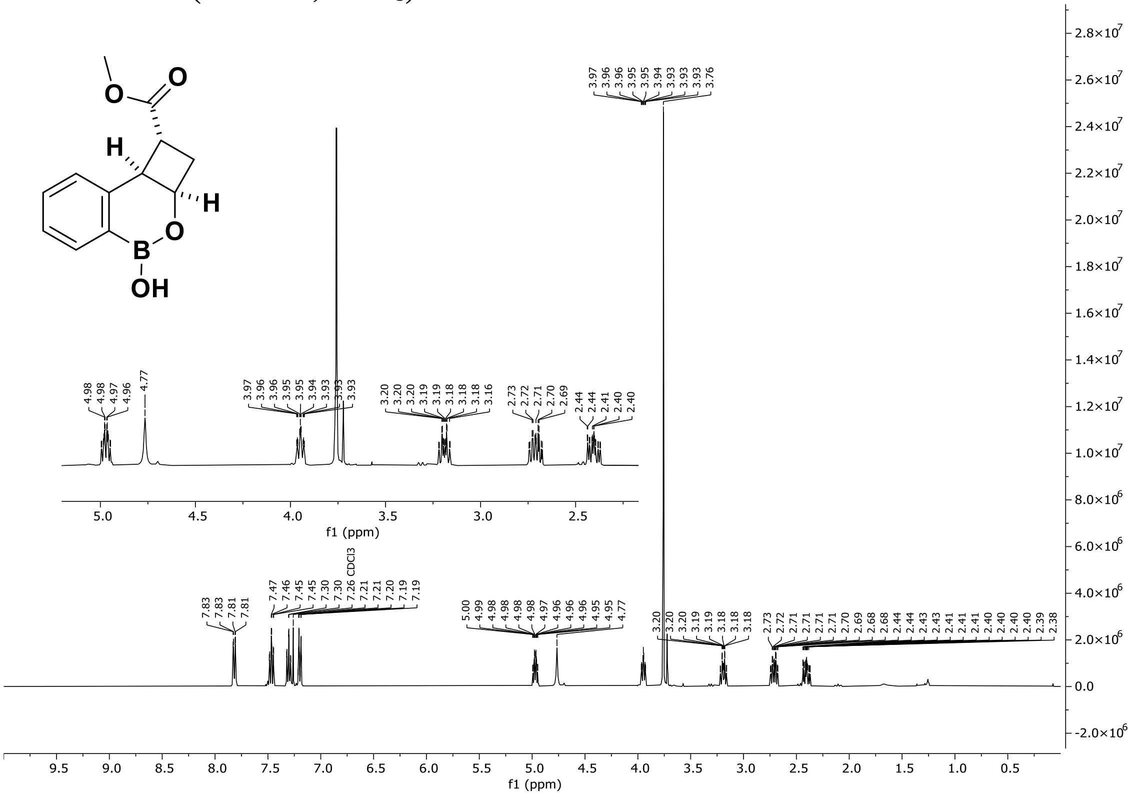

^{13}C-NMR of 22 (100 MHz, $CDCl_3$)

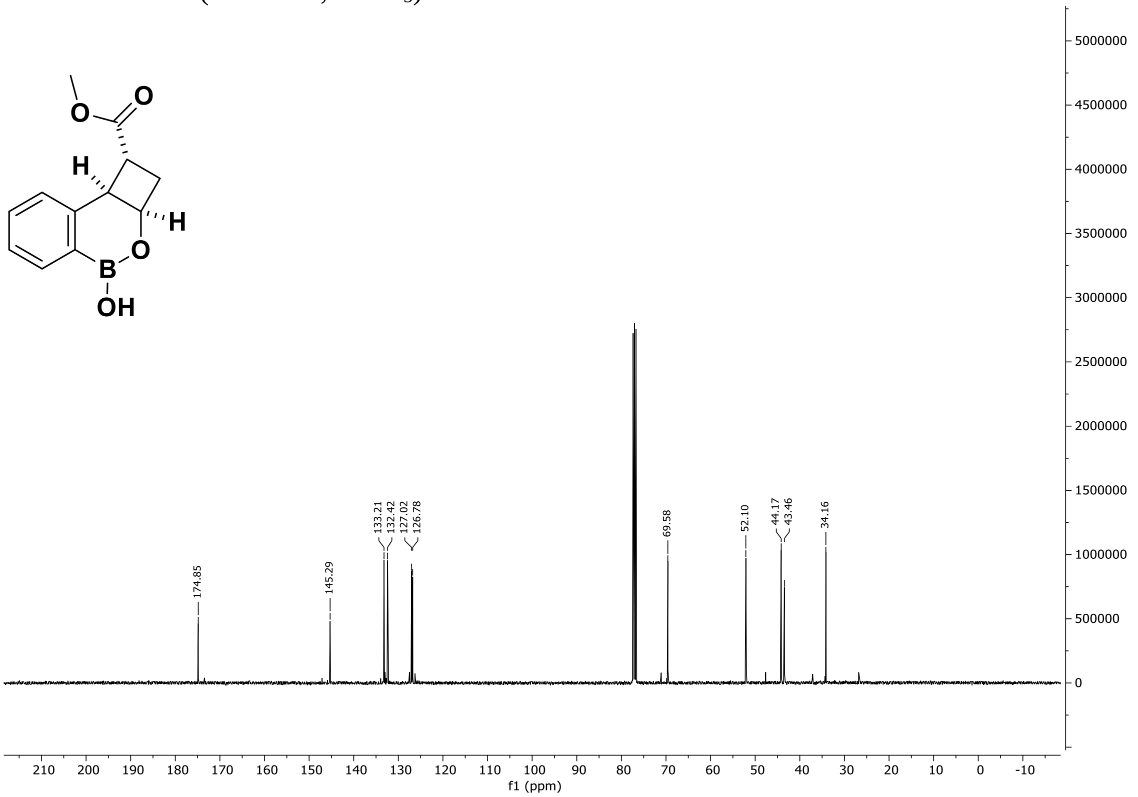

^{11}B-NMR of 22 (128 MHz, $CDCl_3$)

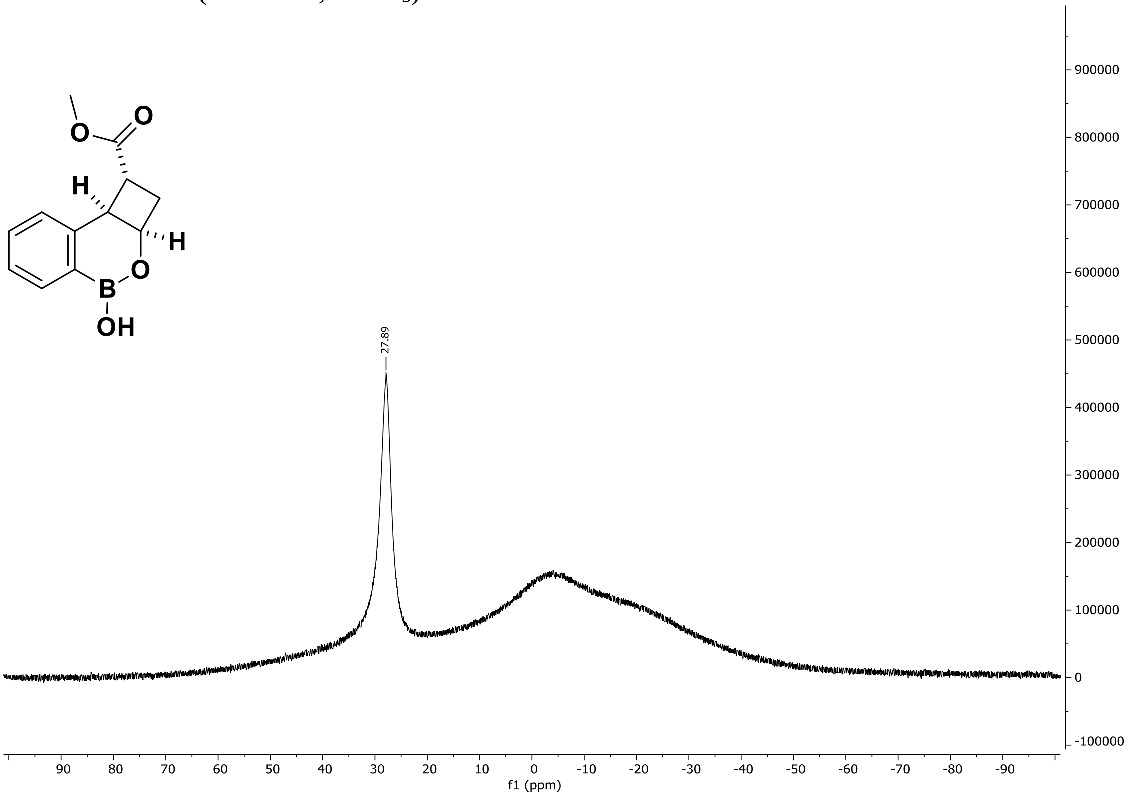

^{1}H-NMR of 23 (400 MHz, $CDCl_3$)

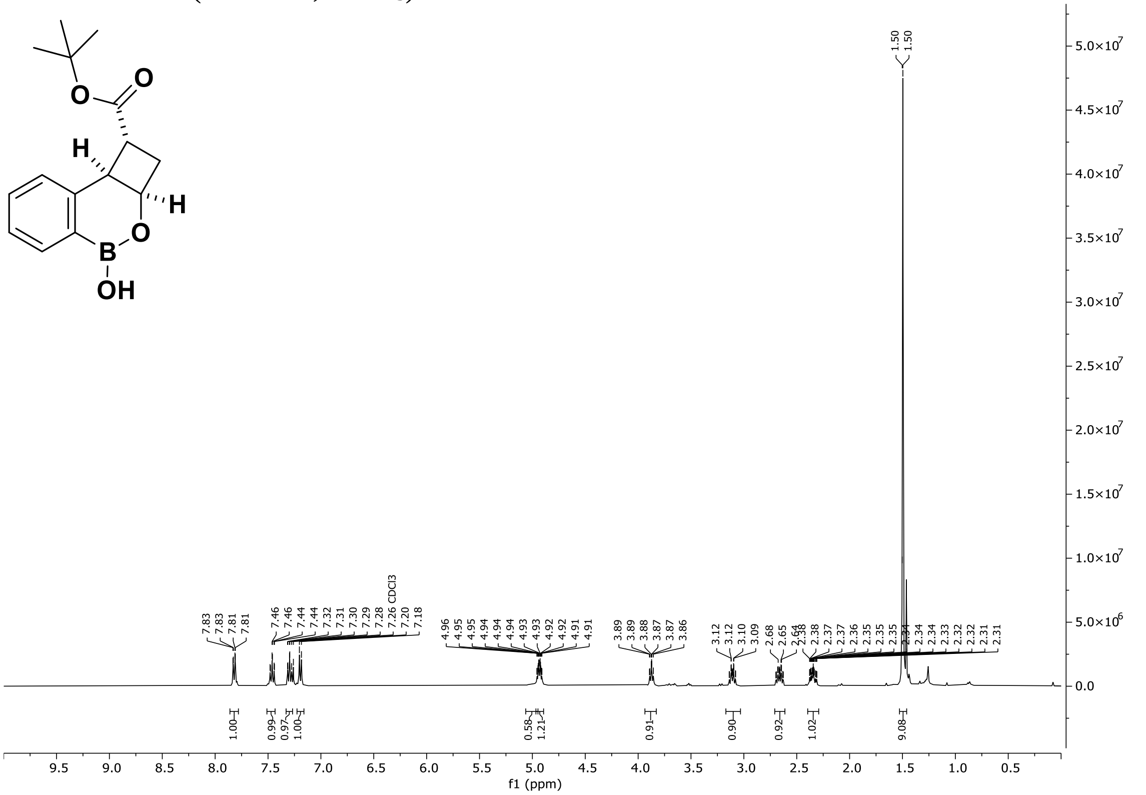

^{13}C-NMR of 23 (100 MHz, $CDCl_3$)

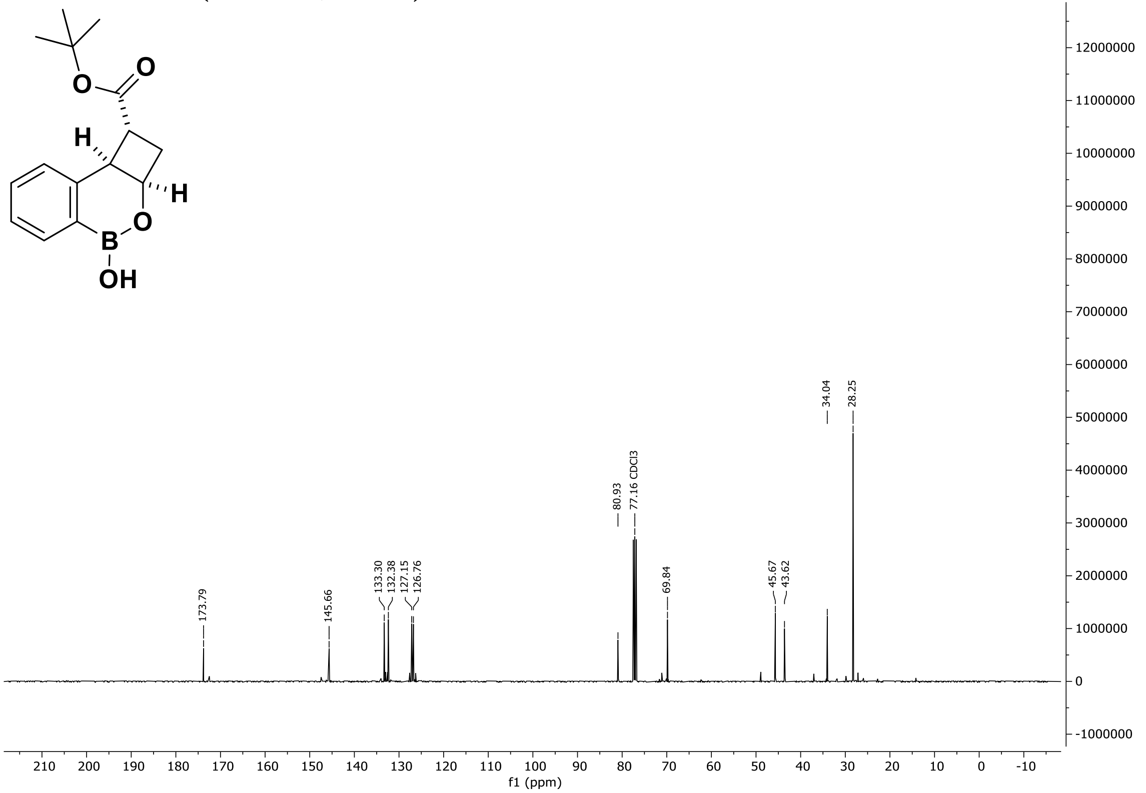

^{11}B-NMR of 23 (128 MHz, $CDCl_3$)

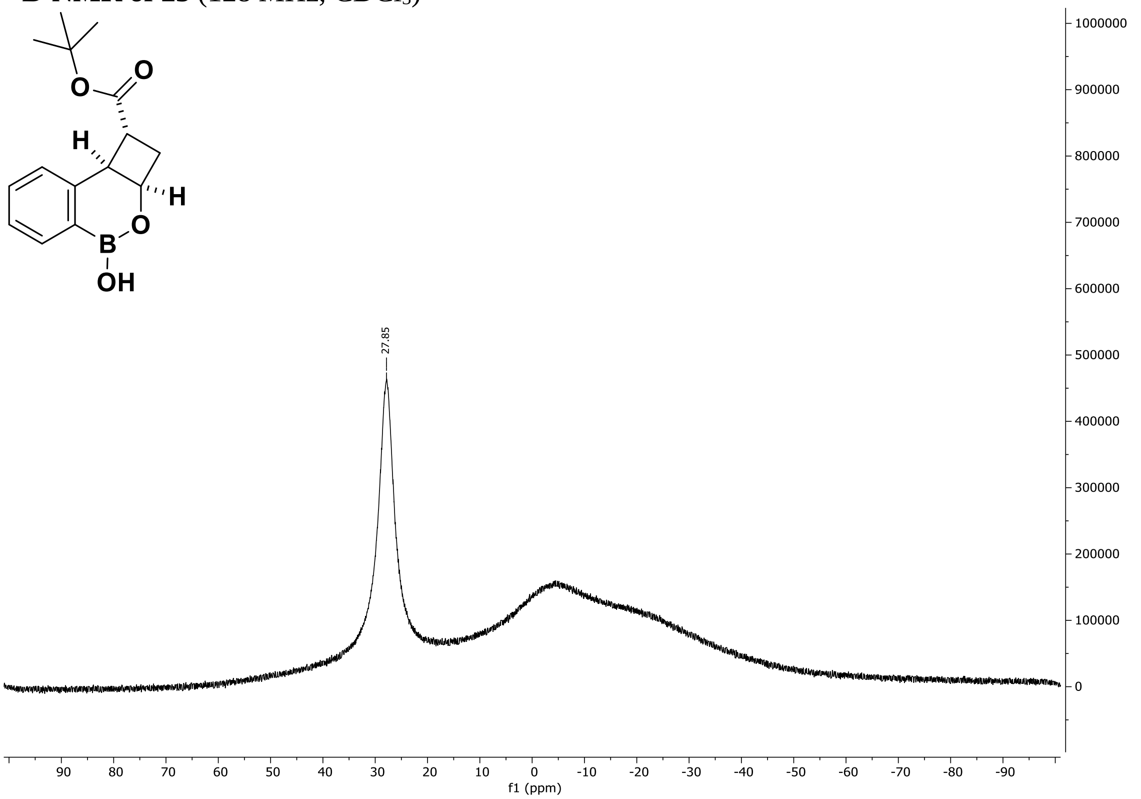

^{1}H-NMR of 24 (400 MHz, $CDCl_3$)

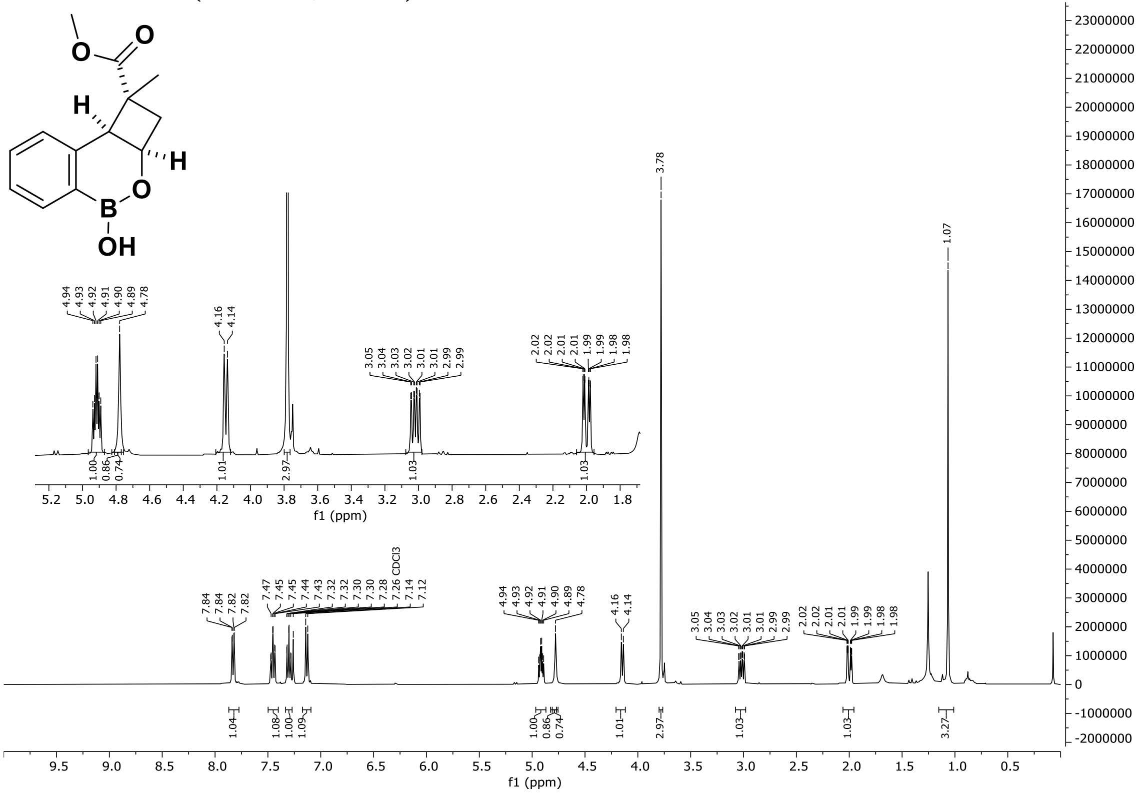

NOESY of 24

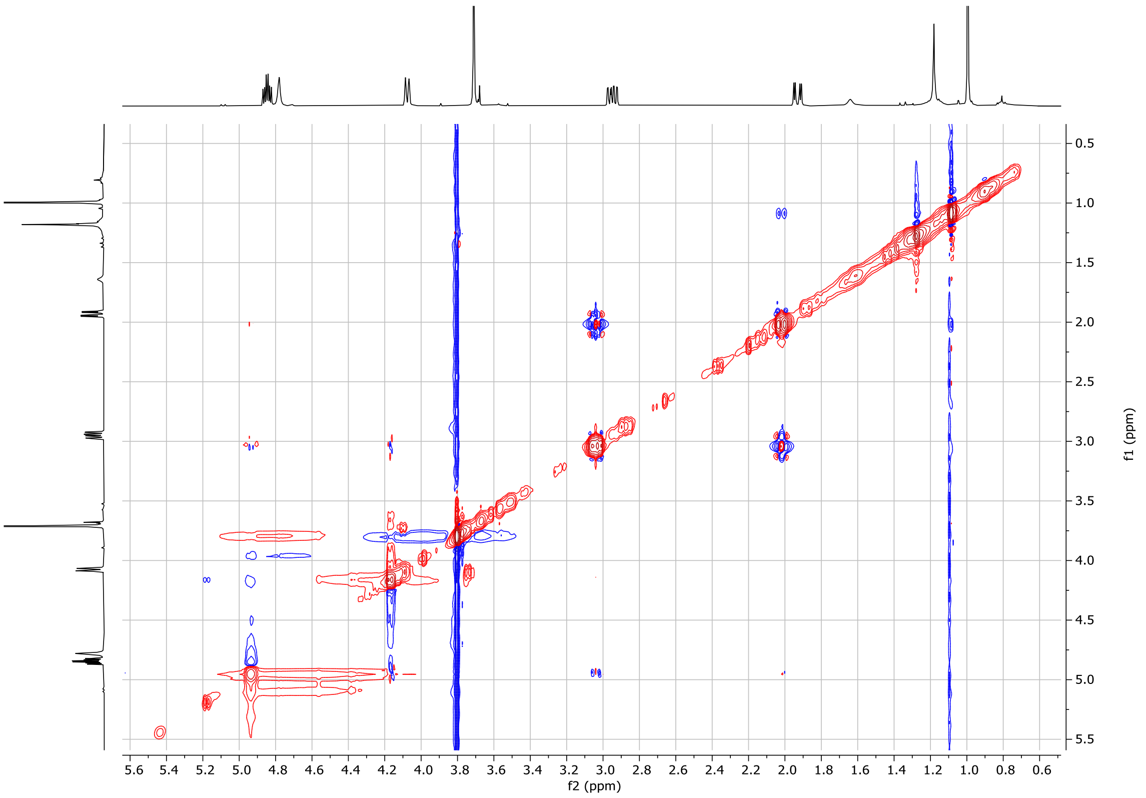

^{13}C-NMR of 24 (100 MHz, $CDCl_3$)

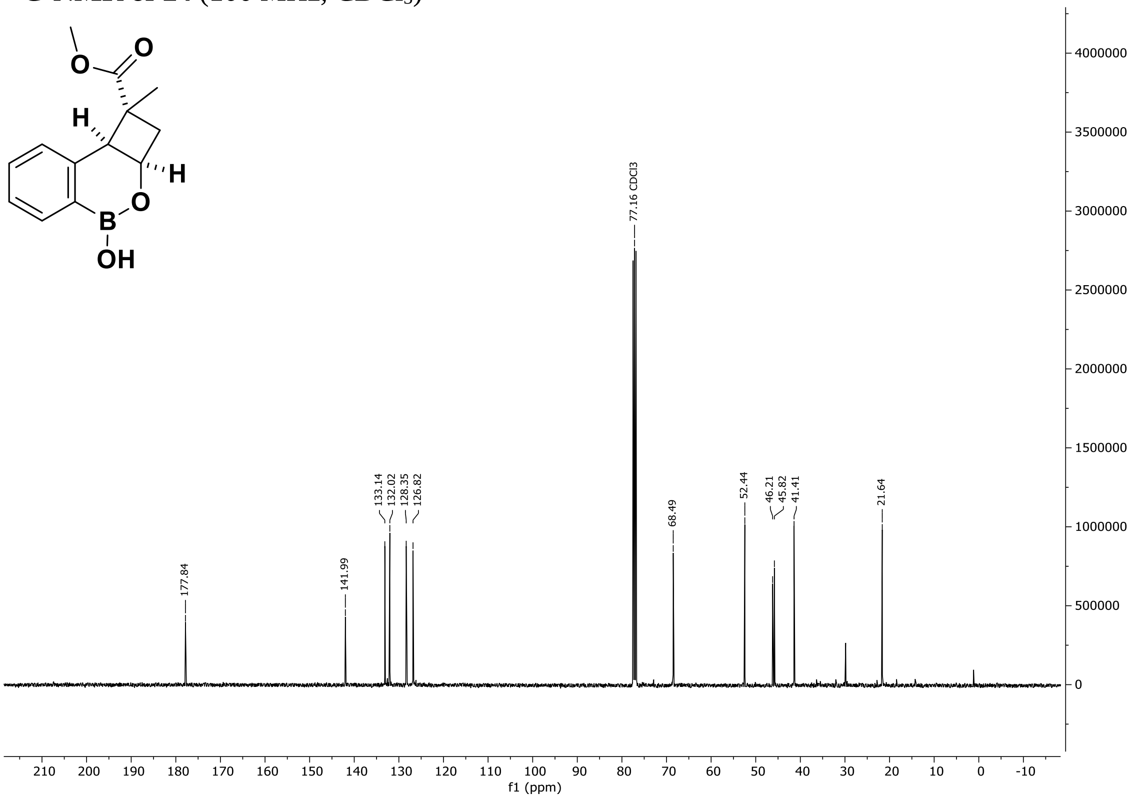

^{11}B-NMR of 24 (128 MHz, $CDCl_3$)

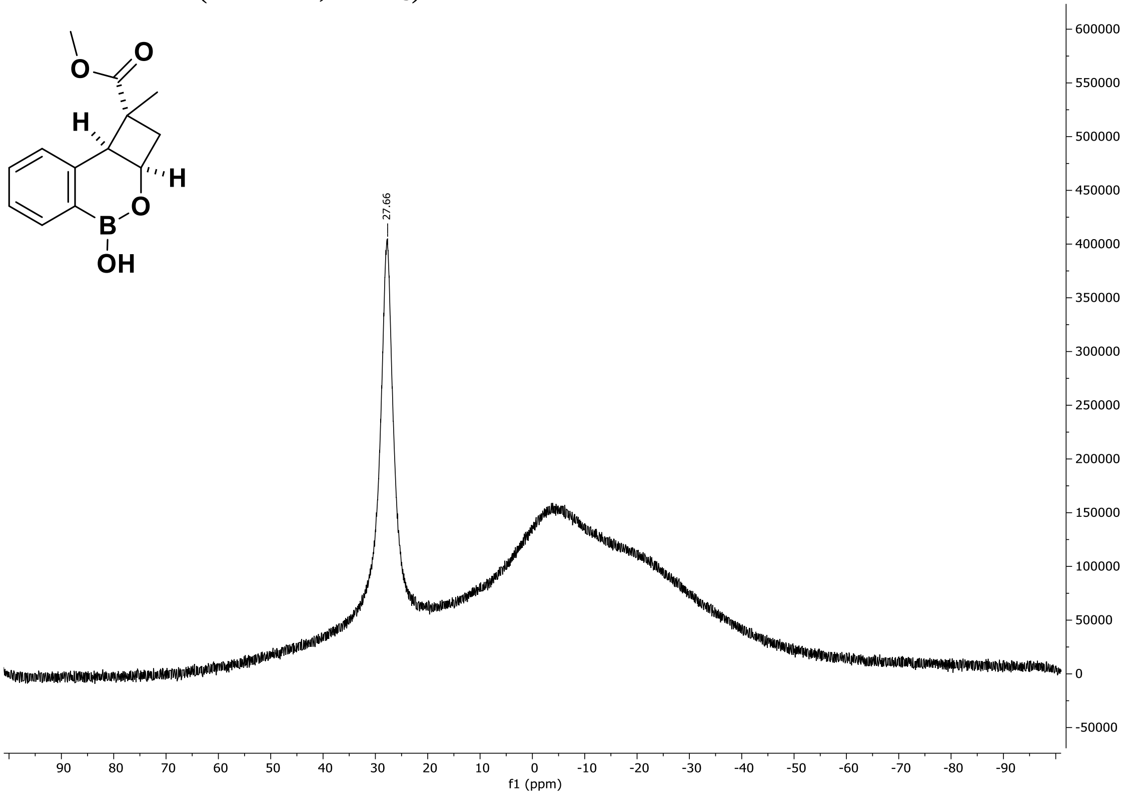

^{1}H-NMR of 25 (400 MHz, $CDCl_3$)

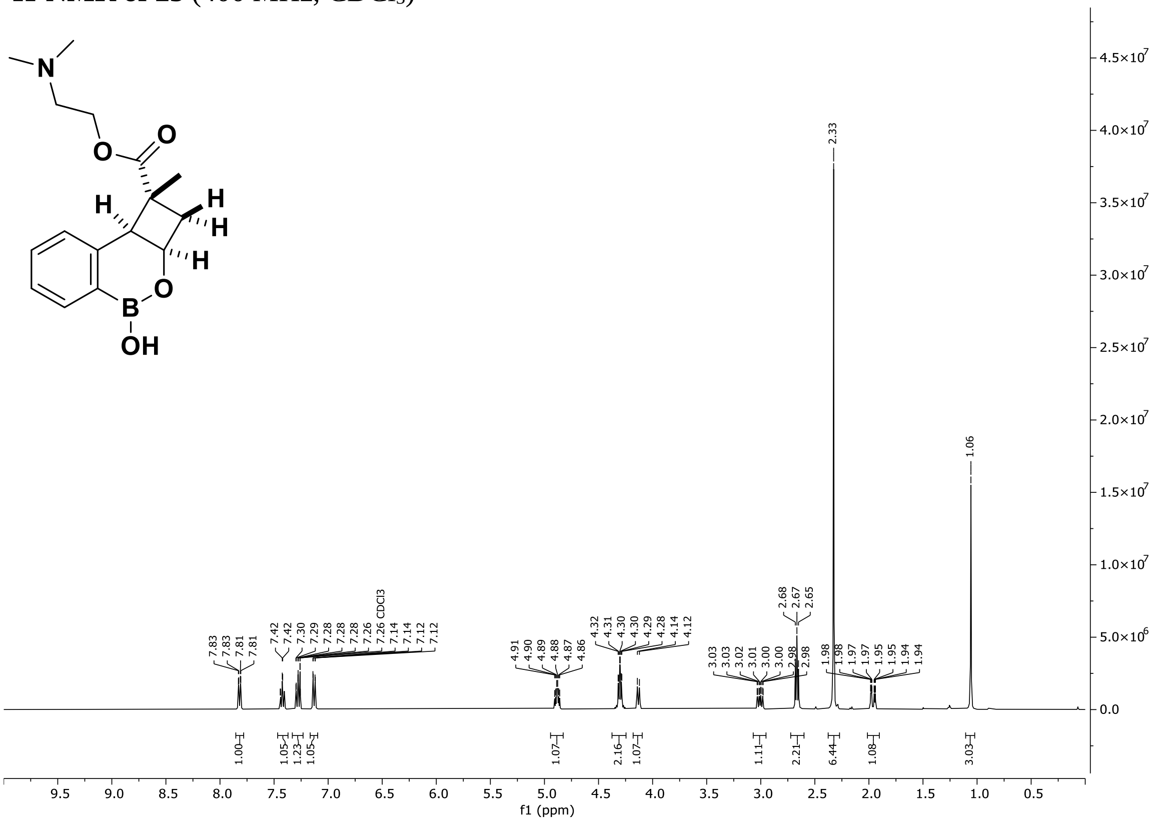

^{13}C-NMR of 25 (100 MHz, $CDCl_3$)

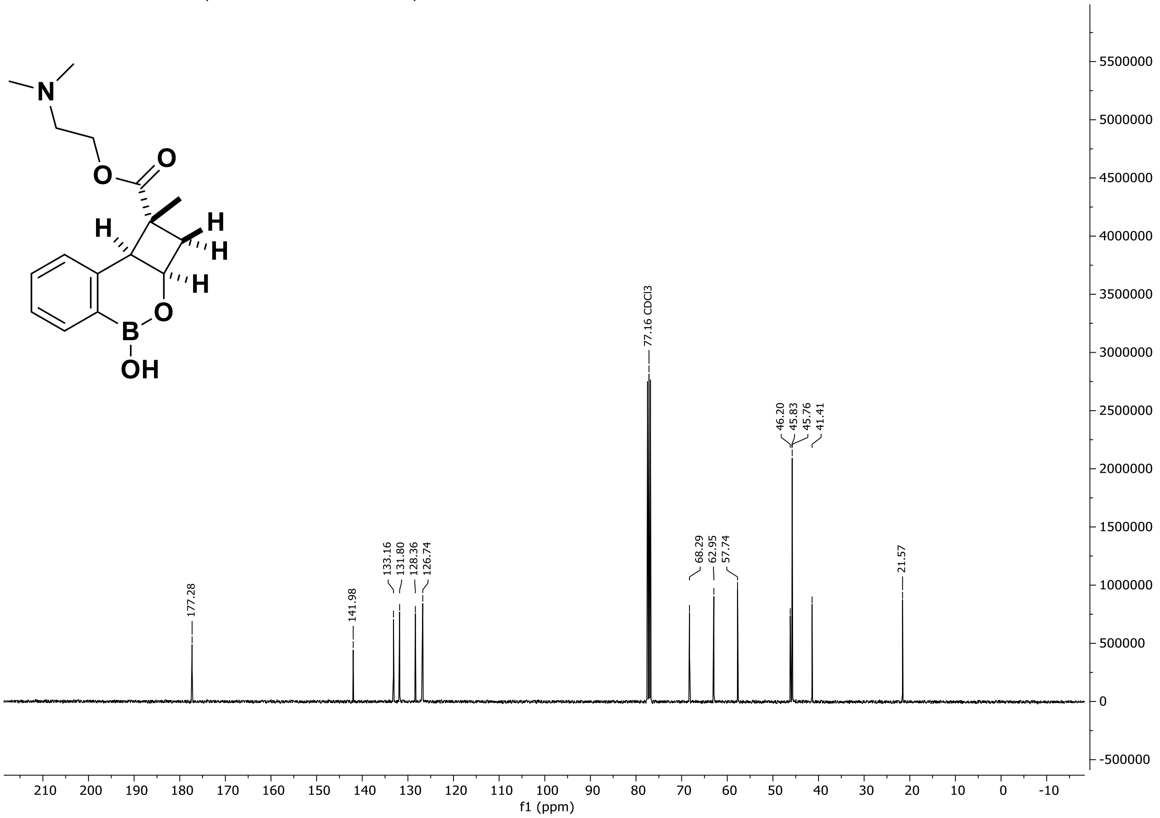

^{11}B-NMR of 25 (128 MHz $CDCl_3$)

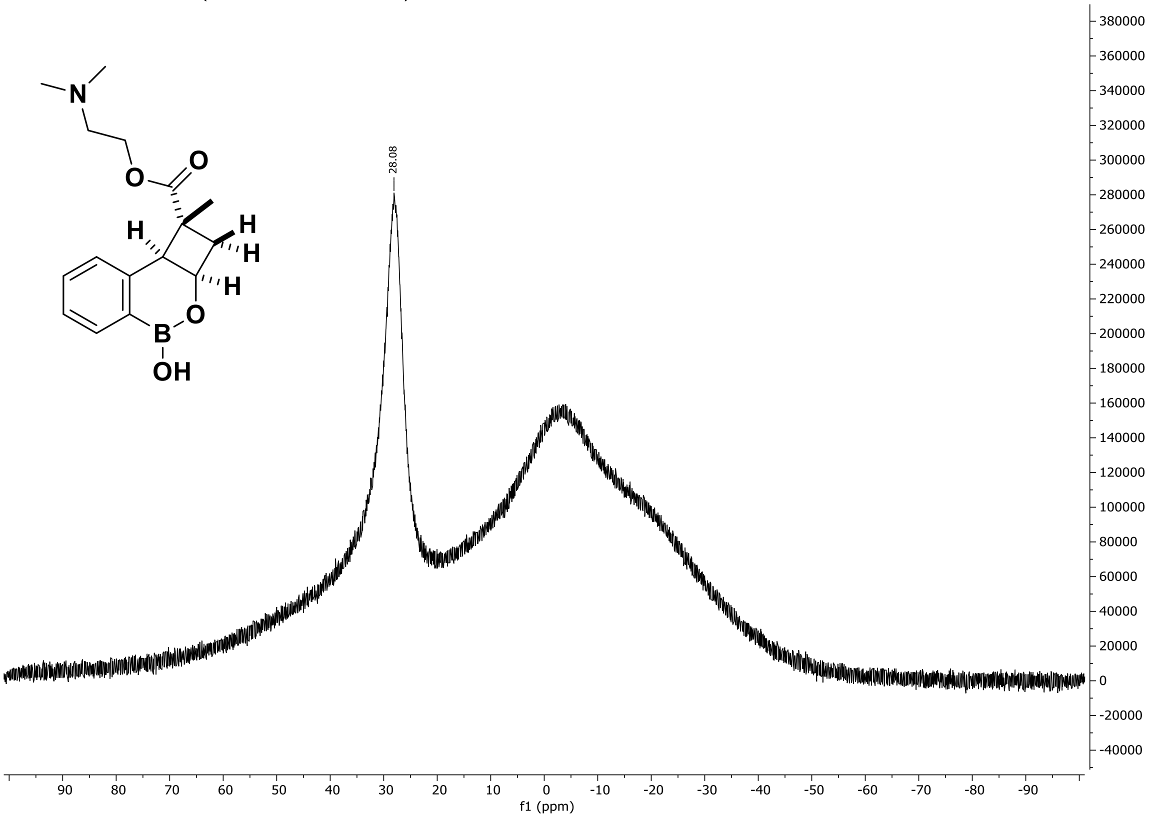

^{1}H-NMR of 26 (400 MHz, $CDCl_3$)

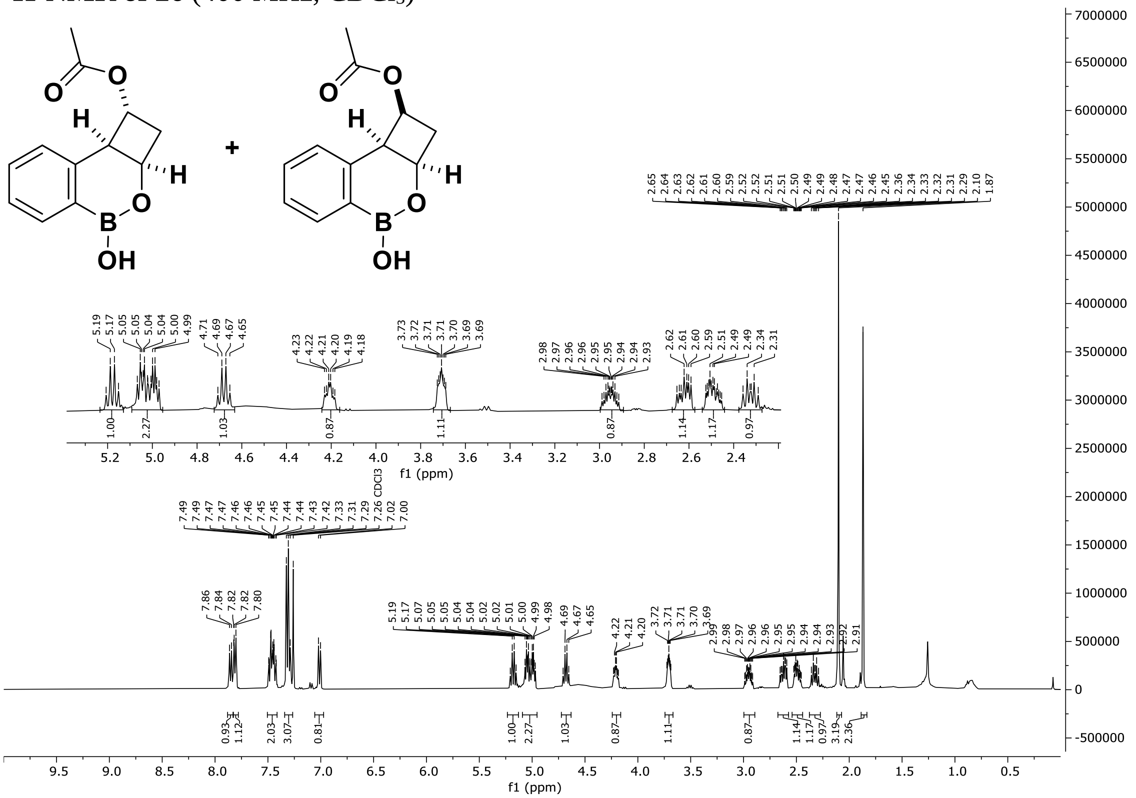

^{13}C-NMR of 26 (100 MHz, $CDCl_3$)

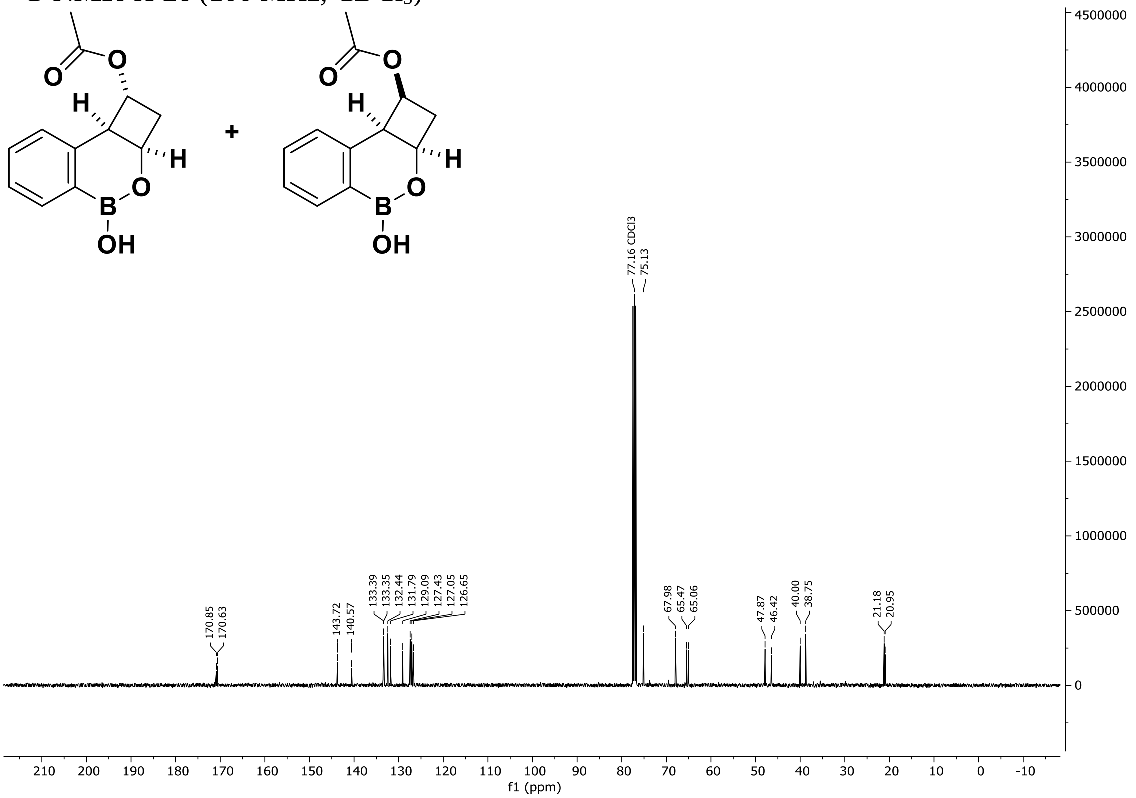

^{11}B-NMR of 26 (128 MHz, $CDCl_3$)

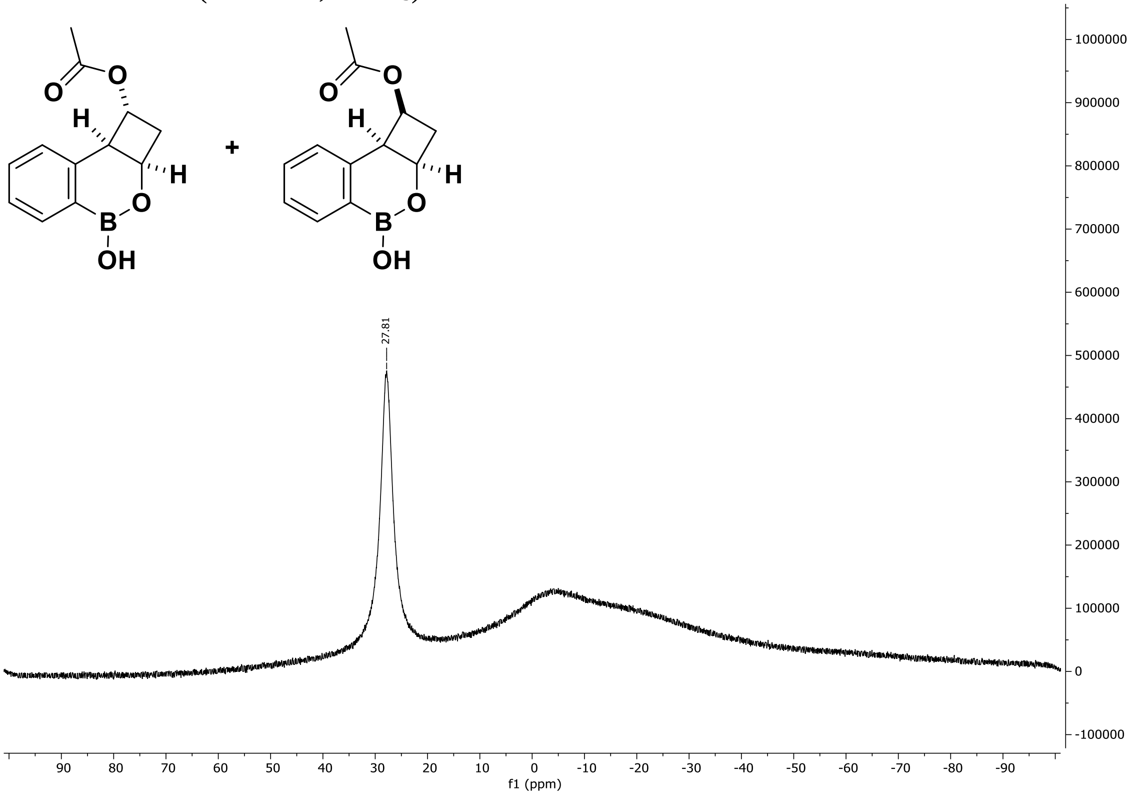

^{1}H-NMR of 27 (400 MHz, $CDCl_3$)

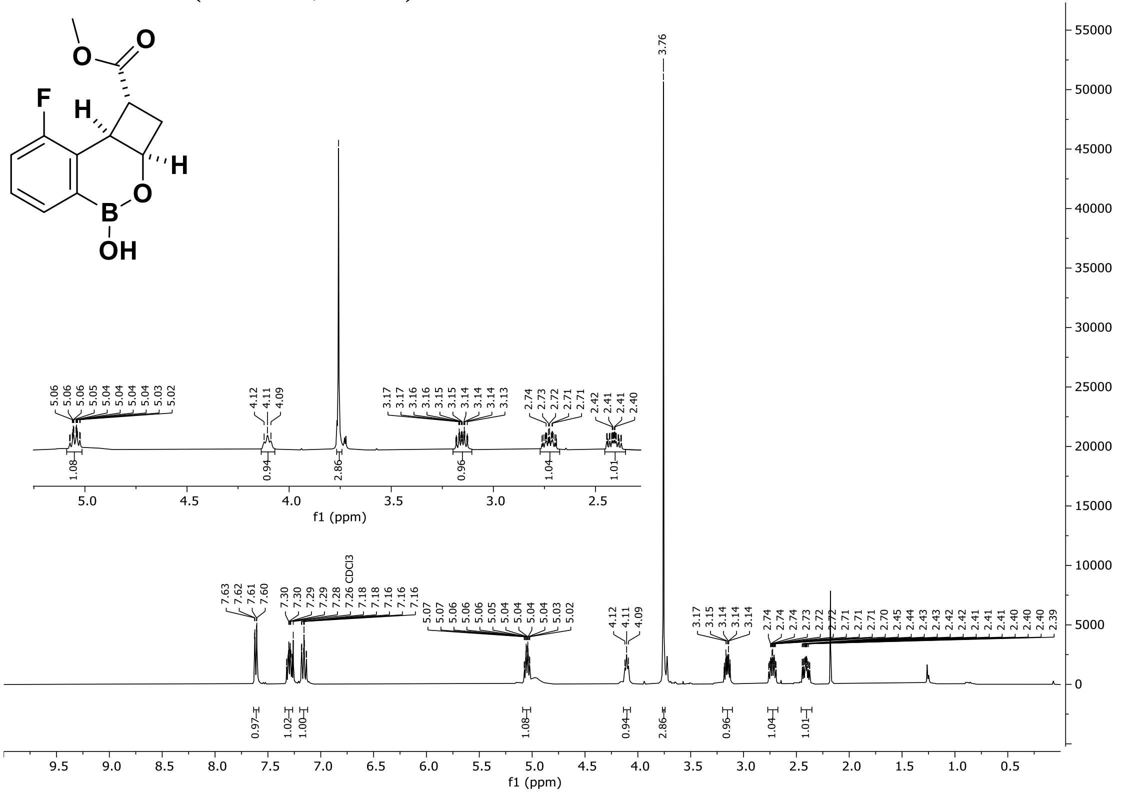

^{13}C-NMR of 27 (101 MHz, $CDCl_3$)

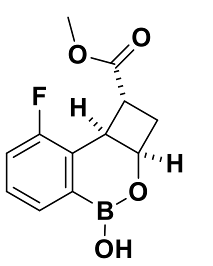

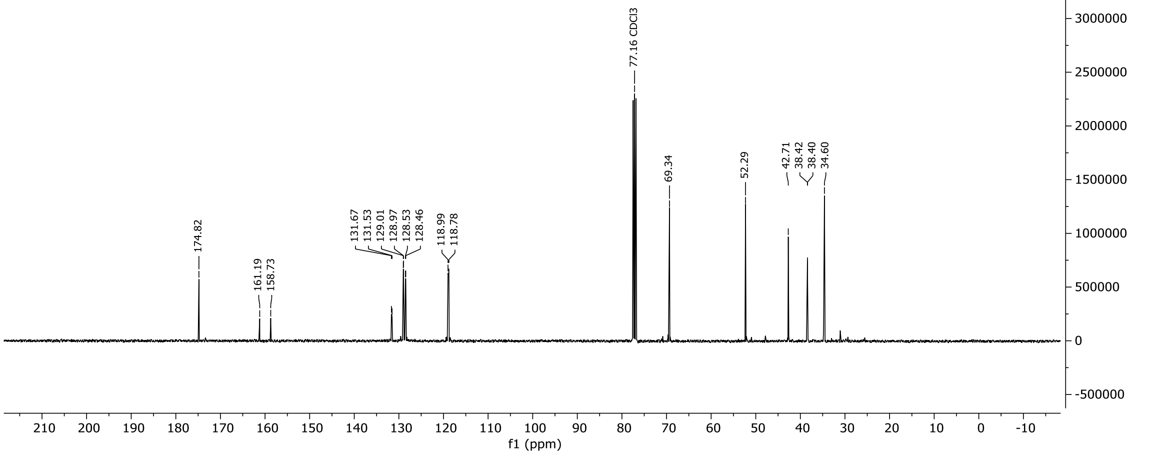

^{11}B-NMR of 27 (128 MHz, $CDCl_3$)

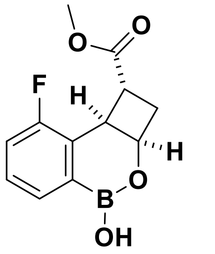

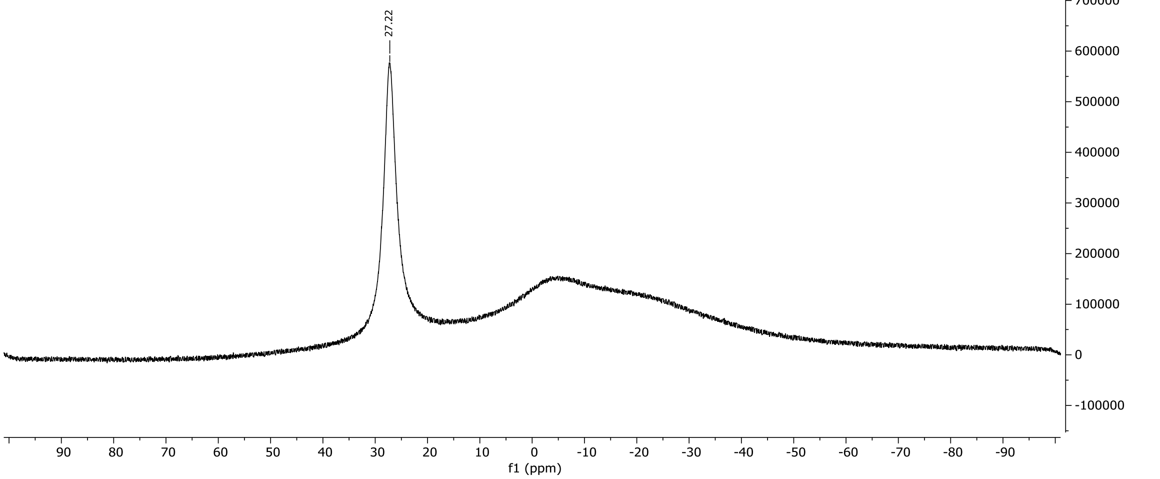

^{19}F-NMR of 27 (376 MHz, $CDCl_3$)

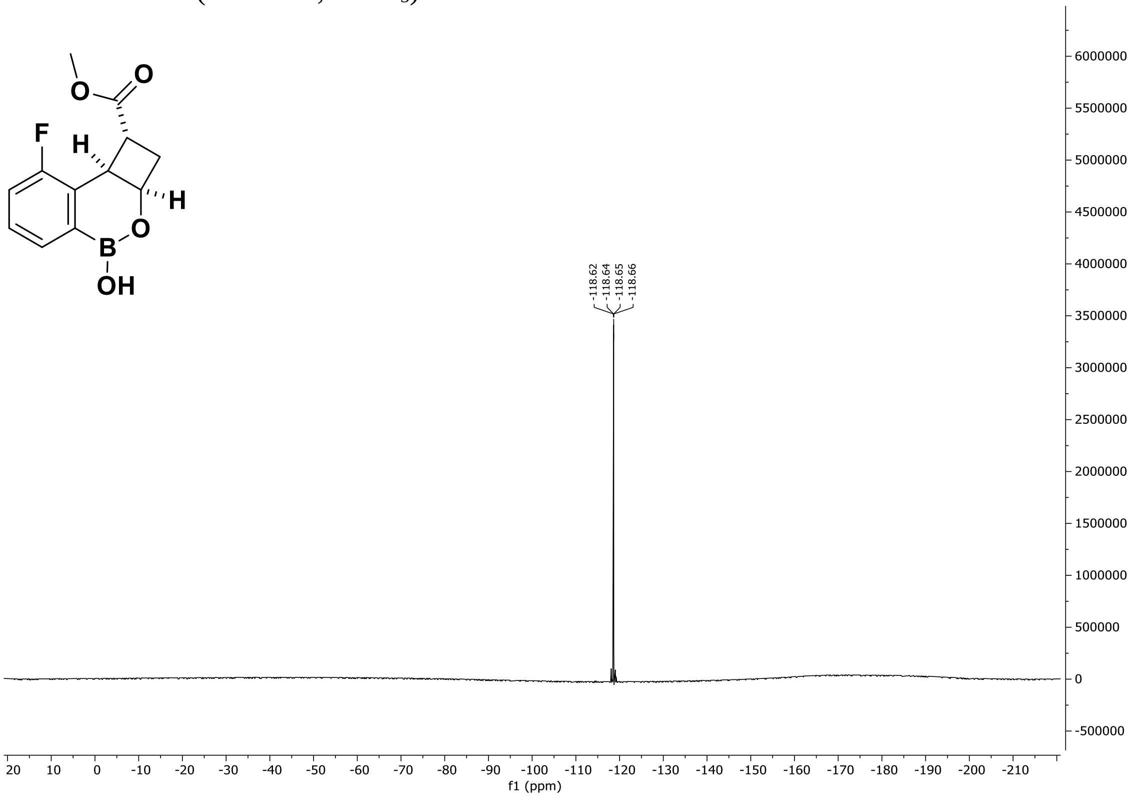

^{1}H-NMR of 28 (400 MHz, $CDCl_3$)

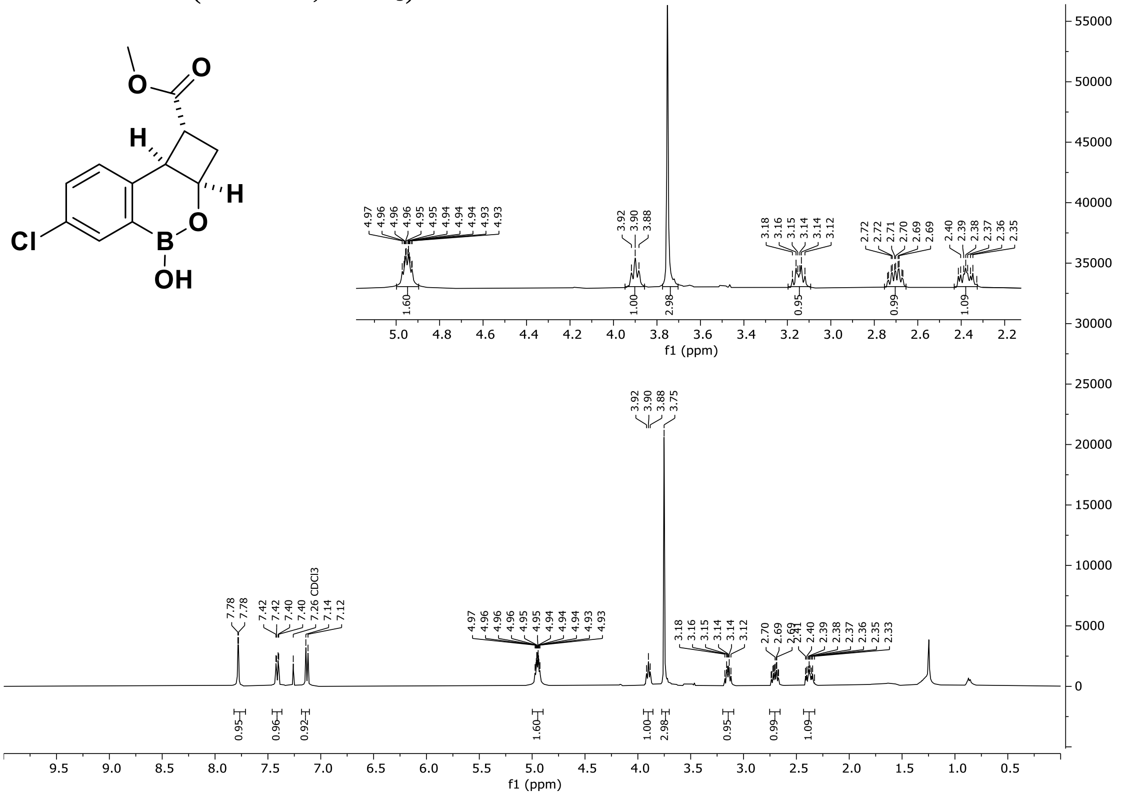

^{13}C-NMR of 28 (101 MHz, $CDCl_3$)

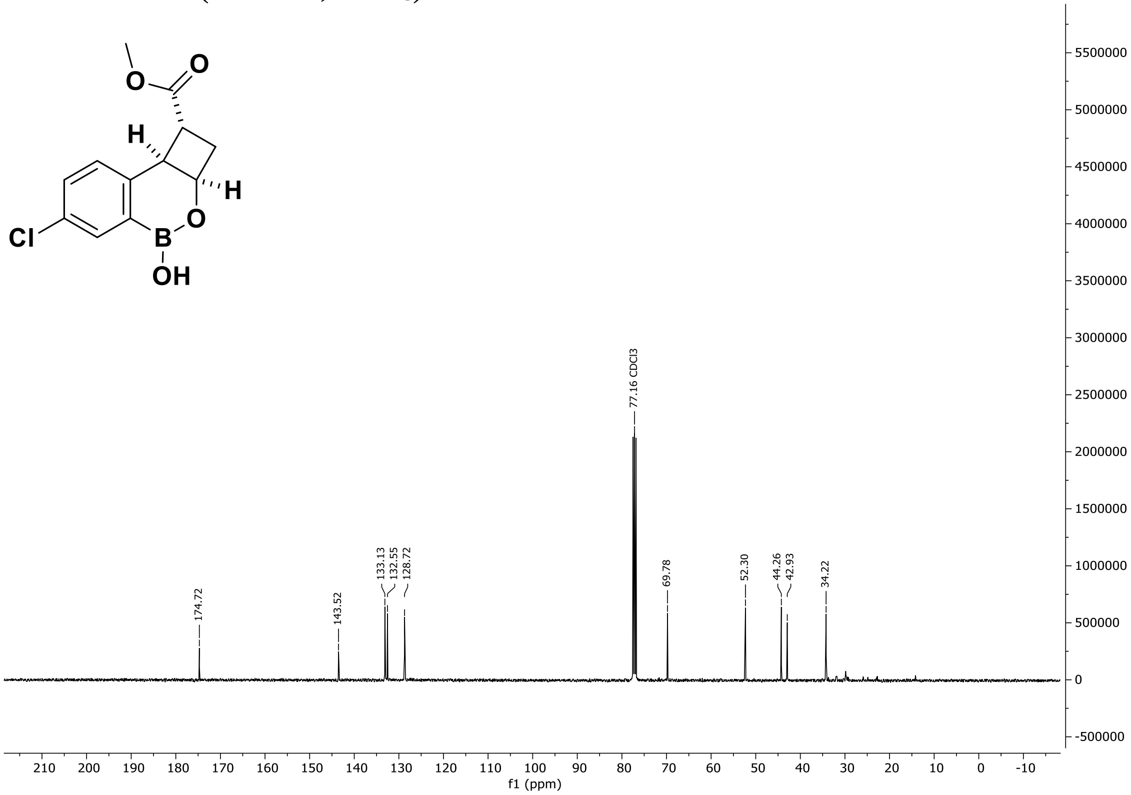

^{11}B-NMR of 28 (128 MHz, $CDCl_3$)

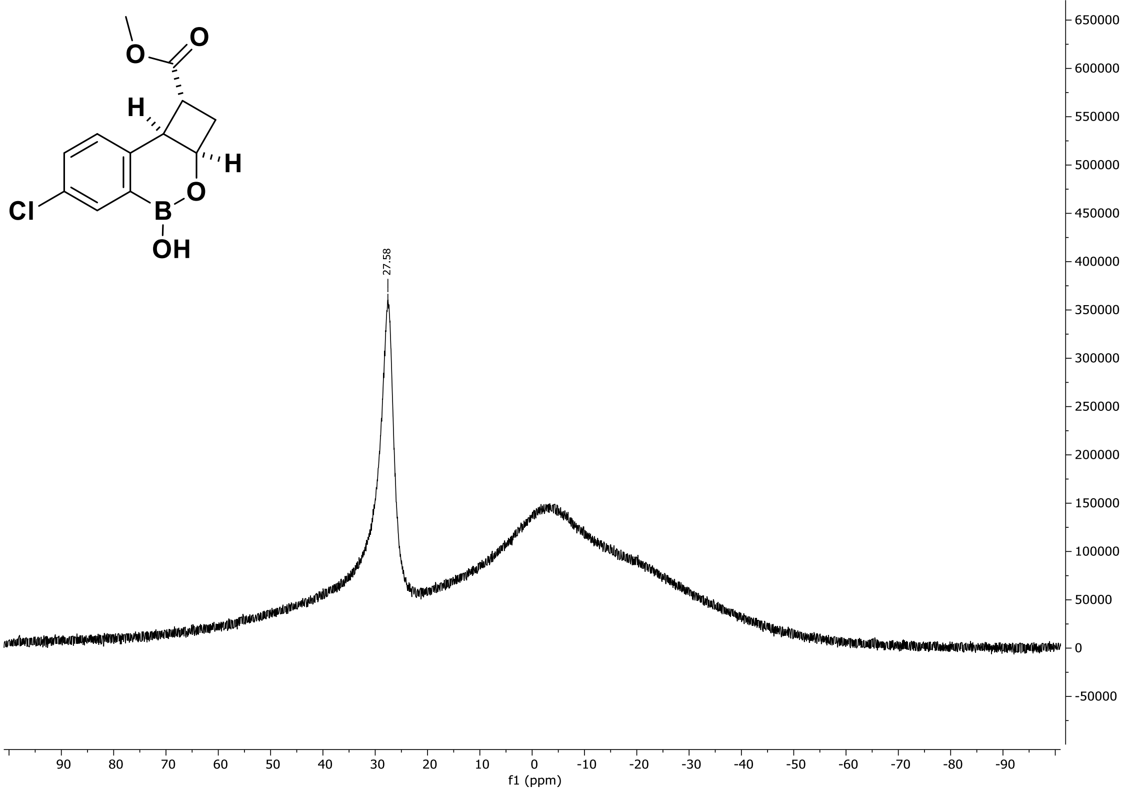

^{1}H-NMR of 29 (400 MHz, $CDCl_3$)

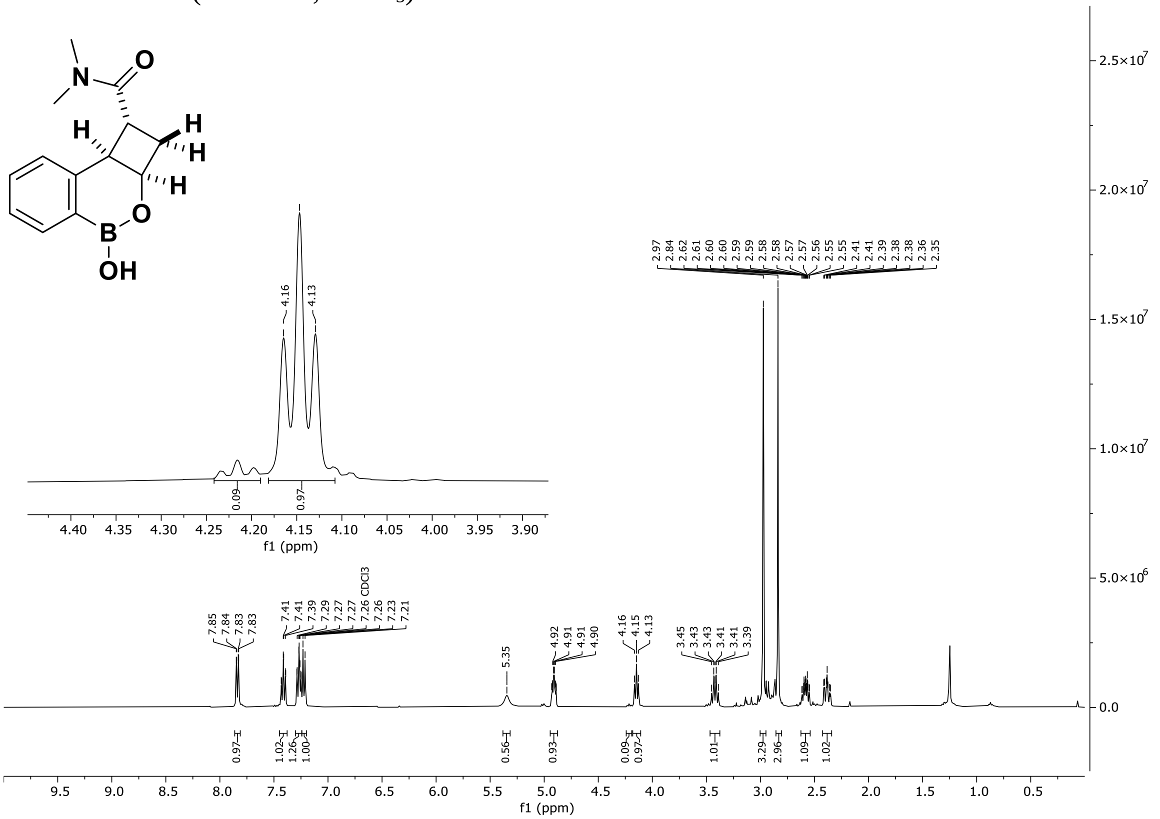

^{13}C-NMR of 29 (100 MHz, $CDCl_3$)

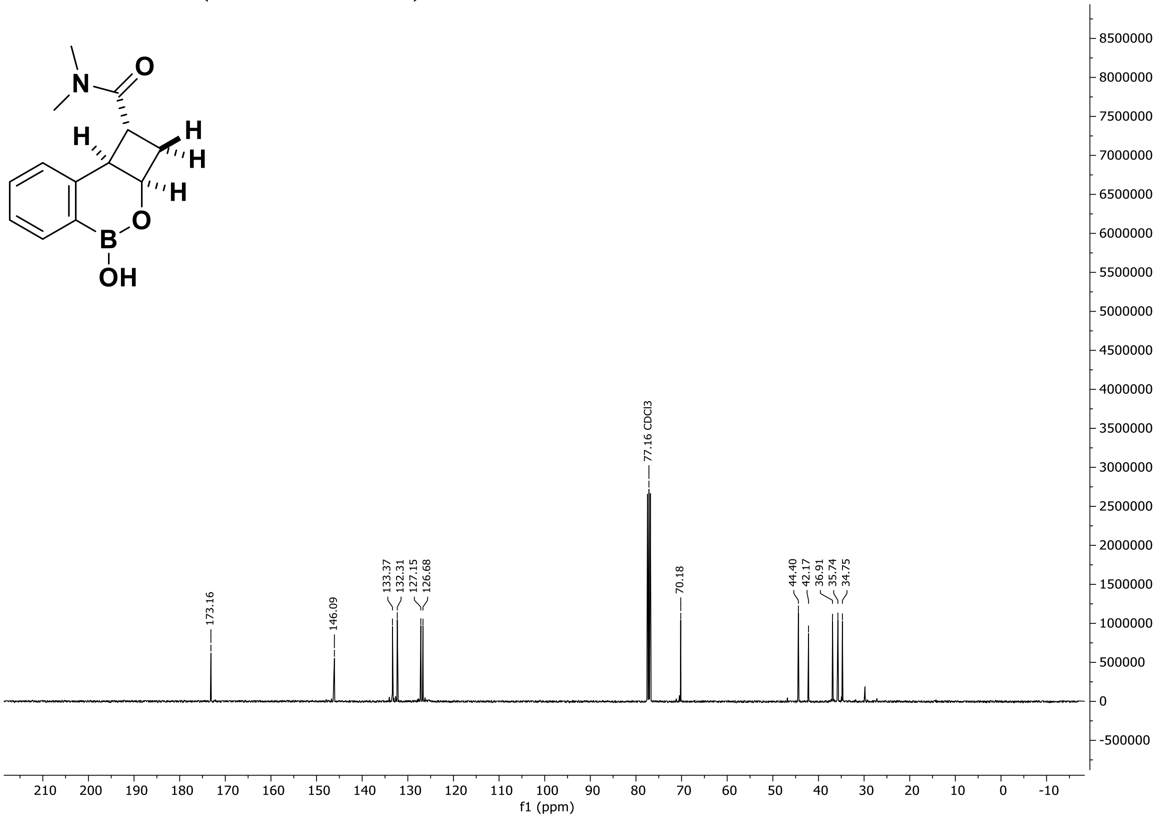

^{11}B-NMR of 29 (128 MHz CDCl$_3$)

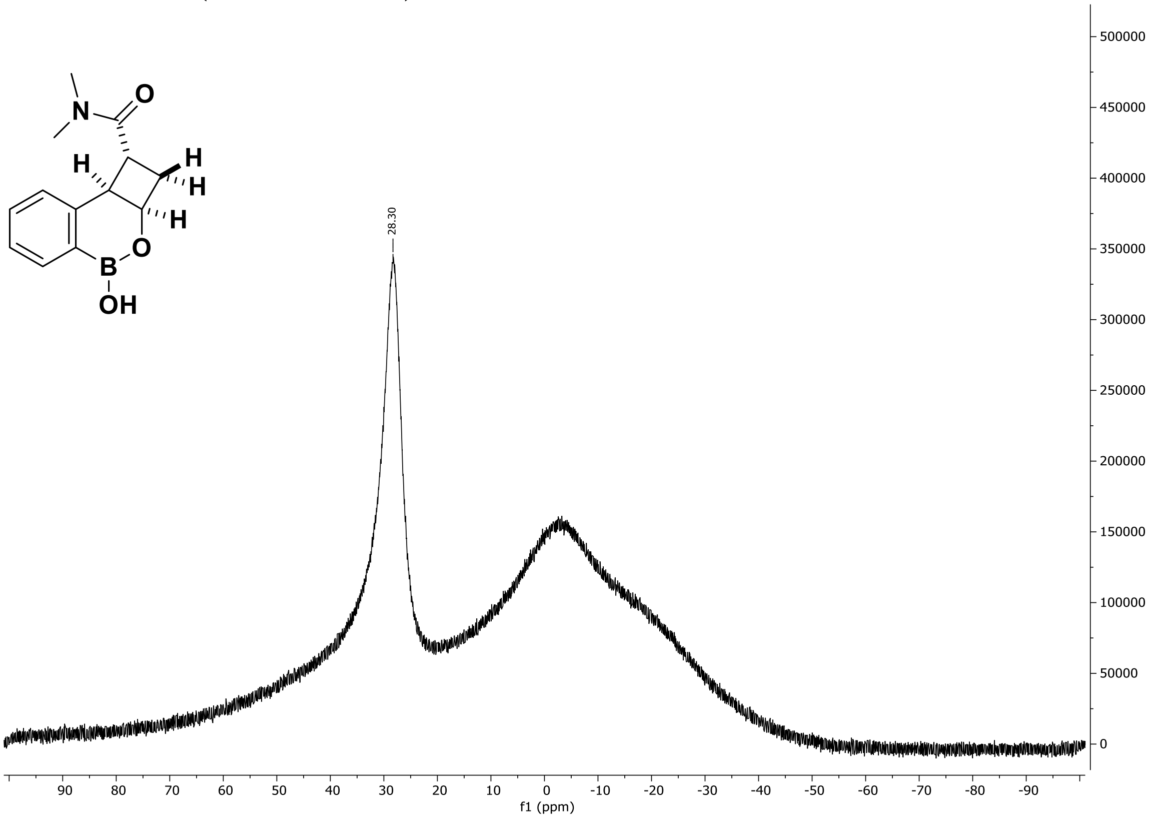

^{1}H-NMR of 30 (400 MHz, CDCl$_3$)

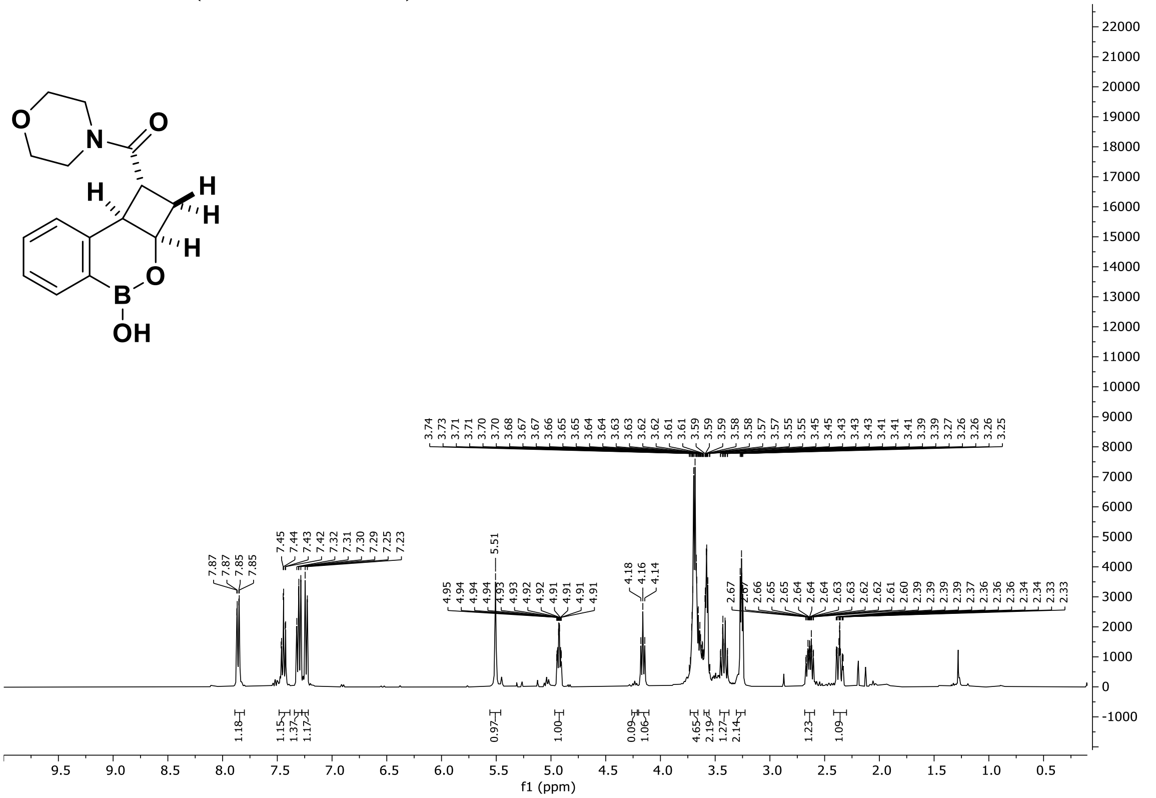

^{13}C-NMR of 30 (100 MHz, $CDCl_3$)

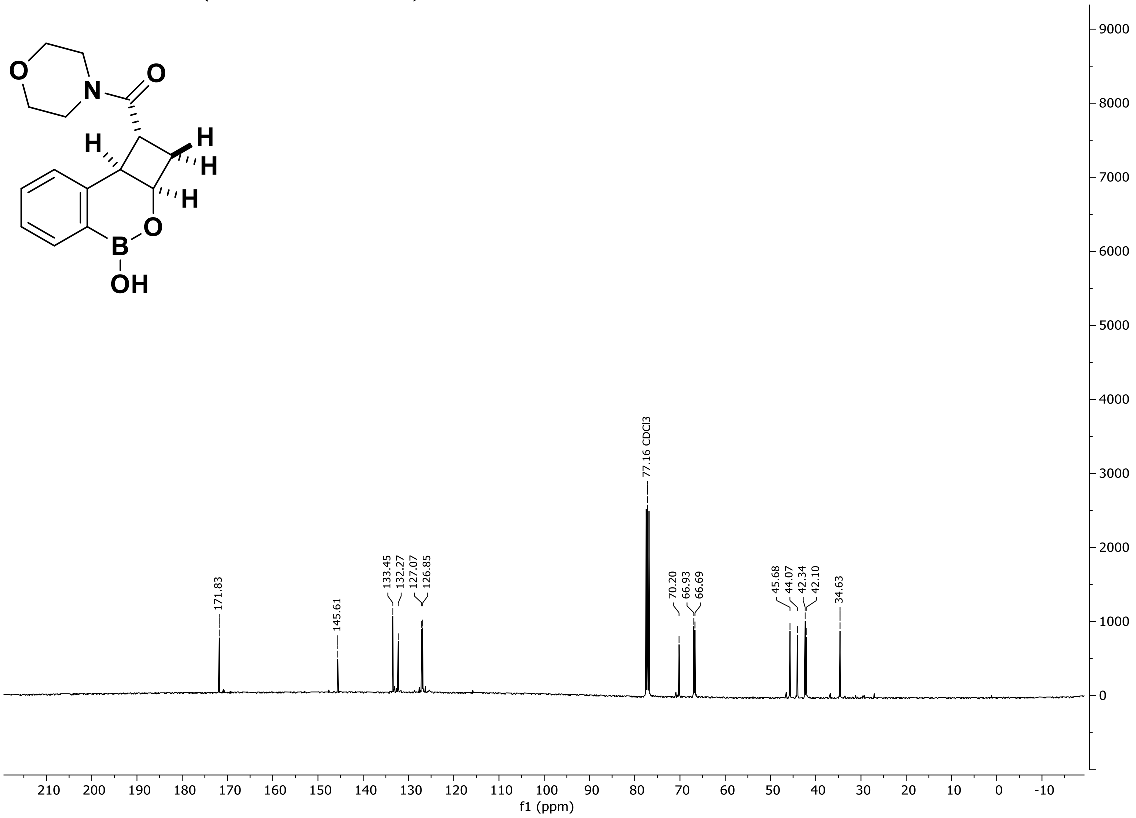

^{11}B-NMR of 30 (128 MHz $CDCl_3$)

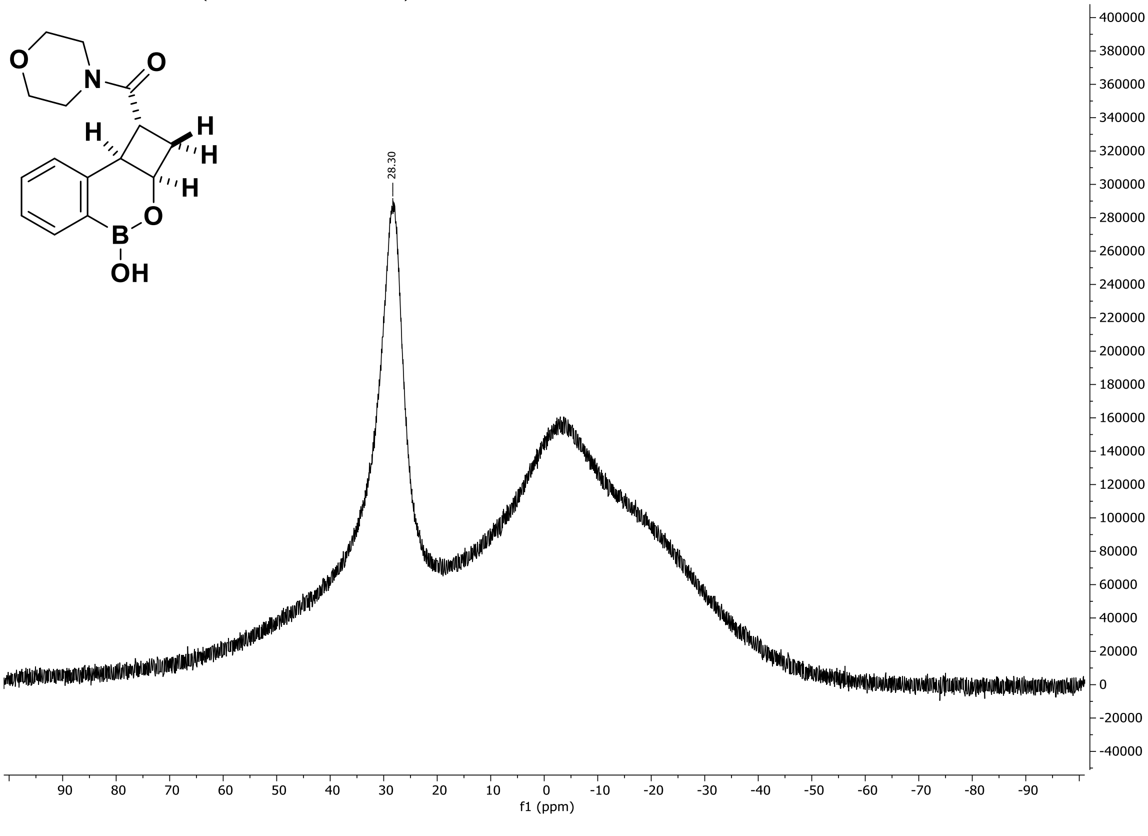

^{1}H-NMR of 31 (400 MHz, $CDCl_3$)

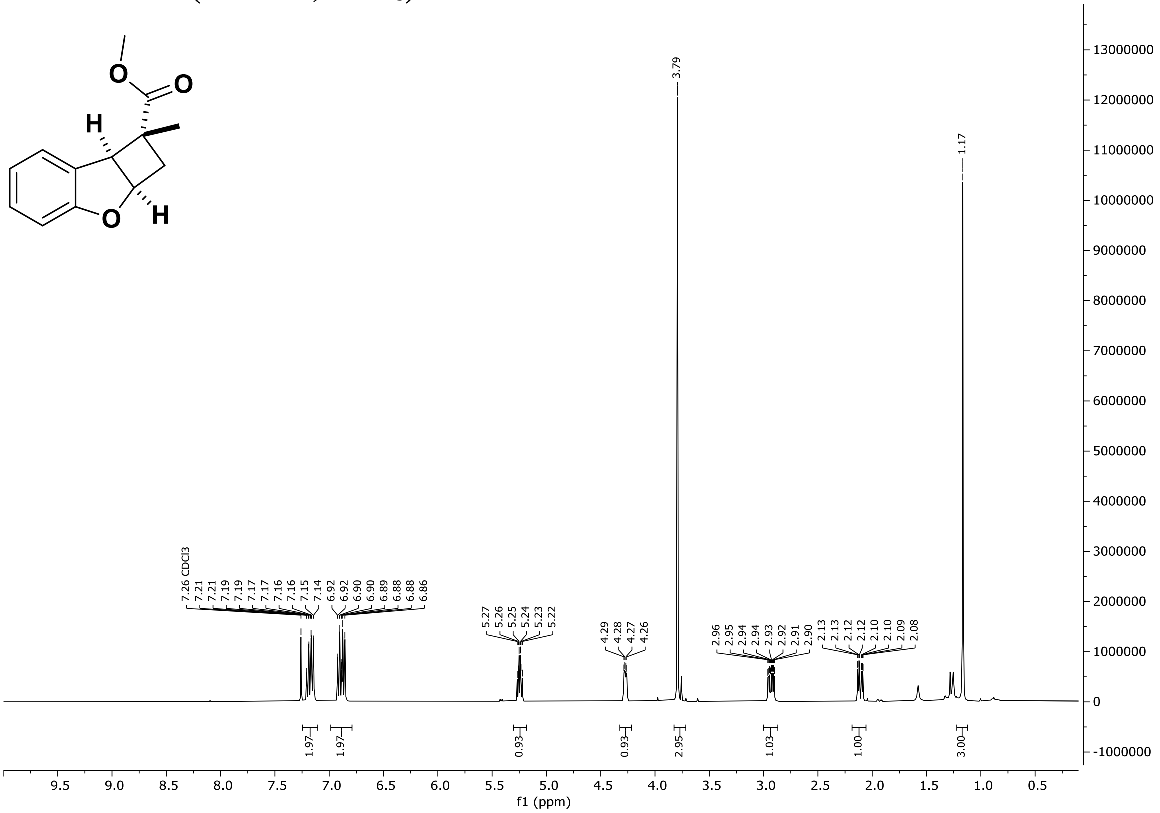

^{13}C-NMR of 31 (100 MHz, $CDCl_3$)

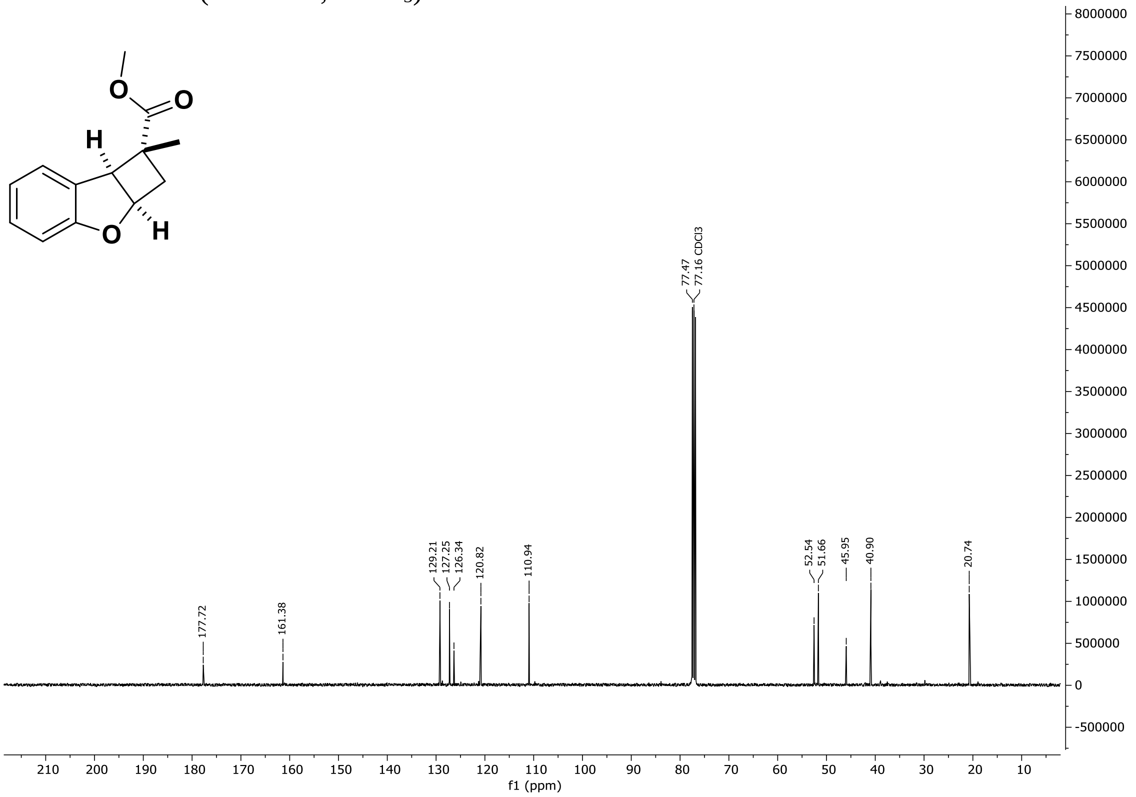

^{1}H-NMR of 32 (400 MHz, $CDCl_3$)

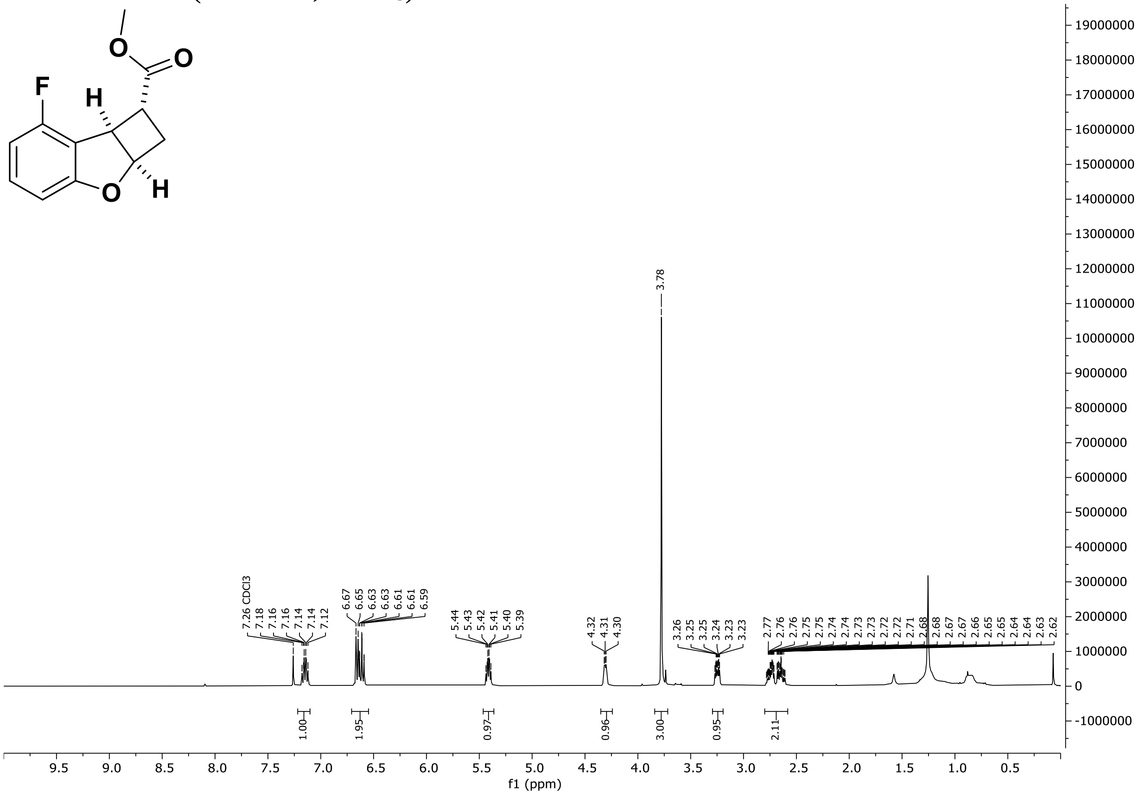

^{13}C-NMR of 32 (100 MHz, $CDCl_3$)

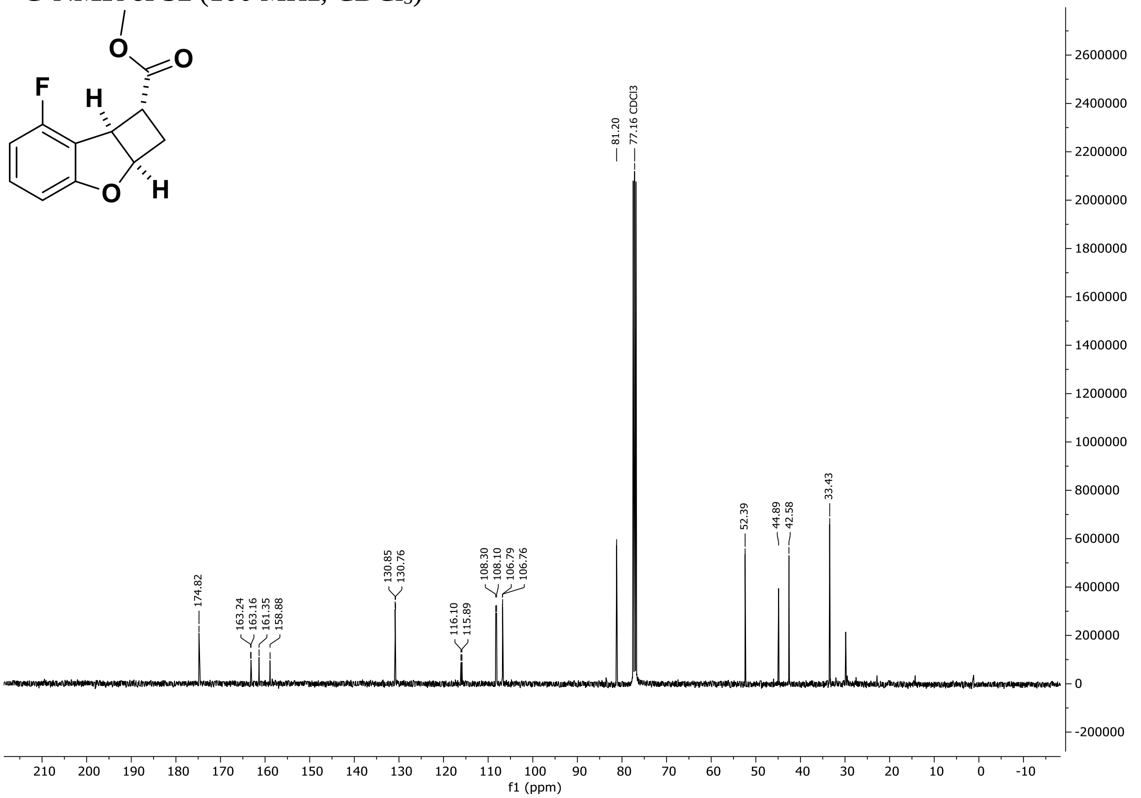

^{19}F-NMR of 32 (376 MHz, $CDCl_3$)

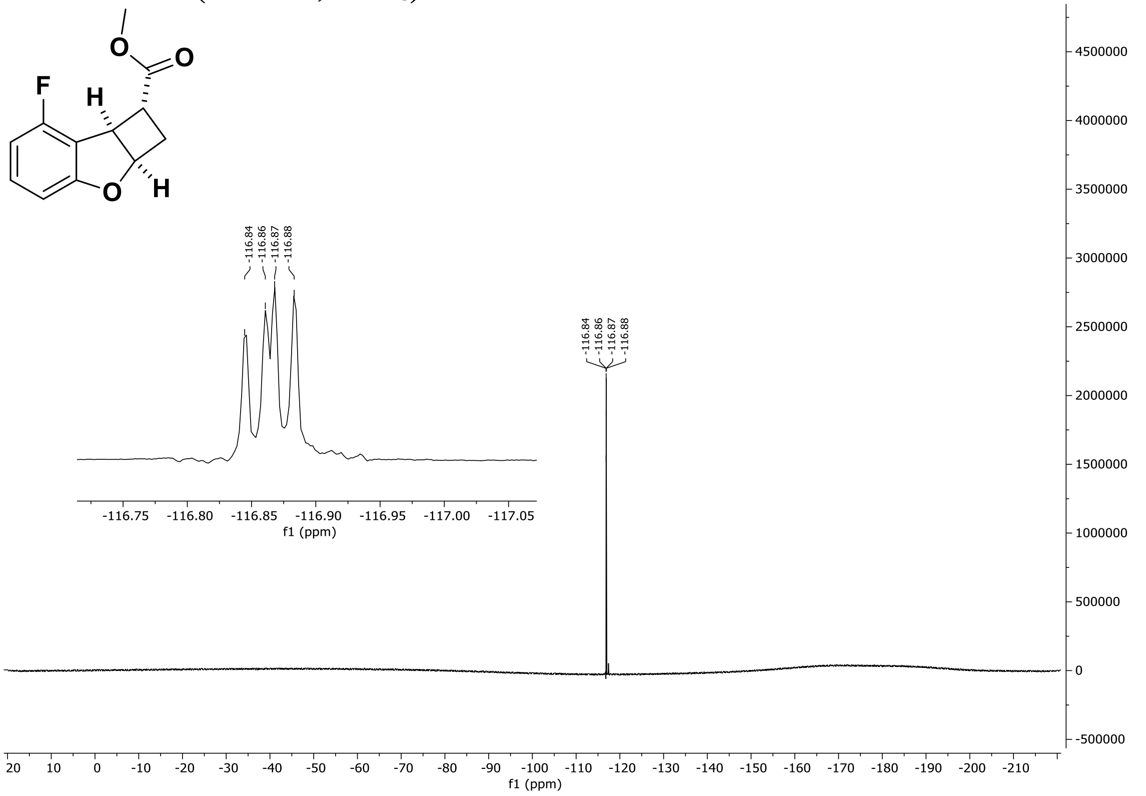

^{1}H-NMR of 33-*anti* (400 MHz, $CDCl_3$)

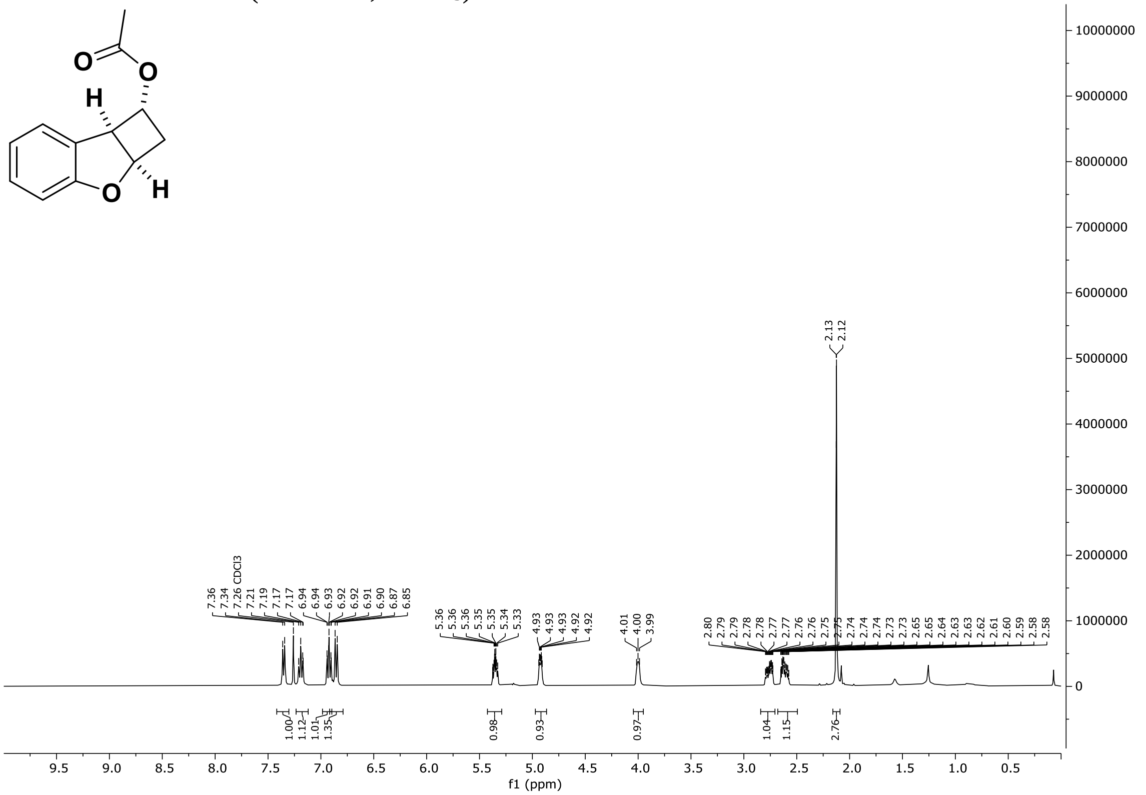

^{13}C-NMR of 33-*anti* (100 MHz, $CDCl_3$)

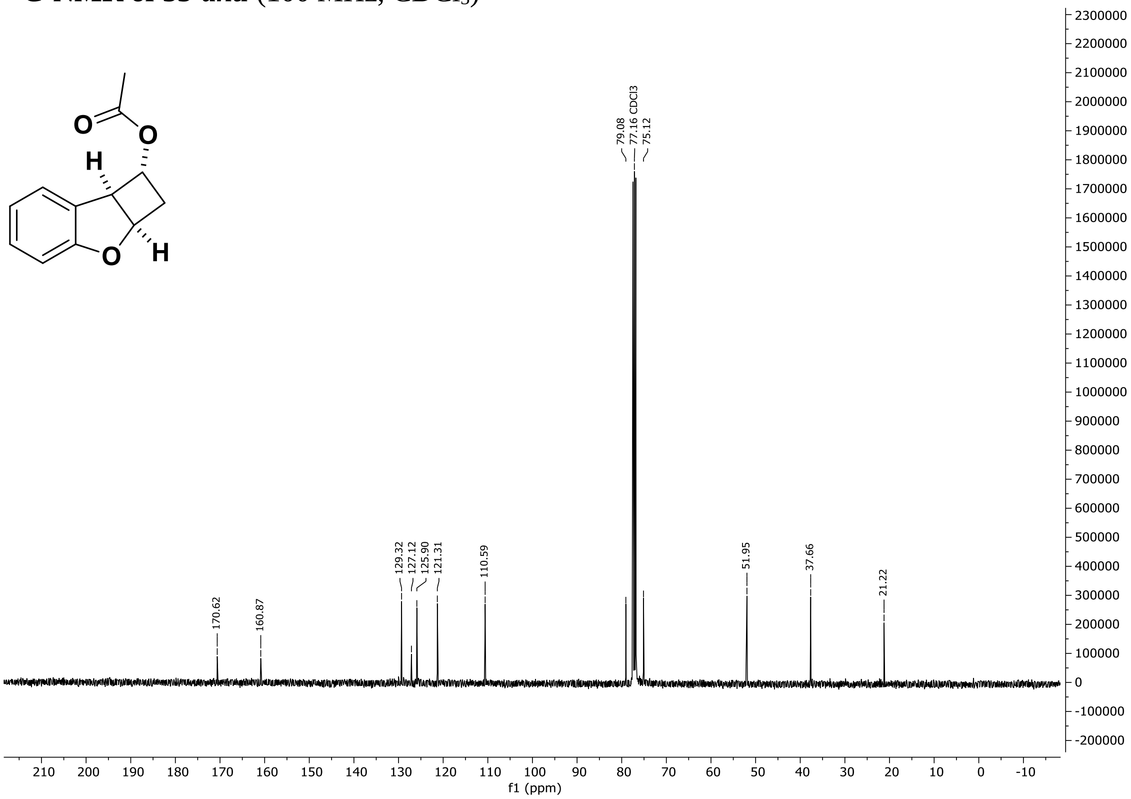

^{1}H-NMR of 33-*syn* (400 MHz, $CDCl_3$)

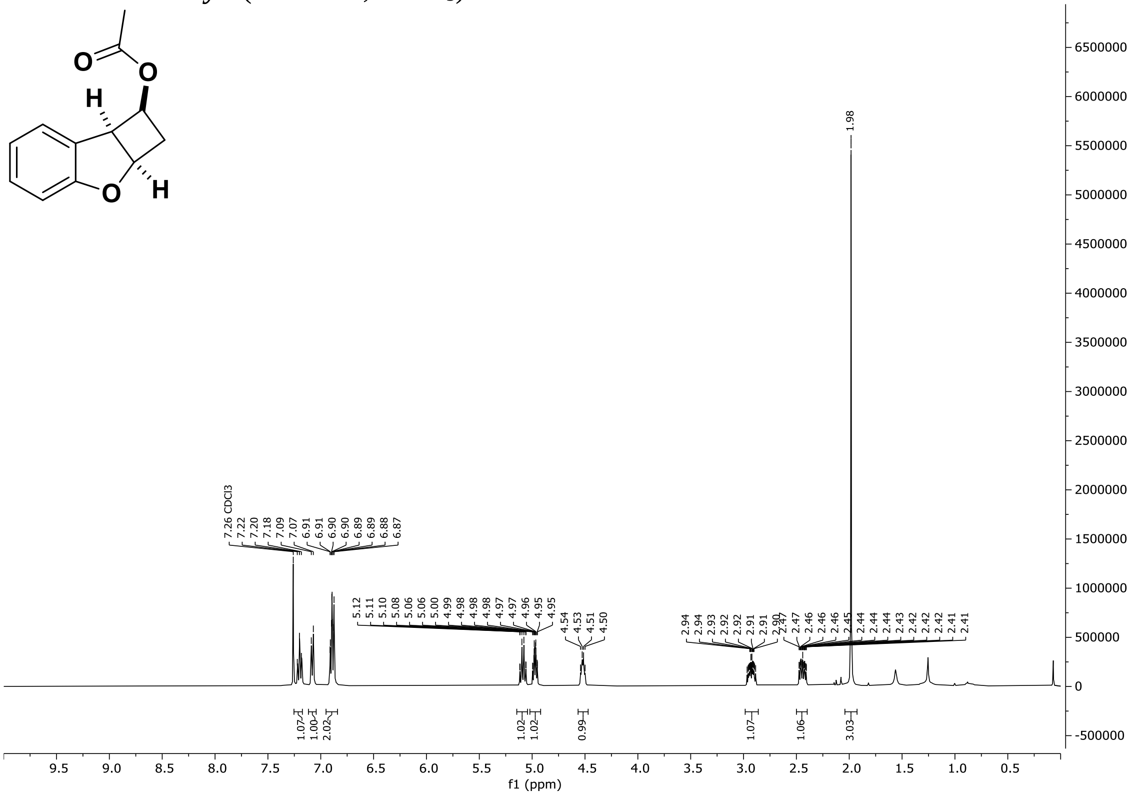

^{13}C-NMR of 33-*syn* (100 MHz, $CDCl_3$)

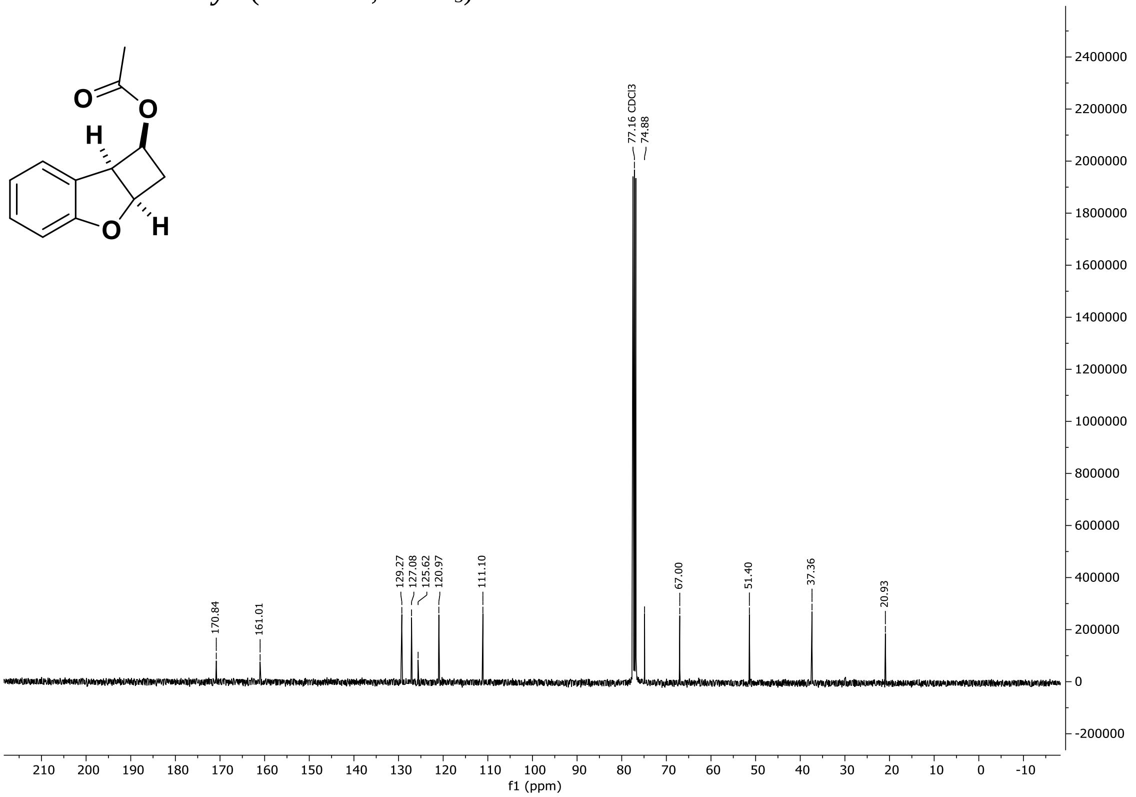

^{1}H-NMR of 34 (400 MHz, $CDCl_3$)

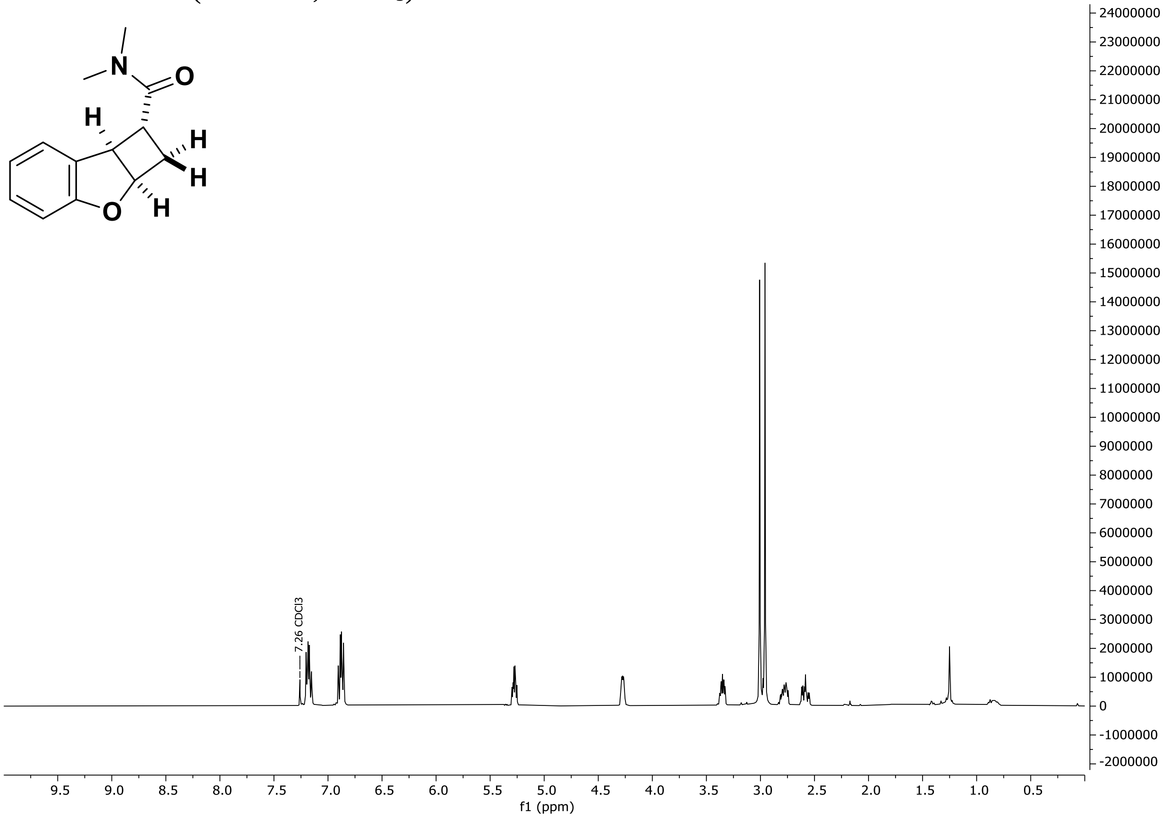

^{13}C-NMR of 34 (100 MHz, $CDCl_3$)

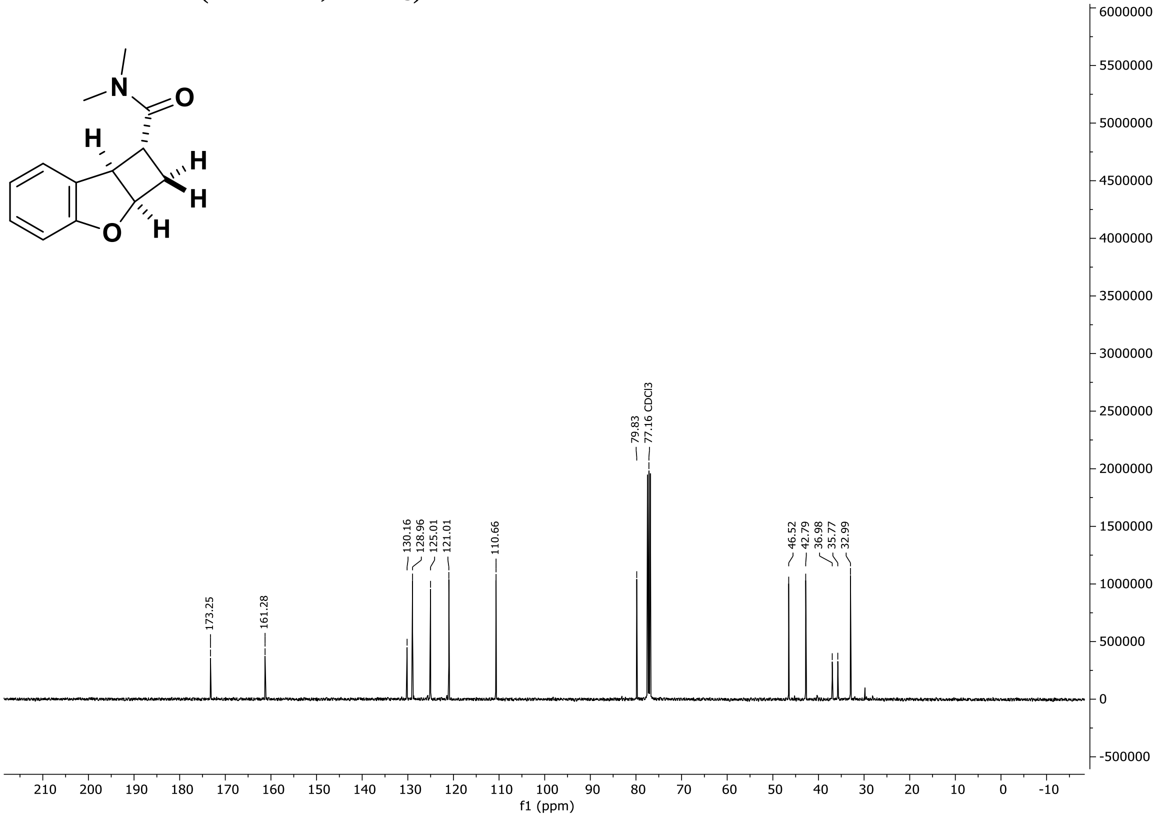

^{1}H-NMR of 35 (400 MHz, $CDCl_3$)

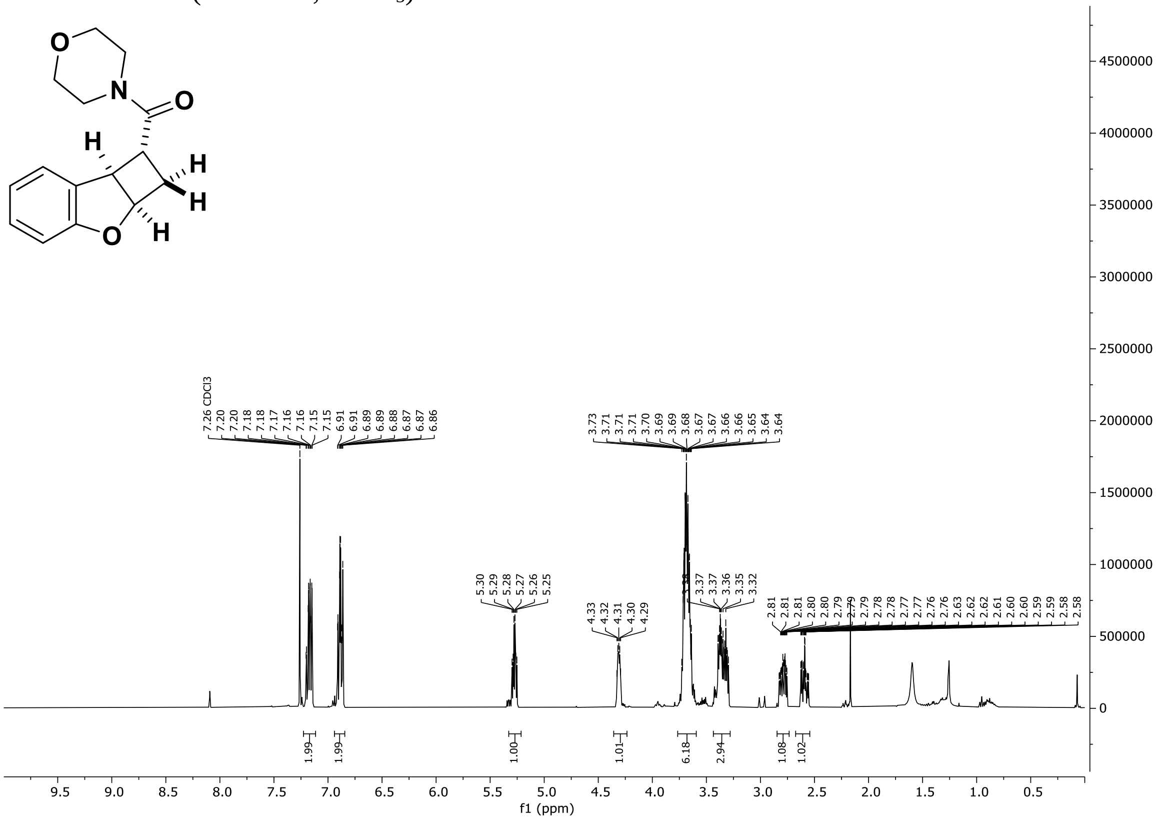

^{13}C-NMR of 35 (100 MHz, $CDCl_3$)

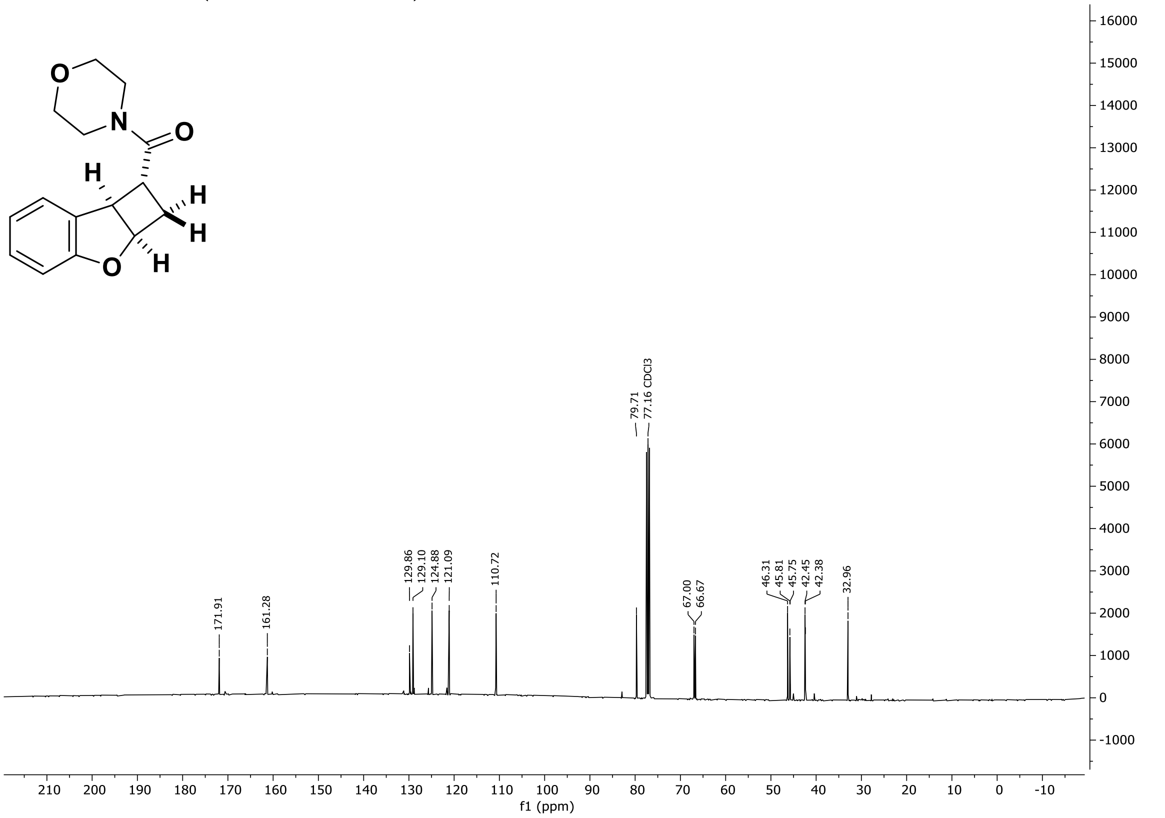

^{1}H-NMR of 36 (400 MHz, $CDCl_3$)

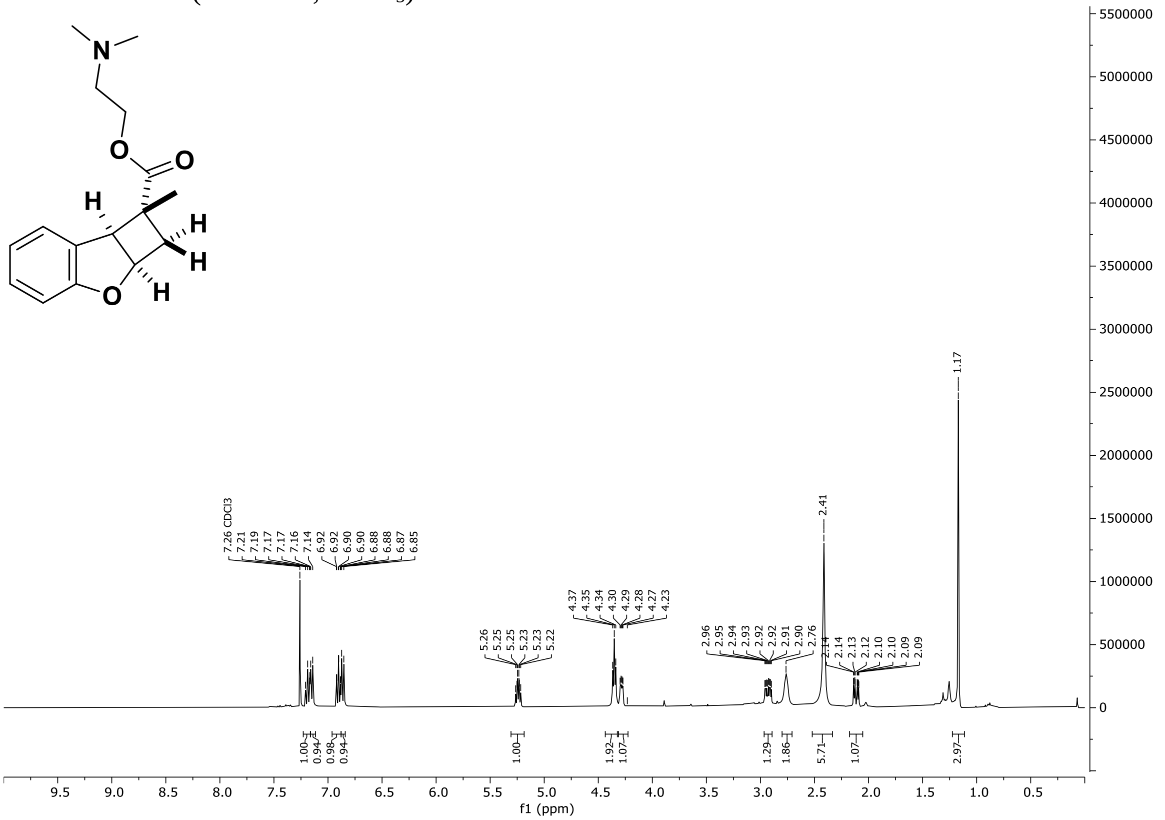

^{13}C-NMR of 36 (100 MHz, $CDCl_3$)

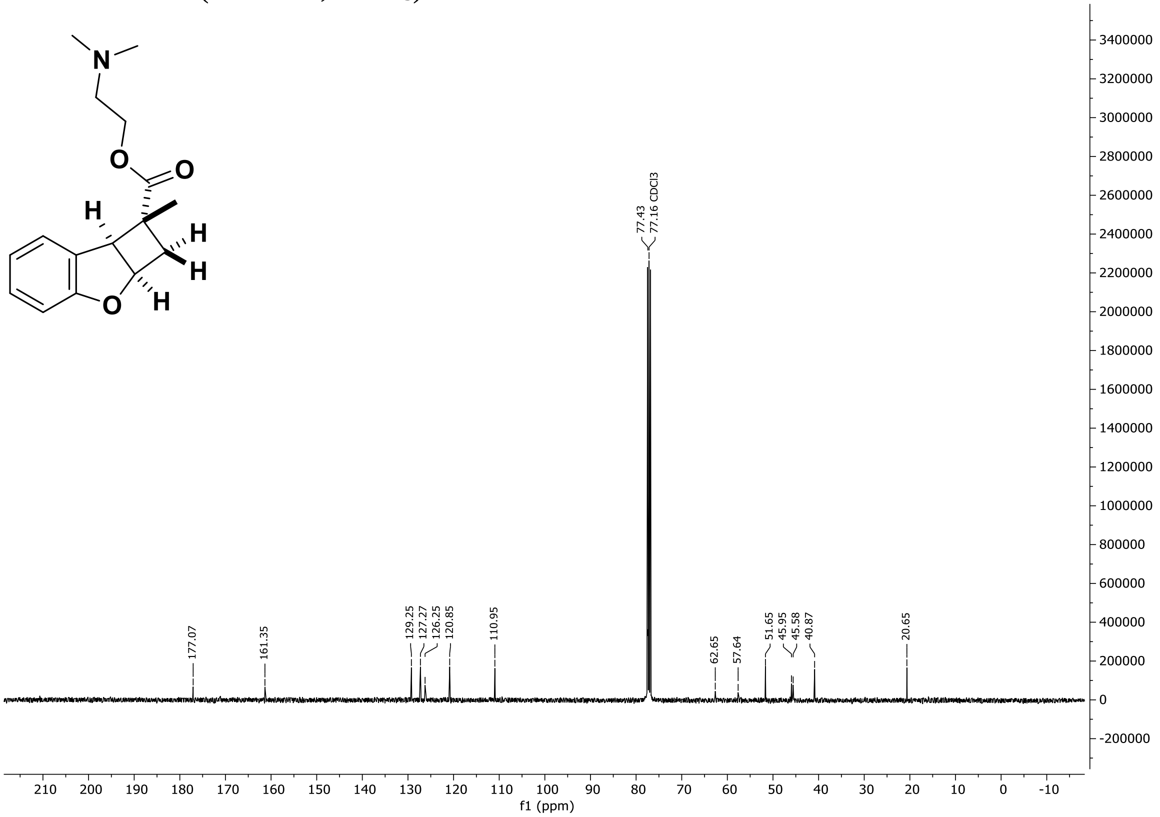

^{1}H-NMR of 37 (400 MHz, Acetone-d_6)

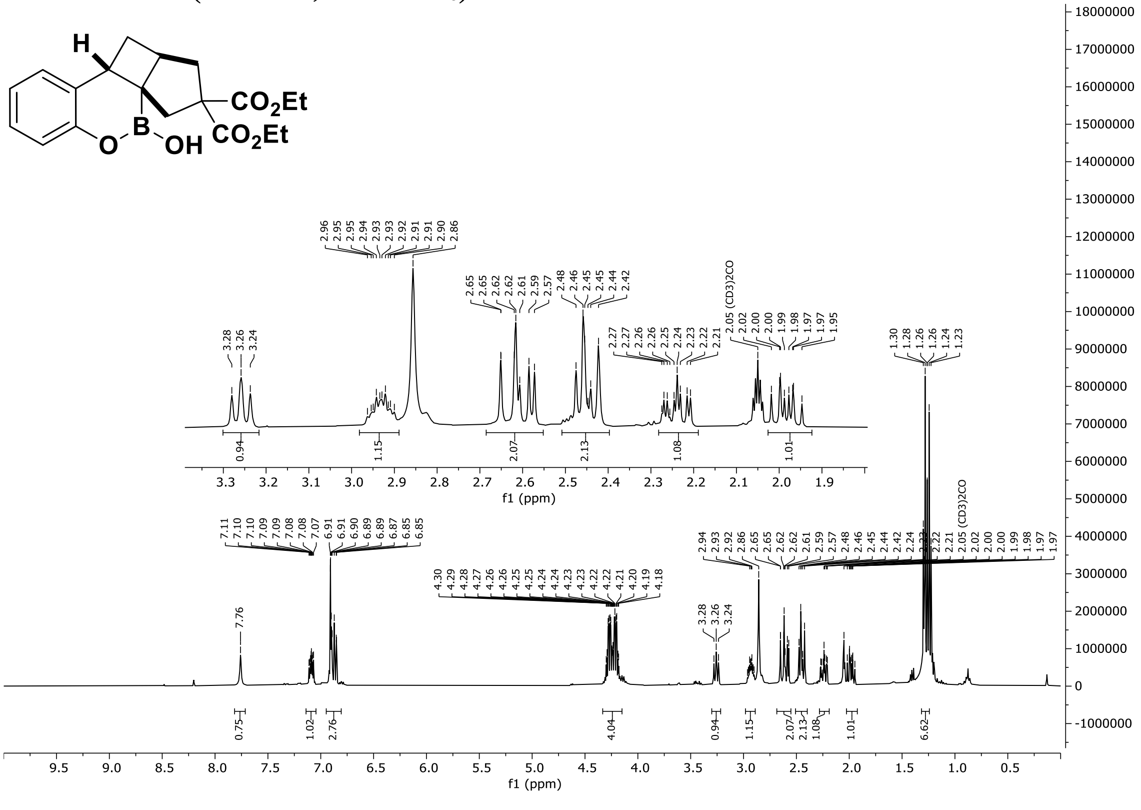

^{13}C-NMR of 37 (100 MHz, Acetone-d_6)

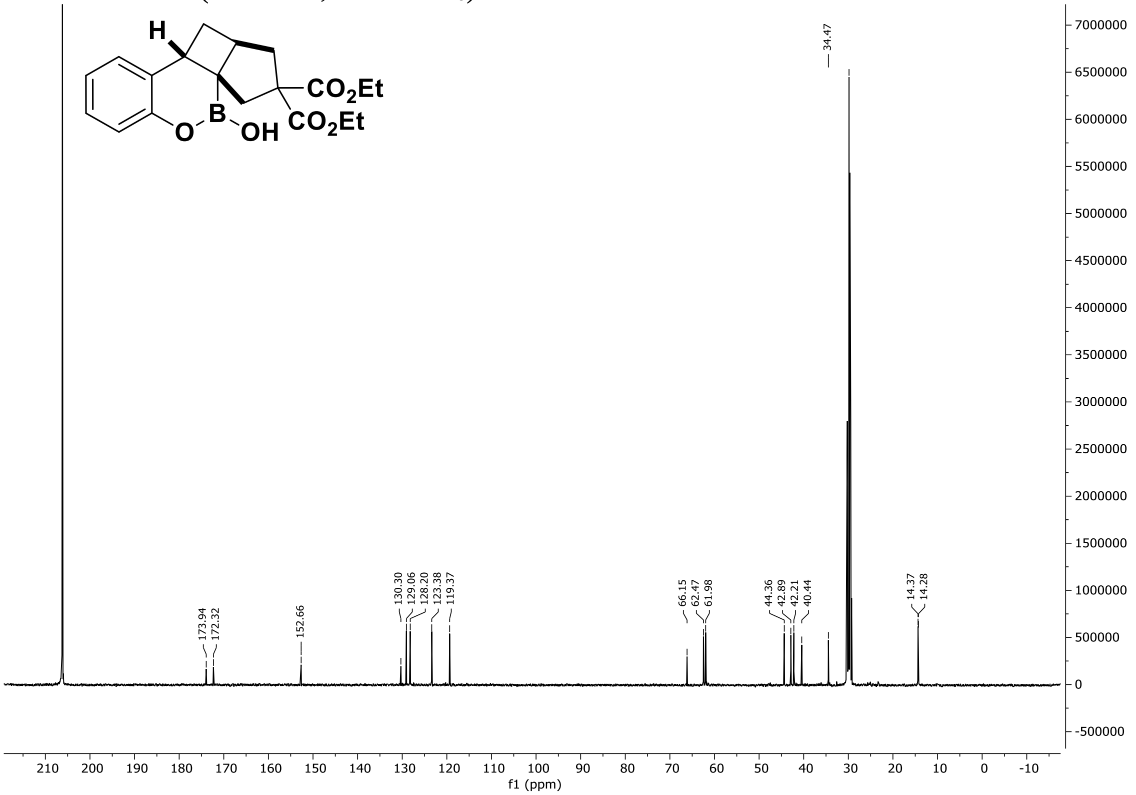

^{11}B-NMR of 37 (128 MHz, Acetone-d_6)

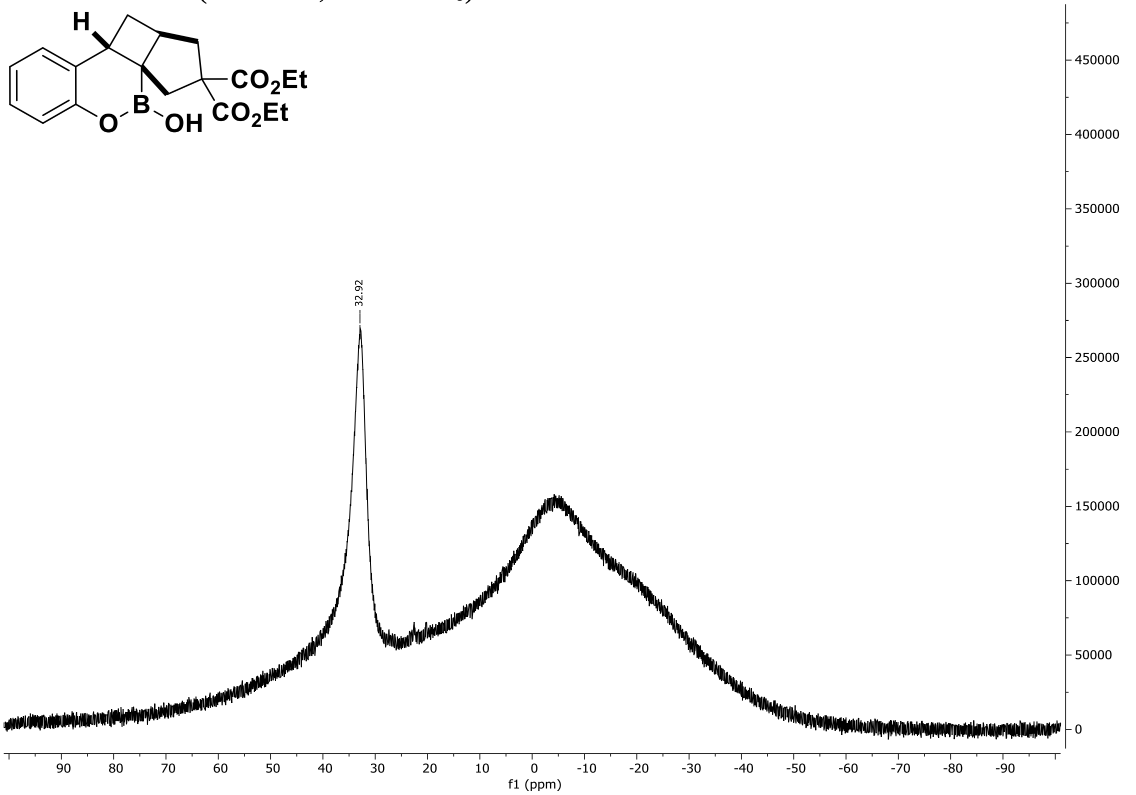

^{1}H-NMR of 38 (400 MHz, $CDCl_3$)

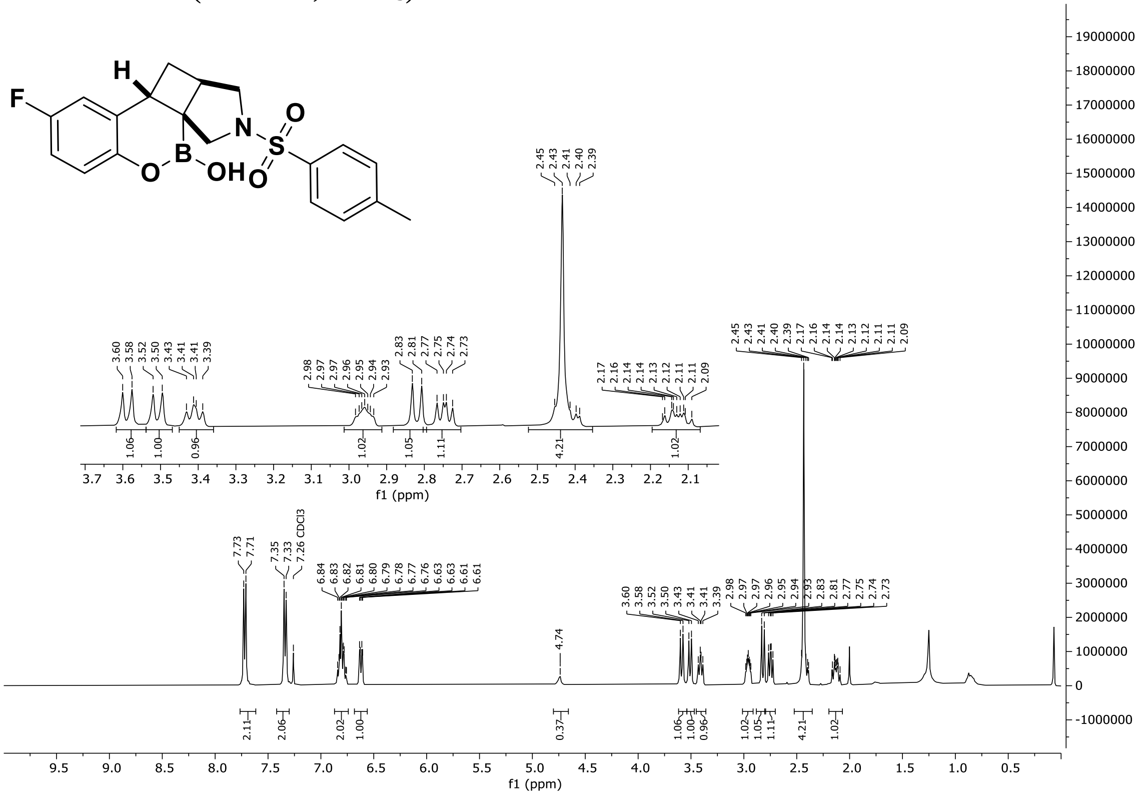

^{13}C-NMR of 38 (100 MHz, $CDCl_3$)

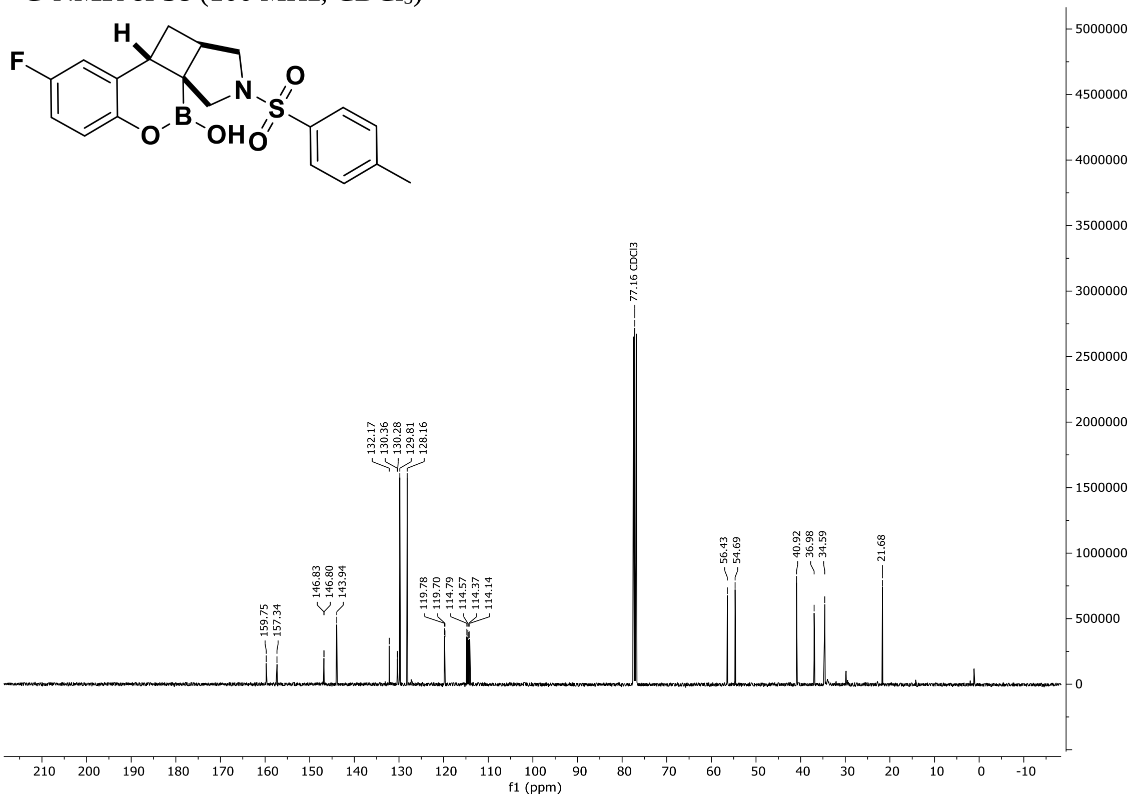

^{11}B-NMR of 38 (128 MHz $CDCl_3$)

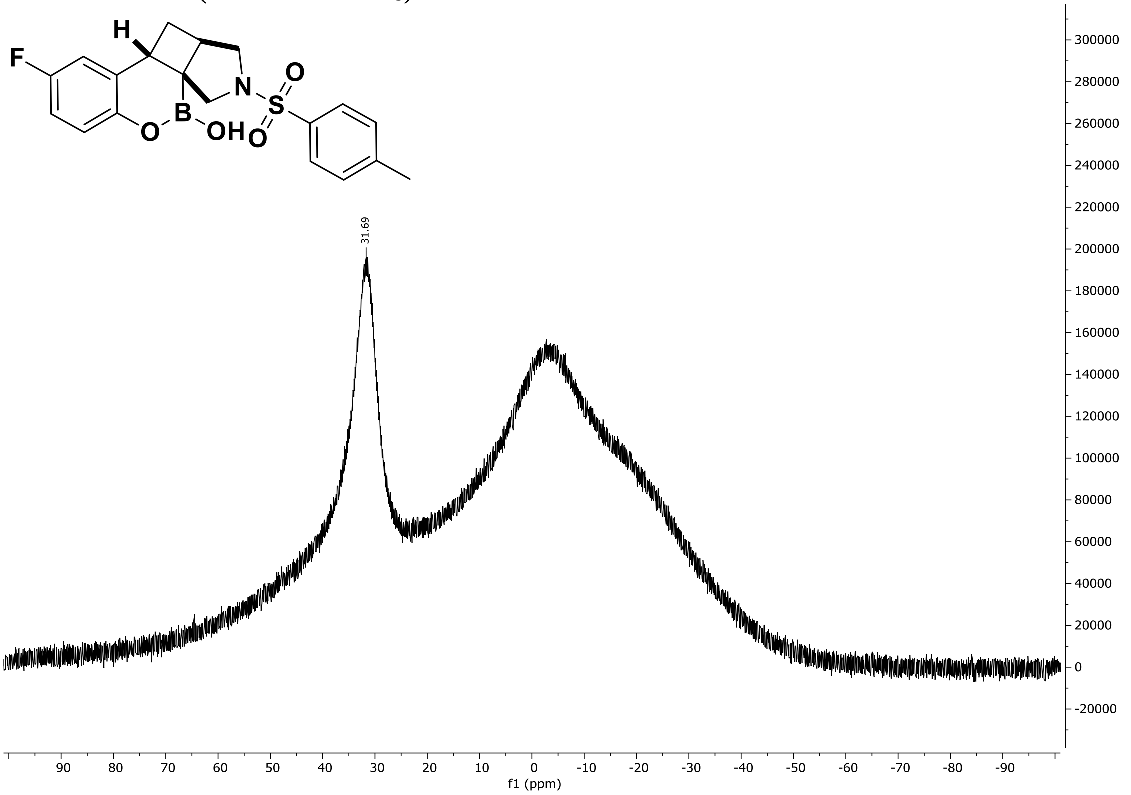

^{19}F-NMR of 38 (376 MHz, $CDCl_3$)

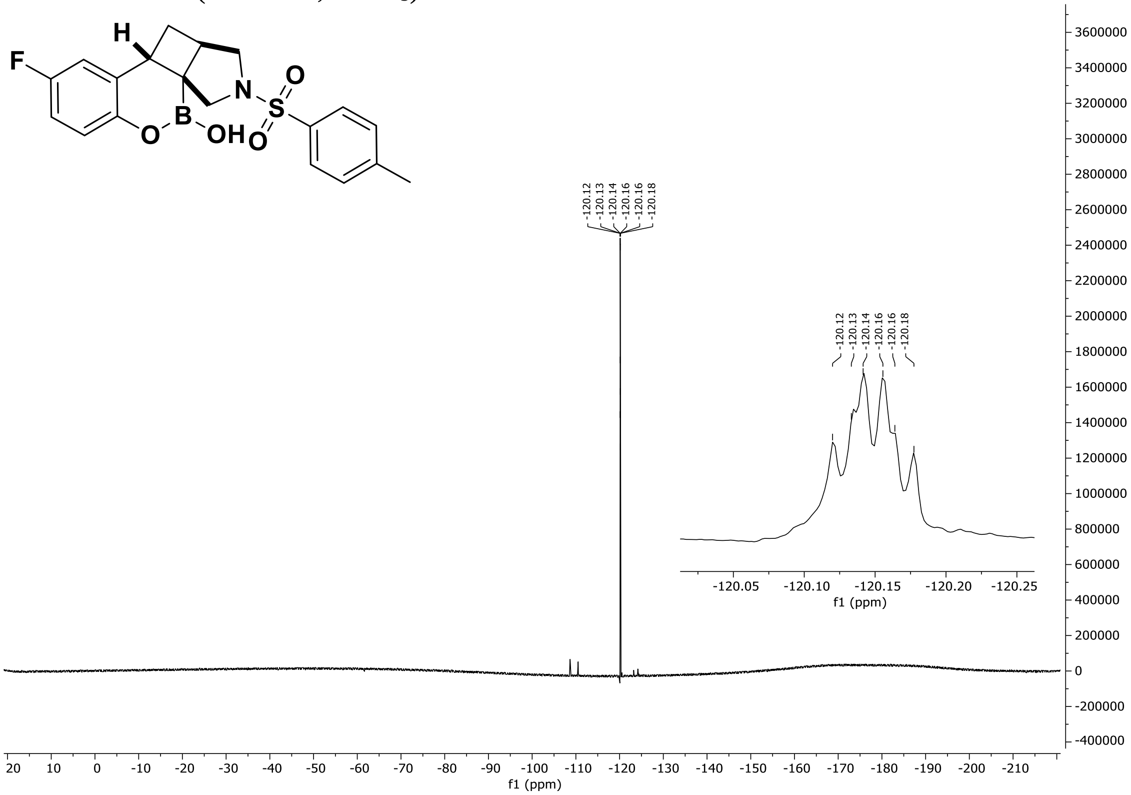

^{1}H-NMR of 39 (400 MHz, Acetone-d_6)

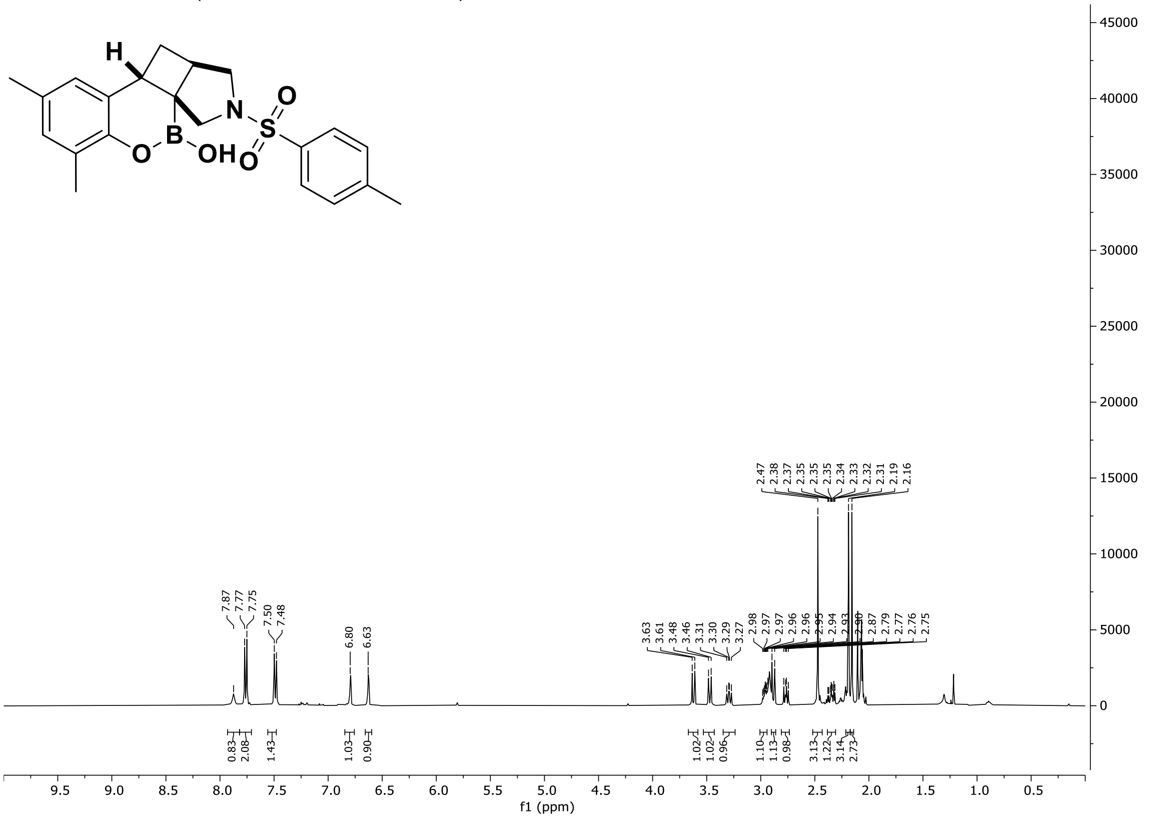

^{13}C-NMR of 39 (100 MHz, Acetone-d_6)

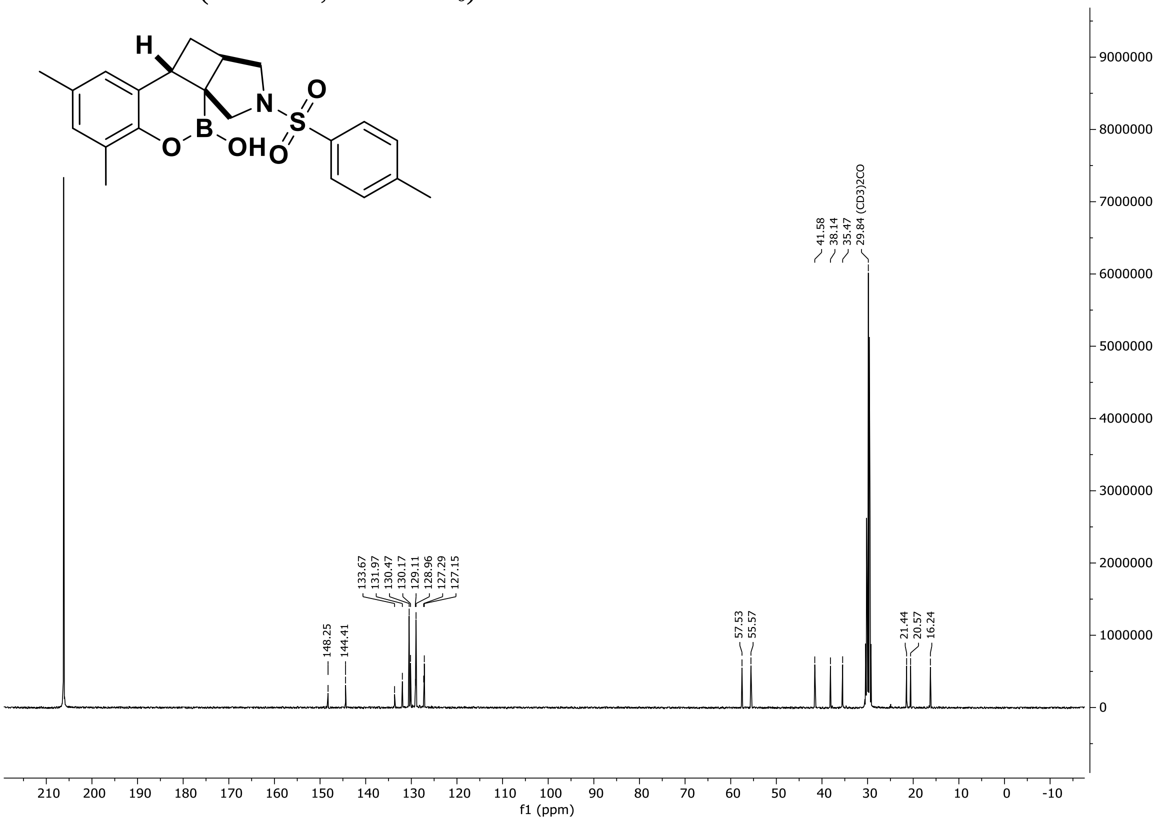

^{11}B-NMR of 39 (128 MHz, Acetone-d_6)

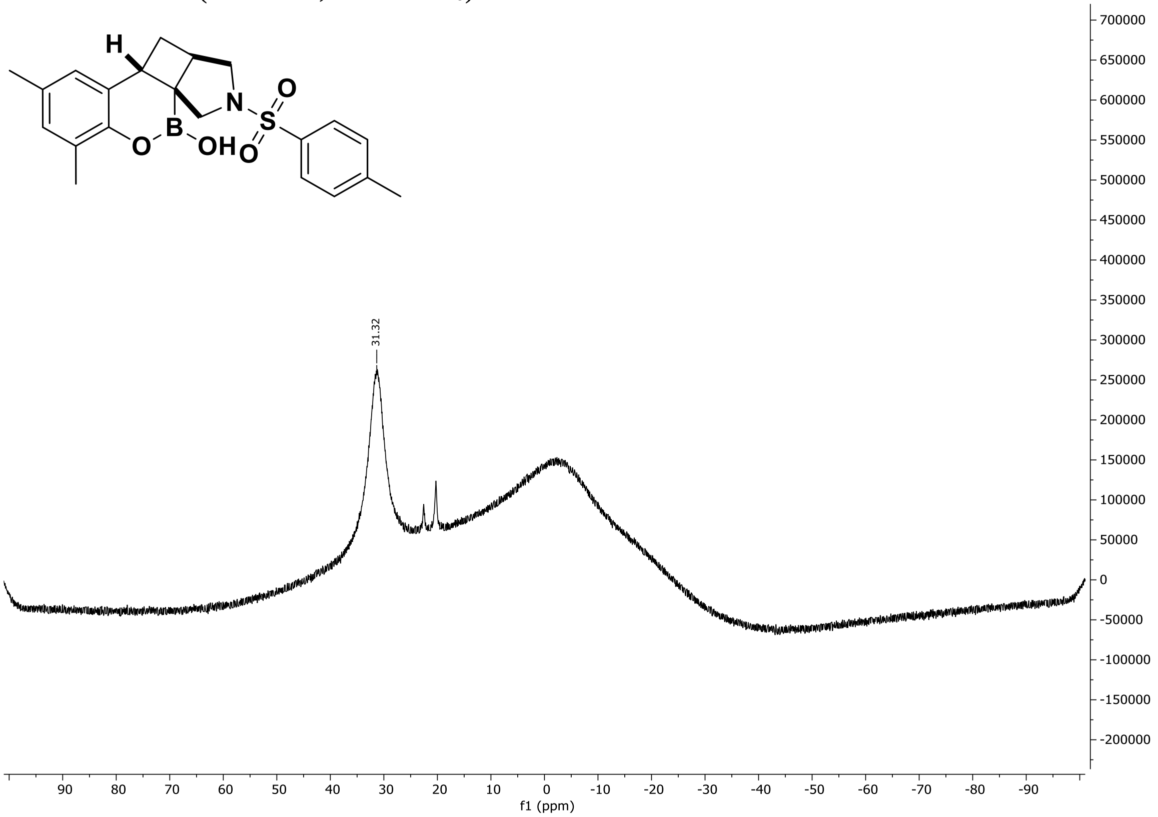

^{1}H-NMR of 40 (400 MHz, Acetone-d_6)

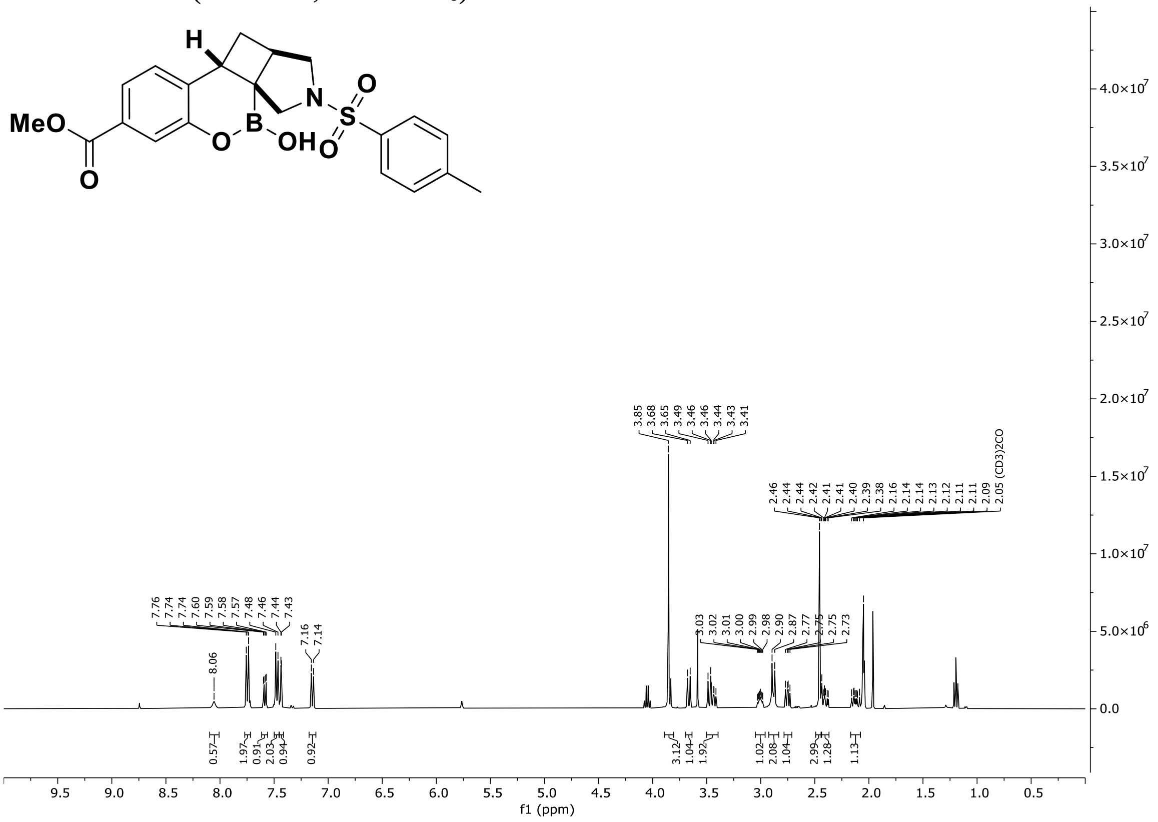

^{13}C-NMR of 40 (100 MHz, Acetone-d_6)

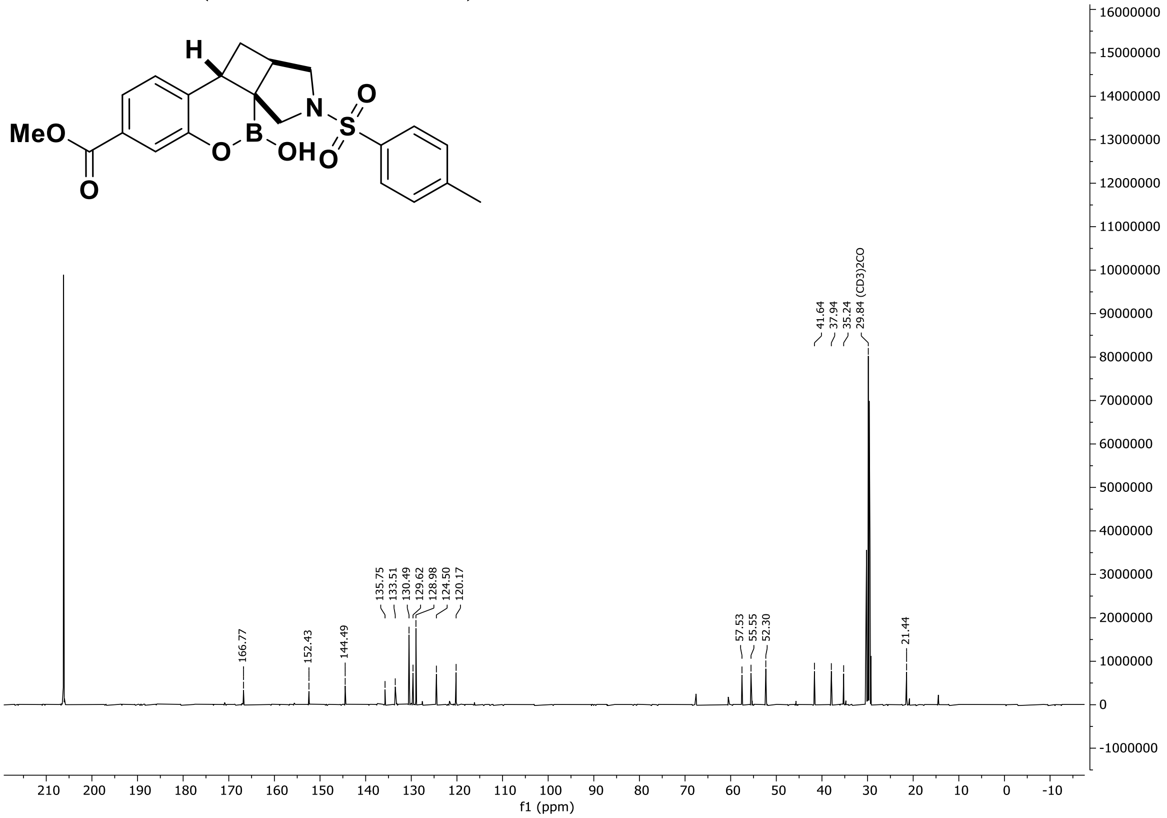

^{11}B-NMR of 40 (128 MHz, Acetone-d_6), with boric acid, marked with a blue dot

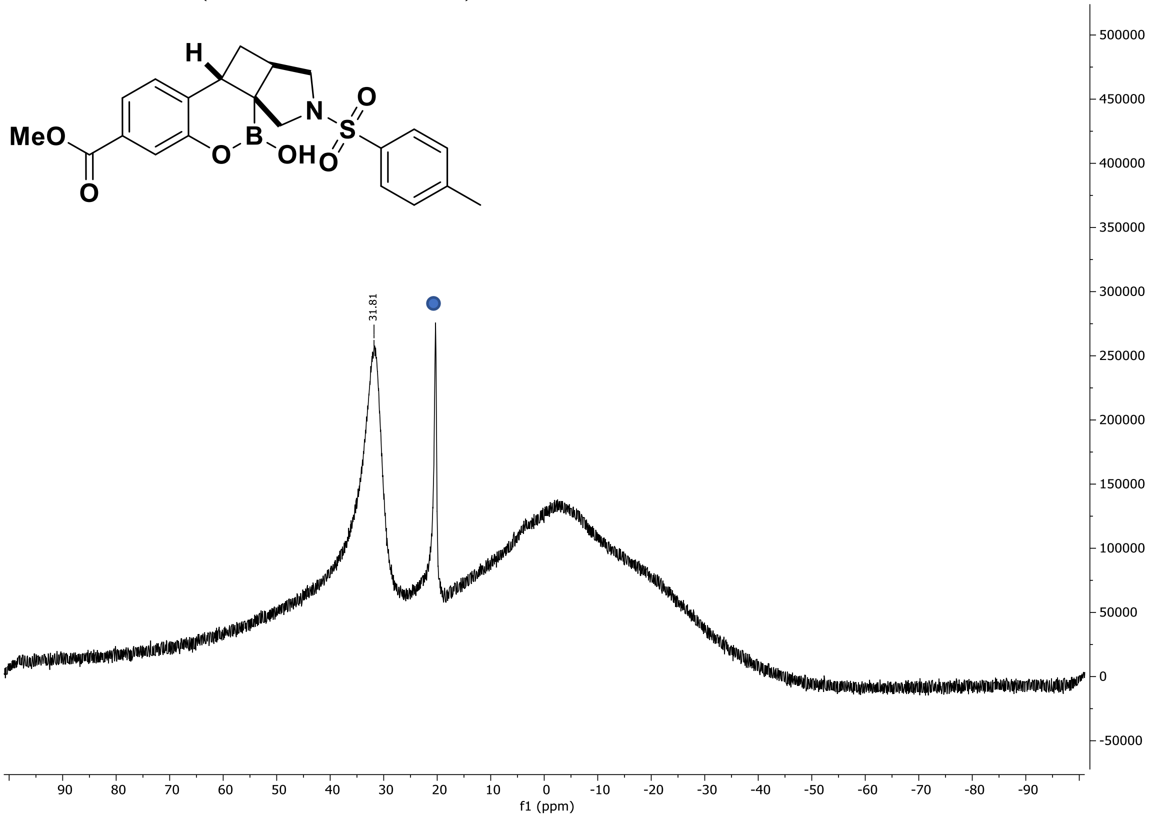

^{1}H-NMR of 41 (400 MHz, $CDCl_3$)

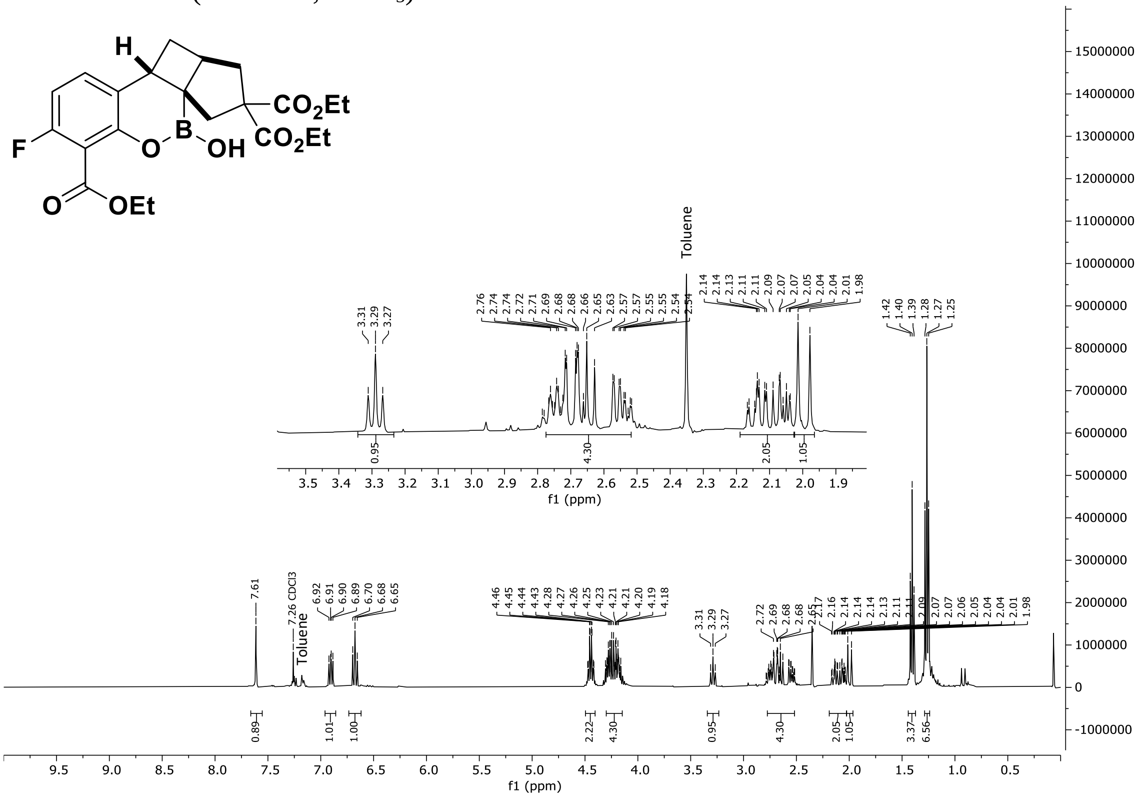

^{13}C-NMR of 41 (101 MHz, $CDCl_3$), with toluene impurity marked with blue dots

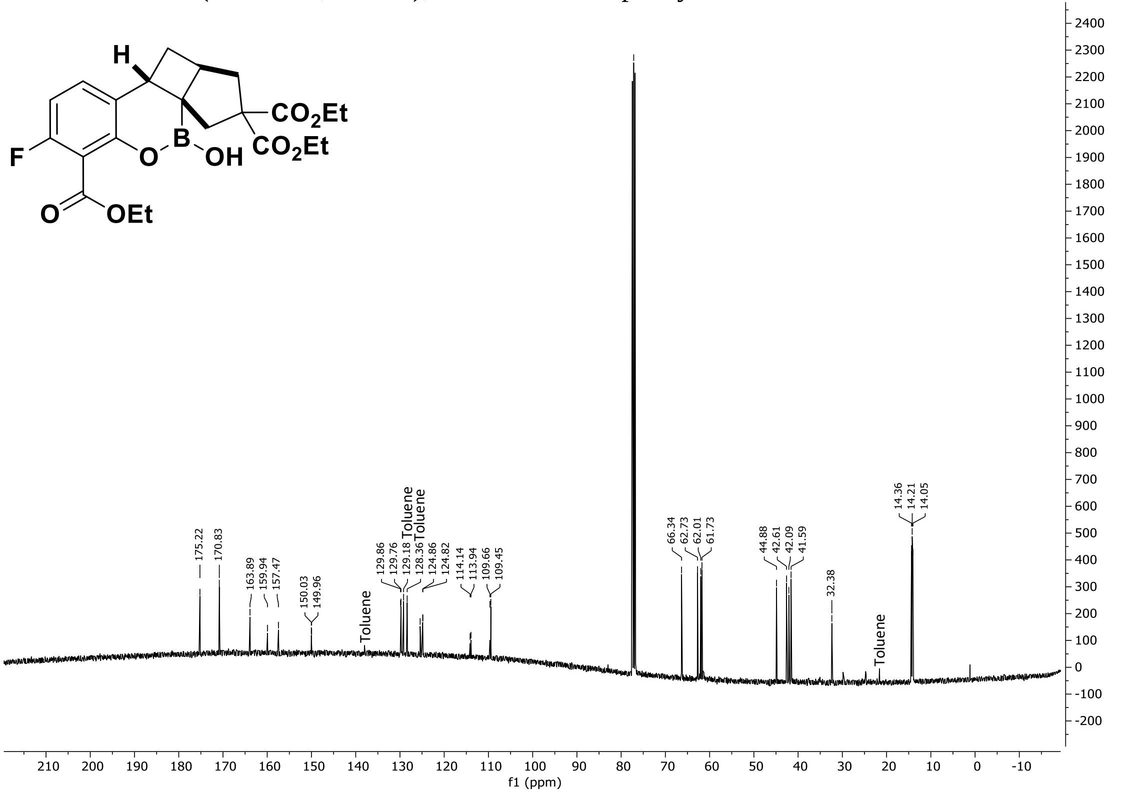

^{11}B-NMR of 41 (128 MHz $CDCl_3$)

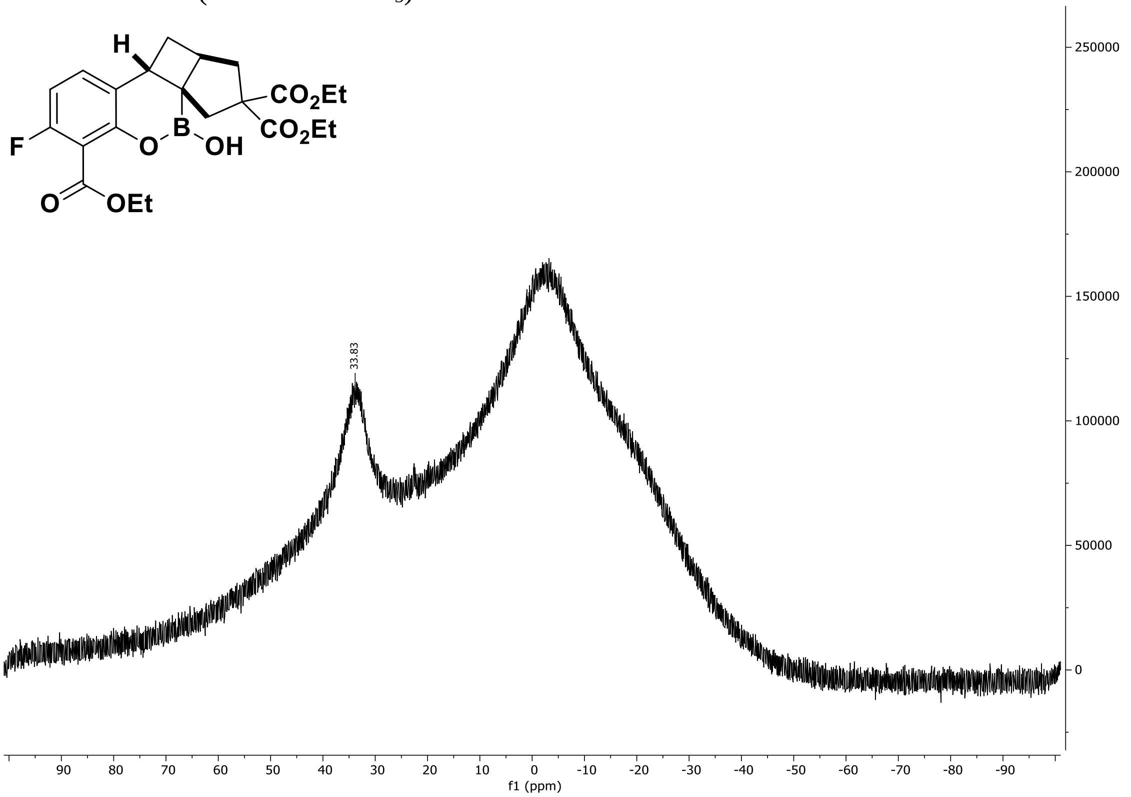

^{19}F-NMR of 41 (376 MHz, $CDCl_3$)

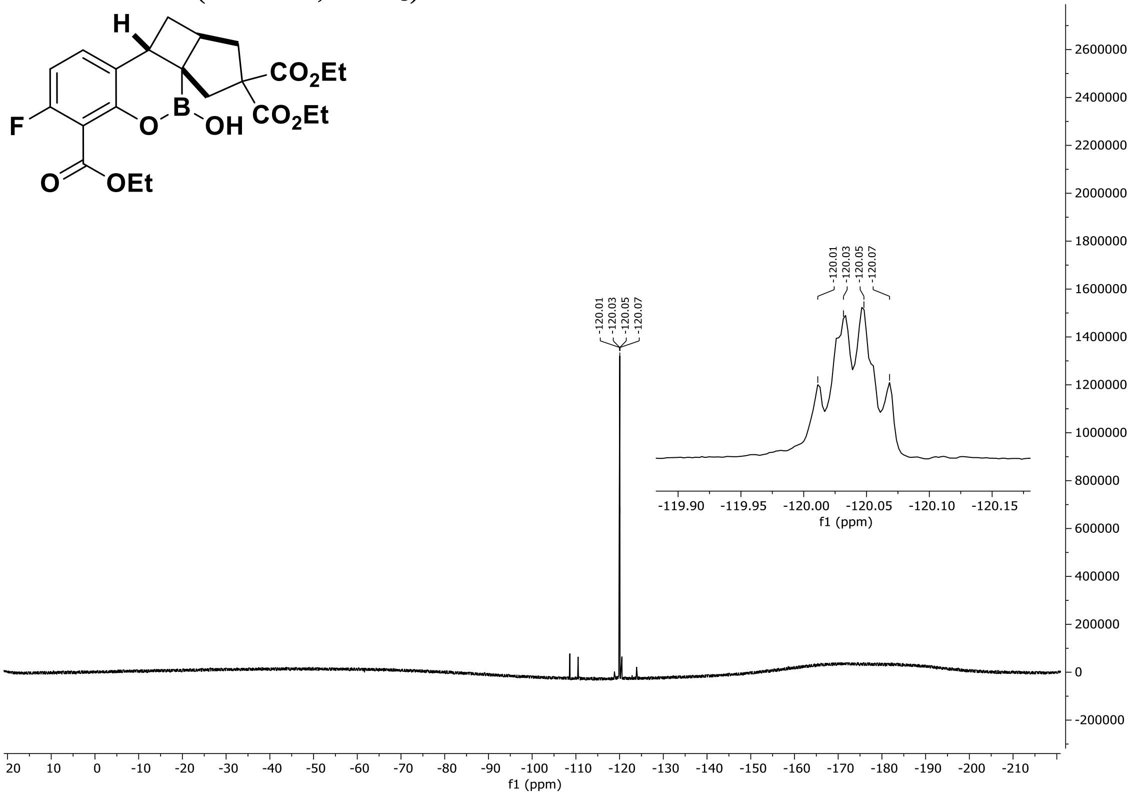

^{1}H-NMR of 42 (400 MHz, $CDCl_3$)

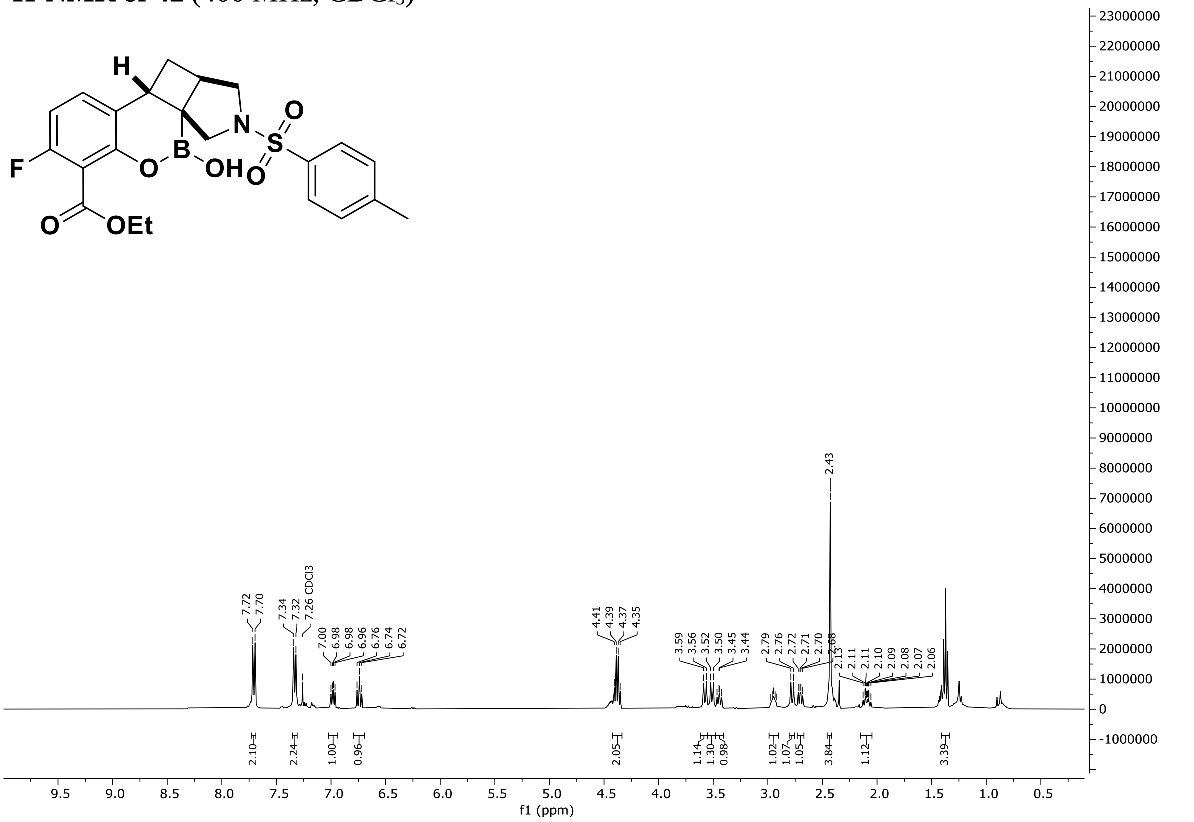

^{13}C-NMR of 42 (100 MHz, $CDCl_3$)

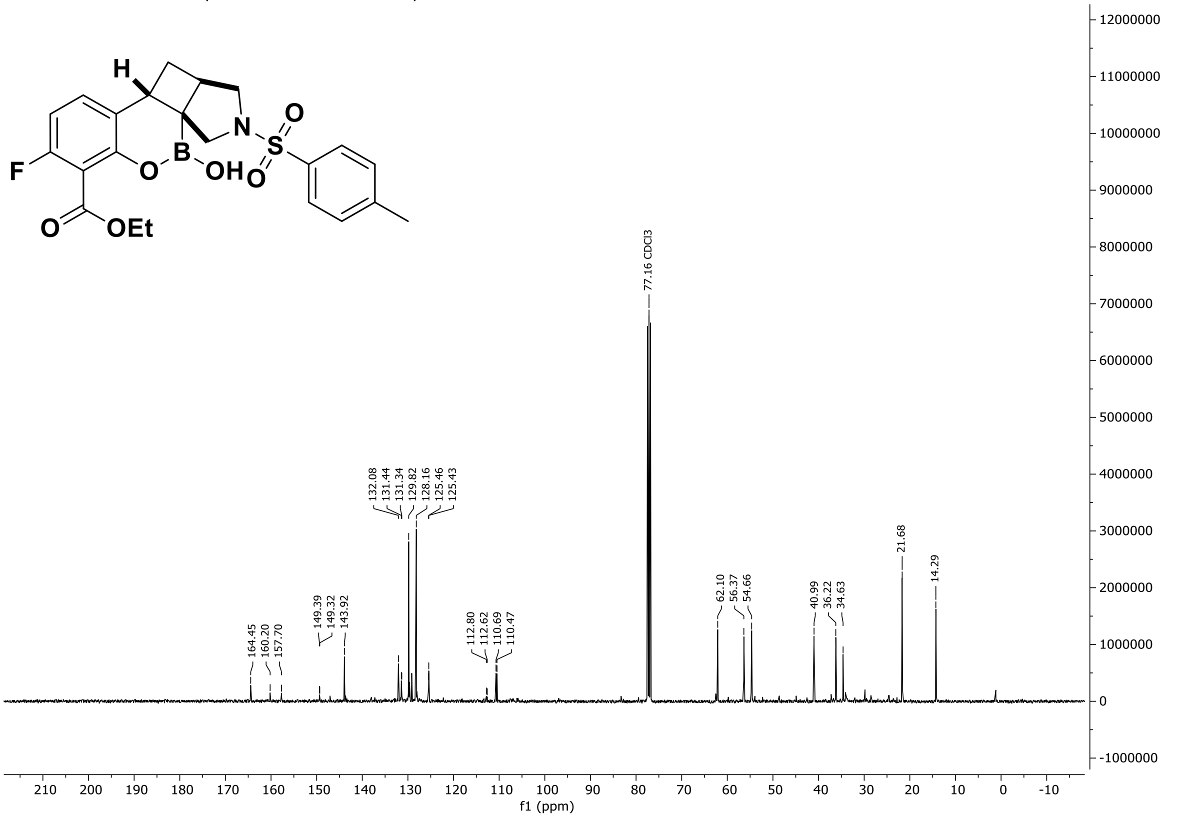

^{19}F-NMR of 42 (376 MHz, $CDCl_3$)

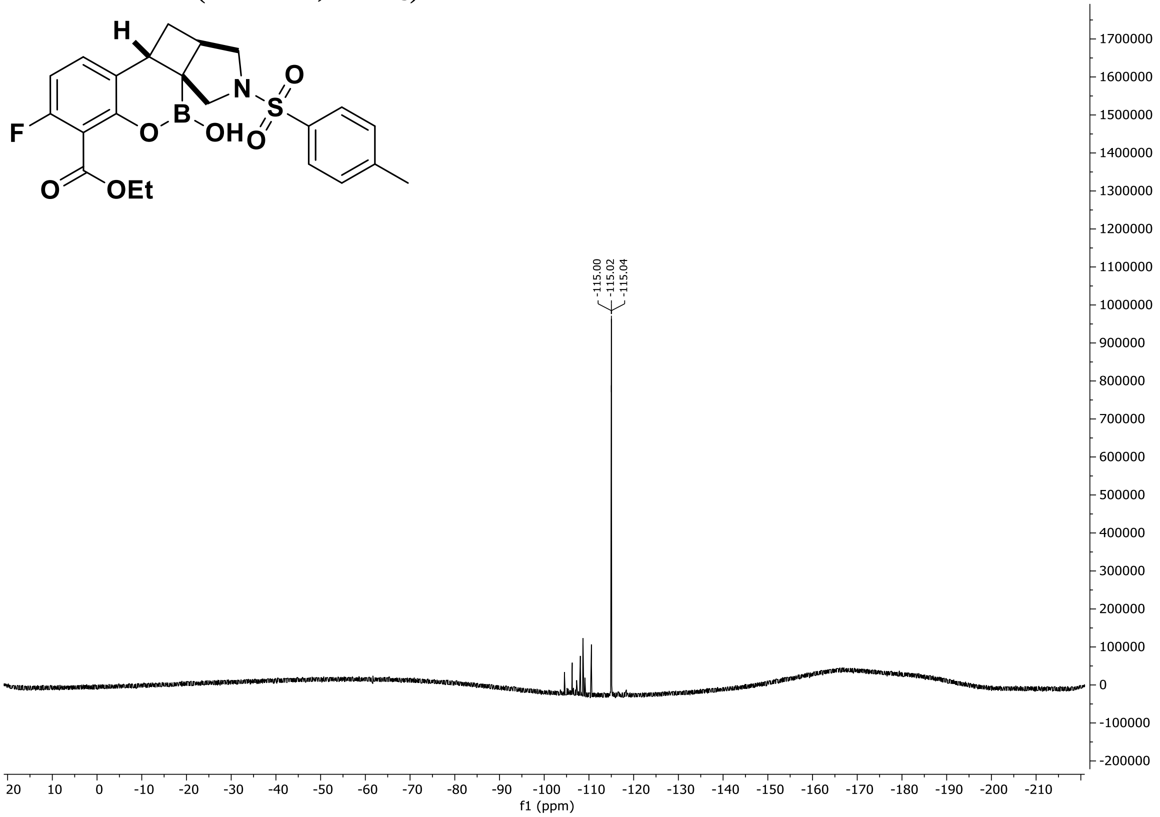

^{11}B-NMR of 42 (128 MHz $CDCl_3$)

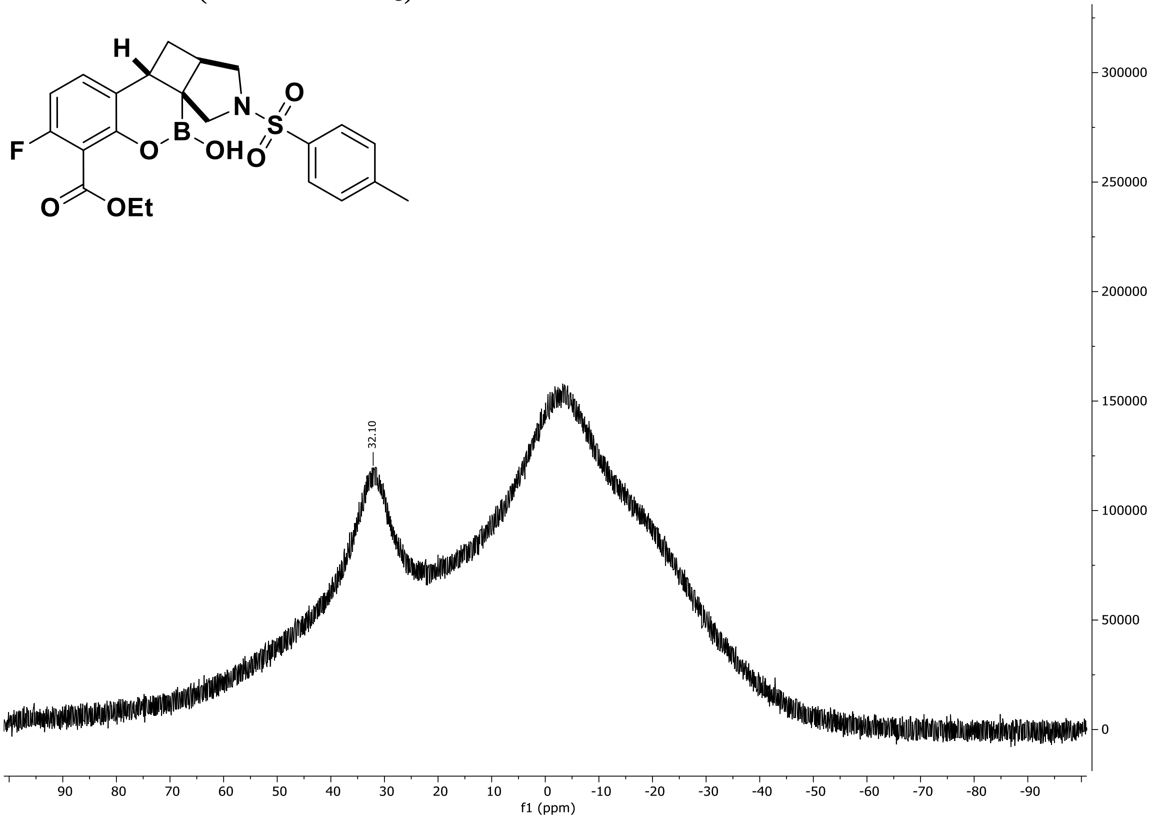

^{1}H-NMR of 43 (400 MHz, $CDCl_3$)

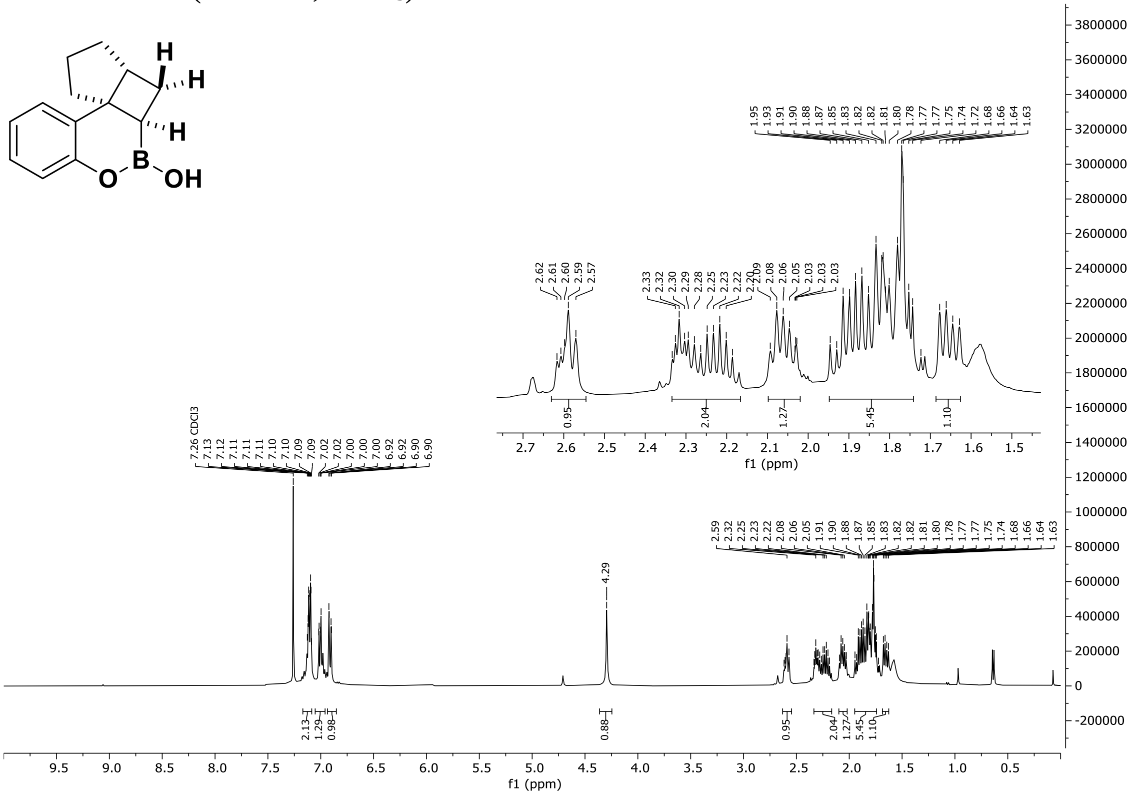

^{13}C-NMR of 43 (100 MHz, $CDCl_3$)

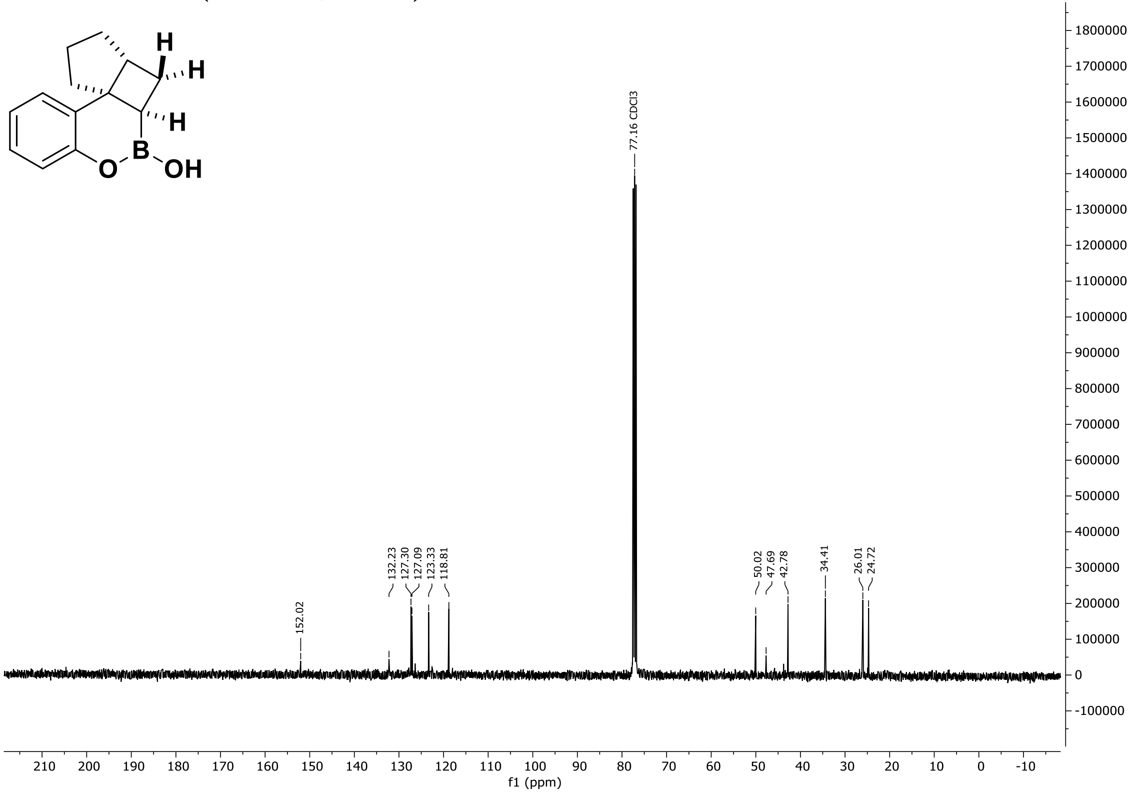

^{11}B-NMR of 43 (128 MHz $CDCl_3$)

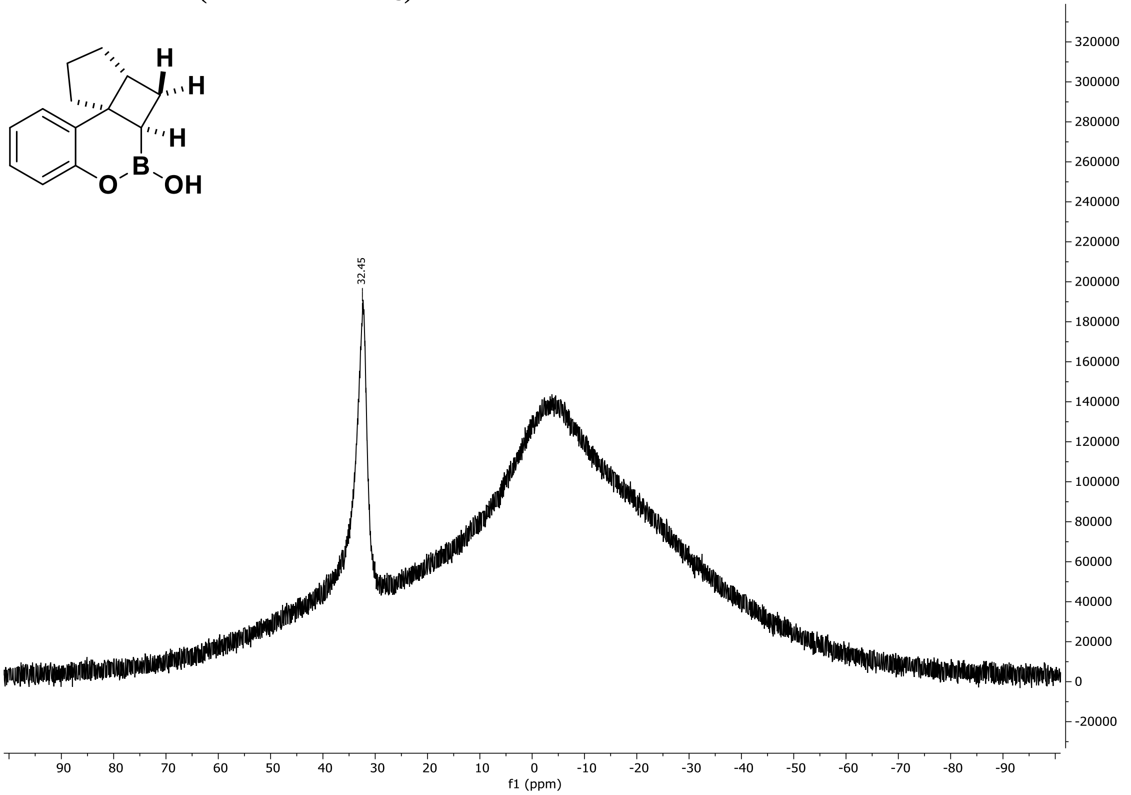

^{1}H-NMR of 3 (400 MHz, $CDCl_3$)

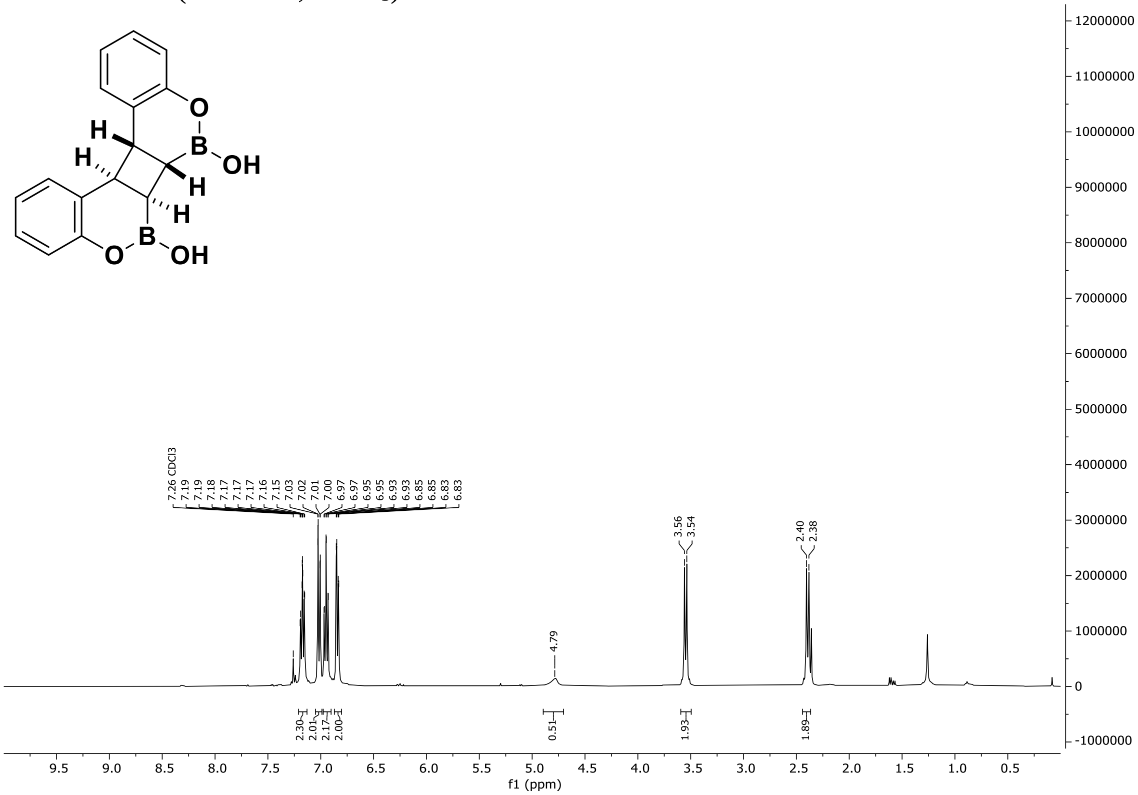

^{13}C-NMR of 3 (100 MHz, $CDCl_3$)

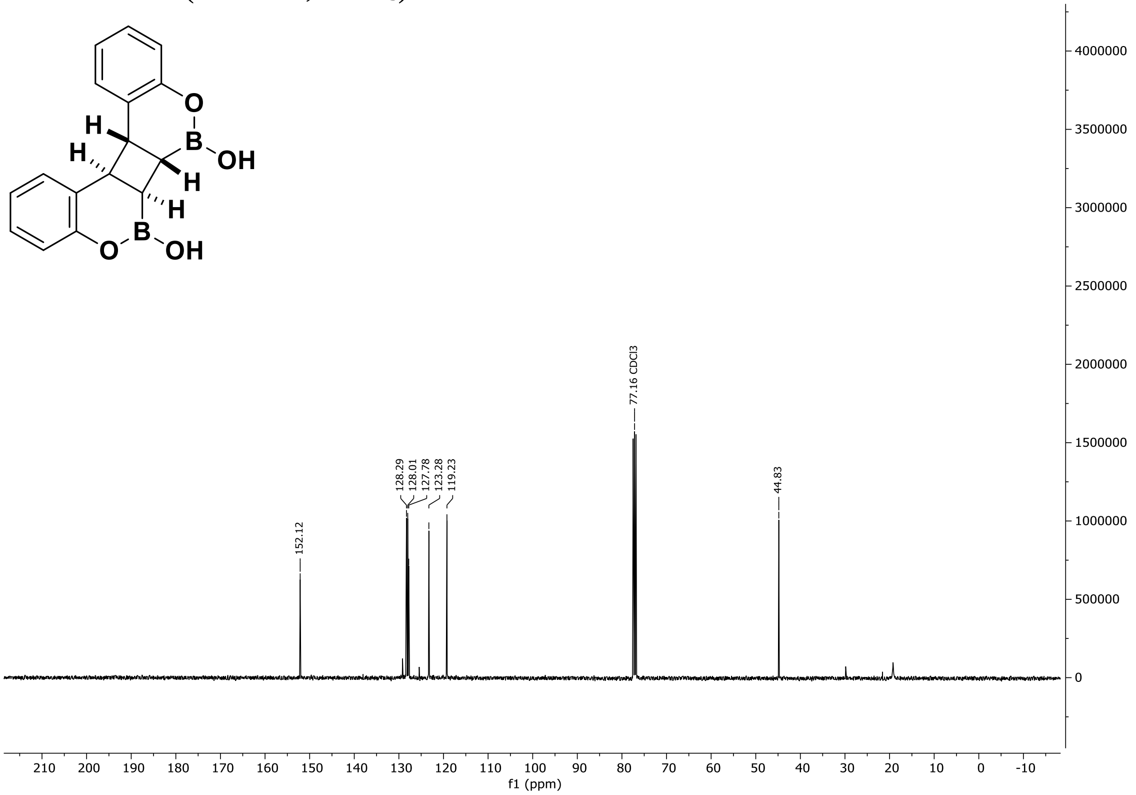

^{11}B-NMR of 3 (128 MHz $CDCl_3$)

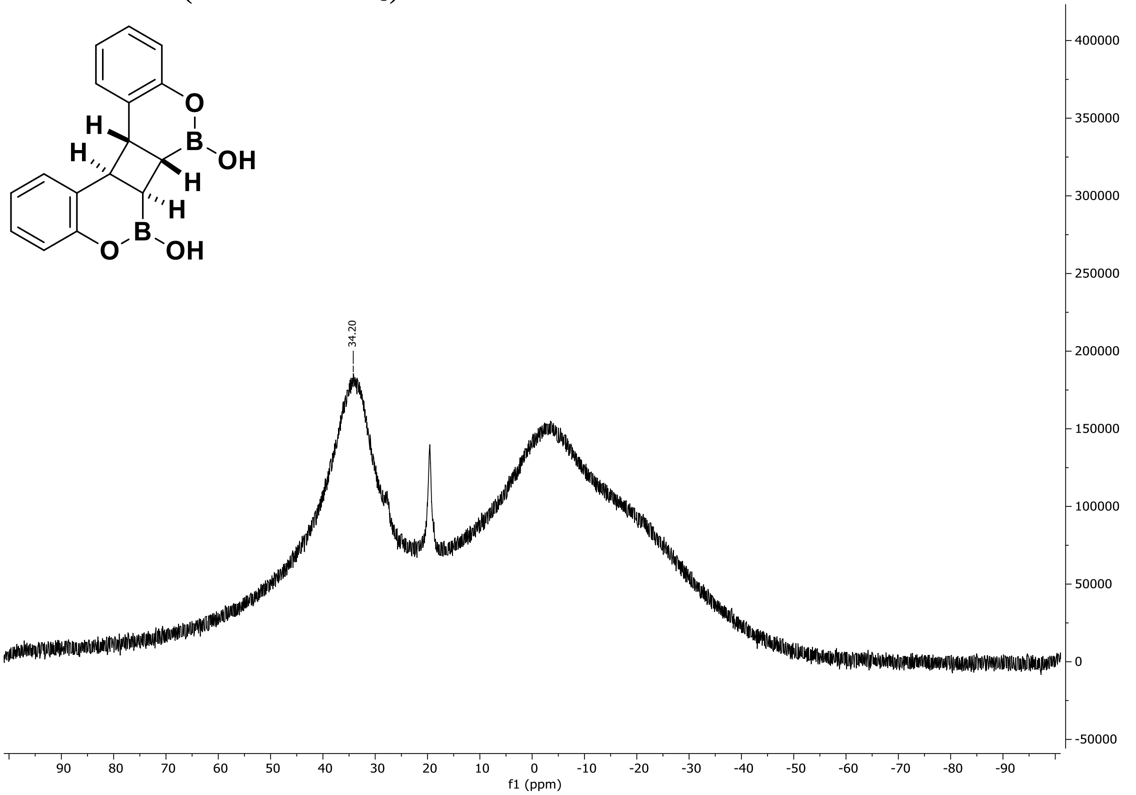

NOESY of 3

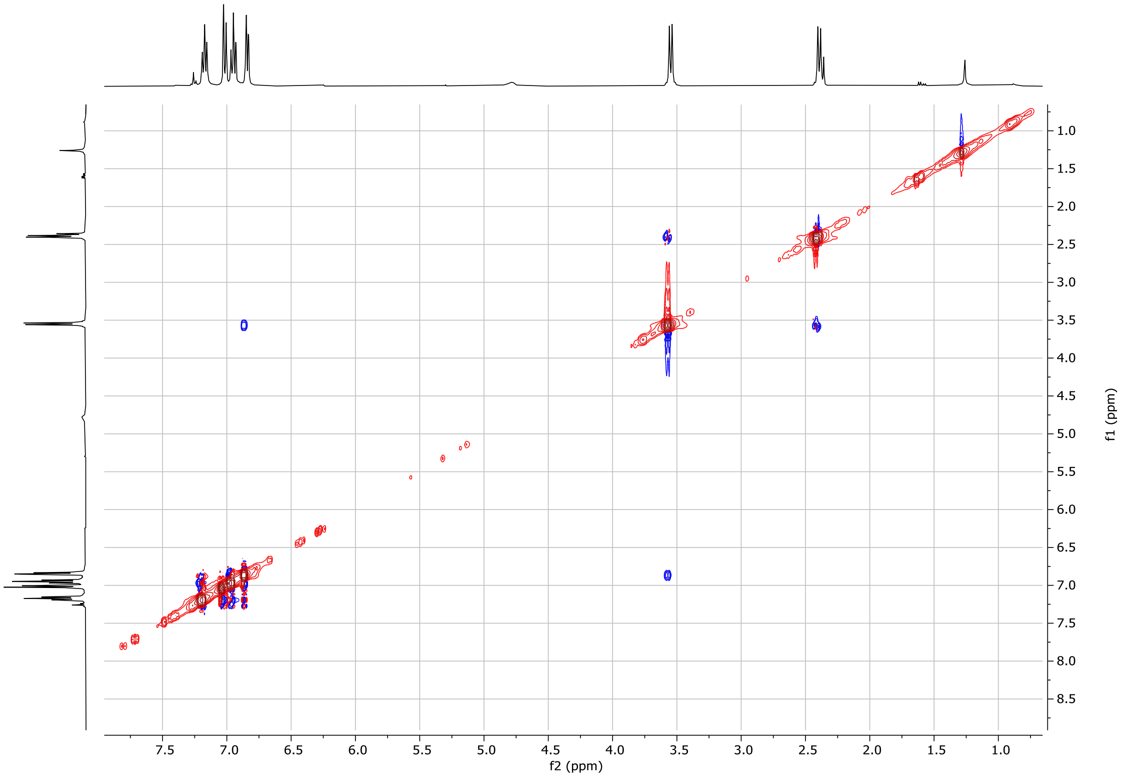

7.5
7.0
6.5
6.0
5.5
5.0
4.5
4.0
3.5
3.0
2.5
2.0
1.5
1.0
f2 (ppm)
1.0
1.5
2.0
2.5
3.0
3.5
4.0
4.5
5.0
5.5
6.0
6.5
7.0
7.5
8.0
8.5
f1 (ppm)
